# Supplementary material for: Construction of C-B axial chirality via dynamic kinetic asymmetric cross-coupling mediated by tetracoordinate boron
Source: Nat Commun. 2023 Jul 24;14:4438. doi: 10.1038/s41467-023-40164-6 (PMC10366327; doi:10.1038/s41467-023-40164-6)
Supplement: Supplementary file 1 — Supplementary Information [file 41467_2023_40164_MOESM1_ESM.pdf]

Supplementary Information

for

Construction of C-B axial chirality via dynamic kinetic asymmetric cross-coupling mediated by tetracoordinate boron

Kai Yang[1,‡], Yanfei Mao[1,‡], Zhihan Zhang[2,‡], Jie Xu[1], Hao Wang[1], Yong He[1], Peiyuan Yu[2]*, Qiuling Song[1,3]*

[1]Key Laboratory of Molecule Synthesis and Function Discovery, Fujian Province University, College of Chemistry at Fuzhou University, Fuzhou, Fujian, 350108, China.

[2]Department of Chemistry and Shenzhen Grubbs Institute, Guangdong Provincial Key Laboratory of Catalysis, Southern University of Science and Technology, Shenzhen, Guangdong 518055, China.

[3]School of Chemistry and Chemical Engineering, Henan Normal University, Xinxiang, Henan, 453007, China.

[‡]These authors contributed equally: Kai Yang, Yanfei Mao, Zhihan Zhang.

E-mail: yupy@sustech.edu.cn; qsong@fzu.edu.cn

Contents

Supplementary Methods 1

1 Computational Investigations..1

1.1 Computational details..1

1.2 Additional computational results..1

2 Experimental Studies 4

2.1 General information 4

2.2 Synthesis of 2,1-borazaronaphthalenes..5

2.3 Synthesis of 3-bromo-2,1-borazaronaphthalenes..10

2.4 General procedure for the synthesis of atropisomers with a single C-B stereogenic axis............14

2.5 General Procedure for the synthesis of atropisomers with adjacent diaxes of C-B and C-C bonds 32

2.6 Synthetic transformations..35

2.7 Mechanism experiments..37

2.8 Rotational barriers of C-B axially chiral compound **3a** 38

2.9 Crystal structure of compound **3a** (CCDC 2245394)..40

2.10 Two-dimensional NMR analysis of **3ai**..41

3 NMR Spectra..45

4 HPLC Spectra 159

5 Supplementary References..205

Supplementary Methods

1 Computational Investigations

1.1 Computational details

All of the calculations were performed using the Gaussian 16 A.03 program.[1] Structures were optimized at the B3LYP level of density functional theory[2] with Grimme's D3 dispersion correction[3] in solution (toluene as solvent), using the CPCM model[4, 5]. For optimizations, Def2SVP was used for all atoms.[6] Conformational searches on the transition states and intermediates were initially performed using Grimme's programs xTB 6.3 and CREST 2.10.2.[7] All the conformers within 3 kcal/mol were further optimized at the level of B3LYP-D3/CPCM/Def2SVP level of theory. Frequency calculations have been performed to verify the optimized structures as local minima or transition states and to obtain Gibbs free energy at 298 K. To reduce error caused by the breakdown of the harmonic oscillator approximation, Truhlar's quasiharmonic correction was used to compute molecular entropies by setting all positive frequencies that are less than 100 cm^{-1} to 100 cm^{-1}.[8] Intrinsic reaction coordinate (IRC) calculations were carried out to make sure that every transition state links relevant intermediates.[9] The electronic energies were further refined by carrying out single-point energy calculations using B3LYP level of density functional theory[2] with Grimme's D3 dispersion correction[3] in solution (toluene as solvent), using the CPCM model[4, 5]. Ahlrichs' triple zeta basis set, Def2TZVP was applied for all atoms in single-point energy calculations.[6, 10] For ECD spectrum simulations, Head-Gordon's long-range corrected (LC) hybrid density functional ωB97X-D was chosen for both excited states calculations and Boltzmann distribution of multiple conformers.[11] Structural optimizations and frequency calculations with the aforementioned quasiharmonic correction to obtain thermal and entropic correction were performed with the ωB97X-D functional with Def2SVP basis set in gas phase. Further refinement of single point energy were performed at the ωB97X-D/Def2TZVP level, using the SMD model[12] (n-hexane as solvent) to account solvation effect. The CD spectrum was calculated by time-dependent density functional theory (TD-DFT, NSTATES=70) at ωB97X-D/Def2TZVP level using SMD (acetonitrile as solvent) model.The three-dimensional (3D) structures were depicted using CYLview software.

1.2 Additional computational results

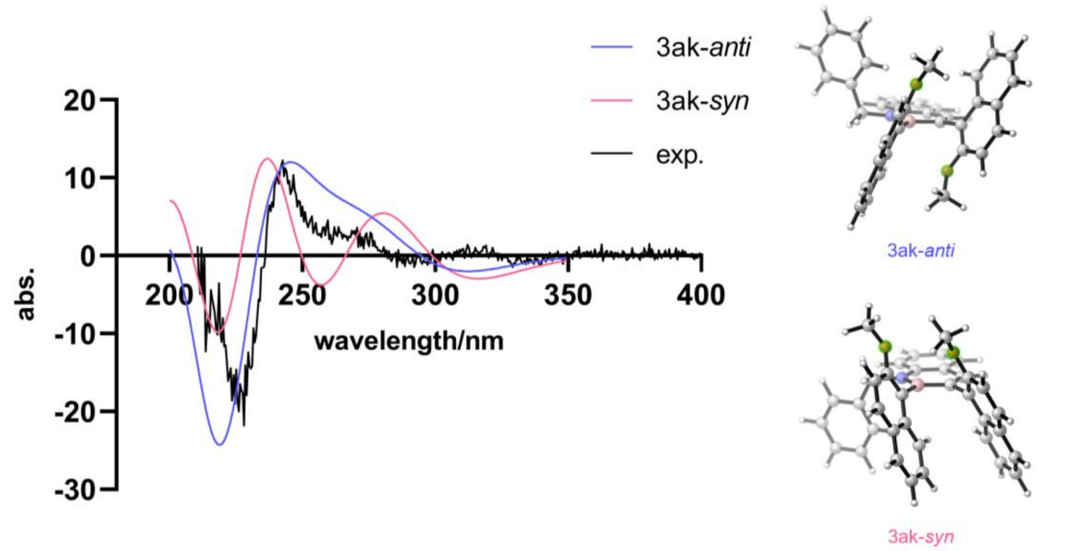

Supplementary Figure 1. Comparison between the simulated ECD-spectrum (blue line for **3ak-*anti*** and pink line for **3ak-*syn***) and experimental ECD-spectrum (black curve) for **3ak**.

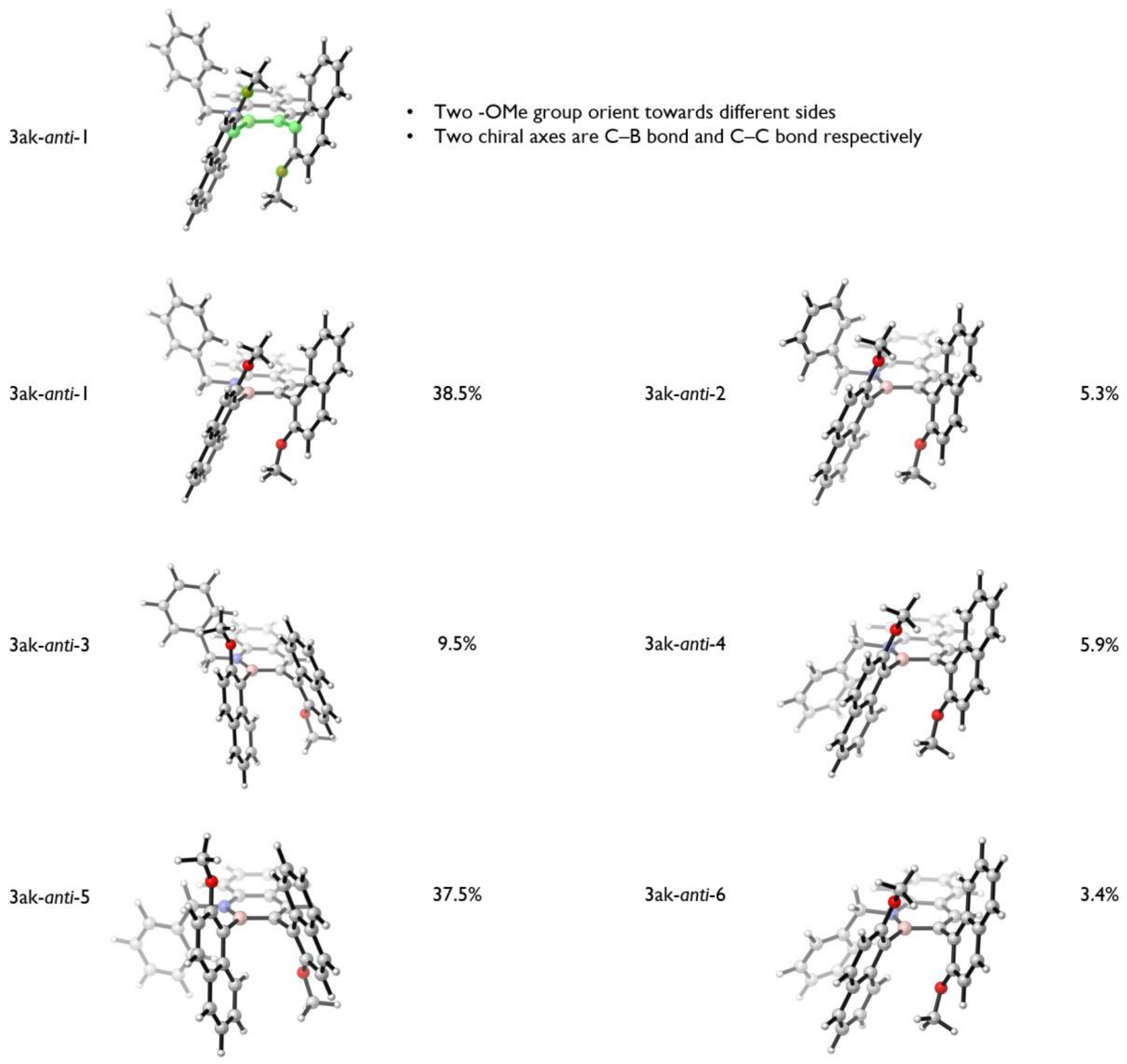

Supplementary Figure 2. 3D geometry structures and Boltzmann distribution of conformers of **3ak-*anti***. Two OMe group orient towards opposite sides. Different conformers differ by the orientations of benzylic group (up and down) and phenyl group of this benzylic group.

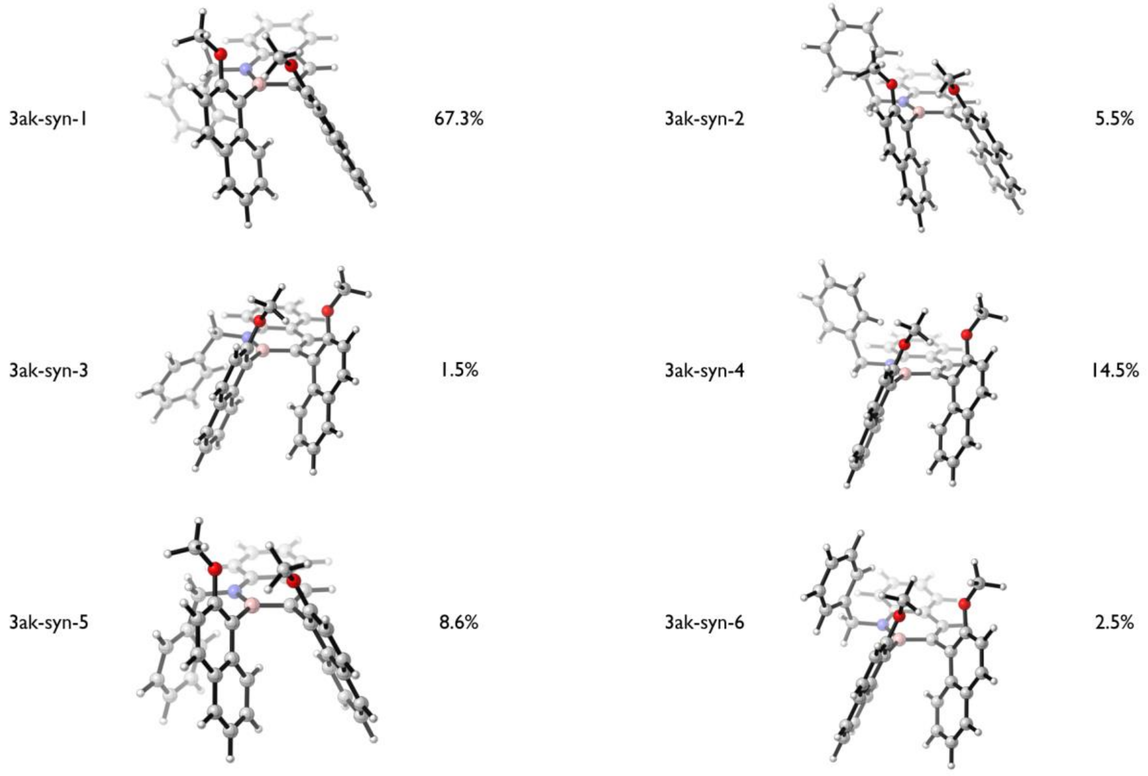

Supplementary Figure 3. 3D geometry structures and Boltzmann distribution of conformers of **3ak-*syn***. Two OMe group orients toward the same side. Different conformers differ by the orientations of benzylic group (up and down) and phenyl group of this benzylic group.

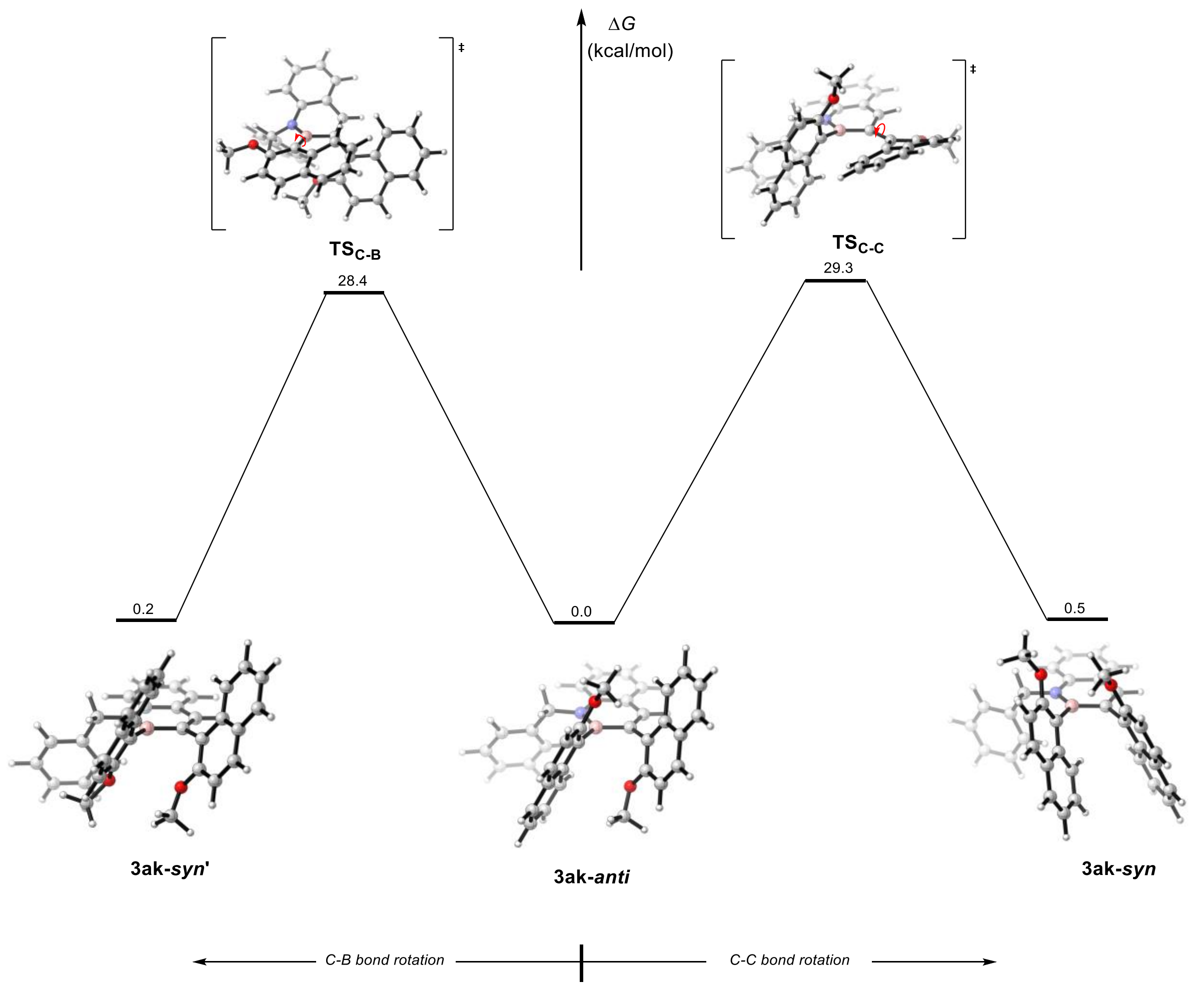

Supplementary Figure 4. Free energy profile for C–C and C–B bond rotations of **3ak** at the level of B3LYP-D3/CPCM/Def2TZVP//B3LYP-D3/CPCM/Def2SVP. Energy are given in kcal/mol.

2 Experimental Studies

2.1 General information

Unless otherwise noted, materials obtained from commercial suppliers were used without further purification. Flash column chromatography was performed over silica gel (200-300 mesh). ^{1}H NMR and ^{13}C NMR spectra were recorded at ambient temperature using Bruker 400M, JEOL 500M and 600 M spectrometers, chemical shifts (in ppm) were referenced to $CDCl_3$ (δ = 7.26 ppm) as internal standards. ^{13}C NMR spectra were obtained by using the same NMR spectrometers and were calibrated with $CDCl_3$ (δ = 77.16 ppm). Data for ^{1}H NMR are recorded as following abbreviations: multiplicity (s = singlet, d = doublet, t = triplet, q = quarter, m = multiplet), coupling constant (*J*, Hz). High resolution mass spectroscopy (HRMS) analysis was performed at an Exactive Plus (Thermo Scientific) or Agilent 8890-7250. HPLC analysis was performed at Waters e2695.

2.2 Synthesis of 2,1-borazaronaphthalenes

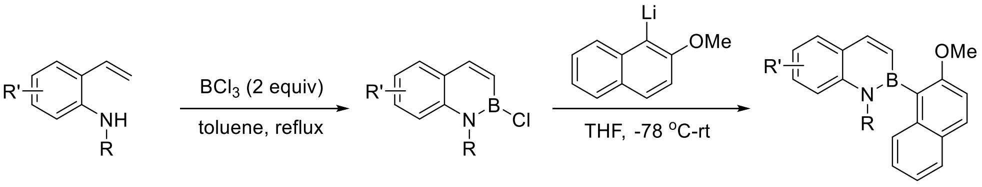

Step 1

In air, a 100 mL schlenk tube was charged with 2-aminostyrene (5 mmol). The tube was evacuated and filled with argon for three cycles, and dry toluene (10 mL) was added under argon. The mixture was cooled to -30 °C, and BCl_3 (10 mmol, 1.0 M in toluene) was slowly added dropwise. After the addition, the mixture was stirred at room temperature for 1 hour. Then, the reaction was continued to reflux for 18 hours. After returning to room temperature, the solvent was removed under reduced pressure and used directly in the next reaction.

Step 2

In argon, the crude product from step 1 is dissolved with THF (5 mL), and the mixture was cooled to -78 °C. Then, aryllithium (1.3 equiv, in THF), prepared from bromoarenes and n-butyllithium, was added to the mixture and stirred at -78 °C for 1 hours. After slowly returning to room temperature, the mixture was quenched by saturated NH_4Cl solution, the aqueous layer was extracted with ethyl acetate. The combined organic layers were dried over anhydrous Na_2SO_4. After removal of the solvent, the crude reaction mixture was purified on silica gel (petroleum ether and ethyl acetate) to afford the corresponding 2,1-borazaronaphthalene.

1-benzyl-2-(2-methoxynaphthalen-1-yl)-1,2-dihydrobenzo[e][1,2]azaborinine (1a-s)

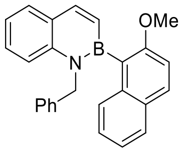

White solid, mp: 105-106 °C. Yield: 58%. R_f = 0.33 (silica gel, PE:EA = 30:1).

^{1}H NMR (500 MHz, Chloroform-*d*) δ 8.14 (d, *J* = 11.3 Hz, 1H), 7.83 (d, *J* = 9.0 Hz, 1H), 7.81 – 7.77 (m, 1H), 7.74 (dd, *J* = 7.8, 1.5 Hz, 1H), 7.54 – 7.49 (m, 1H), 7.40 (d, *J* = 8.5 Hz, 1H), 7.36 – 7.32 (m, 1H), 7.31 – 7.26 (m, 2H), 7.26 – 7.19 (m, 2H), 7.18 – 7.13 (m, 2H), 7.12 – 7.06 (m, 3H), 7.03 (d, *J* = 11.3 Hz, 1H), 5.25 (d, *J* = 16.5 Hz, 1H), 5.20 (d, *J* = 16.5 Hz, 1H), 3.67 (s, 3H). **^{13}C NMR (126 MHz, Chloroform-*d*)** δ 158.6, 144.9, 141.7, 139.2, 136.5, 130.5, 129.8, 129.2, 128.4, 128.3 (2C), 127.7, 127.6, 126.6, 126.4, 126.1, 123.3, 121.0, 117.2, 113.0, 55.9, 53.1. **^{11}B NMR (160 MHz, Chloroform-*d*)** δ 37.08. **HRMS (ESI)** calcd for $C_{26}H_{23}BNO$ $[M+H]^+$: 376.1867, found: 376.1865.

1-benzyl-6-methoxy-2-(2-methoxynaphthalen-1-yl)-1,2-dihydrobenzo[e][1,2]azaborinine (1b-s)

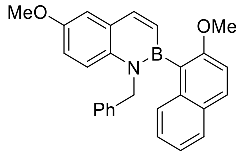

Purple solid, mp: 163-165 °C. Yield: 51%. R_f = 0.2 (silica gel, PE:EA = 30:1).

^{1}H NMR (500 MHz, Chloroform-*d*) δ 8.07 (d, *J* = 11.3 Hz, 1H), 7.82 (d, *J* = 9.0 Hz, 1H), 7.80 – 7.75 (m, 1H), 7.57 – 7.49 (m, 1H), 7.34 – 7.27 (m, 3H), 7.22 (d, *J* = 9.0 Hz, 1H), 7.19 (d, *J* = 3.0 Hz, 1H), 7.18 – 7.12 (m, 2H), 7.12 – 7.06 (m, 3H), 7.02 (d, *J* = 11.3 Hz, 1H), 6.97 (dd, *J* = 9.2, 3.0 Hz, 1H), 5.21 (d, *J* = 16.5 Hz, 1H), 5.16 (d, *J* = 16.5 Hz, 1H), 3.86 (s, 3H), 3.66 (s, 3H). **^{13}C NMR (126 MHz, Chloroform-*d*)** δ 158.6, 153.8, 144.4,

139.3, 136.6, 136.4, 129.7, 129.2, 128.4, 128.3, 127.6, 126.5, 126.4, 126.0, 123.3, 118.3, 117.0, 113.0, 112.0, 55.89, 55.7, 53.2. **^{11}B NMR (160 MHz, Chloroform-*d*)** δ 36.92. **HRMS (ESI)** calcd for $C_{27}H_{25}BNO_2$ $[M+H]^+$: 406.1973, found: 406.1970.

1-benzyl-2-(2-methoxynaphthalen-1-yl)-6-methyl-1,2-dihydrobenzo[e][1,2]azaborinine (1c-s)

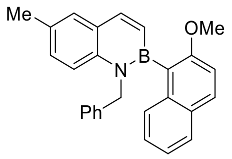

White solid, mp: 78-80 ºC. Yield: 63%. R_f = 0.29 (silica gel, PE:EA = 30:1).

^{1}H NMR (500 MHz, Chloroform-*d*) δ 8.18 (d, *J* = 11.3 Hz, 1H), 7.93 – 7.85 (m, 2H), 7.68 – 7.61 (m, 2H), 7.43 – 7.35 (m, 3H), 7.30 (d, *J* = 9.0 Hz, 1H), 7.28 – 7.22 (m, 3H), 7.22 – 7.15 (m, 3H), 7.13 (d, *J* = 11.3 Hz, 1H), 5.34 (d, *J* = 16.5 Hz, 1H), 5.29 (d, *J* = 16.5 Hz, 1H), 3.74 (s, 3H), 2.50 (s, 3H). **^{13}C NMR (126 MHz, Chloroform-*d*)** δ 158.6, 144.7, 139.7, 139.3, 136.5, 130.2, 129.7, 129.6, 129.2, 128.3, 127.6, 126.5, 126.4, 126.0, 123.3, 117.0, 113.0, 55.9, 53.0, 20.7. **^{11}B NMR (160 MHz, Chloroform-*d*)** δ 37.26. **HRMS (ESI)** calcd for $C_{27}H_{25}BNO$ $[M+H]^+$: 390.2024, found: 390.2023.

1-benzyl-7-fluoro-2-(2-methoxynaphthalen-1-yl)-1,2-dihydrobenzo[e][1,2]azaborinine (1d-s)

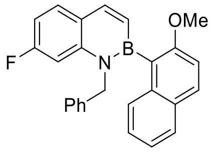

White solid, mp: 165-167 ºC. Yield: 58%. R_f = 0.29 (silica gel, PE:EA = 30:1).

^{1}H NMR (600 MHz, Chloroform-*d*) δ 8.13 (d, *J* = 11.3 Hz, 1H), 7.87 (d, *J* = 8.9 Hz, 1H), 7.85 – 7.81 (m, 1H), 7.72 (dd, *J* = 8.7, 6.6 Hz, 1H), 7.57 – 7.52 (m, 1H), 7.37 – 7.30 (m, 2H), 7.27 (d, *J* = 9.0 Hz, 1H), 7.24 – 7.18 (m, 2H), 7.17 – 7.11 (m, 4H), 7.03 (d, *J* = 11.3 Hz, 1H), 7.00 – 6.95 (m, 1H), 5.26 (d, *J* = 16.5 Hz, 1H), 5.17 (d, *J* = 16.5 Hz, 1H), 3.72 (s, 3H). **^{13}C NMR (151 MHz, Chloroform-*d*)** δ 162.7 (d, *J* = 246.1 Hz), 158.6, 144.3, 143.1 (d, *J* = 10.6 Hz), 138.5, 136.4, 131.9 (d, *J* = 10.6 Hz), 130.0, 129.2, 128.5, 128.4, 127.5, 126.8, 126.4, 126.1, 124.4, 123.4, 112.9, 109.3 (*J* = 22.6 Hz), 103.8 (d, *J* = 25.6 Hz), 55.9, 53.3. **^{11}B NMR (160 MHz, Chloroform-*d*)** δ 37.77. **^{19}F NMR (565 MHz, Chloroform-*d*)** δ -110.63.**HRMS (ESI)** calcd for $C_{26}H_{22}BFNO$ $[M+H]^+$: 394.1773, found: 394.1770.

4-benzyl-3-(2-methoxynaphthalen-1-yl)-3,4-dihydronaphtho[1,2-e][1,2]azaborinine (1e-s)

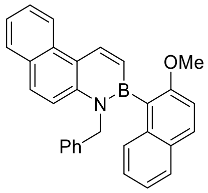

White solid, mp: > 250 ºC. Yield: 5%. R_f = 0.25 (silica gel, PE:EA = 30:1).

^{1}H NMR (500 MHz, Chloroform-*d*) δ 9.20 (d, *J* = 11.7 Hz, 1H), 8.69 (d, *J* = 8.5 Hz, 1H), 7.84 (m, 3H), 7.77 (d, *J* = 9.3 Hz, 1H), 7.69 – 7.63 (m, 2H), 7.61 – 7.55 (m, 1H), 7.50 (t, *J* = 7.4 Hz, 1H), 7.35 – 7.23 (m, 4H), 7.20 – 7.14 (m, 2H), 7.14 – 7.07 (m, 3H), 5.42 (d, *J* = 17.7 Hz, 1H), 5.38 (d, *J* = 17.7 Hz, 1H), 3.66 (s, 3H). **^{13}C NMR (126 MHz, Chloroform-*d*)** δ 158.7, 140.3, 139.4, 138.9, 136.7, 131.7, 129.8, 129.2, 129.2, 128.9, 128.5, 128.4,

128.3, 127.7, 127.0, 126.6, 126.4, 126.0, 124.6, 123.3, 122.2, 121.2, 117.9, 113.0, 55.9, 53.7. **^{11}B NMR (160 MHz, Chloroform-*d*)** δ 37.05. **HRMS (ESI)** calcd for $C_{30}H_{25}BNO$ $[M+H]^+$: 426.2024, found: 426.2026.

2-(2-methoxynaphthalen-1-yl)-1-(4-methylbenzyl)-1,2-dihydrobenzo[e][1,2]azaborinine (1f-s)

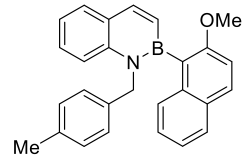

White solid, mp: 158-159 °C. Yield: 48%. R_f = 0.4 (silica gel, PE:EA = 30:1).

^{1}H NMR (500 MHz, Chloroform-*d*) δ 8.18 (d, *J* = 11.3 Hz, 1H), 7.87 (d, *J* = 9.0 Hz, 1H), 7.85 – 7.81 (m, 1H), 7.78 (dd, *J* = 7.8, 1.5 Hz, 1H), 7.63 – 7.55 (m, 1H), 7.48 (d, *J* = 8.5 Hz, 1H), 7.43 – 7.37 (m, 1H), 7.36 – 7.31 (m, 2H), 7.28 (d, *J* = 9.1 Hz, 1H), 7.26 – 7.21 (m, 1H), 7.08 (d, *J* = 11.3 Hz, 1H), 7.05 (d, *J* = 8.2 Hz, 2H), 7.01 (d, *J* = 8.1 Hz, 2H), 5.27 (d, *J* = 16.3 Hz, 1H), 5.22 (d, *J* = 16.3 Hz, 1H), 3.74 (s, 3H), 2.27 (s, 3H). **^{13}C NMR (126 MHz, Chloroform-*d*)** δ 158.6, 144.9, 141.7, 136.5, 136.2, 136.0, 130.4, 129.8, 129.2, 129.1, 128.3, 128.3, 127.7, 127.6, 126.3, 126.0, 123.3, 121.0, 117.2, 113.0, 56.0, 52.9, 21.1. **^{11}B NMR (160 MHz, Chloroform-*d*)** δ 37.66. **HRMS (ESI)** calcd for $C_{27}H_{25}BNO$ $[M+H]^+$: 390.2024, found: 390.2022.

1-(4-fluorobenzyl)-2-(2-methoxynaphthalen-1-yl)-1,2-dihydrobenzo[e][1,2]azaborinine (1g-s)

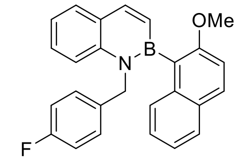

White solid, mp: 122-124 °C. Yield: 61%. R_f = 0.36 (silica gel, PE:EA = 30:1).

^{1}H NMR (500 MHz, Chloroform-*d*) δ 8.18 (d, *J* = 11.3 Hz, 1H), 7.88 (d, *J* = 9.0 Hz, 1H), 7.86 – 7.82 (m, 1H), 7.79 (d, *J* = 7.5 Hz, 1H), 7.57 – 7.52 (m, 1H), 7.43 – 7.38 (m, 2H), 7.36 – 7.31 (m, 2H), 7.29 – 7.23 (m, 2H), 7.12 – 7.05 (m, 3H), 6.92 – 6.85 (m, 2H), 5.26 (d, *J* = 16.4 Hz, 1H), 5.20 (d, *J* = 16.4 Hz, 1H), 3.71 (s, 3H). **^{13}C NMR (126 MHz, Chloroform-*d*)** δ 161.6 (d, *J* = 244.4 Hz), 158.6, 145.0, 141.5, 136.4, 134.7, 130.6, 129.9, 129.20, 128.4, 127.9 (d, *J* = 7.6 Hz), 127.7, 127.5, 126.1, 123.4, 121.2, 117.0, 115.2 (d, *J* = 21.4 Hz), 112.9, 55.9, 52.3. **^{11}B NMR (160 MHz, Chloroform-*d*)** δ 37.49. **^{19}F NMR (471 MHz, Chloroform-*d*)** δ -116.47. **HRMS (ESI)** calcd for $C_{26}H_{22}BFNO$ $[M+H]^+$: 394.1773, found: 394.1775.

1-(4-chlorobenzyl)-2-(2-methoxynaphthalen-1-yl)-1,2-dihydrobenzo[e][1,2]azaborinine (1h-s)

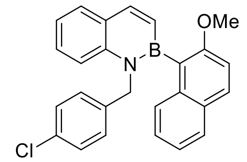

White solid, mp: 134-136 °C. Yield: 44%. R_f = 0.38 (silica gel, PE:EA = 30:1).

^{1}H NMR (500 MHz, Chloroform-*d*) δ 8.17 (d, *J* = 11.3 Hz, 1H), 7.87 (d, *J* = 9.0 Hz, 1H), 7.85 – 7.81 (m, 1H), 7.78 (dd, *J* = 7.7, 1.3 Hz, 1H), 7.54 – 7.49 (m, 1H), 7.42 – 7.36 (m, 2H), 7.36 – 7.30 (m, 2H), 7.29 – 7.22 (m, 2H), 7.20 – 7.14 (m, 2H) , 1H, 7.10 – 7.03 (m, 3H), 5.24 (d, *J* = 16.6 Hz, 1H), 5.18 (d, *J* = 16.6 Hz, 1H), 3.70 (s, 3H). **^{13}C NMR (126 MHz, Chloroform-*d*)** δ 158.6, 145.0, 141.4, 137.7, 136.4, 132.2, 130.6, 129.9, 129.2, 128.5, 128.4, 127.8, 127.7, 127.5, 126.1, 123.4, 121.2, 116.9, 112.9, 55.9, 52.4. **^{11}B NMR (160 MHz, Chloroform-*d*)** δ 37.75. **HRMS (ESI)** calcd for $C_{26}H_{22}BClNO$ $[M+H]^+$: 410.1477, found: 410.1479.

1-butyl-2-(2-methoxynaphthalen-1-yl)-1,2-dihydrobenzo[e][1,2]azaborinine (1i-s)

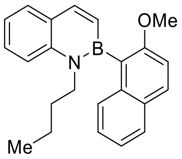

Light purple oil. Yield: 62%. R_f = 0.4 (silica gel, PE:EA = 30:1).

^{1}H NMR (500 MHz, Chloroform-*d*) δ 8.16 (d, *J* = 11.2 Hz, 1H), 7.96 (d, *J* = 8.9 Hz, 1H), 7.93 – 7.89 (m, 1H), 7.83 (dd, *J* = 7.8, 1.6 Hz, 1H), 7.73 (d, *J* = 8.6 Hz, 1H), 7.66 – 7.60 (m, 1H), 7.57 – 7.52 (m, 1H), 7.42 – 7.31 (m, 4H), 7.03 (d, *J* = 11.2 Hz, 1H), 4.14 – 4.06 (m, 1H), 4.04 – 3.96 (m, 1H), 3.91 (s, 3H), 1.86 – 1.67 (m, 2H), 1.24 – 1.14 (m, 2H), 0.72 (t, *J* = 7.4 Hz, 3H). **^{13}C NMR (126 MHz, Chloroform-*d*)** δ 158.2, 144.4, 141.6, 136.5, 130.7, 129.6, 129.3, 128.3, 128.3, 127.8, 127.6, 125.8, 123.3, 120.7, 115.6, 113.2, 56.2, 48.7, 31.7, 20.2, 13.7. **^{11}B NMR (160 MHz, Chloroform-*d*)** δ 36.85. **HRMS (ESI)** calcd for $C_{23}H_{25}BNO$ $[M+H]^+$: 342.2024, found: 342.2022.

2-(2-methoxynaphthalen-1-yl)-1-(thiophen-2-ylmethyl)-1,2-dihydrobenzo[e][1,2]azaborinine (1j-s)

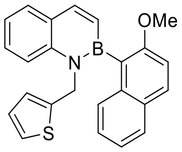

Light yellow oil. Yield: 50%. R_f = 0.33 (silica gel, PE:EA = 30:1).

^{1}H NMR (500 MHz, Chloroform-*d*) δ 8.16 (d, *J* = 11.0 Hz, 1H), 7.91 (d, *J* = 8.9 Hz, 1H), 7.87 – 7.83 (m, 1H), 7.79 (dd, *J* = 7.8, 1.6 Hz, 1H), 7.70 (d, *J* = 8.6 Hz, 1H), 7.59 – 7.54 (m, 1H), 7.51 – 7.45 (m, 1H), 7.37 – 7.31 (m, 1H), 7.30 – 7.26 (m, 1H), 7.09 – 7.02 (m, 1H), 6.81 (dd, *J* = 5.0, 3.5 Hz, 1H), 6.77 – 6.74 (m, 1H), 5.46 (d, *J* = 16.3 Hz, 1H), 5.41 (d, *J* = 16.2 Hz, 1H), 3.79 (s, 3H). **^{13}C NMR (126 MHz, Chloroform-*d*)** δ 158.5, 145.0, 142.8, 141.5, 136.6, 130.6, 130.0, 129.2, 128.4, 128.3, 127.7, 127.6, 126.6, 126.0, 124.8, 123.8, 123.4, 121.2, 116.5, 113.0, 56.0, 48.4. **^{11}B NMR (160 MHz, Chloroform-*d*)** δ 38.58. **HRMS (ESI)** calcd for $C_{24}H_{21}BNOS$ $[M+H]^+$: 382.1431, found: 382.1429.

1-isopropyl-2-(2-methoxynaphthalen-1-yl)-1,2-dihydrobenzo[e][1,2]azaborinine (1k-s)

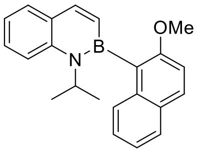

White solid, mp: 54-56 °C. Yield: 34%. R_f = 0.36 (silica gel, PE:EA = 30:1).

^{1}H NMR (500 MHz, Chloroform-*d*) δ 8.05 (d, *J* = 11.2 Hz, 1H), 7.94 (d, *J* = 8.6 Hz, 1H), 7.90 (d, *J* = 9.0 Hz, 1H), 7.87 – 7.83 (m, 1H), 7.77 (dd, *J* = 7.8, 1.6 Hz, 1H), 7.56 – 7.46 (m, 2H), 7.37 – 7.23 (m, 4H), 6.86 (d, *J* = 11.2 Hz, 1H), 4.89 – 4.77 (m, 1H), 3.85 (s, 3H), 1.68 (s, 3H), 1.53 (d, *J* = 7.2 Hz, 3H). **^{13}C NMR (126 MHz, Chloroform-*d*)** δ 157.8, 144.7, 136.4, 131.2, 129.3, 128.74, 128.2, 128.0, 127.2, 125.8, 123.4, 120.4, 118.8, 113.1, 56.2, 52.9, 21.9. **^{11}B NMR (160 MHz, Chloroform-*d*)** δ 37.03. **HRMS (ESI)** calcd for $C_{22}H_{23}BNO$ $[M+H]^+$: 328.1867, found: 328.1864.

1-benzyl-2-(2-methoxy-6-methylnaphthalen-1-yl)-1,2-dihydrobenzo[e][1,2]azaborinine (1l-s)

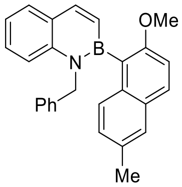

White solid, mp: 170-171 °C. Yield: 48%. R_f = 0.34 (silica gel, PE:EA = 30:1).

^{1}H NMR (500 MHz, Chloroform-*d*) δ 8.16 (d, *J* = 11.3 Hz, 1H), 7.79 – 7.74 (m, 2H), 7.59 (s, 1H), 7.48 – 7.40 (m, 2H), 7.39 – 7.34 (m, 1H), 7.25 – 7.15 (m, 5H), 7.14 – 7.10 (m, 3H), 7.06 (d, *J* = 11.3 Hz, 1H), 5.28 (d, *J* = 16.7 Hz, 1H), 5.23 (d, *J* = 16.5 Hz, 1H), 3.67 (s, 3H), 2.48 (s, 3H). **^{13}C NMR (126 MHz, Chloroform-*d*)** δ 158.1, 144.8, 141.7, 139.2, 134.6, 132.7, 130.5, 129.4, 129.1, 128.4, 128.4, 128.3, 127.7, 127.4, 127.3, 126.5, 126.4, 121.0, 117.2, 113.1, 55.9, 53.1, 21.6. **^{11}B NMR (160 MHz, Chloroform-*d*)** δ 38.00. **HRMS (ESI)** calcd for $C_{27}H_{25}BNO$ $[M+H]^+$: 390.2024, found: 390.2020.

1-benzyl-2-(2-ethoxynaphthalen-1-yl)-1,2-dihydrobenzo[e][1,2]azaborinine (1m-s)

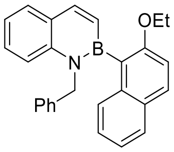

White solid, mp: 56-58 °C. Yield: 60%. R_f = 0.33 (silica gel, PE:EA = 30:1).

^{1}H NMR (500 MHz, Chloroform-*d*) δ 8.12 (d, *J* = 11.3 Hz, 1H), 7.84 – 7.77 (m, 2H), 7.74 (dd, *J* = 7.8, 1.4 Hz, 1H), 7.55 – 7.48 (m, 1H), 7.42 (d, *J* = 8.5 Hz, 1H), 7.38 – 7.32 (m, 1H), 7.31 – 7.27 (m, 2H), 7.26 – 7.19 (m, 2H), 7.18 – 7.07 (m, 5H), 7.03 (d, *J* = 11.3 Hz, 1H), 5.28 (d, *J* = 16.4 Hz, 1H), 5.22 (d, *J* = 16.4 Hz, 1H), 4.15 – 3.98 (m, 2H), 1.24 (t, *J* = 7.0 Hz, 3H). **^{13}C NMR (126 MHz, Chloroform-*d*)** δ 158.0, 144.6, 141.6, 139.3, 136.5, 130.4, 129.7, 129.2, 128.4, 128.4, 128.2, 127.8, 127.5, 126.6, 126.5, 126.0, 123.3, 123.0, 117.2, 114.3, 64.3, 53.2, 15.2. **^{11}B NMR (160 MHz, Chloroform-*d*)** δ 37.84. **HRMS (ESI)** calcd for $C_{27}H_{25}BNO$ $[M+H]^+$: 390.2024, found: 390.2023.

1-benzyl-2-(2-(ethylthio)naphthalen-1-yl)-1,2-dihydrobenzo[e][1,2]azaborinine (1n-s)

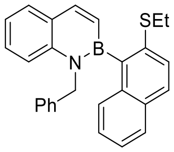

White solid, mp: 143-144 °C. Yield: 75%. R_f = 0.35 (silica gel, PE:EA = 30:1).

^{1}H NMR (500 MHz, Chloroform-*d*) δ 8.16 (d, *J* = 11.3 Hz, 1H), 7.83 – 7.75 (m, 3H), 7.53 (d, *J* = 8.6 Hz, 2H), 7.43 – 7.34 (m, 3H), 7.33 – 7.28 (m, 1H), 7.25 – 7.21 (m, 1H), 7.19 – 7.13 (m, 2H), 7.12 – 7.07 (m, 3H), 7.02 (d, *J* = 11.3 Hz, 1H), 5.24 (d, *J* = 16.6 Hz, 1H), 5.20 (d, *J* = 16.6 Hz, 1H), 3.01 – 2.92 (m, 1H), 2.87 – 2.78 (m, 1H), 1.23 (t, *J* = 7.4 Hz, 3H). **^{13}C NMR (126 MHz, Chloroform-*d*)** δ 144.9, 141.5, 138.7, 136.0, 135.6, 131.8, 130.5, 128.5, 128.4, 128.4, 128.3, 127.7, 127.6, 126.7, 126.4, 126.1, 125.5, 121.2, 117.4, 53.1, 29.3, 14.8. **^{11}B NMR (160 MHz, Chloroform-*d*)** δ 37.04. **HRMS (ESI)** calcd for $C_{27}H_{25}BNS$ $[M+H]^+$: 406.1795, found: 406.1794.

2.3 Synthesis of 3-bromo-2,1-borazaronaphthalenes

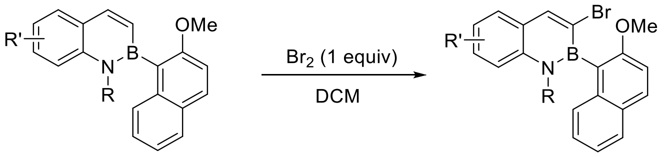

In argon, the 2,1-borazaronaphthalene from step 2 is dissolved with DCM (25 mL), and the mixture was cooled to -20 ºC. Then, Br_2 (1.0 equiv) in DCM (25ml) was slowly added dropwise. Upon completion, the reaction was quenched by saturated $Na_2S_2O_3$ solution, the aqueous layer was extracted with DCM. The combined organic layers were dried over anhydrous Na_2SO_4. the crude reaction mixture was purified on silica gel (petroleum ether and ethyl acetate) to afford the corresponding 3-bromo-2,1-borazaronaphthalene.

1-benzyl-3-bromo-2-(2-methoxynaphthalen-1-yl)-1,2-dihydrobenzo[e][1,2]azaborinine (1a)

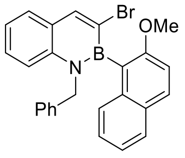

White solid, mp: 112-114 ºC. Yield: 60%. R_f = 0.4 (silica gel, PE:DCM = 5:1).

^{1}H NMR (400 MHz, Chloroform-*d*) δ 8.51 (s, 1H), 7.92 (d, *J* = 9.0 Hz, 1H), 7.89 – 7.82 (m, 1H), 7.73 (d, *J* = 7.6 Hz, 1H), 7.48 – 7.40 (m, 3H), 7.39 – 7.33 (m, 2H), 7.32 – 7.26 (m, 2H), 7.22 (dd, *J* = 7.8, 6.4 Hz, 2H), 7.19 – 7.11 (m, 3H), 5.31 (d, *J* = 16.5 Hz, 1H), 5.23 (d, *J* = 16.4 Hz, 1H), 3.77 (s, 3H). **^{13}C NMR (101 MHz, Chloroform-*d*)** δ 159.0, 145.6, 140.6, 138.4, 135.9, 130.4, 129.8, 129.1, 128.7, 128.5, 128.5, 127.1, 126.9, 126.8, 126.4, 126.3, 123.5, 121.9, 117.5, 112.9, 56.0, 54.1. **^{11}B NMR (128 MHz, Chloroform-*d*)** δ 38.5. **HRMS (ESI)** calcd for $C_{26}H_{22}BBrNO$ $[M+H]^+$: 454.0972, found: 454.0970.

1-benzyl-3-bromo-2-(2-methoxynaphthalen-1-yl)-6-methyl-1,2-dihydrobenzo[e][1,2]azaborinine (1b)

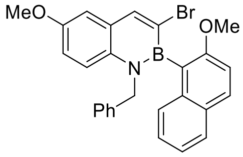

White solid, mp: 72-74ºC. Yield: 56%. R_f = 0.4 (silica gel, PE:DCM = 5:1)

^{1}H NMR (500 MHz, Chloroform-*d*) δ 8.42 (s, 1H), 7.87 (d, *J* = 9.0 Hz, 1H), 7.84 – 7.79 (m, 1H), 7.47 – 7.39 (m, 1H), 7.35 – 7.29 (m, 3H), 7.27 – 7.24 (m, 1H), 7.19 (dd, *J* = 7.9, 6.7 Hz, 2H), 7.15 – 7.08 (m, 4H), 7.01 (dd, *J* = 9.3, 3.0 Hz, 1H), 5.24 (d, *J* = 16.4 Hz, 1H), 5.15 (d, *J* = 16.4 Hz, 1H), 3.87 (s, 3H), 3.74 (s, 3H). **^{13}C NMR (101 MHz, Chloroform-*d*)** δ 159.0, 154.3, 145.1, 138.6, 136.0, 135.3, 130.3, 129.1, 128.5, 127.8, 126.9, 126.8, 126.3, 123.4, 118.6, 117.5, 113.0, 111.1, 56.0, 55.8, 54.2. **^{11}B NMR (128 MHz, Chloroform-*d*)** δ 38.5. **HRMS (ESI)** calcd for $C_{27}H_{24}BBrNO_2$ $[M+H]^+$: 484.1078, found: 484.1082.

1-benzyl-3-bromo-6-methoxy-2-(2-methoxynaphthalen-1-yl)-1,2-dihydrobenzo[e][1,2]azaborinine (1c)

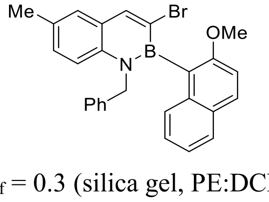

White solid, mp: 109-111ºC. Yield: 64%. R_f = 0.3 (silica gel, PE:DCM = 5:1)

^{1}H NMR (500 MHz, Chloroform-*d*) δ 8.40 (s, 1H), 7.87 (d, *J* = 9.0 Hz, 1H), 7.83 – 7.77 (m, 1H), 7.49 – 7.46 (m, 1H), 7.44 – 7.39 (m, 1H), 7.34 – 7.28 (m, 2H), 7.27 – 7.23 (m, 2H), 7.22 – 7.15 (m, 3H), 7.11 (dd, *J* = 14.5, 7.2 Hz, 3H), 5.23 (d, *J* = 16.4 Hz, 1H), 5.16 (d, *J* = 16.4 Hz, 1H), 3.72 (s, 3H), 2.42 (s, 3H). **^{13}C NMR (101 MHz, Chloroform-*d*)** δ 159.00, 145.40, 138.69, 138.61, 136.01, 131.25, 130.33, 130.03, 129.46, 129.10, 128.5, 127.1, 126.9, 126.7, 126.3, 123.4, 117.3, 113.0, 56.0, 54.0, 20.7. **^{11}B NMR (128 MHz, Chloroform-*d*)** δ 39.2. **HRMS (ESI)** calcd for $C_{27}H_{24}BBrNO$ [M+H]$^+$: 468.1129, found: 468.1130.

1-benzyl-3-bromo-7-fluoro-2-(2-methoxynaphthalen-1-yl)-1,2-dihydrobenzo[e][1,2]azaborinine (1d)

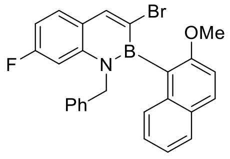

White solid, mp: 95-97ºC. Yield: 36%. R_f = 0.4 (silica gel, PE:DCM = 5:1)

^{1}H NMR (400 MHz, Chloroform-*d*) δ 8.47 (s, 1H), 7.93 (d, *J* = 8.9 Hz, 1H), 7.87 (d, *J* = 6.7 Hz, 1H), 7.69 (t, *J* = 7.4 Hz, 1H), 7.48 – 7.33 (m, 3H), 7.33 – 7.12 (m, 11H), 7.03 (t, *J* = 8.0 Hz, 2H), 5.29 (d, *J* = 16.4 Hz, 1H), 5.14 (d, *J* = 16.4 Hz, 1H), 3.80 (s, 3H). **^{13}C NMR (101 MHz, Chloroform-*d*)** δ 164.0, 161.6, 159.0, 145.1, 142.1 (d, *J* = 10.4 Hz), 137.7, 135.9, 131.3 (d, *J* = 10.1 Hz), 130.6, 129.1, 128.6, 128.6, 127.1, 126.7, 126.5, 126.3, 123.8 (d, *J* = 1.6 Hz), 123.5, 112.9, 110.5, 110.2, 104.4, 104.2, 56.0, 54.4. **^{11}B NMR (128 MHz, Chloroform-*d*)** δ 40.2. **HRMS (ESI)** calcd for $C_{26}H_{21}BBrFNO$ [M+H]$^+$: 472.0878, found: 472.0879.

4-benzyl-2-bromo-3-(2-methoxynaphthalen-1-yl)-3,4-dihydronaphtho[1,2-e][1,2]azaborinine (1e)

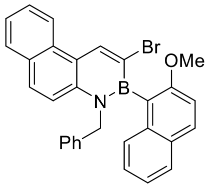

White solid, mp: 98-100ºC. Yield: 70%. R_f = 0.3 (silica gel, PE:DCM = 2:1)

^{1}H NMR (400 MHz, Chloroform-*d*) δ 9.49 (s, 1H), 8.60 (d, *J* = 8.5 Hz, 1H), 7.93 – 7.77 (m, 4), 7.71 – 7.66 (m, 1), 7.61 (d, *J* = 9.4 Hz, 1H), 7.53 (t, *J* = 7.3 Hz, 1H), 7.48 – 7.42 (m, 1H), 7.36 – 7.24 (m, 3), 7.23 – 7.06 (m, 5), 5.49 – 5.25 (m, 2H), 3.72 (s, 3H). **^{13}C NMR (101 MHz, Chloroform-*d*)** δ 159.0, 140.6, 139.5, 138.6, 136.0, 130.7, 130.4, 129.7, 129.2, 129.2, 128.6, 128.5, 128.5, 127.5, 127.0, 126.9, 126.4, 126.3, 125.2, 123.5, 122.1, 121.1, 117.8, 113.0, 56.0, 54.7. **^{11}B NMR (128 MHz, Chloroform-*d*)** δ 38.5. **HRMS (ESI)** calcd for $C_{30}H_{24}BBrNO$ [M+H]$^+$: 504.1129, found: 504.1128.

3-bromo-2-(2-methoxynaphthalen-1-yl)-1-(4-methylbenzyl)-1,2-dihydrobenzo[e][1,2]azaborinine (1f)

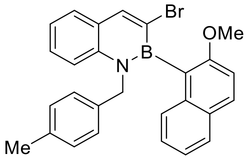

White solid, mp: 78-80 ºC. Yield: 56%. R_f = 0.4 (silica gel, PE:DCM =5:1)

^{1}H NMR (500 MHz, Chloroform-*d*) δ 8.46 (s, 1H), 7.87 (d, *J* = 9.0 Hz, 1H), 7.85 – 7.79 (m, 1H), 7.68 (dd, *J* = 7.8, 1.4 Hz, 1H), 7.44 – 7.35 (m, 3H), 7.35 – 7.29 (m, 2H), 7.28 – 7.21 (m, 2H), 7.00 (d, *J* = 2.5 Hz, 4H), 5.16 (q, 2H), 3.76 (s, 3H), 2.24 (s, 3H). **^{13}C NMR (126 MHz, Chloroform-*d*)** δ 159.0, 145.6, 140.7, 136.3, 136.0, 135.4, 130.4, 129.8, 129.2, 129.1, 128.7, 128.5, 127.2, 126.9, 126.4, 126.3, 123.4, 121.8, 117.5, 113.0, 56.1,

53.9, 21.1. **^{11}B NMR (128 MHz, Chloroform-*d*)** δ 39.8. **HRMS (ESI)** calcd for $C_{27}H_{24}BBrNO$ $[M+H]^+$: 468.1129, found: 468.1137.

3-bromo-1-(4-fluorobenzyl)-2-(2-methoxynaphthalen-1-yl)-1,2-dihydrobenzo[e][1,2]azaborinine (1g)

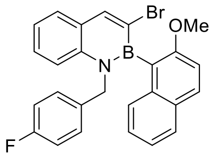

White solid, mp: 110-112°C. Yield: 51%. R_f = 0.4 (silica gel, PE:DCM = 5:1)

^{1}H NMR (400 MHz, Chloroform-*d*) δ 8.49 (s, 1H), 7.91 (d, *J* = 9.0 Hz, 1H), 7.87 – 7.81 (m, 1H), 7.72 (d, *J* = 7.7 Hz, 1H), 7.41 (d, *J* = 7.7 Hz, 2H), 7.34 (d, *J* = 6.2 Hz, 3H), 7.29 (d, *J* = 6.0 Hz, 2H), 7.17 (d, *J* = 7.7 Hz, 2H), 7.05 (d, *J* = 7.8 Hz, 2H), 5.20 (q, *J* = 16.6 Hz, 2H), 3.76 (s, 3H). **^{13}C NMR (101 MHz, Chloroform-*d*)** δ 159.0, 145.7, 140.4, 137.0, 135.9, 132.5, 130.5, 129.9, 129.1, 128.8, 128.6, 128.6, 127.8, 127.2, 126.8, 126.5, 123.6, 122.1, 117.2, 112.9, 56.0, 53.4. **^{11}B NMR (128 MHz, Chloroform-*d*)** δ 40.8. **HRMS (ESI)** calcd for $C_{26}H_{21}BBrFNO$ $[M+H]^+$: 472.0878, found: 472.0873.

3-bromo-1-(4-chlorobenzyl)-2-(2-methoxynaphthalen-1-yl)-1,2-dihydrobenzo[e][1,2]azaborinine (1h)

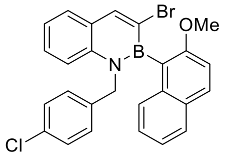

White solid, mp: 106-108 ^{o}C. Yield: 53%. R_f = 0.4 (silica gel, PE:DCM =5:1)

^{1}H NMR (400 MHz, Chloroform-*d*) δ 8.49 (s, 1H), 7.91 (d, *J* = 8.9 Hz, 1H), 7.88 – 7.83 (m, 1H), 7.72 (d, *J* = 7.7 Hz, 1H), 7.47 – 7.32 (m, 5H), 7.29 (t, *J* = 7.9 Hz, 2H), 7.14 – 7.04 (m, 2H), 6.89 (t, *J* = 8.2 Hz, 2H), 5.21 (q, *J* = 16.3 Hz, 2H), 3.77 (s, 3H). **^{13}C NMR (101 MHz, Chloroform-*d*)** δ 162.9, 160.5, 159.0, 145.7, 140.4, 135.9, 134.0 (d, *J* = 3.0 Hz), 130.5, 129.9, 129.1, 128.6 (d, *J* = 20.0 Hz), 127.9 (d, *J* = 7.9 Hz), 127.2, 126.8, 126.5, 123.5, 122.0, 117.3, 115.3 (d, *J* = 21.4 Hz), 112.9, 56.0, 53.3. **^{11}B NMR (128 MHz, Chloroform-*d*)** δ 39.9. **HRMS (ESI)** calcd for $C_{26}H_{21}BBrClNO$ $[M+H]^+$: 488.0583, found: 488.0583.

3-bromo-1-butyl-2-(2-methoxynaphthalen-1-yl)-1,2-dihydrobenzo[e][1,2]azaborinine (1i)

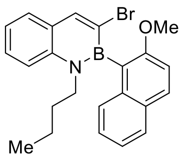

White solid, mp: 114-116 ^{o}C. Yield: 40%. R_f = 0.45 (silica gel, PE:DCM =5:1)

^{1}H NMR (500 MHz, Chloroform-*d*) δ 8.39 (s, 1H), 7.94 (d, *J* = 9.0 Hz, 1H), 7.87 – 7.83 (m, 1H), 7.69 (dd, *J* = 7.6, 1.1 Hz, 1H), 7.62 – 7.54 (m, 2H), 7.40 – 7.20 (m, 5H), 4.04 – 3.80 (m, 5H), 1.76 – 1.59 (m, 2H), 1.18 – 1.02 (m, 2H), 0.64 (t, *J* = 7.4 Hz, 3H). **^{13}C NMR (126 MHz, Chloroform-*d*)** δ 158.6, 145.1, 140.6, 136.0, 130.2, 130.1, 129.2, 128.7, 128.4, 127.1, 127.0, 126.2, 123.5, 121.6, 115.9, 113.3, 56.4, 49.8, 31.7, 20.2, 13.6. **^{11}B NMR (128 MHz, Chloroform-*d*)** δ 37.8. **HRMS (ESI)** calcd for $C_{23}H_{24}BBrNO$ $[M+H]^+$: 420.1129, found: 420.1131.

3-bromo-2-(2-methoxynaphthalen-1-yl)-1-(thiophen-2-ylmethyl)-1,2-dihydrobenzo[e][1,2]azaborinine (1j)

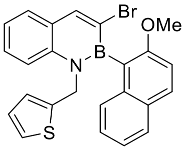

White solid, mp: 196-198 °C. Yield: 22%. R_f = 0.4 (silica gel, PE:DCM =5:1)

^{1}H NMR (500 MHz, Chloroform-*d*) δ 8.42 (s, 1H), 7.90 (d, *J* = 9.0 Hz, 1H), 7.85 – 7.79 (m, 1H), 7.68 (dd, *J* = 7.8, 1.5 Hz, 1H), 7.62 (d, *J* = 8.6 Hz, 1H), 7.50 – 7.43 (m, 1H), 7.40 – 7.35 (m, 1H), 7.33 – 7.27 (m, 3H), 7.25 (d, *J* = 7.7 Hz, 1H), 7.03 (dd, *J* = 5.1, 1.2 Hz, 1H), 6.77 (dd, *J* = 5.0, 3.5 Hz, 1H), 6.72 – 6.67 (m, 1H), 5.35 (d, *J* = 3.1 Hz, 2H), 3.78 (s, 3H). **^{13}C NMR (126 MHz, Chloroform-*d*)** δ 159.0, 145.8, 141.8, 140.5, 136.0, 130.6, 129.9, 129.1, 128.8, 128.5, 127.2, 127.0, 126.7, 126.4, 125.2, 124.2, 123.5, 122.1, 116.8, 113.0, 56.1, 49.4. **^{11}B NMR (128 MHz, Chloroform-*d*)** δ 40.0. **HRMS (ESI)** calcd for $C_{24}H_{20}BBrNOS$ $[M+H]^+$: 460.0537, found: 460.0536.

3-bromo-1-isopropyl-2-(2-methoxynaphthalen-1-yl)-1,2-dihydrobenzo[e][1,2]azaborinine (1k)

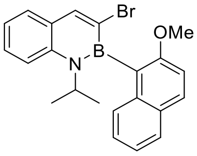

White solid, mp: 64-66 °C. Yield: 30%. R_f = 0.5 (silica gel, PE:DCM = 5:1).

^{1}H NMR (400 MHz, Chloroform-*d*) δ 8.38 (s, 1H), 8.00 – 7.83 (m, 3H), 7.71 (d, *J* = 7.8 Hz, 1H), 7.55 (t, *J* = 7.7 Hz, 1H), 7.45 – 7.22 (m, 5H), 4.95 – 4.68 (m, 1H), 3.90 (s, 3H), 1.83 – 1.35 (m, 6H). **^{13}C NMR (101 MHz, Chloroform-*d*)** δ 158.2, 145.4, 139.6, 136.0, 130.5, 129.9, 129.2, 128.4, 128.1, 127.5, 127.1, 126.2, 123.6, 121.3, 119.0, 113.3, 56.4, 54.4, 21.7, 21.6. **^{11}B NMR (128 MHz, Chloroform-*d*)** δ 37.9. **HRMS (ESI)** calcd for $C_{22}H_{22}BBrNO$ $[M+H]^+$: 406.0972, found: 406.0973.

1-benzyl-3-bromo-2-(2-methoxy-6-methylnaphthalen-1-yl)-1,2-dihydrobenzo[e][1,2]azaborinine (1l)

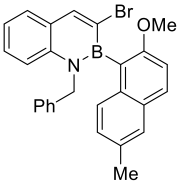

White solid, mp: 94-96 °C. Yield: 61%. R_f = 0.4 (silica gel, PE:DCM =5:1)

^{1}H NMR (500 MHz, Chloroform-*d*) δ 8.48 (s, 1H), 7.80 (d, *J* = 9.0 Hz, 1H), 7.70 (d, *J* = 8.6 Hz, 1H), 7.61 (s, 1H), 7.45 – 7.37 (m, 2H), 7.34 (d, *J* = 8.5 Hz, 1H), 7.28 – 7.16 (m, 5H), 7.16 – 7.09 (m, 3H), 5.28 (d, *J* = 16.4 Hz, 1H), 5.20 (d, *J* = 16.4 Hz, 1H), 3.72 (s, 3H), 2.48 (s, 3H). **^{13}C NMR (126 MHz, Chloroform-*d*)** δ 158.5, 145.6, 140.6, 138.5, 134.1, 132.8, 129.8, 129.6, 129.3, 128.7, 128.7, 128.5, 127.5 127.2, 126.8, 126.7, 126.3, 121.9, 117.5, 113.0, 56.0, 54.1, 21.6. **^{11}B NMR (128 MHz, Chloroform-*d*)** δ 37.4. **HRMS (ESI)** calcd for $C_{27}H_{24}BBrNO$ $[M+H]^+$: 468.1129, found: 468.1128.

1-benzyl-3-bromo-2-(2-ethoxynaphthalen-1-yl)-1,2-dihydrobenzo[e][1,2]azaborinine (1m)

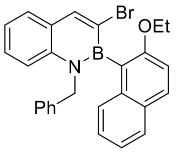

White solid, mp: 102-104°C. Yield: 60%. R_f = 0.4 (silica gel, PE:DCM = 5:1)

^{1}H NMR (500 MHz, Chloroform-*d*) δ 8.53 (s, 1H), 7.97 – 7.80 (m, 2H), 7.73 (d, *J* = 7.3 Hz, 1H), 7.55 – 7.45 (m, 2H), 7.45 – 7.33 (m, 3H), 7.32 – 7.12 (m, 7H), 5.39 (d, *J* = 16.3 Hz, 1H), 5.26 (d, *J* = 16.3 Hz, 1H), 4.25 – 4.07 (m, 2H), 1.36 (t, *J* = 7.0 Hz, 3H). **^{13}C NMR (126 MHz, Chloroform-*d*)** δ 158.47, 145.42, 140.50, 138.48, 135.96, 130.27, 129.71, 129.03, 128.57, 128.50, 128.43, 127.15, 126.81, 126.73, 126.41, 126.32, 123.34, 121.81, 117.45, 113.89, 64.19, 54.14, 15.24. **^{11}B NMR (160 MHz, Chloroform-*d*)** δ 38.22. **HRMS (ESI)** calcd for $C_{27}H_{24}BBrNO$ $[M+H]^+$: 468.1129, found: 468.1130.

1-benzyl-3-bromo-2-(2-(ethylthio)naphthalen-1-yl)-1,2-dihydrobenzo[e][1,2]azaborinine (1n)

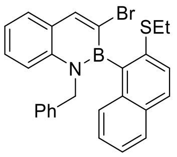

White solid, mp: 94-96°C. Yield: 80%. R_f = 0.4 (silica gel, PE:DCM = 5:1)

^{1}H NMR (500 MHz, Chloroform-*d*) δ 8.53 (s, 1H), 7.84 (d, *J* = 8.5 Hz, 2H), 7.73 (d, *J* = 7.6 Hz, 1H), 7.58 (d, *J* = 8.6 Hz, 1H), 7.48 (d, *J* = 8.1 Hz, 1H), 7.45 – 7.38 (m, 3H), 7.38 – 7.32 (m, 1H), 7.31 – 7.23 (m, 1H), 7.22 – 7.16 (m, 2H), 7.16 – 7.06 (m, 3H), 5.25 (dd, *J* = 4.1 Hz, 2H), 3.05 (dq, *J* = 12.5, 7.4 Hz, 1H), 2.89 (dq, *J* = 12.1, 7.4 Hz, 1H), 1.29 (t, *J* = 7.4 Hz, 3H). **^{13}C NMR (126 MHz, Chloroform-*d*)** δ 145.82, 140.53, 137.89, 136.58, 135.25, 131.70, 129.82, 128.95, 128.78, 128.48, 128.46, 127.54, 127.51, 127.08, 126.81, 126.35, 126.26, 125.63, 122.00, 117.67, 53.93, 29.30, 14.82. **^{11}B NMR (160 MHz, Chloroform-*d*)** δ 36.9. **HRMS (ESI)** calcd for $C_{27}H_{24}BBrNS$ $[M+H]^+$: 484.0900, found: 484.0903.

2.4 General procedure for the synthesis of atropisomers with a single C-B stereogenic axis

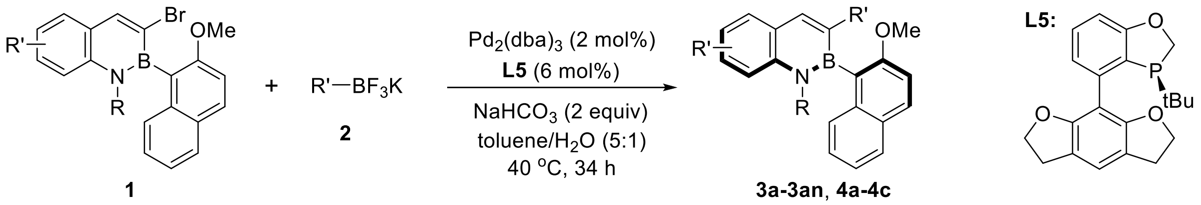

In air, a 25 mL schlenk tube was charged with **1** (0.1 mmol, 1 equiv), **2** (0.13 mmol, 1.3 equiv), $Pd_2(dba)_3$ (2 mol%), **L5** (6 mol%) and $NaHCO_3$ (0.2 mmol, 2.0 equiv). The tube was evacuated and filled with argon for three cycles. Then, 1.5 mL of toluene and 0.3 ml water was added under argon. The reaction was allowed to stir at 40 °C for 34 hours. Upon completion, proper amount of silica gel was added to the reaction mixture. After removal of the solvent, the crude reaction mixture was purified on silica gel (petroleum ether and ethyl acetate) to afford the desired products.

(R_a)-1-benzyl-2-(2-methoxynaphthalen-1-yl)-3-(3-methoxyphenyl)-1,2-dihydrobenzo[e][1,2]azaborinine (3a)

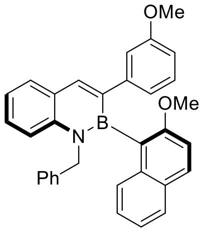

White solid, mp: 85-87 °C. Yield: 87%. R_f = 0.25 (silica gel, PE:DCM = 5:1).

^{1}H NMR (500 MHz, Chloroform-*d*) δ 8.18 (s, 1H), 7.84 (dd, *J* = 7.8, 1.5 Hz, 1H), 7.78 (dd, *J* = 9.4, 2.2 Hz, 2H), 7.59 – 7.52 (m, 1H), 7.47 (d, *J* = 8.5 Hz, 1H), 7.43 – 7.37 (m, 1H), 7.35 – 7.25 (m, 3H), 7.24 – 7.12 (m, 5H), 7.11 – 7.03 (m, 2H), 6.91 (dt, *J* = 7.6, 1.2 Hz, 1H), 6.66 – 6.56 (m, 2H), 5.37 (d, *J* = 16.4 Hz, 1H), 5.22 (d, *J* = 16.4 Hz, 1H), 3.48 (s, 3H), 3.21 (s, 3H). **^{13}C NMR (126 MHz, Chloroform-*d*)** δ 158.9, 158.8, 146.2, 142.4, 141.0, 139.0, 136.4, 130.7, 129.8, 129.1, 128.6, 128.4, 128.3, 127.3, 127.3, 126.6, 126.5, 126.3, 123.3, 121.4, 120.2, 117.1, 113.0, 112.9, 112.5, 55.8, 54.6, 53.5. **^{11}B NMR (128 MHz, Chloroform-*d*)** δ 39.8. **HRMS (ESI)** calcd for $C_{33}H_{29}BNO_2$ $[M+H]^+$: 482.2286, found: 482.2293. **HPLC analysis**: DAICEL CHIRALCEL IA-3, hexane/isopropanol = 90/10, 1.0 mL/min, λ = 254 nm, t_R (major) = 4.87 min, t_R (minor) = 5.91 min, 96% ee. **$[\alpha]^{25}_D$**: +294.5 (*c* 0.5, $CHCl_3$).

(R_a)-1-benzyl-2-(2-methoxynaphthalen-1-yl)-3-(4-methoxyphenyl)-1,2-dihydrobenzo[e][1,2]azaborinine (3b)

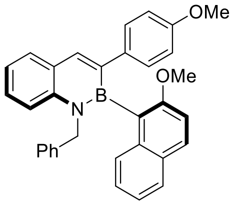

White solid, mp: 82-84°C. Yield: 80%.R_f = 0.20 (silica gel, PE:DCM = 5:1).

^{1}H NMR (500 MHz, Chloroform-*d*) δ 8.29 (s, 1H), 8.07 – 7.91 (m, 3H), 7.73 – 7.61 (m, 2H), 7.59 – 7.52 (m, 1H), 7.51 – 7.42 (m, 3H), 7.41 – 7.23 (m, 8H), 6.85 – 6.71 (m, 2H), 5.48 (d, 16.4 Hz, 1H), 5.37 (d, *J* = 16.4 Hz, 1H), 3.87 (s, 3H), 3.67 (s, 3H). **^{13}C NMR (126 MHz, Chloroform-*d*)** δ 158.9, 157.9, 141.7, 140.8, 139.1, 137.4, 136.3, 130.5, 129.8, 129.1, 129.0, 128.4, 128.0, 127.4, 127.2, 126.5, 126.5, 126.2, 123.2, 121.4, 117.1, 113.1, 112.9, 55.7, 55.2, 53.4. **^{11}B NMR (128 MHz, Chloroform-*d*)** δ 39.8. **HRMS (ESI)** calcd for $C_{33}H_{29}BNO_2$ $[M+H]^+$: 482.2286, found: 482.2288. **HPLC analysis**: DAICEL CHIRALCEL IA-3, hexane/isopropanol = 90/10, 1.0 mL/min, λ = 254 nm, t_R (major) = 4.97 min, t_R (minor) = 6.87 min, 94% ee. **$[\alpha]^{25}_D$**: +265.7 (*c* 0.5, $CHCl_3$).

(R_a)-1-benzyl-2-(2-methoxynaphthalen-1-yl)-3-(4-(methylthio)phenyl)-1,2-dihydrobenzo[e][1,2]azaborinine (3c)

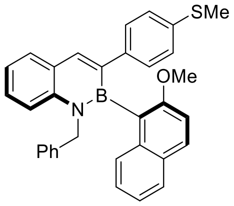

White solid, mp: 78-80°C. Yield: 90%. R_f = 0.4 (silica gel, PE:DCM = 5:1)

^{1}H NMR (500 MHz, Chloroform-*d*) δ 8.09 (s, 1H), 7.84 – 7.73 (m, 3H), 7.49 – 7.41 (m, 2H), 7.39 – 7.34 (m, 1H), 7.29 – 7.22 (m, 3H), 7.19 – 7.16 (m, 2H), 7.14 – 7.03 (m, 6H), 6.94 (d, *J* = 8.4 Hz, 2H), 5.29 (d, *J* = 16.4 Hz, 1H), 5.16 (d, *J* = 16.4 Hz, 1H), 3.46 (s, 3H), 2.36 (s, 3H). **^{3}C NMR (126 MHz, Chloroform-*d*)** δ 159.0, 142.2, 142.0, 141.0, 139.1, 136.3, 135.2, 130.6, 129.9, 129.1, 128.4, 128.4, 128.4, 128.3, 127.3, 127.2, 126.6, 126.5, 126.3, 126.1, 123.2, 121.4, 117.1, 112.9, 55.7, 53.4, 16.1. **^{11}B NMR (128 MHz, Chloroform-*d*)** δ 40.9 **HRMS (ESI)** calcd for $C_{33}H_{29}BNOS$ $[M+H]^+$: 498.2057, found: 498.2061. **HPLC analysis**: DAICEL CHIRALCEL IA-3, hexane/isopropanol = 90/10, 1.0 mL/min, λ = 254 nm, t_R (major) = 4.85 min, t_R (minor) = 6.17 min, 80% ee. **[α]^{25}$_D$**: +132.0 (*c* 0.1, $CHCl_3$).

(*R$_a$*)-1-benzyl-2-(2-methoxynaphthalen-1-yl)-3-(4-(methylthio)phenyl)-1,2-dihydrobenzo[e][1,2]azaborinine (3d)

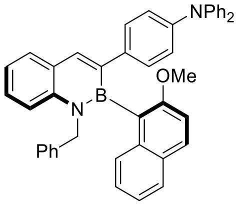

White solid, mp: 72-74°C. Yield: 99%. R_f = 0.4 (silica gel, PE:DCM = 5:1).

^{1}H NMR (500 MHz, Chloroform-*d*) δ 8.16 (s, 1H), 7.86 – 7.75 (m, 3H), 7.47 (t, *J* = 7.8 Hz, 2H), 7.40 – 7.35 (m, 1H), 7.31 – 7.24 (m, 3H), 7.21 – 7.12 (m, 10H), 7.02 – 6.92 (m, 8H), 6.79 – 6.72 (m, 2H), 5.37 (d, *J* = 16.4 Hz, 1H), 5.26 (d, *J* = 16.4 Hz, 1H), 3.55(s, 3H). **^{13}C NMR (101 MHz, Chloroform-*d*)** δ 159.0, 148.0, 145.5, 141.5, 140.9, 139.7, 139.1, 136.3, 130.5, 130.0, 129.1, 129.1, 128.9, 128.4, 128.3, 128.1, 127.4, 127.3, 126.6, 126.5, 126.1, 123.8, 123.7, 123.2, 122.3, 121.4, 117.1, 112.7, 55.7, 53.5. **^{11}B NMR (128 MHz, Chloroform-*d*) δ** 41.4. **HRMS (ESI)** calcd for $C_{44}H_{36}BN_2O$ $[M+H]^+$: 619.2915, found: 619.2921. **HPLC analysis**: DAICEL CHIRALCEL IA-3, hexane/isopropanol = 98/2, 1.0 mL/min, λ = 254 nm, t_R (major) = 6.52min, t_R (minor) = 7.39 min, 83% ee. **[α]^{25}$_D$**: +132.4 (*c* 0.5, $CHCl_3$).

(*R$_a$*)-1-benzyl-3-(3-chloro-4-methoxyphenyl)-2-(2-methoxynaphthalen-1-yl)-1,2-dihydrobenzo[e][1,2]azaborinine (3e)

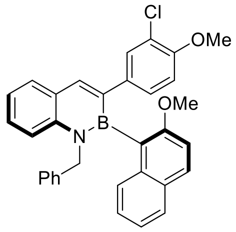

White solid, mp: 79-81 °C. Yield: 67%. R_f = 0.2 (silica gel, PE:DCM = 5:1).

^{1}H NMR (400 MHz, Chloroform-*d*) δ 8.10 (s, 1H), 7.81 (q, *J* = 8.6 Hz, 3H), 7.52 – 7.35 (m, 3H), 7.34 – 7.25 (m, 4H), 7.24 – 7.07 (m, 6H), 6.93 (d, *J* = 8.5 Hz, 1H), 6.56 (d, *J* = 8.5 Hz, 1H), 5.31 (d, *J* = 16.4 Hz, 1H), 5.19 (d, *J* = 16.4 Hz, 1H), 3.77 (s, 3H), 3.56 (s, 3H). **^{13}C NMR (126 MHz, Chloroform-*d*)** δ 158.9, 153.1, 142.1, 141.0, 139.0, 138.3, 136.2, 130.6, 130.0, 129.7, 129.1, 128.4, 128.4, 127.2, 127.1, 127.1, 126.6, 126.5, 126.3, 123.3, 121.5, 121.4, 117.1, 112.7, 111.3, 56.1, 55.6, 53.4. **^{11}B NMR (128 MHz, Chloroform-*d*)** δ 40.8

HRMS (ESI) calcd for $C_{33}H_{29}BClNO_2$ $[M+H]^+$: 516.1896, found: 546.1896. **HPLC analysis**: DAICEL CHIRALCEL IA-3, hexane/isopropanol = 90/10, 1.0 mL/min, λ = 254 nm, t_R (major) = 5.76 min, t_R (minor) = 7.08 min, 91% ee. **[α]^{25}$_D$**: +216.0 (*c* 0.5, $CHCl_3$).

(R_a)-1-benzyl-3-(3-fluoro-4-methoxyphenyl)-2-(2-methoxynaphthalen-1-yl)-1,2-dihydrobenzo[e][1,2]azaborinine (3f)

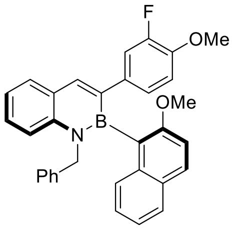

White solid, mp: 74-76 °C. Yield: 99%. R_f = 0.3 (silica gel, PE:DCM = 5:1).

^{1}H NMR (500 MHz, Chloroform-*d*) δ 8.10 (d, *J* = 2.4 Hz, 1H), 7.88 – 7.72 (m, 3H), 7.50 – 7.43 (m, 2H), 7.41 – 7.34 (m, 1H), 7.30 – 7.24 (m, 3H), 7.23 – 7.18 (m, 2H), 7.16 – 7.06 (m, 4H), 7.00 – 6.92 (m, 1H), 6.84 (d, *J* = 8.5 Hz, 1H), 6.61 (t, *J* = 8.7 Hz, 1H), 5.31 (d, *J* = 16.5 Hz, 1H), 5.19 (d, *J* = 16.4 Hz, 1H), 3.74 (s, 3H), 3.54 (s, 3H). **^{13}C NMR (101 MHz, Chloroform-*d*)** δ 158.9, 153.0, 150.6, 145.7 (d, *J* = 10.9 Hz), 142.2, 140.9, 139.0, 138.2, 138.2, 136.2, 130.6, 130.0, 129.1, 128.4, 128.4, 127.2, 127.0, 126.6, 126.4, 126.3, 123.5 (d, *J* = 3.3 Hz), 123.3, 121.5, 117.1, 115.6 (d, *J* = 18.3 Hz), 112.7, 112.6 (d, *J* = 2.2 Hz), 56.1, 56.0, 53.4. **^{19}F NMR (471 MHz, Chloroform-*d*)** δ -137.0. **^{11}B NMR (128 MHz, Chloroform-*d*)** δ 40.0. **HRMS (ESI)** calcd for $C_{33}H_{28}BFNO_2$ $[M+H]^+$: 500.2192, found: 500.2196. **HPLC analysis**: DAICEL CHIRALCEL IA-3, hexane/isopropanol = 90/10, 1.0 mL/min, λ = 254 nm, t_R (major) = 5.87 min, t_R (minor) = 7.54 min, 93% ee. **$[\alpha]^{25}_{D}$**: +248.0 (*c* 0.3, $CHCl_3$).

(R_a)-1-benzyl-3-(4-methoxy-3-methylphenyl)-2-(2-methoxynaphthalen-1-yl)-1,2-dihydrobenzo[e][1,2]azaborinine (3g)

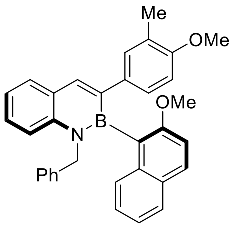

White solid, mp: 81-83 °C. Yield: 99%. R_f = 0.25 (silica gel, PE:DCM = 3:1).

^{1}H NMR (400 MHz, Chloroform-*d*) δ 8.11 (s, 1H), 7.81 (dd, *J* = 14.5, 8.1 Hz, 3H), 7.53 – 7.41 (m, 2H), 7.37 (t, *J* = 7.7 Hz, 1H), 7.28 (t, *J* = 7.4 Hz, 3H), 7.25 – 7.07 (m, 6H), 6.98 (s, 1H), 6.92 (d, *J* = 8.4 Hz, 1H), 6.50 (d, *J* = 8.4 Hz, 1H), 5.32 (d, *J* = 16.4 Hz, 1H), 5.19 (d, *J* = 16.4 Hz, 1H), 3.71 (s, 3H), 3.51 (s, 3H), 2.00 (s, 3H). **^{13}C NMR (101 MHz, Chloroform-*d*)** δ 158.9, 156.1, 141.5, 140.7, 139.2, 136.9, 136.3, 130.5, 130.4, 129.7, 129.1, 128.3, 128.3, 127.9, 127.5, 127.3, 126.5, 126.5, 126.1, 126.1, 125.3, 123.2, 121.3, 117.0, 112.9, 109.3, 55.7, 55.2, 53.4, 16.2. **^{11}B NMR (128 MHz, Chloroform-*d*)** δ 41.0. **HRMS (ESI)** calcd for $C_{34}H_{31}BNO_2$ $[M+H]^+$: 496.2442, found: 496.2449. **HPLC analysis**: DAICEL CHIRALCEL IA-3, hexane/isopropanol = 90/10, 1.0 mL/min, λ = 254 nm, t_R (major) = 4.80 min, t_R (minor) = 6.31 min, 92% ee. **$[\alpha]^{25}_{D}$**: +244.2 (*c* 0.5, $CHCl_3$).

(R_a)-1-benzyl-3-(2,3-dihydrobenzo[b][1,4]dioxin-6-yl)-2-(2-methoxynaphthalen-1-yl)-1,2-dihydrobenzo[e][1,2]azaborinine (3h)

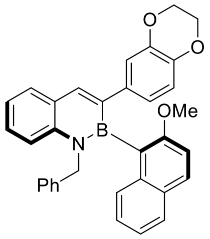

White solid, mp: 84-86 ^{o}C. Yield: 99%. R_f = 0.3 (silica gel, PE:DCM = 2:1).

^{1}H R (500 MHz, Chloroform-*d*) δ 8.06 s, 1H), 7.85 – 7.65 (m, 3H), 7.46 – 7.37 (m, 2H), 7.37 – 7.29 (m, 1H), 7.29 – 7.21 (m, 3H), 7.20 – 7.14 (m, 2H), 7.11 (d, J = 8.7 Hz, 4H), 6.77 (d, J = 2.1 Hz, 1H), 6.56 (dd, J = 8.4, 2.1 Hz, 1H), 6.48 (d, J = 8.4 Hz, 1H), 5.25 (d, J = 16.5 Hz, 1H), 5.14 (d, J = 16.5 Hz, 1H), 4.12(s, 3H), 3.52(s, 3H). **^{13}C NMR (101 MHz, Chloroform-*d*)** δ 158.9, 142.8, 142.0, 141.9, 140.9, 139.1, 138.6, 136.3, 130.5, 129.8, 129.1, 128.4, 128.3, 128.1, 127.3, 127.3, 126.5, 126.5, 126.1, 123.2, 121.4, 117.1, 116.6, 116.3, 112.8, 64.4, 64.4, 55.7, 53.4. **^{11}B NMR (128 MHz, Chloroform-*d*)** δ 41.2. **HRMS (ESI)** calcd for $C_{34}H_{29}BNO_3$ $[M+H]^+$: 510.2235, found:510.2242. **HPLC analysis**: DAICEL CHIRALCEL IA-3, hexane/isopropanol = 98/2, 1.0 mL/min, λ = 254 nm, t_R (major) = 14.26 min, t_R (minor) = 23.71 min, 90% ee. **$[\alpha]^{25}_{D}$**: +185.4 (*c* 0.5, $CHCl_3$).

(R_a)-3-(benzo[d][1,3]dioxol-5-yl)-1-benzyl-2-(2-methoxynaphthalen-1-yl)-1,2-dihydrobenzo[e][1,2]azaborinine (3i)

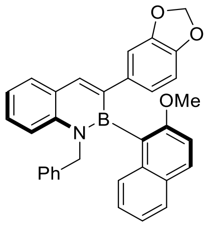

White solid, mp: 82-84 ^{o}C. Yield: 79%. R_f = 0.4 (silica gel, PE:DCM = 2:1).

^{1}H NMR (400 MHz, Chloroform-*d*) δ 8.10 (s, 1H), 7.81 (q, J = 8.9 Hz, 3H), 7.46 (d, J = 7.8 Hz, 2H), 7.39 (t, J = 7.6 Hz, 1H), 7.32 – 7.26 (m, 3H), 7.24 – 7.07 (m, 6H), 6.72 (s, 1H), 6.66 (d, J = 8.0 Hz, 1H), 6.53 (d, J = 8.0 Hz, 1H), 5.81 (s, 2H), 5.31 (d, J = 16.4 Hz, 1H), 5.20 (d, J = 16.4 Hz, 1H), 3.56 (s, 3H). **^{13}C NMR (101 MHz, Chloroform-*d*)** δ 158.9, 147.0, 145.7, 142.0, 140.9, 139.2, 139.1, 136.2, 130.5, 129.9, 129.1, 128.4, 128.4, 128.2, 127.3, 127.2, 126.5, 126.5, 126.2, 123.2, 121.4, 121.3, 117.1, 112.7, 108.6, 107.7, 100.6, 55.6, 53.4. **^{11}B NMR (128 MHz, Chloroform-*d*)** 40.53. **HRMS (ESI)** calcd for $C_{33}H_{28}BNO_3$ $[M+H]^+$: 496.2079, found: 492.2084. **HPLC analysis**: DAICEL CHIRALCEL IA-3, hexane/isopropanol = 98/2, 1.0 mL/min, λ = 254 nm, t_R (major) = 9.63 min, t_R (minor) = 15.51 min, 96% ee. **$[\alpha]^{25}_{D}$**: +260.7 (*c* 0.5, $CHCl_3$).

(R_a)-1-ethyl-2-(2-methoxy-6-methylnaphthalen-1-yl)-2,3-dihydro-1H-naphtho[1,8-de][1,3,2]diazaborinine (3j)

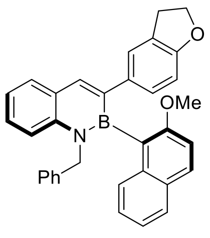

White solid, mp: 82-84 °C. Yield: 99%. R_f = 0.35 (silica gel, PE:DCM = 2:1).

^{1}H NMR (500 MHz, Chloroform-*d*) δ 8.06 (s, 1H), 7.77 (dd, *J* = 16.1, 7.8 Hz, 3H), 7.50 – 7.39 (m, 2H), 7.34 (t, *J* = 7.7 Hz, 1H), 7.28 – 7.21 (m, 3H), 7.20 – 7.05 (m, 6H), 6.96 (s, 1H), 6.87 (d, *J* = 8.2 Hz, 1H), 6.50 – 6.40 (m, 1H), 5.28 (d, *J* = 16.4 Hz, 1H), 5.16 (d, *J* = 16.4 Hz, 1H), 4.51 – 4.35 (m, 2H), 3.49 (s, 3H), 2.95 (t, *J* = 8.5 Hz, 2H). **^{13}C NMR (101 MHz, Chloroform-*d*)** δ 158.9, 158.4, 141.5, 140.7, 139.1, 137.5, 136.3, 130.4, 129.8, 129.1, 128.4, 127.9, 127.7, 127.5, 127.3, 126.5, 126.5, 126.1, 126.1, 124.5, 123.2, 121.3, 117.1, 112.8, 108.3, 71.1, 55.7, 53.4, 29.8. **^{11}B NMR (128 MHz, Chloroform-*d*)** δ 40.8. **HRMS (ESI)** calcd for $C_{34}H_{29}BNO_2$ $[M+H]^+$: 494.2286, found: 494.2285. **HPLC analysis**: DAICEL CHIRALCEL IA-3, hexane/isopropanol = 98/2, 1.0 mL/min, λ = 254 nm, t_R (major) = 9.84 min, t_R (minor) = 16.24 min, 91% ee. **$[\alpha]^{25}_D$**: +247.0 (*c* 0.5, $CHCl_3$).

(*R_a*)-methyl3-(1-benzyl-2-(2-methoxynaphthalen-1-yl)-1,2-dihydrobenzo[e][1,2]azaborinin-3-yl)benzoate (3k)

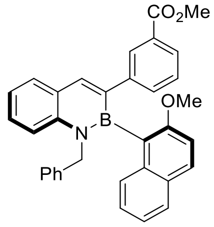

White solid, mp: 170-172 °C. Yield: 70%. R_f = 0.5 (silica gel, PE:EtOAc = 5:1).

^{1}H NMR (500 MHz, Chloroform-*d*) δ 8.14 (s, 1H), 7.91 (s, 1H), 7.80 (d, *J* = 7.5 Hz, 1H), 7.72 (dd, *J* = 9.0, 4.0 Hz, 2H), 7.67 (d, *J* = 7.7 Hz, 1H), 7.44 (d, *J* = 8.2 Hz, 2H), 7.39 – 7.32 (m, 1H), 7.27 – 7.20 (m, 4H), 7.19 – 7.07 (m, 5H), 7.07 – 6.99 (m, 2H), 5.32 (d, *J* = 16.4 Hz, 1H), 5.18 (d, *J* = 16.4 Hz, 1H), 3.74 (s, 3H), 3.48 (s, 3H). **^{13}C NMR (101 MHz, Chloroform-*d*)** δ 167.4, 158.9, 144.9, 143.4, 142.7, 141.1, 138.9, 136.2, 132.4, 130.7, 130.6, 130.0, 129.4, 129.4, 129.1, 129.0, 128.6, 128.5, 128.4, 127.6, 127.2, 127.0, 126.9, 126.6, 126.4, 126.3, 125.5, 123.2, 121.5, 117.1, 112.6, 55.5, 53.5, 51.7. **^{11}B NMR (128 MHz, Chloroform-*d*)** δ 40.5. **HRMS (ESI)** calcd for $C_{34}H_{29}BNO_3$ $[M+H]^+$: 510.2235, found: 510.2245. **HPLC analysis**: DAICEL CHIRALCEL IA-3, hexane/isopropanol = 98/2, 1.0mL/min, λ = 254 nm, t_R (major) = 10.49 min, t_R (minor) = 13.11 min, 93% ee. **$[\alpha]^{25}_D$**: +202.0 (*c* 0.5, $CHCl_3$).

(*R_a*)-1-benzyl-2-(2-methoxynaphthalen-1-yl)-3-(4-(1,2,2-triphenylvinyl)phenyl)-1,2-dihydrobenzo[e][1,2]azaborinine (3l)

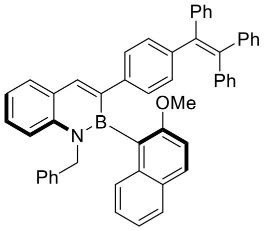

White solid, mp: 60-62 °C. Yield: 54%. R_f = 0.15 (silica gel, PE: DCM = 3:1).

^{1}H NMR (500 MHz, Chloroform-*d*) δ 8.08 (s, 1H), 7.78 (dd, *J* = 8.4, 4.1 Hz, 3H), 7.43 (d, *J* = 8.4 Hz, 2H), 7.35 (t, *J* = 7.4 Hz, 1H), 7.32 – 7.27 (m, 1H), 7.25 – 7.21 (m, 2H), 7.20 – 7.15 (m,2H), 7.11 (dd, *J* = 14.1, 7.0 Hz, 4H), 7.08 – 7.03 (m. 7H), 6.96 (t, *J* = 7.6 Hz, 4H), 6.93 – 6.86 (m, 4H), 6.83 (d, *J* = 7.8 Hz, 2H), 6.68 (d, *J* = 8.1 Hz, 2H), 5.32 (d, *J* = 16.3 Hz, 1H), 5.18 (d, *J* = 16.4 Hz, 1H), 3.43 (s, 3H). **^{13}C NMR (101 MHz, Chloroform-*d*)** δ 158.1, 145.4, 141.3, 140.7, 136.1, 135.1, 129.8, 129.0, 128.8, 128.3, 128.2, 127.7, 127.4, 126.7, 126.5, 126.1, 126.0, 125.9, 125.5, 125.1, 124.2, 123.0, 122.6, 112.6, 57.6, 55.9. **^{11}B NMR (128 MHz,**

Chloroform-*d*) δ 42.5. **HRMS (ESI)** calcd for $C_{52}H_{41}BNO$ $[M+H]^+$: 706.3276, found: 706.3285. **HPLC analysis**: DAICEL CHIRALCEL IA-3, hexane/isopropanol = 98/2, 1.0 mL/min, λ = 254 nm, t_R (major) = 6.57 min, t_R (minor) = 7.41 min, 79% ee. **$[\alpha]^{25}_D$**: +364.0 (*c* 0.2, $CHCl_3$).

(*R*a)-1-benzyl-2-(2-methoxynaphthalen-1-yl)-3-(naphthalen-2-yl)-1,2-dihydrobenzo[e][1,2]azaborinine (3m)

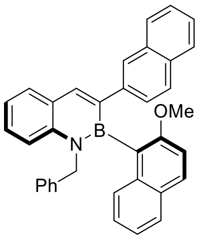

White solid, mp: 69-71 °C. Yield: 99%. R_f = 0.45 (silica gel, PE:DCM = 5:1).

^{1}H NMR (400 MHz, Chloroform-*d*) δ 8.22 (s, 1H), 7.89 – 7.80 (m, 1H), 7.77 – 7.70 (m, 2H), 7.68 – 7.62 (m, 1H), 7.61 (s, 1H), 7.55 – 7.48 (m, 2H), 7.46 (d, *J* = 8.6 Hz, 2H), 7.41 – 7.34 (m, 1H), 7.32 – 7.23 (m, 6H), 7.22 – 7.09 (m, 5H), 7.03 (d, *J* = 9.0 Hz, 1H), 5.34 (d, *J* = 16.4 Hz, 1H), 5.19 (d, *J* = 16.4 Hz, 1H), 3.41 (s, 3H). **^{13}C NMR (101 MHz, Chloroform-*d*)** δ 159.0, 142.8, 142.5, 141.0, 139.1, 136.3, 133.5, 131.9, 130.7, 129.9, 129.1, 128.4, 128.4, 128.4, 128.0, 127.4, 127.4, 127.2, 127.1, 126.8, 126.6, 126.5, 126.3, 125.5, 125.0, 123.2, 121.5, 117.1, 112.8, 55.6, 53.5. **^{11}B NMR (128 MHz, Chloroform-*d*)** δ 40.7. **HRMS (ESI)** calcd for $C_{36}H_{29}BNO$ $[M+H]^+$: 502.2337, found: 502.2340. **HPLC analysis**: DAICEL CHIRALCEL IA-3, hexane/isopropanol = 90/10, 1.0 mL/min, λ = 254 nm, t_R (major) = 5.17 min, t_R (minor) = 7.20 min, 94% ee. **$[\alpha]^{25}_D$**: +208.5 (*c* 0.5, $CHCl_3$).

(*R*a)- 1-benzyl-2-(2-methoxynaphthalen-1-yl)-3-(pyren-2-yl)-1,2-dihydrobenzo[e][1,2]azaborinine (3n)

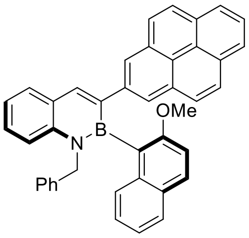

White solid, mp: 54-56 °C. Yield: 25%. R_f = 0.5 (silica gel, PE:DCM = 3:1).

^{1}H NMR (400 MHz, Chloroform-*d*) δ 8.46 (s, 1H), 8.12 (d, *J* = 7.6 Hz, 2H), 8.03 (s, 2H), 7.96 (q, *J* = 6.3, 5.8 Hz, 4H), 7.86 – 7.68 (m, 5H), 7.59 (d, *J* = 8.5 Hz, 1H), 7.49 (t, *J* = 7.6 Hz, 1H), 7.42 – 7.17 (m, 8H), 7.03 (d, *J* = 9.0 Hz, 1H), 5.52 (d, *J* = 16.3 Hz, 1H), 5.35 (d, *J* = 16.4 Hz, 1H), 3.42 (s, 3H). **^{13}C NMR (101 MHz, Chloroform-*d*)** δ 159.1, 143.6, 142.6, 141.1, 139.1, 136.4, 131.0, 130.8, 130.6, 130.0, 129.1, 128.5, 128.4, 127.7, 127.5, 127.2, 126.9, 126.6, 126.6, 126.4, 125.5, 125.1, 124.7, 124.6, 123.3, 123.1, 121.5, 117.2, 112.7, 55.6, 53.6. **^{11}B NMR (128 MHz, Chloroform-*d*)** δ 41.5. **HRMS (ESI)** calcd for $C_{42}H_{31}BNO$ $[M+H]^+$: 576.2493, found: 576.2502. **HPLC analysis**: DAICEL CHIRALCEL IA-3, hexane/isopropanol = 98/2, 1.0 mL/min, λ = 254 nm, t_R (major) = 11.42 min, t_R (minor) = 18.92 min, 96% ee. **$[\alpha]^{25}_D$**: +364.0 (*c* 0.2, $CHCl_3$).

(*R*$_a$)-2-(1-benzyl-2-(2-methoxynaphthalen-1-yl)-1,2-dihydrobenzo[e][1,2]azaborinin-3-yl)-9-phenyl-9H-carbazole (3o)

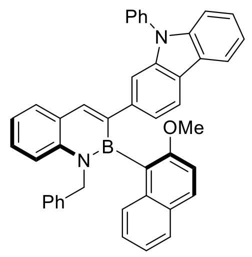

White solid, mp: 130-132 ºC. Yield: 99%. R_f = 0.3 (silica gel, PE:DCM = 3:1).
^{1}H NMR (500 MHz, Chloroform-*d*) δ 8.22 (s, 1H), 7.95 – 7.89 (m, 1H), 7.82 (d, *J* = 6.9 Hz, 1H), 7.74 (d, *J* = 7.7 Hz, 1H), 7.69 (dd, *J* = 14.2, 8.4 Hz, 2H), 7.54 (d, *J* = 8.1 Hz,1H), 7.47 (t, *J* = 7.7 Hz, 2H), 7.44 – 7.39 (m, 3H), 7.36-7.27 (m, 4H), 7.25 – 7.18 (m, 4H), 7.18 – 7.13 (m, 5H), 7.11-7.06 (m, 1H), 7.03 (dd, *J* = 17.1, 8.8 Hz, 2H), 5.33 (d, *J* = 16.5 Hz, 1H), 5.18 (d, *J* = 16.4 Hz, 1H), 3.42 (s, 3H).**^{13}C NMRa (126 MHz, Chloroform-*d*)** δ 159.0, 148.0, 145.5, 141.5, 140.9, 139.7, 139.1, 136.3, 130.5, 130.0, 129.1, 129.1, 128.9, 128.4, 128.3, 128.1, 127.4, 127.3, 126.6, 126.5, 126.1, 123.9, 123.8, 123.2, 122.3, 121.4, 117.1, 112.7, 55.7, 53.5. **^{11}B NMR (128 MHz, Chloroform-*d*)** δ 42.9. **HRMS (ESI)** calcd for $C_{44}H_{35}BN_2O$ $[M+H]^+$: 617.2759, found: 617.2765. **HPLC analysis**: DAICEL CHIRALCEL IA-3, hexane/isopropanol = 90/10, 1.0 mL/min, λ = 254 nm, t_R (major) = 10.35 min, t_R (minor) = 12.10 min, 87% ee. **$[\alpha]^{25}_D$**: +132.2 (*c* 0.5, $CHCl_3$).

(*R*$_a$)-9-(4-(1-benzyl-2-(2-methoxynaphthalen-1-yl)-1,2-dihydrobenzo[e][1,2]azaborinin-3-yl)phenyl)-9H-carbazole (3p)

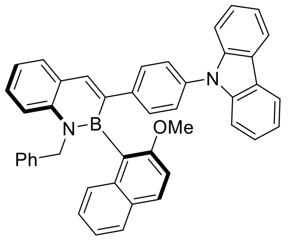

White solid, mp:73-75 ºC. Yield: 40%. R_f = 0.25 (silica gel, PE:DCM = 3:1).
^{1}H NMR (500 MHz, Chloroform-*d*) δ 8.24 (s, 1H), 8.10 (d, *J* = 7.6 Hz, 2H), 7.87 (d, *J* = 7.4 Hz, 1H), 7.80 (dd, *J* = 12.4, 8.2 Hz, 2H), 7.51 (dd, *J* = 18.4, 7.8 Hz, 2H), 7.38 (dt, *J* = 24.5, 7.4 Hz, 3H), 7.32 – 7.22 (m, 7H), 7.22 – 7.07 (m, 10H), 5.39 (d, *J* = 16.4 Hz, 1H), 5.26 (d, *J* = 16.4 Hz, 1H), 3.55 (s, 3H). **^{13}C NMR (101 MHz, Chloroform-*d*)** δ 159.0, 144.2, 142.4, 141.1, 141.0, 139.0, 136.3, 130.7, 130.2, 129.4, 129.2, 128.5, 128.5, 128.5, 127.3, 127.2, 126.7, 126.5, 126.4, 126.1, 125.8, 123.3, 123.3, 121.6, 120.3, 119.7, 117.2, 112.7, 109.9, 55.7, 53.6. **^{11}B NMR (128 MHz, Chloroform-*d*)** δ 41.7. **HRMS (ESI)** calcd for $C_{44}H_{34}BN_2O$ $[M+H]^+$: 617.2759, found: 617.2764. **HPLC analysis**: DAICEL CHIRALCEL IA-3, hexane/isopropanol = 90/10, 1.0 mL/min, λ = 254 nm, t_R (major) = 5.54 min, t_R (minor) = 7.01 min, 88% ee. **$[\alpha]^{25}_D$**: +130.6 (*c* 0.5, $CHCl_3$).

(*R*$_a$)- 1-benzyl-2-(2-methoxynaphthalen-1-yl)-3-phenyl-1,2-dihydrobenzo[e][1,2]azaborinine (3q)

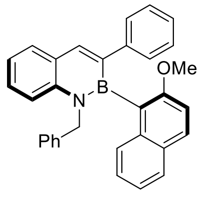

White solid, mp: 74-76 °C. Yield: 99%. R_f = 0.25 (silica gel, PE:DCM = 5:1).
^{1}H NMR (500 MHz, Chloroform-*d*) δ 8.13 (s, 1H), 7.88 – 7.81 (m, 1H), 7.77 (d, *J* = 8.6 Hz, 2H), 7.52 (dd, *J* = 5.6, 3.3 Hz, 1H), 7.47 (d, *J* = 8.5 Hz, 1H), 7.41 - 7.35 (m, 1H), 7.32 – 7.24 (m, 3H), 7.22 – 7.11 (m, 7H), 7.10 – 7.00 (m, 4H), 5.34 (d, *J* = 16.4 Hz, 1H), 5.20 (d, *J* = 16.4 Hz, 1H), 3.46 (s, 3H). **^{13}C NMR (126 MHz, Chloroform-*d*)** δ 159.0, 144.9, 142.4, 140.9, 139.1, 136.3, 130.6, 129.9, 129.0, 128.4, 128.3, 128.0, 127.6, 127.4, 127.2, 126.6, 126.5, 126.2, 125.7, 123.2, 121.4, 117.1, 112.8, 55.7, 53.4. **^{11}B NMR (128 MHz, Chloroform-*d*)** δ 40.5. **HRMS (ESI)** calcd for $C_{32}H_{27}BNO$ [M+H]$^+$: 452.2180, found: 452.2170. **HPLC analysis**: DAICEL CHIRALCEL IA-3, hexane/isopropanol = 98/2, 1mL/min, λ = 254 nm, t_R (major) = 5.87 min, t_R (minor) = 9.34 min, 97% ee. **[α]$^{25}_{D}$**: +290.2 (*c* 0.5, $CHCl_3$).

(*R*$_a$)-1-benzyl-3-(furan-3-yl)-2-(2-methoxynaphthalen-1-yl)-1,2-dihydrobenzo[e][1,2]azaborinine (3r)

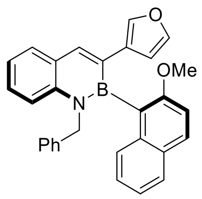

White solid, mp: 194-196 °C. Yield: 48%. R_f = 0.2 (silica gel, PE:DCM = 5:1).
^{1}H NMR (400 MHz, Chloroform-*d*) δ 8.25 (s, 1H), 7.90 (d, *J* = 9.0 Hz, 1H), 7.83 (t, *J* = 6.3 Hz, 2H), 7.47 – 7.39 (m, 2H), 7.39 – 7.18 (m, 8H), 7.14 (t, *J* = 5.8 Hz, 3H), 6.63 – 6.57 (m, 1H), 6.52 (s, 1H), 5.29 (d, *J* = 16.6 Hz, 1H), 5.18 (d, *J* = 16.6 Hz, 1H), 3.59 (s, 3H). **^{13}C NMR (101 MHz, Chloroform-*d*)** δ 158.8, 142.3, 140.7, 140.1, 139.7, 139.0, 136.1, 130.4, 130.2, 129.2, 128.4, 128.0, 127.6, 127.3, 127.0, 126.5, 126.4, 123.6, 121.5, 117.0, 117.0, 113.0, 109.0, 77.5, 55.8, 53.3. **^{11}B NMR (128 MHz, Chloroform-*d*)** δ 40.7. **HRMS (ESI)** calcd for $C_{30}H_{25}BNO_2$ [M+H]$^+$: 442.1973, found: 442.1975. **HPLC analysis**: DAICEL CHIRALCEL IA-3, hexane/isopropanol = 90/10, 1.0 mL/min, λ = 254 nm, t_R (major) = 6.77 min, t_R (minor) = 11.49 min, 83% ee. **[α]$^{25}_{D}$**: +324.8 (*c* 0.5, $CHCl_3$).

(*R*$_a$)-1-benzyl-2-(2-methoxynaphthalen-1-yl)-3-(thiophen-3-yl)-1,2-dihydrobenzo[e][1,2]azaborinine (3s)

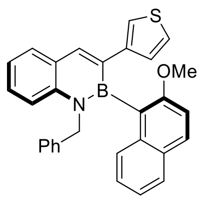

White solid, mp: 168 - 170 °C. Yield: 95%. R_f = 0.2 (silica gel, PE:DCM = 5:1).
^{1}H NMR (500 MHz, Chloroform-*d*) δ 8.23(s, 1H), 7.79 (dd, *J* = 14.4, 8.2 Hz, 3H), 7.44 (d, *J* = 7.6 Hz, 1H), 7.39 (d, *J* = 8.5 Hz, 1H), 7.35 – 7.29 (m, 1H), 7.29 - 7.18 (m, 3H), 7.17 – 7.05 (m, 6H), 6.98 (dd, *J* = 4.9, 3.0 Hz, 1H), 6.96 – 6.90 (m, 1H), 6.70 – 6.61 (m, 1H), 5.26 (d, *J* = 16.4 Hz, 1H), 5.13 (d, *J* = 16.4 Hz, 1H), 3.45(s, 3H). **^{13}C NMR (126 MHz, Chloroform-*d*)** δ 159.0, 144.84, 141.3, 140.9, 139.0, 136.2, 130.5, 130.0, 129.2, 128.4, 128.4, 128.2, 127.7, 127.3, 127.2, 126.6, 126.5, 126.4, 124.1, 123.4, 121.5, 120.8, 117.1, 113.2, 55.8, 53.3. **^{11}B NMR (128 MHz, Chloroform-*d*)** δ 40.3. **HRMS (ESI)** calcd for $C_{30}H_{25}BNOS$ [M+H]$^+$: 458.1744, found: 458.1746. **HPLC analysis**: DAICEL CHIRALCEL IA-3, hexane/isopropanol = 90/10, 1.0 mL/min, λ = 254 nm, t_R (major) = 5.66 min, t_R (minor) = 10.93 min, 82% ee. **[α]$^{25}_{D}$**: +298.4 (*c* 0.5, $CHCl_3$).

(R_a)-3-(benzofuran-3-yl)-1-benzyl-2-(2-methoxynaphthalen-1-yl)-1,2-dihydrobenzo[e][1,2]azaborinine (3t)

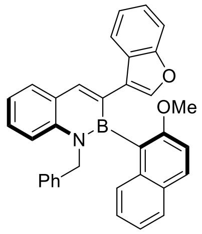

White solid, mp: 68-70 °C. Yield: 85%. R_f = 0.2 (silica gel, PE: DCM = 5:1).

^{1}H NMR (400 MHz, Chloroform-*d*) δ 8.15 (s, 1H), 7.84 (d, *J* = 7.5 Hz, 1H), 7.76 (d, *J* = 8.9 Hz, 2H), 7.56 – 7.44 (m, 3H), 7.43 – 7.36 (m, 2H), 7.34 – 7.24 (m, 4H), 7.24 – 7.12 (m, 5H), 7.11 – 7.01 (m, 2H), 6.61 – 6.51 (m, 1H), 5.33 (d, *J* = 16.4 Hz, 1H), 5.20 (d, *J* = 16.4 Hz, 1H), 3.46 (s, 3H). **^{13}C NMR (101 MHz, Chloroform-*d*)** δ 158.9, 153.7, 144.6, 142.5, 140.9, 139.9, 139.1, 136.3, 130.6, 129.8, 129.1, 128.4, 128.2, 127.4, 127.2, 127.0, 126.6, 126.5, 126.2, 125.0, 123.2, 121.4, 120.1, 117.1, 112.8, 110.2, 106.8, 55.6, 53.5. **^{11}B NMR (128 MHz, Chloroform-*d*)** δ 40.8. **HRMS (ESI)** calcd for $C_{34}H_{27}ClBNO_2$ $[M+H]^+$: 492.2129, found: 492.2137. **HPLC analysis**: DAICEL CHIRALCEL IA-3, hexane/isopropanol = 98/2, 1.0 mL/min, λ = 254 nm, t_R (major) = 7.48 min, t_R (minor) = 11.45 min, 96% ee. **$[\alpha]^{25}_D$**: +266.6 (*c* 0.5, $CHCl_3$).

(R_a)- (*E*)-1-benzyl-2-(2-methoxynaphthalen-1-yl)-3-styryl-1,2-dihydrobenzo[e][1,2]azaborinine (3u)

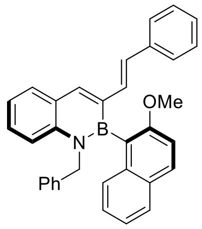

White solid, mp: 115 – 117 °C. Yield: 61%. R_f = 0.2 (silica gel, PE:DCM = 3:1).

^{1}H NMR (500 MHz, Chloroform-*d*) δ 8.29(s, 1H), 7.91 (d, *J* = 9.0 Hz, 1H), 7.83 (dd, *J* = 16.0, 7.7 Hz, 2H), 7.46 (d, *J* = 8.0 Hz, 1H), 7.40 (d, *J* = 8.5 Hz, 1H), 7.37 – 7.22 (m, 5H), 7.18 (q, *J* = 6.9, 5.7 Hz, 4H), 7.11 (dd, *J* = 16.1, 7.6 Hz, 6H), 6.98 (d, *J* = 16.2 Hz, 1H), 6.62 (d, *J* = 16.2 Hz, 1H), 5.28 (d, *J* = 16.3 Hz, 1H), 5.18 (d, *J* = 16.5 Hz, 1H), 3.65(s, 3H). **^{13}C NMR (126 MHz, Chloroform-*d*)** δ 158.9, 141.2, 140.3, 139.3, 138.4, 136.2, 132.1, 130.5, 130.1, 129.3, 128.7, 128.4, 128.4, 128.2, 127.5, 127.2, 126.9, 126.6, 126.4, 126.4, 123.5, 121.5, 117.1, 113.0, 55.9, 53.2. **^{11}B NMR (128 MHz, Chloroform-*d*)** δ 41.4. **HRMS (ESI)** calcd for $C_{34}H_{29}BNO$ $[M+H]^+$: 478.2337, found: 478.2341. **HPLC analysis**: DAICEL CHIRALCEL IA-3, hexane/isopropanol = 90/10, 1.0 mL/min, λ = 254 nm, t_R (major) = 6.02 min, t_R (minor) = 8.71 min, 40% ee. **$[\alpha]^{25}_D$**: +107.6 (*c* 0.5, $CHCl_3$).

(R_a)-1-benzyl-2-(2-methoxynaphthalen-1-yl)-3-(1-phenylvinyl)-1,2-dihydrobenzo[e][1,2]azaborinine (3v)

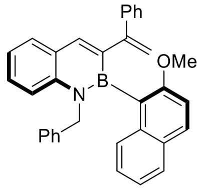

White solid, mp: 51-53 °C. Yield: 82%. R_f = 0.35 (silica gel, PE:DCM = 4:1).

^{1}H NMR (500 MHz, Chloroform-*d*) δ 8.01 (s, 1H), 7.76 (d, *J* = 7.8 Hz, 1H), 7.71 – 7.65 (m, 1H), 7.63 (d, *J* = 8.9 Hz, 1H), 7.42 (d, *J* = 8.2 Hz, 2H), 7.41 – 7.31 (m, 1H), 7.25 – 7.20 (m, 3H), 7.20 – 7.15 (m, 2H), 7.11 (dd, *J* = 15.5, 7.3 Hz, 3H), 7.02 – 6.92 (m, 5H), 6.89 (d, *J* = 9.0 Hz, 1H), 5.28 (d, *J* = 16.5 Hz, 1H), 5.20 (d, *J* = 16.5 Hz, 1H), 5.01 (d, *J* = 8.7 Hz, 2H), 3.50(s, 3H). **^{13}C NMR (101 MHz, Chloroform-*d*)** δ 158.2, 153.1, 142.7, 142.5, 141.2, 139.2, 136.2, 130.522, 129.6, 128.8, 128.3, 128.3, 128.0, 127.6, 127.4, 127.3, 127.1, 126.5, 126.5, 126.4, 125.6, 122.8, 121.3, 117.1, 113.2, 111.8, 55.0, 53.4. **^{11}B NMR (128 MHz, Chloroform-*d*)** δ 40.6. **HRMS (ESI)** calcd for C34H29BNO $[M+H]^+$: 478.2337, found: 478.2336. **HPLC analysis**: DAICEL CHIRALCEL AD-H, hexane/isopropanol = 99/1, 1.0 mL/min, λ = 254 nm, t_R (major) = 6.18 min, t_R (minor) = 7.32 min, 92% ee. **$[\alpha]^{25}_D$**: +167.4 (*c* 0.5, $CHCl_3$).

(*R$_a$*)-1-benzyl-2-(2-methoxynaphthalen-1-yl)-3-(prop-1-en-2-yl)-1,2-dihydrobenzo[e][1,2]azaborinine (3w)

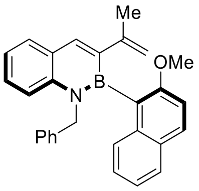

Light Yellow solid, mp: 53-55 °C. Yield: 88%. R_f = 0. 5 (silica gel, PE:DCM = 5:1).

^{1}H NMR (400 MHz, Chloroform-*d*) δ 7.99 (s, 1H), 7.87 – 7.68 (m, 3H), 7.40 (d, *J* = 7.2 Hz, 1H), 7.37 – 7.23 (m, 4H), 7.23 – 7.11 (m, 4H), 7.07 (t, *J* = 8.1 Hz, 3H), 5.20 (d, *J* = 16.5 Hz, 1H), 5.11 (d, *J* = 16.5 Hz, 1H), 4.64 (d, *J* = 11.6 Hz, 2H), 3.61 (s, 3H), 1.74 (s, 3H). **^{13}C NMR (101 MHz, Chloroform-*d*)** δ 158.6, 148.2, 141.0, 140.2, 139.2, 136.3, 130.4, 129.7, 129.1, 128.3, 128.3, 128.1, 127.5, 127.1, 126.5, 126.4, 126.0, 123.3, 121.3, 117.0, 112.6, 112.5, 55.6, 53.2, 23.5. **^{11}B NMR (128 MHz, Chloroform-*d*)** δ 39.8. **HRMS (ESI)** calcd for $C_{29}H_{27}BNO$ $[M+H]^+$: 416.2180, found: 416.2183. **HPLC analysis**: DAICEL CHIRALCEL AD-H, hexane/isopropanol = 99/1, 1.0 mL/min, λ = 254 nm, t_R (major) = 5.99 min, t_R (mnor) = 6.65 min, 88% ee. **$[\alpha]^{25}_D$**: +284.7 (*c* 0.5, $CHCl_3$).

(*R$_a$*)-1-benzyl-6-methoxy-2-(2-methoxynaphthalen-1-yl)-3-(3-methoxyphenyl)-1,2-dihydrobenzo[e][1,2]azaborinine (3x)

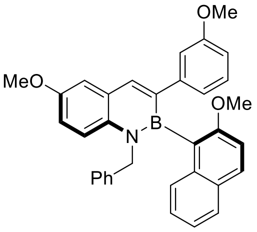

Yellow solid, mp: 69-71 °C. Yield: 99%. R_f = 0.1 (silica gel, PE:DCM = 3:1).

^{1}H NMR (400 MHz, Chloroform-*d*) δ 8.11 (s, 1H), 7.77 (d, *J* = 8.7 Hz, 2H), 7.55 (d, *J* = 7.9 Hz, 1H), 7.37 (d, *J* = 9.2 Hz, 1H), 7.34 – 7.25 (m, 3H), 7.23 – 7.00 (m, 8H), 6.89 (d, *J* = 7.5 Hz, 1H), 6.59 (d, *J* = 8.1 Hz, 2H), 5.33 (d, *J* = 16.4 Hz, 1H), 5.17 (d, *J* = 16.4 Hz, 1H), 3.91 (s, 3H), 3.48 (s, 3H), 3.20 (s, 3H). **^{13}C NMR (101 MHz, Chloroform-*d*)** δ 159.0, 158.7, 154.1, 146.2, 141.9, 139.1, 136. 5, 135.7, 129.8, 129.1, 128.6, 128.4, 128.4, 127.9, 127.3, 126.6, 126.5, 126.3, 123.2, 120.1, 118.3, 117.1, 113.0, 112.9, 112.4, 112.0, 55.8, 55.8, 54.6, 53.6. **^{11}B NMR (128 MHz, Chloroform-d)** δ 40.1. **HRMS (ESI)** calcd for $C_{34}H_{31}BNO_3$ $[M+H]^+$: 512.2392, found: 512.2397. IA-3, hexane/isopropanol = 90/10, 1.0 mL/min, λ = 254 nm, t_R (major) = 5.37 min, t_R (minor) = 7.46 min, 98% ee. **$[\alpha]^{25}_D$**: +245.5 (*c* 0.2, $CHCl_3$).

(R_a)-1-benzyl-2-(2-methoxynaphthalen-1-yl)-3-(3-methoxyphenyl)-6-methyl-1,2-dihydrobenzo[e][1,2]azaborinine (3y)

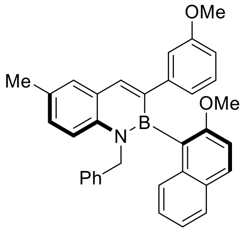

White solid, mp: 70 – 72 °C. Yield: 77%. R_f = 0.2 (silica gel, PE:DCM = 3:1).

^{1}H NMR (500 MHz, Chloroform-*d*) δ 8.07 (s, 1H), 7.75 (dd, *J* = 9.2, 2.8 Hz, 2H), 7.62 – 7.56 (m, 1H), 7.50 (d, *J* = 7.9 Hz, 1H), 7.32 (d, *J* = 8.6 Hz, 1H), 7.29 – 7.23 (m, 2H), 7.21 – 7.15 (m, 3H), 7.14 – 7.05 (m, 4H), 7.02 (t, *J* = 7.8 Hz, 1H), 6.85 (d, *J* = 7.6 Hz, 1H), 6.59 – 6.49 (m, 2H), 5.29 (d, *J* = 16.4 Hz, 1H), 5.15 (d, *J* = 16.4 Hz, 1H), 3.44 (s, 3H), 3.17 (s, 3H), 2.44 (s, 3H). **^{13}C NMR (101 MHz, Chloroform-*d*)** δ 159.0, 158.8.0, 146.4, 142.1, 139.2, 139.1, 136.5, 130.7, 130.4, 129.8, 129.7, 129.1, 128.6, 128.4, 128.4, 127.343, 127.3, 126.6, 126.5, 126.3, 123.2, 120.2, 117.0, 113.1, 112.8, 112.5, 55.8, 54.6, 53.5, 20.8. **^{11}B NMR (128 MHz, Chloroform-*d*)** δ 40.5. **HRMS (ESI)** calcd for $C_{34}H_{31}BNO_2$ $[M+H]^+$: 496.2442, found: 496.2448. **HPLC analysis**: DAICEL CHIRALCEL IA-3, hexane/isopropanol = 90/10, 1.0 mL/min, λ = 254 nm, t_R (major) = 4.72 min, t_R (minor) = 6.01 min, 97% ee. **$[\alpha]^{25}_D$**: +284.0 (*c* 0.2, $CHCl_3$).

(R_a)-1-benzyl-7-fluoro-2-(2-methoxynaphthalen-1-yl)-3-(3-methoxyphenyl)-1,2-dihydrobenzo[e][1,2]azaborinine (3z)

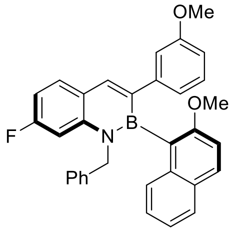

White solid, mp: 75-77 °C. Yield: 98%. R_f = 0.25 (silica gel, PE:DCM = 3:1).

^{1}H NMR (500 MHz, Chloroform-*d*) δ 8.14 (s, 1H), 7.86 – 7.71 (m, 3H), 7.54 (d, *J* = 8.1 Hz, 1H), 7.40 – 7.27 (m, 2H), 7.26 – 7.14 (m, 6H), 7.13 – 6.98 (m, 3H), 6.90 (d, *J* = 7.6 Hz, 1H), 6.62 (d, *J* = 9.0 Hz, 2H), 5.36 (d, *J* = 16.4 Hz, 1H), 5.13 (d, *J* = 16.4 Hz, 1H), 3.50 (s, 3H), 3.22 (s, 3H). **^{13}C NMR (126 MHz, Chloroform-*d*)** δ 163.7, 161.7, 158.9, 158.8, 145.9, 142.3 (d, *J* = 10.9 Hz), 141.9 – 141.29 (m), 138.3, 136.3, 132.1 (d, *J* = 10.3 Hz), 130.0, 129.1, 128.7, 128.5, 128.4, 127.1, 126.8, 126.5, 126.4, 123.9 (d, *J* = 2.2 Hz), 123.3, 120.1, 112.9 (d, *J* = 11.7 Hz), 112.5, 109.8 (d, *J* = 23.1 Hz), 103.8 (d, *J* = 26.0 Hz), 55.7, 54.6, 53.7. **^{19}F NMR (471 MHz, Chloroform-*d*)** δ -110.5. **^{11}B NMR (128 MHz, Chloroform-*d*)** δ 40.7. **HRMS (ESI)** calcd for $C_{33}H_{28}BFNO_2$ $[M+H]^+$: 500.2192, found: 500.2198. **HPLC analysis**: DAICEL CHIRALCEL IA-3, hexane/isopropanol = 90/10, 1.0 mL/min, λ = 254 nm, t_R (major) = 4.92 min, t_R (minor) = 6.15 min, 96% ee. **$[\alpha]^{25}_D$**: +248.5 (*c* 0.2, $CHCl_3$).

(R_a)-4-benzyl-3-(2-methoxynaphthalen-1-yl)-2-(3-methoxyphenyl)-3,4-dihydronaphtho[1,2-e][1,2]azaborinine (3aa)

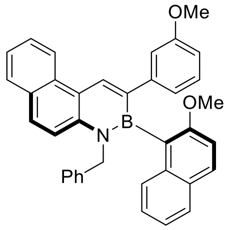

White solid, mp: 64-66 °C. Yield: 75%. R_f = 0.2 (silica gel, PE:DCM = 1:1).

^{1}H NMR (400 MHz, Chloroform-*d*) δ 9.18 (s, 1H), 8.76 (d, *J* = 8.4 Hz, 1H), 7.99 – 7.60 (m, 6H), 7.52 (q, *J* = 7.5, 6.2 Hz, 2H), 7.35 – 7.03 (m, 9H), 6.98 (d, *J* = 7.3 Hz, 1H), 6.75 – 6.55 (m, 2H), 5.47 (d, *J* = 16.6 Hz, 1H), 5.35 (d, *J* = 16.6 Hz, 1H), 3.47 (s, 3H), 3.22 (s, 3H). **^{13}C NMR (101 MHz, Chloroform-*d*)** δ 159.0 , 158.8, 146.9, 139.6, 139.2, 136.9, 136.5, 131.7, 129.8, 129.2, 129.1, 128.7, 128.5, 128.5, 128.4, 127.4, 127.1, 126.7, 126.5, 126.3, 124.8, 123.3, 122.3, 120.9, 120.4, 117.8, 113.0, 112.8, 112.7, 55.8, 54.7, 54.1. **^{11}B NMR (128 MHz, Chloroform-*d*)** δ 40.2. **HRMS (ESI)** calcd for $C_{37}H_{31}BNO_2$ $[M+H]^+$: 532.2442, found: 532.2442. **HPLC analysis**: DAICEL CHIRALCEL IA-3, hexane/isopropanol = 98/2, 1.0 mL/min, λ = 254 nm, t_R (major) = 10.01 min, t_R (minor) = 20.18 min, 97% ee. **$[\alpha]^{25}_D$**: +188.0 (*c* 0.5, $CHCl_3$).

(R_a)-2-(2-methoxynaphthalen-1-yl)-3-(3-methoxyphenyl)-1-(4-methylbenzyl)-1,2-dihydrobenzo[e][1,2]azaborinine (3ab)

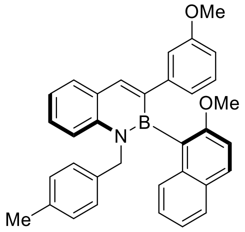

Yellow solid, mp: 61-63 °C. Yield: 97%. R_f = 0.1 (silica gel, PE:DCM = 5:1).

^{1}H NMR (500 MHz, Chloroform-*d*) δ 8.12 (s, 1H), 7.80 (d, *J* = 7.7 Hz, 1H), 7.74 (d, *J* = 9.0 Hz, 2H), 7.50 (d, *J* = 8.0 Hz, 1H), 7.44 (d, *J* = 8.5 Hz, 1H), 7.40 – 7.33 (m, 1H), 7.32 – 7.21 (m, 3H), 7.10 – 6.95 (m, 6H), 6.88 – 6.83 (m, 1H), 6.60 – 6.51 (m, 2H), 5.28 (d, *J* = 16.3 Hz, 1H), 5.13 (d, *J* = 16.3 Hz, 1H), 3.46 (s, 3H), 3.16 (s, 3H),2.23 (s, 3H). **^{13}C NMR (101 MHz, Chloroform-*d*)** δ 159.0, 158.8, 146.3, 142.3, 141.1, 136.4, 136.1, 136.0, 130.7, 129.8, 129.2, 129.1, 128.6, 128.4, 128.3, 127.3, 126.4, 126.3, 123.2, 121.4, 120.2, 117.2, 113.1, 112.9, 112.5, 55.8, 54.6, 53.3, 21.1. **^{11}B NMR (128 MHz, Chloroform-*d*)** δ 40.5. **HRMS (ESI)** calcd for $C_{34}H_{31}BNO_2$ $[M+H]^+$: 496.2442, found: 496.2438. **HPLC analysis**: DAICEL CHIRALCEL IA-3, hexane/isopropanol = 90/10, 1.0 mL/min, λ = 254 nm, t_R (major) = 4.60 min, t_R (minor) = 5.38 min, 95% ee. **$[\alpha]^{25}_D$**: +261.0 (*c* 0.2, $CHCl_3$).

(R_a)-1-(4-fluorobenzyl)-2-(2-methoxynaphthalen-1-yl)-3-(3-methoxyphenyl)-1,2-dihydrobenzo[e][1,2]azaborinine (3ac)

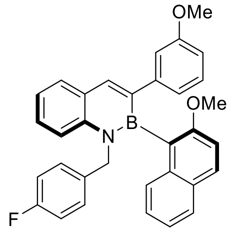

White solid, mp: 52 – 54 ºC. Yield: 99%. R_f = 0.15 (silica gel, PE:DCM = 5:1).
^{1}H NMR (500 MHz, Chloroform-*d*) δ 8.15 (s, 1H), 7.83 (d, *J* = 7.5 Hz, 1H), 7.80 – 7.74 (m, 2H), 7.50 (d, *J* = 7.8 Hz, 1H), 7.44 – 7.36 (m, 2H), 7.33 – 7.22 (m, 3H), 7.15 – 7.07 (m, 3H), 7.03 (t, *J* = 7.9 Hz, 1H), 6.92 – 6.82 (m, 3H), 6.62 – 6.52 (m, 2H), 5.29 (d, *J* = 16.3 Hz, 1H), 5.16 (d, *J* = 16.3 Hz, 1H), 3.47 (s, 3H), 3.18 (s, 3H). **^{13}C NMR (101 MHz, Chloroform-*d*)** δ 162.9, 160.5, 158.9, 158.8, 146.1, 142.4, 140.8, 136.4, 134.6 (d, *J* = 3.1 Hz), 130.8, 129.9, 129.1, 128.7, 128.4 (d, *J* = 6.1 Hz), 128.0 (d, *J* = 7.9 Hz), 127.3, 127.2, 126.4, 123.3, 121.6, 120.1, 116.9, 115.3, 115.1, 113.0 (d, *J* = 11.0 Hz), 112.5, 55.8, 54.6, 52.8. **^{19}F NMR (471 MHz, Chloroform-*d*)** δ -116.4. **^{11}B NMR (128 MHz, Chloroform-*d*)** δ 41.0. **HRMS (ESI)** calcd for $C_{33}H_{28}BFNO_2$ $[M+H]^+$: 500.2192, found: 500.2197.**HPLC analysis**: DAICEL CHIRALCEL IA-3, hexane/isopropanol = 90/10, 1.0 mL/min, λ = 254 nm, t_R (major) = 5.20min, t_R (minor) = 6.42 min, 96% ee. **$[\alpha]^{25}_D$**: +205.3 (*c* 0.2, $CHCl_3$).

(R_a)-1-(4-chlorobenzyl)-2-(2-methoxynaphthalen-1-yl)-3-(3-methoxyphenyl)-1,2-dihydrobenzo[e][1,2]azaborinine (3ad)

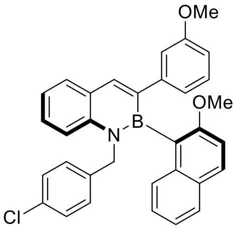

White solid, mp: 72 – 74 ºC. Yield: 78%. R_f = 0.3 (silica gel, PE:DCM = 5:1).
^{1}H NMR (500 MHz, Chloroform-*d*) δ 8.15 (s, 1H), 7.83 (d, *J* = 7.5 Hz, 1H), 7.80 – 7.73 (m, 2H), 7.50 (d, *J* = 7.9 Hz, 1H), 7.42 – 7.35 (m, 2H), 7.33 – 7.23 (m, 3H), 7.15 (d, *J* = 8.4 Hz, 2H), 7.11 – 6.99 (m, 4H), 6.87 (d, *J* = 7.5 Hz, 1H), 6.62 – 6.52 (m, 2H), 5.29 (d, *J* = 16.5 Hz, 1H), 5.15 (d, *J* = 16.5 Hz, 1H), 3.47 (s, 3H), 3.19 (s, 3H). **^{13}C NMR (126 MHz, Chloroform-*d*)** δ 158.9, 158.8, 146.0, 142.4, 140.7, 137.5, 136.3, 132.3, 130.8, 130.0, 129.1, 128.7, 128.5, 128.5, 128.4, 127.9, 127.3, 127.2, 126.4, 123.4, 121.6, 120.1, 116.8, 113.0, 112.9, 112.5, 55.8, 54.6, 52.8. **^{11}B NMR (128 MHz, Chloroform-*d*)** δ 41.7. **HRMS (ESI)** calcd for $C_{33}H_{28}BClNO_2$ $[M+H]^+$: 516.1896, found: 516.1903. **HPLC analysis**: DAICEL CHIRALCEL IA-3, hexane/isopropanol = 90/10, 1.0 mL/min, λ = 254 nm, t_R (major) = 5.24 min, t_R (minor) = 6.46 min, 97% ee. **$[\alpha]^{25}_D$**: +274.5 (*c* 0.2, $CHCl_3$).

(R_a)-1-butyl-2-(2-methoxynaphthalen-1-yl)-3-(3-methoxyphenyl)-1,2-dihydrobenzo[e][1,2]azaborinine (3ae)

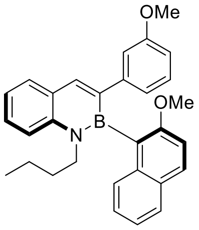

Yelow oil. Yield: 99%. R_f = 0.15 (silica gel, PE:DCM = 5:1).

^{1}H NMR (500 MHz, Chloroform-*d*) δ 8.10 (s, 1H), 7.89 – 7.77 (m, 3H), 7.67 (d, *J* = 8.5 Hz, 1H), 7.62 – 7.55 (m, 1H), 7.51 – 7.46 (m, 1H), 7.36 – 7.27 (m, 3H), 7.20 (d, *J* = 9.0 Hz, 1H), 7.02 (t, *J* = 7.9 Hz, 1H), 6.83 (d, *J* = 7.6 Hz, 1H), 6.62 – 6.55 (m, 1H), 6.55 – 6.51 (m, 1H), 4.13 – 3.85 (m, 2H), 3.60 (s, 3H), 3.18 (s, 3H), 1.80 – 1.64 (m, 2H), 1.18 – 1.04 (m, 2H), 0.65 (t, *J* = 7.4 Hz, 3H). **^{13}C NMR (101 MHz, Chloroform-*d*)** δ 158.7, 158.6, 146.3, 142.0, 141.0, 136.5, 131.0, 129.7, 129.2, 128.6, 128.4, 128.3, 127.5, 127.2, 126.2, 123.3, 121.1, 120.2, 115.5, 113.2, 112.8, 112.4, 56.1, 54.6, 49.1, 31.7, 20.2, 13.7. **^{11}B NMR (128 MHz, Chloroform-*d*)** δ 38.6. **HRMS (ESI)** calcd for $C_{30}H_{31}BNO_2$ $[M+H]^+$: 448.2442, found: 448.2442. **HPLC analysis**: DAICEL CHIRALCEL IA-3, hexane/isopropanol = 90/10, 1.0 mL/min, λ = 254 nm, t_R (major) = 4.16 min, t_R (minor) = 4.72 min, 93% ee. **$[\alpha]^{25}_D$**: +30. 9 (*c* 0.45, $CHCl_3$).

(R_a)-2-(2-methoxynaphthalen-1-yl)-3-(3-methoxyphenyl)-1-(thiophen-2-ylmethyl)-1,2-dihydrobenzo[e][1,2]azaborinine (3af)

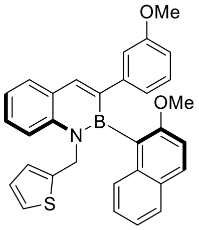

White solid, mp: 136 – 138 °C. Yield: 82%. R_f = 0.1 (silica gel, PE:DCM = 5:1).

^{1}H NMR (500 MHz, Chloroform-*d*) δ 8.11 (s, 1H), 7.89 – 7.73 (m, 3H), 7.67 (d, *J* = 8.3 Hz, 1H), 7.46 (d, *J* = 5.9 Hz, 2H), 7.35 – 7.22 (m, 3H), 7.12 (d, *J* = 8.8 Hz, 1H), 7.05 – 6.97 (m, 2H), 6.88 – 6.69 (m, 3H), 6.55 (d, *J* = 12.9 Hz, 2H), 5.40 (m, 2H), 3.51 (s, 3H), 3.17 (s, 3H). **^{13}C NMR (126 MHz, Chloroform-*d*)** δ 159.0, 158.7, 146.1, 142.5, 142.4, 140.8, 136.4, 130.8, 130.0, 129.1, 128.6, 128.4, 128.4, 127.3, 126.7, 126.3, 125.1, 123.9, 123.3, 121.6, 120.1, 116.5, 113.0, 112.9, 112.4, 55.8, 54.6, 48.8. **^{11}B NMR (128 MHz, Chloroform-*d*)** δ 40.3. **HRMS (ESI)** calcd for $C_{31}H_{27}BNO_2S$ $[M+H]^+$: 488.1850, found: 488.1854. **HPLC analysis**: DAICEL CHIRALCEL IA-3, hexane/isopropanol = 90/10, 1.0 mL/min, λ = 254 nm, t_R (major) = 5.20 min, t_R (minor) = 6.62 min, 97% ee. **$[\alpha]^{25}_D$**: +137.5 (*c* 0.2, $CHCl_3$).

(R_a)-2-(2,6-dimethoxynaphthalen-1-yl)-1-(naphthalen-2-ylmethyl)-2,3-dihydro-1H-naphtho[1,8-de][1,3,2]diazaborinine (3ag)

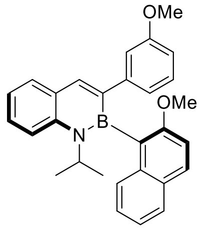

White solid, mp: 52 – 54 ºC. Yield: 33%. R_f = 0.3 (silica gel, PE:DCM = 1:1).

^{1}H NMR (400 MHz, Chloroform-*d*) δ 8.04 (s, 1H), 7.96 (d, *J* = 8.7 Hz, 1H), 7.88 – 7.75 (m, 3H), 7.60 – 7.47 (m, 2H), 7.35 – 7.25 (m, 3H), 7.19 (d, *J* = 9.0 Hz, 1H), 6.98 (t, *J* = 7.9 Hz, 1H), 6.77 (d, *J* = 7.5 Hz, 1H), 6.54 (dd, *J* = 8.1, 1.9 Hz, 1H), 6.45 (s, 1H), 4.99 – 4.74 (m, 1H), 3.64 (s, 3H), 3.16 (s, 3H), 1.61 (dd, *J* = 23.8, 7.0 Hz, 6H). **^{13}C NMR (101 MHz, Chloroform-*d*)** δ 158.6, 158.2, 146.4, 142.4, 140.0, 136.5, 131.4, 129.4, 129.2, 128.5, 128.3, 127.7, 127.2, 126.0, 123.3, 120.8, 120.2, 118.7, 113.1, 112.7, 112.4, 56.1, 54.6, 53.3, 21.8, 21.7. **^{11}B NMR (128 MHz, Chloroform-*d*)** δ 39.1. **HRMS (ESI)** calcd for $C_{29}H_{29}BNO_2$ $[M+H]^+$: 434.2286, found: 434.2286. **HPLC analysis**: DAICEL CHIRALCEL OZ-3, hexane/isopropanol = 99/1, 0.5 mL/min, λ = 254 nm, t_R (major) = 18.63 min, t_R (minor) = 23.74 min, 97% ee. **$[\alpha]^{25}_D$**: -334.0 (*c* 0.25, $CHCl_3$).

(R_a)-1-benzyl-2-(2-methoxy-6-methylnaphthalen-1-yl)-3-(3-methoxyphenyl)-1,2-dihydrobenzo[e][1,2]azaborinine (3ah)

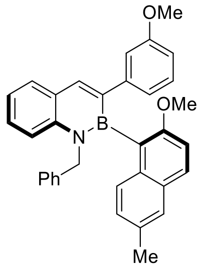

White solid, mp: 66 – 68 ºC. Yield: 99%. R_f = 0.35 (silica gel, PE:DCM = 3:1).

^{1}H NMR (500 MHz, Chloroform-*d*) δ 8.13 (s, 1H), 7.80 (d, *J* = 7.8 Hz, 1H), 7.66 (d, *J* = 9.0 Hz, 1H), 7.52 (s, 1H), 7.45 – 7.33 (m, 3H), 7.24 (t, *J* = 7.4 Hz, 1H), 7.20 – 7.15 (m, 2H), 7.15 – 7.07 (m, 4H), 7.02 (dd, *J* = 14.9, 8.1 Hz,2H), 6.88 – 6.82 (m, 1H), 6.61 – 6.52 (m, 2H), 5.31 (d, *J* = 16.4 Hz,1H), 5.16 (d, *J* = 16.4 Hz, 1H), 3.43 (s, 3H), 3.22 (s, 3H), 2.43 (s, 3H). **^{13}C NMR (101 MHz, Chloroform-*d*)** δ 158.8, 158.4, 146.2, 142.3, 141.0, 139.1, 134.5, 132.6, 130.7, 129.4, 129.1, 128.6, 128.4, 128.3, 127.3, 127.3, 127.1, 126.6, 126.5, 121.4, 120.2, 117.1, 113.1, 112.8, 112.6, 55.8, 54.7, 53.5, 21.6. **^{11}B NMR (128 MHz, Chloroform-*d*)** δ 40.9. **HRMS (ESI)** calcd for $C_{34}H_{31}BNO_2$ $[M+H]^+$: 496.2442, found: 496.2451. **HPLC analysis**: DAICEL CHIRALCEL AD-H, hexane/isopropanol = 99/1, 1.0 mL/min, λ = 254 nm, t_R (major) = 10.56 min, t_R (minor) = 54.70 min, 99% ee. **$[\alpha]^{25}_D$**: +294.0 (*c* 0.2, $CHCl_3$).

(R_a)-1-benzyl-2-(2-ethoxynaphthalen-1-yl)-3-(3-methoxyphenyl)-1,2-dihydrobenzo[e][1,2]azaborinine (3ai)

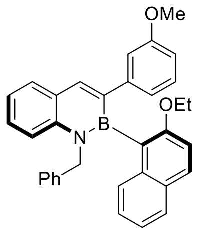

White solid, mp: 54-56 °C. Yield: 87%. R_f = 0.3 (silica gel, PE:DCM = 3:1).

^{1}H NMR (500 MHz, Chloroform-*d*) δ 8.15 (s, 1H), 7.83 (dd, *J* = 7.8, 1.5 Hz, 1H), 7.80 – 7.77 (m, 1H), 7.75 (d, *J* = 9.0 Hz, 1H), 7.57 (d, *J* = 8.3 Hz, 1H), 7.46 (d, *J* = 8.5 Hz, 1H), 7.41 – 7.24 (m, 4H), 7.23 – 7.16 (m, 4H), 7.15 – 7.10 (m, 1H), 7.08 – 7.01 (m, 1H), 6.89 – 6.85 (m, 1H), 6.63 – 6.58 (m, 1H), 6.52 (dd, *J* = 2.4, 1.7 Hz, 1H), 5.40 (d, *J* = 16.3 Hz, 1H), 5.20 (d, *J* = 16.3 Hz, 1H), 3.91 – 3.82 (m, 1H), 3.58 – 3.49 (m, 1H), 3.15 (s, 3H), 1.14 (t, *J* = 7.0 Hz, 3H). **^{13}C NMR (126 MHz, Chloroform-*d*)** δ 158.69, 158.31, 146.26, 142.17, 140.90, 139.11, 136.58, 130.63, 129.67, 129.06, 128.59, 128.42, 128.34, 128.22, 127.38, 127.14, 126.63, 126.58, 126.35, 123.14, 121.38, 120.15, 117.09, 113.69, 112.91, 112.39, 63.57, 54.55, 53.51, 15.08. **^{11}B NMR (160 MHz,)** δ 36.7. **HRMS (ESI)** calcd for $C_{34}H_{31}BNO_2$ $[M+H]^+$: 496.2442, found: 496.2446. **HPLC analysis**: DAICEL CHIRALCEL IA-3, hexane/isopropanol = 98/2, 1.0 mL/min, λ = 254 nm, t_R (major) = 5.74 min, t_R (minor) = 7.89 min, 89% ee. **$[\alpha]^{25}_D$**: +231.1 (*c* 0.5, *n*-Hexane).

(R_a)-1-benzyl-2-(2-(ethylthio)naphthalen-1-yl)-3-(3-methoxyphenyl)-1,2-dihydrobenzo[e][1,2]azaborinine (3aj)

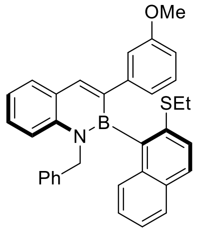

White solid, mp: 138-140 °C. Yield: 67%. R_f = 0.3 (silica gel, PE:DCM = 3:1).

^{1}H NMR (500 MHz, Chloroform-*d*) δ 8.17 (s, 1H), 7.84 (dd, *J* = 7.7, 1.4 Hz, 1H), 7.81 – 7.76 (m, 1H), 7.71 (d, *J* = 8.6 Hz, 1H), 7.64 – 7.59 (m, 1H), 7.43 – 7.32 (m, 5H), 7.29 – 7.23 (m, 1H), 7.20 – 7.14 (m, 2H), 7.11 (t, *J* = 6.9 Hz, 3H), 7.07 – 6.99 (m, 1H), 6.89 – 6.82 (m, 1H), 6.58 (ddd, *J* = 8.2, 2.6, 0.9 Hz, 1H), 6.43 – 6.38 (m, 1H), 5.30 (d, *J* = 16.7 Hz, 1H), 5.18 (d, *J* = 16.7 Hz, 1H), 3.05 (s, 3H), 2.66 – 2.48 (m, 1H), 2.44 – 2.20 (m, 1H), 0.95 (t, *J* = 7.4 Hz, 3H). **^{13}C NMR (126 MHz, Chloroform-*d*)** δ 158.8, 145.8, 142.8, 140.9, 138.5, 137.2, 136.0, 131.7, 130.8, 128.8, 128.5, 128.4, 128.4, 128.0, 127.5, 127.3, 126.7, 126.5, 126.4, 125.5, 121.6, 120.4, 117.3, 113.2, 54.5, 53.3, 29.2, 14.0. **^{11}B NMR (160 MHz,)** δ 36.8. **HRMS (ESI)** calcd for $C_{34}H_{31}BNOS$ $[M+H]^+$: 512.2214, found: 512.2219. **HPLC analysis**: DAICEL CHIRALCEL IA-3, hexane/isopropanol = 98/2, 1.0 mL/min, λ = 254 nm, t_R (minor) = 7.19 min, t_R (major) = 10.14 min, 30% ee. **$[\alpha]^{25}_D$**: -22.4 (*c* 0.5, $CHCl_3$).

(R_a)-ethyl-2-(4-(1-benzyl-2-(2-methoxynaphthalen-1-yl)-1,2-dihydrobenzo[e][1,2]azaborinin-3-yl)phenoxy)-2-methylpropanoate (4a)

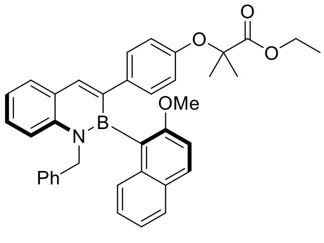

Yellowish oil. Yield: 92%. R_f = 0.45 (silica gel, PE:EtOAc = 2.5:1).

^{1}H NMR (400 MHz, Chloroform-*d*) δ 8.12 (s, 1H), 7.84 (d, *J* = 7.7 Hz, 1H), 7.81 – 7.74 (m, 2H), 7.54 – 7.43 (m, 2H), 7.39 (t, *J* = 7.7 Hz, 1H), 7.29 (dd, *J* = 6.5, 3.0 Hz, 3H), 7.25 – 7.13 (m, 5H), 7.10 (d, *J* = 9.0 Hz, 1H), 7.00 (d, *J* = 8.5 Hz, 2H), 6.57 (d, *J* = 8.5 Hz, 2H), 5.36 (d, *J* = 16.4 Hz, 1H), 5.22 (d, *J* = 16.4 Hz, 1H), 4.17 (q, *J* = 7.1 Hz, 2H), 3.49 (s, 3H), 1.54 (d, *J* = 3.3 Hz, 6H), 1.15 (t, *J* = 7.1 Hz, 3H). **^{13}C NMR (126 MHz, Chloroform-*d*)** δ 174.5, 158.9, 153.5, 141.7, 140.8, 139.1, 138.9, 136.2, 130.5, 129.9, 129.1, 128.7, 128.6, 128.4, 128.1, 127.4, 127.2, 126.5, 126.1, 123.1, 121.3, 118.4, 117.1, 112.8, 79.1, 61.4, 55.7, 53.4, 25.4, 25.4, 14.1. **^{11}B NMR (128 MHz, Chloroform-*d*)** δ 41.2. **HRMS (ESI)** calcd for $C_{38}H_{37}BNO_4$ $[M+H]^+$: 582.2810, found: 582.2810. **HPLC analysis**: DAICEL CHIRALCEL IA-3, hexane/isopropanol = 99/1, 0.5 mL/min, λ = 254 nm, t_R (major) = 25.17 min, t_R (minor) = 31.26 min, 96% ee. **$[\alpha]^{25}_D$**: +165.6 (*c* 0.66, $CHCl_3$).

(R_a)-(8R,9S,13S,14S)-2-(1-benzyl-2-(2-methoxynaphthalen-1-yl)-1,2-dihydrobenzo[e][1,2]azaborinin-3-yl)-13-methyl-6,7,8,9,11,12,13,14,15,16-decahydro-17H-cyclopenta[a]phenanthren-17-one (4b)

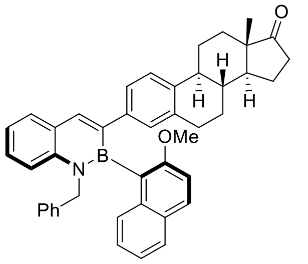

White solid, mp: 100-102 °C. Yield: 48%, dr = 15:1. R_f = 0.4 (silica gel, PE:EtOAc = 8:1).

^{1}H NMR (500 MHz, Chloroform-*d*) δ 8.13 (s, 1H), 7.85 – 7.72 (m, 3H), 7.46 (dd, *J* = 18.5, 7.5 Hz, 2H), 7.36 (t, 1H), 7.31 – 7.22 (m, 3H), 7.19 (t, 2H), 7.13 (q, *J* = 16.9, 8.5 Hz, 4H), 7.00 – 6.89 (m, 3H), 5.30 (d, *J* = 16.5 Hz, 1H), 5.18 (d, *J* = 16.4 Hz, 1H), 3.50 (s, 3H), 2.80 – 2.59 (m, 2H), 2.49 (dd, *J* = 19.1, 8.7 Hz, 1H), 2.38 – 2.23 (m, 1H), 2.22 – 2.08 (m, 2H), 2.08 – 1.99 (m, 1H), 1.91 (d, *J* = 8.7 Hz, 2H), 1.62 – 1.53 (m, 2H), 1.51 – 1.38 (m, 4H), 0.88 (s, 3H). **^{13}C NMR (126 MHz, Chloroform-*d*)** δ 221.2, 158.9, 142.2, 140.9, 139.1, 137.0, 136.3, 135.4, 130.5, 129.7, 129.0, 128.6, 128.3, 128.3, 128.1, 127.3, 126.5, 126.5, 126.1, 125.3, 124.5, 123.1, 121.3, 117.0, **^{11}B NMR (128 MHz, Chloroform-*d*)** δ 42.0. **HRMS (ESI)** calcd for $C_{44}H_{43}BNO_2$ $[M+H]^+$: 628.3381, found: 628.3381. **HPLC analysis**: DAICEL CHIRALCEL IA-3, hexane/isopropanol = 90/10, 1.0 mL/min, λ = 254 nm, t_R (major) = 8.99 min, t_R (minor) = 10.58 min, dr = 94:6. **$[\alpha]^{25}_D$**: +19.6 (*c* 0.5, $CHCl_3$).

(*Ra*)-ethyl-2-(4-(1-benzyl-2-(2-methoxynaphthalen-1-yl)-1,2-dihydrobenzo[e][1,2]azaborinin-3-yl)phenoxy)-2-methylpropanoate (4c)

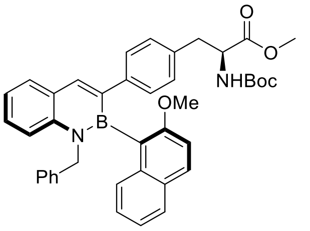

White solid, mp: 67-69 °C. Yield: 37%, dr = 49:1. R_f = 0.4 (silica gel, PE:EtOAc = 3:1).
^{1}H NMR (500 MHz, Chloroform-*d*) δ 8.12 (s, 1H), 7.83 (d, *J* = 6.7 Hz, 1H), 7.79 (d, *J* = 8.5 Hz, 2H), 7.55 – 7.43 (m, 2H), 7.38 (t, *J* = 8.1 Hz, 1H), 7.31 – 7.23 (m, 3H), 7.22 – 7.17 (m, 2H), 7.17 – 7.02 (m, 6H), 6.86 – 6.77 (m, 2H), 5.34 (d, *J* = 16.3 Hz, 1H), 5.20 (d, *J* = 16.3 Hz, 1H), 4.88 (s, 1H), 4.49 (s, 1H), 3.54 (s, 3H), 3.45 (s, 3H), 2.95 (s, 2H), 1.47 (s, 9H). **^{13}C NMR (126 MHz, Chloroform-*d*)** δ 172.4, 159.0, 155.2, 143.6, 142.2, 140.9, 1390, 136.2, 133.1, 130.6, 129.9, 129.0, 128.5, 128.4, 128.2, 128.1, 127.3, 127.1, 126.6, 126.5, 126.2, 123.2, 121.4, 117.1, 112.8, 79.9, 55.6, 54.5, 53.4, 52.2, 38.0, 28.4. **^{11}B NMR (128 MHz, Chloroform-*d*)** δ 41.8. **HRMS (ESI)** calcd for $C_{41}H_{42}BN_2O_5$ $[M+H]^+$: 653.3484, found: 653.3184. **HPLC analysis**: DAICEL CHIRALCEL OD-3, hexane/isopropanol = 99/1, 1.0 mL/min, λ = 254 nm, t_R (minor) = 29.01 min, t_R (major) = 37.64 min, dr = 98:2. **$[\alpha]^{25}_{D}$**: +234.0 (*c* 0.50, $CHCl_3$).

2.5 General Procedure for the synthesis of atropisomers with adjacent diaxes of C-B and C-C bonds

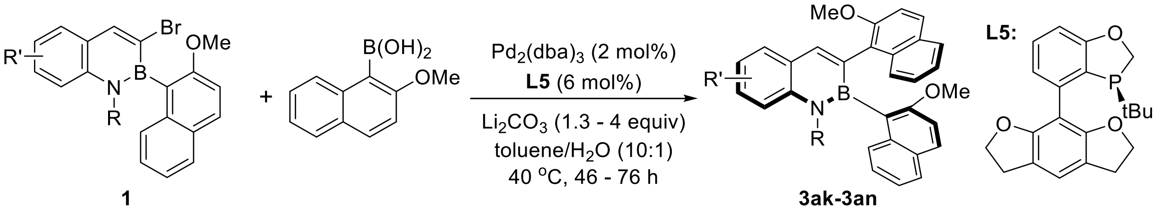

In air, a 25 mL schlenk tube was charged with **1** (0.1 mmol, 1.0 equiv), (2-methoxy-1-naphthyl)boronic acid (0.13 mmol - 4.0 mmol, 1.3 equiv - 4.0 equiv), $Pd_2(dba)_3$ (2 mol%), **L5** (6 mol%) and Li_2CO_3 (0.2 mmol - 0.4 mmol, 2 equiv - 4.0 equiv). The tube was evacuated and filled with argon for three cycles. Then, 1.5 mL of toluene and 150 μL water was added under argon. The reaction was allowed to stir at 40 °C for 46 - 76 hours. Upon completion, proper amount of silica gel was added to the reaction mixture. After removal of the solvent, the crude reaction mixture was purified on silica gel (petroleum ether and dichloromethane) to afford the desired products.

(*Ra,Sa*)-1-benzyl-2,3-bis(2-methoxynaphthalen-1-yl)-1,2-dihydrobenzo[e][1,2]azaborinine (3ak)

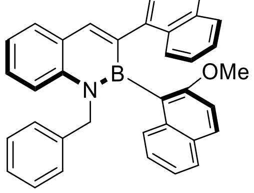

In air, a 25 mL schlenk tube was charged with **1** (0.1 mmol, 1.0 equiv), (2-methoxy-1-naphthyl)boronic acid (0.13 mmol, 1.3 equiv), $Pd_2(dba)_3$ (2 mol%), **L5** (6 mol%) and Li_2CO_3 (0.2 mmol, 2 equiv). The tube was evacuated and filled with argon for three cycles. Then, 1.5 mL of toluene and 150 μL water was added under

argon. The reaction was allowed to stir at 40 °C for 46 hours. Upon completion, proper amount of silica gel was added to the reaction mixture. After removal of the solvent, the crude reaction mixture was purified on silica gel (petroleum ether and dichloromethane) to afford the desired products.White solid, mp: 70-72 °C. Yield: 48%. R_f = 0.4 (silica gel, PE:DCM = 3:1).

^{1}H NMR (500 MHz, Acetone-*d*$_6$) δ 8.01 – 7.95 (m, 2H), 7.83 (dd, *J* = 7.7, 1.6 Hz, 1H), 7.80 – 7.76 (m, 1H), 7.69 (d, *J* = 8.1 Hz, 1H), 7.66 (d, *J* = 8.2 Hz, 1H), 7.62 (d, *J* = 8.6 Hz, 1H), 7.56 (dd, *J* = 8.9, 6.2 Hz, 2H), 7.42 (ddd, *J* = 8.7, 7.2, 1.7 Hz, 1H), 7.36 (m, 1.6 Hz, 2H), 7.30 – 7.26 (m, 3H), 7.26 – 7.15 (m, 4H), 7.10 (t, *J* = 7.3 Hz, 1H), 6.94 (d, *J* = 9.0 Hz, 1H), 6.82 (d, *J* = 9.0 Hz, 1H), 5.37 (m, 2H), 3.06 (d, *J* = 7.5 Hz, 6H). **^{13}C NMR (126 MHz, Chloroform-*d*)** δ 158.8, 152.5, 144.4, 140.9, 139.3, 136.3, 133.6, 130.5, 129.2, 128.9, 128.7, 128.4, 128.3, 128.1, 128.1, 127.6, 127.4, 127.4, 127.1, 126.7, 126.6, 125.6, 125.2, 122.7, 122.6, 121.2, 117.0, 111.8, 111.6, 54.8, 54.4, 53.7. **^{11}B NMR (128 MHz, Chloroform-*d*)** δ 40.8. **HRMS (ESI)** calcd for $C_{37}H_{31}BNO_2$ $[M+H]^+$: 532.2442, found: 532.2441. **HPLC analysis**: DAICEL CHIRALCEL IA-3, hexane/isopropanol = 90/10, 1.0 mL/min, λ = 254 nm, t_R (major) = 5.34 min, t_R (minor) = 10.98 min, 97% ee. **$[\alpha]^{25}_D$**: +128.6 (*c* 0.35, $CHCl_3$).

(*R*$_a$,*S*$_a$)-1-benzyl-2,3-bis(2-methoxynaphthalen-1-yl)-6-methyl-1,2-dihydrobenzo[e][1,2]azaborinine (3al)

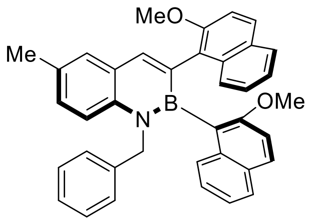

In air, a 25 mL schlenk tube was charged with **1** (0.1 mmol, 1.0 equiv), (2-methoxy-1-naphthyl)boronic acid (0.2 mmol, 2.0 equiv), $Pd_2(dba)_3$ (2 mol%), **L5** (6 mol%) and Li_2CO_3 (0.2 mmol, 2 equiv). The tube was evacuated and filled with argon for three cycles. Then, 1.5 mL of toluene and 150 µL water was added under argon. The reaction was allowed to stir at 40 °C for 58 hours. Upon completion, proper amount of silica gel was added to the reaction mixture. After removal of the solvent, the crude reaction mixture was purified on silica gel (petroleum ether and dichloromethane) to afford the desired products. White solid, mp: 85-87 °C. Yield: 85%. R_f = 0.2 (silica gel, PE:DCM = 3:1).

^{1}H NMR (400 MHz, Acetone-*d*$_6$) δ 7.99 (d, *J* = 8.6 Hz, 1H), 7.90 (s, 1H), 7.77 (d, *J* = 8.4 Hz, 1H), 7.72 – 7.60 (m, 3H), 7.55 (dd, *J* = 9.0, 5.0 Hz, 2H), 7.49 (d, *J* = 8.7 Hz, 1H), 7.40 – 7.32 (m, 2H), 7.30 – 7.22 (m, 4H), 7.22 – 7.14 (m, 3H), 7.09 (t, *J* = 7.3 Hz, 1H), 6.93 (d, *J* = 9.0 Hz, 1H), 6.82 (d, *J* = 9.0 Hz, 1H), 5.33 (m, 2H), 3.05 (d, *J* = 4.0 Hz, 6H), 2.41 (s, 3H). **^{13}C NMR (101 MHz, Acetone-*d*$_6$)** δ 159.4, 153.5, 144.9, 140.2, 139.6, 137.1, 134.3, 131.1, 130.7, 130.0, 130.0, 129.7, 129.6, 129.0, 129.0, 128.7, 128.3, 128.2, 127.9, 127.3, 127.2, 126.2, 125.7, 123.4, 123.4, 1179, 112.8, 112.4, 55.2, 54.9, 53.8, 20.6. **^{11}B NMR (128 MHz, Acetone-*d*$_6$)** δ 38.8. **HRMS (ESI)** calcd for $C_{38}H_{33}BNO_2$ $[M+H]^+$: 546.2599, found: 546.2597. **HPLC analysis**: DAICEL CHIRALCEL IA-3, hexane/isopropanol = 90/10, 1.0 mL/min, λ = 254 nm, t_R (major) = 4.92 min, t_R (minor) = 8.68 min, 95% ee. **$[\alpha]^{25}_D$**: +94.8 (*c* 0.5, $CHCl_3$).

(R_a,S_a)-1-(4-chlorobenzyl)-2,3-bis(2-methoxynaphthalen-1-yl)-1,2-dihydrobenzo[e][1,2]azaborinine (3am)

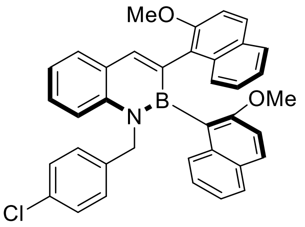

In air, a 25 mL schlenk tube was charged with **1** (0.1 mmol, 1.0 equiv), (2-methoxy-1-naphthyl)boronic acid (0.4 mmol, 4.0 equiv), $Pd_2(dba)_3$ (2 mol%), **L5** (6 mol%) and Li_2CO_3 (0.4 mmol, 4.0 equiv). The tube was evacuated and filled with argon for three cycles. Then, 1.5 mL of toluene and 150 μL water was added under argon. The reaction was allowed to stir at 40 °C for 76 hours. Upon completion, proper amount of silica gel was added to the reaction mixture. After removal of the solvent, the crude reaction mixture was purified on silica gel (petroleum ether and dichloromethane) to afford the desired products. White solid, mp: 80-82°C. Yield: 62%. R_f = 0.2 (silica gel, PE:DCM = 3:1).

^{1}H NMR (600 MHz, Chloroform-*d*) δ 8.02 (s, 1H), 8.00 – 7.94 (m, 1H), 7.75 (td, *J* = 8.2, 1.1 Hz, 2H), 7.68 (dd, *J* = 14.7, 8.1 Hz, 2H), 7.53 (dd, *J* = 8.9, 2.1 Hz, 2H), 7.46 (d, *J* = 8.5 Hz, 1H), 7.44 – 7.30 (m, 3H), 7.28 – 7.22 (m, 3H), 7.15 (s, 4H), 6.78 (d, *J* = 9.0 Hz, 1H), 6.67 (d, *J* = 9.0 Hz, 1H), 5.39 (d, *J* = 16.3 Hz, 1H), 5.19 (d, *J* = 16.2 Hz, 1H), 2.79 (d, *J* = 7.7 Hz, 6H). **^{13}C NMR (101 MHz, Chloroform-*d*)** δ 158.8, 152.5, 144.5, 140.6, 137.8, 136.2, 133.5, 132.3, 130.7, 129.3, 128.9, 128.7, 128.6, 128.1, 128.1, 127.7, 127.4, 127.2, 127.2, 126.5, 125.7, 125.2, 122.7, 122.7, 121.4, 116.8, 111.7, 111.6, 54.8, 54.4, 53.0. **^{11}B NMR (128 MHz, Chloroform-*d*)** δ 41.2. **HRMS (ESI)** calcd for $C_{37}H_{30}BClNO_2$ $[M+H]^+$: 566.2053, found: 566.2056. **HPLC analysis**: DAICEL CHIRALCEL IA-3, hexane/isopropanol = 90/10, 1.0 mL/min, λ = 254 nm, t_R (major) = 5.81 min, t_R (minor) = 11.62 min, 97% ee. **$[\alpha]^{25}_D$**: +97.2 (*c* 0.5, $CHCl_3$).

(R_a,S_a)-1-benzyl-2-(2-methoxy-6-methylnaphthalen-1-yl)-3-(2-methoxynaphthalen-1-yl)-1,2-dihydrobenzo[e][1,2]azaborinine (3an)

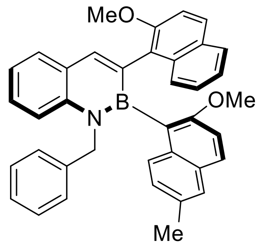

In air, a 25 mL schlenk tube was charged with **1** (0.1 mmol, 1.0 equiv), (2-methoxy-1-naphthyl)boronic acid (0.4 mmol, 4.0 equiv), $Pd_2(dba)_3$ (2 mol%), **L5** (6 mol%) and Li_2CO_3 (0.4 mmol, 4.0 equiv). The tube was evacuated and filled with argon for three cycles. Then, 1.5 mL of toluene and 150 μL water was added under argon. The reaction was allowed to stir at 40 °C for 76 hours. Upon completion, proper amount of silica gel was added to the reaction mixture. After removal of the solvent, the crude reaction mixture was purified on silica gel (petroleum ether and dichloromethane) to afford the desired products. White solid, mp: 64-66°C. Yield: 61%. R_f = 0.2 (silica gel, PE:DCM = 3:1).

^{1}H NMR (600 MHz, Acetone-d_6) δ 7.97 (d, *J* = 10.0 Hz, 2H), 7.83 (dd, *J* = 7.7, 1.6 Hz, 1H), 7.71 – 7.66 (m, 2H), 7.61 (d, *J* = 8.6 Hz, 1H), 7.56 (d, *J* = 9.0 Hz, 1H), 7.45 (d, *J* = 8.9 Hz, 1H), 7.43 – 7.39 (m, 2H), 7.39 – 7.33 (m, 1H), 7.30 – 7.26 (m, 3H), 7.26 – 7.21 (m, 2H), 7.20 – 7.14 (m, 2H), 7.10 (t, *J* = 7.4 Hz, 1H), 6.95 (d, *J* = 9.0 Hz, 1H), 6.78 (d, *J* = 9.0 Hz, 1H), δ 5.35 (d, *J* = 16.3 Hz, 1H), 5.31 (d, *J* = 16.3 Hz, 1H), 3.08 (d, *J* = 19.3 Hz, 6H), 2.39 (s, 3H). **^{13}C NMR (126 MHz, Acetone-d_6)** δ 158.9, 153.5, 145.1, 141.6, 140.1, 135.3,

134.3, 132.5, 131.0, 129.8, 129.7, 129.4, 129.1, 128.9, 128.8, 128.5, 128.3, 128.3, 128.2, 128.0, 127.7, 127.4, 127.3, 127.2, 125.7, 123.4, 122.0, 118.0, 112.9, 112.4, 55.3, 54.9, 53.8, 21.4. **^{11}B NMR (128 MHz, Chloroform-*d*)** δ 41. 8. **HRMS (ESI)** calcd for $C_{38}H_{33}BNO_2$ $[M+H]^+$: 546.2599, found: 546.2605. **HPLC analysis**: DAICEL CHIRALCEL IA-3, hexane/isopropanol = 98/2, 1.0 mL/min, λ = 254 nm, t_R (major) = 8.12 min, t_R (minor) = 46.11 min, 96% ee. **[α]$^{25}_{D}$**: +66.0 (*c* 0.5, $CHCl_3$).

2.6 Synthetic transformations

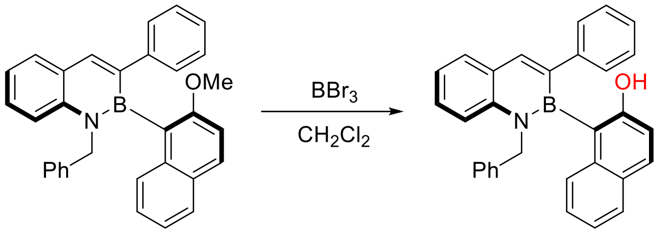

3m **5**

In air, a 10 mL schlenk tube was charged with **3m** (0.1 mmol, 1 equiv). The tube was evacuated and filled with argon for three cycles. Then, 1 mL of dry DCM was added under argon. The reaction was allowed to stir at 0 °C for 20 min. Then, BBr_3 (2.0 M in dichloromethane, 0.20 mL, 0.40 mmol) was added to the cooled tube. The reaction mixture was continued to stir at 0 °C for 4 hours. At 0 °C, the reaction was quenched by water, and the mixture was extracted with DCM and dried over Na_2SO_4. After removal of the solvent, the crude reaction mixture was purified on column chromatography (petroleum ether and ethyl acetate) to afford the desired product **5**.

(*R$_a$*)-1-(1-benzyl-3-phenylbenzo[e][1,2]azaborinin-2(1H)-yl)naphthalen-2-ol (5)

White solid, mp: 78 – 80 °C. Yield: 50%. R_f = 0.5 (silica gel, PE:EtOAc = 3:1).

^{1}H NMR (500 MHz, Chloroform-*d*) δ 8.20 (s, 1H), 7.85 (dd, *J* = 7.8, 1.5 Hz, 1H), 7.78 – 7.74 (m, 1H), 7.66 (d, *J* = 8.8 Hz, 1H), 7.55 – 7.48 (m, 2H), 7.44 – 7.38 (m, 1H), 7.33 – 7.27 (m, 3H), 7.23 – 7.16 (m, 4H), 7.15 – 7.10 (m, 3H), 7.09 – 7.05 (m, 3H), 6.84 (d, *J* = 8.7 Hz, 1H), 5.39 (d, *J* = 16.4 Hz, 1H), 5.28 (d, *J* = 16.4 Hz, 1H), 4.68 (s, 1H). **^{13}C NMR (126 MHz, Chloroform-*d*)** δ 154.7, 144.2, 143.6, 141.0, 138.8, 136.6, 130.8, 130.1, 129.1, 128.6, 128.6, 128.5, 128.0, 127.9, 127.4, 127.3, 126.8, 126.4, 126.3, 126.1, 123.7, 123.2, 121.8, 117.8, 117.3, 109.6, 53.4. **^{11}B NMR (128 MHz, Chloroform-*d*)** δ 39.8. **HRMS (ESI)** calcd for $C_{31}H_{25}BNO$ $[M+H]^+$: 438.2024, found: 438.2018. **HPLC analysis**: DAICEL CHIRALCEL IA-3, hexane/isopropanol = 90/10, 1.0 mL/min, λ = 254 nm, t_R (major) = 15.54 min, t_R (minor) = 20.70 min, 92% ee. **[α]$^{25}_{D}$**: +21.6 (*c* 0.5, $CHCl_3$).

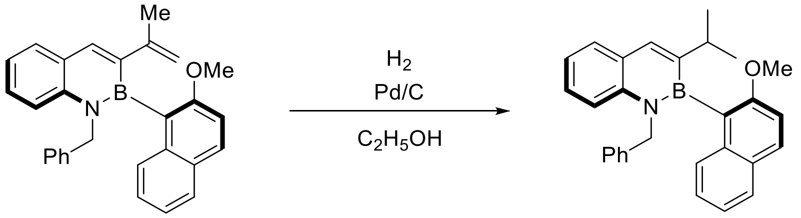

3u **6**

In air, a 25 mL schlenk tube was charged with **3u** (0.1 mmol, 1 equiv) and Pd/C (50 mol%). The tube was evacuated and filled with argon for three cycles, followed by evacuated and filled with a H_2 balloon. Then, 1 mL of MeOH was added under argon. The reaction was allowed to stir at room temperature for 12 hours. Upon completion, the reaction mixture was filtered through celite and washed with ethyl acetate. After removal of the solvent, the crude reaction mixture was purified on silica gel (petroleum ether and dicloromethane) to afford the

desired product **6**.

(R_a)-1-benzyl-3-isopropyl-2-(2-methoxynaphthalen-1-yl)-1,2-dihydrobenzo[e][1,2]azaborinine (6)

White solid, mp: 144-146 °C. Yield: 91%. R_f = 0.5 (silica gel, PE:DCM = 5:1).

^{1}H NMR (400 MHz, Chloroform-*d*) δ 7.98 (s, 1H), 7.85 (dd, *J* = 14.3, 9.1 Hz, 2H), 7.76 (dd, *J* = 7.7, 1.5 Hz, 1H), 7.47 – 7.40 (m, 1H), 7.38 (d, *J* = 8.5 Hz, 1H), 7.34 – 7.27 (m, 3H), 7.27 – 7.22 (m, 2H), 7.22 – 7.15 (m, 2H), 7.11 (t, *J* = 7.5 Hz, 3H), 5.20 (d, *J* = 16.5 Hz, 1H), 5.14 (d, *J* = 16.5 Hz, 1H), 3.69 (s, 3H), 2.68 – 2.53 (m, 1H), 1.11 (d, *J* = 6.8 Hz, 3H), 1.02 (d, *J* = 6.8 Hz, 3H). **^{13}C NMR (101 MHz, Chloroform-*d*)** δ 158.6, 140.5, 139.3, 137.7, 136.5, 129.9, 129.6, 129.0, 128.3, 128.3, 127.7, 127.4, 127.3, 126.4, 126.0, 123.2, 121.0, 116.9, 112.5, 55.4, 53.3, 31.7, 24.0, 23.6. **^{11}B NMR (128 MHz, Chloroform-*d*)** δ 39.8. **HRMS (ESI)** calcd for $C_{33}H_{31}BNO$ $[M+H]^+$: 418.2337, found: 418.2329. **HPLC analysis**: DAICEL CHIRALCEL OD-H, hexane/isopropanol = 99.5/0.5, 0.5 mL/min, λ = 254 nm, t_R (minor) = 9.46 min, t_R (major) = 10.82 min, 89% ee. **$[\alpha]^{25}_D$**: +17.1 (*c* 0.5, $CHCl_3$).

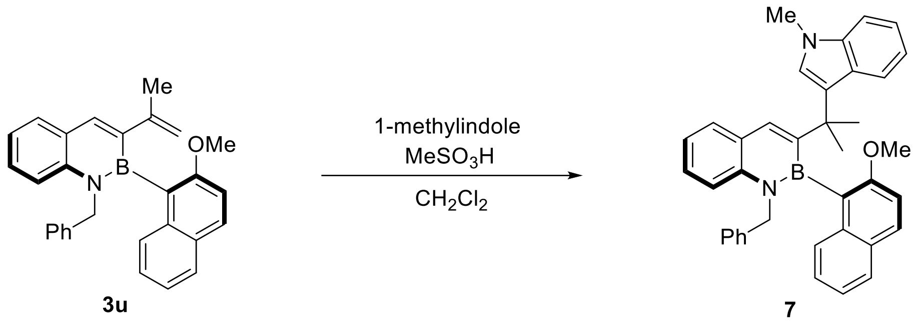

3u **7**

In air, a 10 mL schlenk tube was charged with **3u** (0.1 mmol, 1 equiv) and 1-methylindole (0.15 mmol, 1.5 equiv). The tube was evacuated and filled with argon for three cycles. Then at 0°C, 2 mL of DCM and methanesulfonic acid (10 mol%) were added under argon. The reaction was allowed to stir at room temperature for 6 hours. Upon completion, proper amount of silica gel was added to the reaction mixture. After removal of the solvent, the crude reaction mixture was purified on silica gel (petroleum ether and ethyl acetate) to afford the desired product **7**.

(R_a)-1-benzyl-2-(2-methoxynaphthalen-1-yl)-3-(2-(1-methyl-1H-indol-2-yl)propan-2-yl)-1,2-dihydrobenzo[e][1,2]azaborinine (7)

Coloeless oil. Yield: 37%. R_f = 0.2 (silica gel, PE:DCM = 5:1).

^{1}H NMR (400 MHz, Chloroform-*d*) δ 8.35 (s, 1H), 7.85 (dd, *J* = 7.5, 1.5 Hz, 1H), 7.72 (d, *J* = 8.9 Hz, 1H), 7.63 (d, *J* = 8.1 Hz, 1H), 7.45 (d, *J* = 7.9 Hz, 1H), 7.34 – 7.24 (m, 3H), 7.19 – 6.96 (m, 8H), 6.93 – 6.84 (m, 2H), 6.51 – 6.42 (m, 1H), 6.37 (d, *J* = 8.4 Hz, 1H), 5.75 (s, 1H), 5.00 (d, *J* = 16.8 Hz, 1H), 4.91 (d, *J* = 16.8 Hz, 1H), 3.36 (s, 3H), 3.16 (s, 3H), 1.73 (s, 3H), 1.62 (s, 3H). **^{13}C NMR (101 MHz, Chloroform-*d*)** δ 157.7, 140.6, 139.4, 138.7, 137.4, 136.1, 130.4, 128.5, 128.2, 128.1, 127.5, 127.2, 127.1, 127.0, 127.0, 126.4, 126.1, 124.5, 124.1, 123.7, 122.5, 121.3, 120.9, 120.4, 117.8, 116.9, 111.4, 108.8, 54.3, 52.9, 39.7, 31.8, 30.5, 23.0. **^{11}B NMR (128 MHz, Chloroform-*d*)** δ 41.2. **HRMS (ESI)** calcd for $C_{38}H_{36}BN_2O$ $[M+H]^+$: 547.2915, found: 547.2914. **HPLC analysis**: DAICEL CHIRALCEL AD-H, hexane/isopropanol = 99/1, 1.0mL/min, λ = 254 nm, t_R (minor) = 6.18 min, t_R (major) = 7.32 min, 92% ee. **$[\alpha]^{25}_D$**: +180.2 (*c* 0.5, $CHCl_3$).

2.7 Mechanism experiments

(1) Yields and ee values of product 3a (recovered 1a) at different reaction times

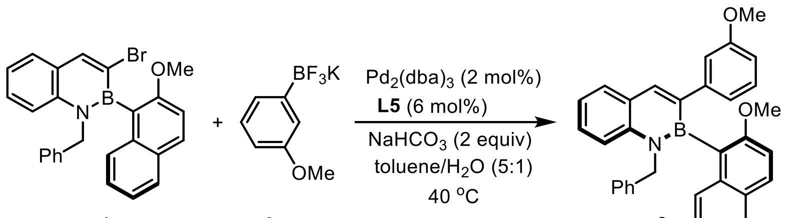

In air, a 25 mL schlenk tube was charged with **1a** (0.2 mmol, 1 equiv), **2a** (0.26 mmol, 1.3 equiv), $Pd_2(dba)_3$ (2 mol%), **L5** (6 mol%), $NaHCO_3$ (0.4 mmol, 2.0 equiv) and pyrene (0.2 mmol, 1 equiv, as internal standard for yield). The tube was evacuated and filled with argon for three cycles. Then, 3.0 mL of toluene and 0.6 ml water was added under argon. The reaction was allowed to stir at 40 °C. An aliquot of reaction mixture (40 μl) was taken out 1, 2, 4, 6, 8, 10, 13 and 16 h. The sample was analyzed by HPLC.

Supplementary Table 1. Yields and ee values of product 3a (recovered 1a) at different reaction times.

t (h)	yield of recovered **1a** (%)	ee of recovered **1a** (%)	yield of **3a** (%)	ee of **3a** (%)
0	100	0	0	--
1	85.5	10	11.4	96
2	71.0	17	26	96
4	43.0	29	52	96
6	26.5	48	69.8	96
8	14.6	78	84.3	96
10	7.9	88	88.6	96
13	2.9	98	92.3	96
16	1.2	99	96.5	96

(2) Ee values of recovered 1a at different reaction times by using ligands L5 and (-)-L5

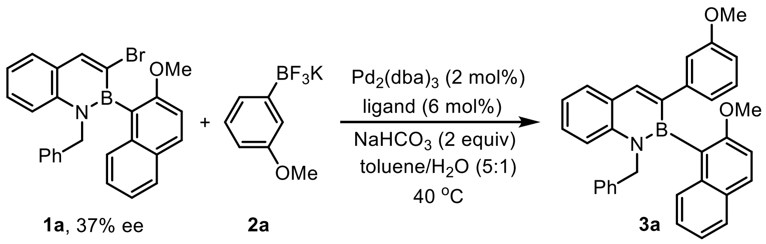

In air, a 25 mL schlenk tube was charged with enantiopure **1a** (37% ee) (0.1 mmol, 1 equiv), **2a** (0.13 mmol, 1.3 equiv), $Pd_2(dba)_3$ (2 mol%), **L5** or **(-)-L5** (6 mol%) and $NaHCO_3$ (0.2 mmol, 2.0 equiv). The tube was evacuated and filled with argon for three cycles. Then, 1.5 mL of toluene and 0.3 ml water was added under argon. The reaction was allowed to stir at 40 °C. An aliquot of reaction mixture (25 μl) was taken out at different times. The sample was analyzed by HPLC.

Supplementary Table 2. Ee values of recovered 1a at different reaction times by using ligands L5 and (-)-L5.

t (h)	ee of recovered **1a** (%) with ligand **L5**	t (min)	ee of recovered **1a** (%) with ligand **(-)-L5**
0	37	0	37
1	51	20	37
2	60	40	33
3	67	60	31
4	78	80	27
5	88	100	25
6	93	120	20
7	>99	150	15
8	>99	180	9
9	>99	210	0
10	>99	240	-12
12	>99	270	-22
		300	-34
		330	-43
		360	-54
		390	-69
		420	-88
		450	-99

(3) Racemization experiment under standard conditions without aryl trifluoroborates

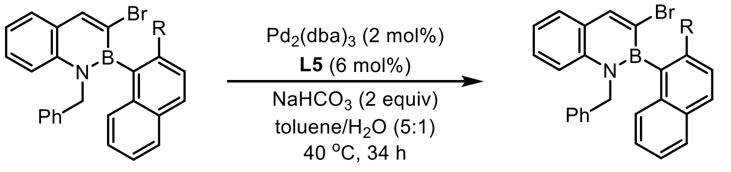

1a, 35% ee　　　　**1a**, 33% ee

In air, a 25 mL schlenk tube was charged with enantiopure **1a** (35% ee) (0.05 mmol, 1 equiv), $Pd_2(dba)_3$ (2 mol%), **L5** (6 mol%) and $NaHCO_3$ (0.1 mmol, 2.0 equiv). The tube was evacuated and filled with argon for three cycles. Then, 0.75 mL of toluene and 0.15 ml water was added under argon. The reaction was allowed to stir at 40 °C for 34 hours. The sample was analyzed by HPLC.

2.8 Rotational barriers of C-B axially chiral compound 3a

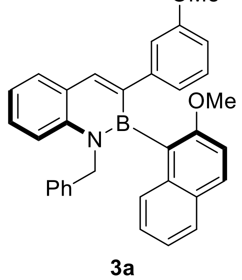

3a

A solution of **(R_a)-3a** (5.0 mg) in mesitylene (1 mL) was heated at the specific temperatures. The ee value was

determined by chiral HPLC analysis at different intervals.

Supplementary Table 3. Rotational Barrier of (*R$_a$*)-3a at 140 ºC

t(s)	ee (%)	Ln(ee_0/ee_t)
0	96.4	0
3600	94.3	0.022025
7200	90.6	0.062052
14400	82.4	0.156921
21600	73.2	0.275311
28800	67.8	0.351944
43200	52.5	0.607693

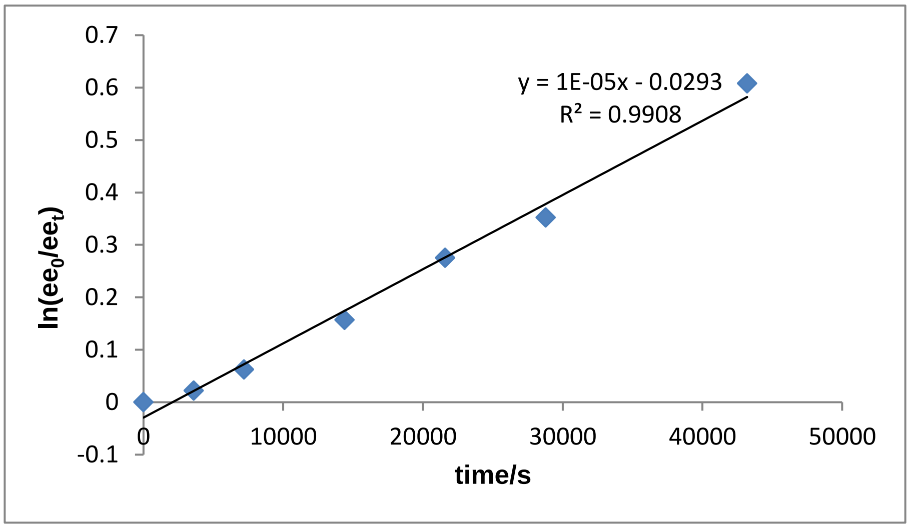

Supplementary Figure 5. The plot of ln(ee_0/ee_t) vs time of (*R$_a$*)-3a at 140 ºC

$k_{racemization}$ (140 ºC) = 1 x 10^{-5} s^{-1}

$k_{enantiomerization}$ (140 ºC) = 0.5 x 10^{-5} s^{-1}

$\Delta G^{\neq}_{enantiomerization}$ = 34.46 kcal/mol

2.9 Crystal structure of compound 3a (CCDC 2245394)

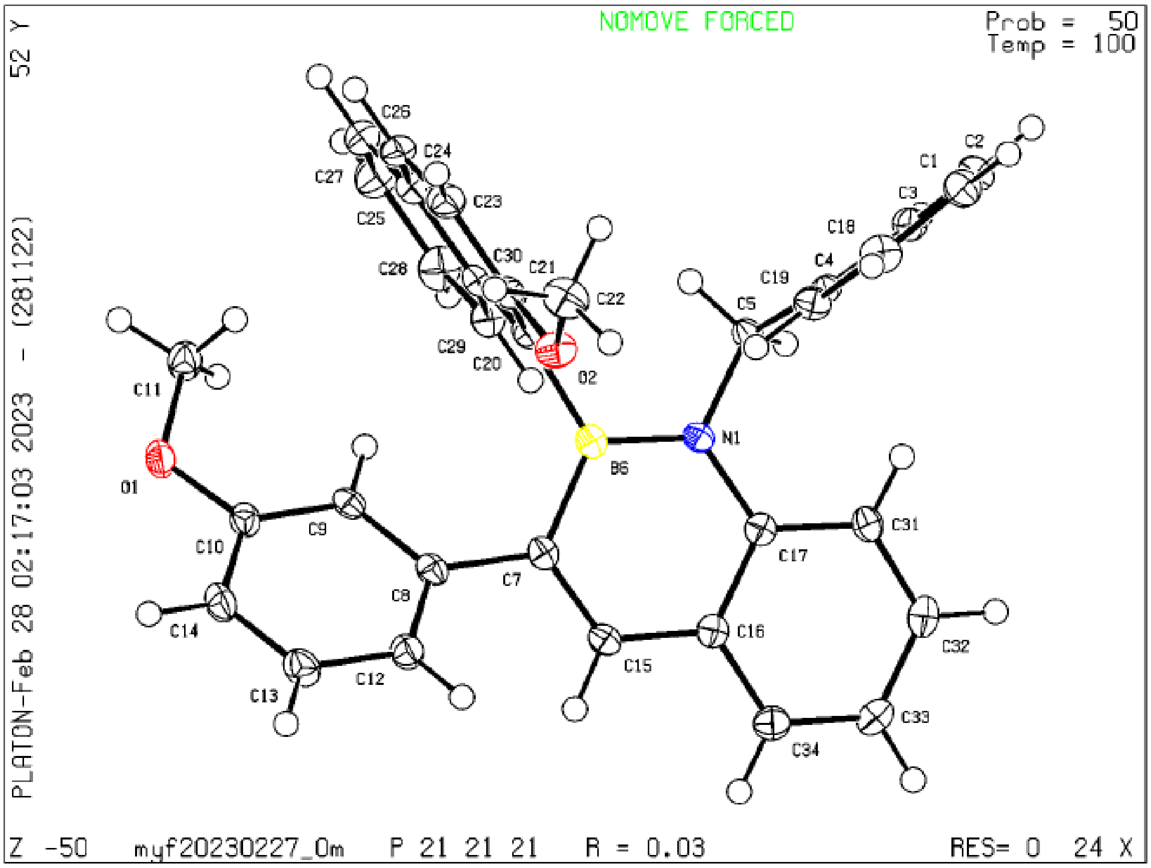

Bond precision:	C-C = 0.0020 A	Wavelength=1.54178	
Cell:	a=8.0196(2)	b=16.1210(4)	c=19.4992(4)
	alpha=90	beta=90	gamma=90
Temperature: 100 K			

	Calculated	Reported
Volume	2520.93(10)	2520.93(10)
Space group	P 21 21 21	P 21 21 21
Hall group	P 2ac 2ab	P 2ac 2ab
Moiety formula	C33 H28 B N O2	C33 H28 B N O2
Sum formula	C33 H28 B N O2	C33 H28 B N O2
Mr	481.37	481.37
Dx,g cm-3	1.268	1.268
Z	4	4
Mu (mm-1)	0.604	0.604
F000	1016.0	1016.0
F000’	1018.80	
h,k,lmax	9,19,23	9,19,23
Nref	4636[2647]	4631
Tmin,Tmax	0.840,0.881	0.581,0.753
Tmin’ 0.795		

Correction method= # Reported T Limits: Tmin=0.581 Tmax=0.753

AbsCorr = MULTI-SCAN

Data completeness= 1.75/1.00 Theta(max)= 68.309

R(reflections)= 0.0254(4597)

wR2(reflections)= 0.0652(4631)

S = 1.060 Npar= 336

Flack parameter 0.06(3)

2.10 Two-dimensional NMR analysis of 3ai

Conclusion: two OMe groups are ***opposite side***, referring to the borazaronaphthalene ring.

Analysis: 1) The single aromatic peak was attributed as 17-CH; 2) No matter the cis or trans, the NOE correlation with 17-CH is only with 9-CH3; 3) The aromatic double-peak hydrogens that have possible correlations with two OMe groups include 6-CH, 10-CH, 7-CH and 14-CH; 4) The HMBC experiment made the 6-CH and 10-CH confirmed (Figure S2, S3 and S4); 5) between 6-CH and 10-CH, the only one has correlation with benzylic H (C1-H) should be 7-CH, thus confirming the 7-CH and 14-CH; 6) The correlation between 7-CH and 9-CH3 , as well as the correlation between 14-CH and 5-CH3 indicated the two OMe groups are ***opposite side***, referring to the borazaronaphthalene ring.

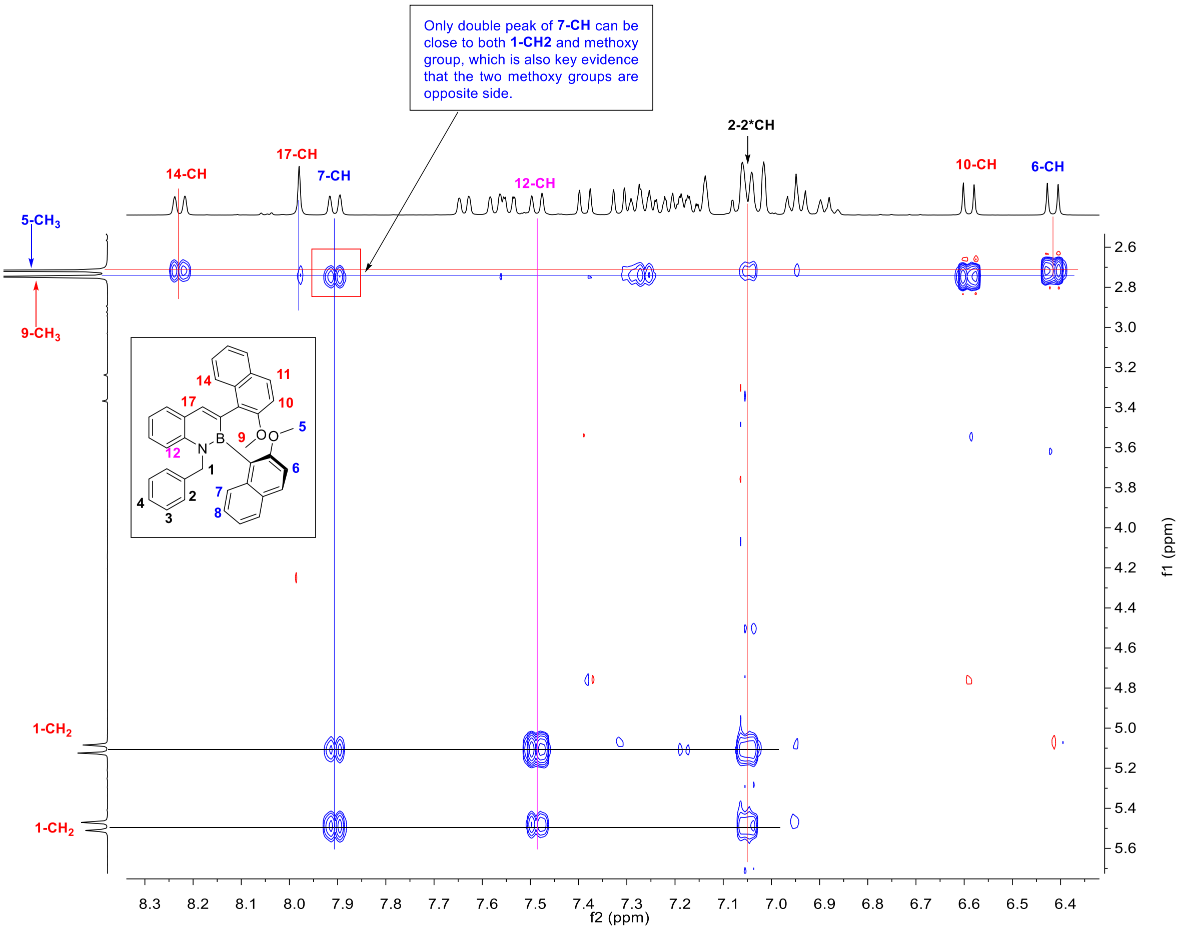

Supplementary Figure 6. NOE spectrum-1 of 3ai in toluene-*d*$_8$.

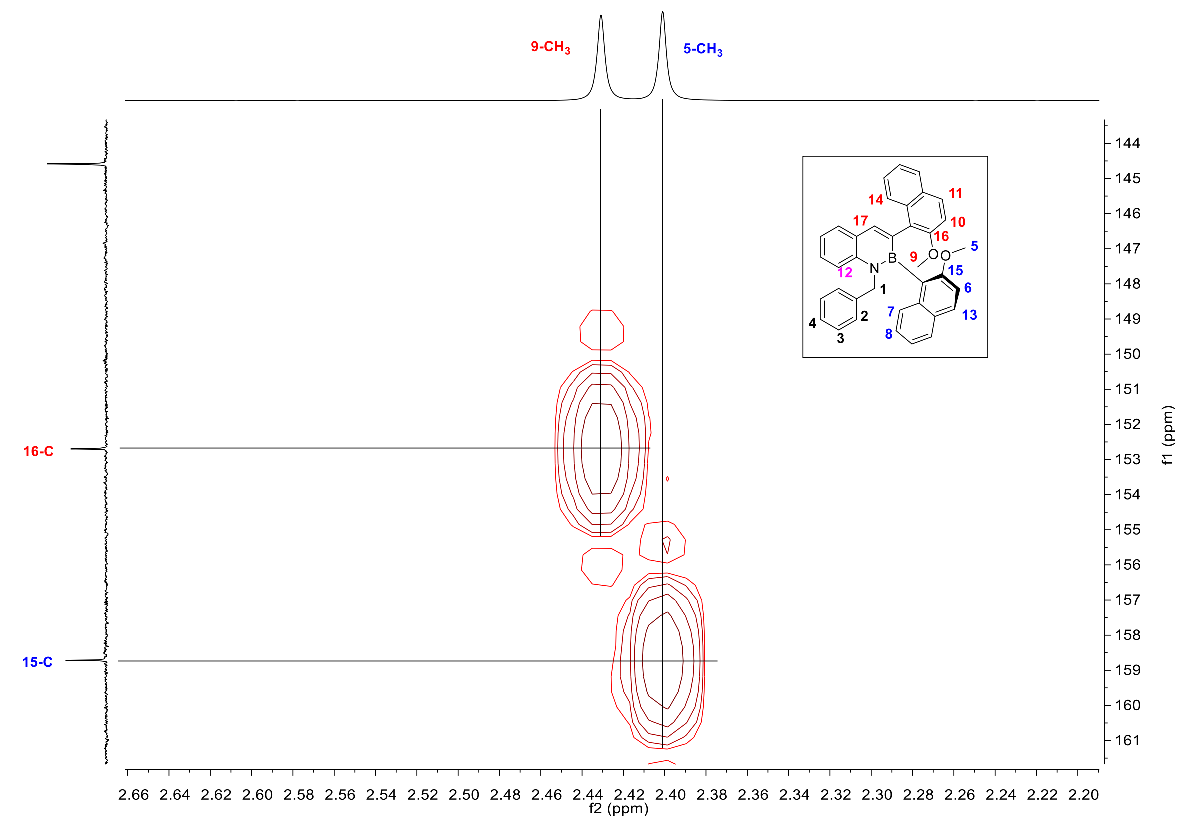

Supplementary Figure 7. HMBC spectrum-1 of 3ai in toluene-*d*8.

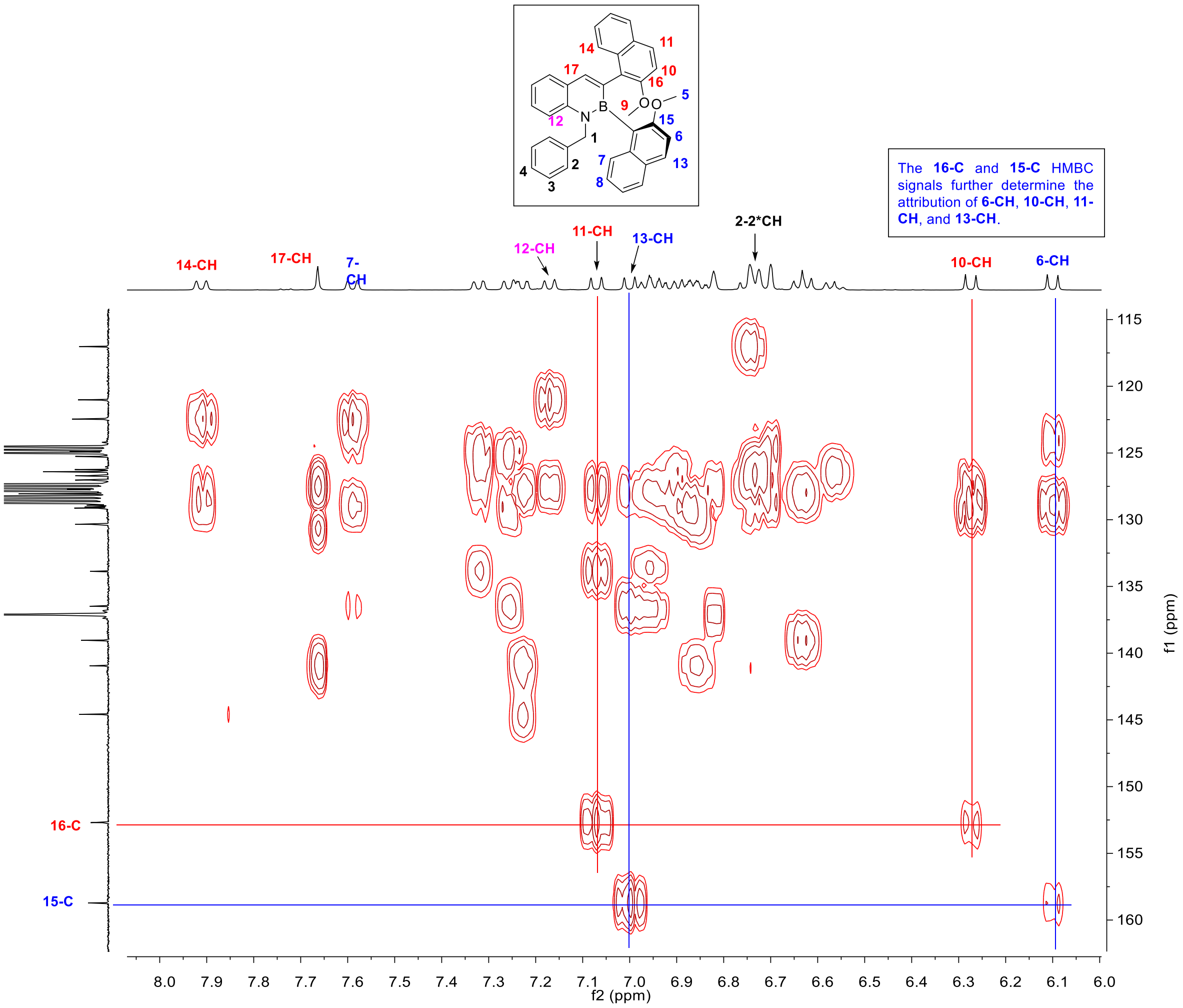

Supplementary Figure 8. HMBC spectrum-2 of **3ai** in toluene-d_8.

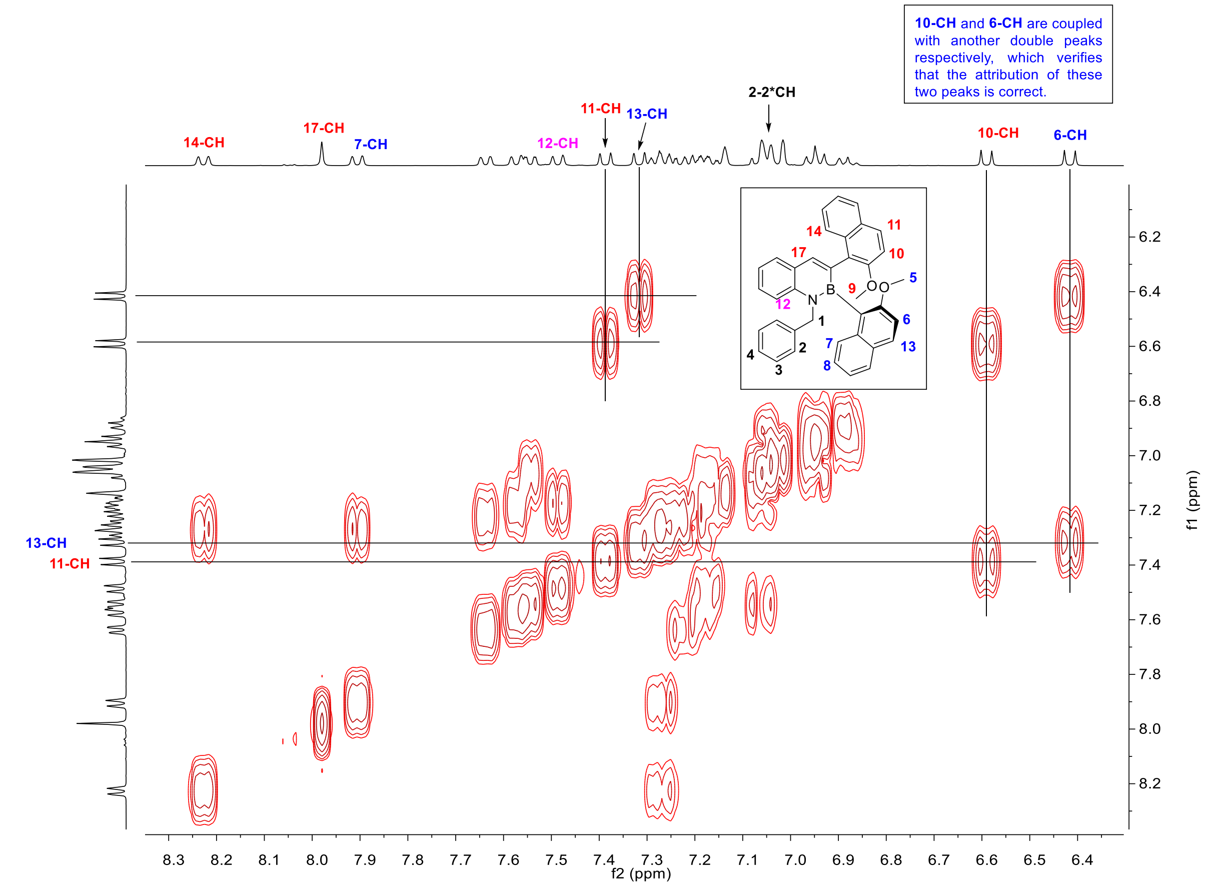

Supplementary Figure 9. NOE spectrum-2 of 3ai in toluene-d_8.

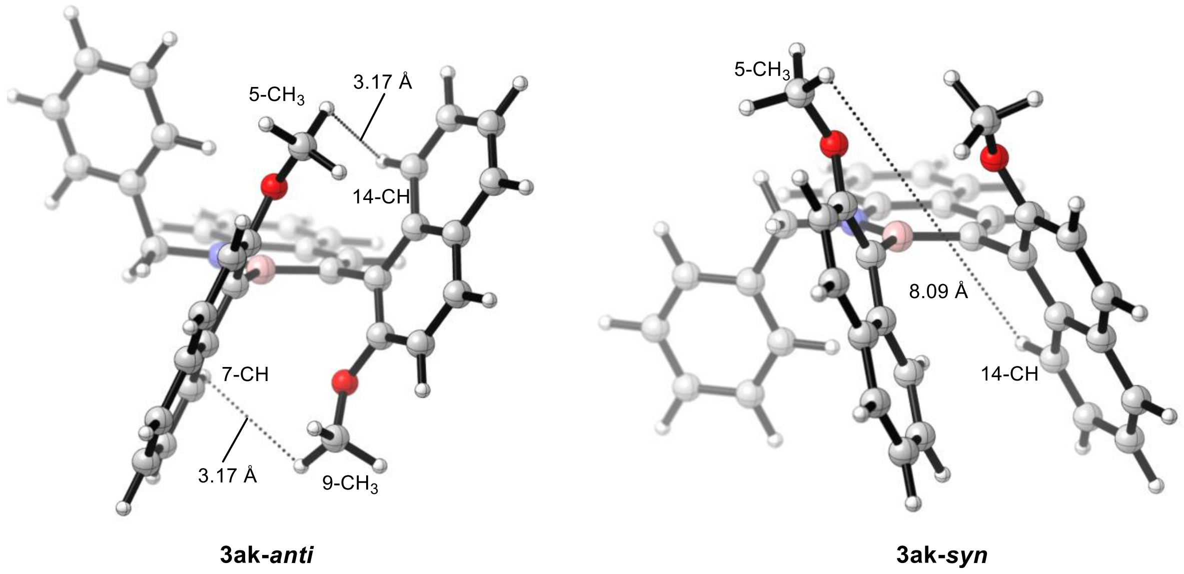

Supplementary Figure 10. 3D geometries of optimized structures of 3ak-*anti* and 3ak-*syn*.

3 NMR Spectra

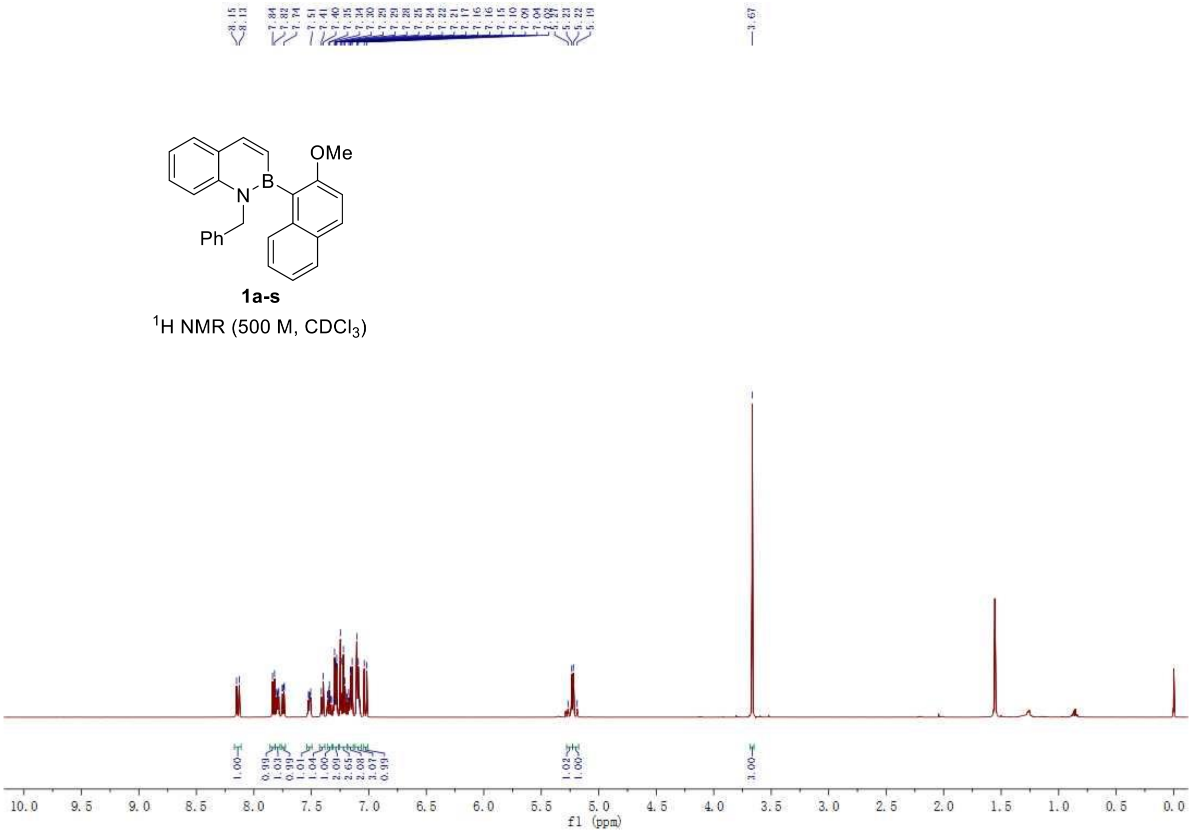

Supplementary Figure 11. ^{1}H NMR spectrum of 1a-s

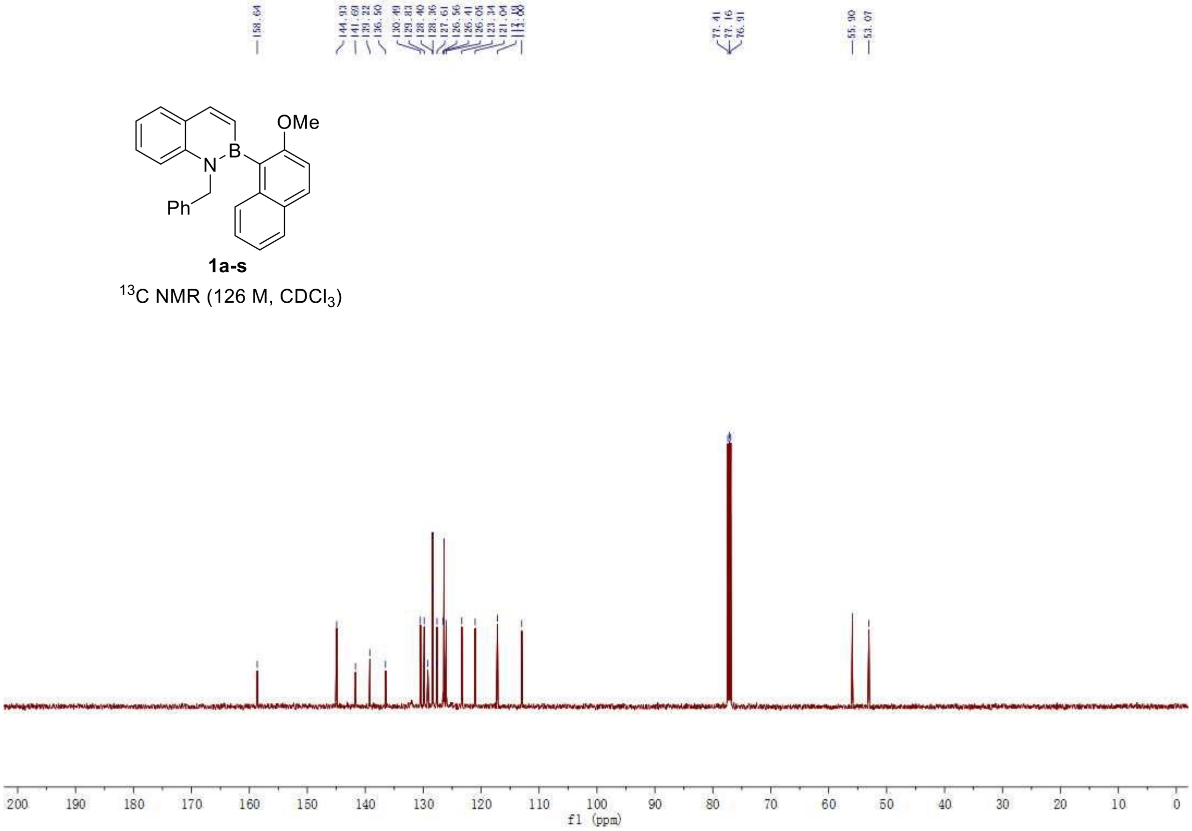

Supplementary Figure 12. ^{13}C NMR spectrum of 1a-s

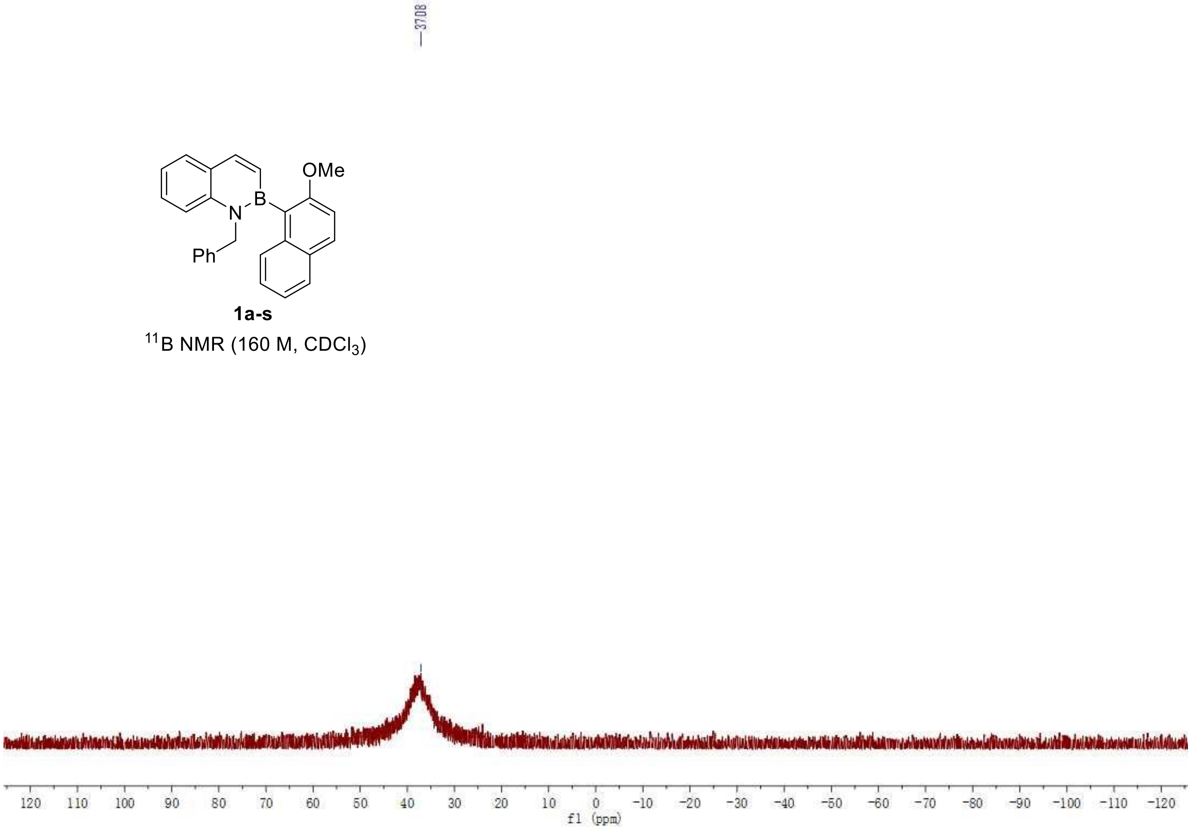

Supplementary Figure 13. ^{11}B NMR spectrum of 1a-s

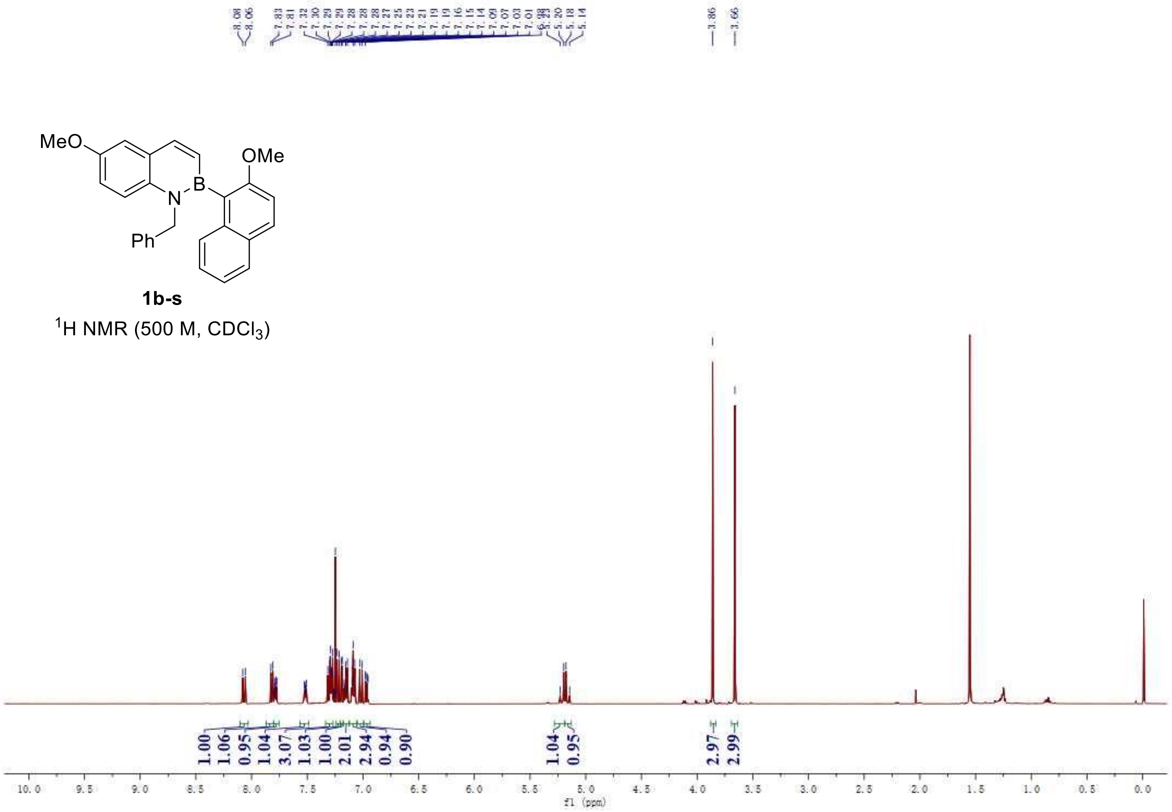

Supplementary Figure 14. ^{1}H NMR spectrum of 1b-s

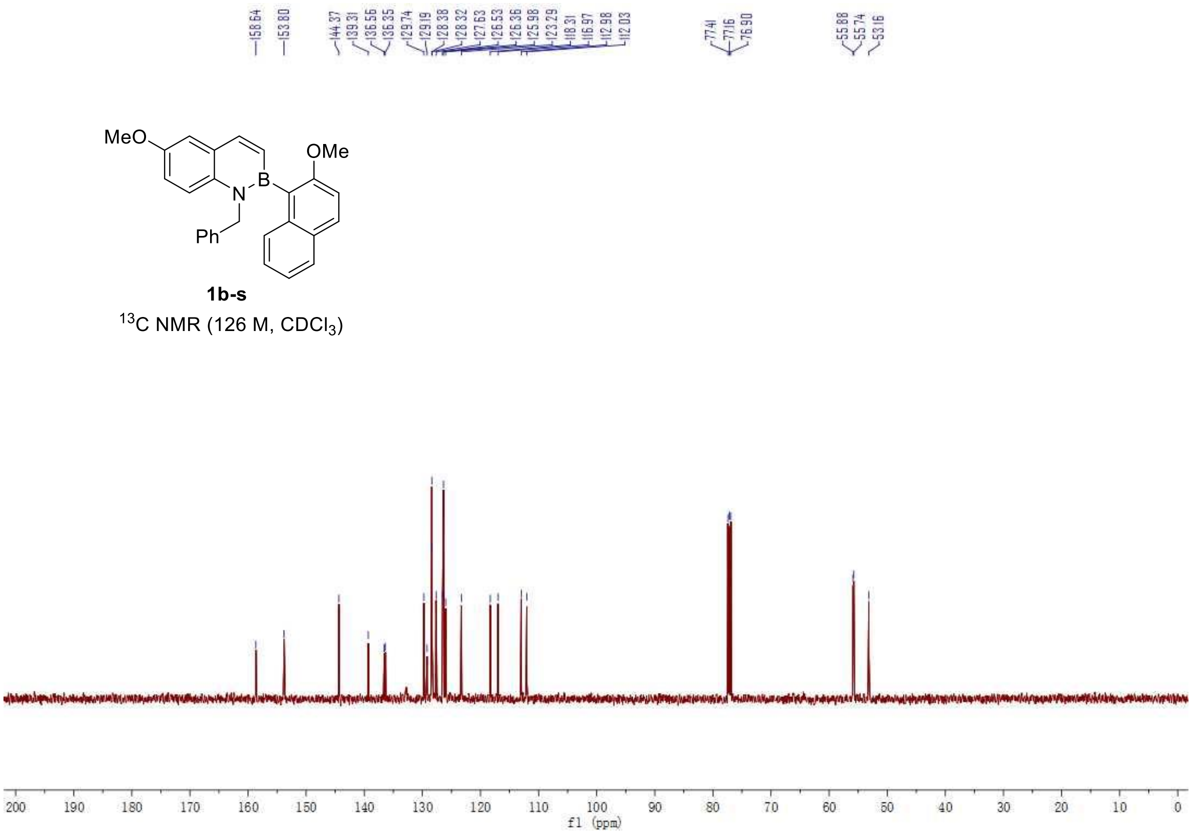

Supplementary Figure 15. ^{13}C NMR spectrum of 1b-s

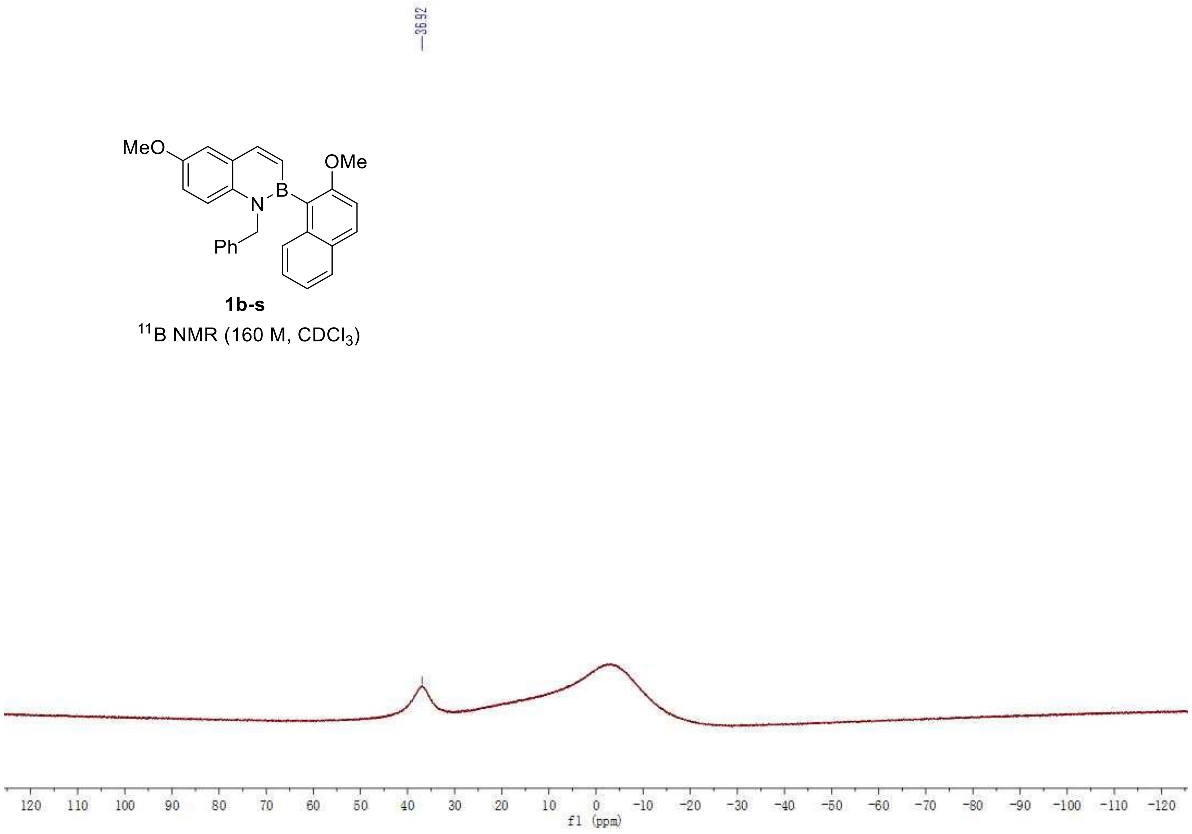

Supplementary Figure 16. ^{11}B NMR spectrum of 1b-s

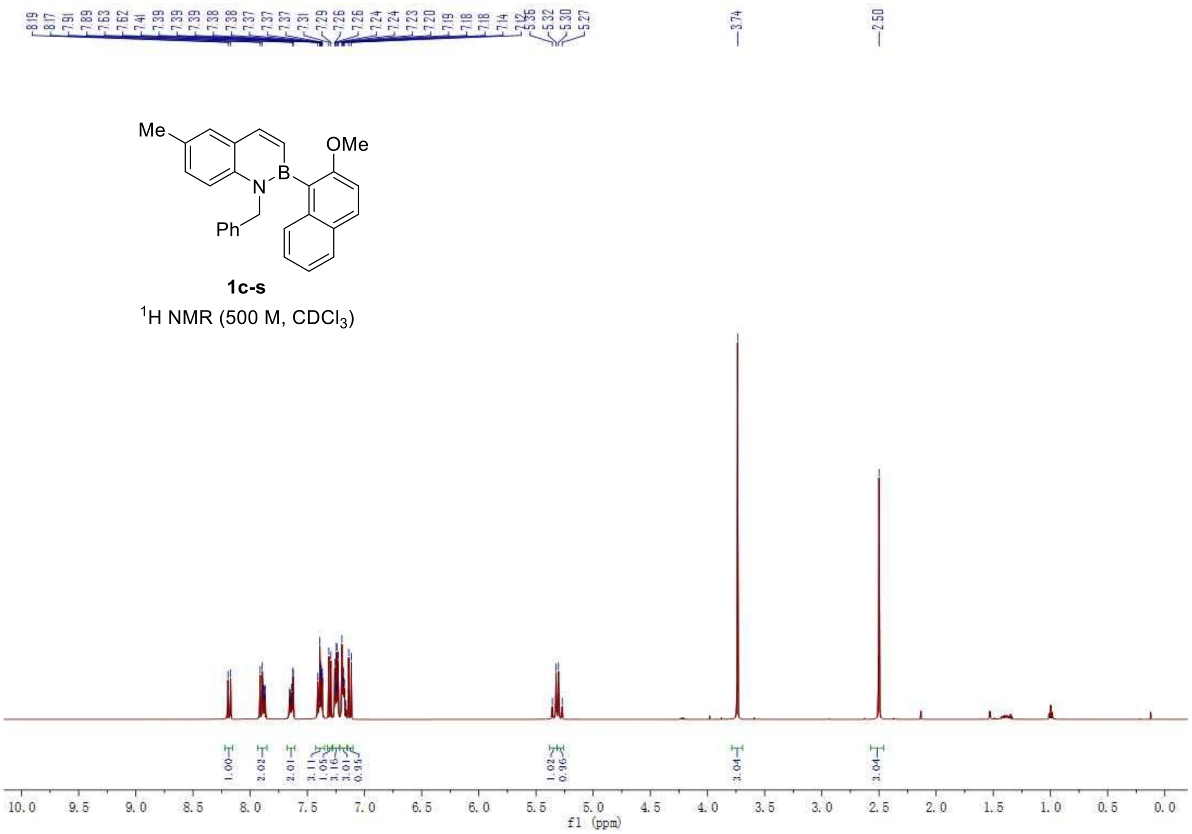

Supplementary Figure 17. ^{1}H NMR spectrum of 1c-s

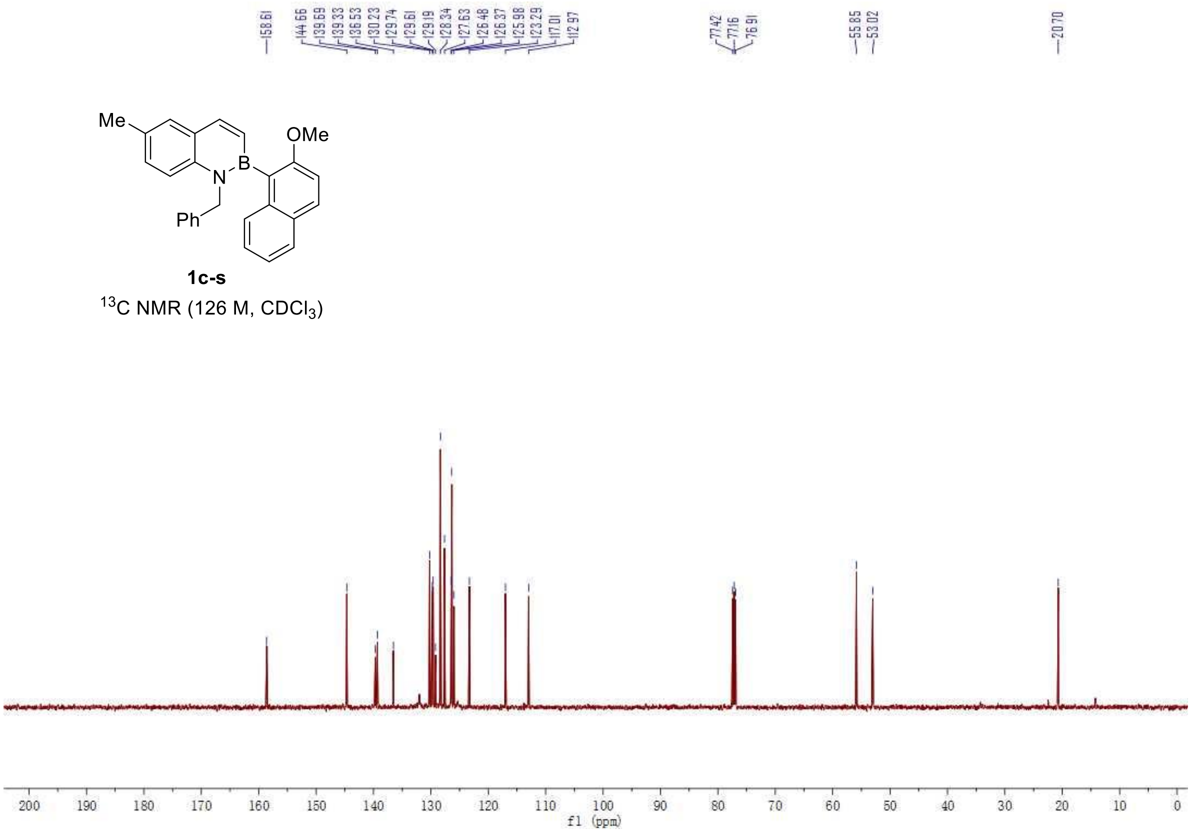

Supplementary Figure 18. ^{13}C NMR spectrum of 1c-s

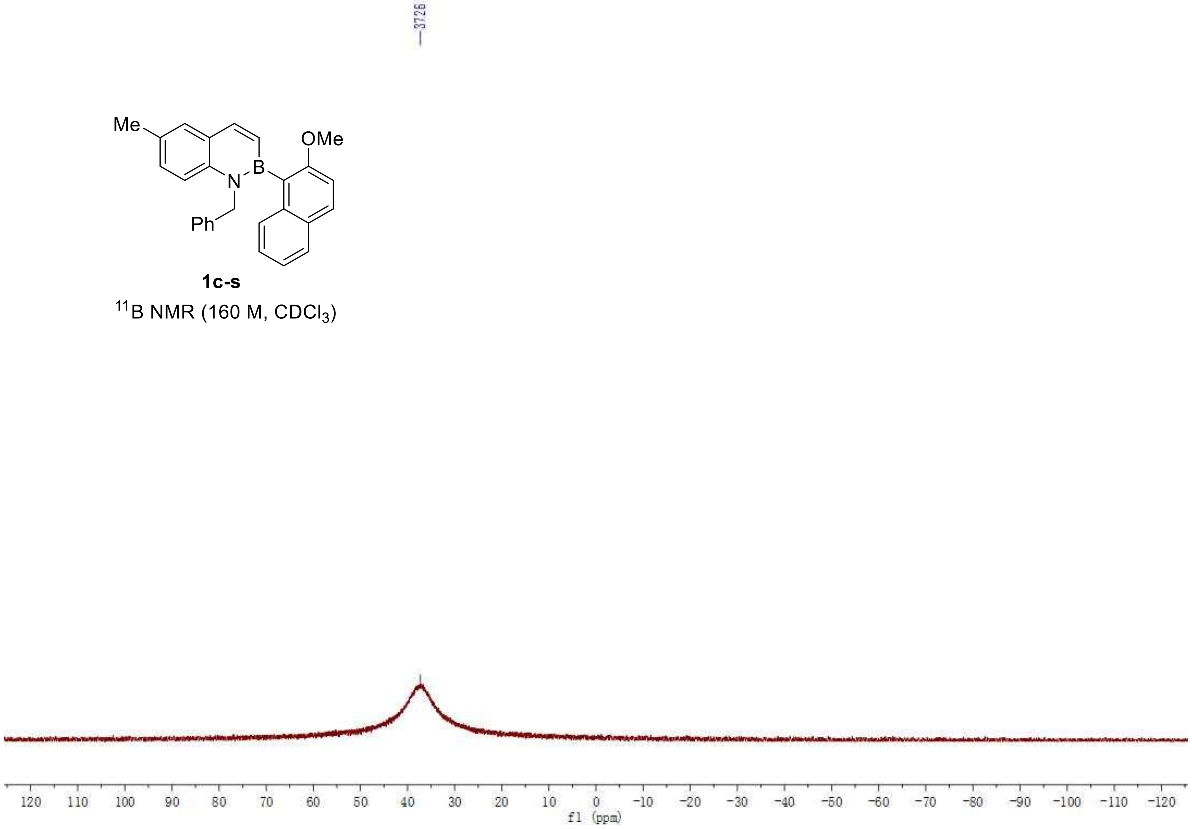

Supplementary Figure 19. ^{11}B NMR spectrum of 1c-s

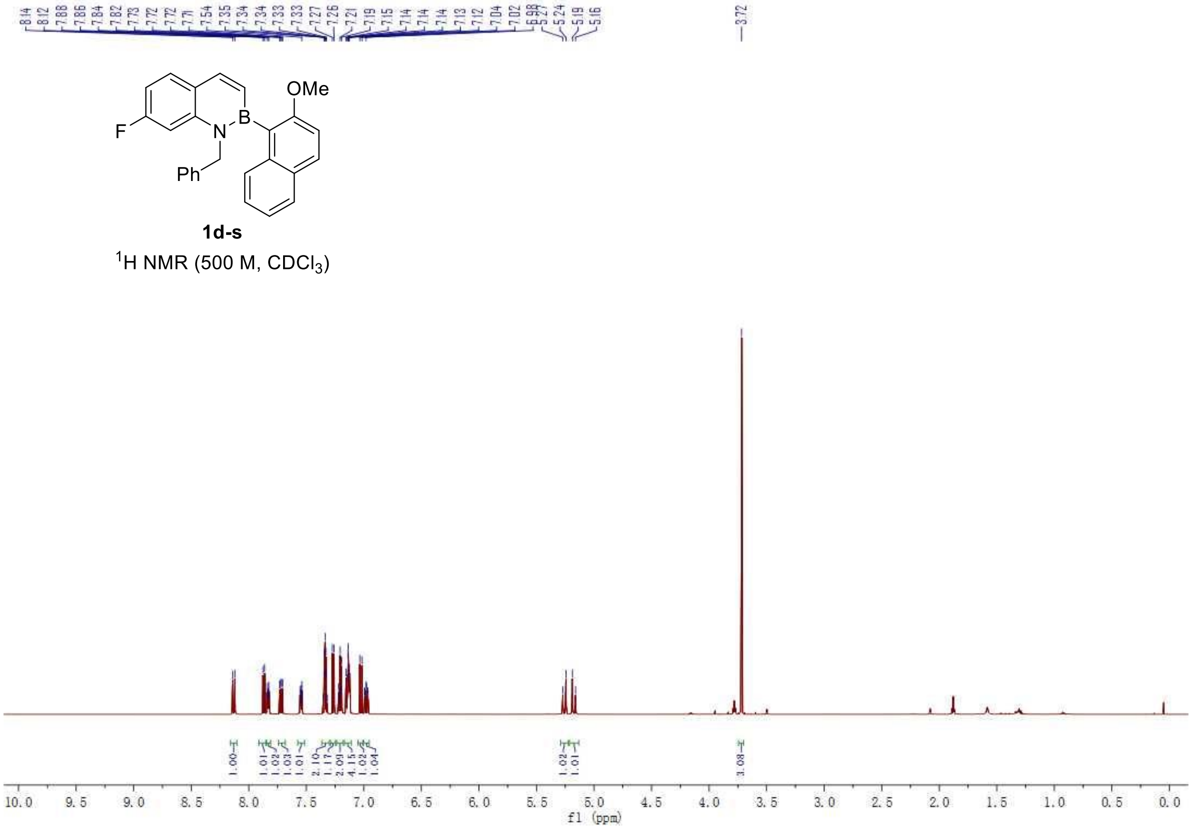

Supplementary Figure 20. ^{1}H NMR spectrum of 1d-s

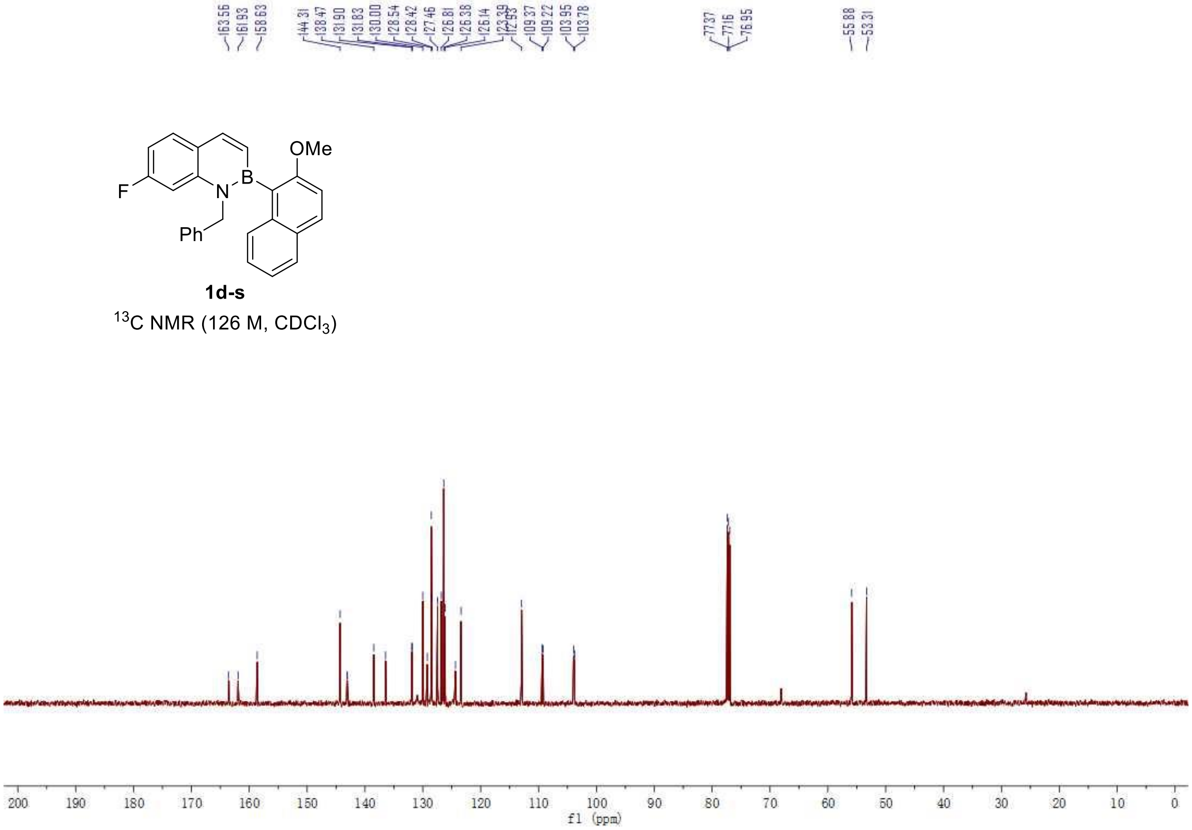

Supplementary Figure 21. ^{13}C NMR spectrum of 1d-s

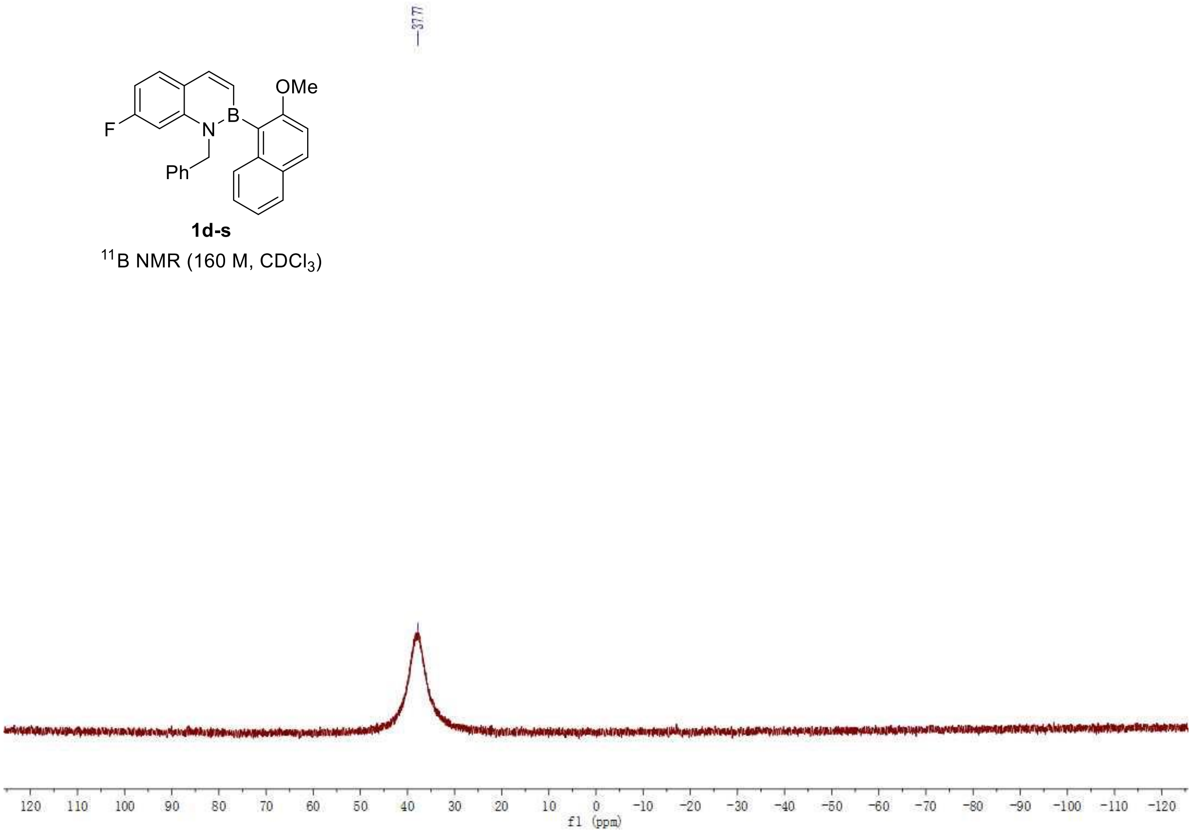

Supplementary Figure 22. ^{11}B NMR spectrum of 1d-s

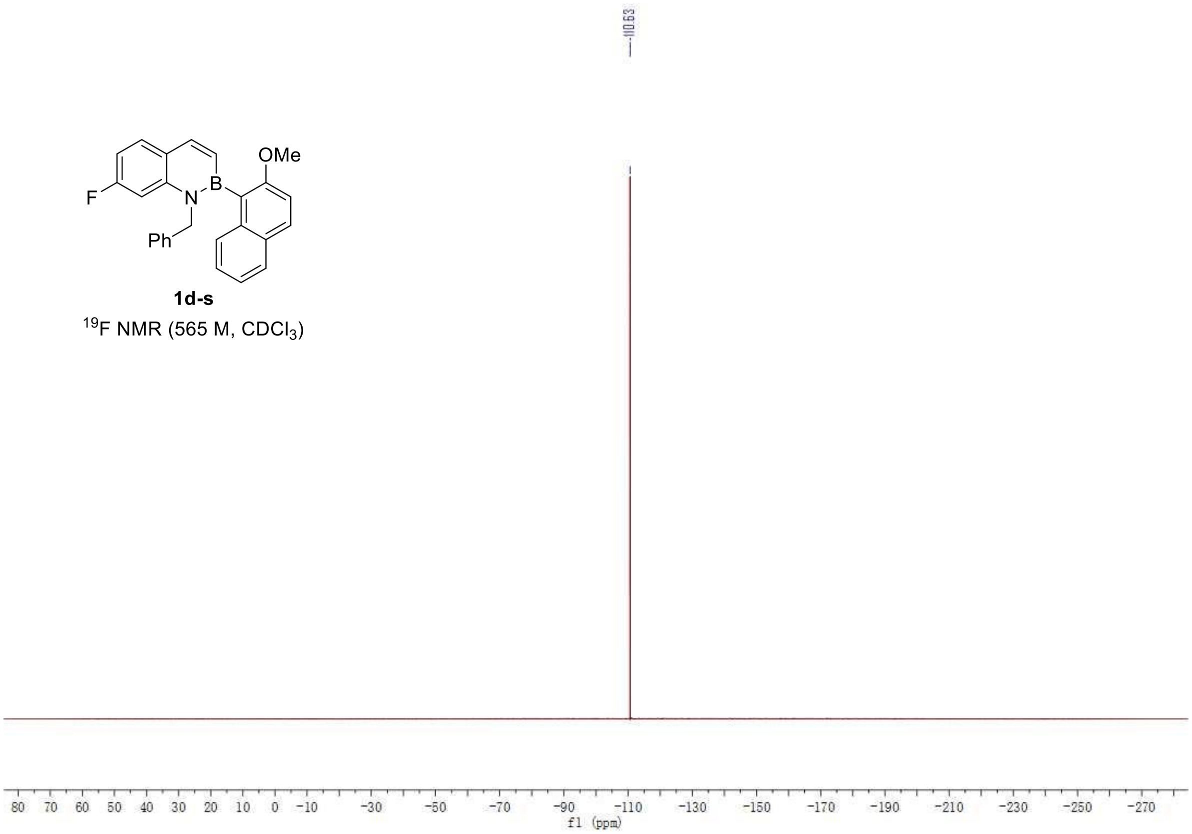

Supplementary Figure 23. ^{19}F NMR spectrum of 1d-s

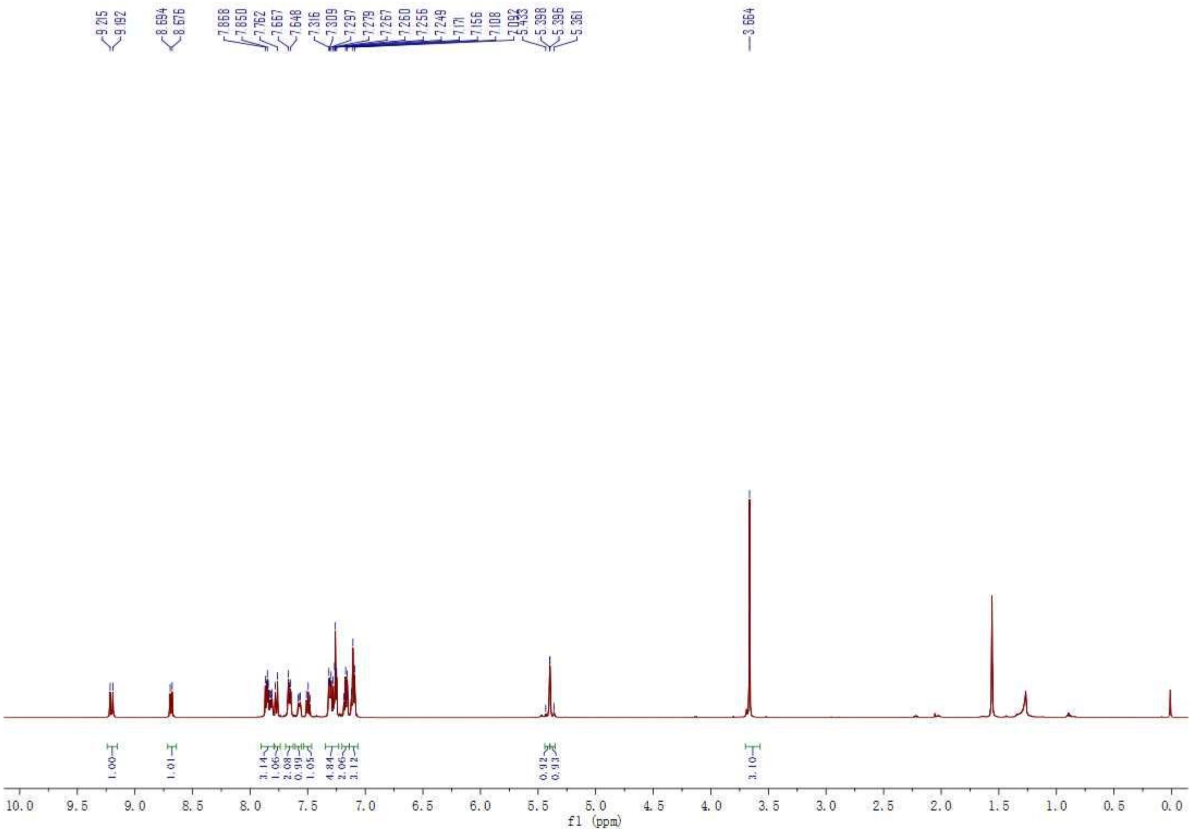

Supplementary Figure 24. ^{1}H NMR spectrum of 1e-s

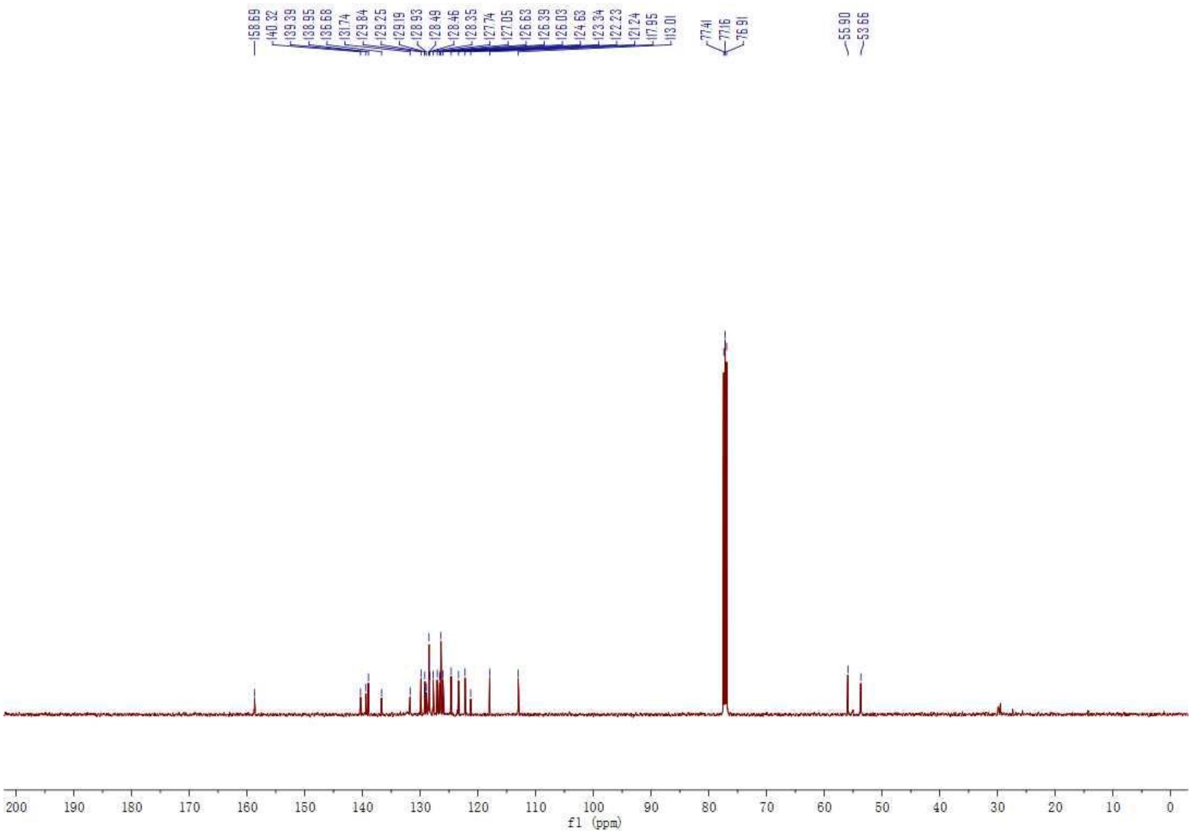

Supplementary Figure 25. ^{13}**C NMR spectrum of 1e-s**

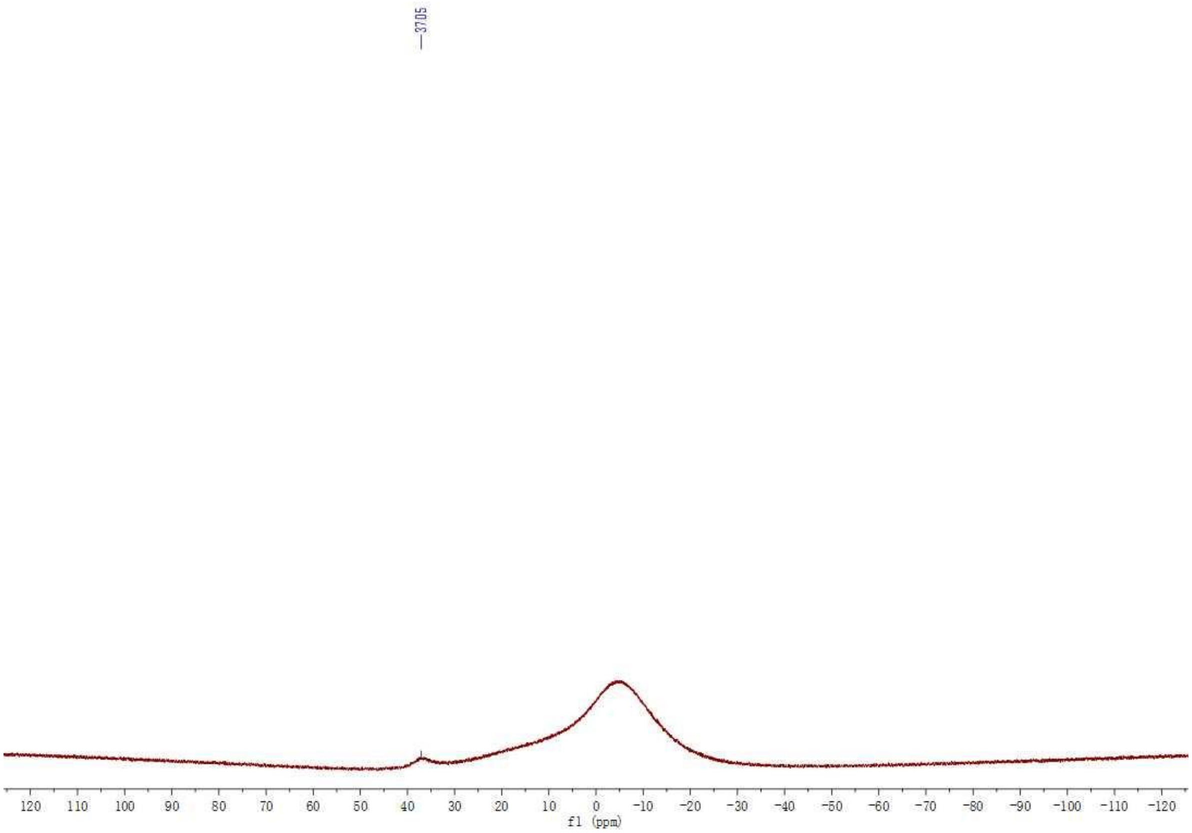

Supplementary Figure 26. ^{11}**B NMR spectrum of 1e-s**

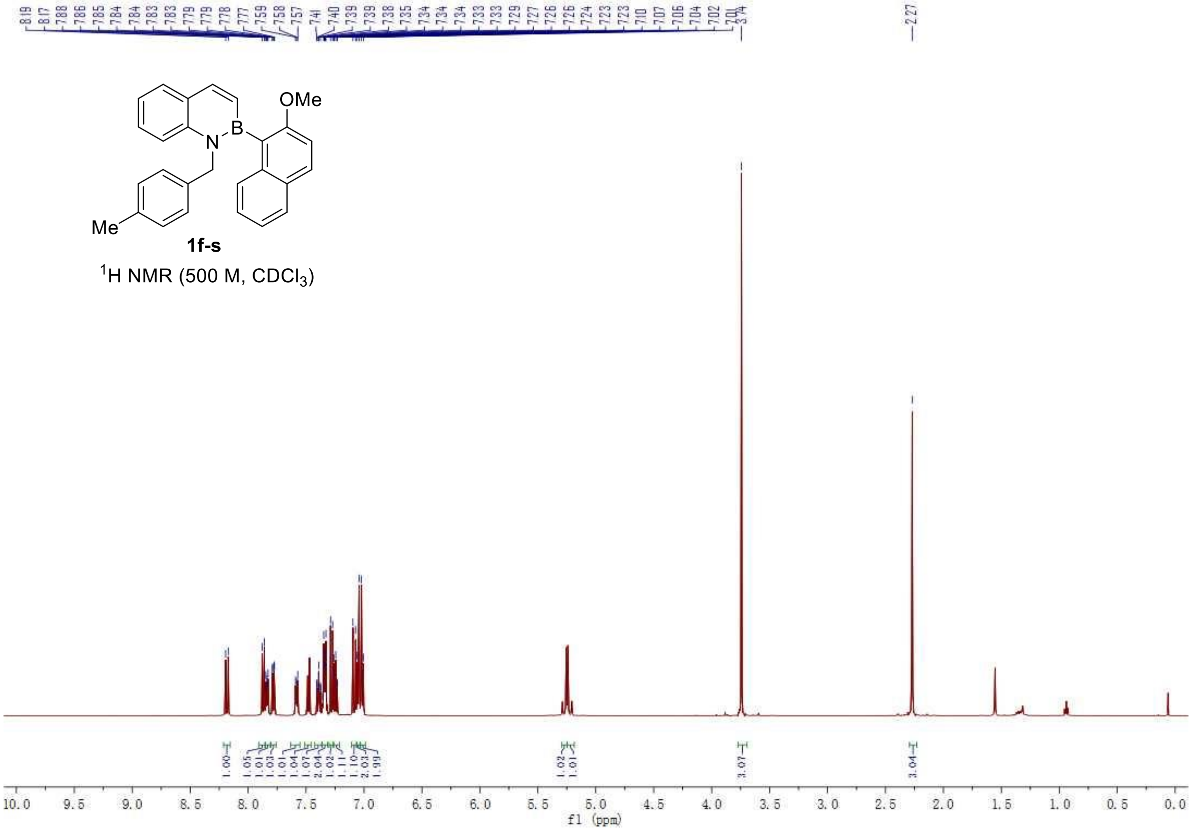

Supplementary Figure 27. ^{1}H NMR spectrum of 1f-s

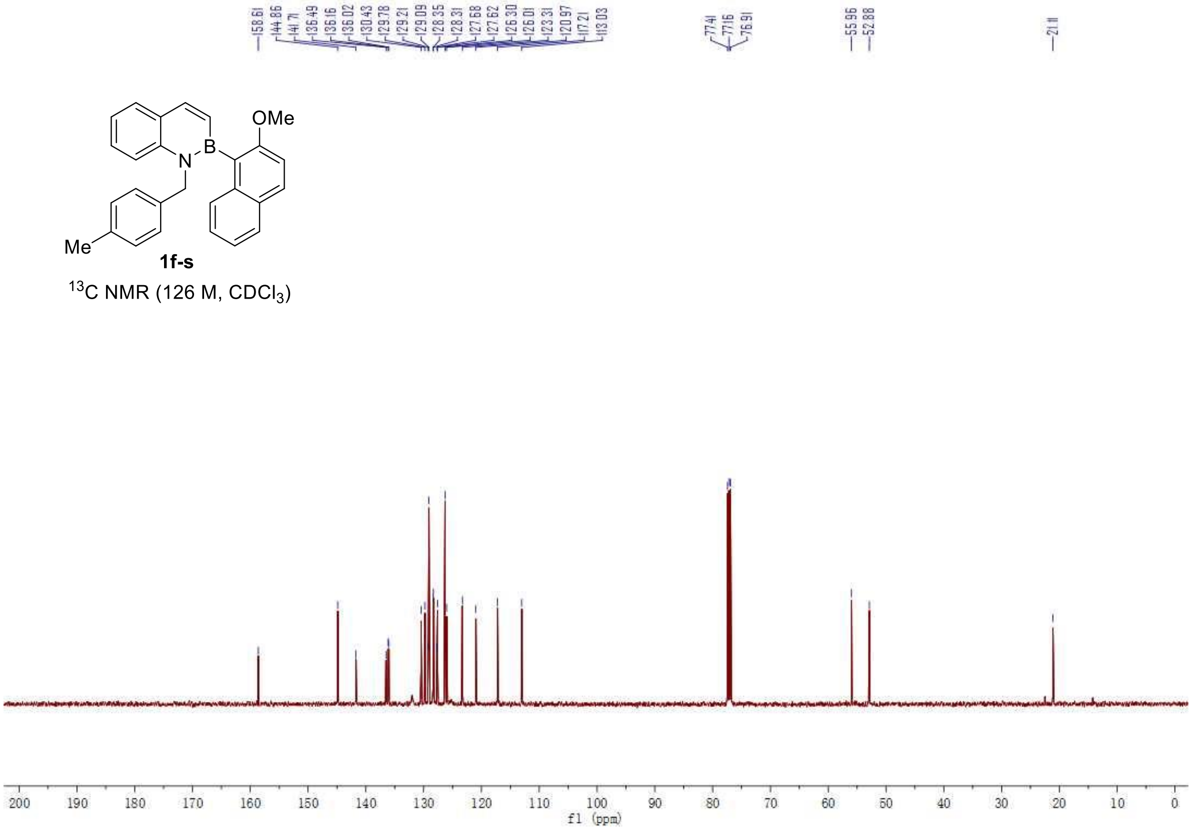

Supplementary Figure 28. ^{13}C NMR spectrum of 1f-s

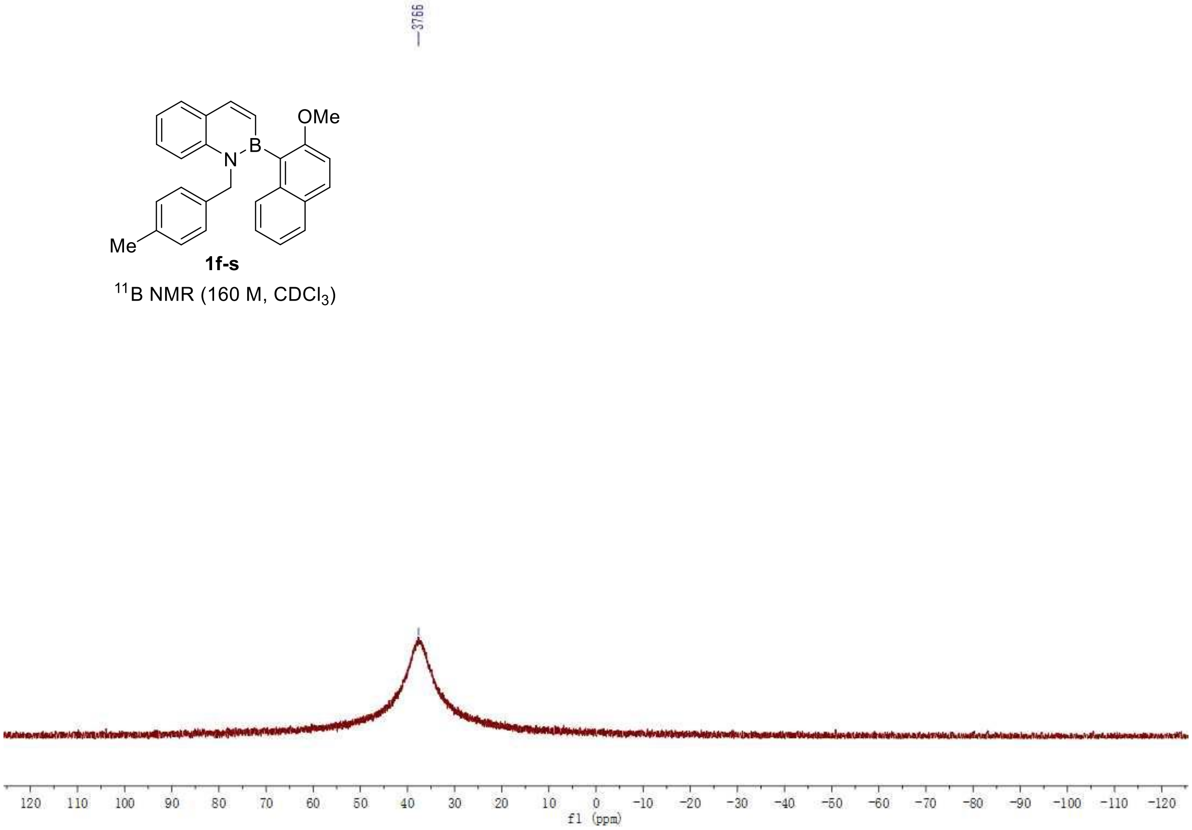

Supplementary Figure 29. ^{11}B NMR spectrum of 1f-s

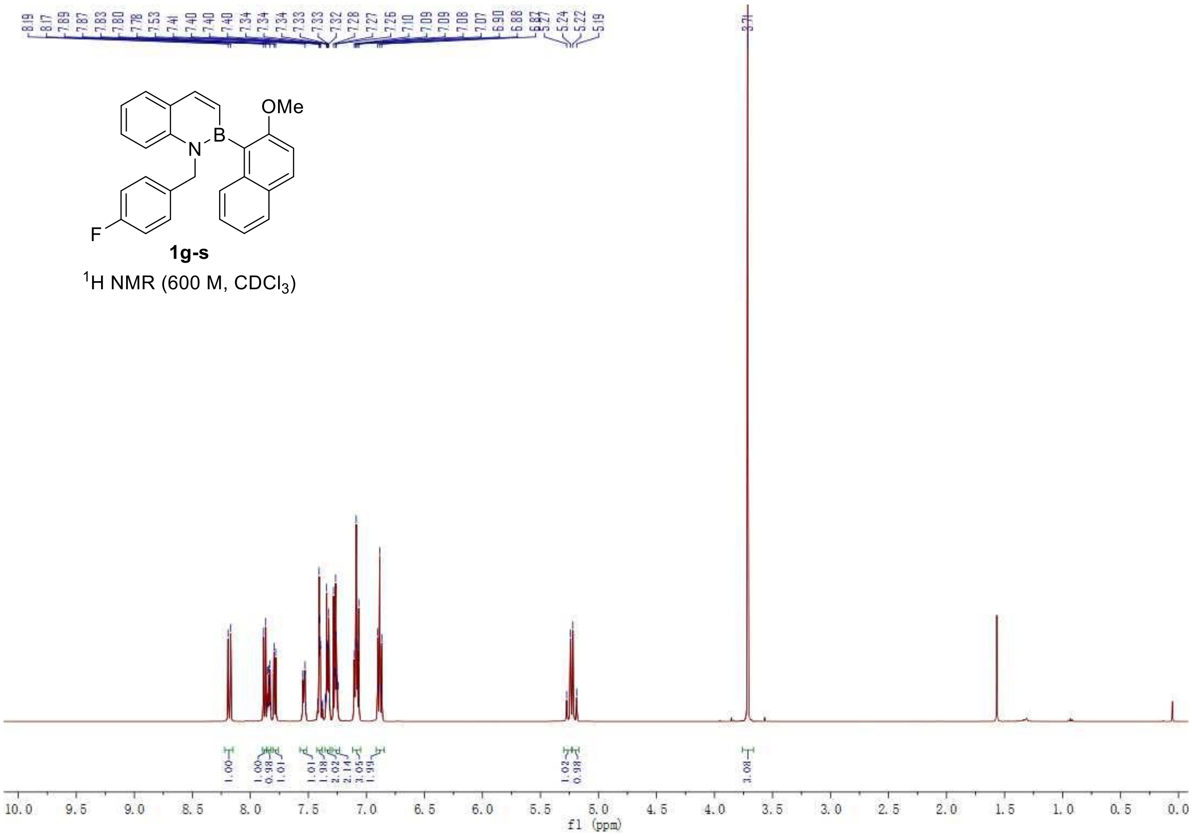

Supplementary Figure 30. ^{1}H NMR spectrum of 1g-s

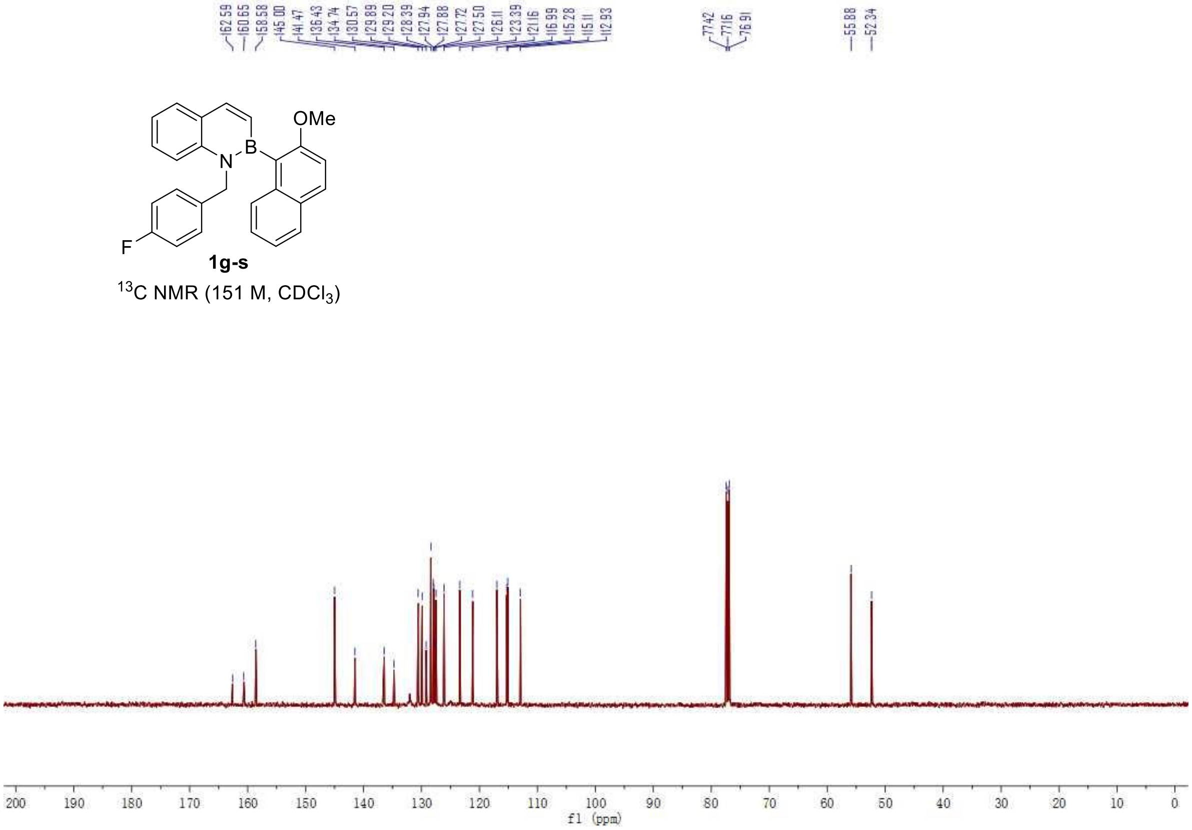

Supplementary Figure 31. ^{13}C NMR spectrum of 1g-s

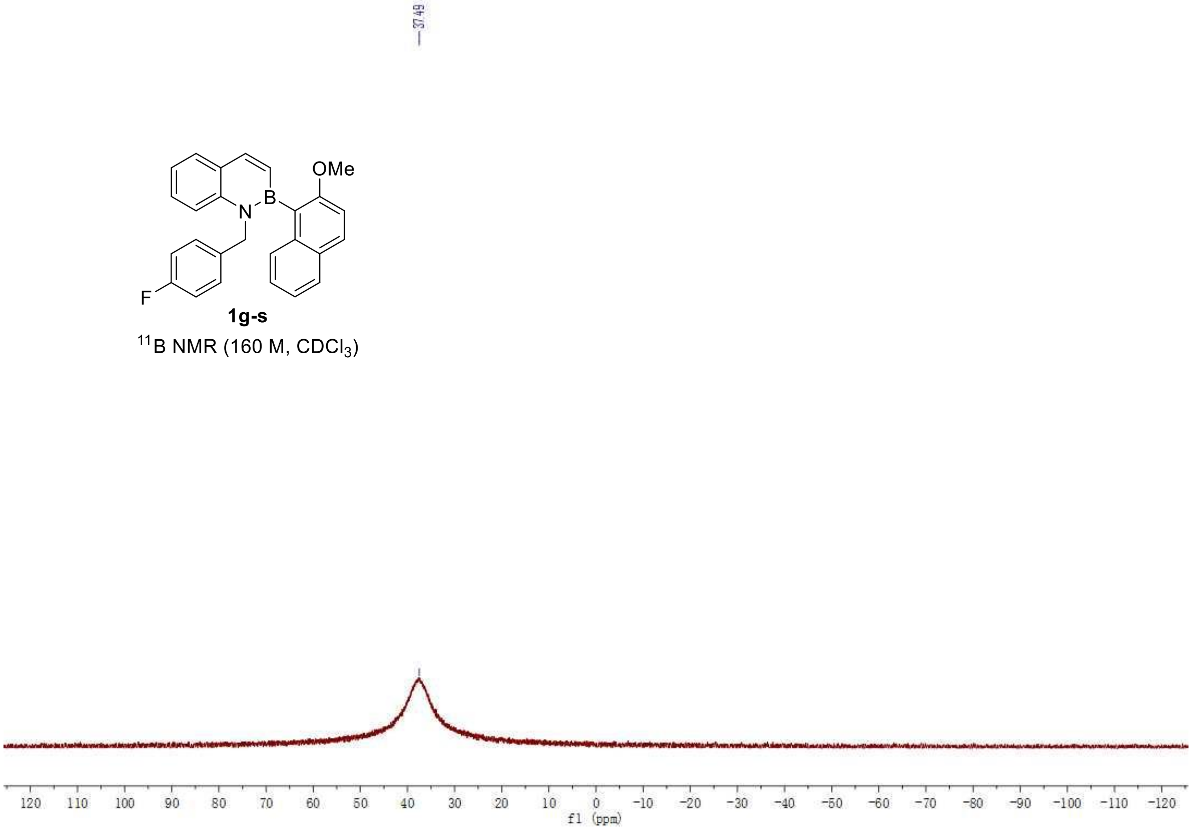

Supplementary Figure 32. ^{11}B NMR spectrum of 1g-s

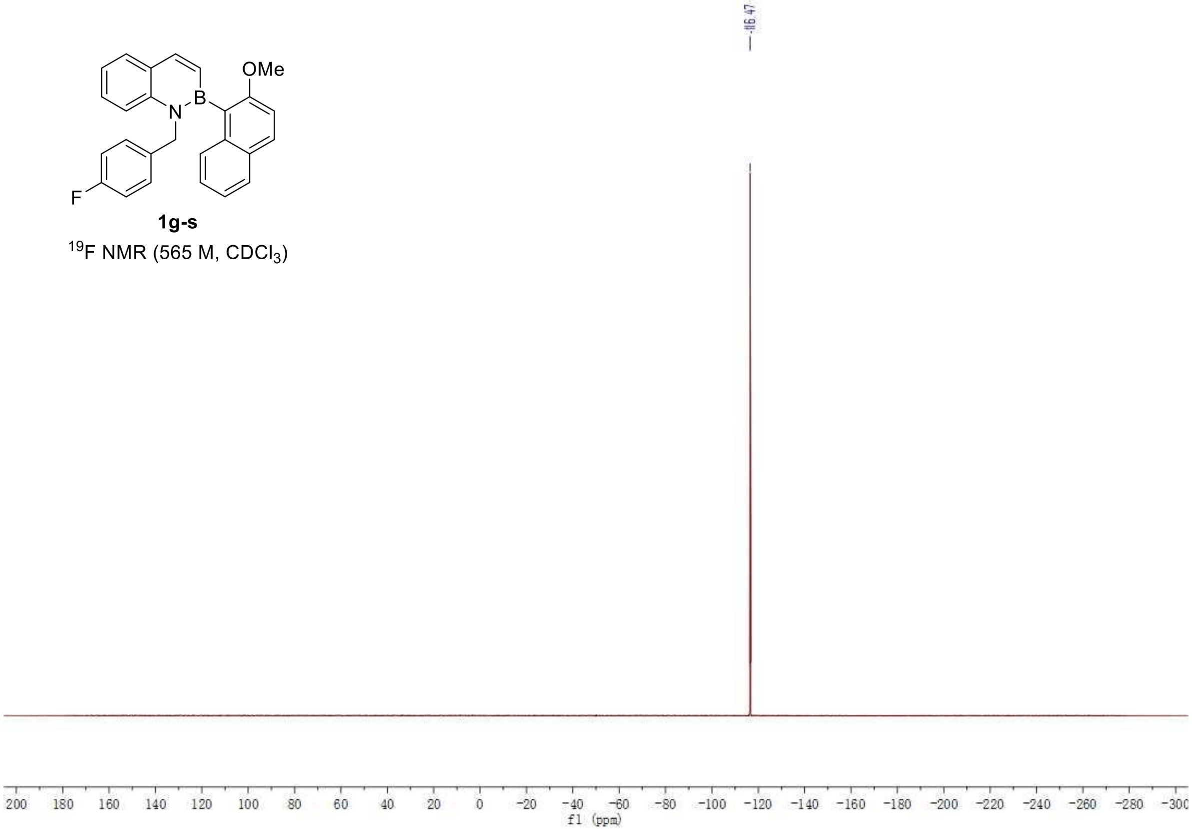

Supplementary Figure 33. ^{19}F NMR spectrum of 1g-s

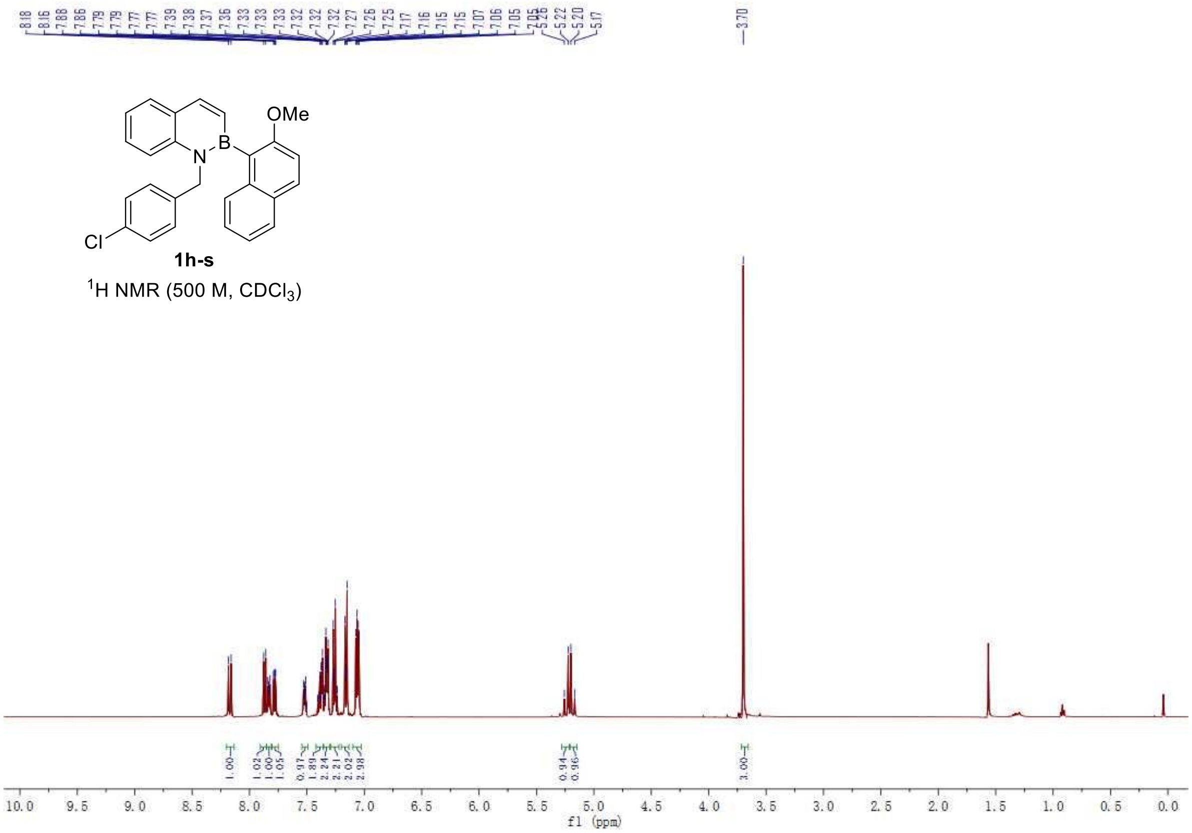

Supplementary Figure 34. ^{1}H NMR spectrum of 1h-s

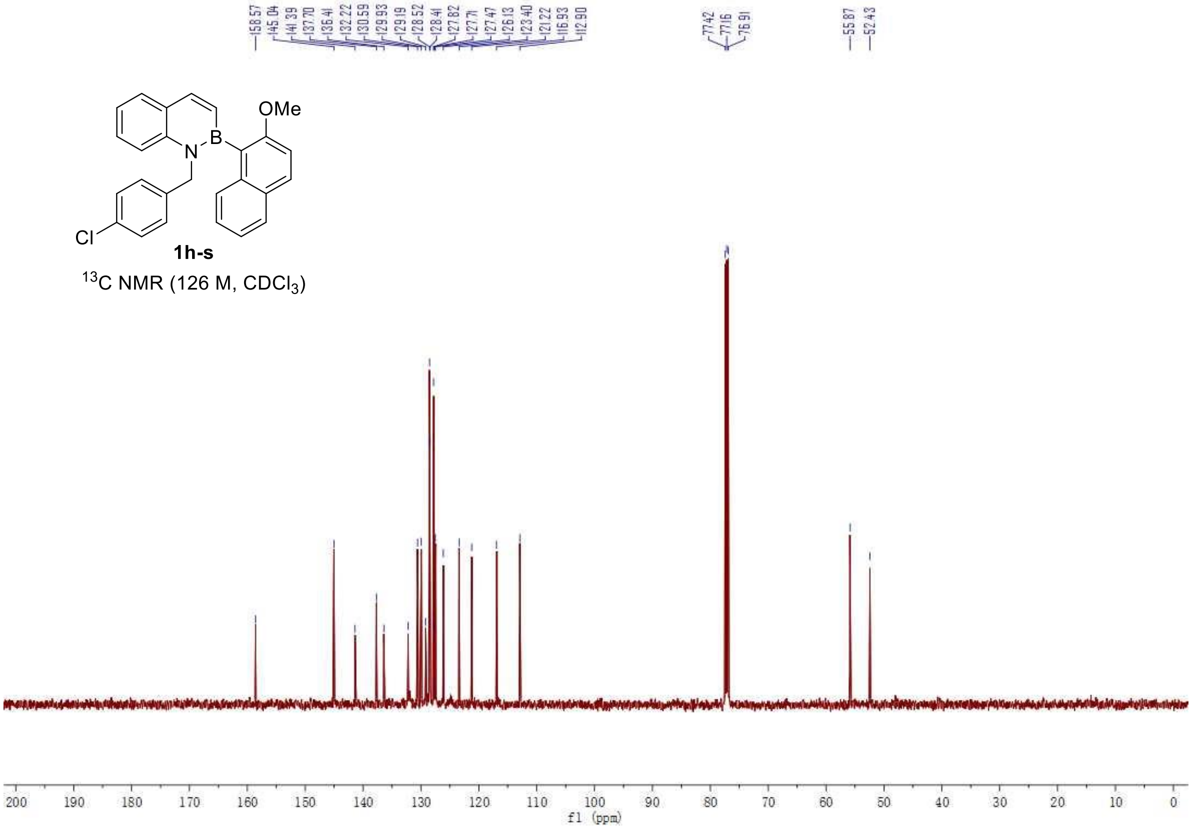

Supplementary Figure 35. ^{13}C NMR spectrum of 1h-s

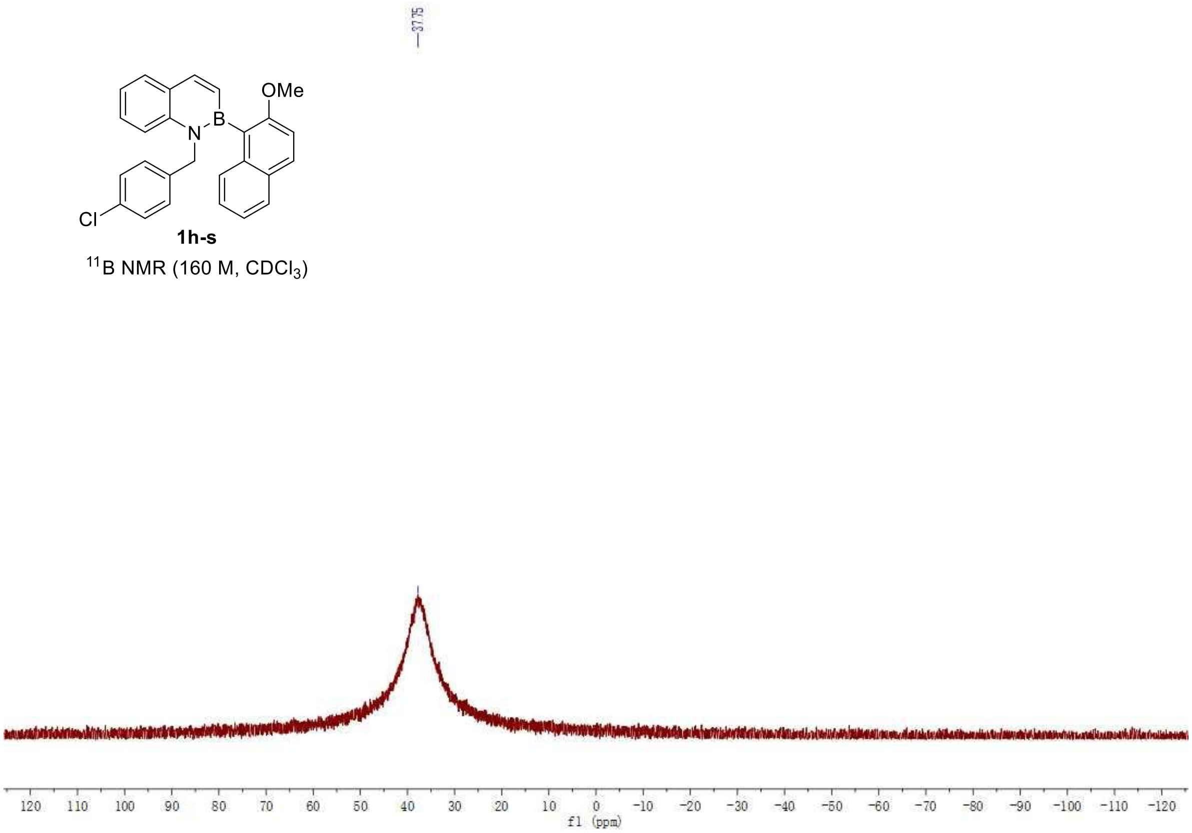

Supplementary Figure 36. ^{11}B NMR spectrum of 1h-s

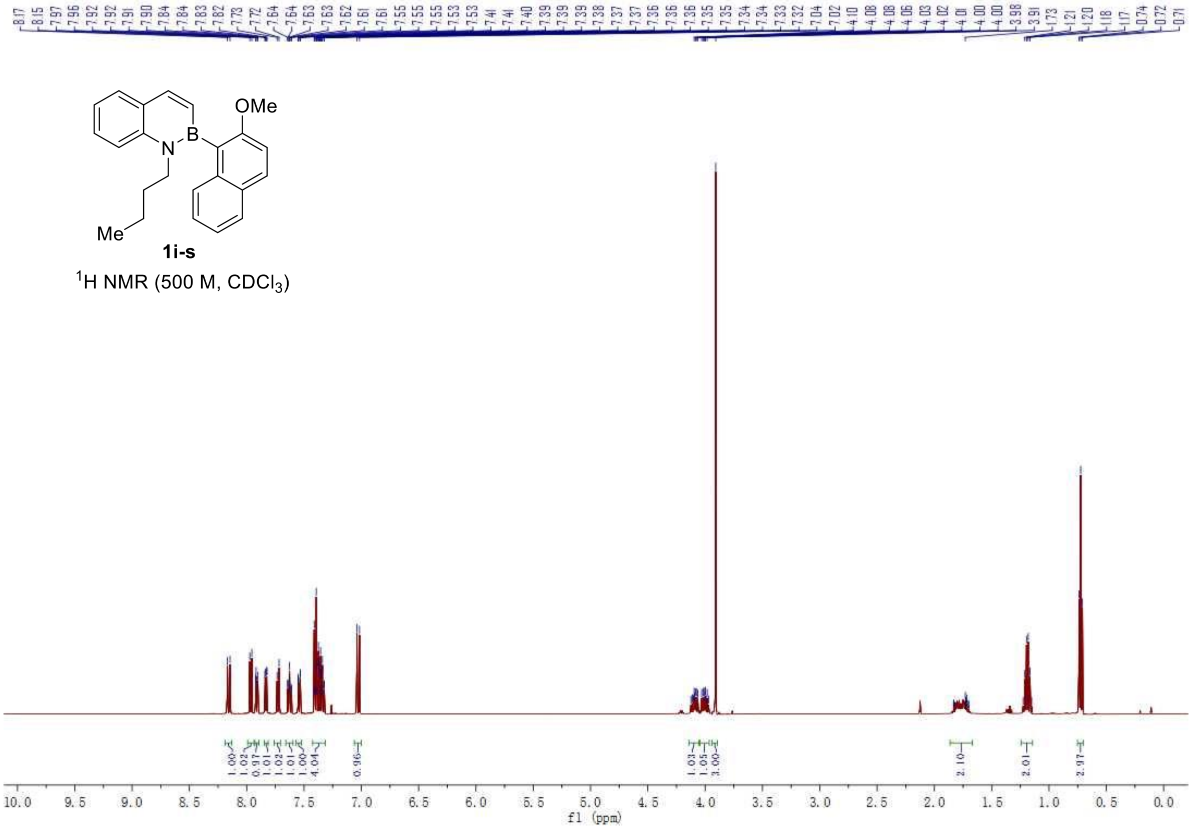

Supplementary Figure 37. ^{1}H NMR spectrum of 1i-s

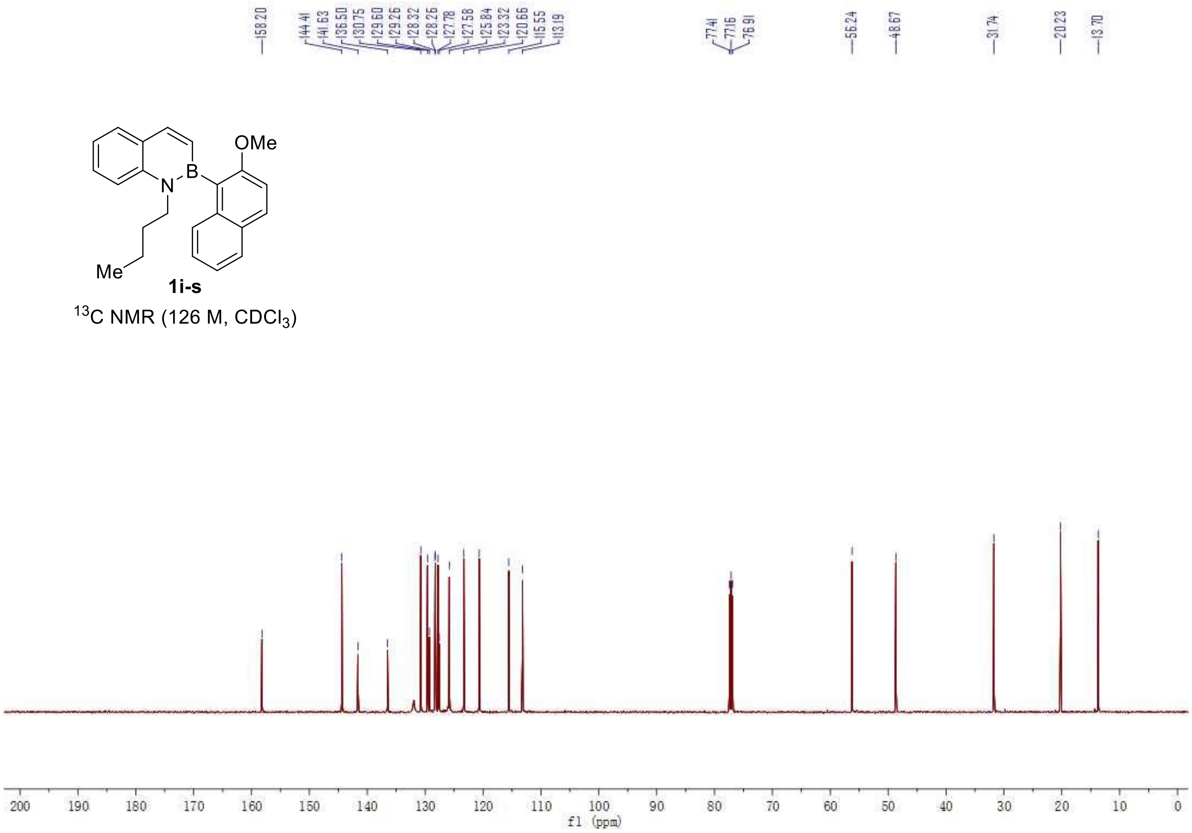

Supplementary Figure 38. ^{13}C NMR spectrum of 1i-s

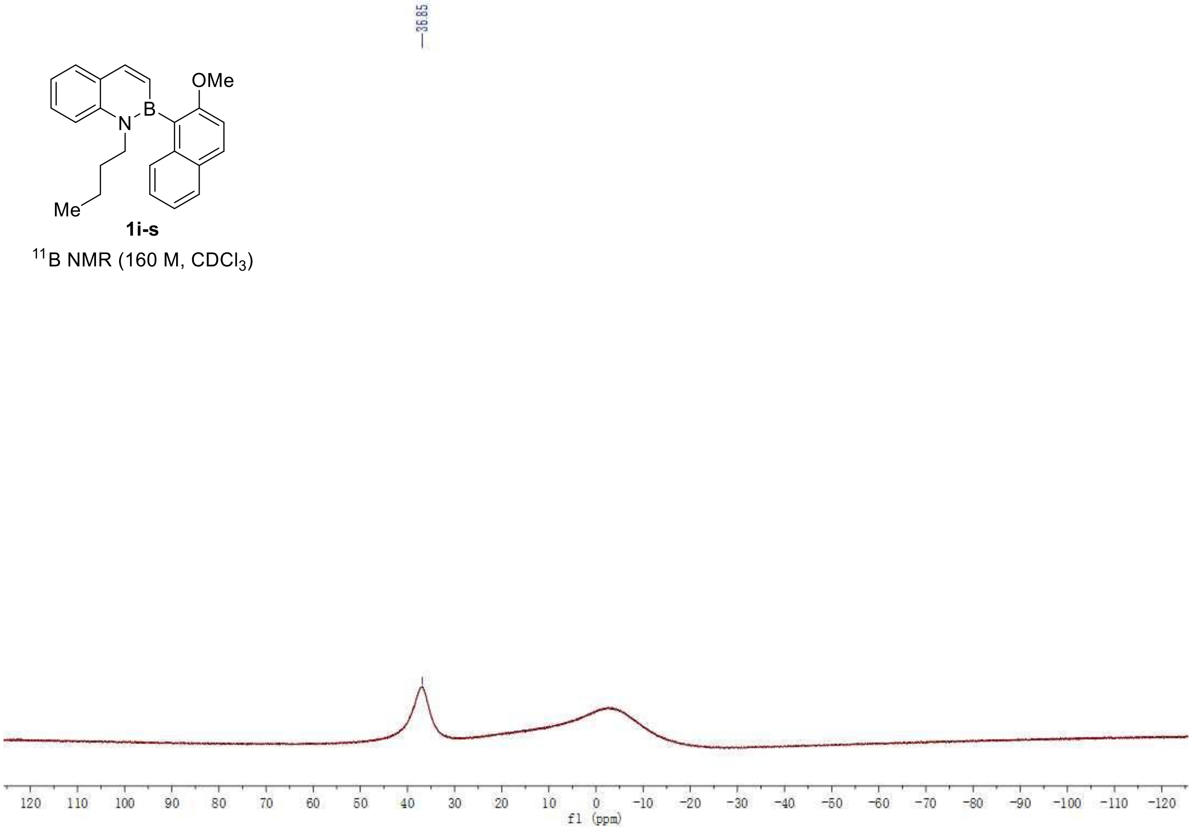

Supplementary Figure 39. ^{11}B NMR spectrum of 1i-s

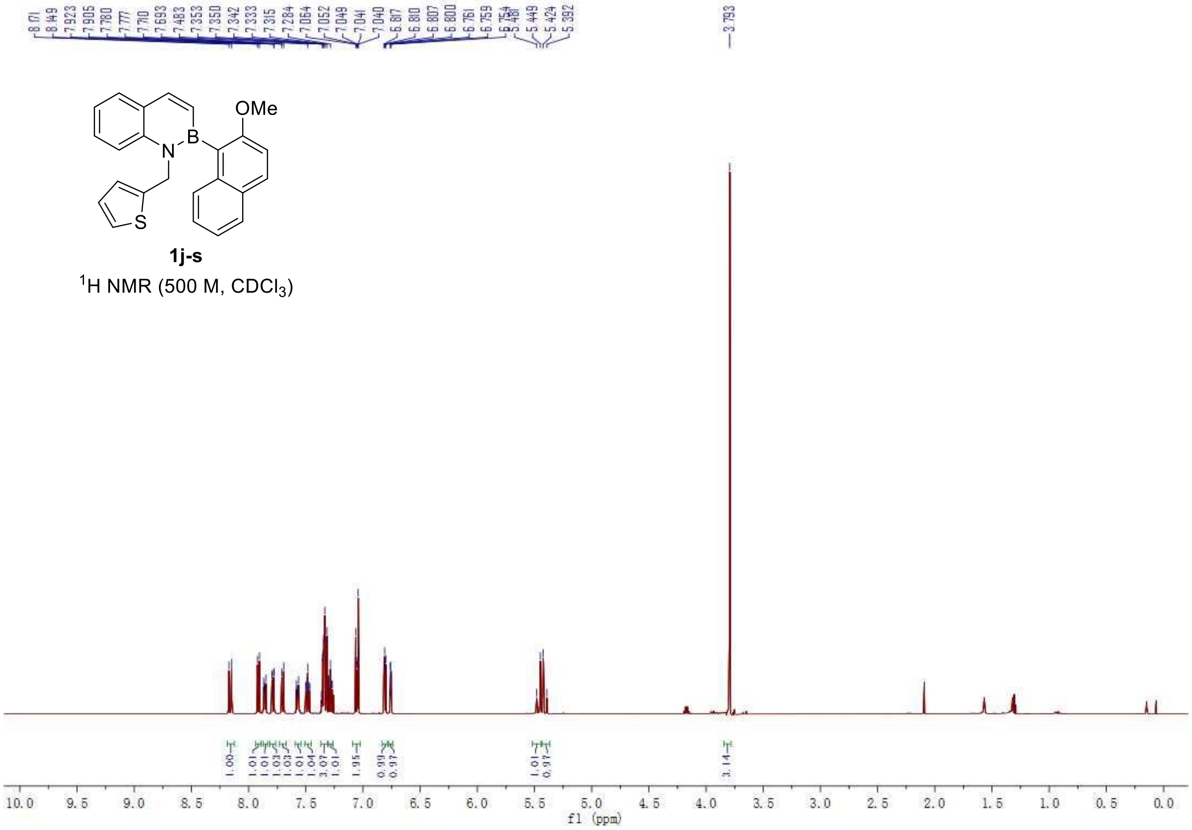

Supplementary Figure 40. ^{1}H NMR spectrum of 1j-s

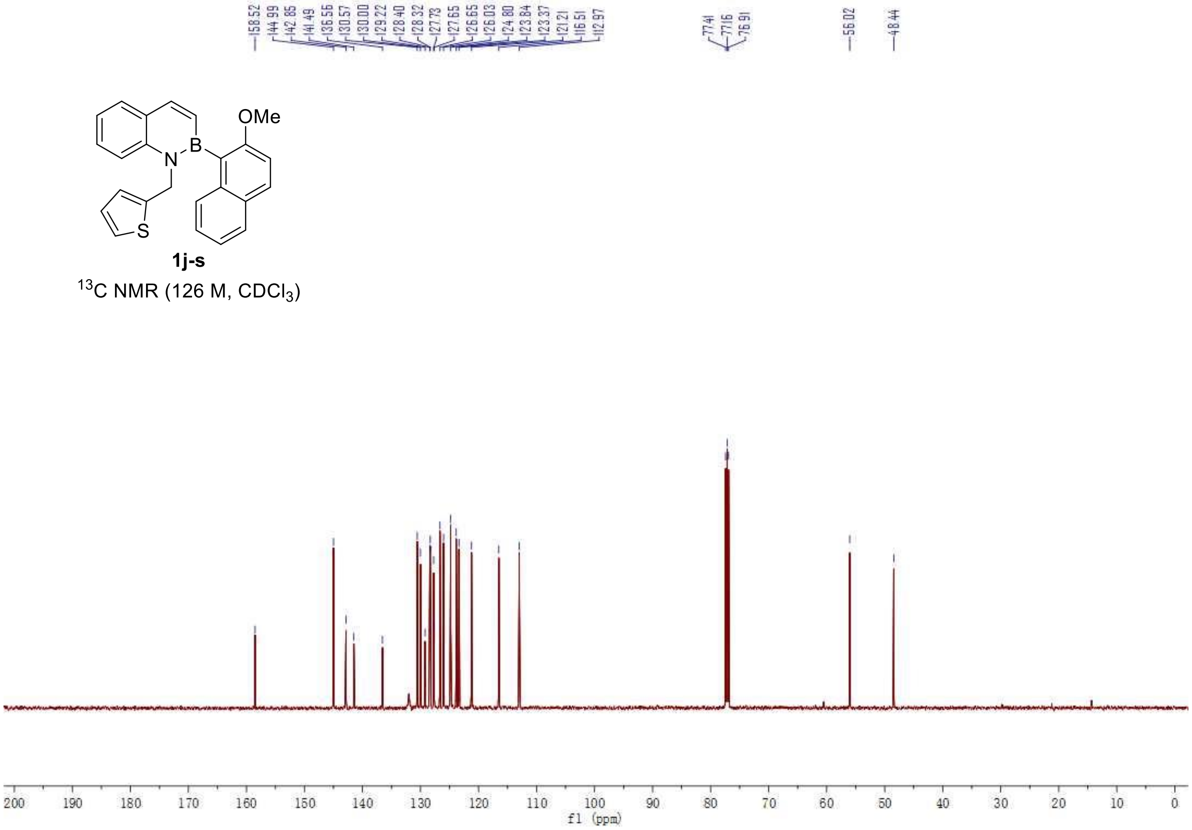

Supplementary Figure 41. ^{13}C NMR spectrum of 1j-s

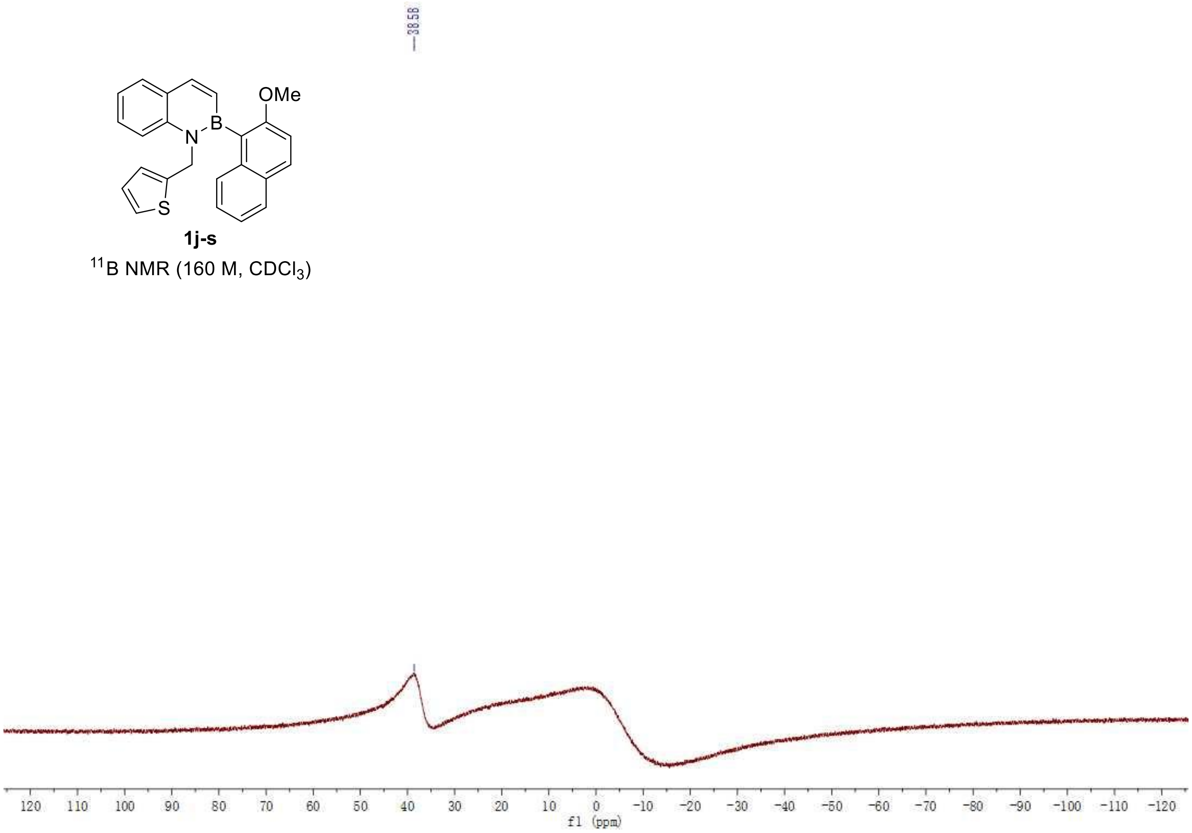

Supplementary Figure 42. ^{11}B NMR spectrum of 1j-s

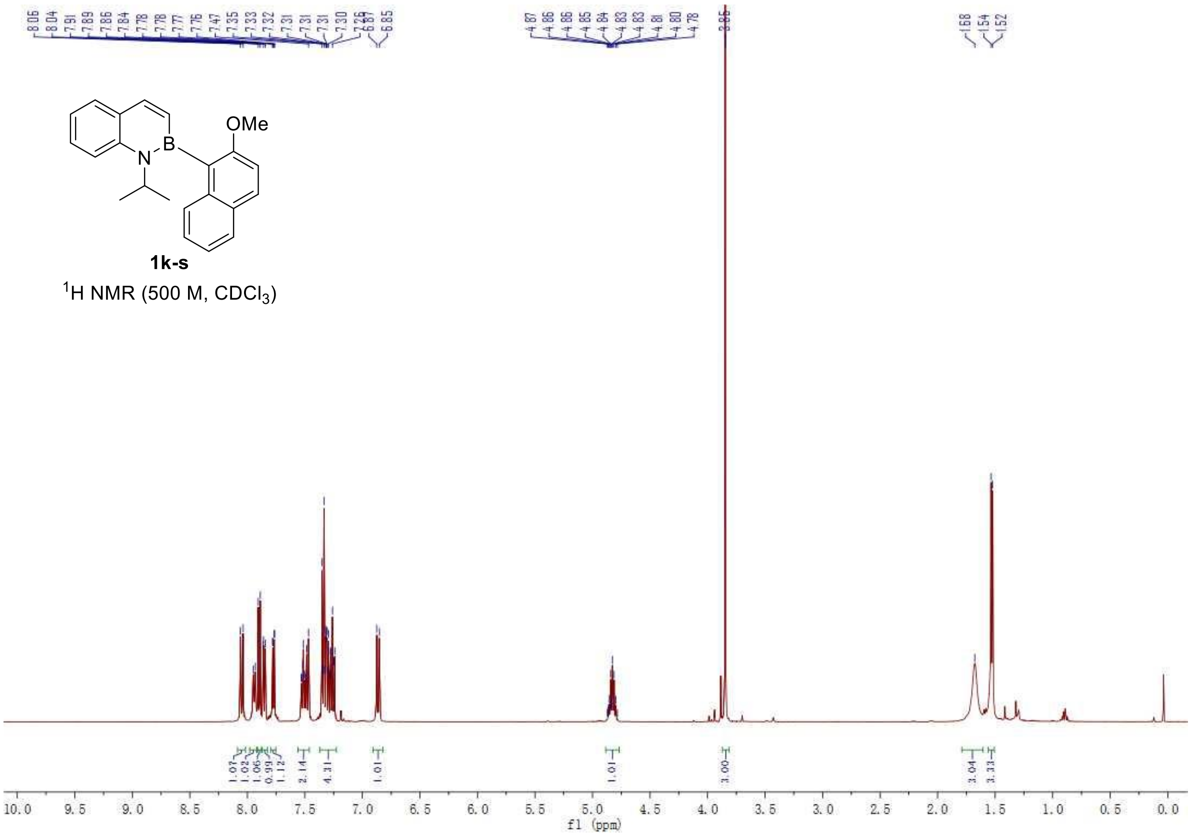

Supplementary Figure 43. ^{1}H NMR spectrum of 1k-s

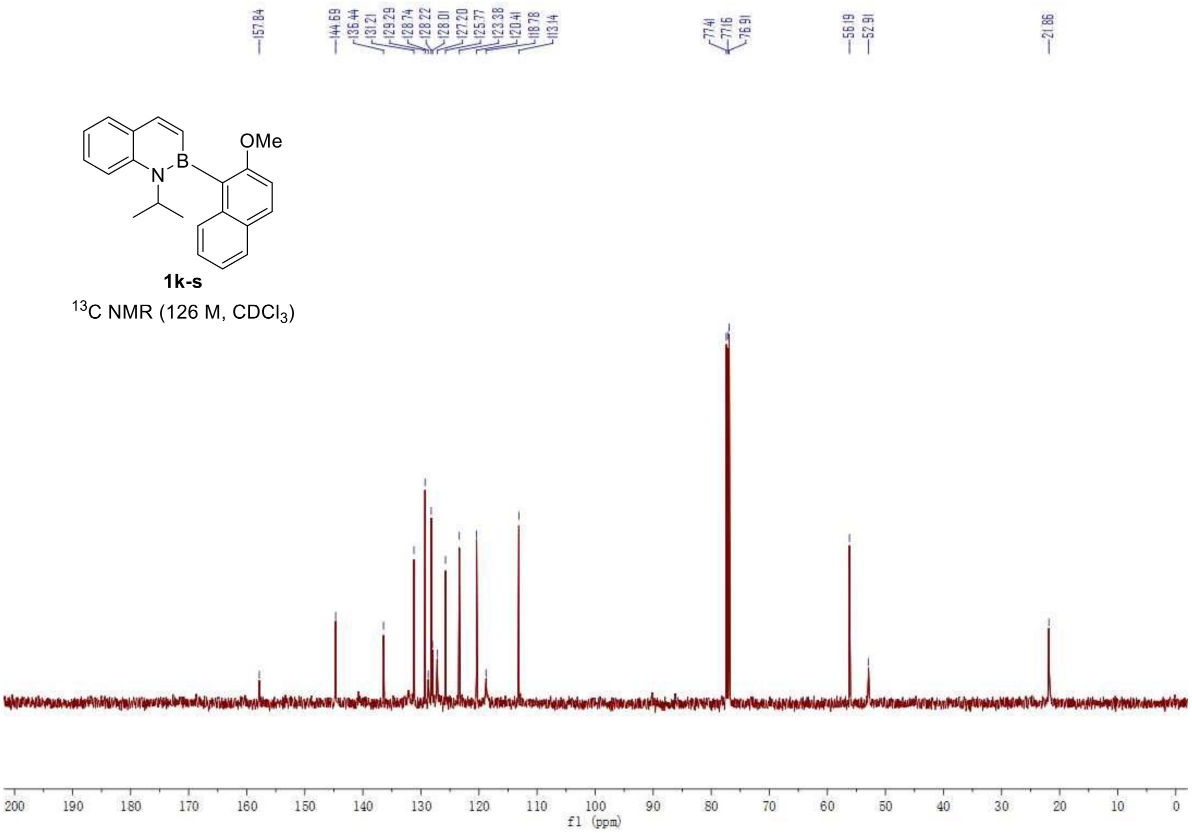

Supplementary Figure 44. ^{13}C NMR spectrum of 1k-s

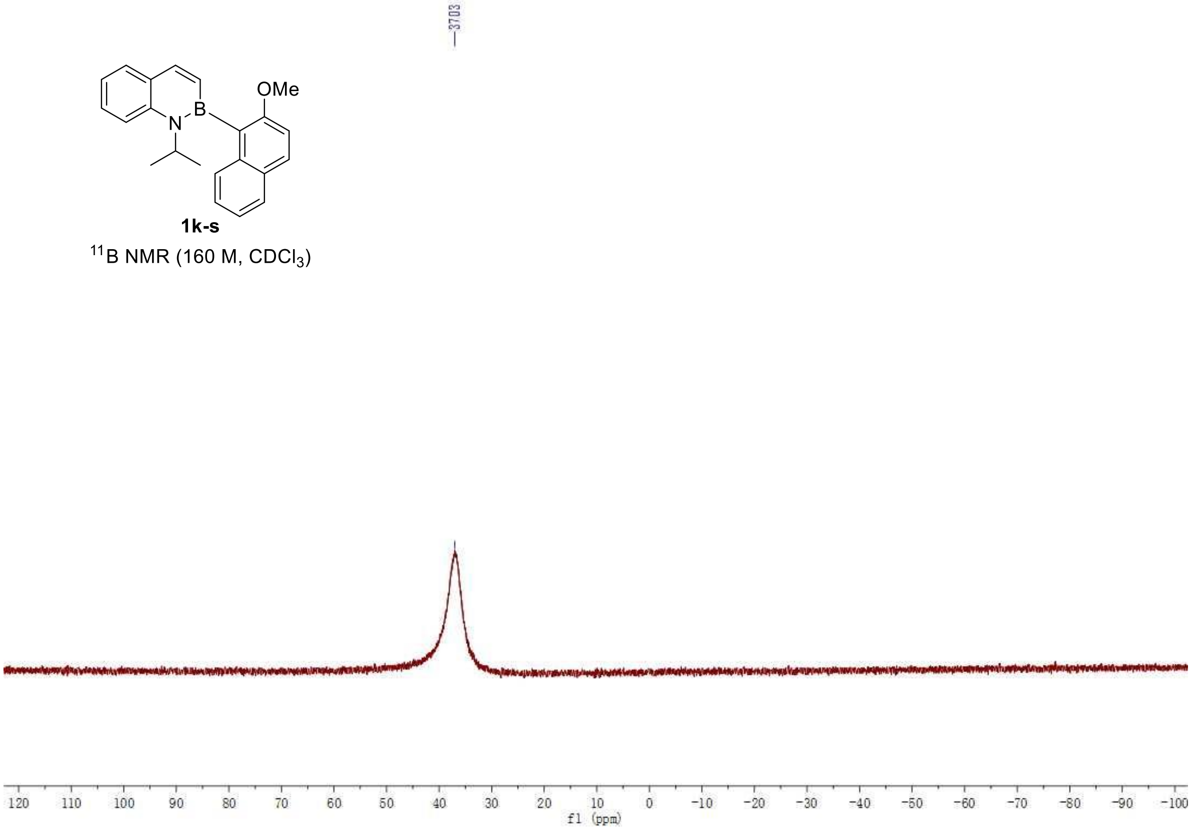

Supplementary Figure 45. ^{11}B NMR spectrum of 1k-s

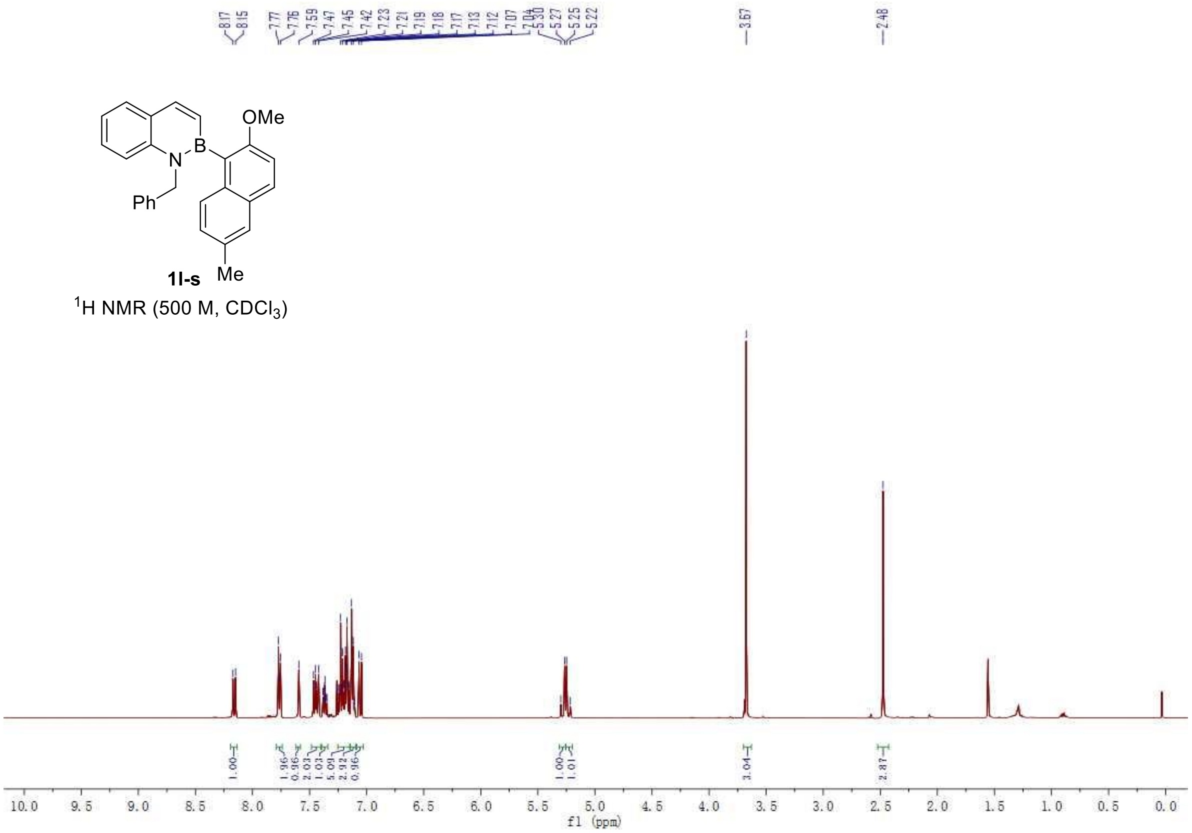

Supplementary Figure 46. ^{1}H NMR spectrum of 1l-s

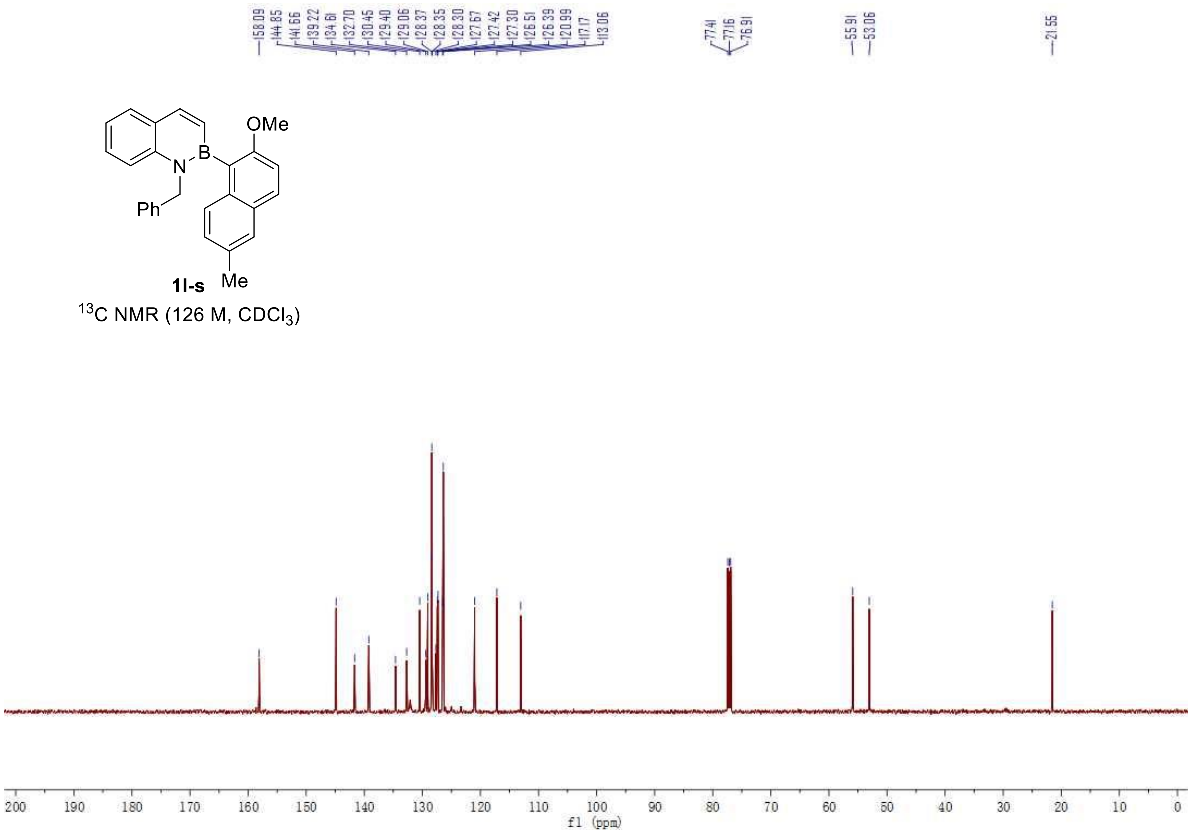

Supplementary Figure 47. ^{13}C NMR spectrum of 1l-s

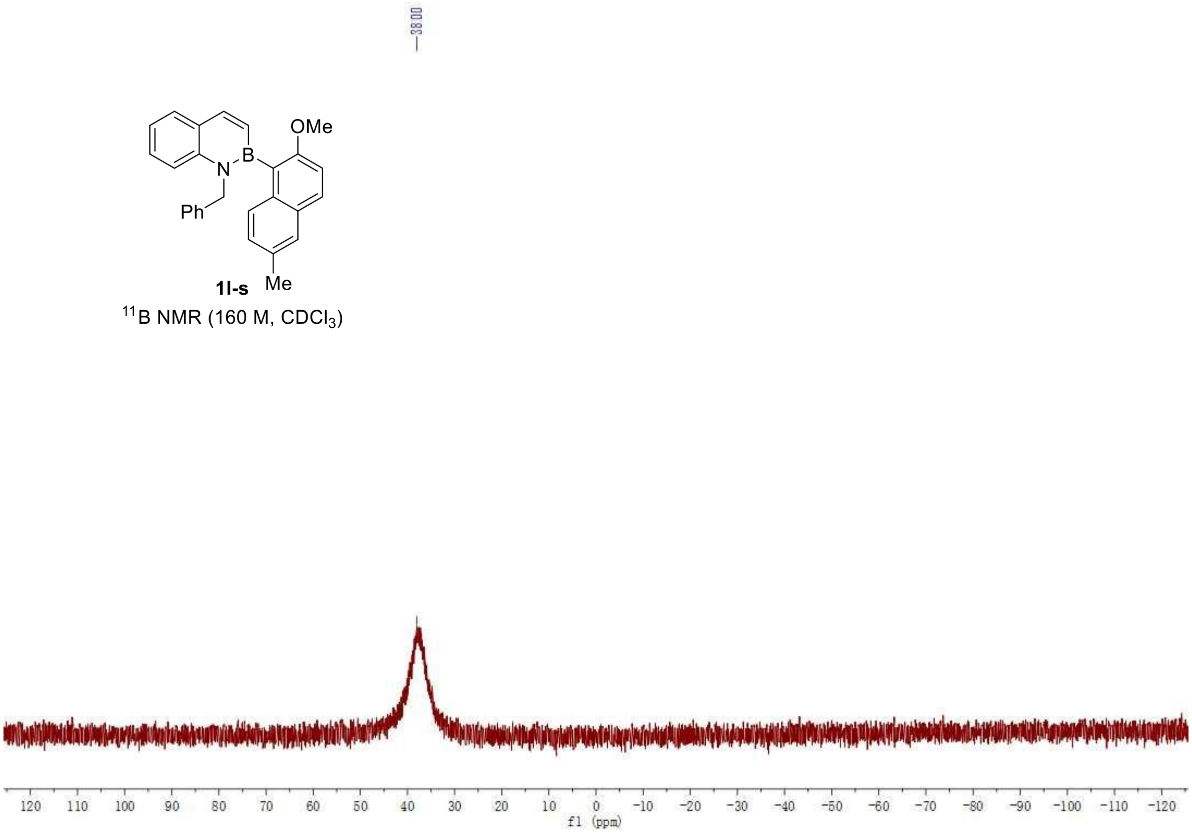

Supplementary Figure 48. ^{11}B NMR spectrum of 1l-s

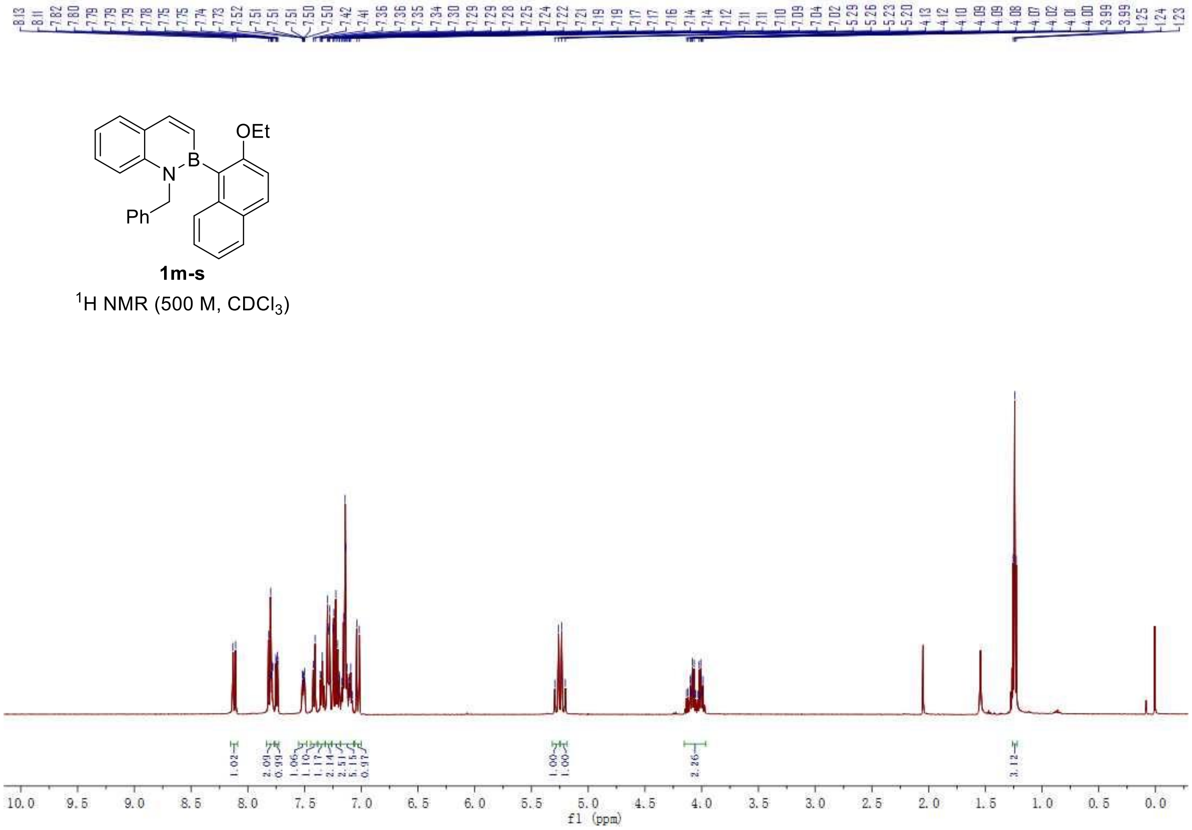

Supplementary Figure 49. ^{1}H NMR spectrum of 1m-s

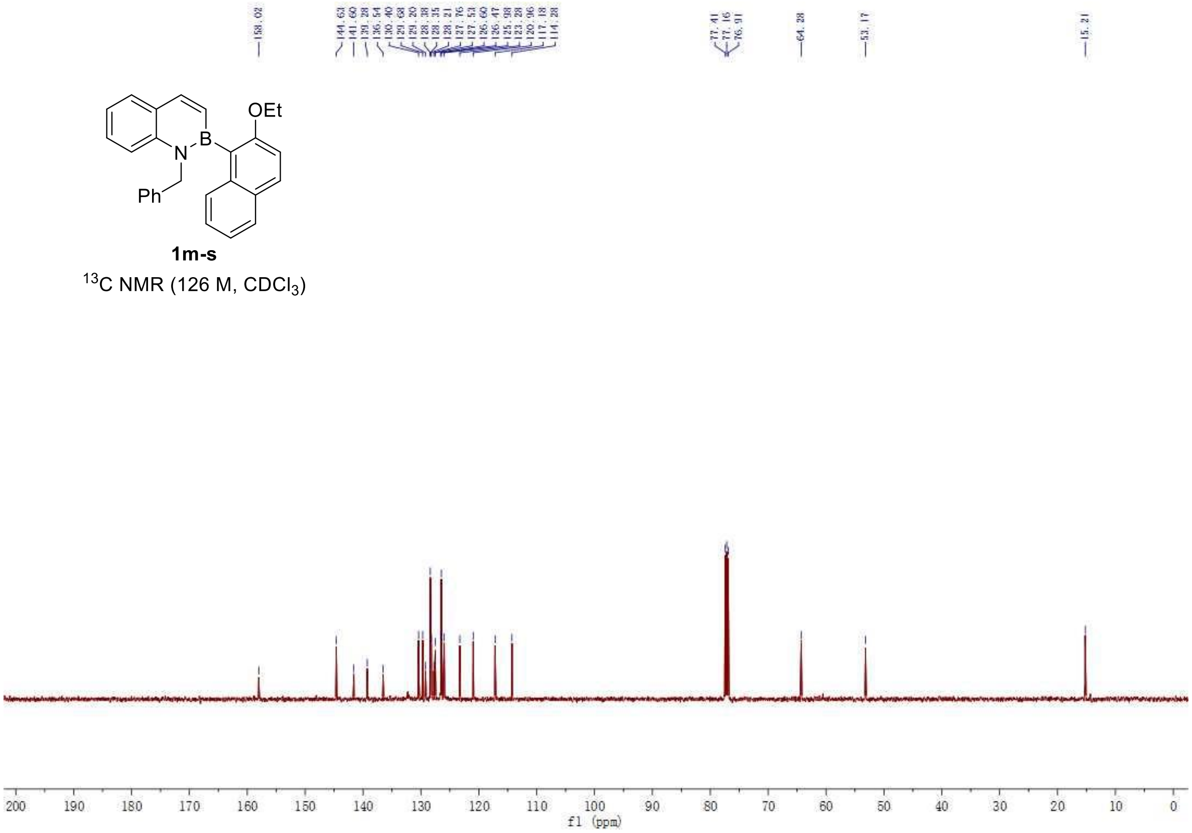

Supplementary Figure 50. ^{13}C NMR spectrum of 1m-s

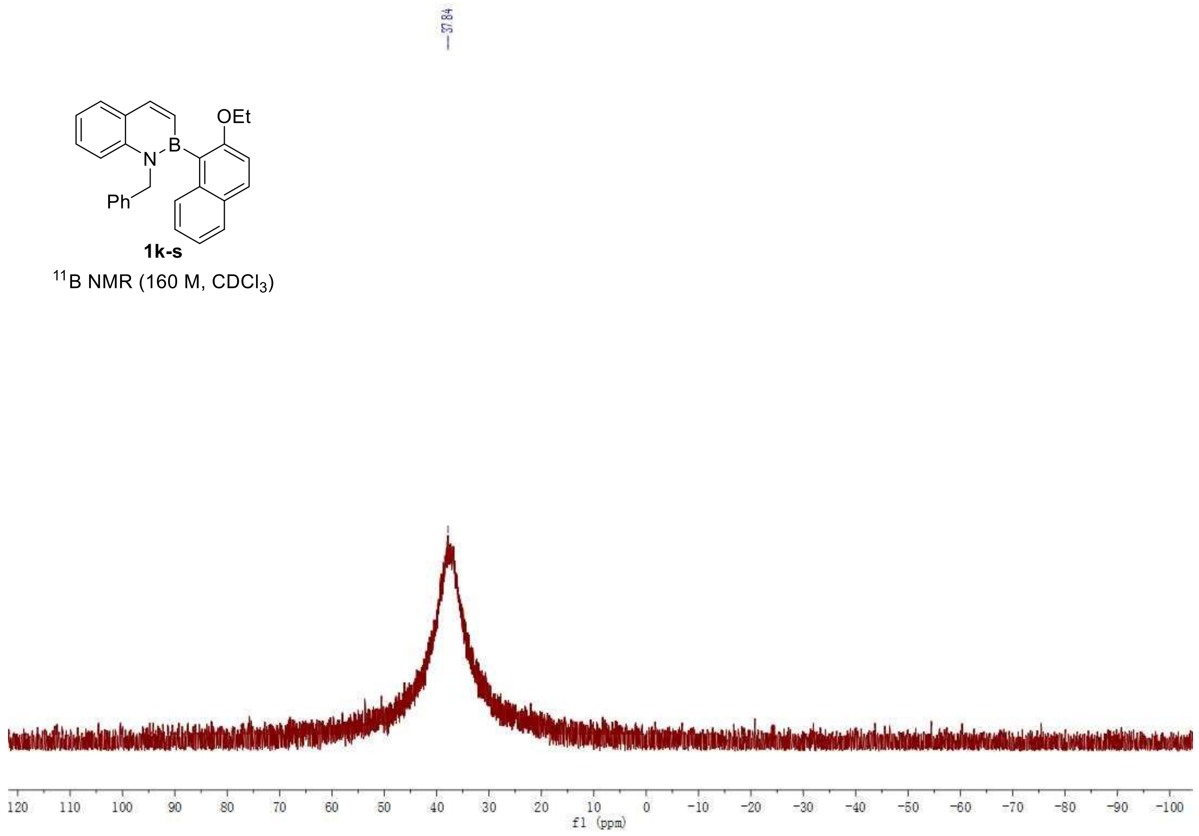

Supplementary Figure 51. ^{11}B NMR spectrum of 1m-s

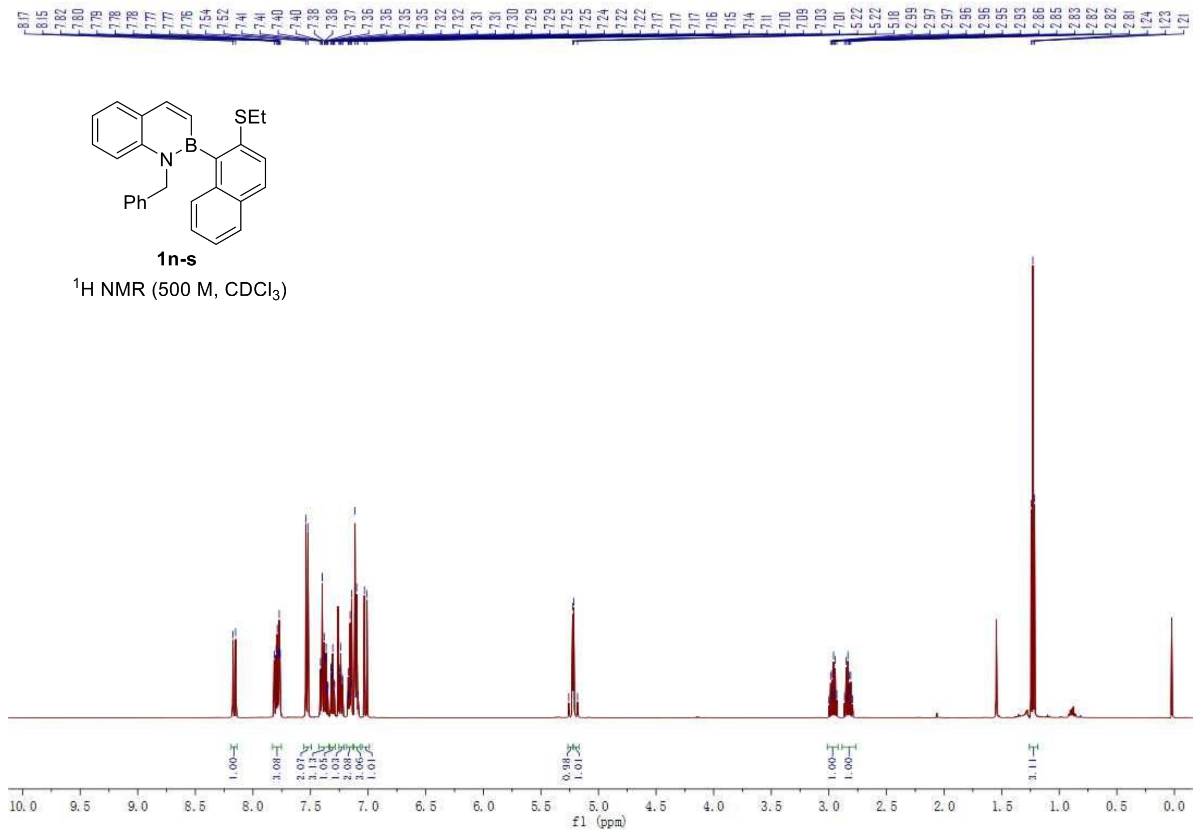

Supplementary Figure 52. ^{1}H NMR spectrum of 1n-s

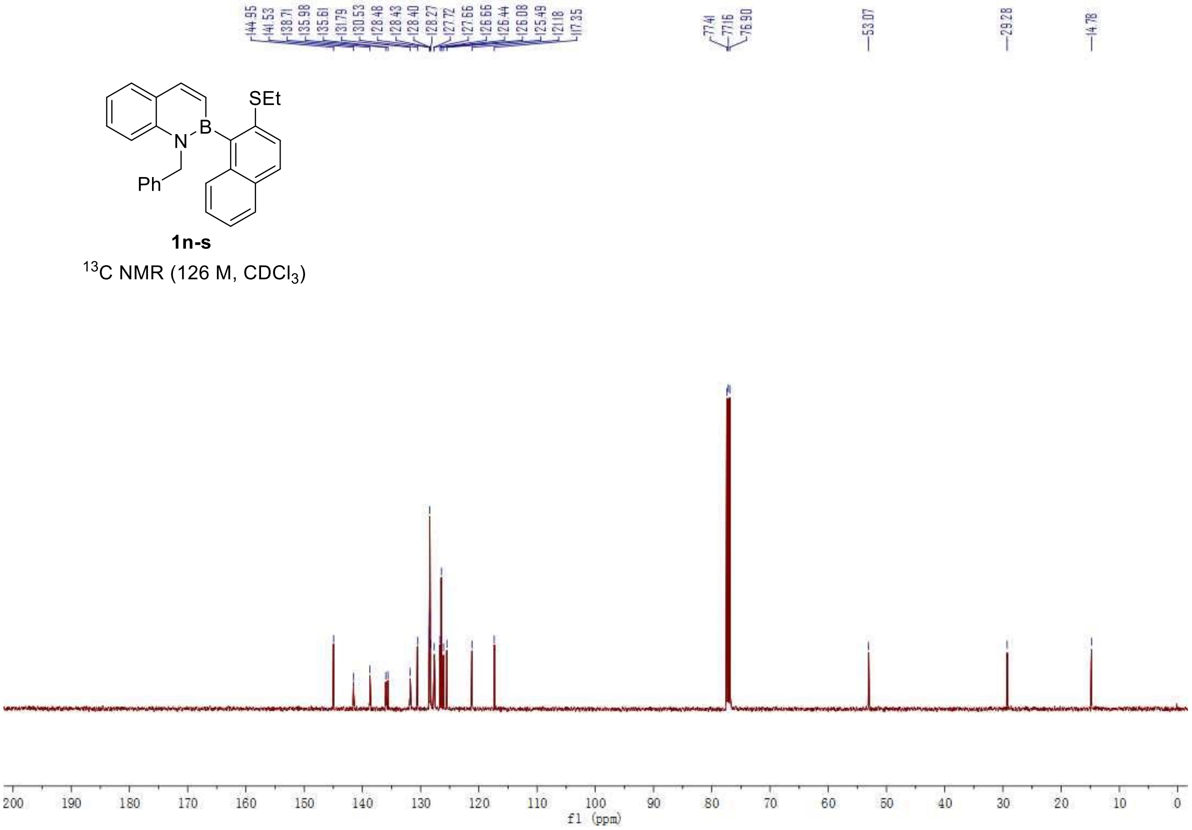

Supplementary Figure 53. ^{13}C NMR spectrum of 1n-s

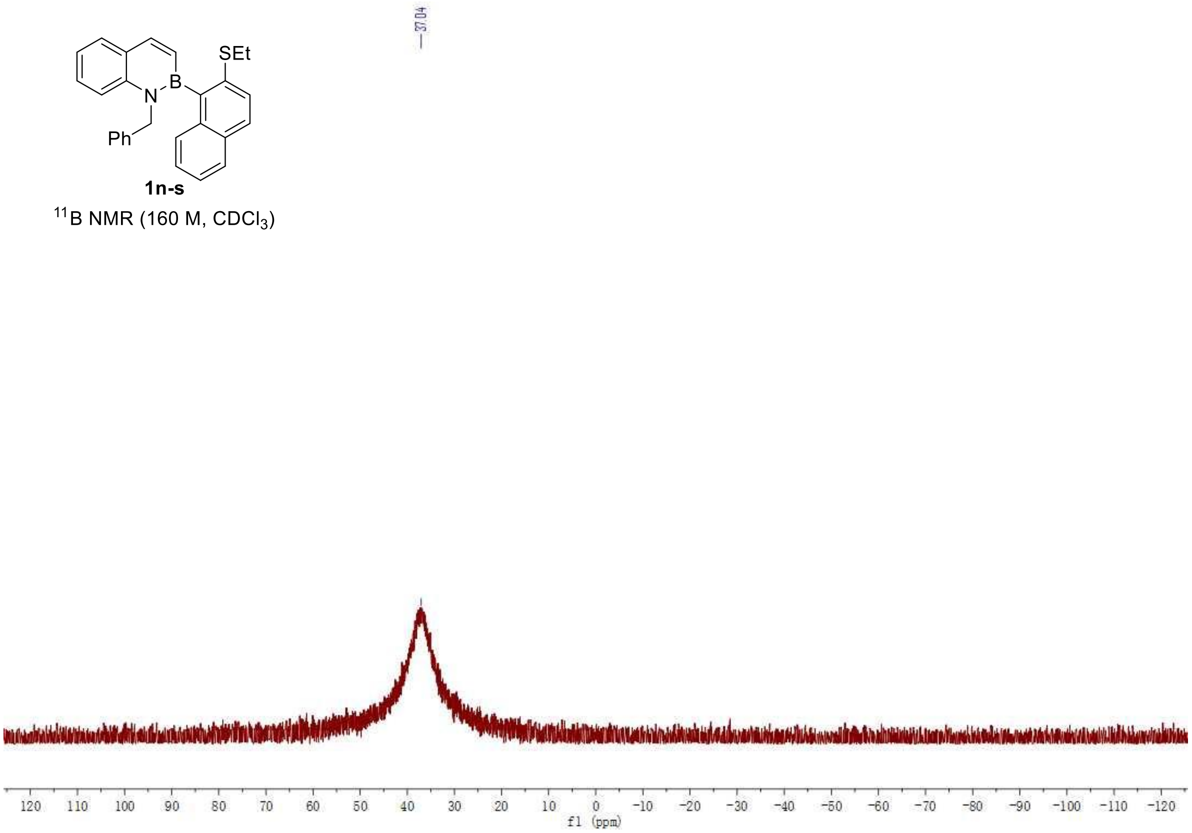

Supplementary Figure 54. ^{11}B NMR spectrum of 1n-s

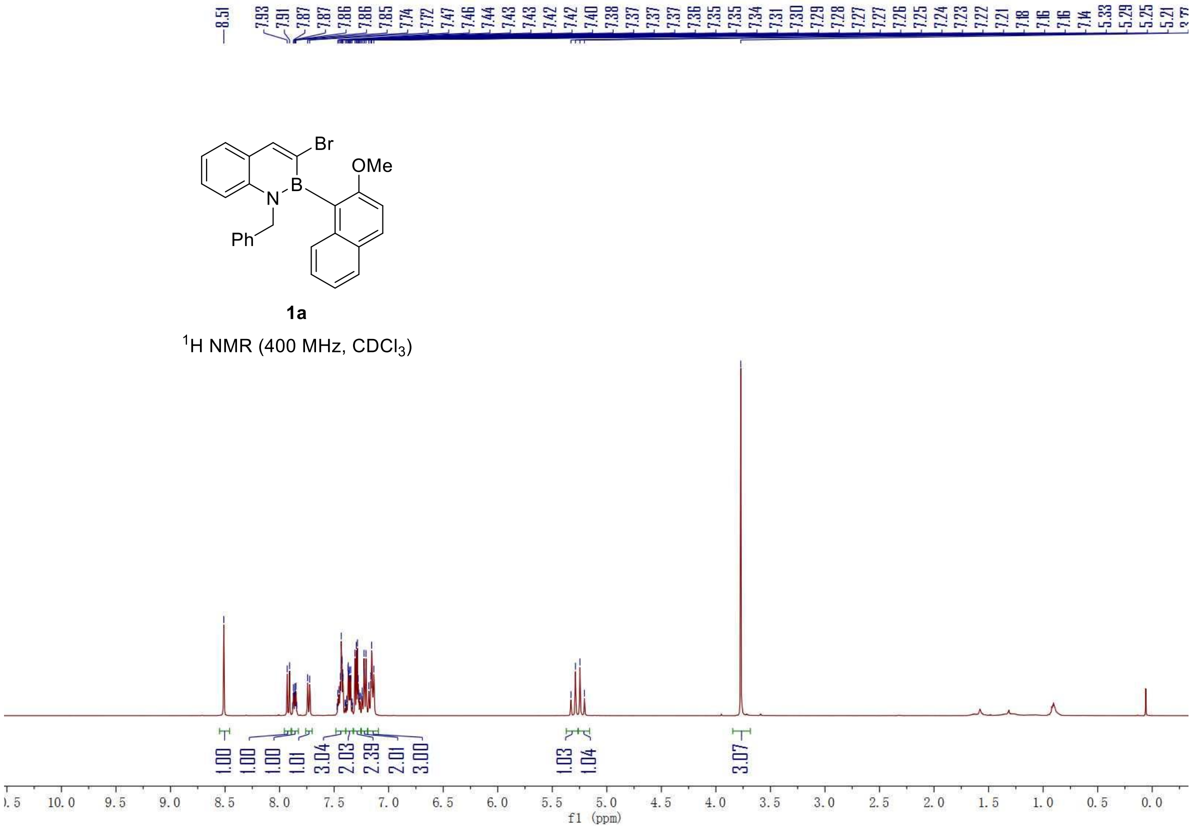

Supplementary Figure 55. ^{1}H NMR spectrum of 1a

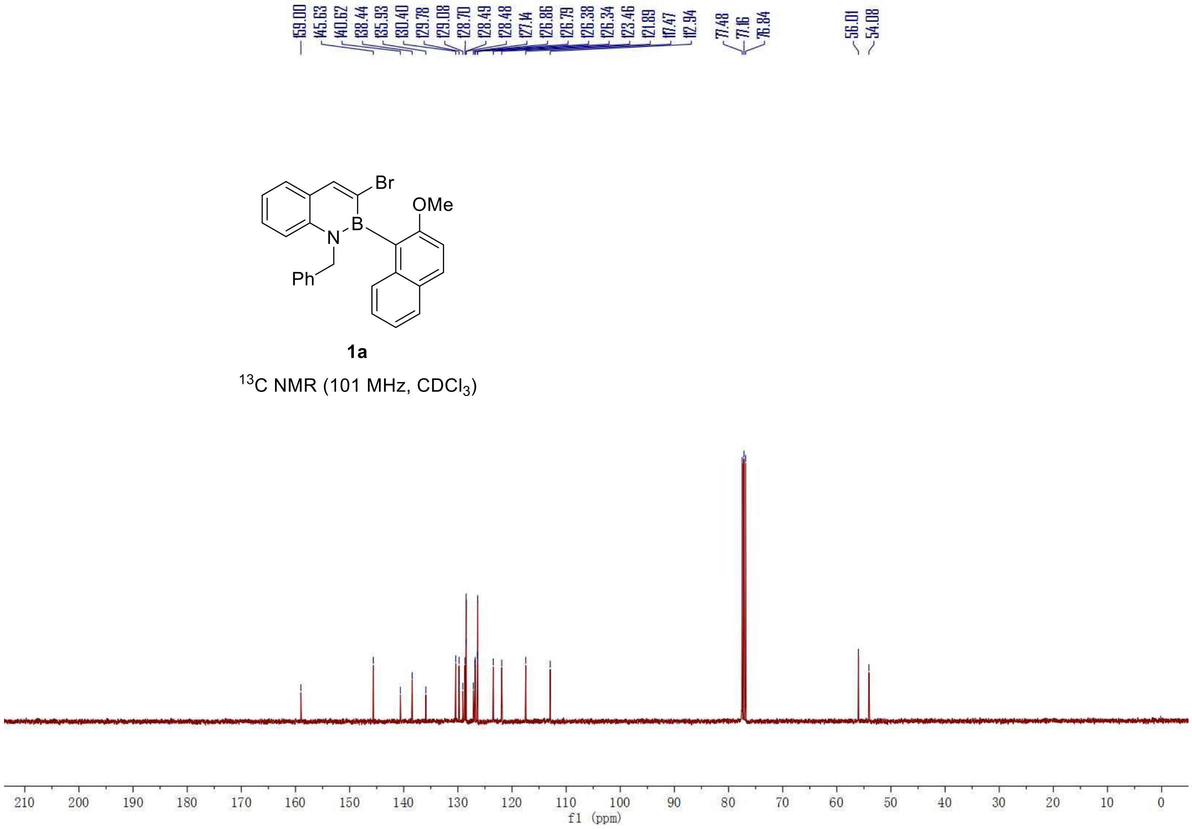

Supplementary Figure 56. ^{13}C NMR spectrum of 1a

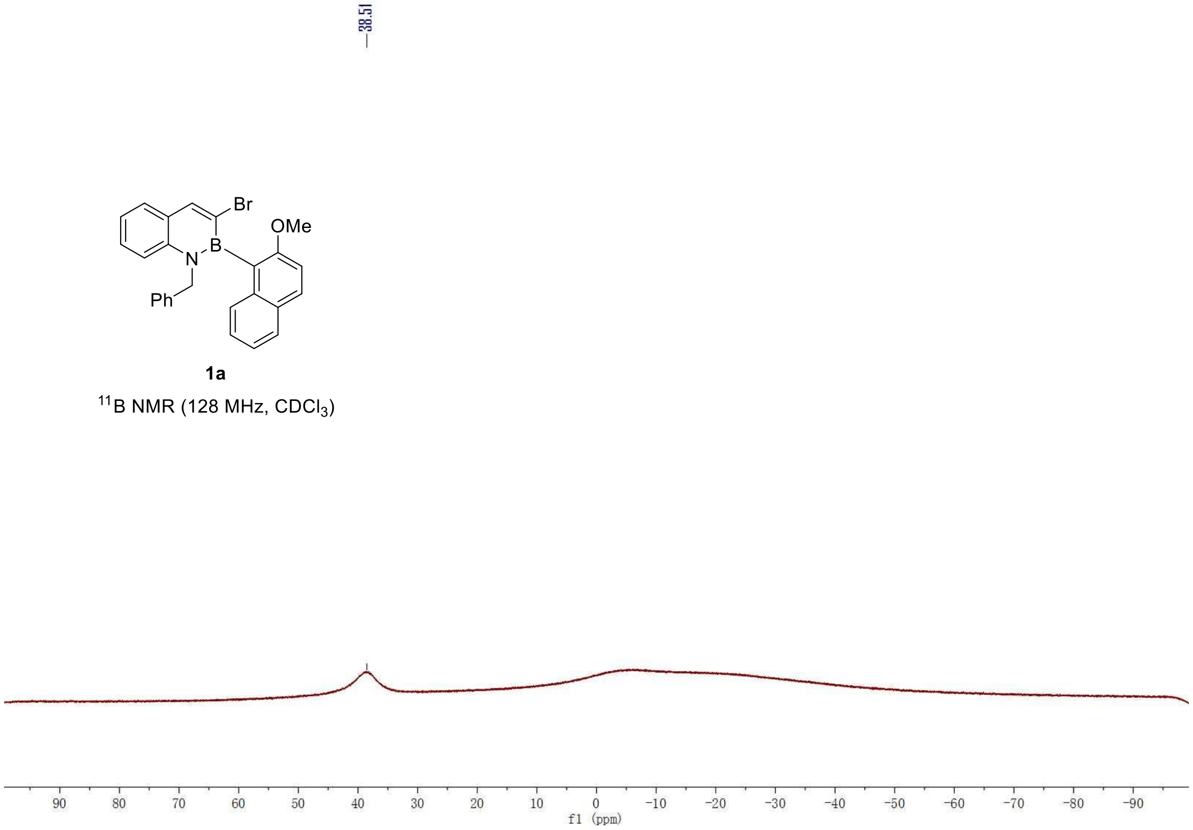

Supplementary Figure 57. ^{11}B NMR spectrum of 1a

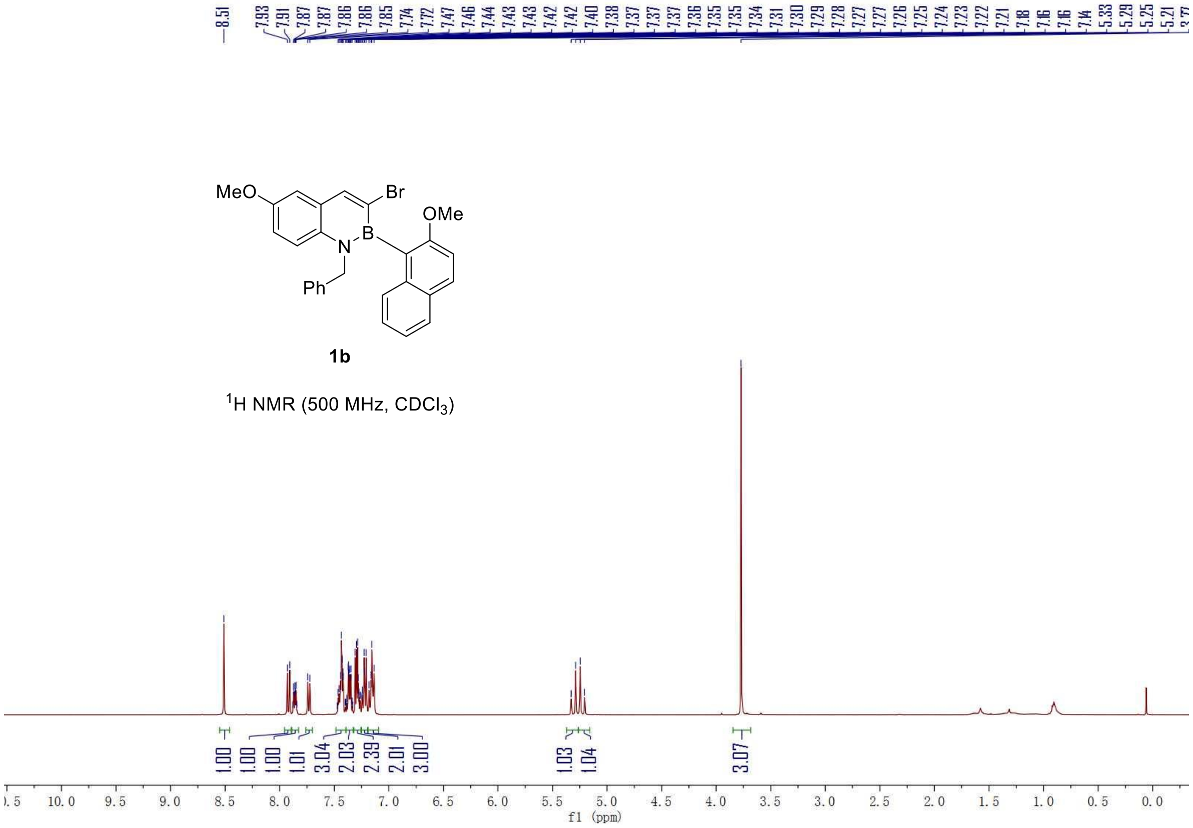

Supplementary Figure 58. ^{1}H NMR spectrum of 1b

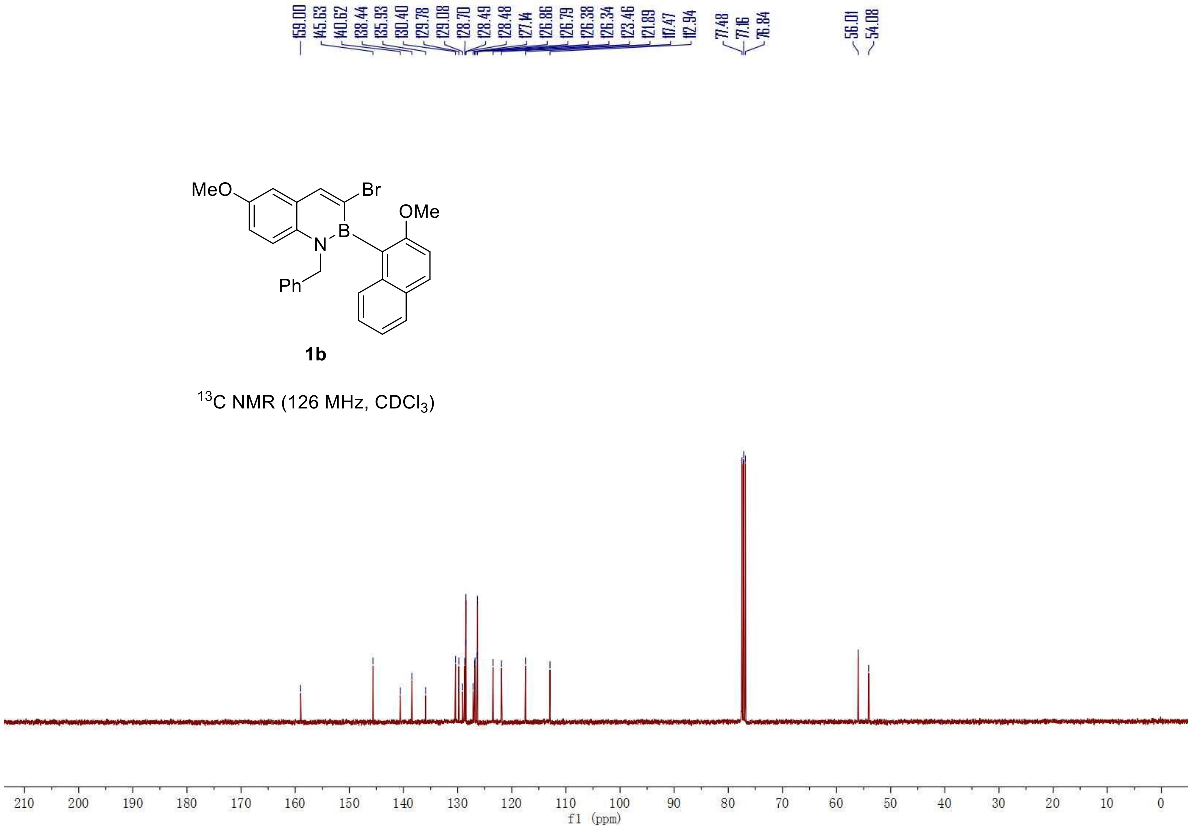

Supplementary Figure 59. ^{13}C NMR spectrum of 1b

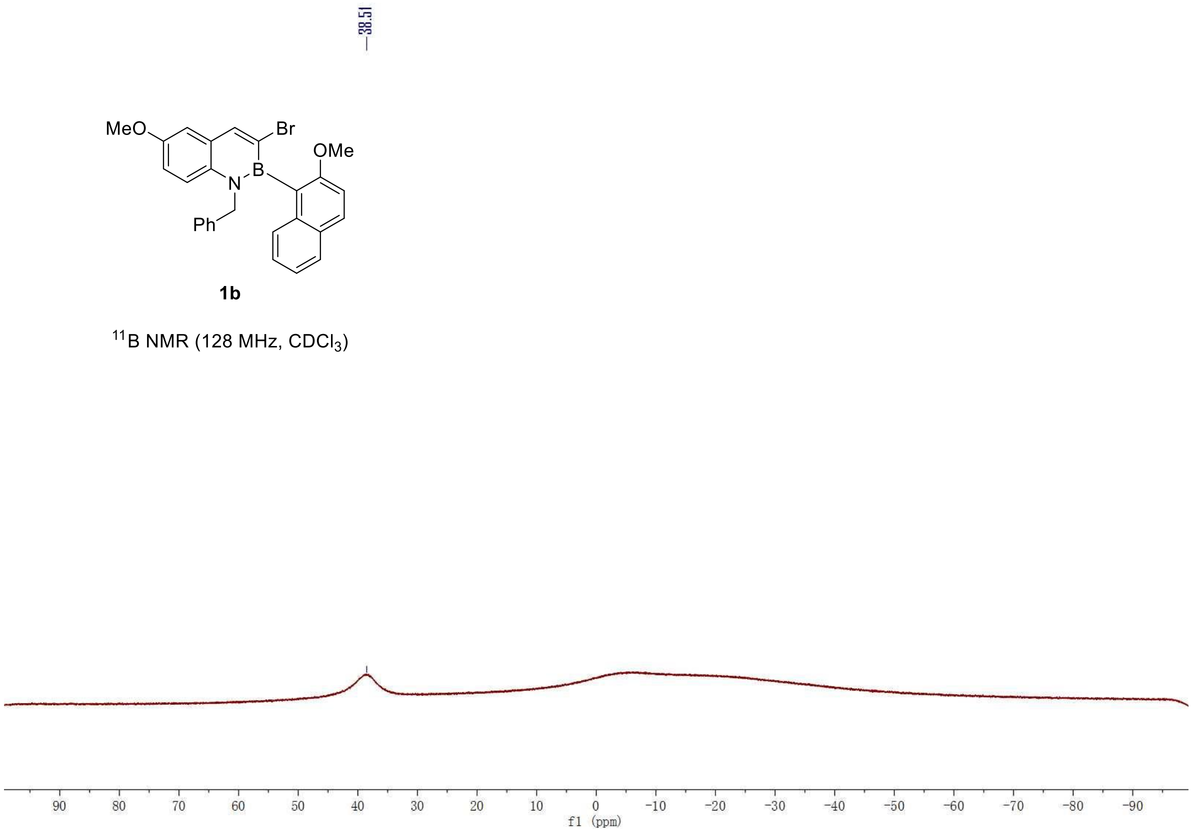

Supplementary Figure 60. ^{11}B NMR spectrum of 1b

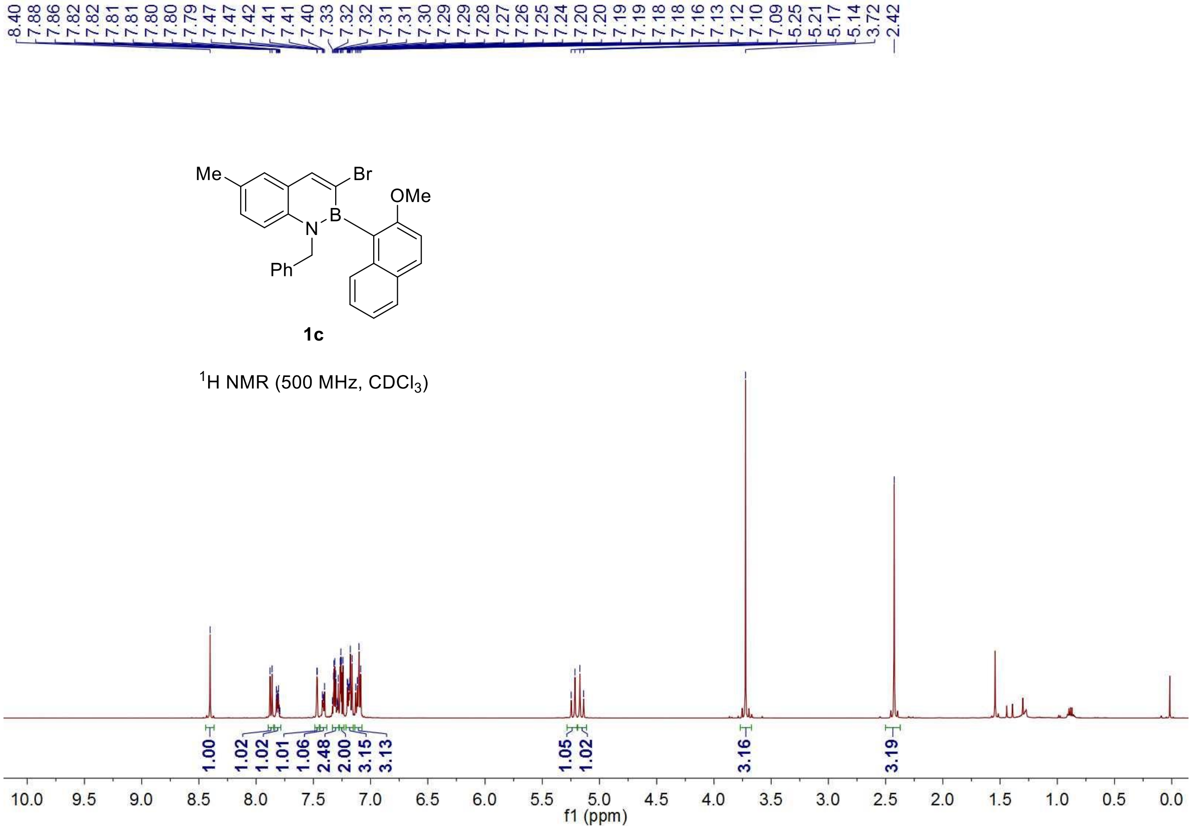

Supplementary Figure 61. ^{1}H NMR spectrum of 1c

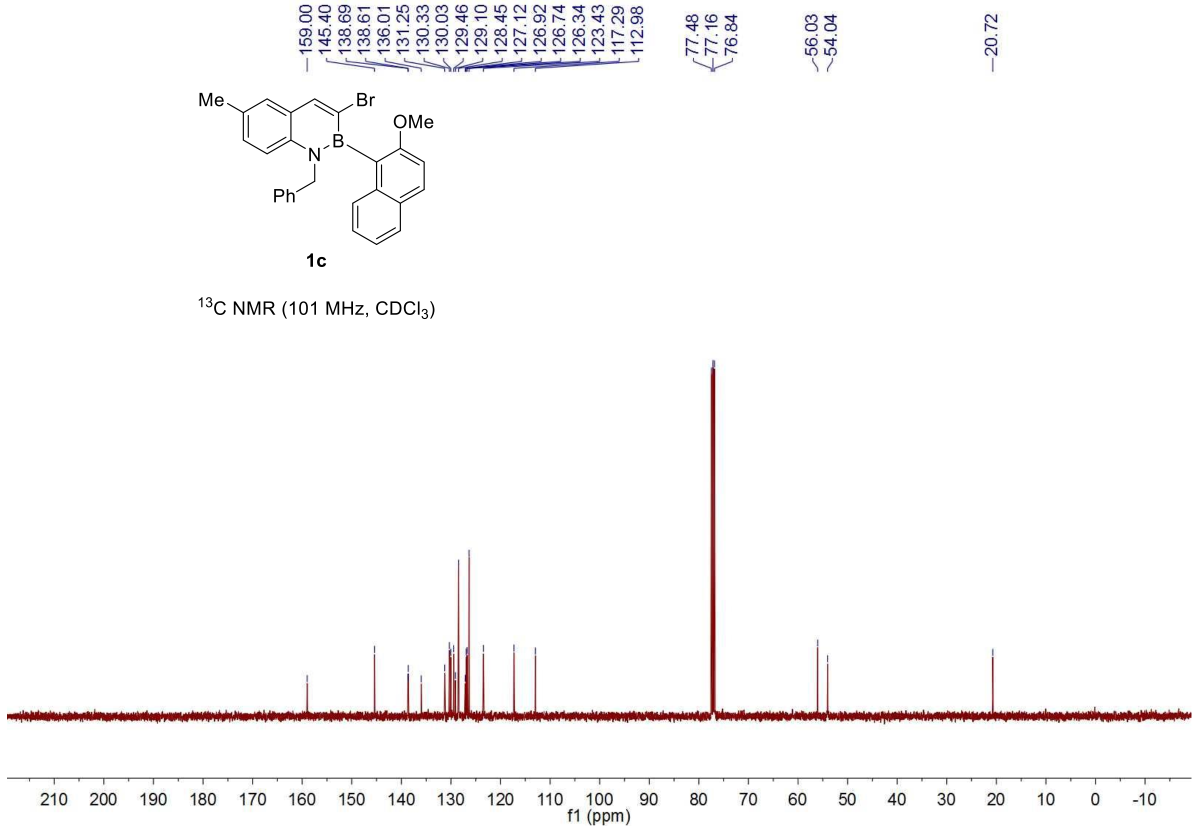

Supplementary Figure 62. ^{13}C NMR spectrum of 1c

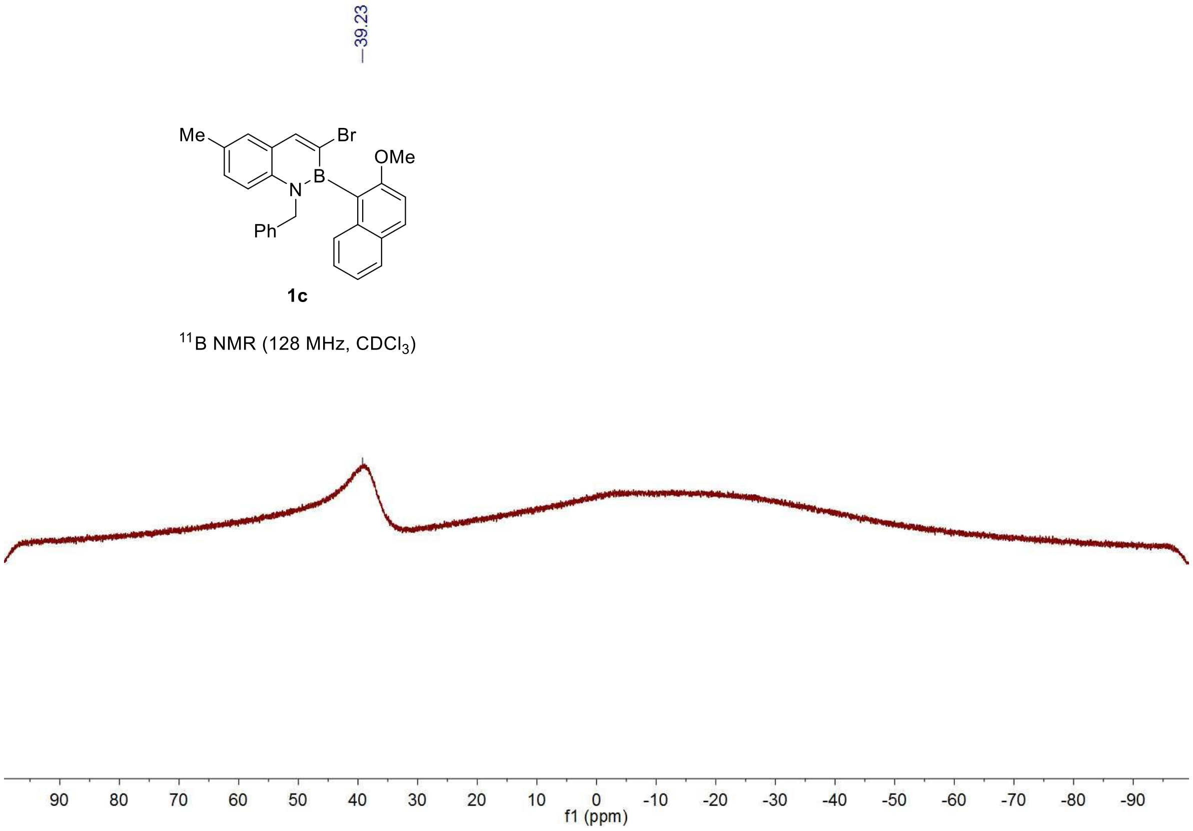

Supplementary Figure 63. ^{11}B NMR spectrum of 1c

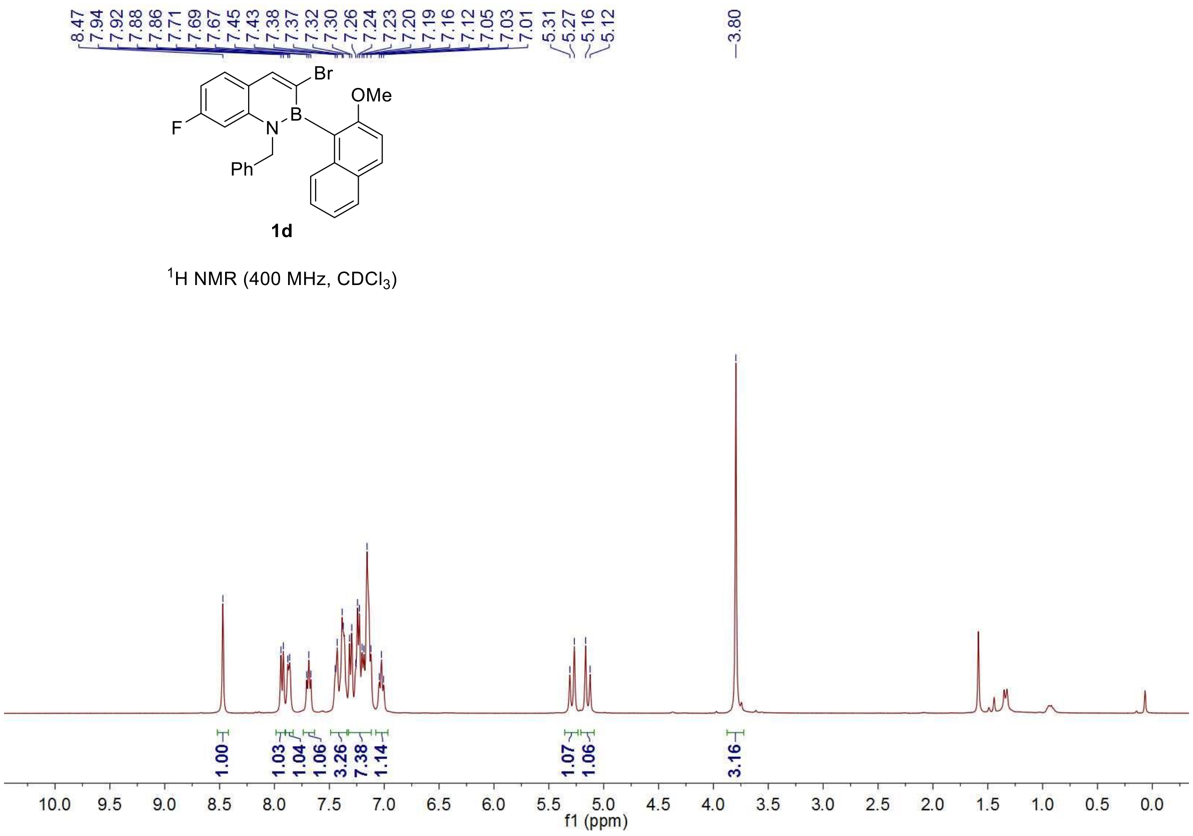

Supplementary Figure 64. ^{1}H NMR spectrum of 1d

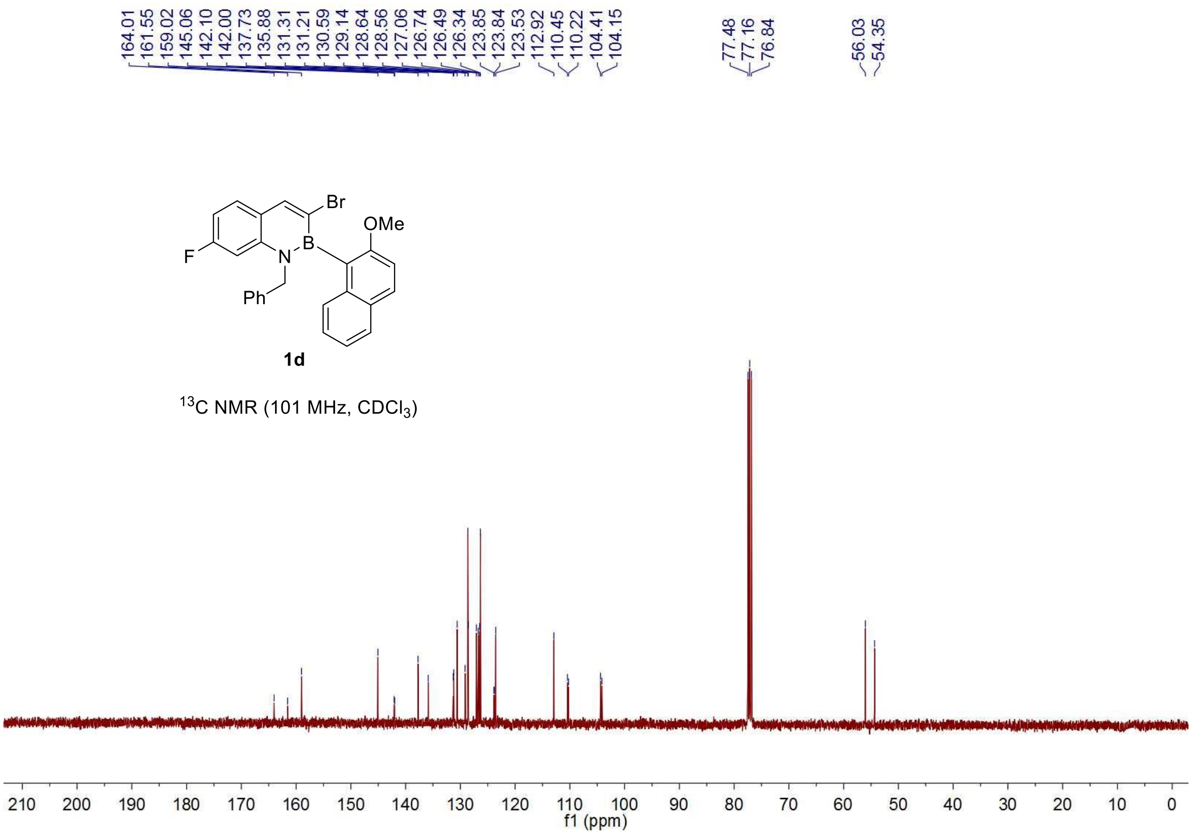

Supplementary Figure 65. ^{13}C NMR spectrum of 1d

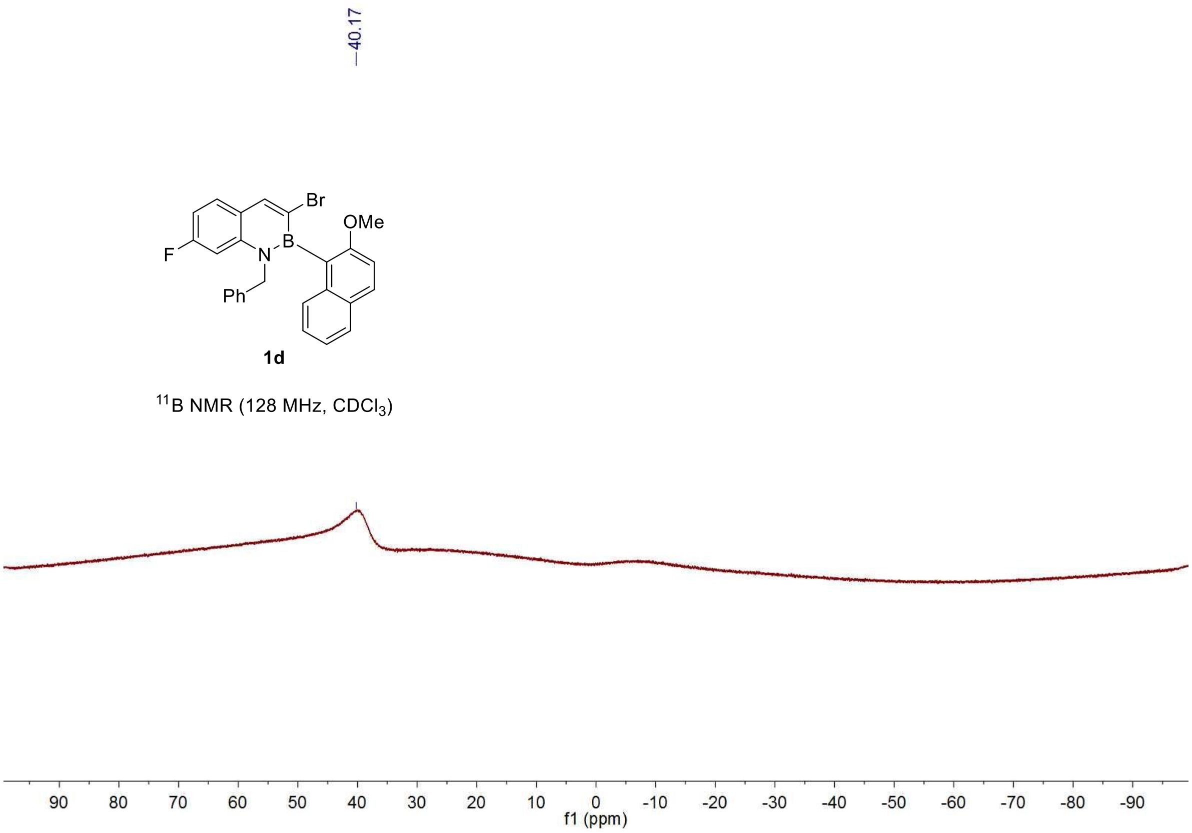

Supplementary Figure 66. ^{11}B NMR spectrum of 1d

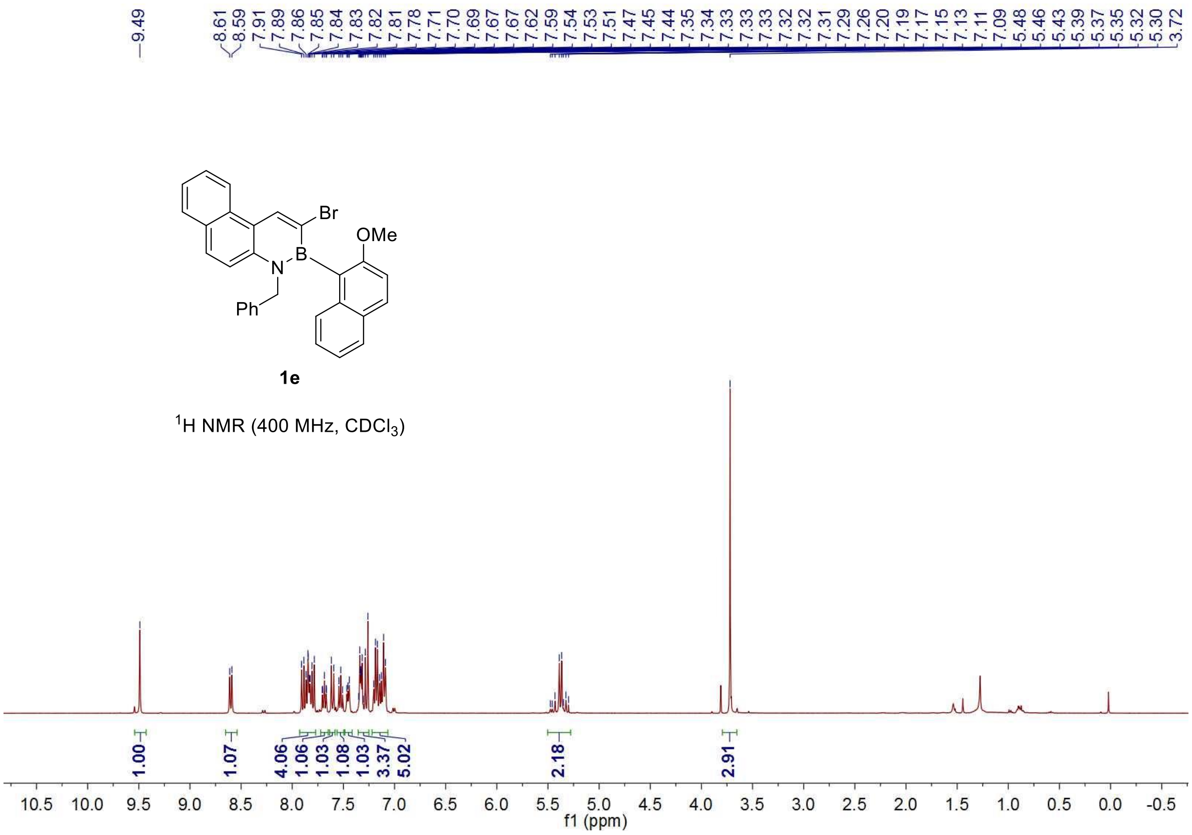

Supplementary Figure 67. ^{1}H NMR spectrum of 1e

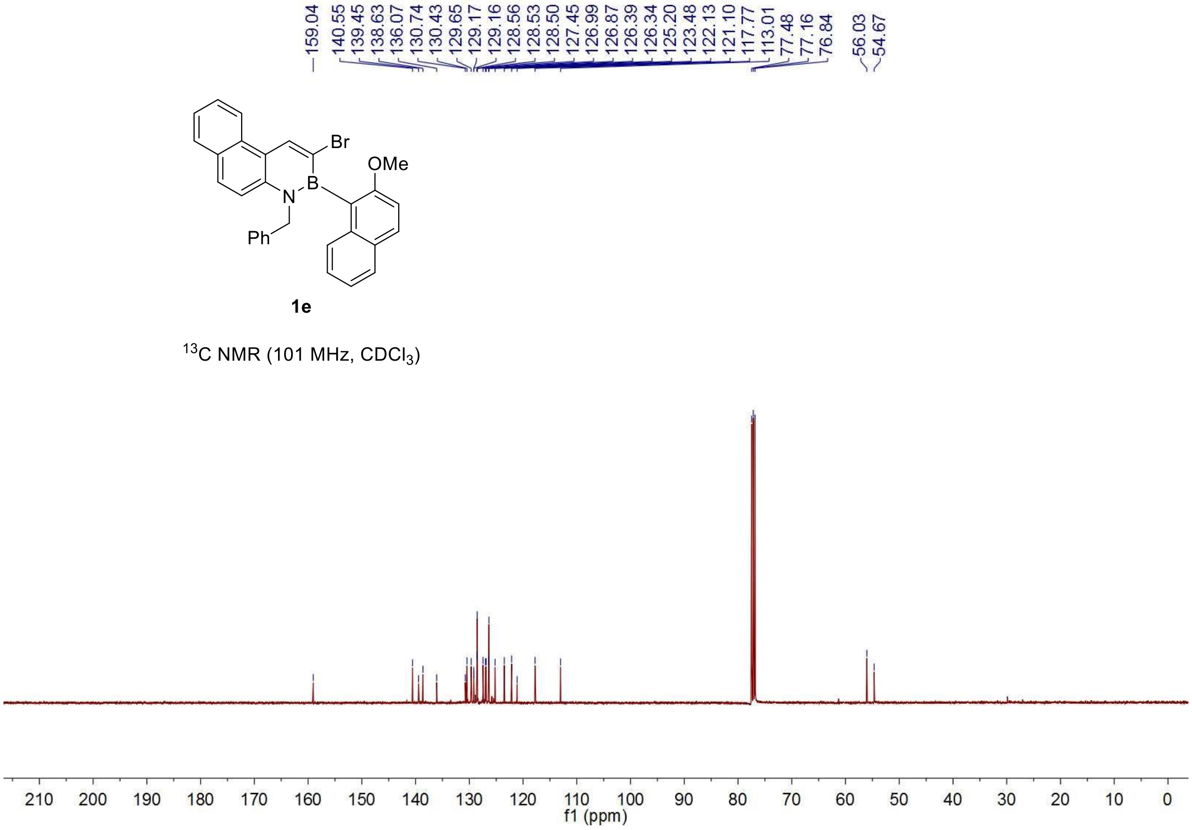

Supplementary Figure 68. ^{13}C NMR spectrum of 1e

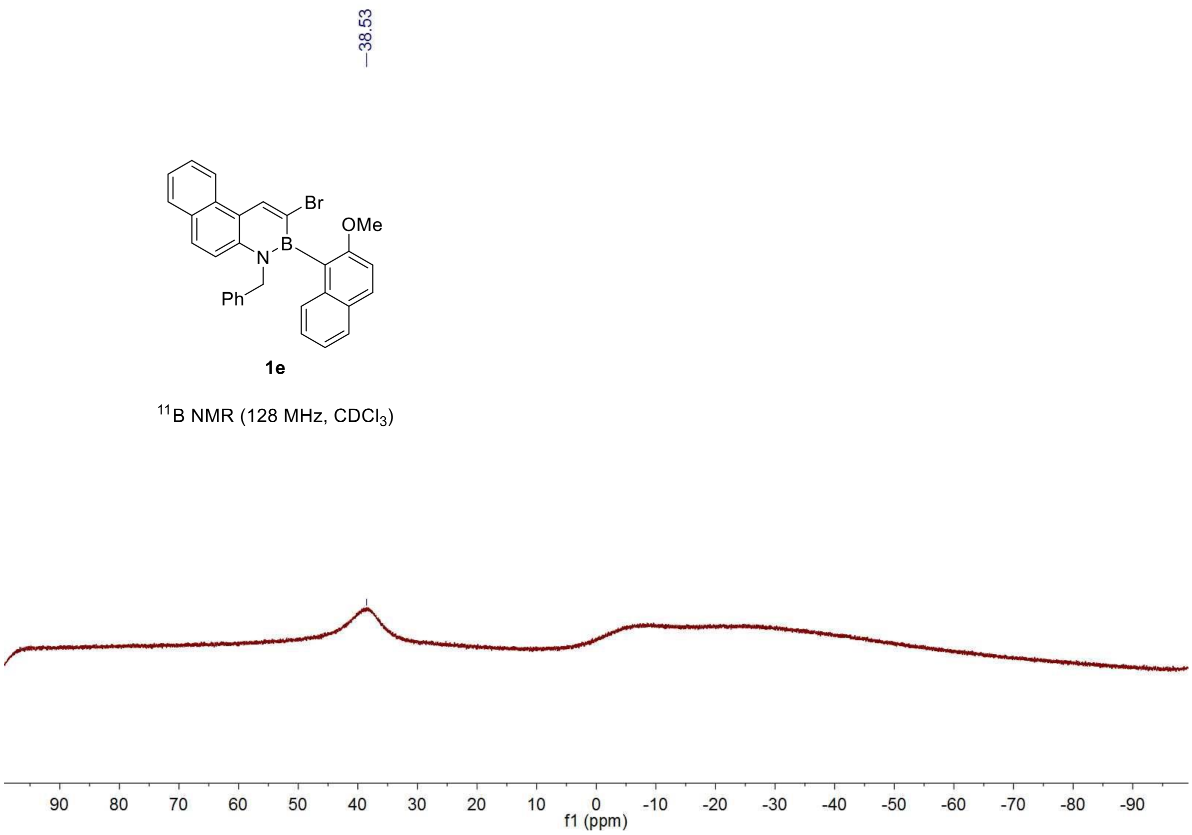

Supplementary Figure 69. ^{11}B NMR spectrum of 1e

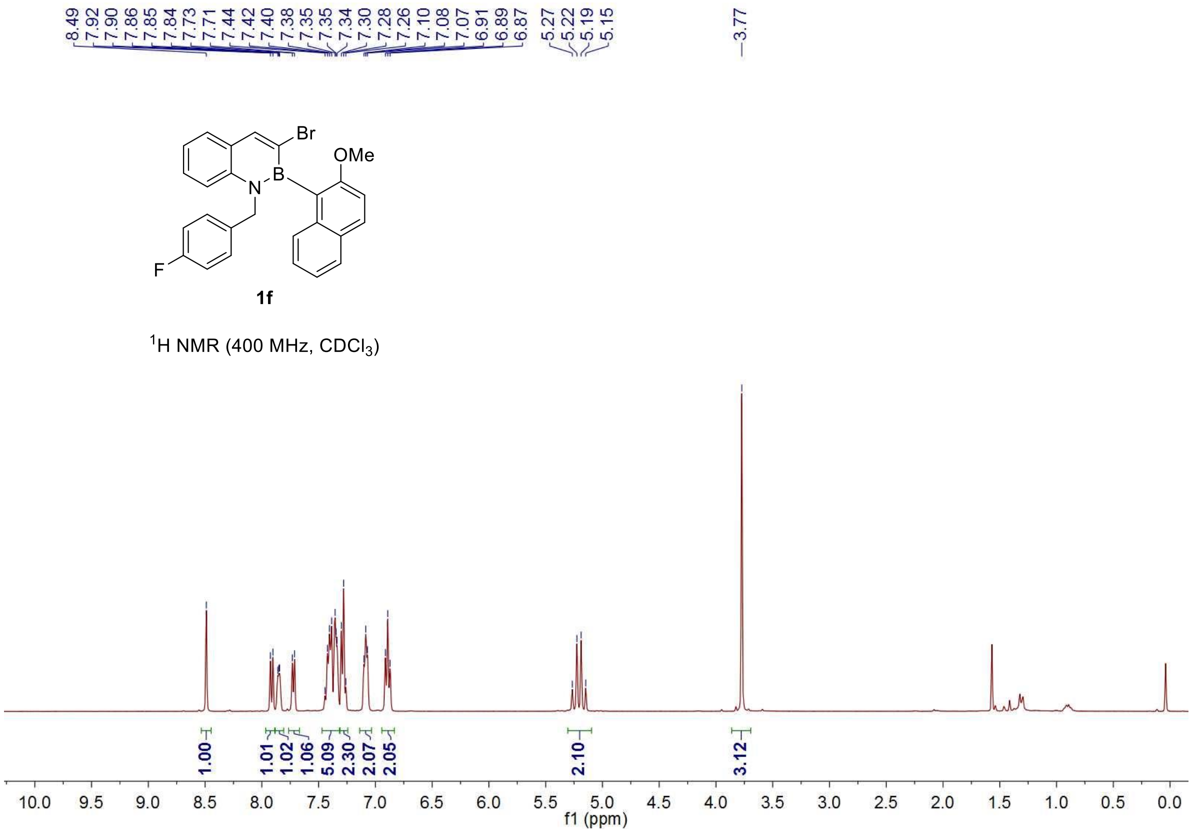

Supplementary Figure 70. ^{1}H NMR spectrum of 1f

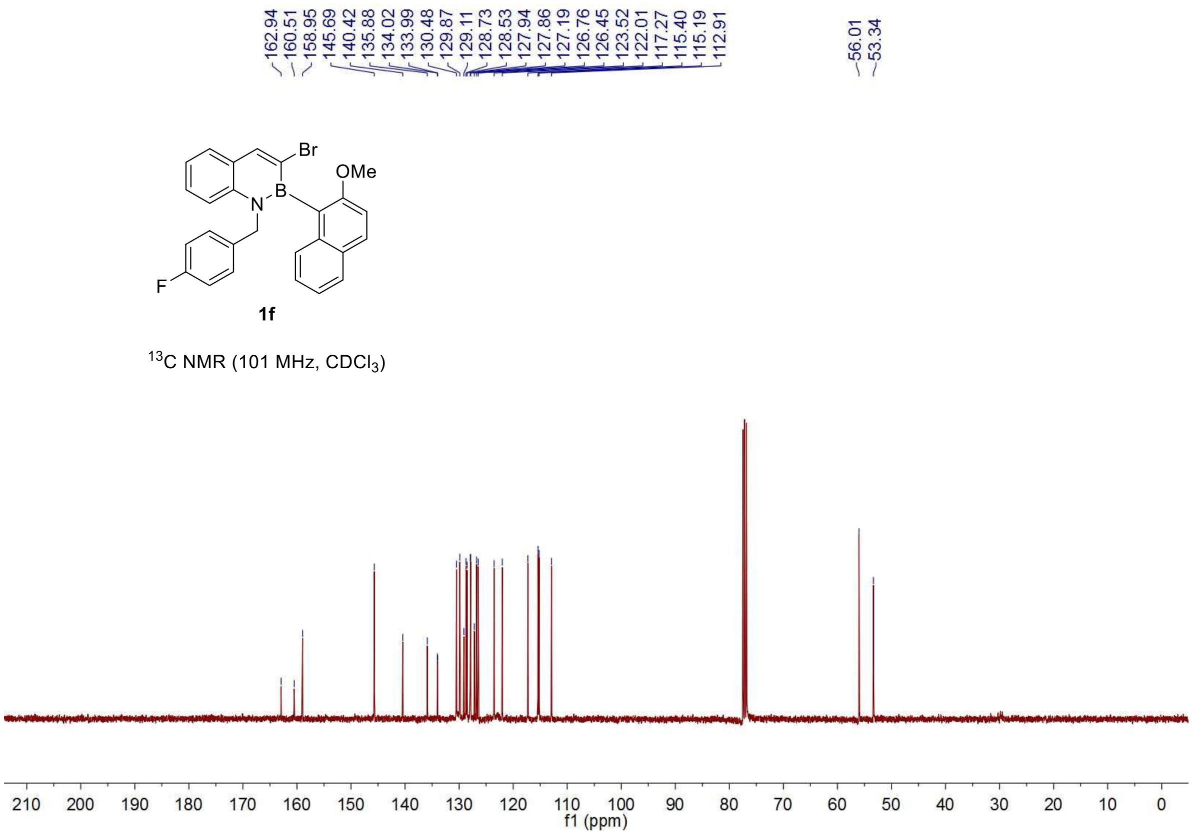

Supplementary Figure 71. ^{13}C NMR spectrum of 1f

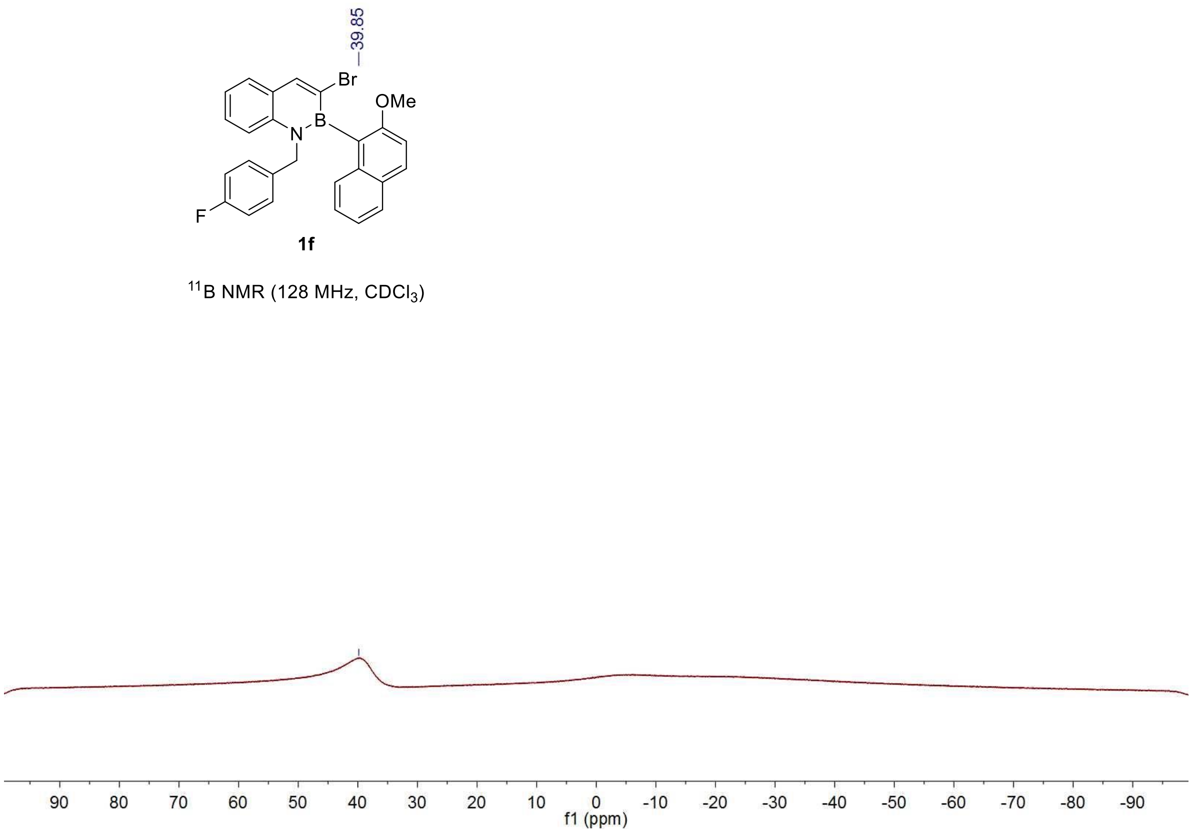

Supplementary Figure 72. ^{11}B NMR spectrum of 1f

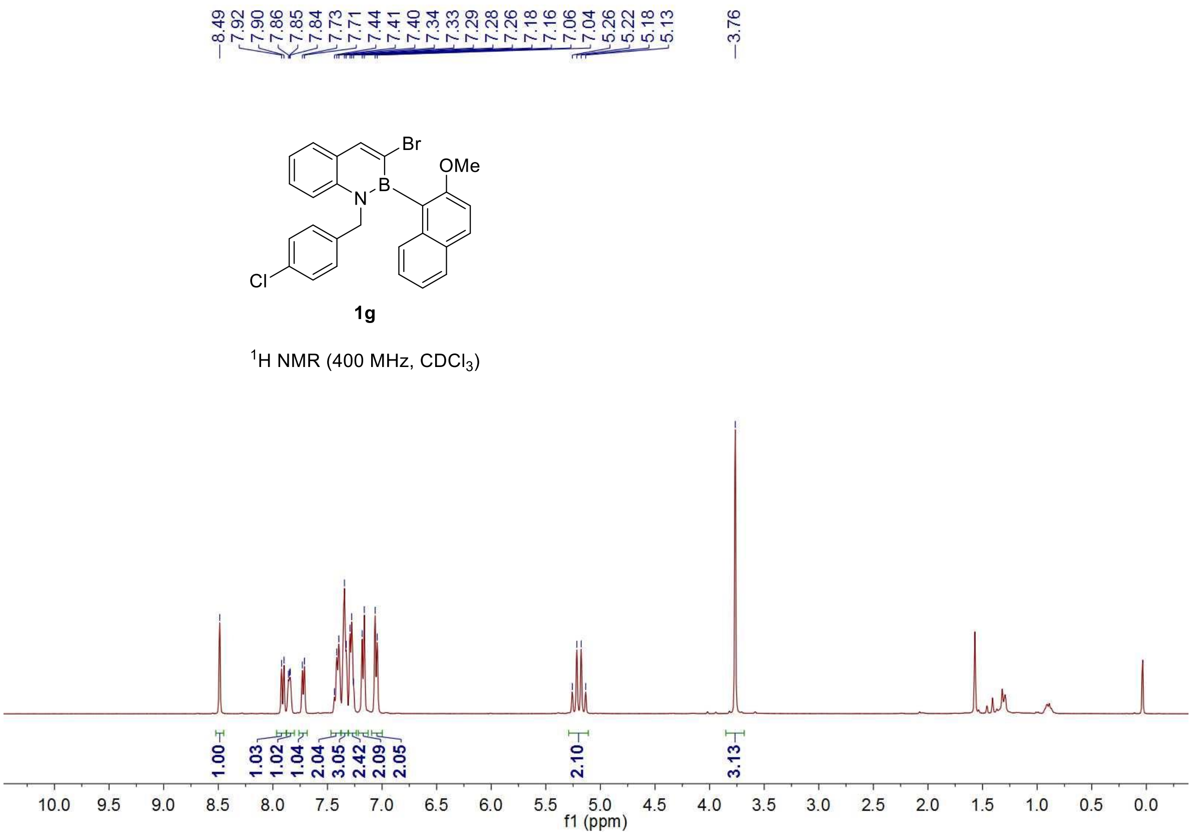

Supplementary Figure 73. ^{1}H NMR spectrum of 1g

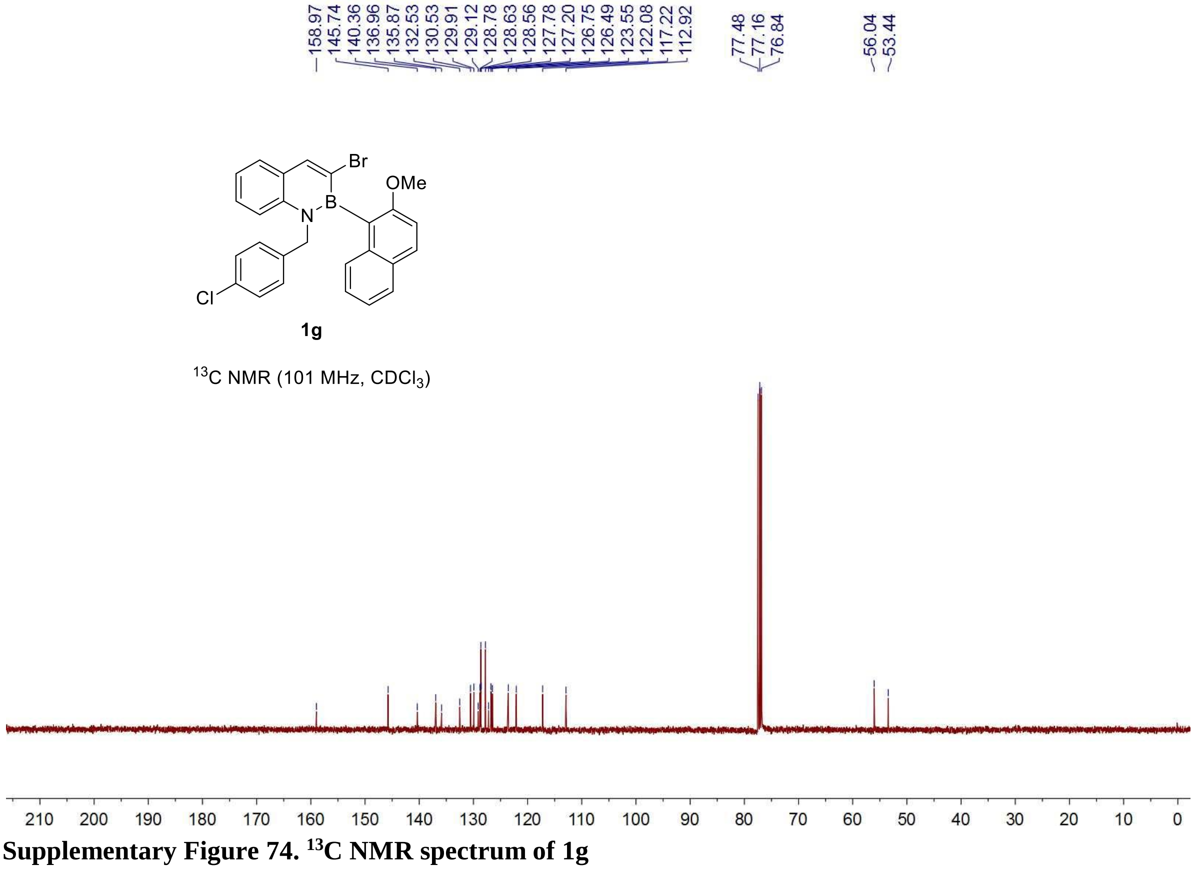

Supplementary Figure 74. ^{13}C NMR spectrum of 1g

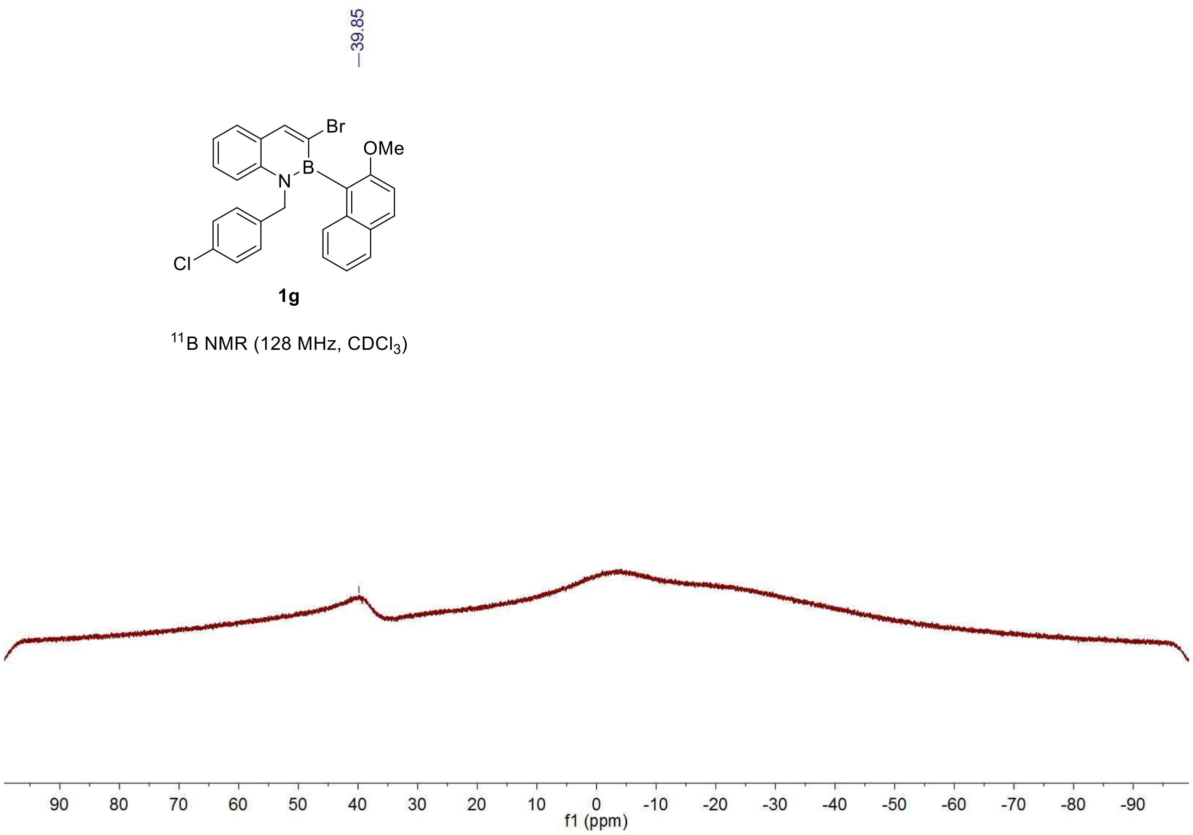

Supplementary Figure 75. ^{11}B NMR spectrum of 1g

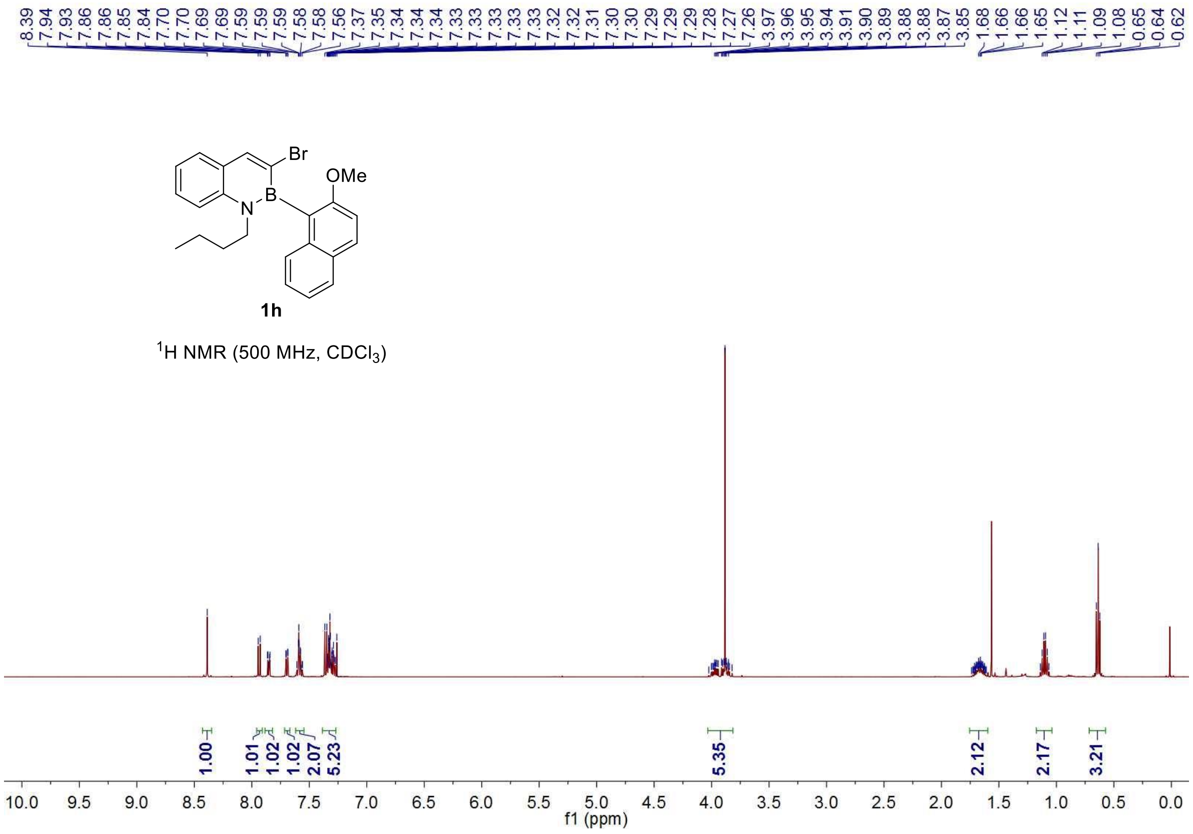

Supplementary Figure 76. ^{1}H NMR spectrum of 1h

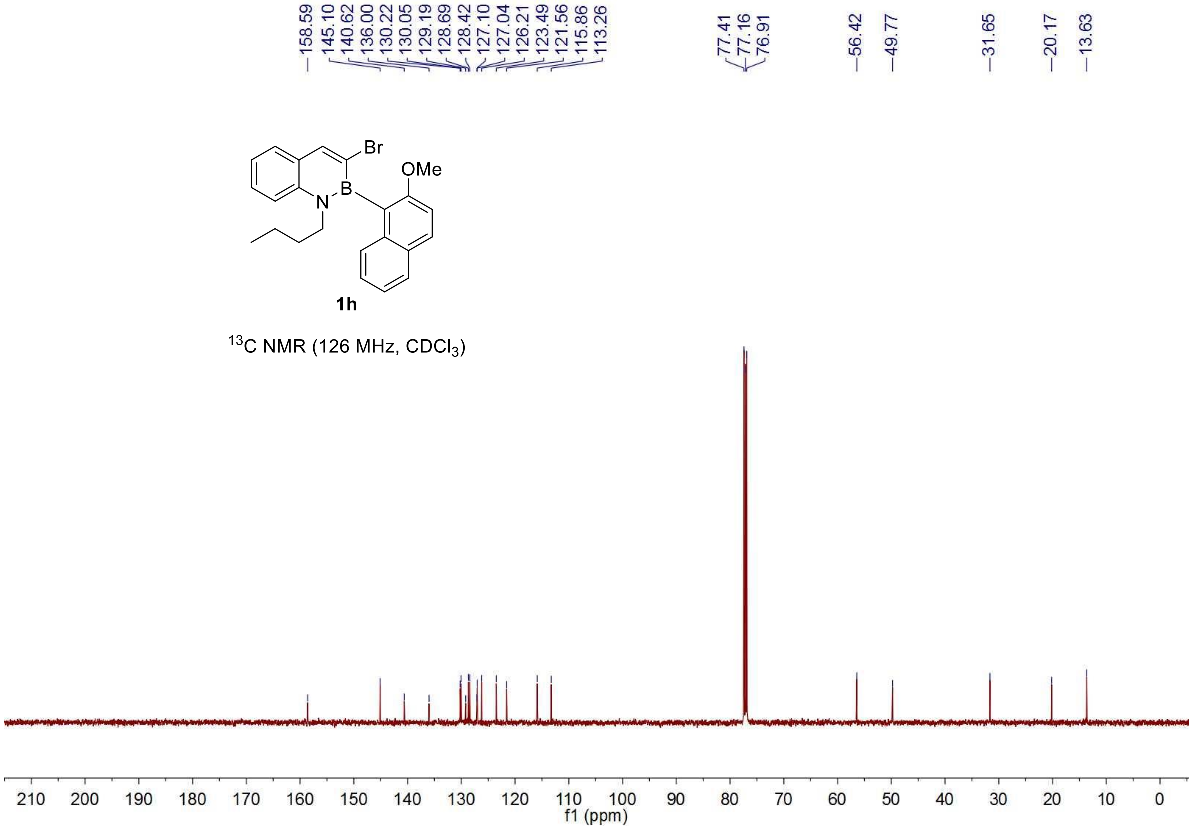

Supplementary Figure 77. ^{13}C NMR spectrum of 1h

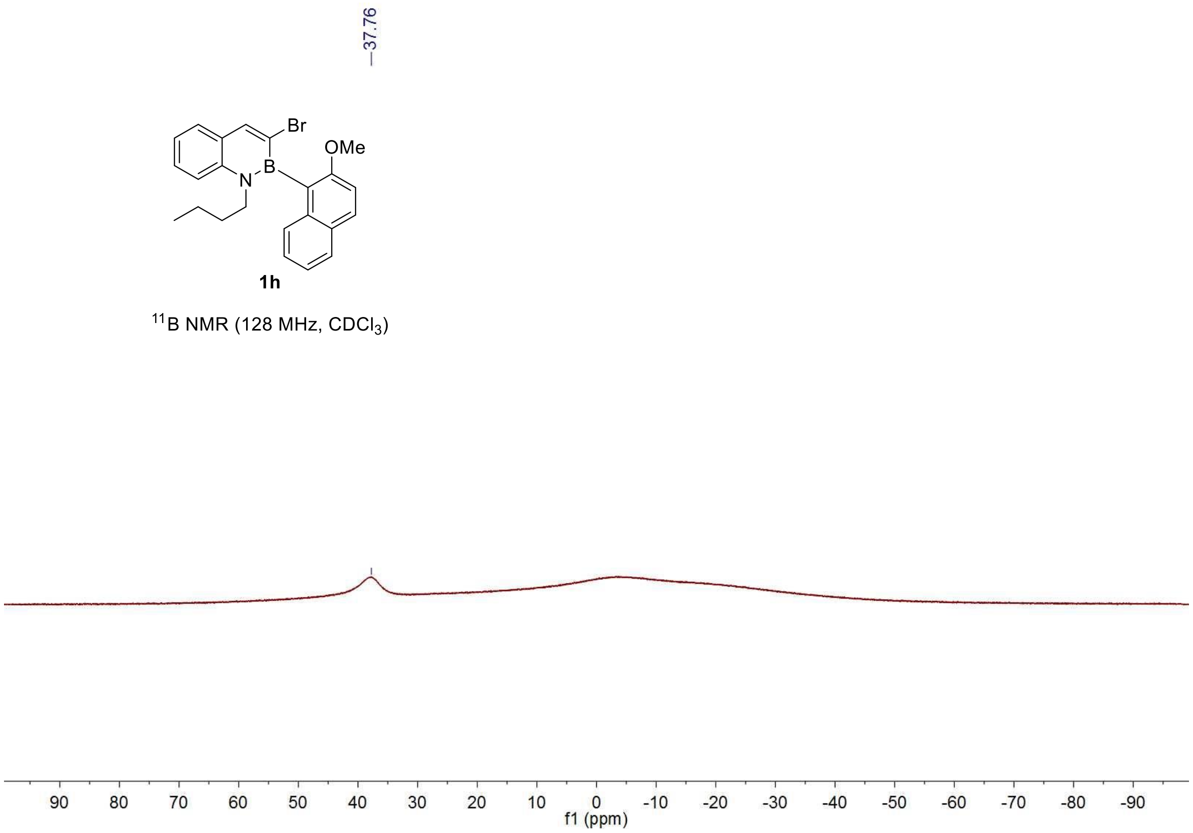

Supplementary Figure 78. ^{11}B NMR spectrum of 1h

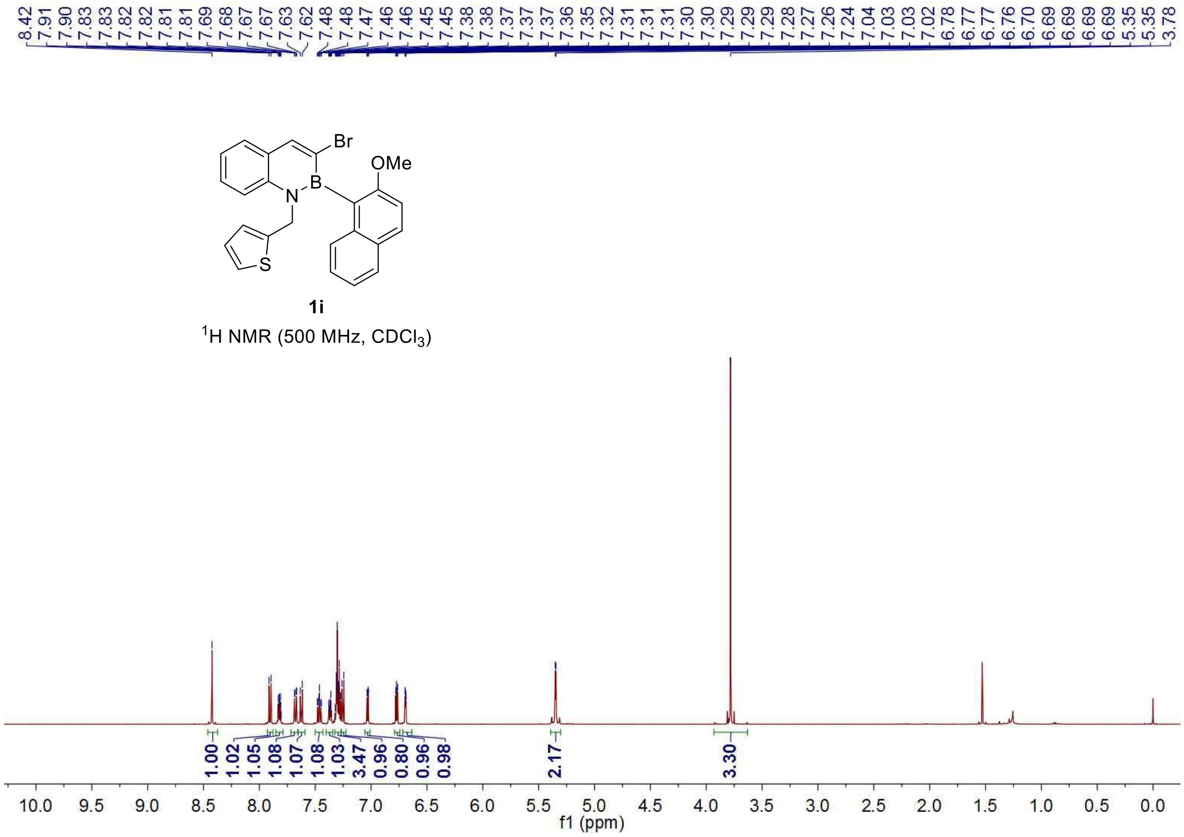

Supplementary Figure 79. ^{1}H NMR spectrum of 1i

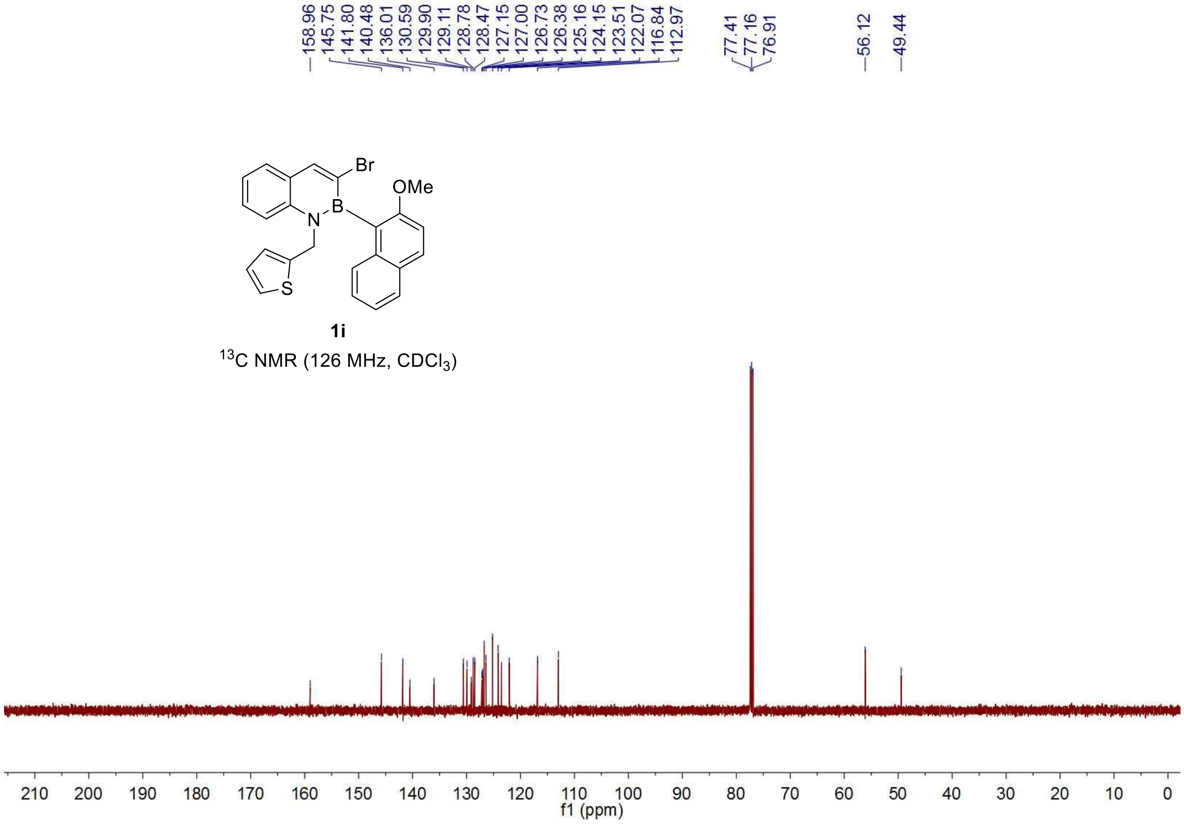

Supplementary Figure 80. ^{13}C NMR spectrum of 1i

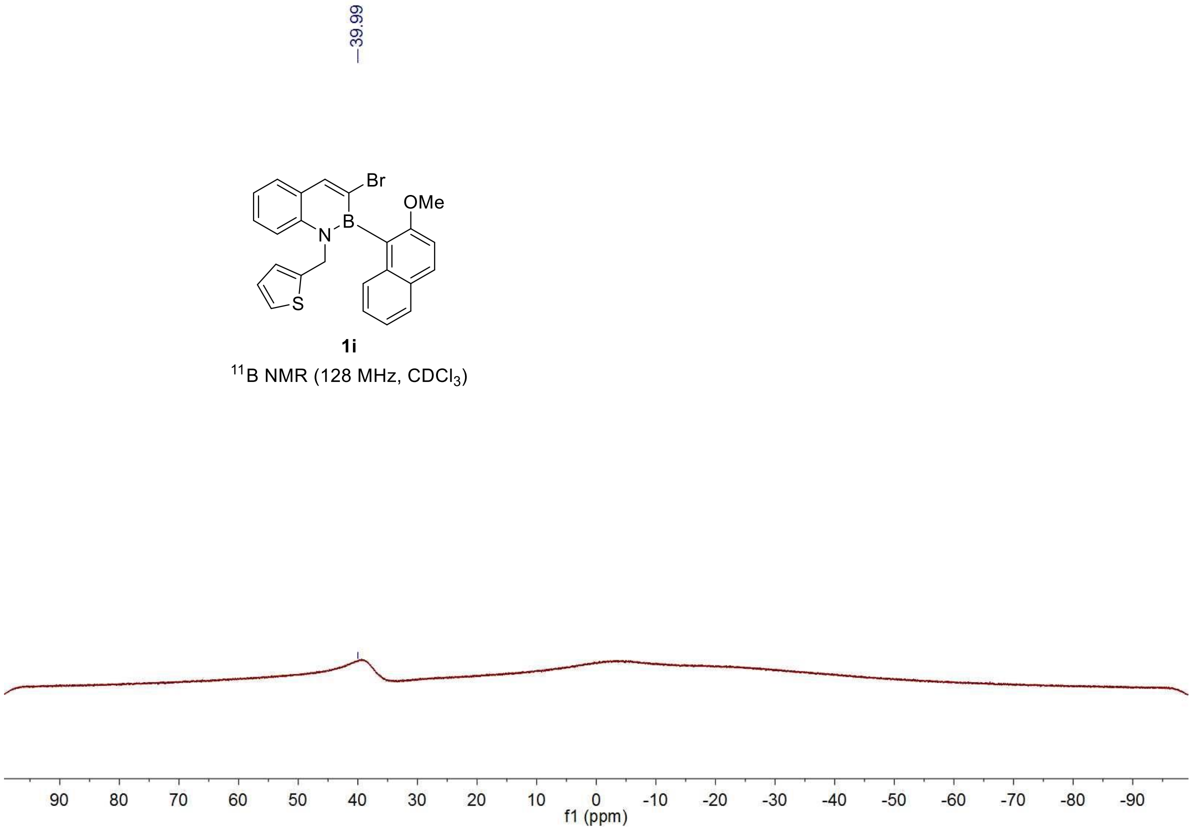

Supplementary Figure 81. ^{11}B NMR spectrum of 1i

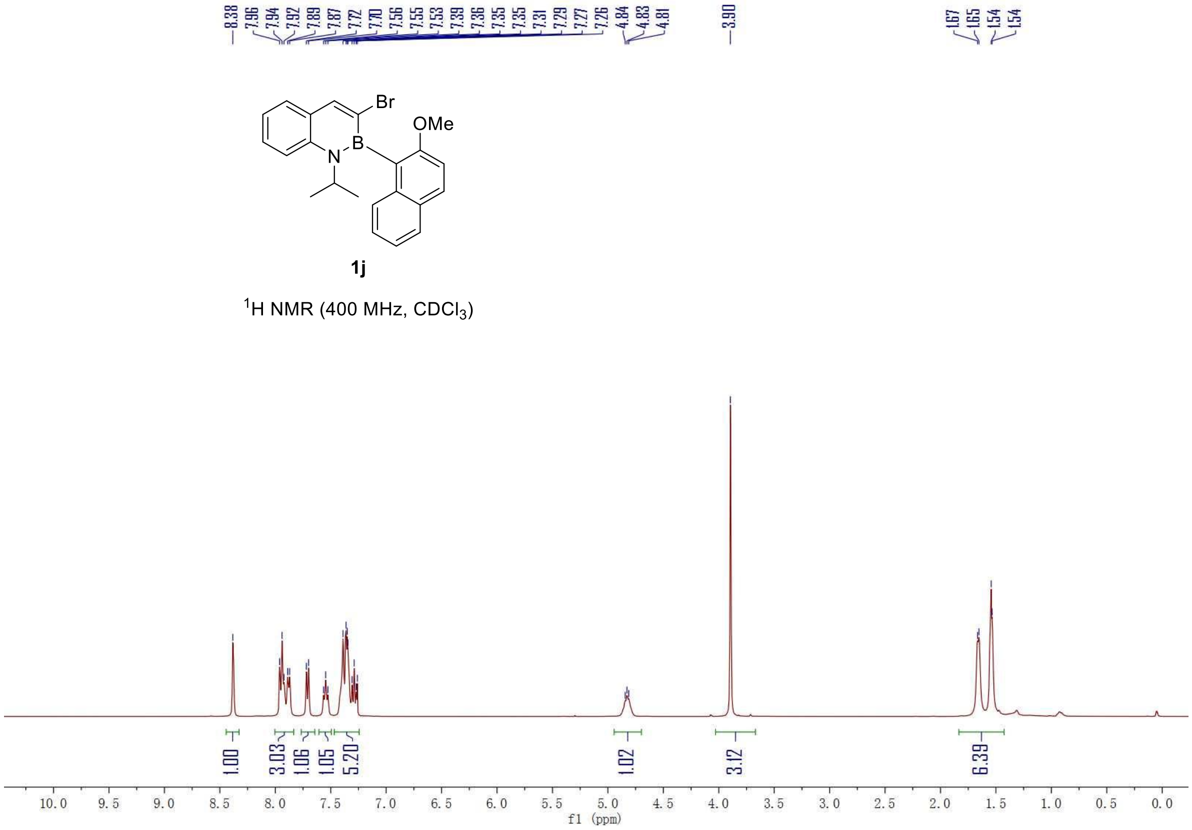

Supplementary Figure 82. ^{1}H NMR spectrum of 1j

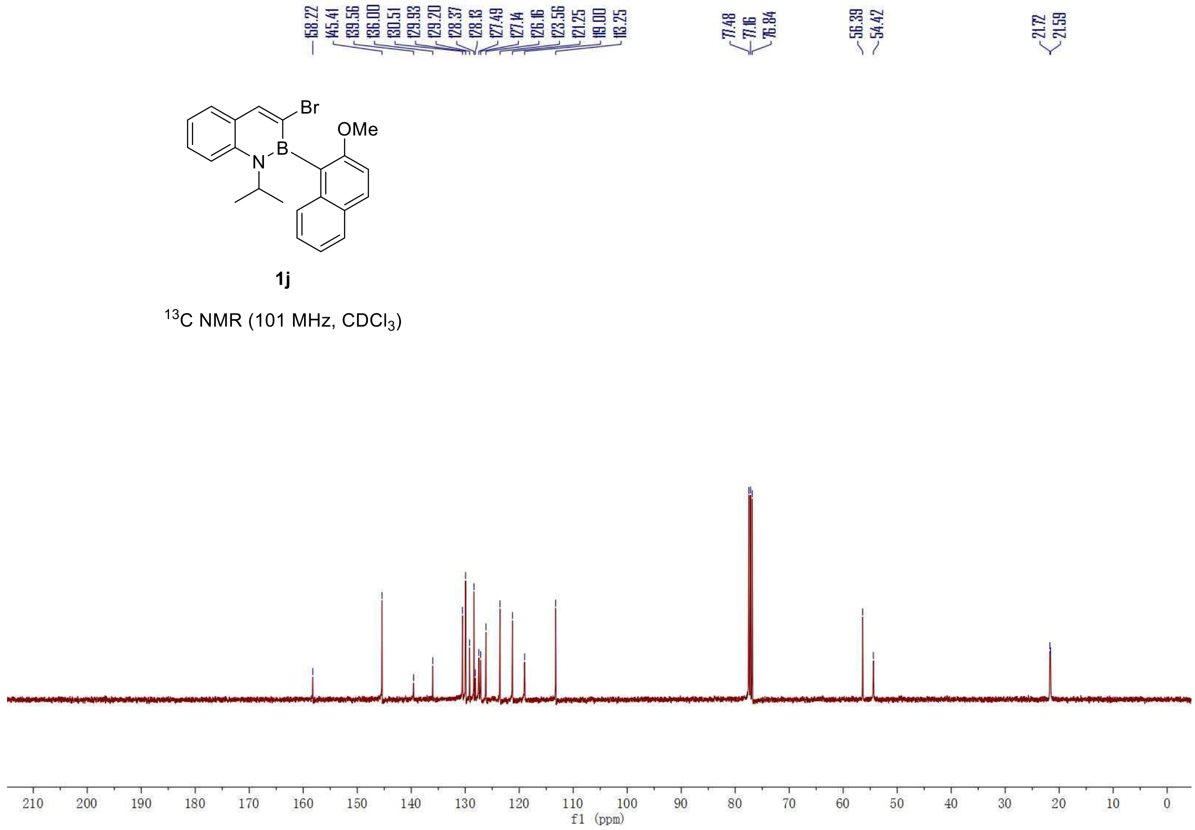

Supplementary Figure 83. ^{13}C NMR spectrum of 1j

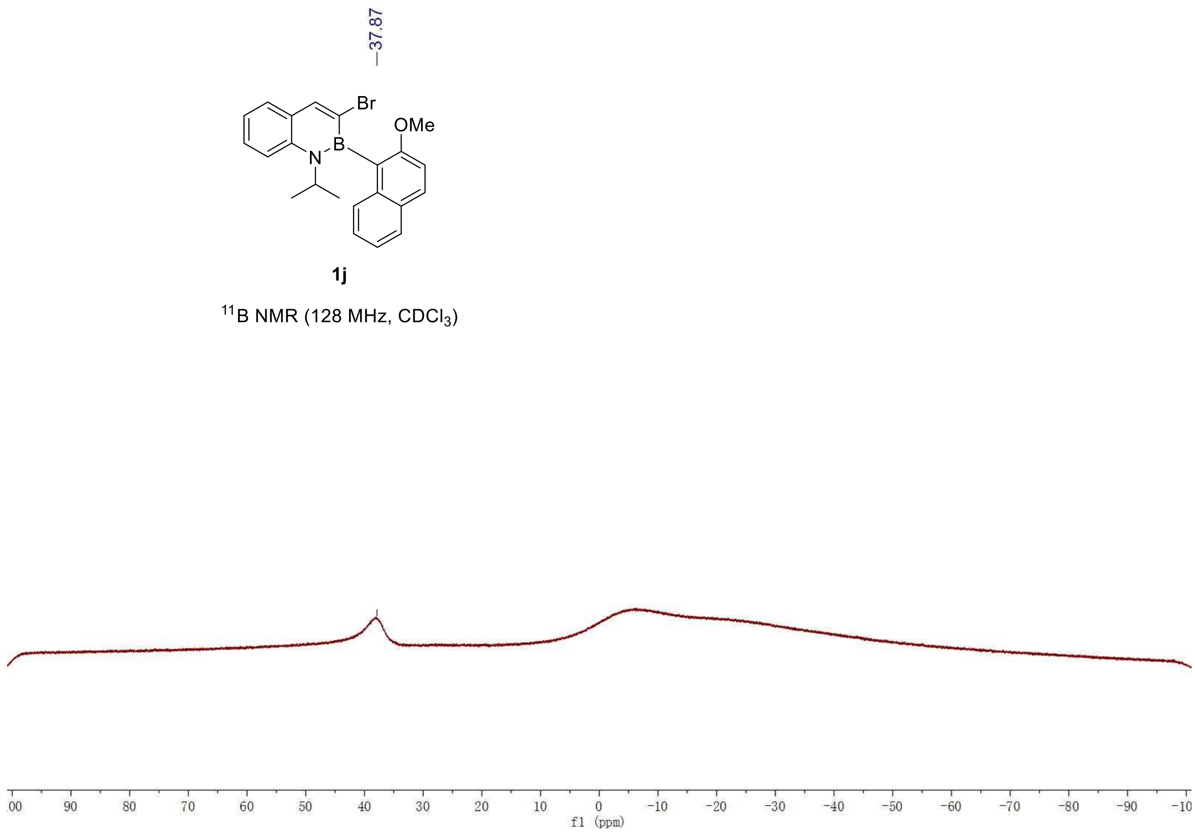

Supplementary Figure 84. ^{11}B NMR spectrum of 1j

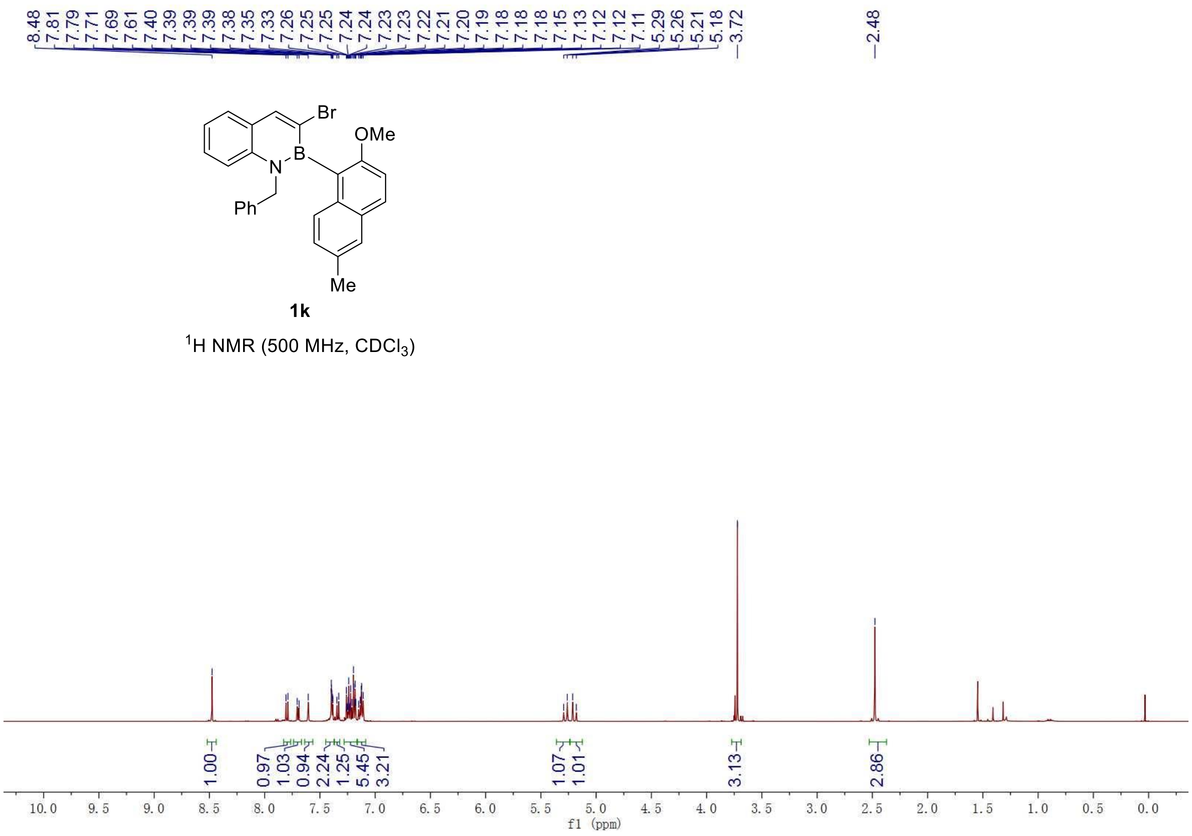

Supplementary Figure 85. ^{1}H NMR spectrum of 1k

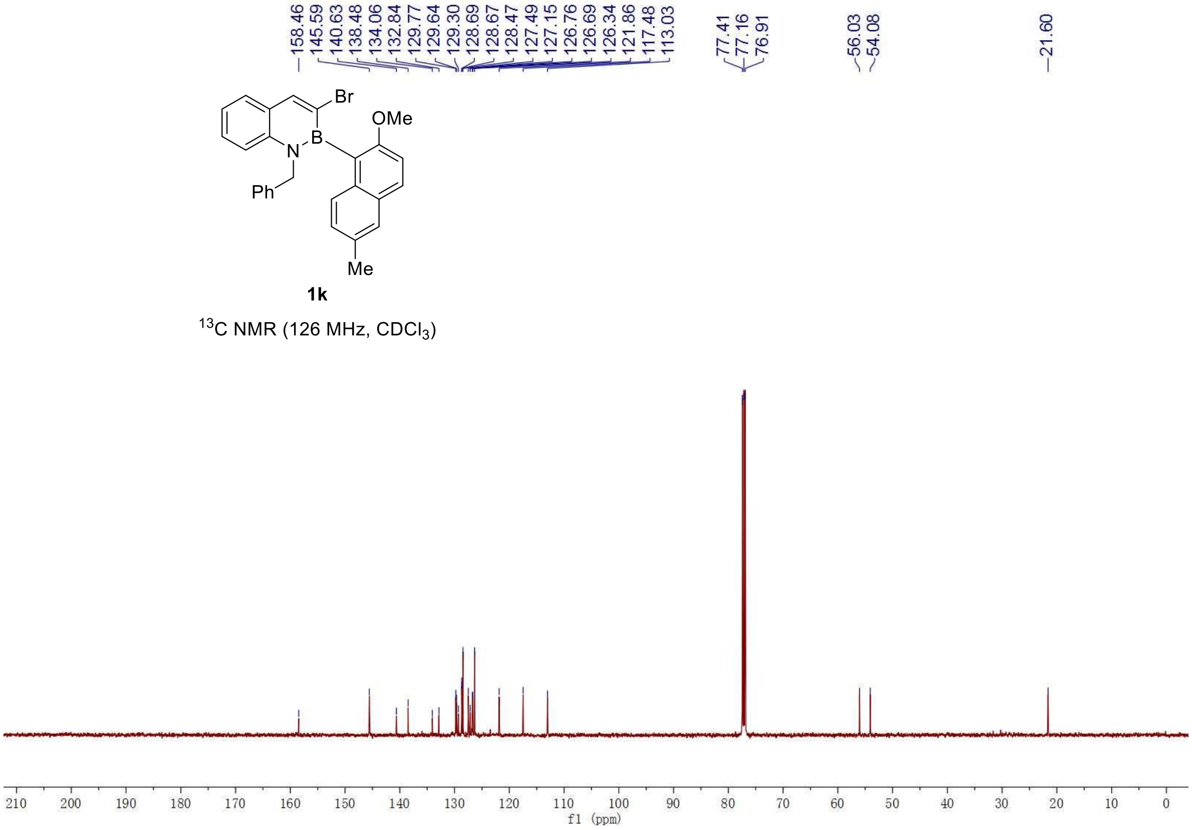

Supplementary Figure 86. ^{13}C NMR spectrum of 1k

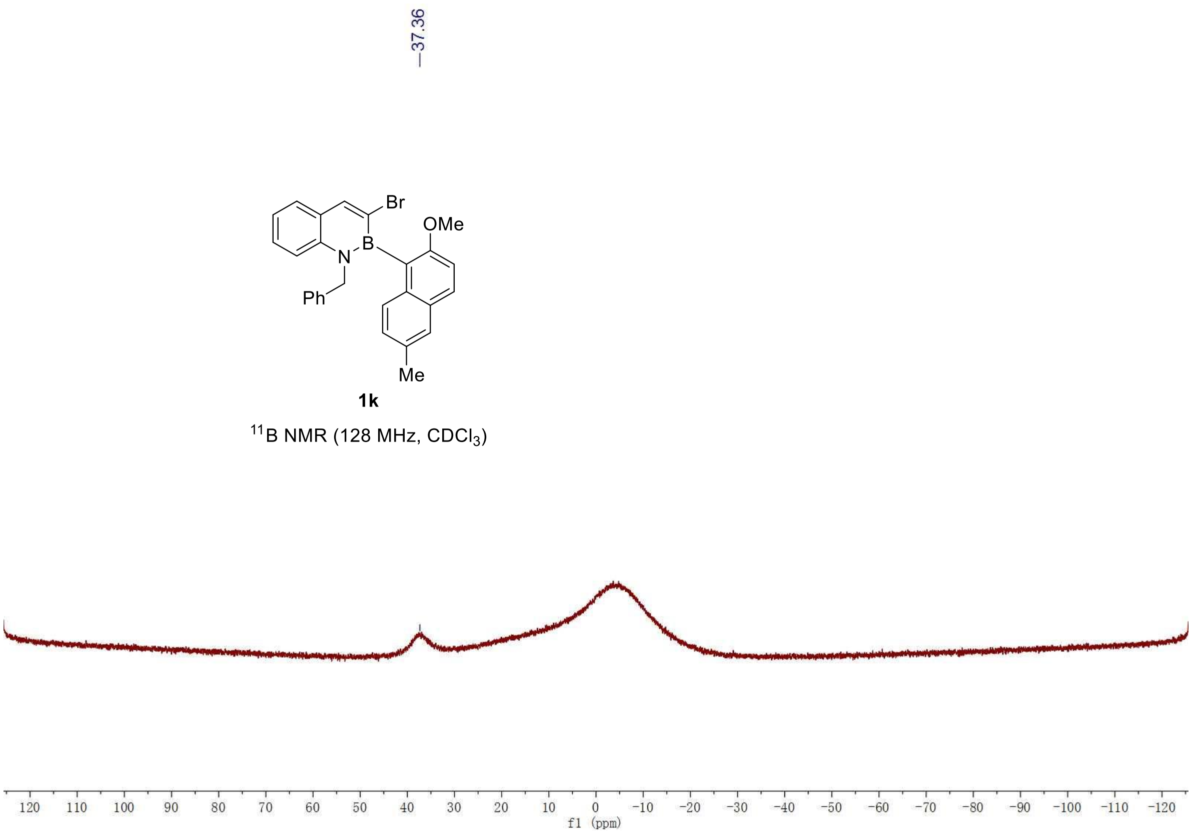

Supplementary Figure 87. ^{11}B NMR spectrum of 1k

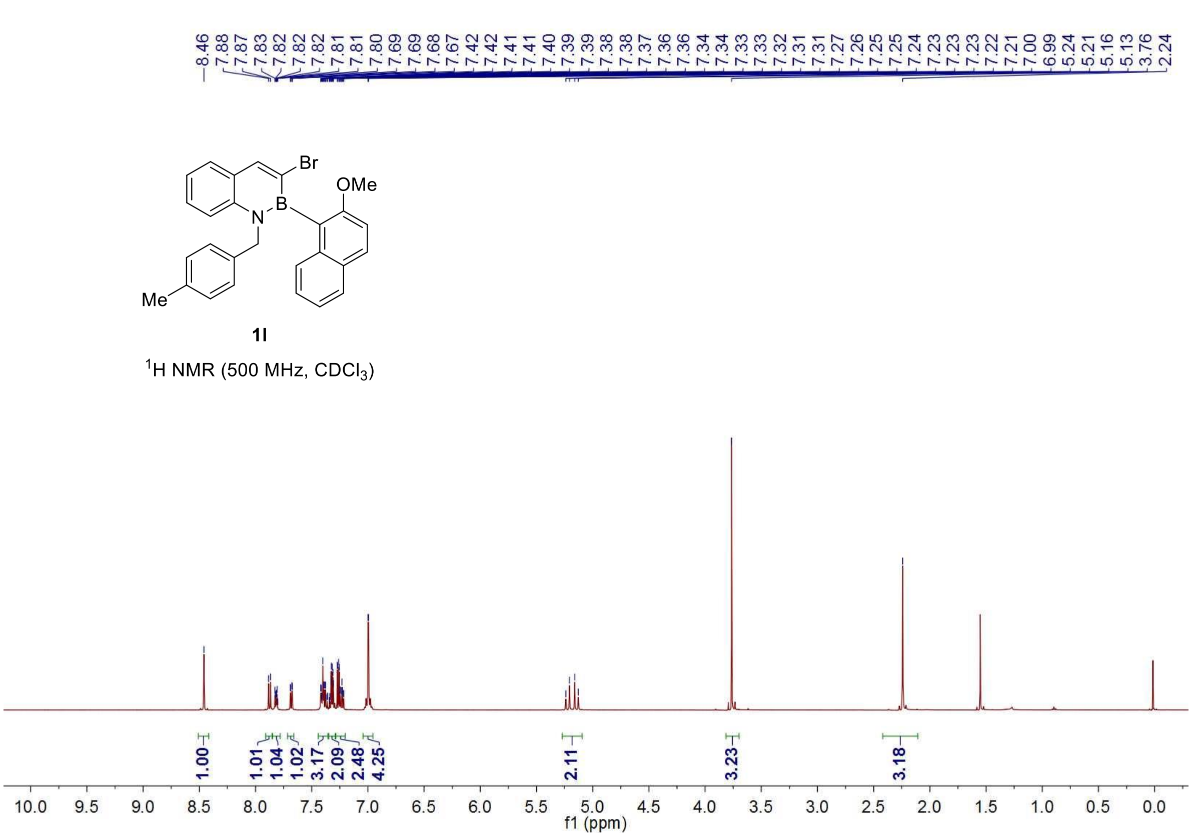

Supplementary Figure 88. ^{1}H NMR spectrum of 1l

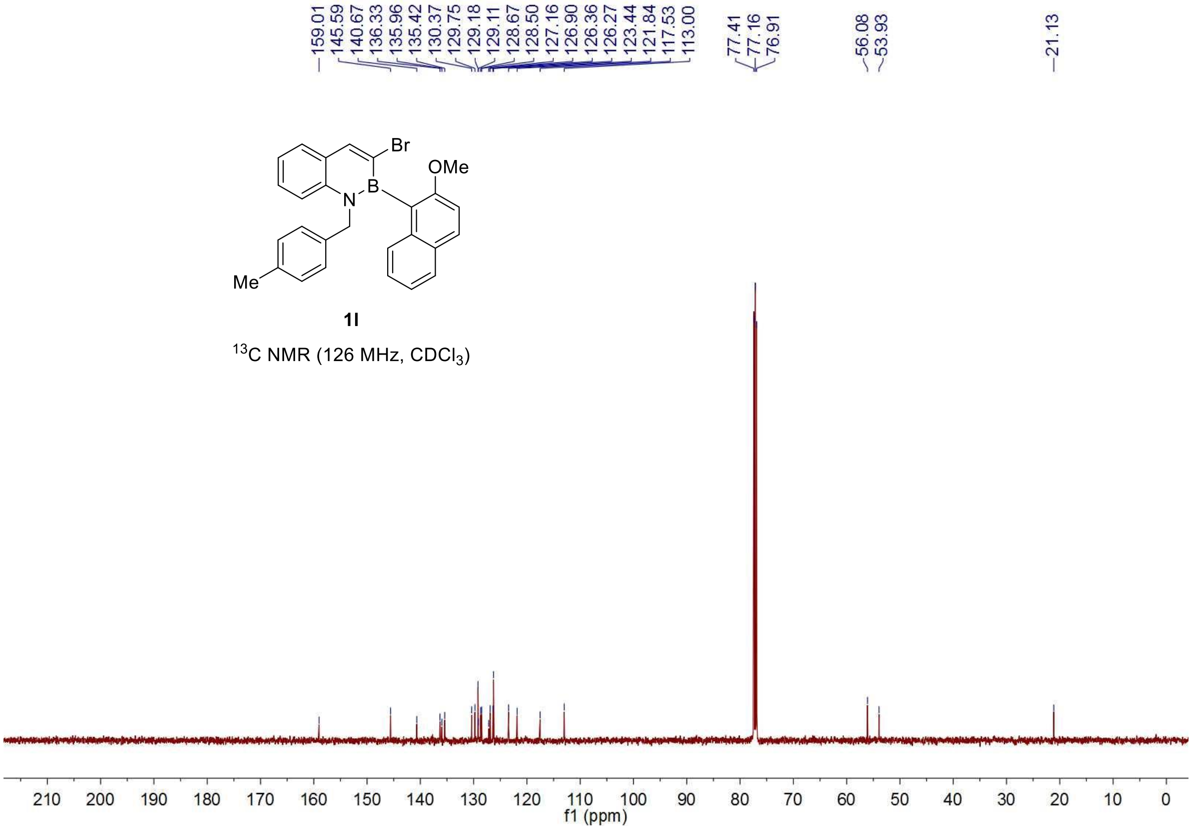

Supplementary Figure 89. ^{13}C NMR spectrum of 1l

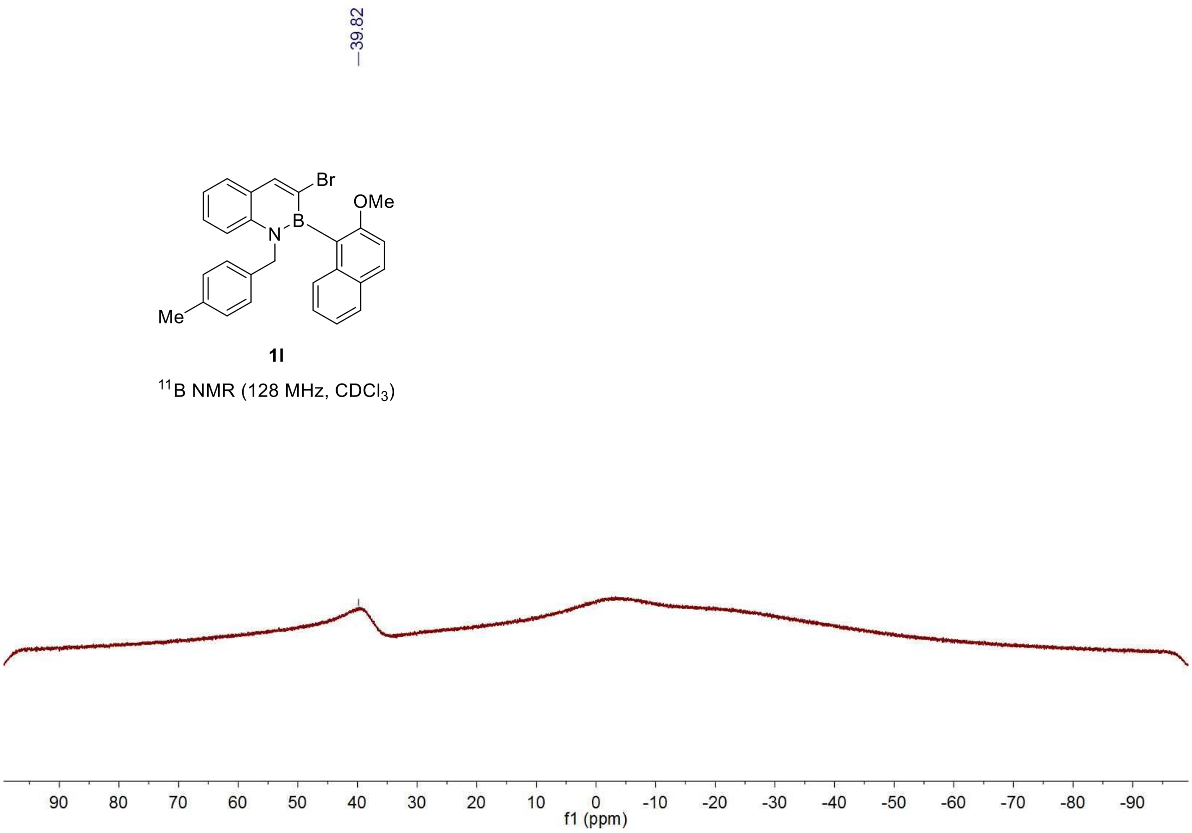

Supplementary Figure 90. ^{11}B NMR spectrum of 1l

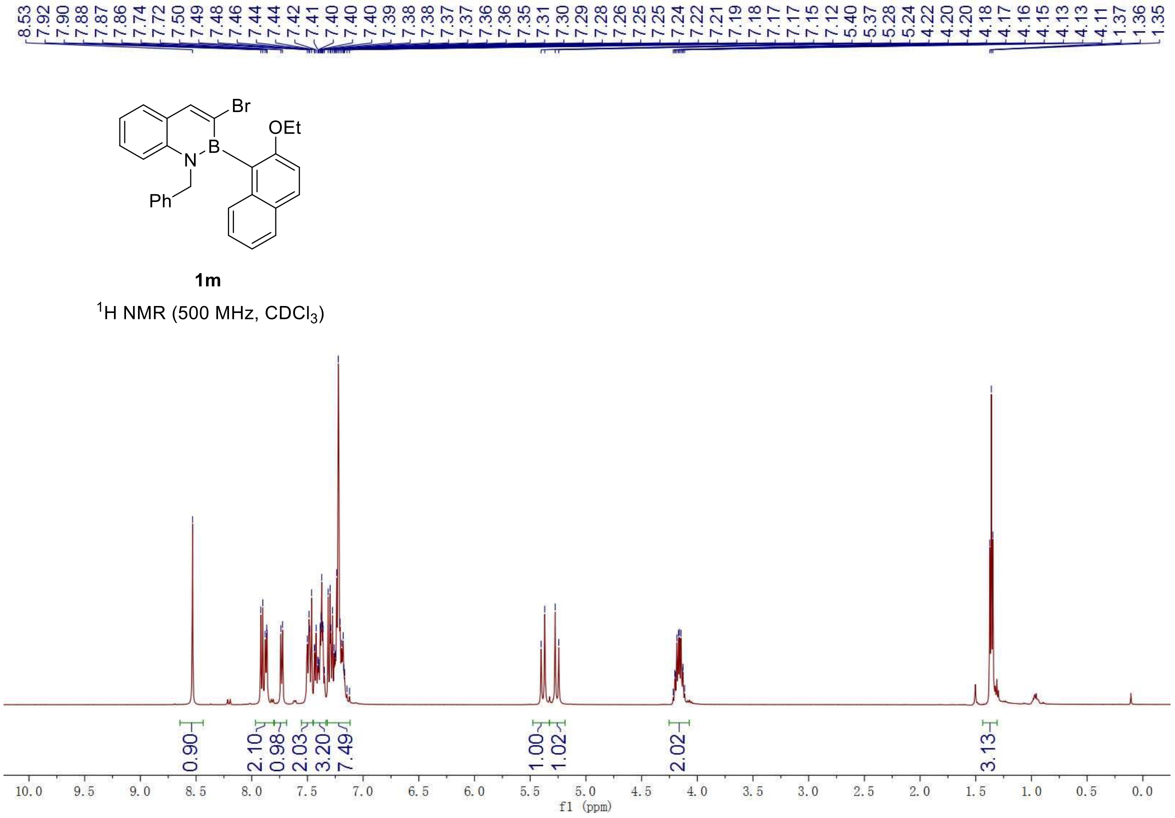

Supplementary Figure 91. ^{1}H NMR spectrum of 1m

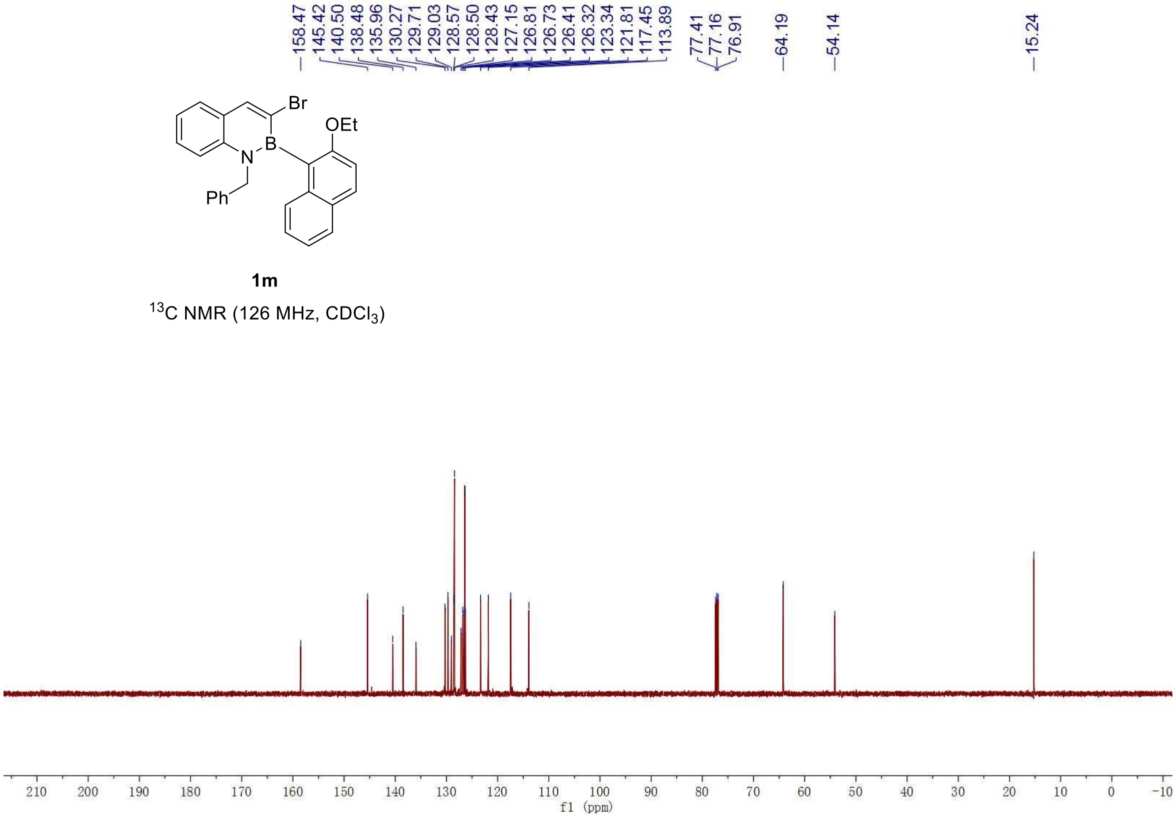

Supplementary Figure 92. ^{13}C NMR spectrum of 1m

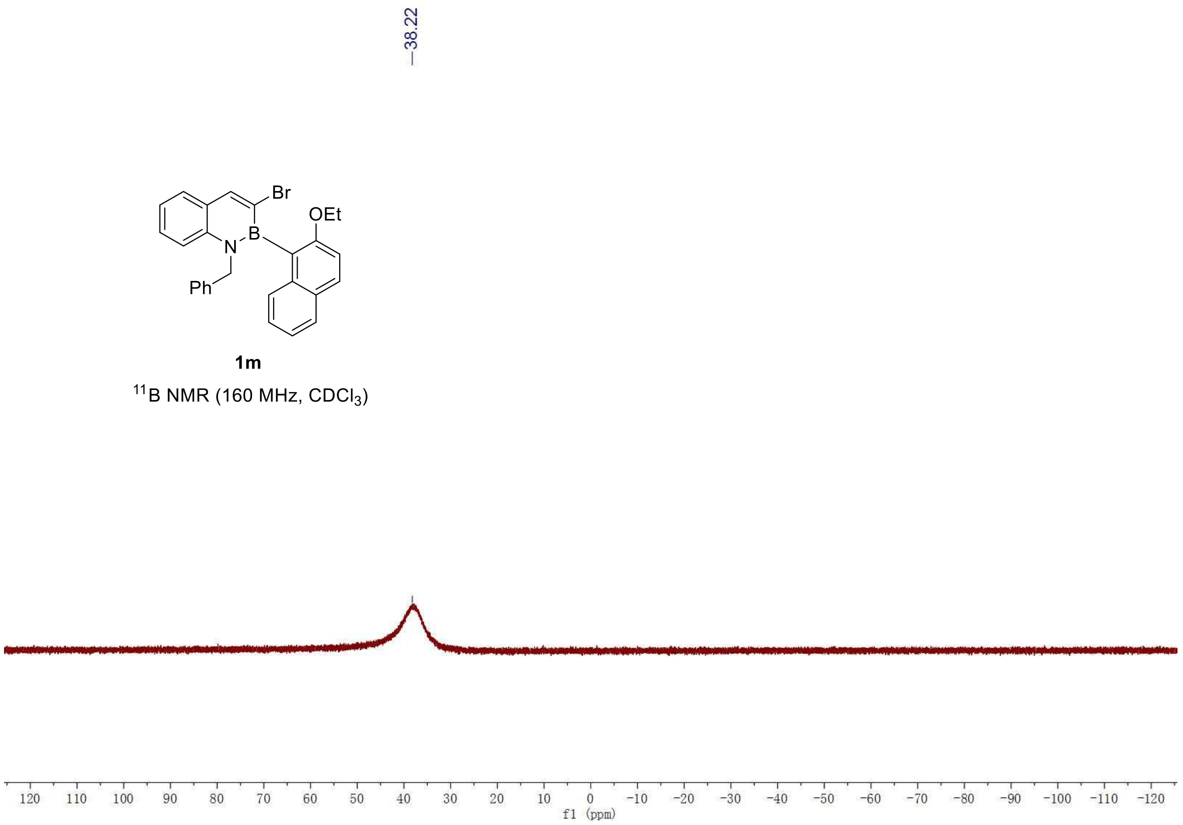

Supplementary Figure 93. ^{11}B NMR spectrum of 1m

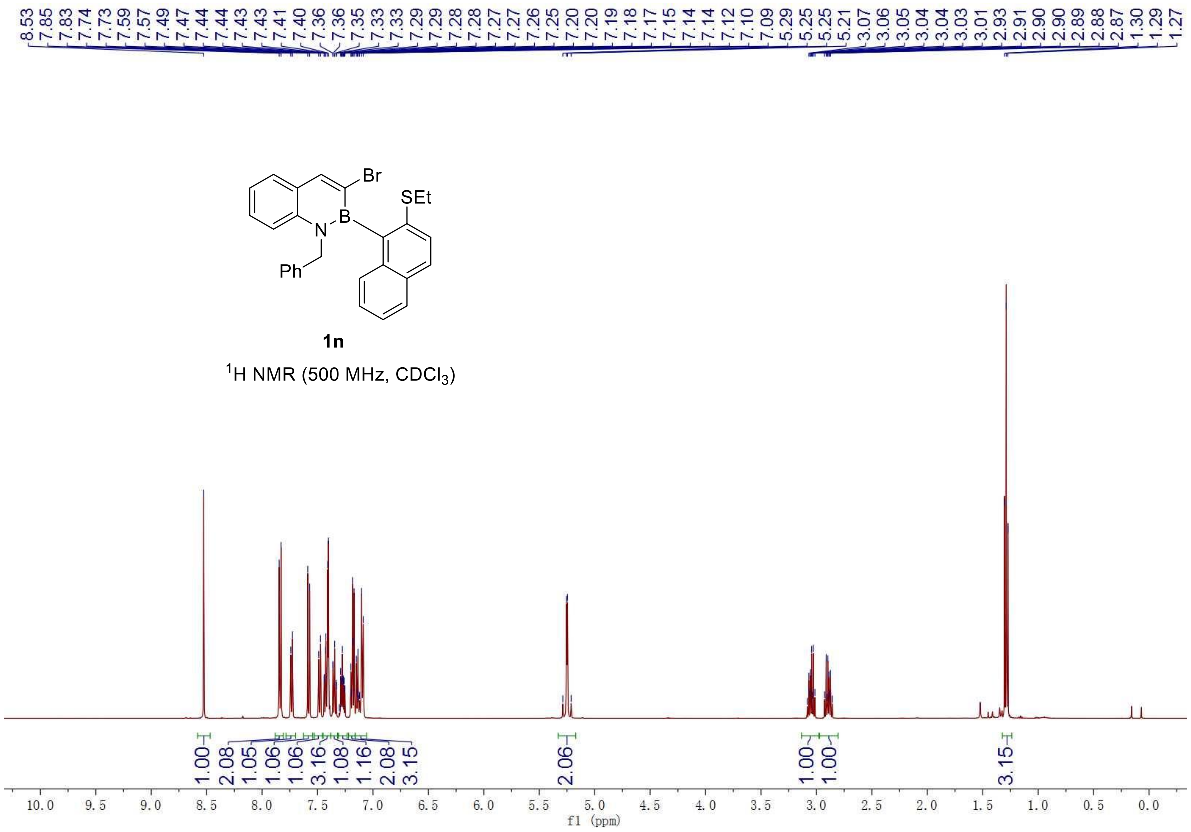

Supplementary Figure 94. ^{1}H NMR spectrum of 1n

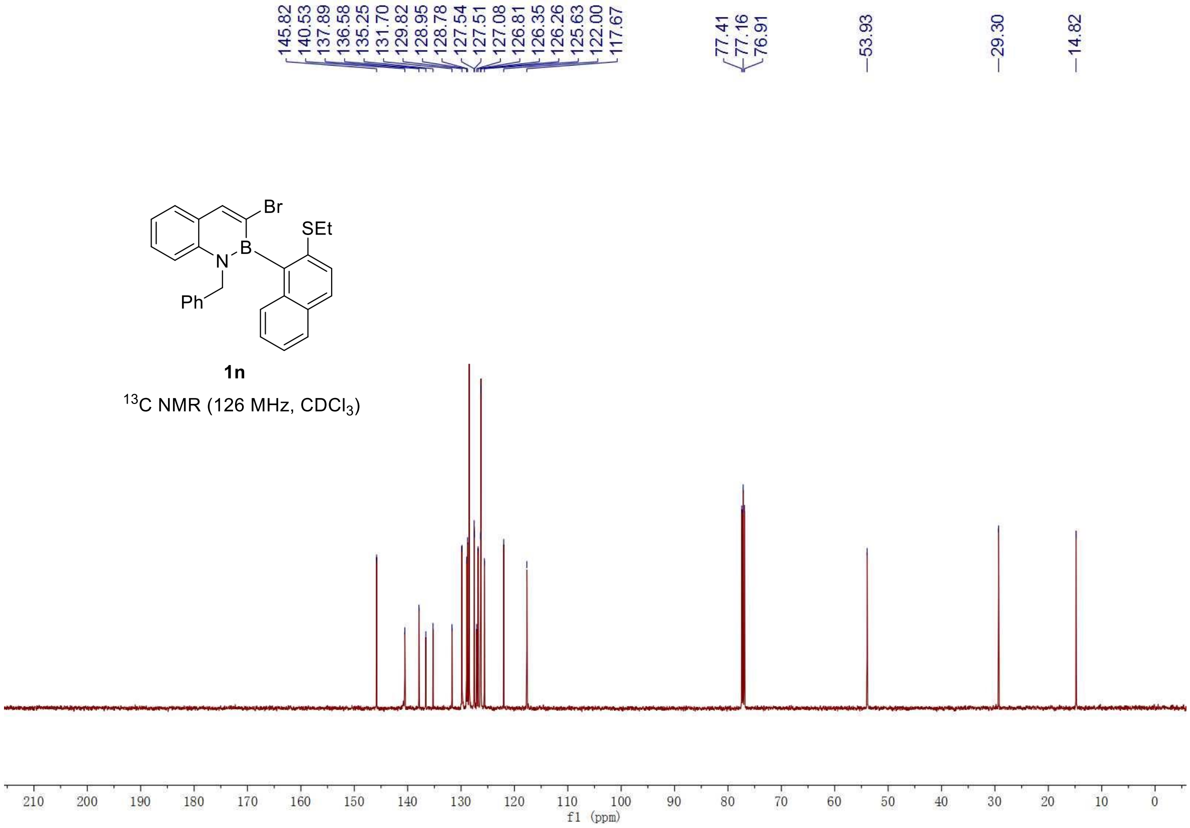

Supplementary Figure 95. ^{13}C NMR spectrum of 1n

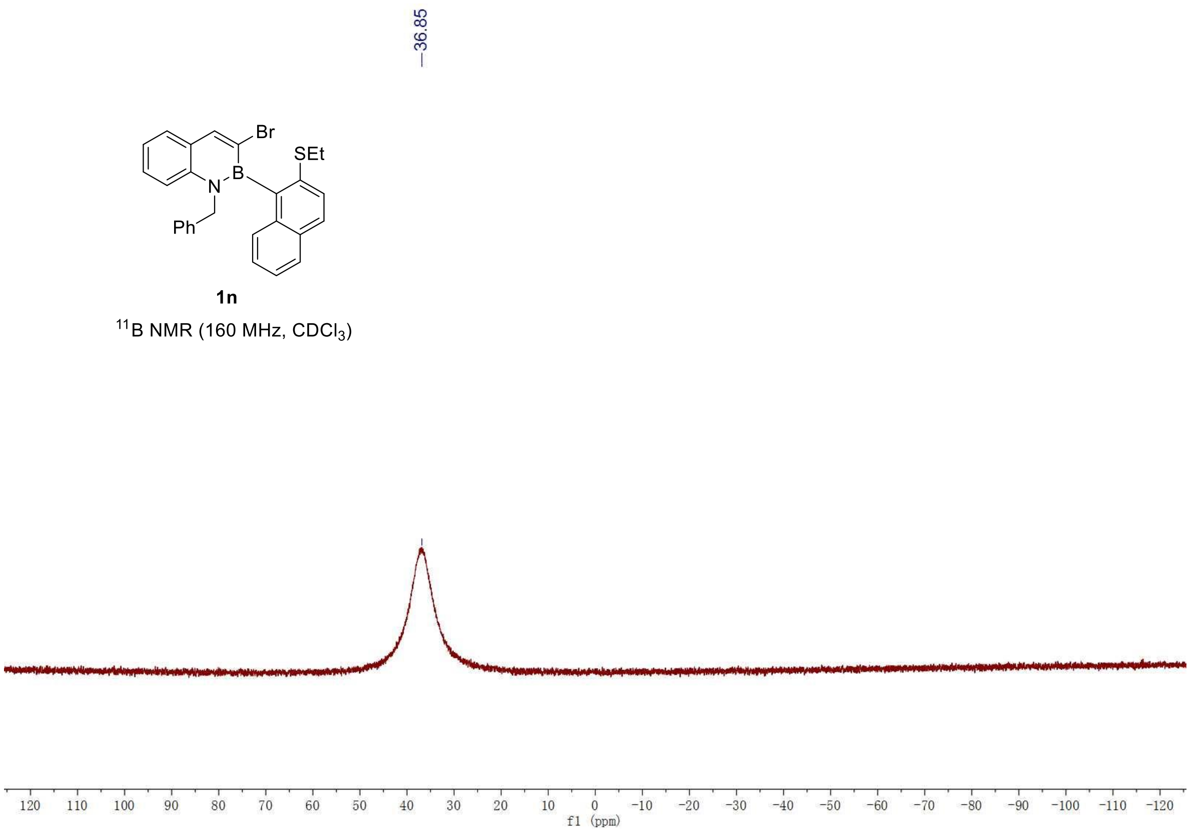

Supplementary Figure 96. ^{11}B NMR spectrum of 1n

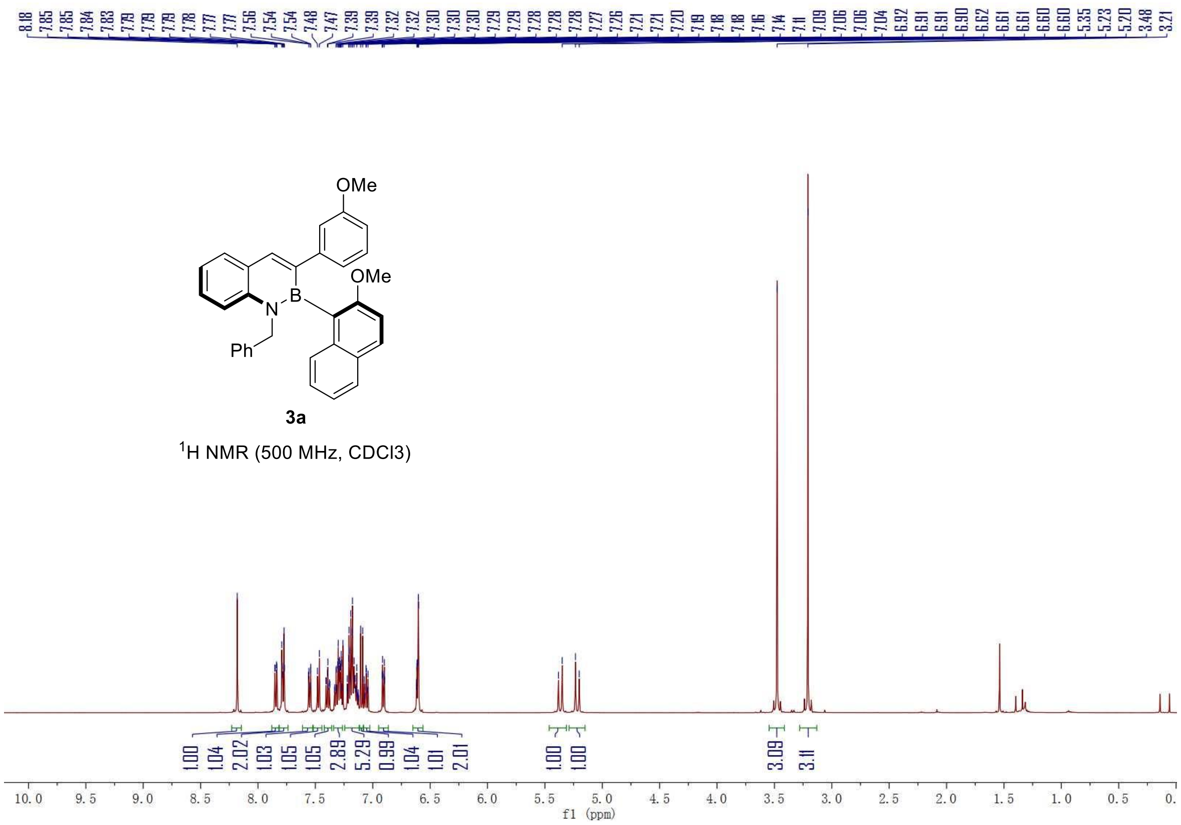

Supplementary Figure 97. ^{1}H NMR spectrum of 3a

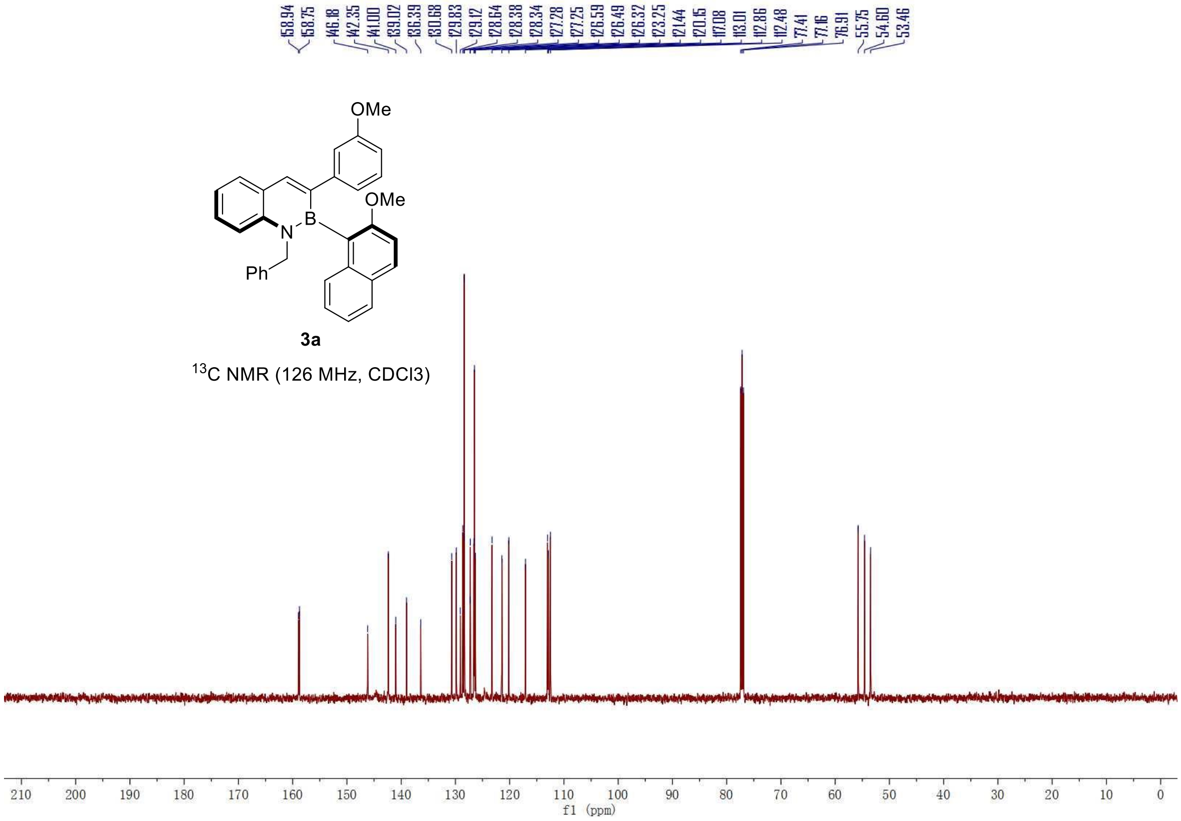

Supplementary Figure 98. ^{13}C NMR spectrum of 3a

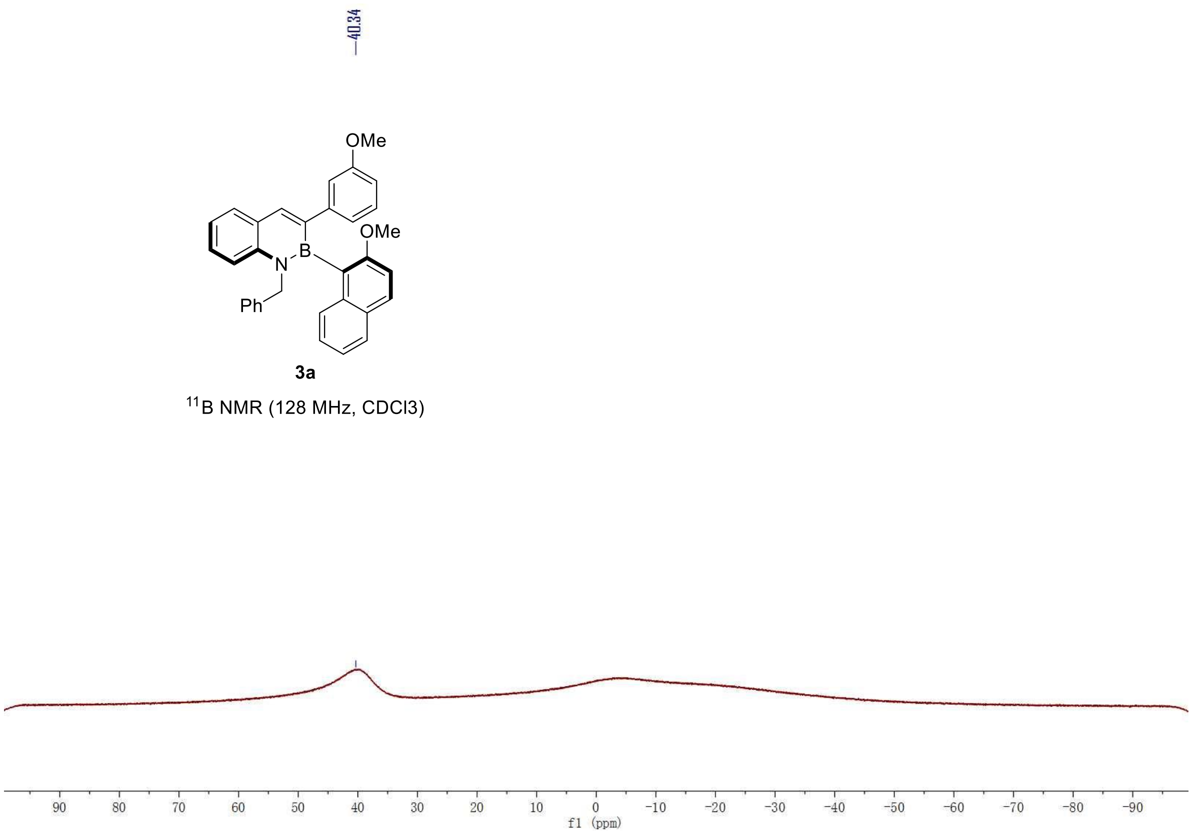

Supplementary Figure 99. ^{11}B NMR spectrum of 3a

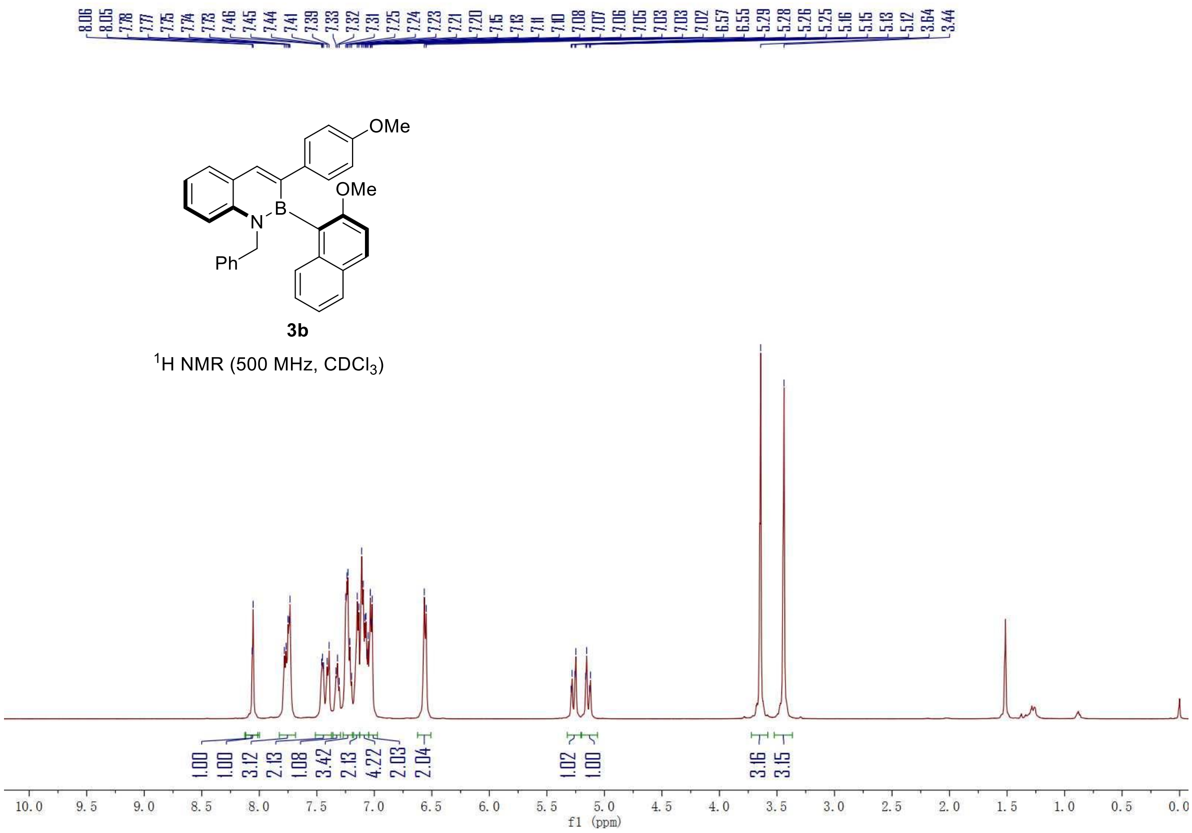

Supplementary Figure 100. ^{1}H NMR spectrum of 3b

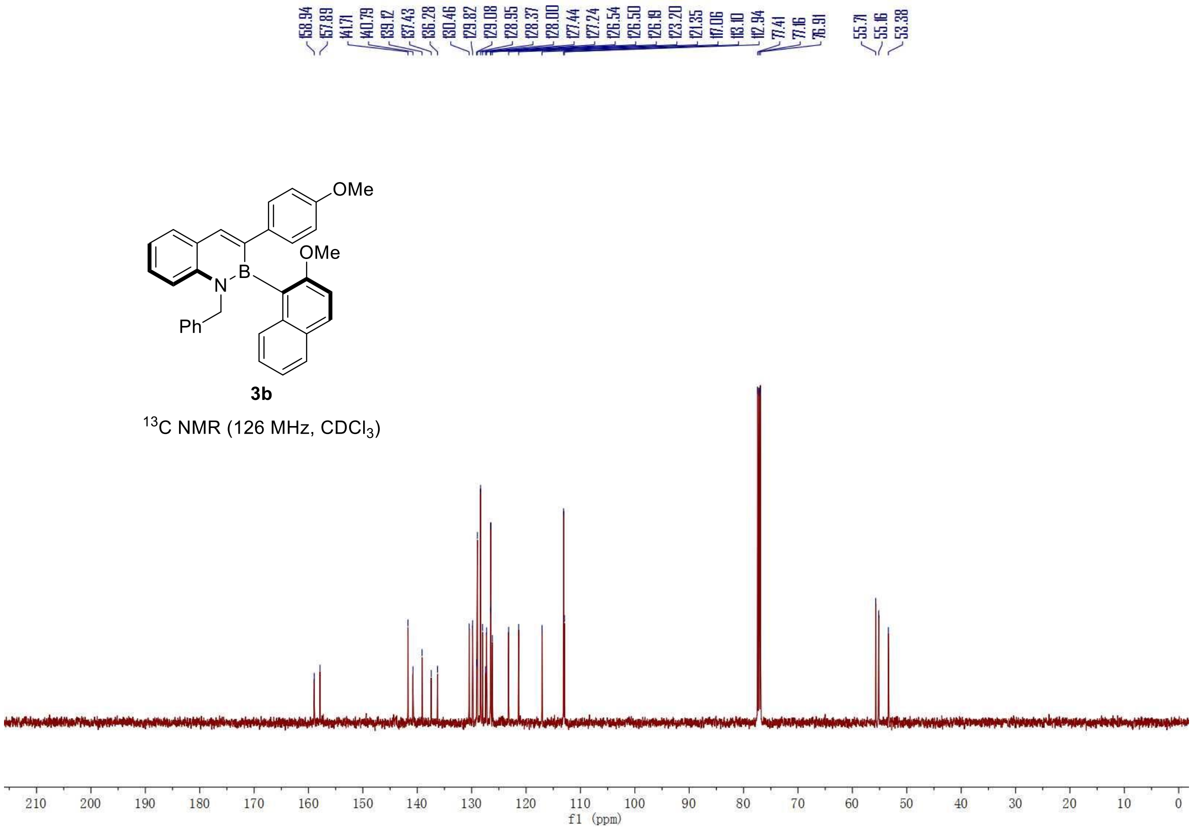

Supplementary Figure 101. ^{13}C NMR spectrum of 3b

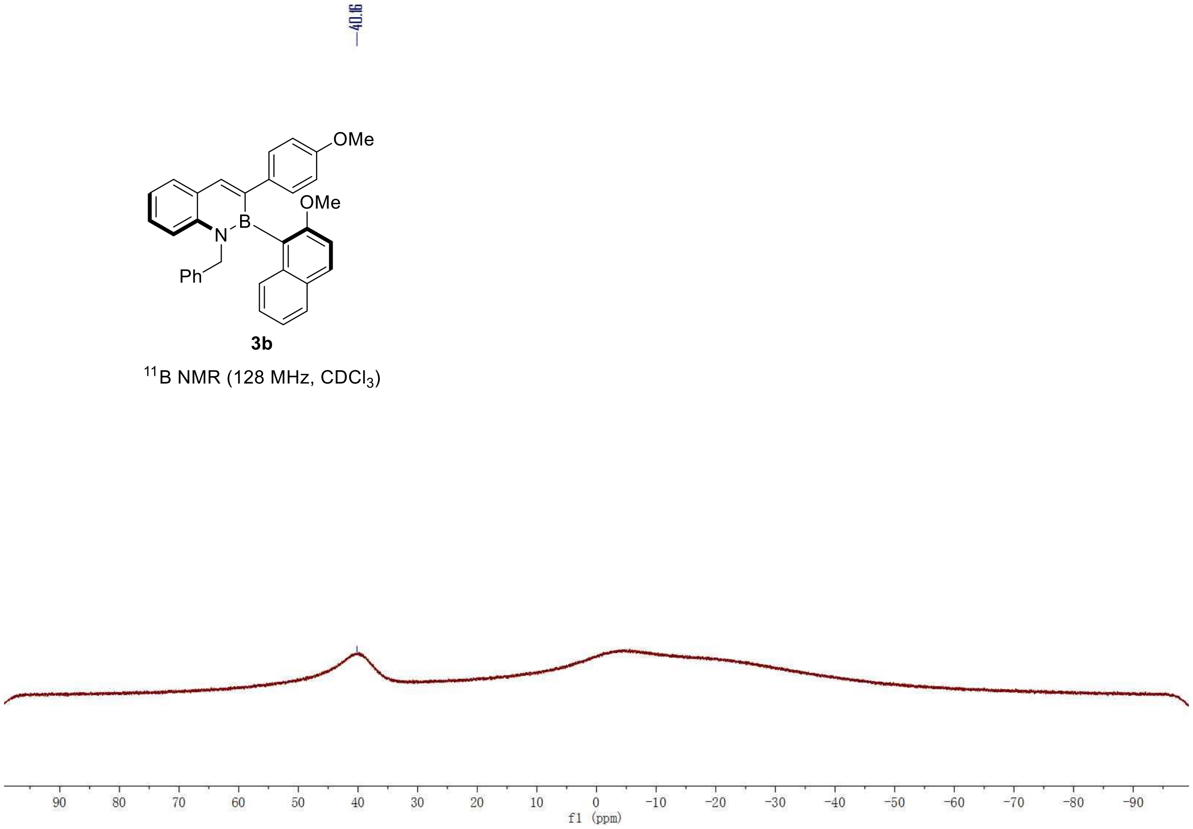

Supplementary Figure 102. ^{11}B NMR spectrum of 3c

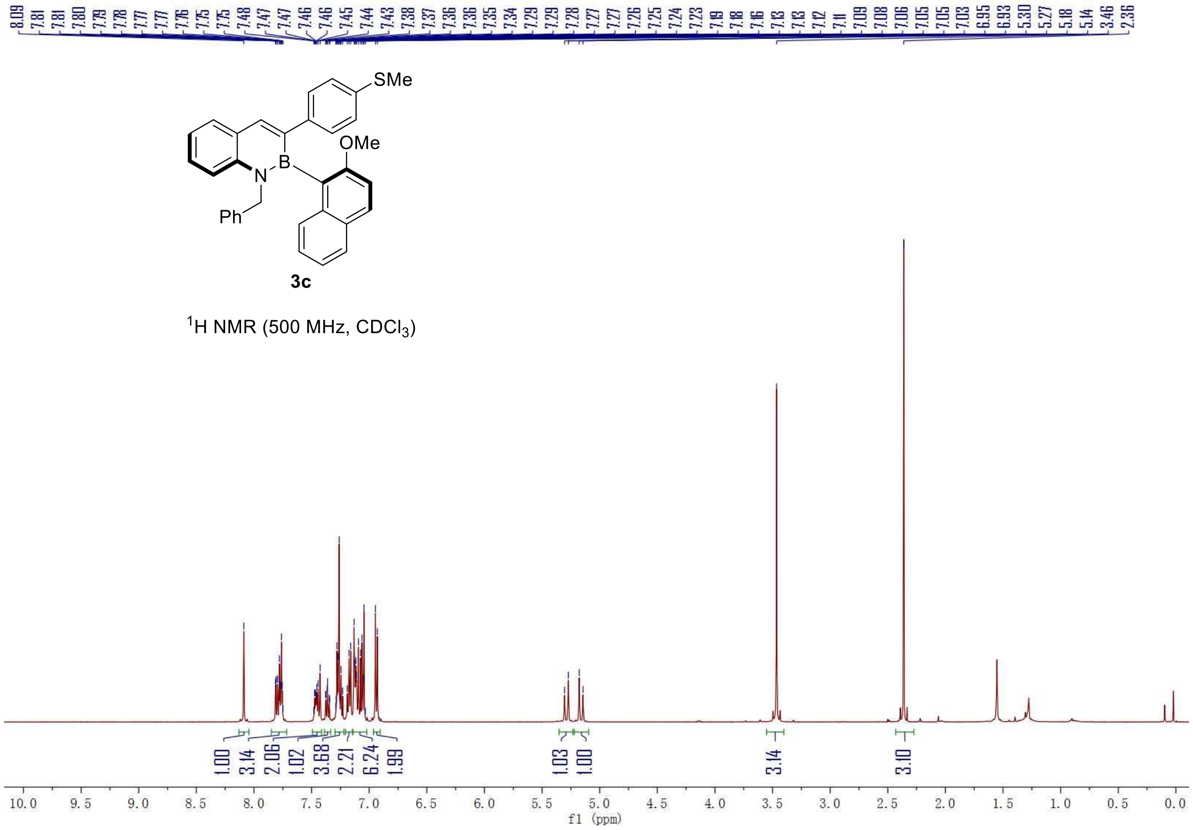

Supplementary Figure 103. ^{1}H NMR spectrum of 3c

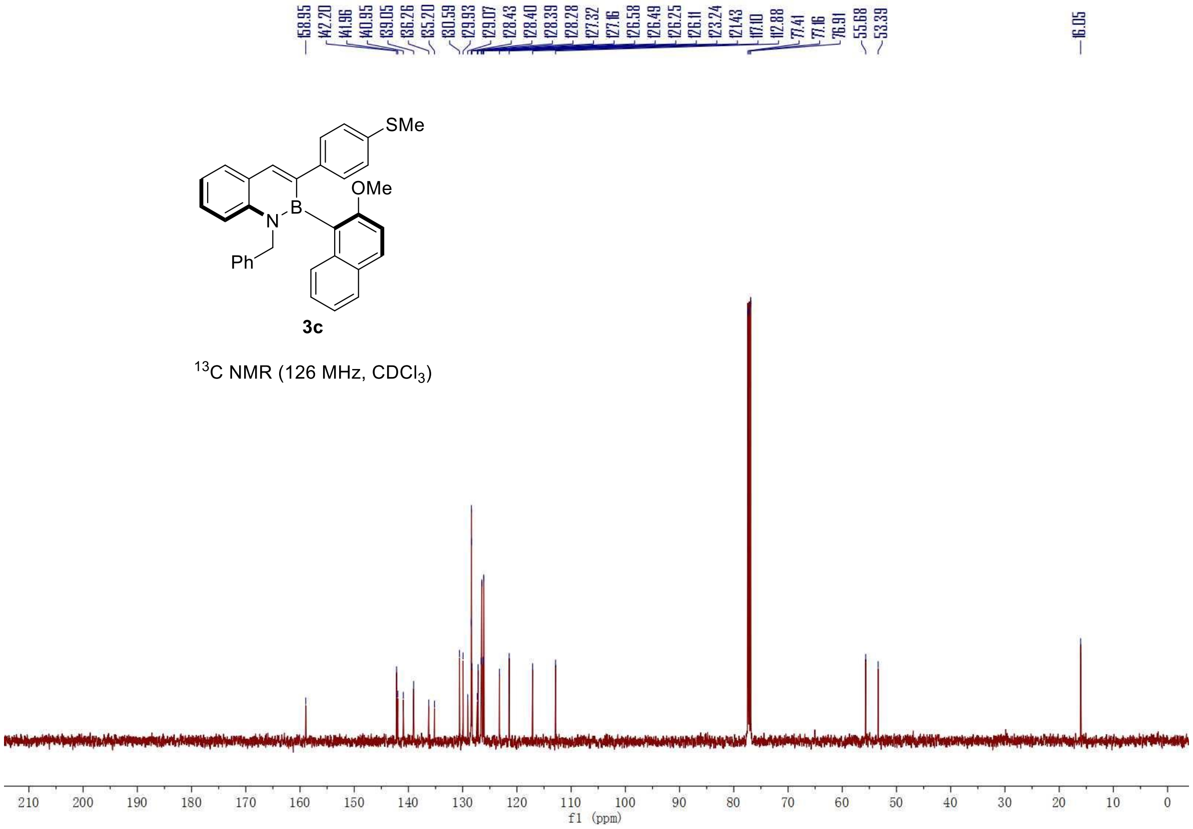

Supplementary Figure 104. ^{13}C NMR spectrum of 3c

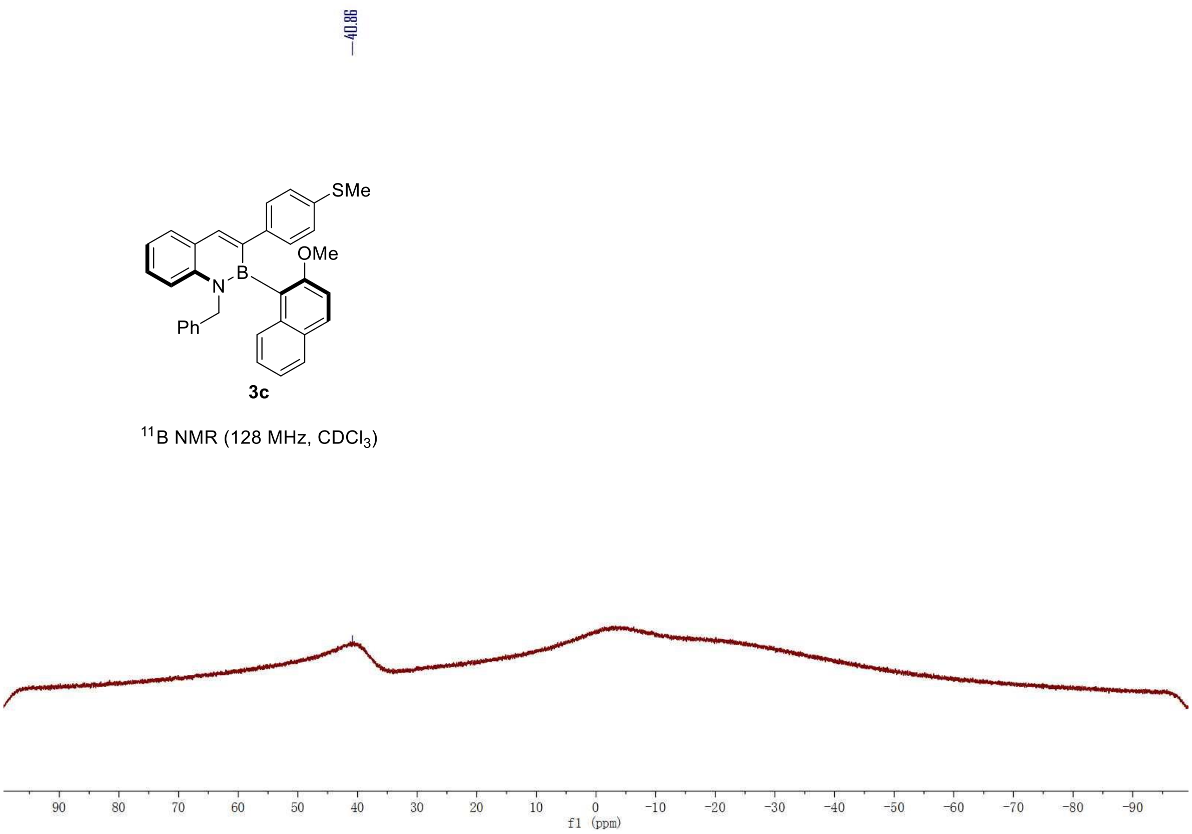

Supplementary Figure 105. ^{11}B NMR spectrum of 3c

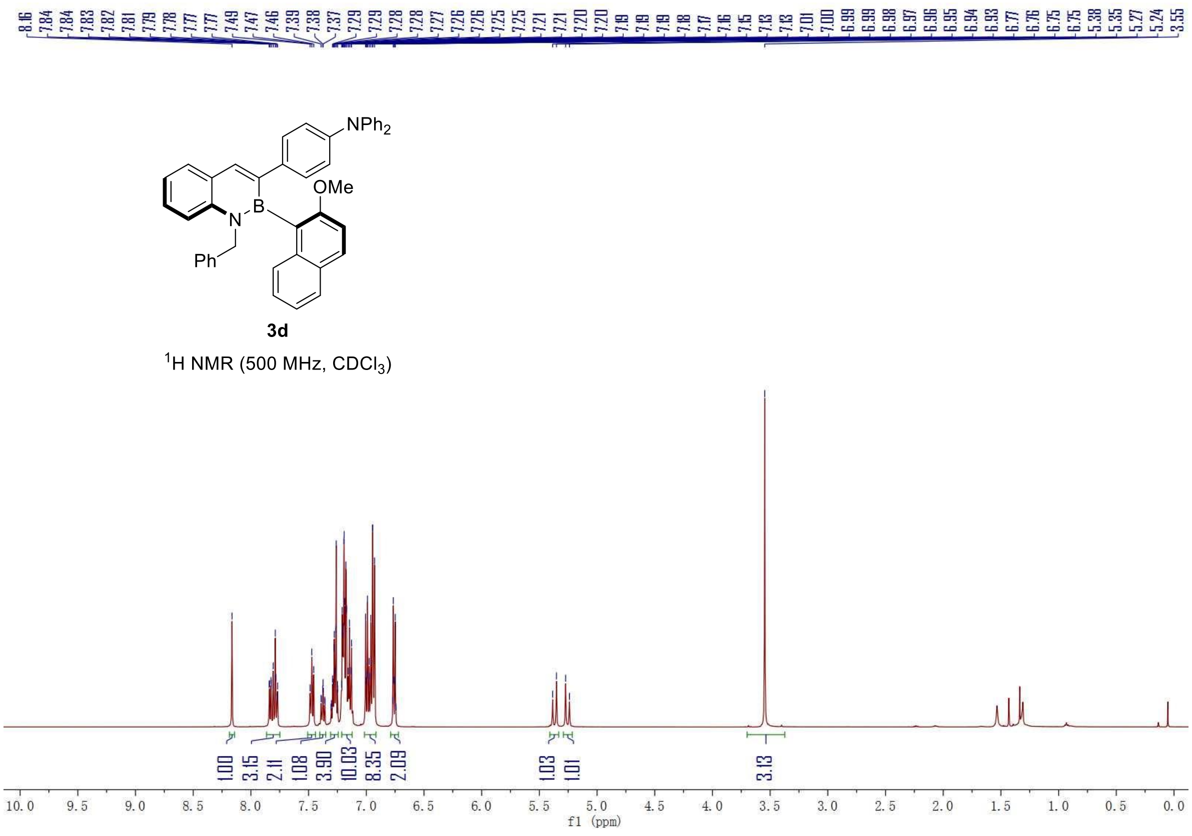

Supplementary Figure 106. ^{1}H NMR spectrum of 3d

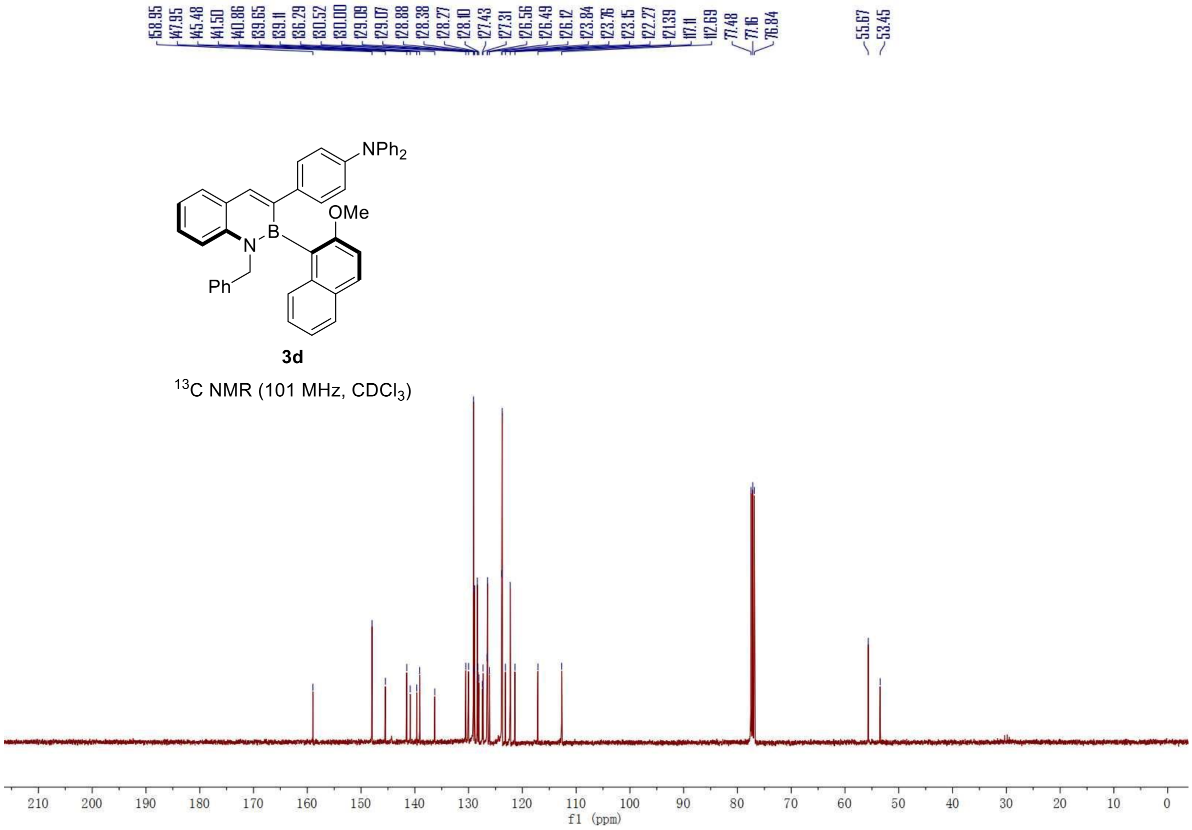

Supplementary Figure 107. ^{13}C NMR spectrum of 3d

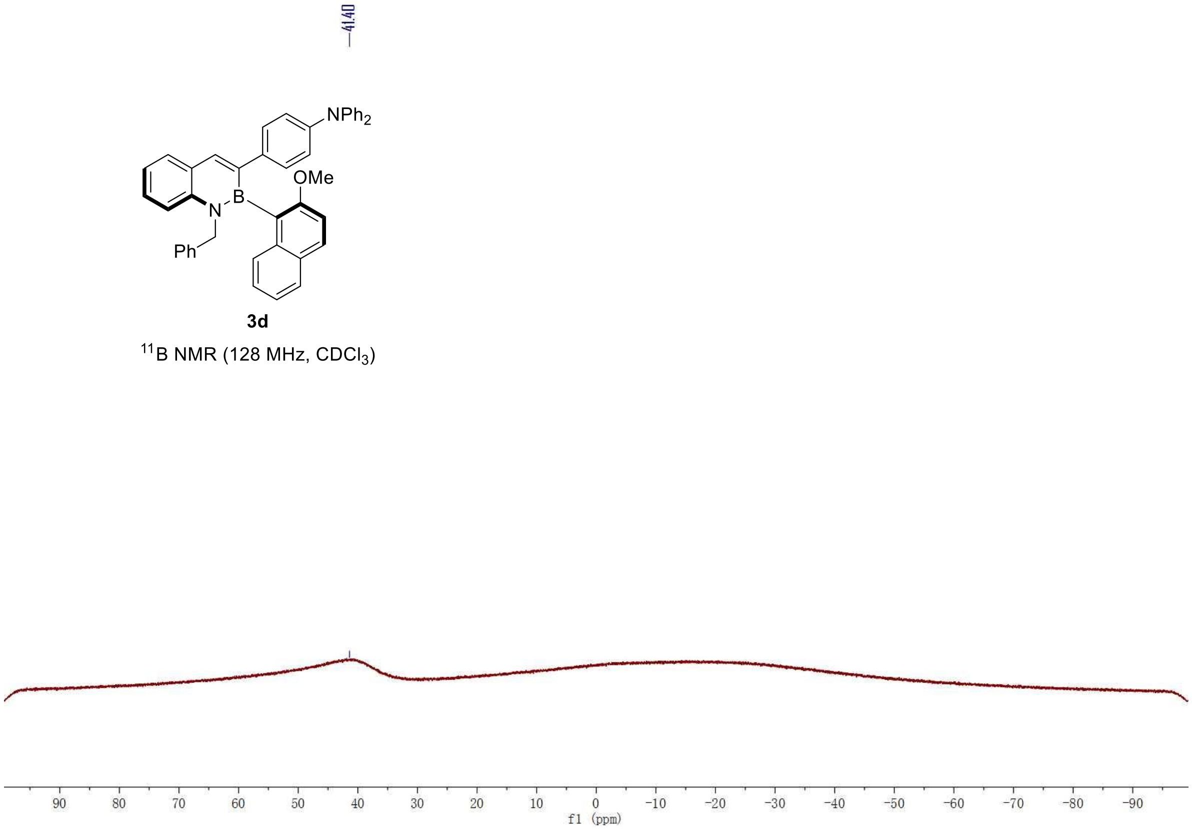

Supplementary Figure 108. ^{11}B NMR spectrum of 3d

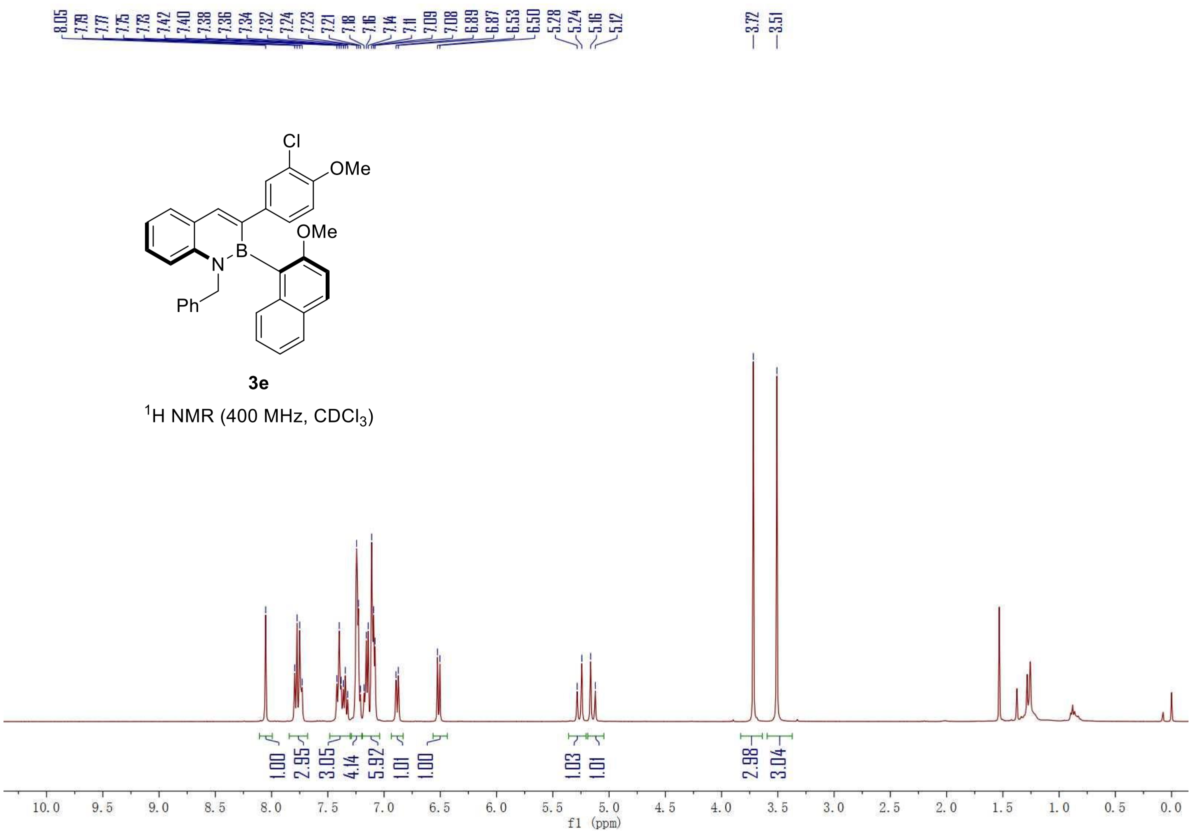

Supplementary Figure 109. ^{1}H NMR spectrum of 3e

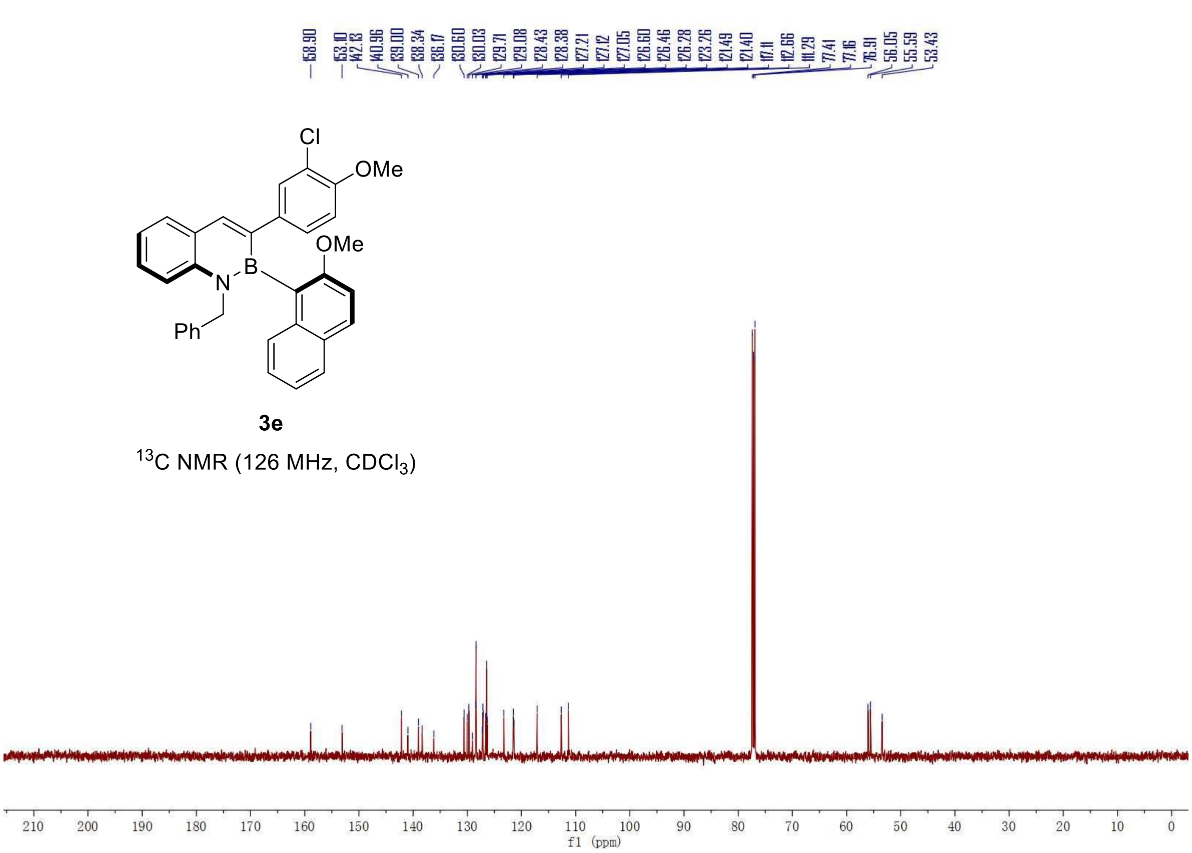

Supplementary Figure 110. ^{13}C NMR spectrum of 3e

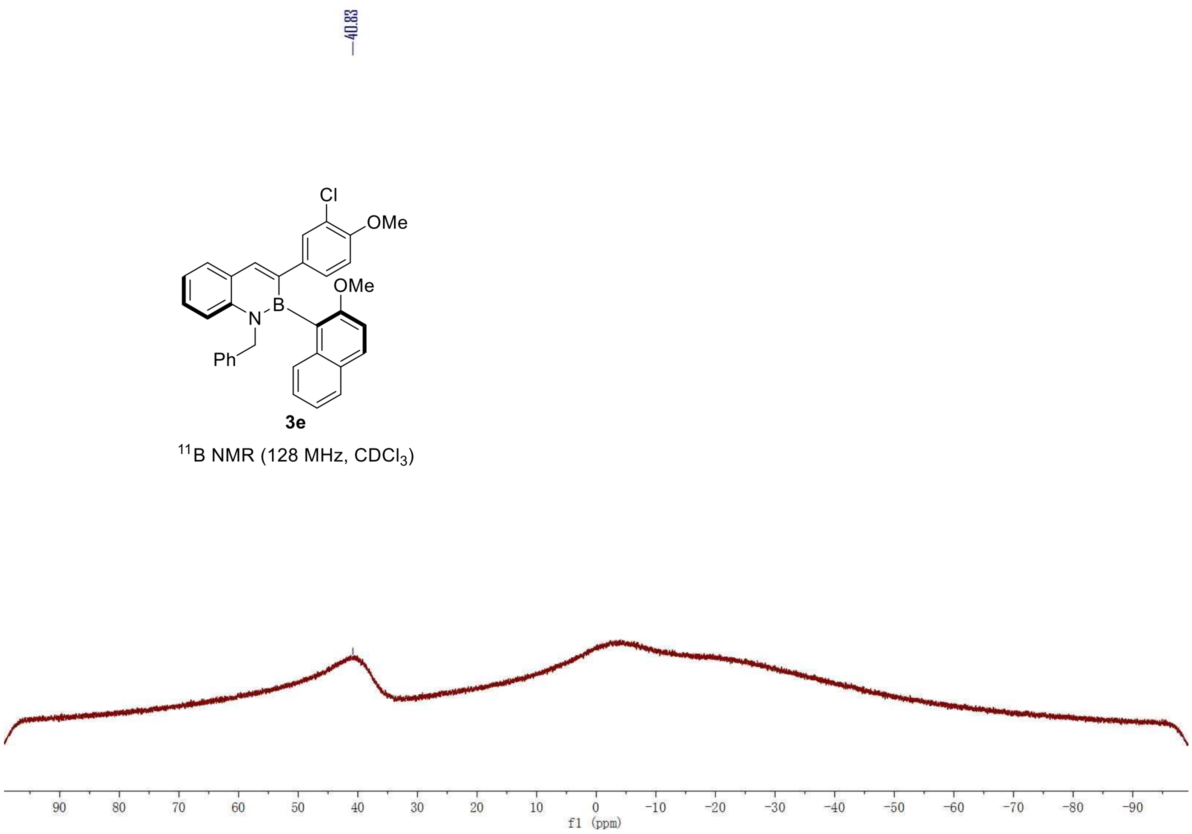

Supplementary Figure 111. ^{11}B NMR spectrum of 3e

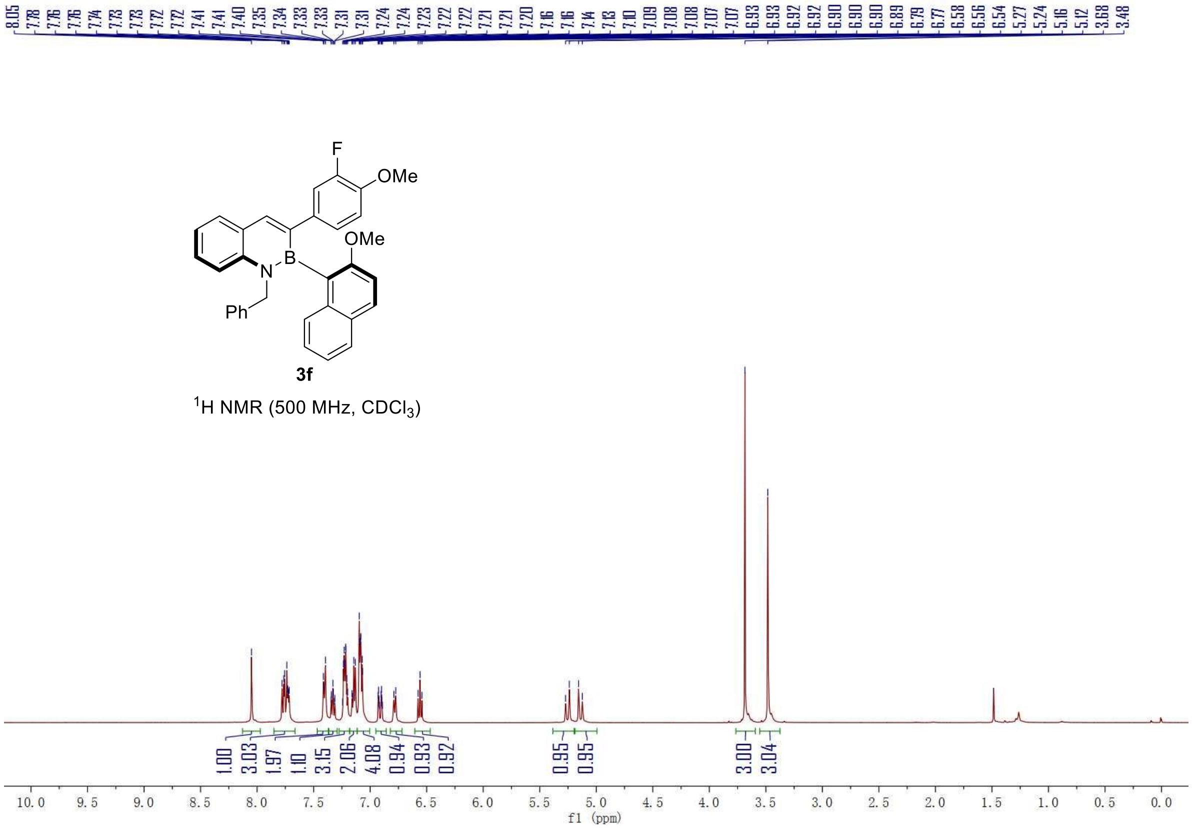

Supplementary Figure 112. ^{1}H NMR spectrum of 3f

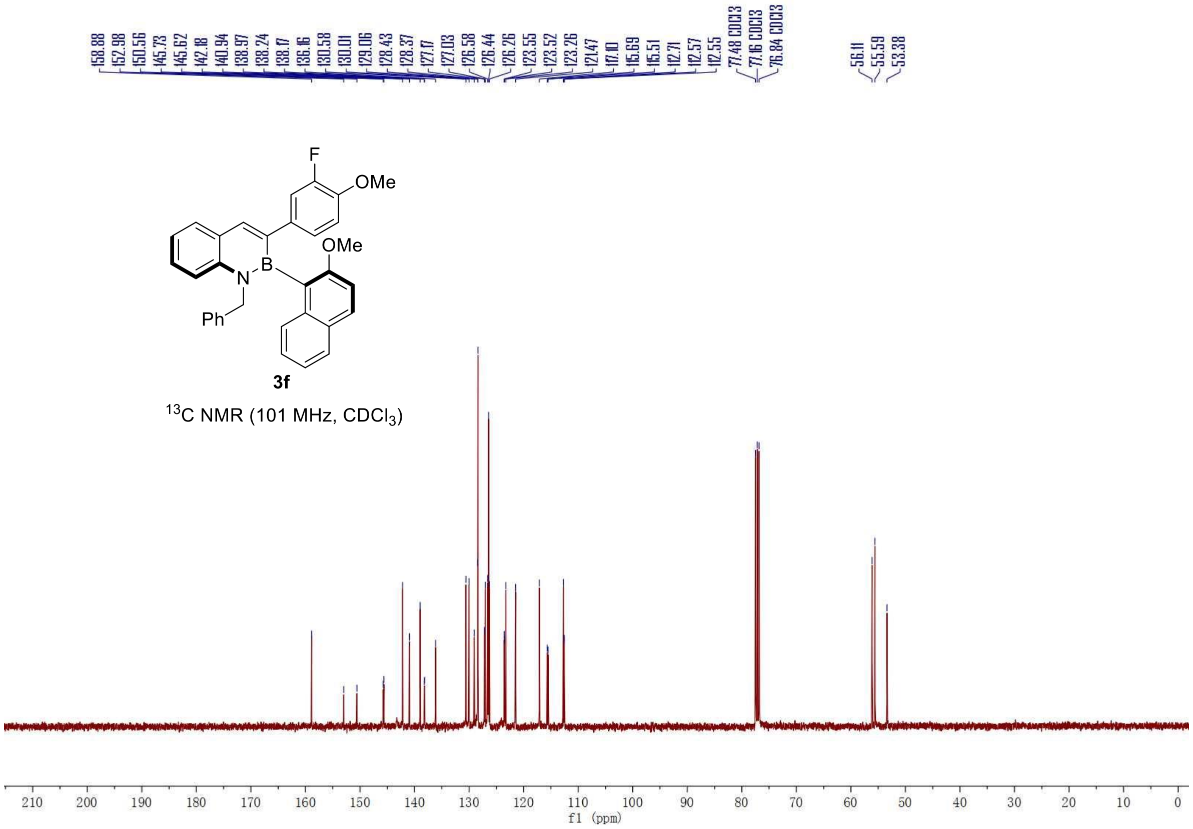

Supplementary Figure 113. ^{13}C NMR spectrum of 3f

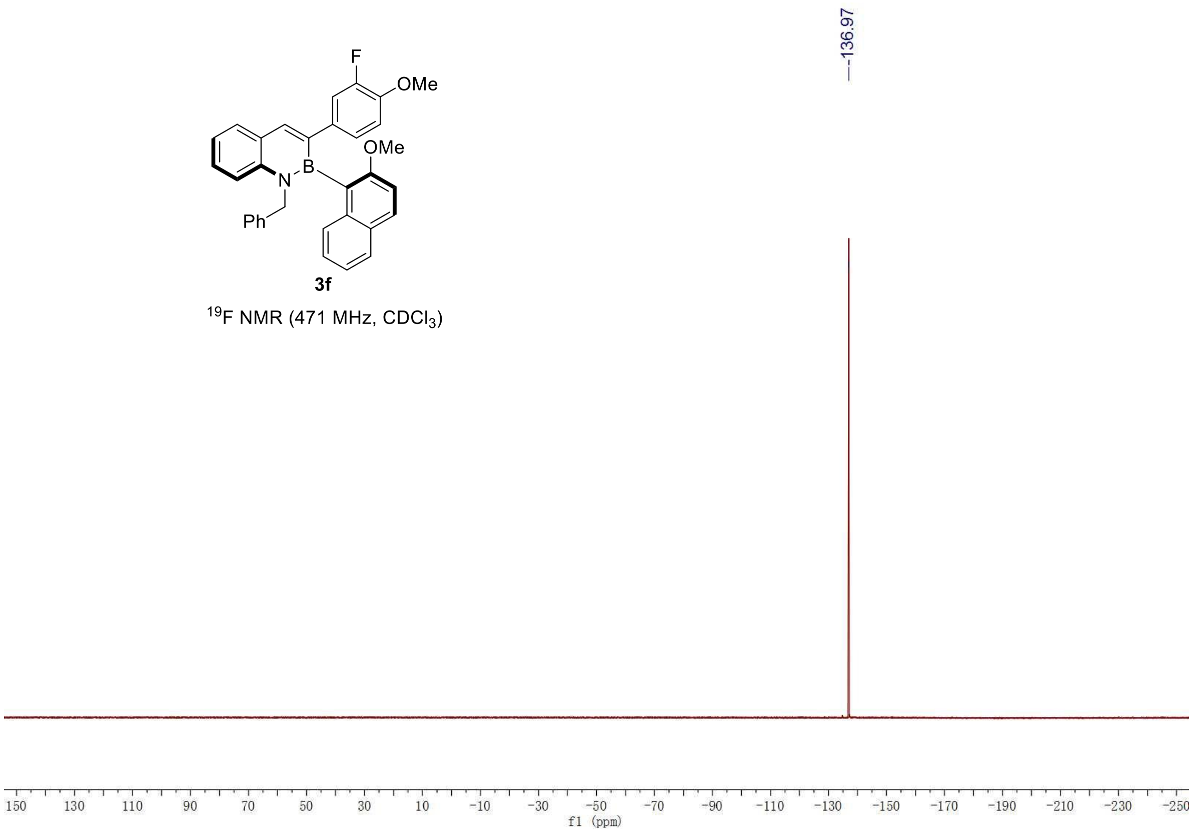

Supplementary Figure 114. ^{19}F NMR spectrum of 3f

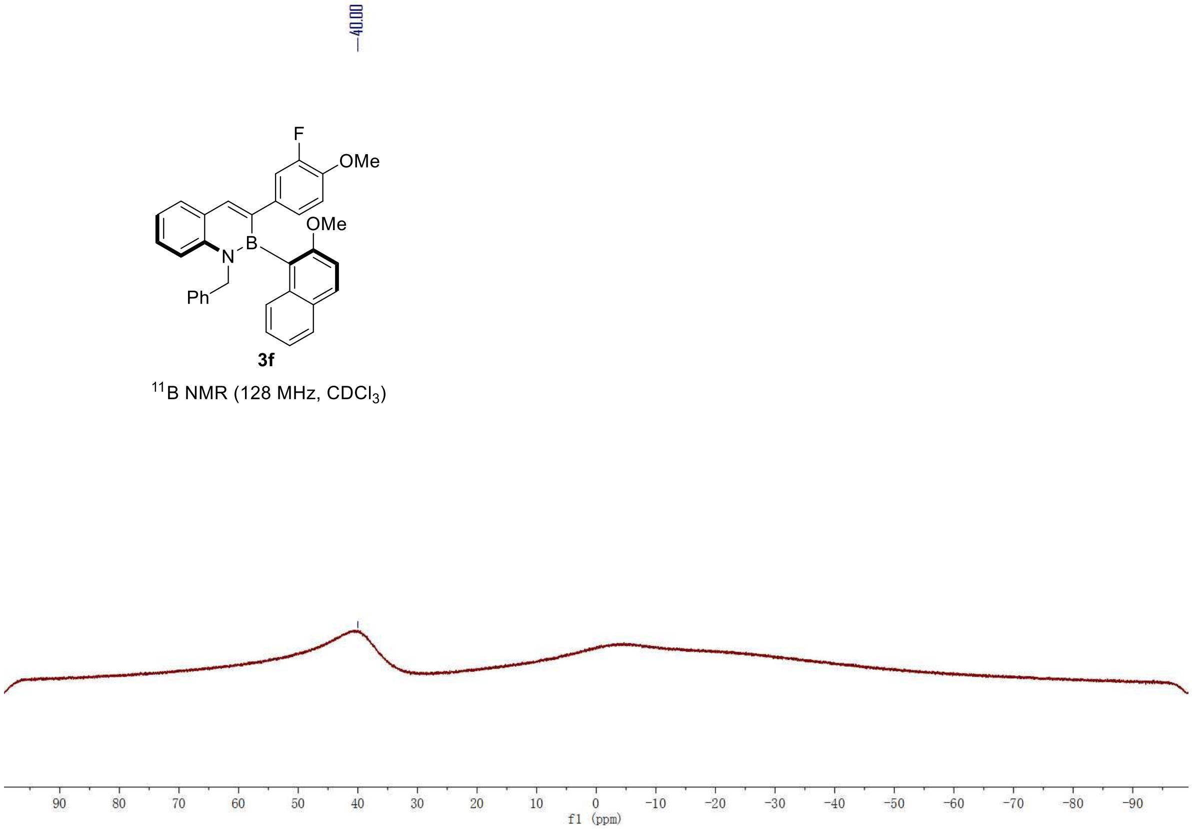

Supplementary Figure 115. ^{11}B NMR spectrum of 3f

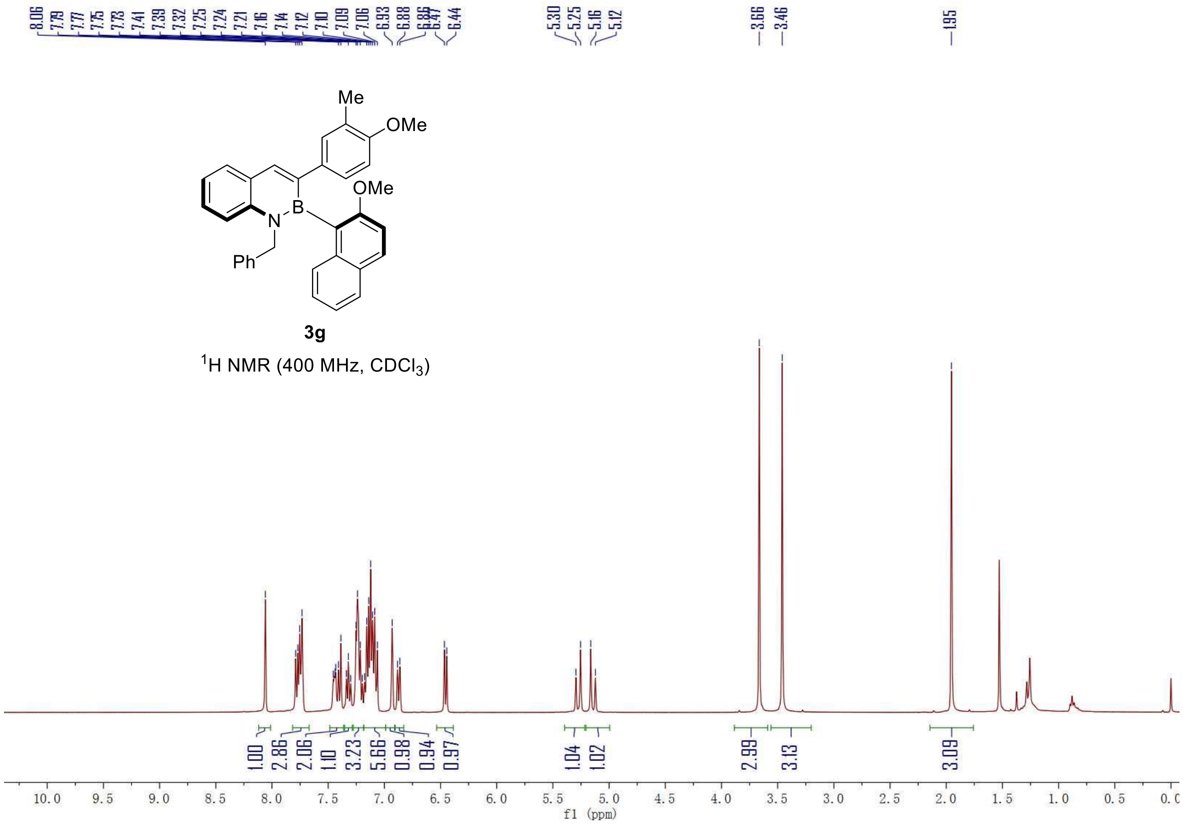

Supplementary Figure 116. ^{1}H NMR spectrum of 3g

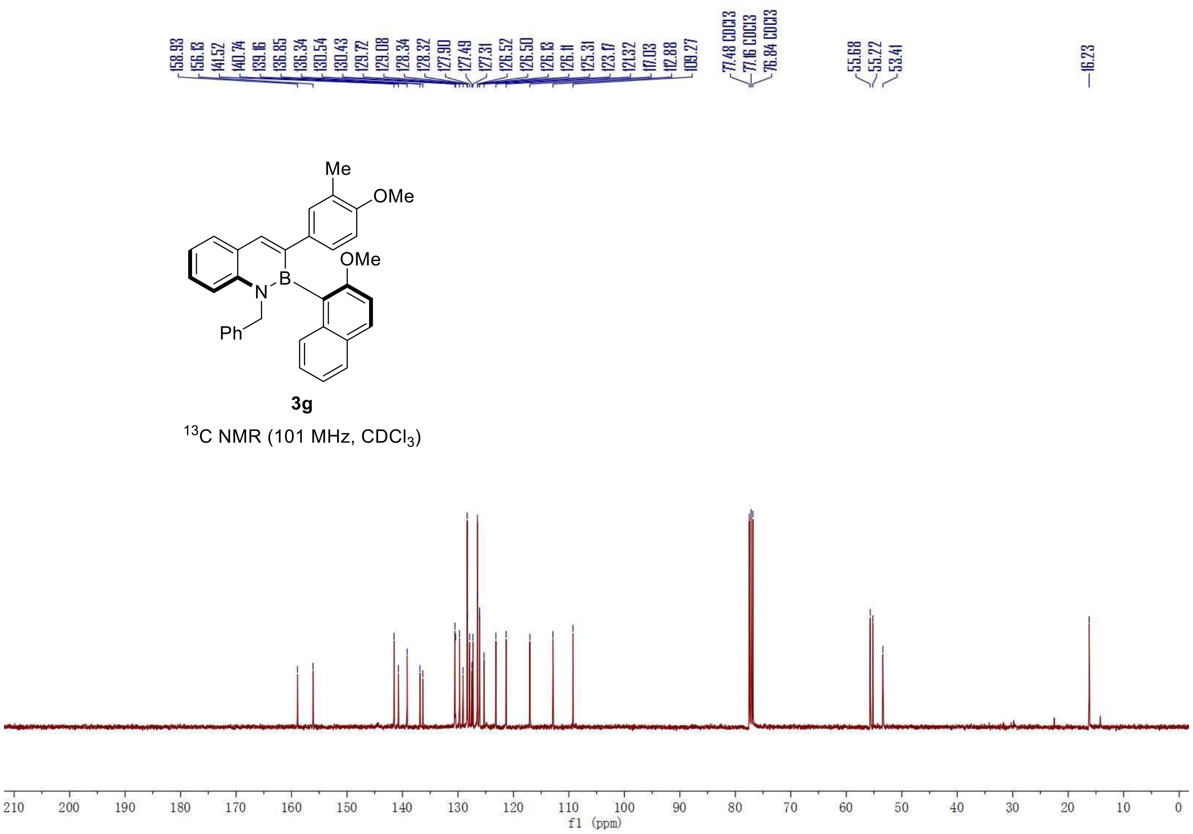

Supplementary Figure 117. ^{13}C NMR spectrum of 3g

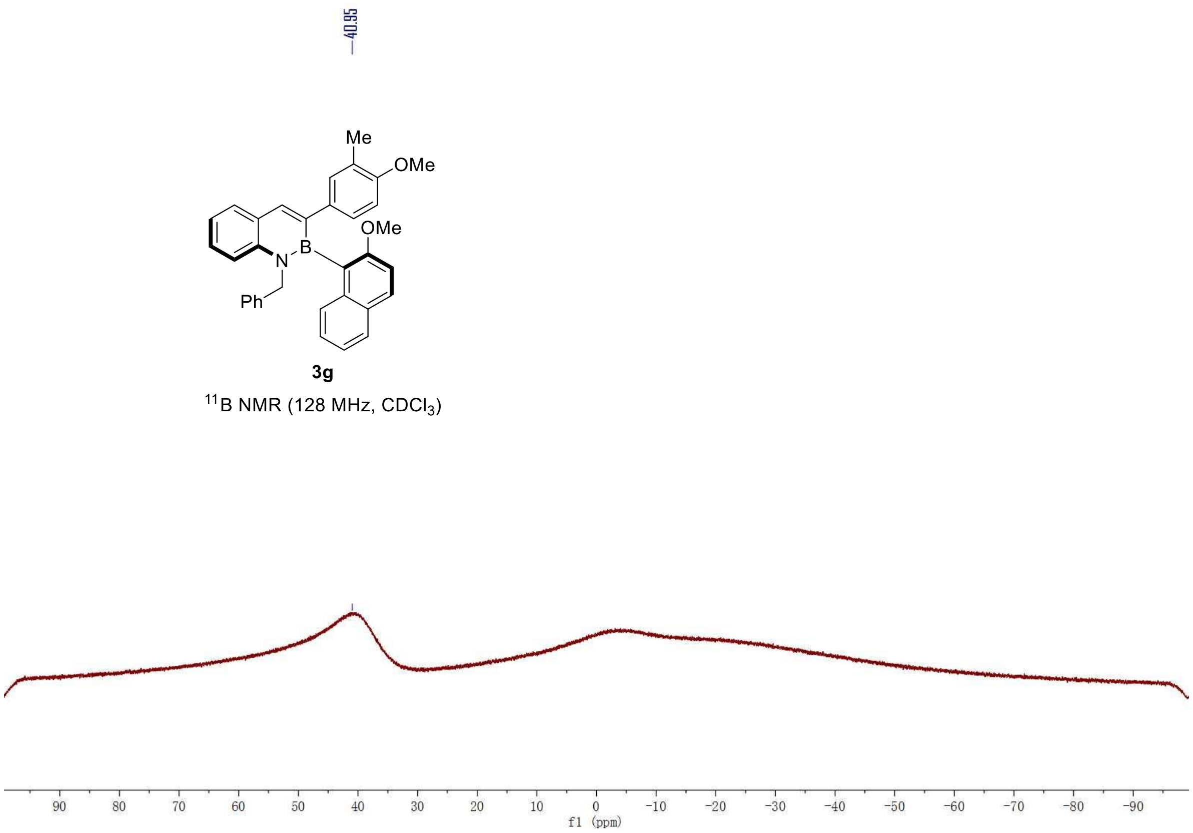

Supplementary Figure 118. ^{11}B NMR spectrum of 3g

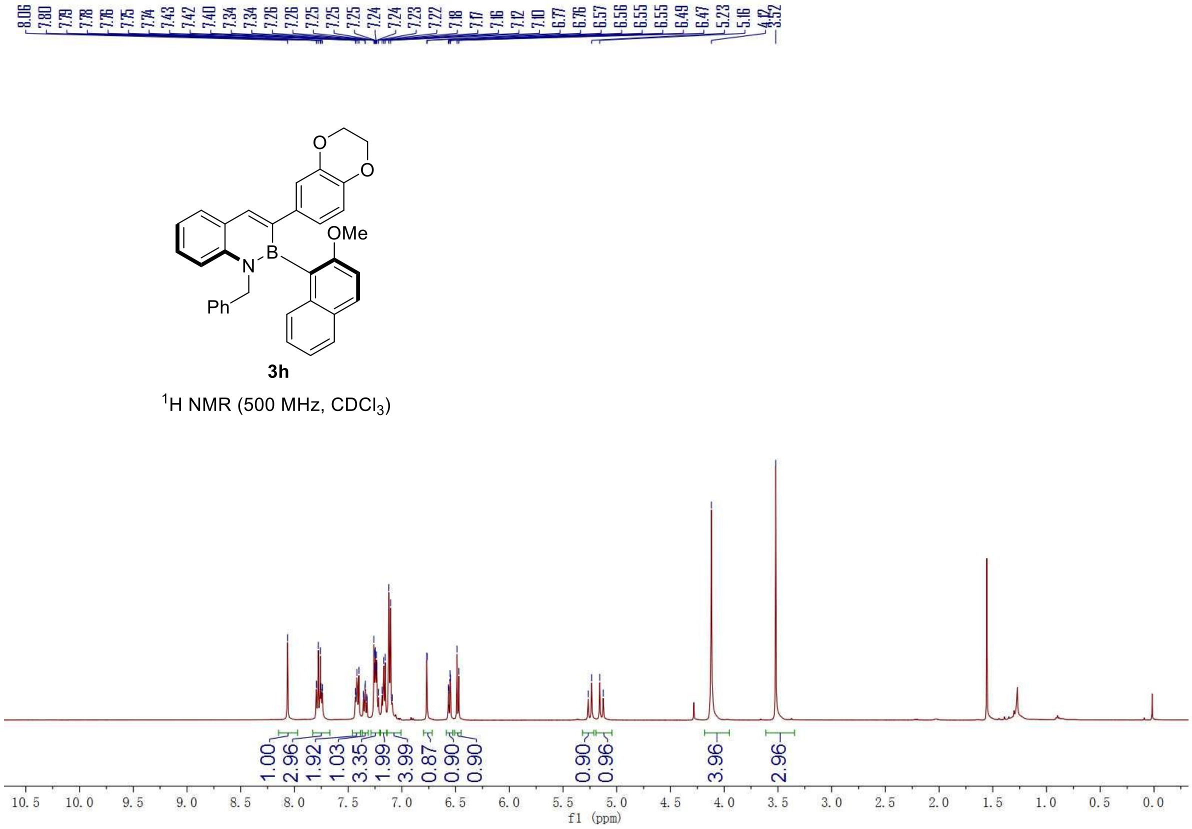

Supplementary Figure 119. ^{1}H NMR spectrum of 3h

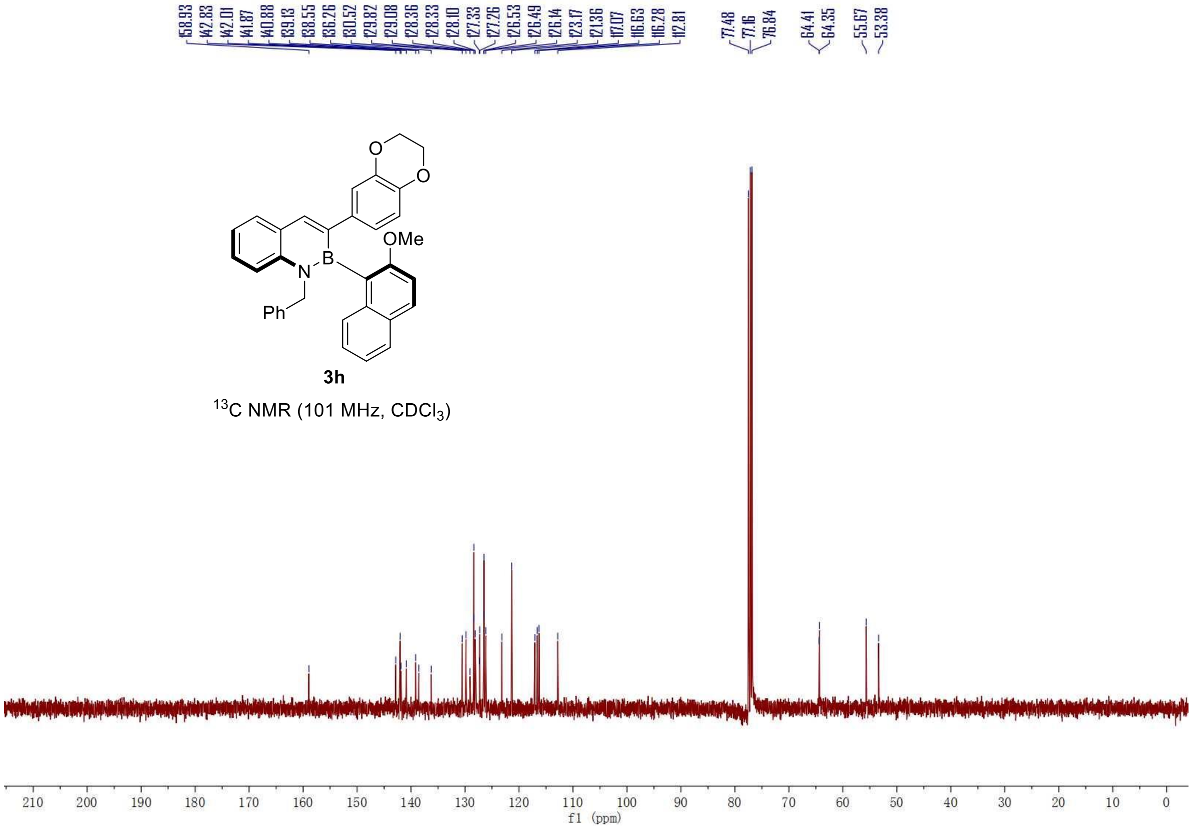

Supplementary Figure 120. ^{13}C NMR spectrum of 3h

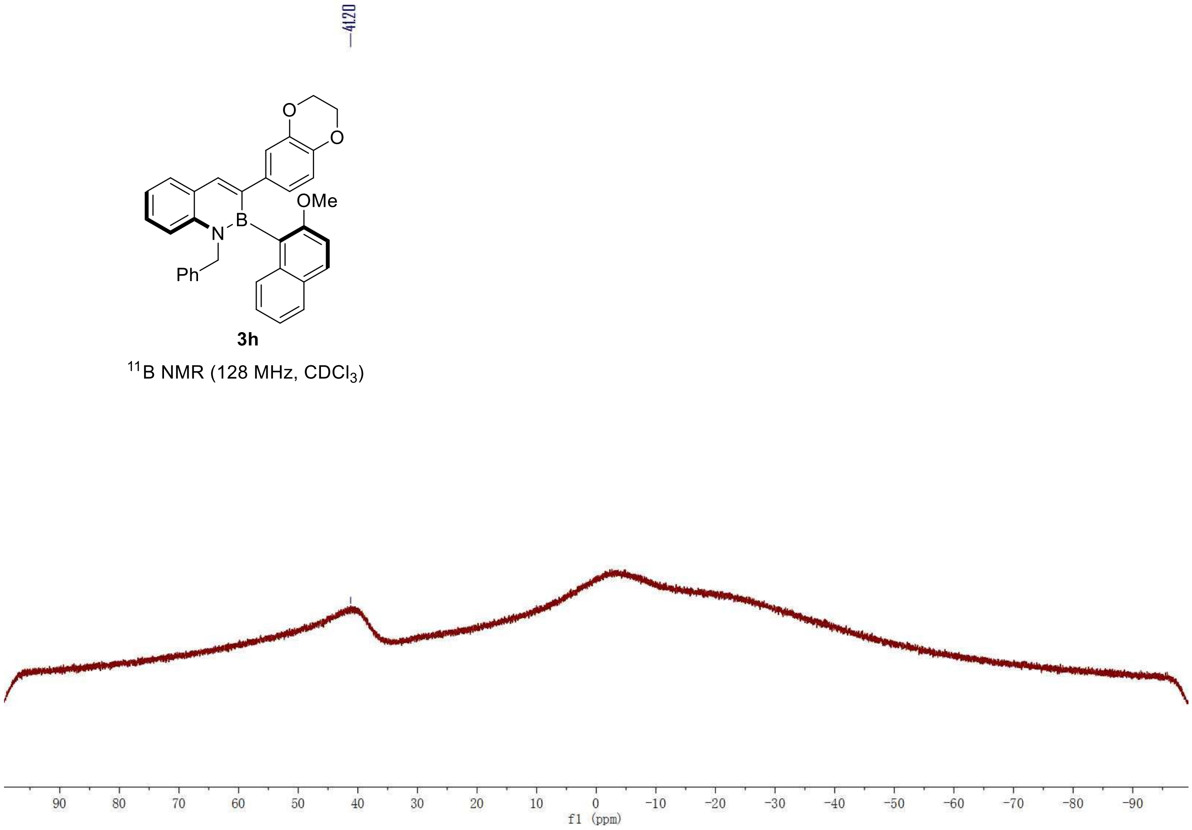

Supplementary Figure 121. ^{11}B NMR spectrum of 3h

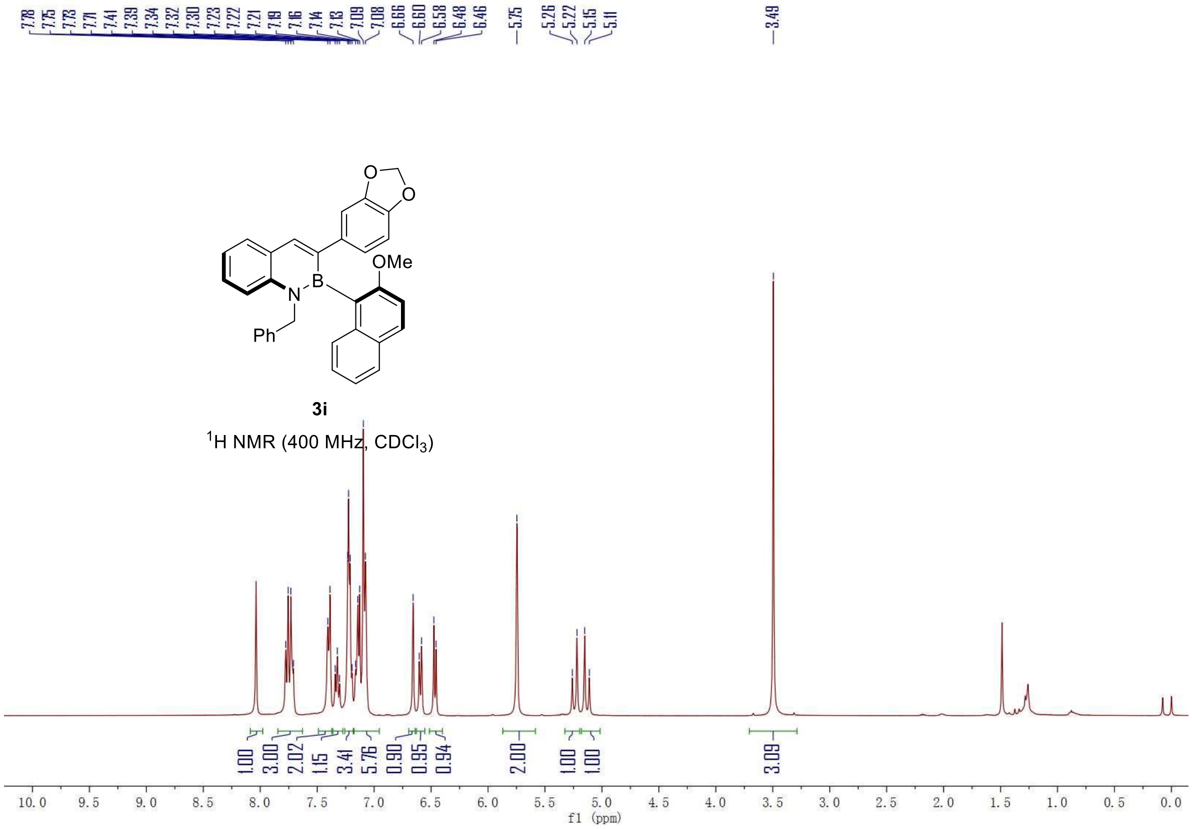

Supplementary Figure 122. ^{1}H NMR spectrum of 3i

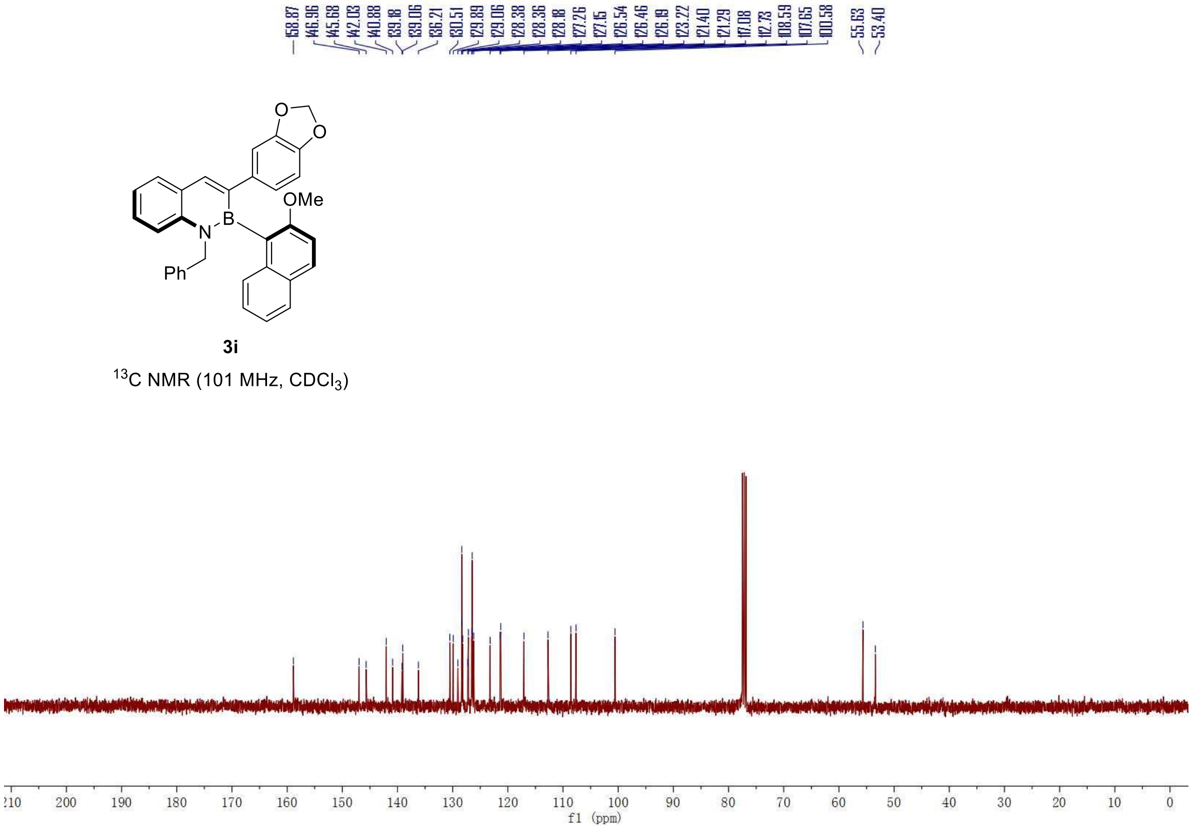

Supplementary Figure 123. ^{13}C NMR spectrum of 3i

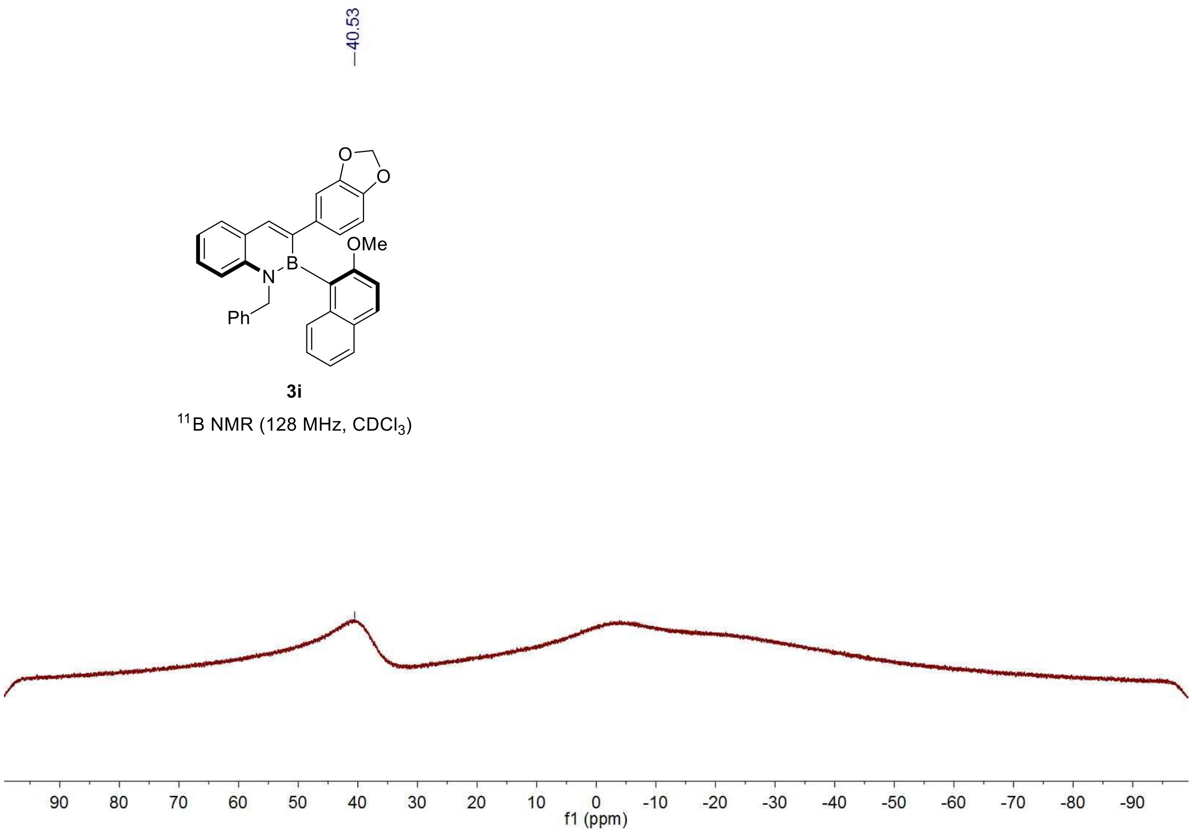

Supplementary Figure 124. ^{11}B NMR spectrum of 3i

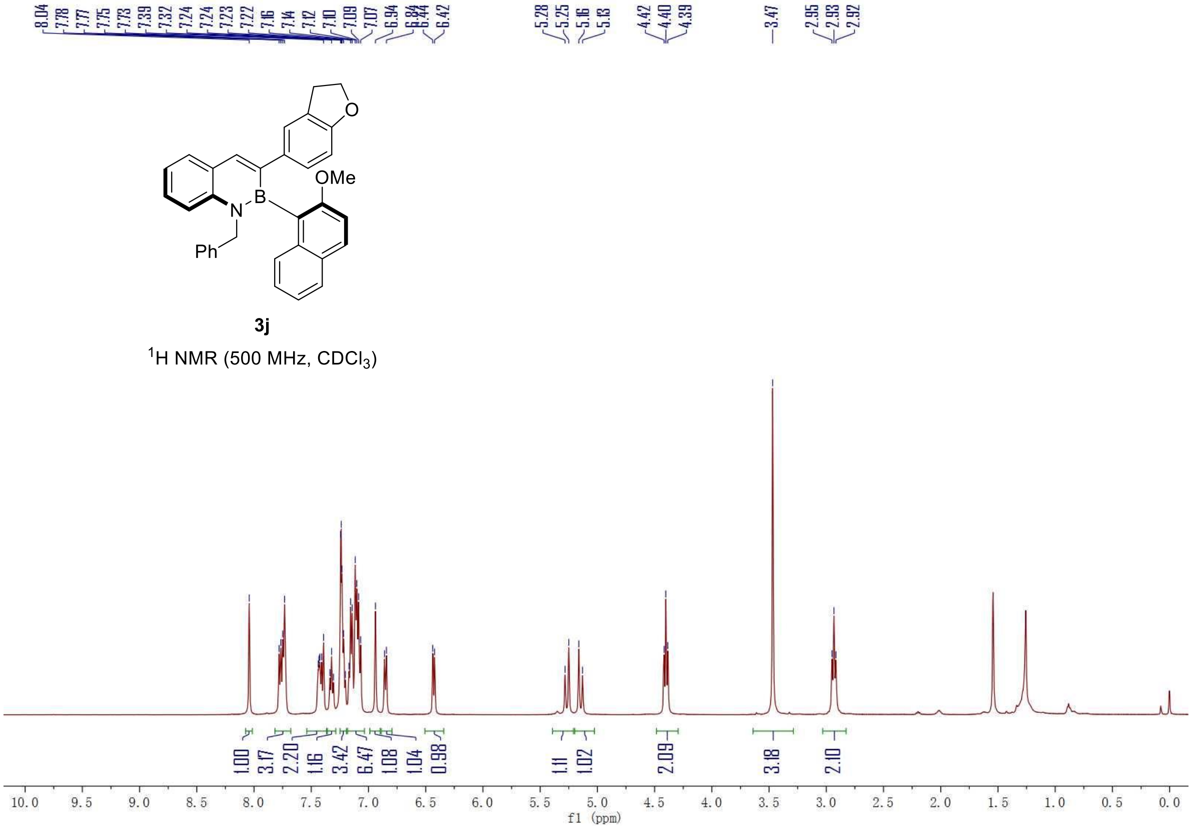

Supplementary Figure 125. ^{1}H NMR spectrum of 3j

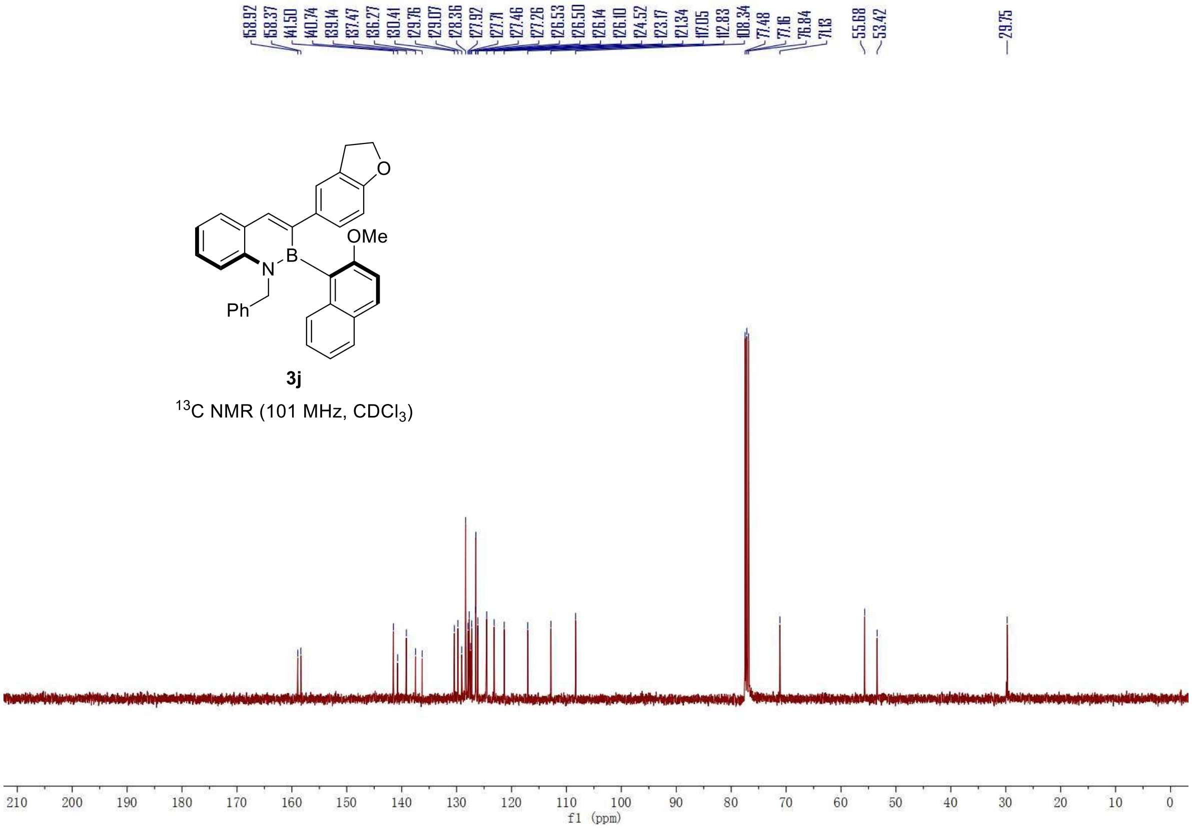

Supplementary Figure 126. ^{13}C NMR spectrum of 3j

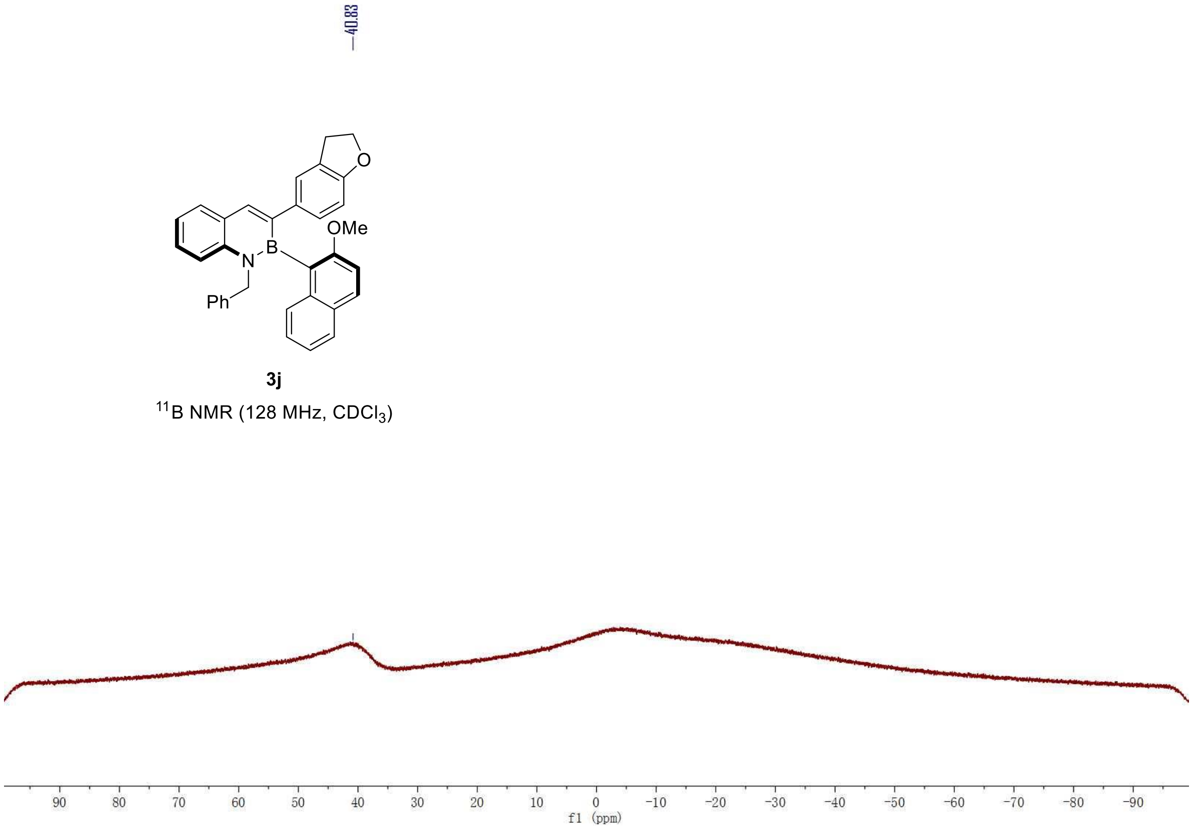

Supplementary Figure 127. ^{11}B NMR spectrum of 3j

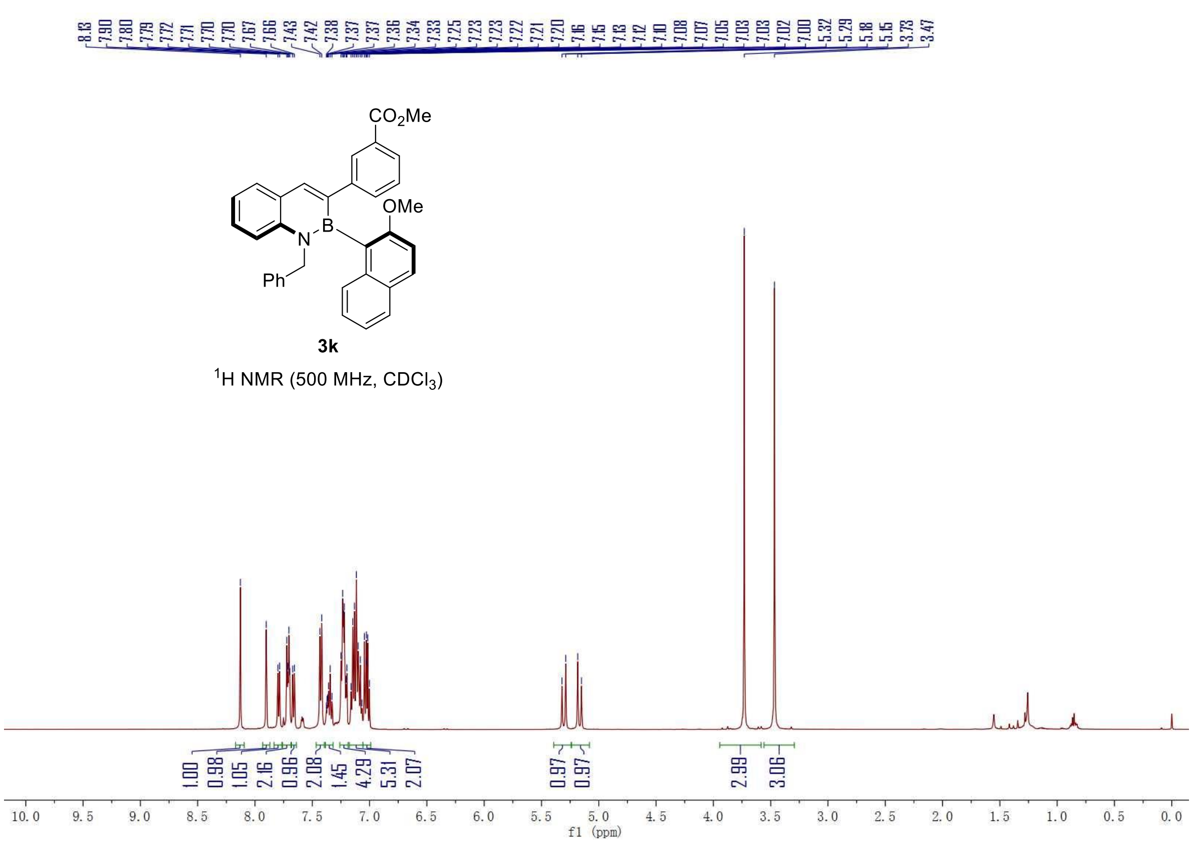

Supplementary Figure 128. ^{1}H NMR spectrum of 3k

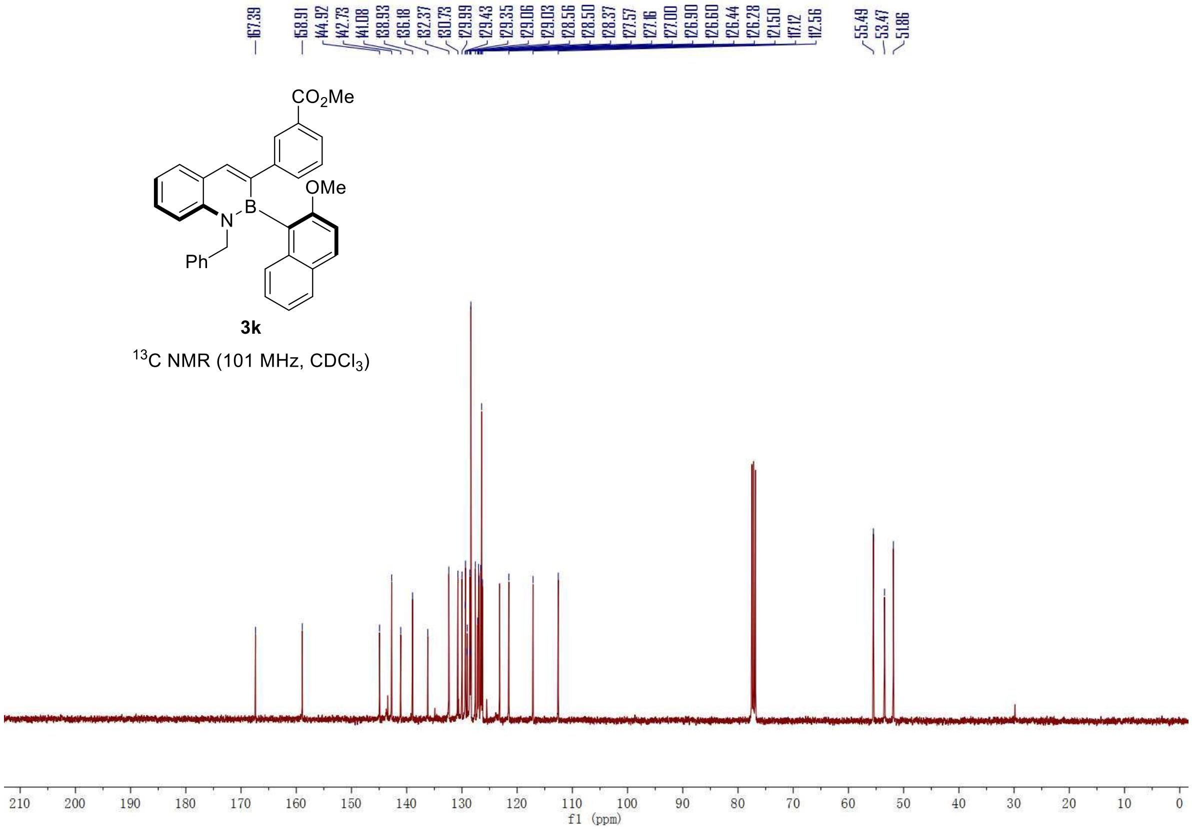

Supplementary Figure 129. ^{13}C NMR spectrum of 3k

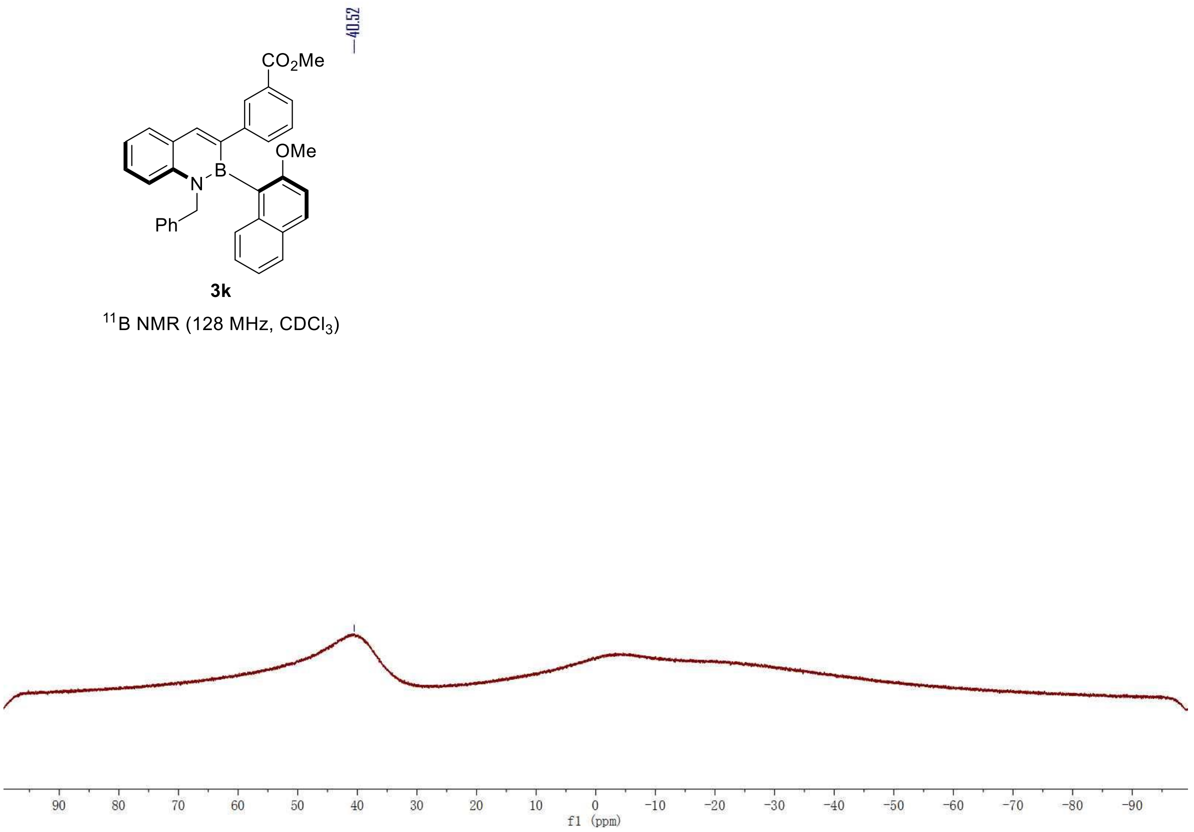

Supplementary Figure 130. ^{11}B NMR spectrum of 3k

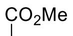

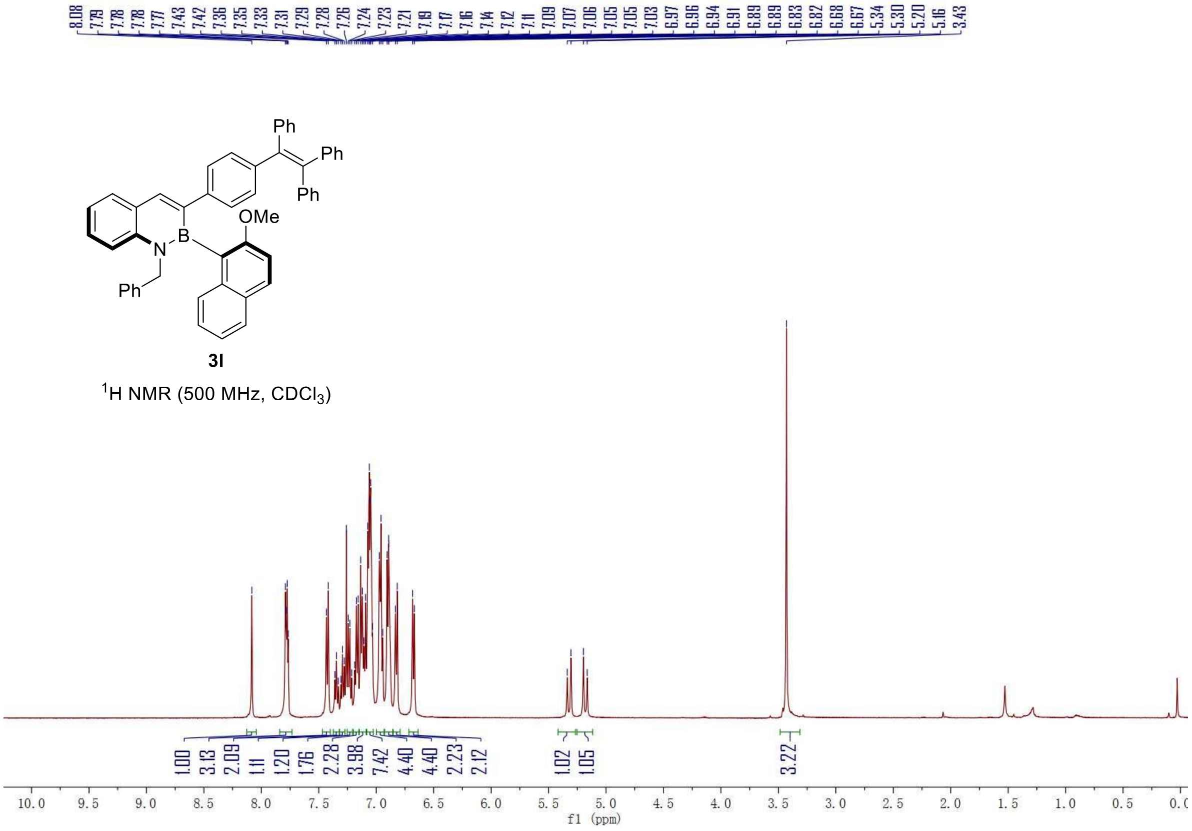

Supplementary Figure 131. ^{1}H NMR spectrum of 3l

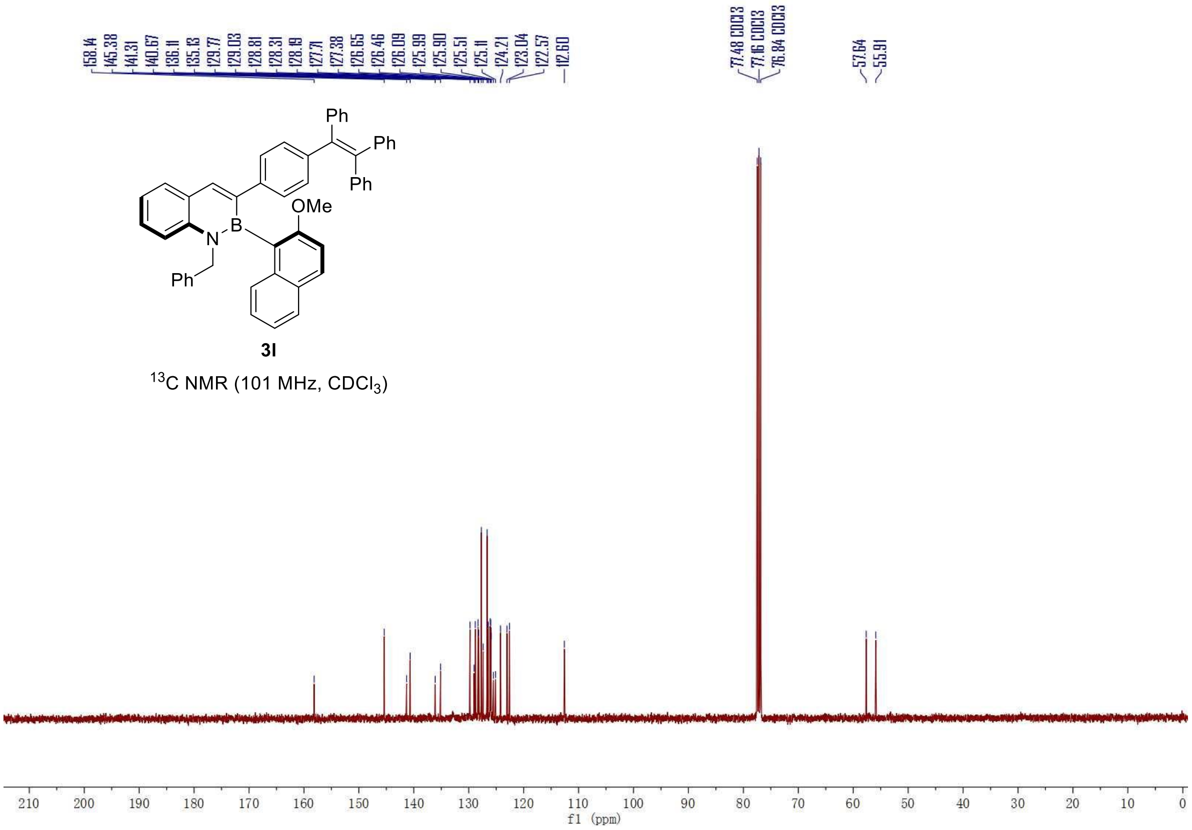

Supplementary Figure 132. ^{13}C NMR spectrum of 3l

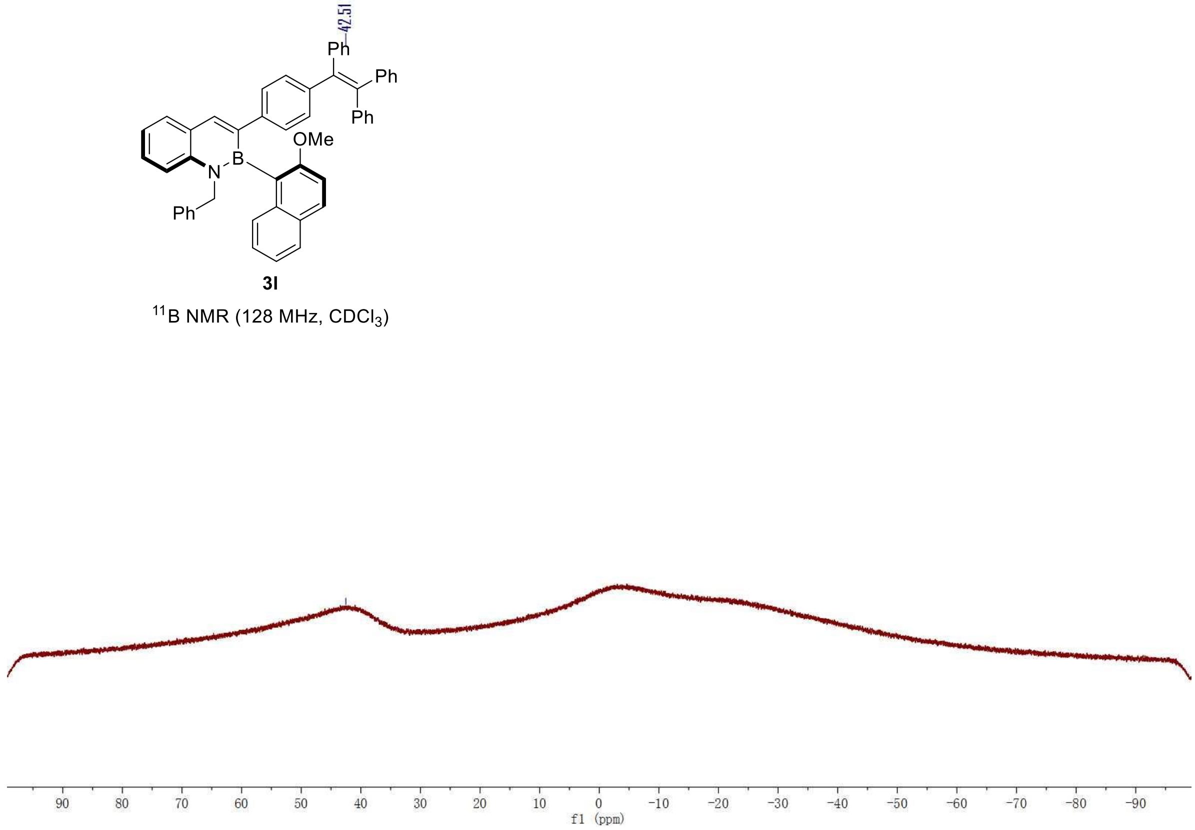

Supplementary Figure 133. ^{11}B NMR spectrum of 3l

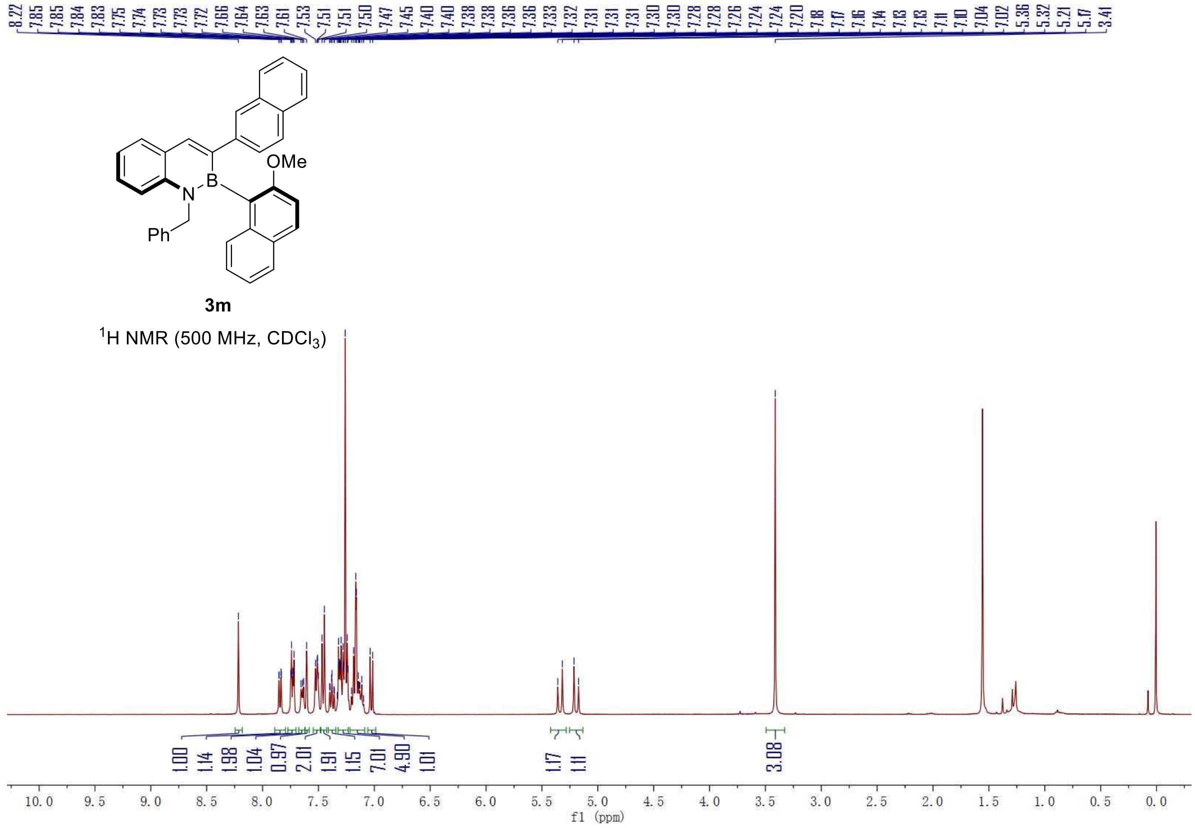

Supplementary Figure 134. ^{1}H NMR spectrum of 3m

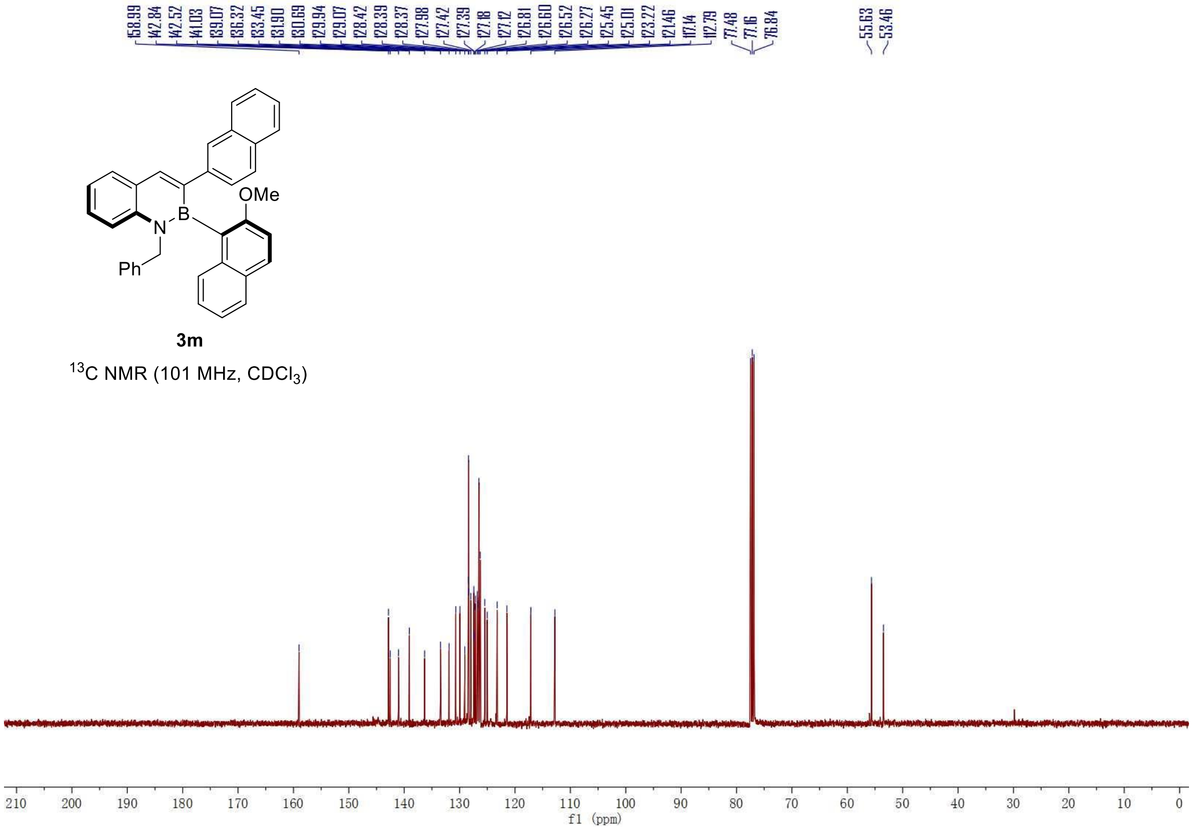

Supplementary Figure 135. ^{13}C NMR spectrum of 3m

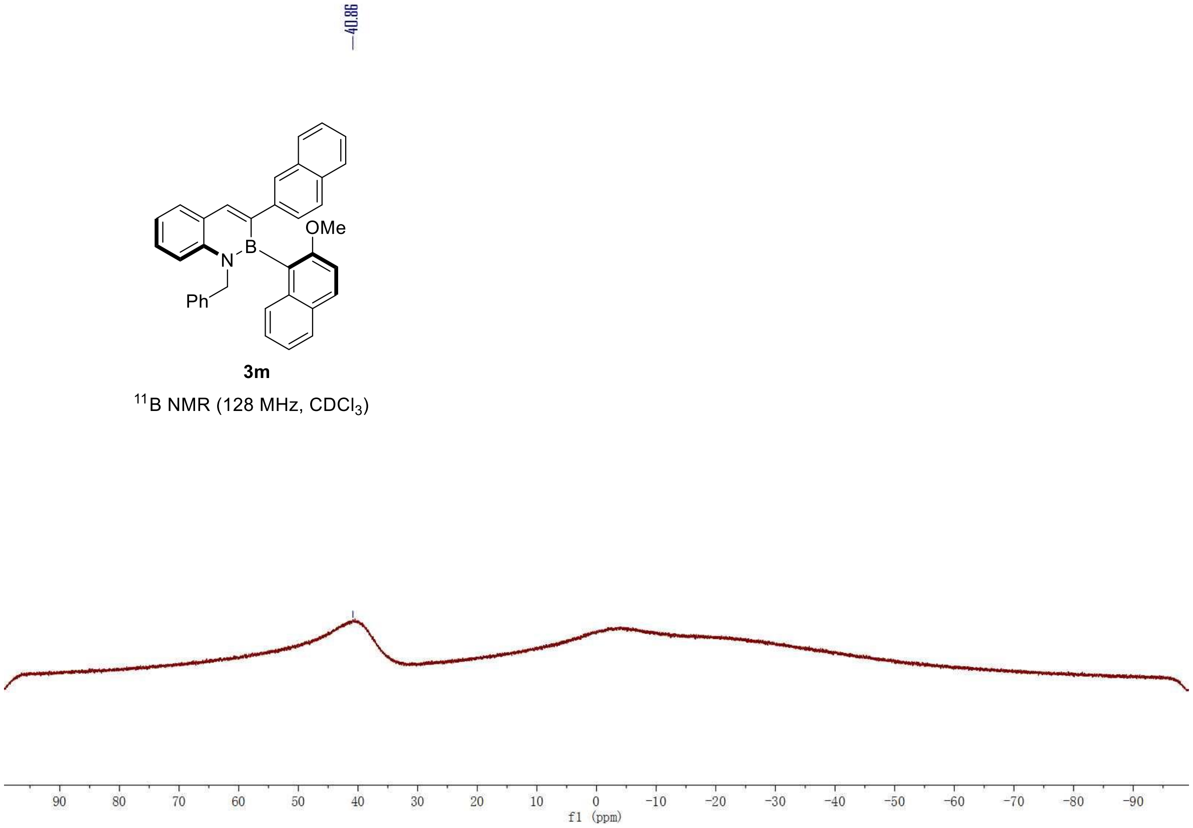

Supplementary Figure 136. ^{11}B NMR spectrum of 3m

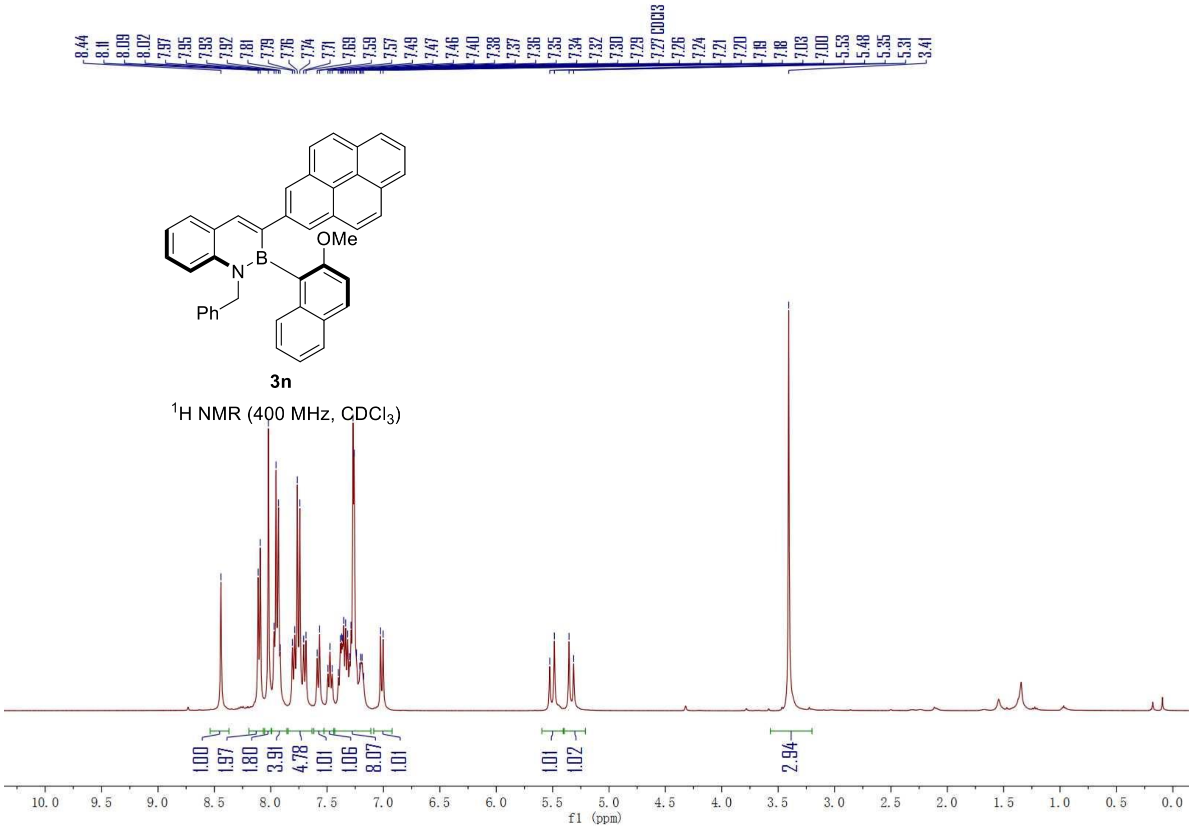

Supplementary Figure 137. ^{1}H NMR spectrum of 3n

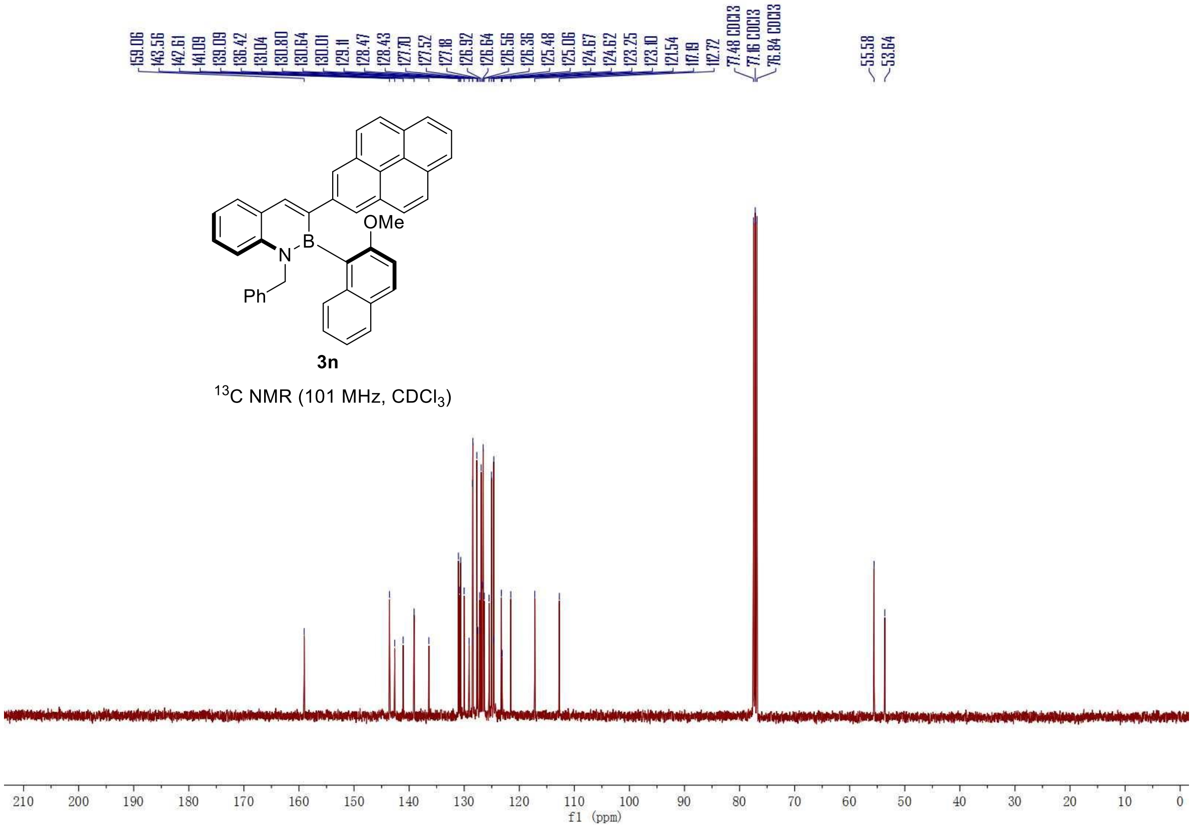

Supplementary Figure 138. ^{13}C NMR spectrum of 3n

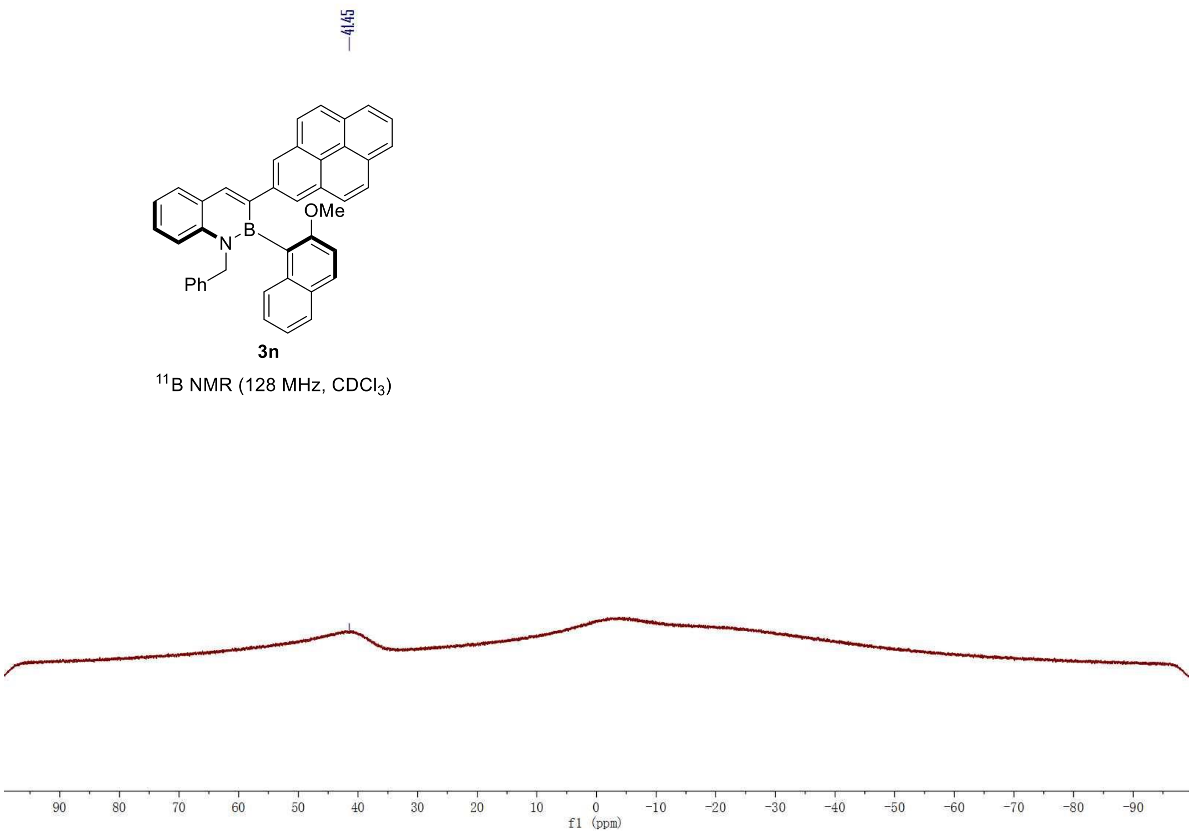

Supplementary Figure 139. ^{11}B NMR spectrum of 3n

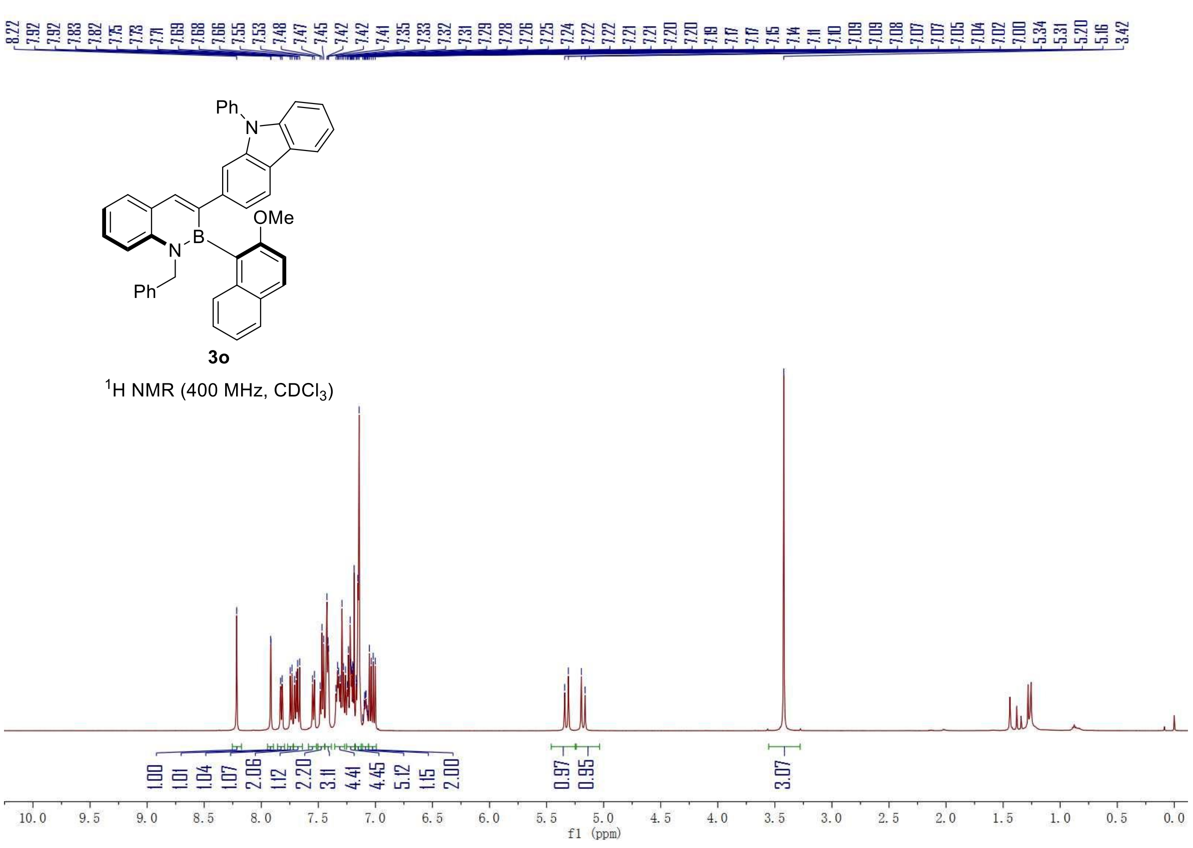

Supplementary Figure 140. ^{1}H NMR spectrum of 3o

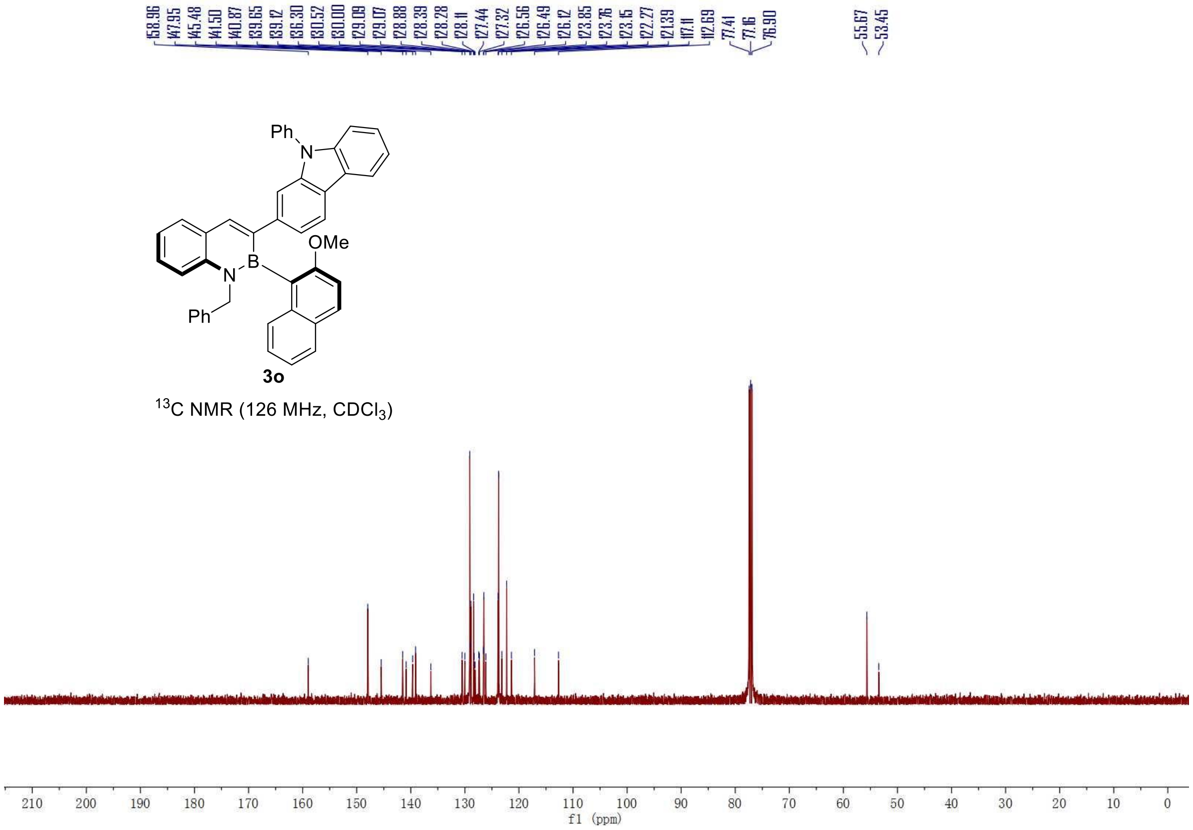

Supplementary Figure 141. ^{13}C NMR spectrum of 3o

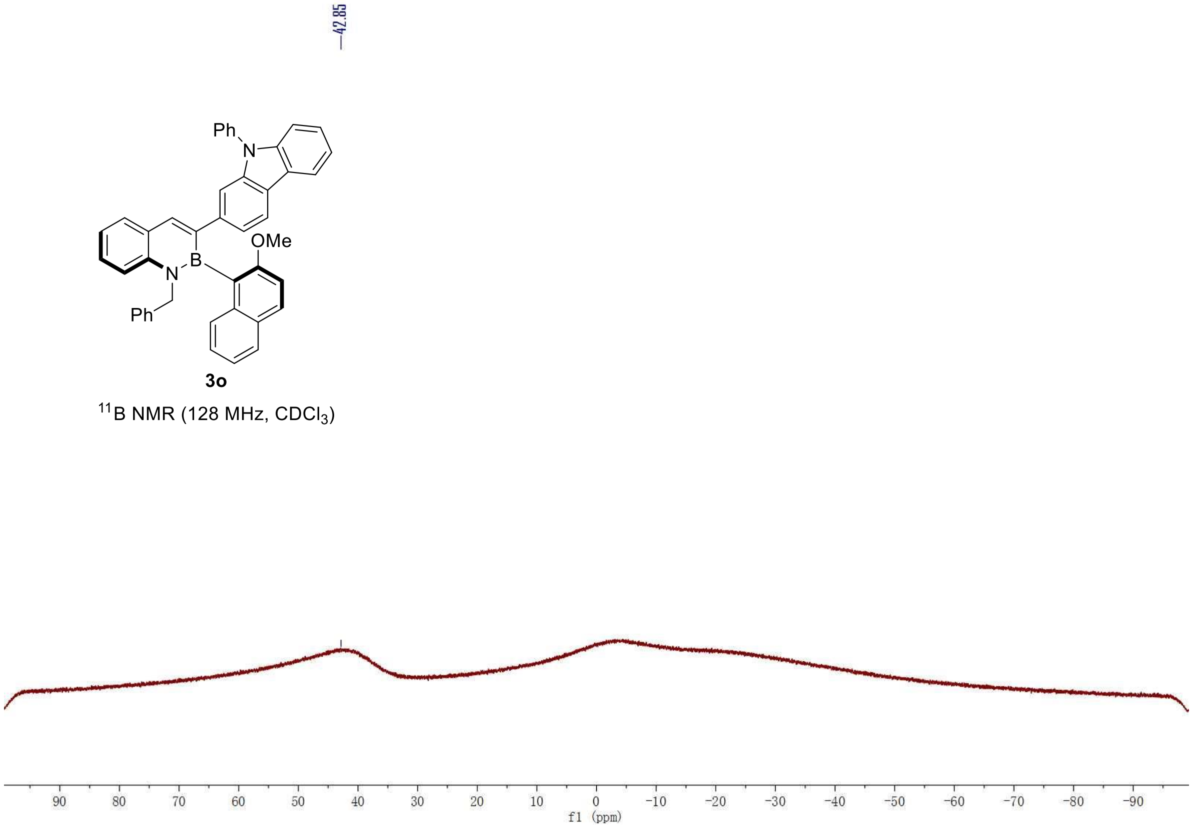

Supplementary Figure 142. ^{11}B NMR spectrum of 3o

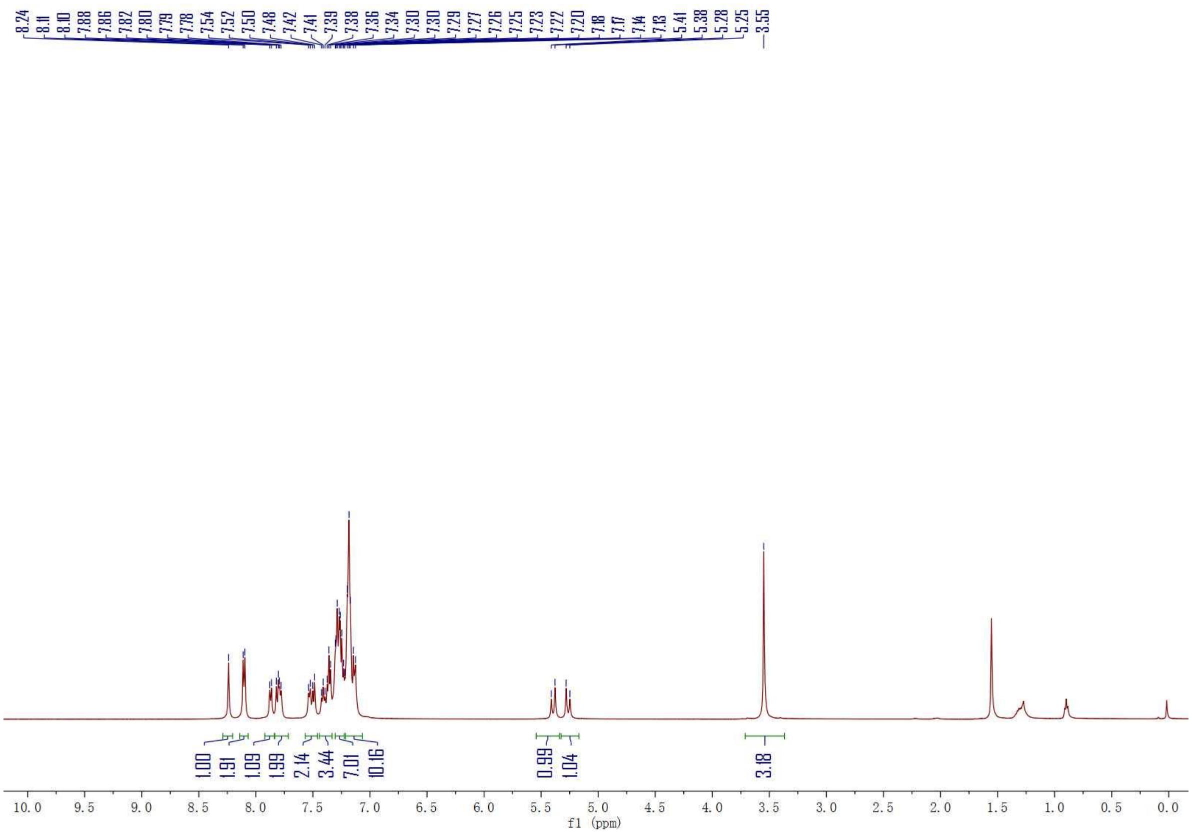

Supplementary Figure 143. ^{1}H NMR spectrum of 1a

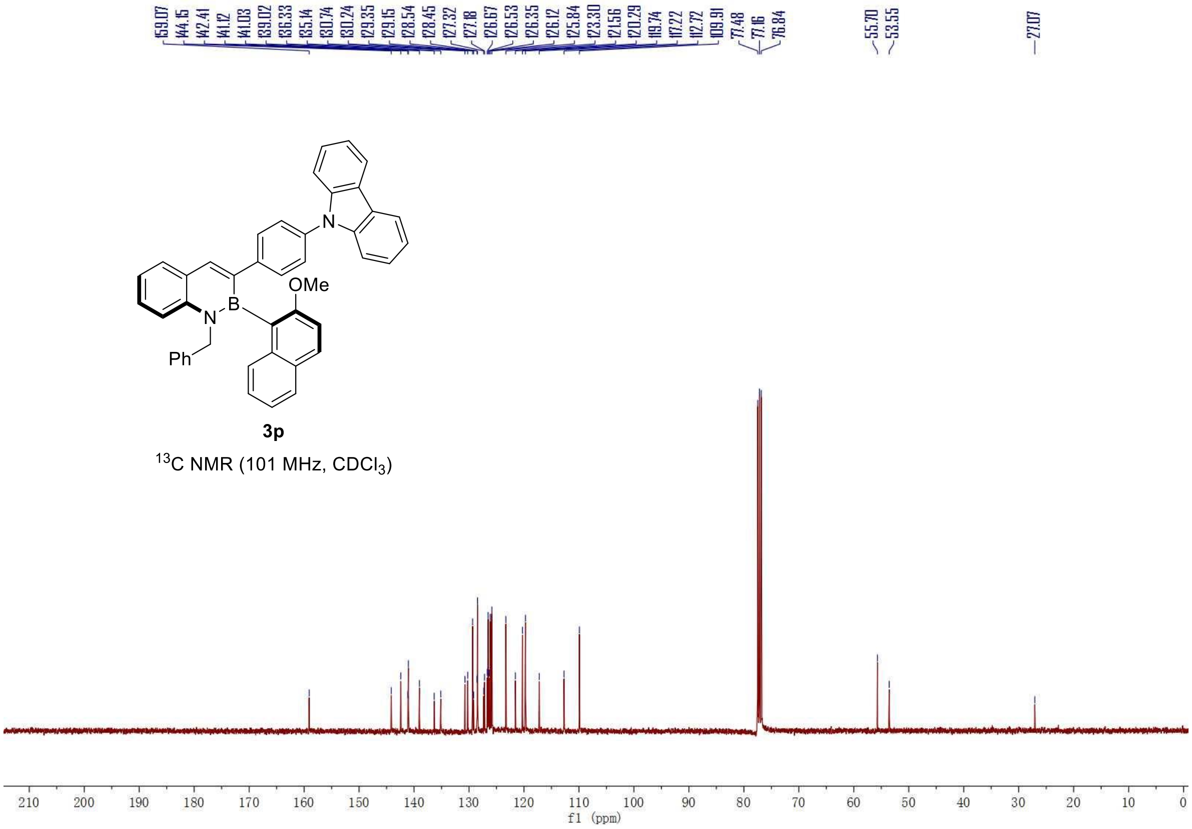

Supplementary Figure 144. ^{13}C NMR spectrum of 3p

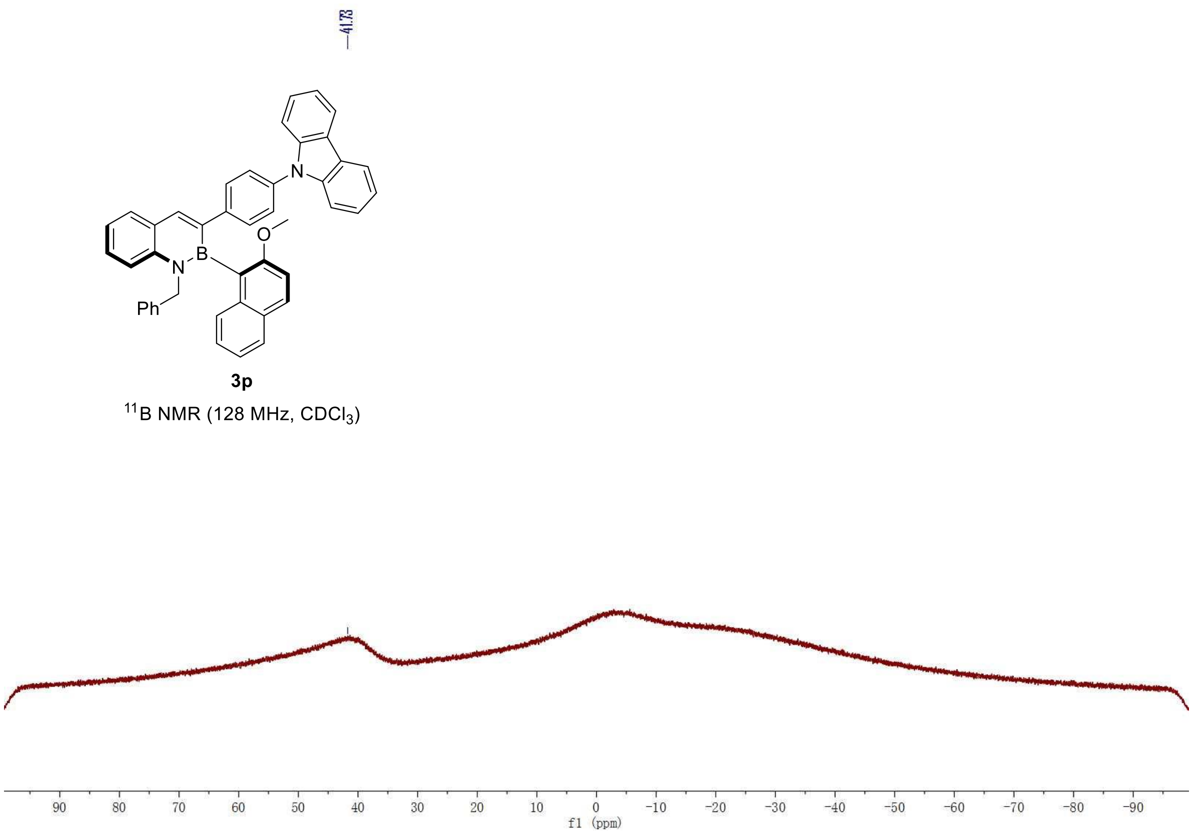

Supplementary Figure 145. ^{11}B NMR spectrum of 3p

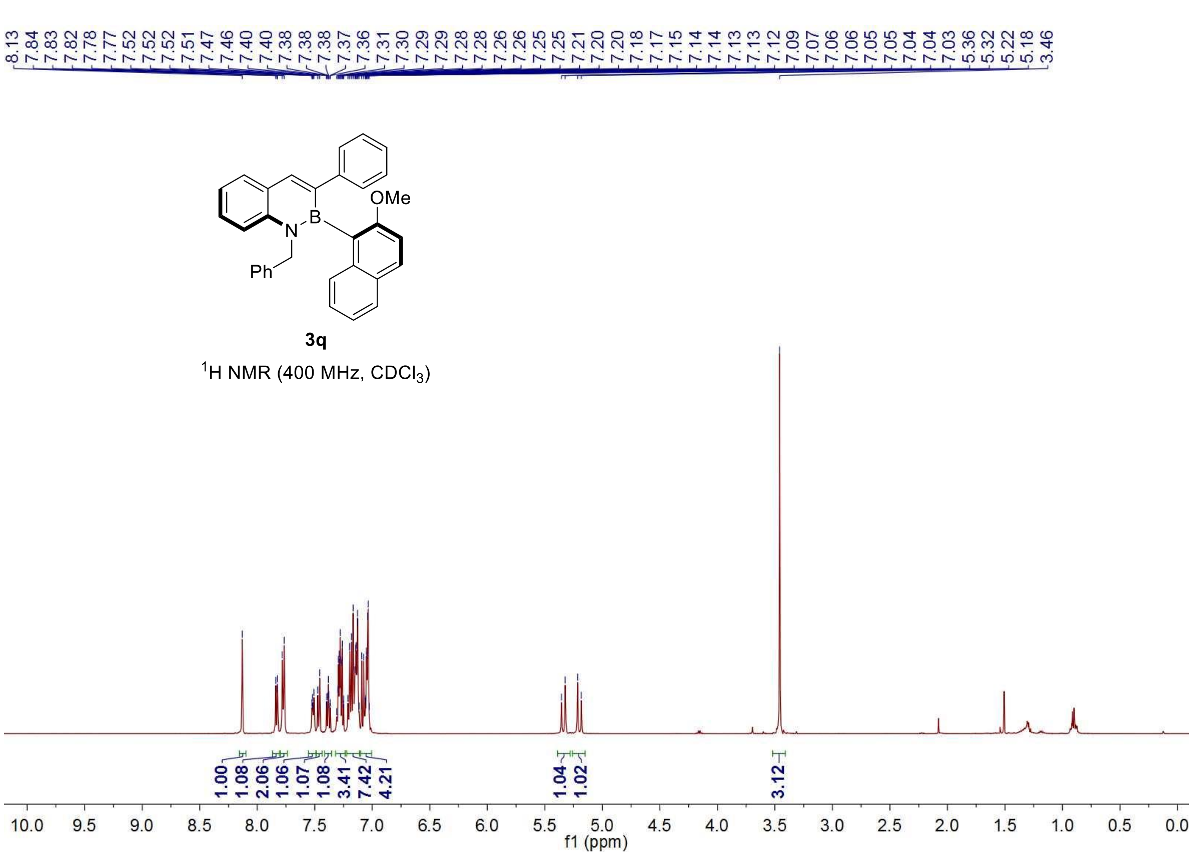

Supplementary Figure 146. ^{1}H NMR spectrum of 3q

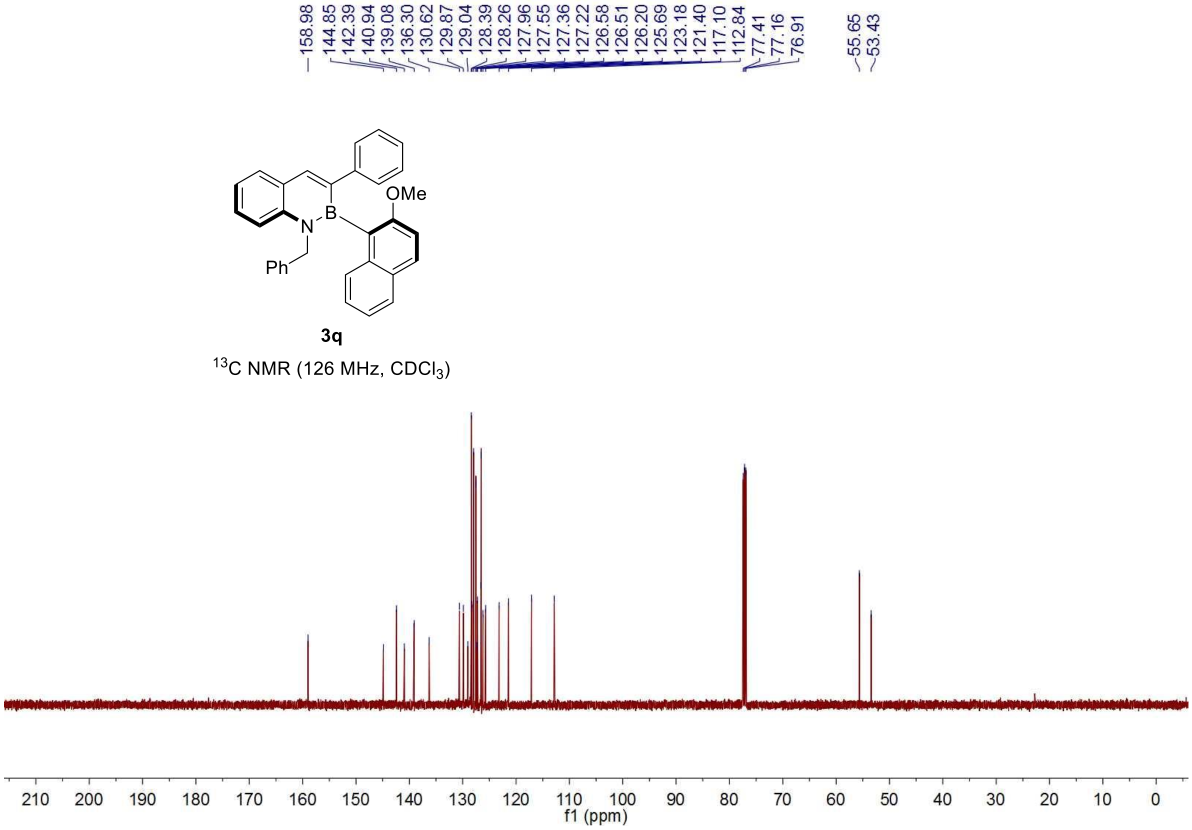

Supplementary Figure 147. ^{13}C NMR spectrum of 3q

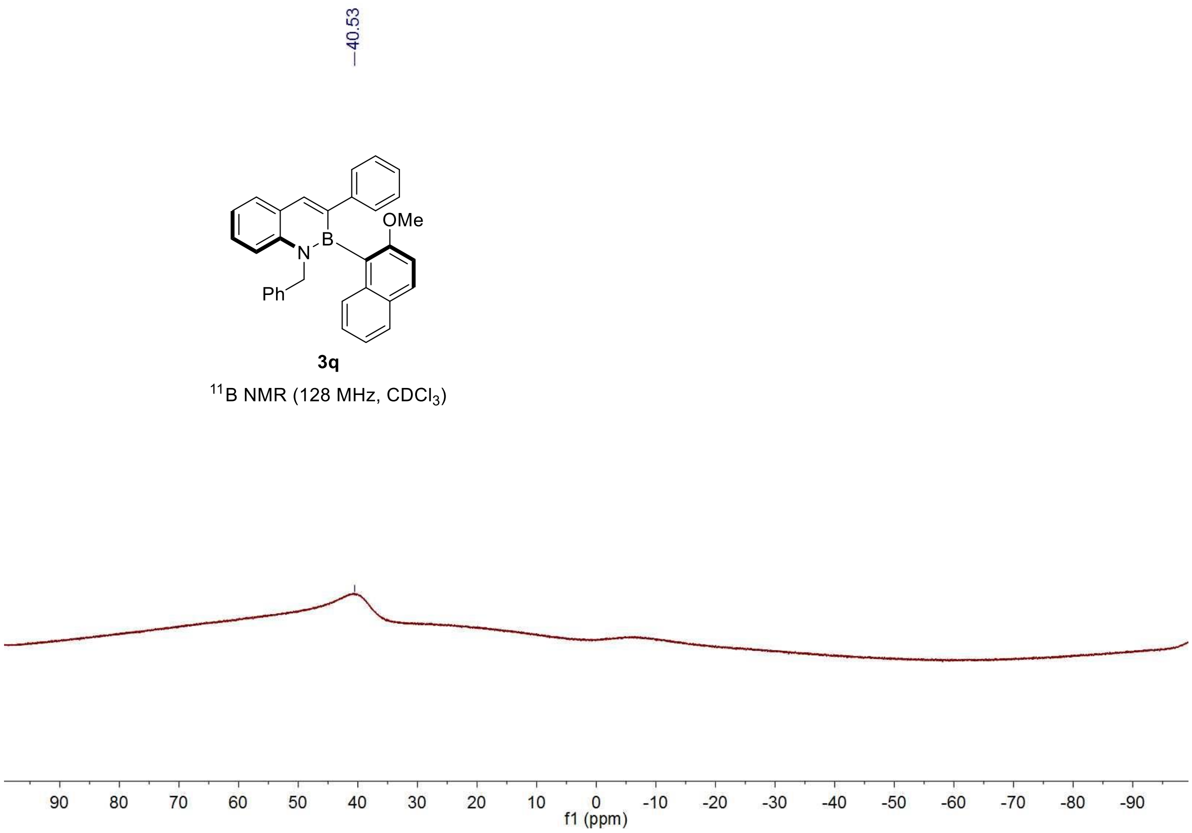

Supplementary Figure 148. ^{11}B NMR spectrum of 3q

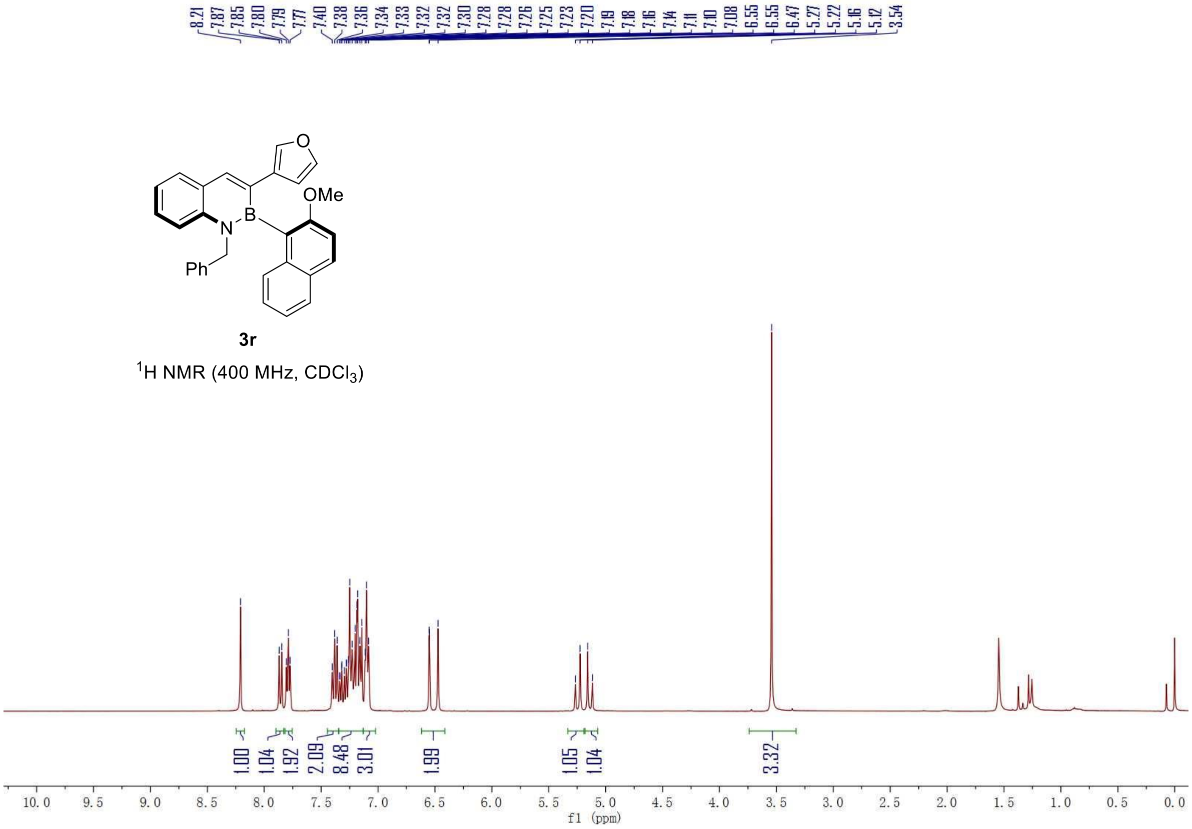

Supplementary Figure 149. ^{1}H NMR spectrum of 3r

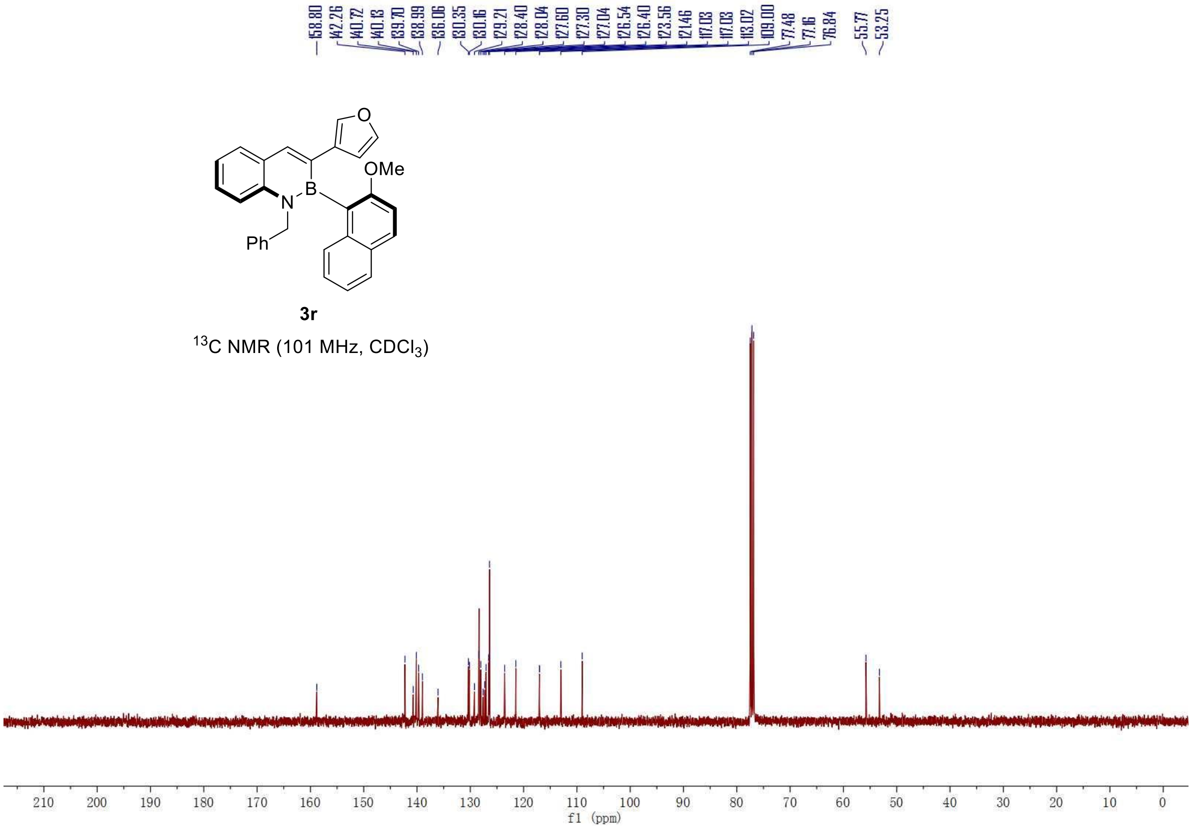

Supplementary Figure 150. ^{13}C NMR spectrum of 3r

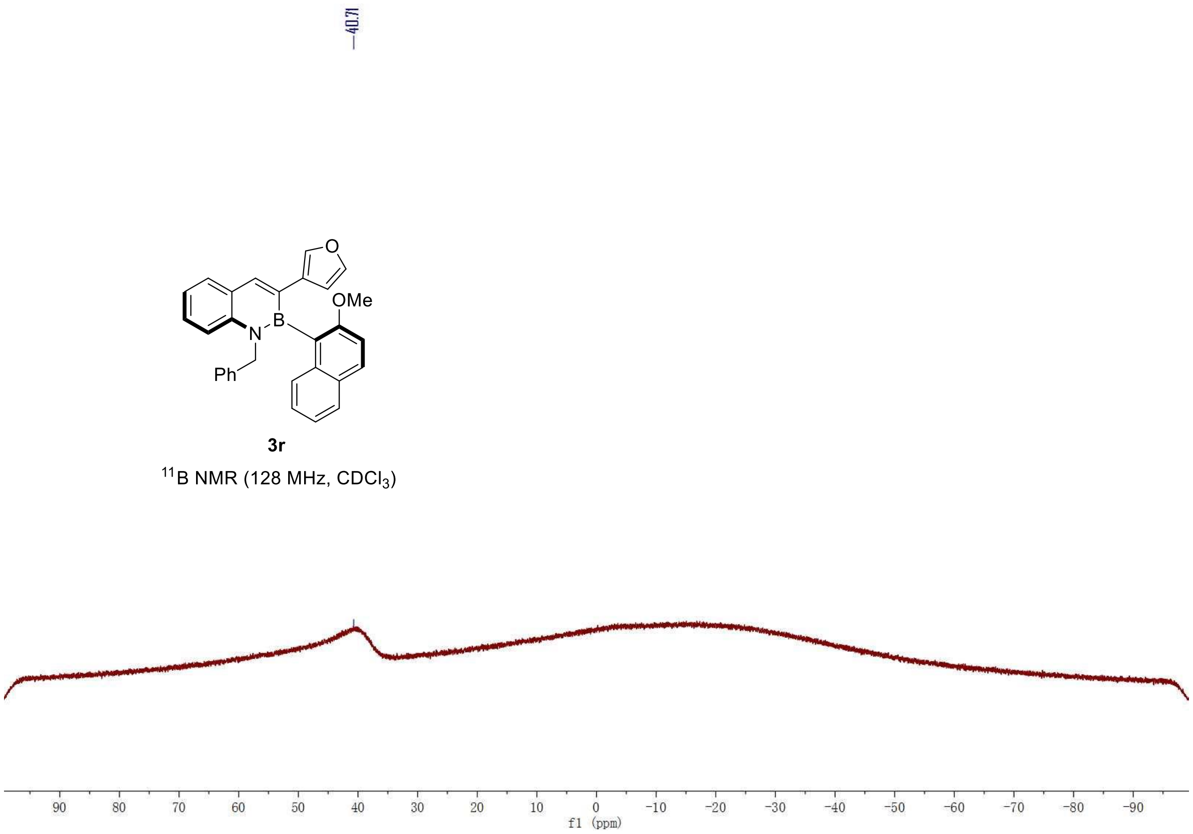

Supplementary Figure 151. ^{11}B NMR spectrum of 3r

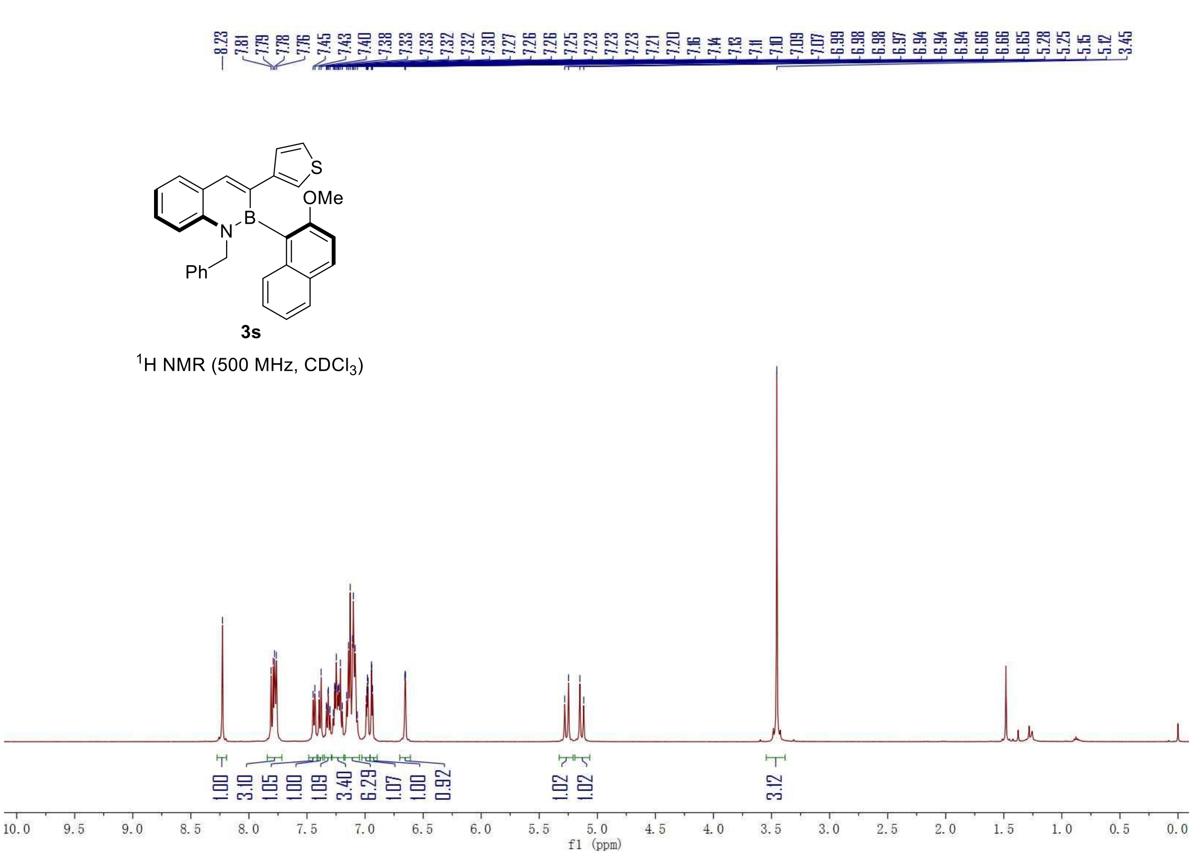

Supplementary Figure 152. ^{1}H NMR spectrum of 3s

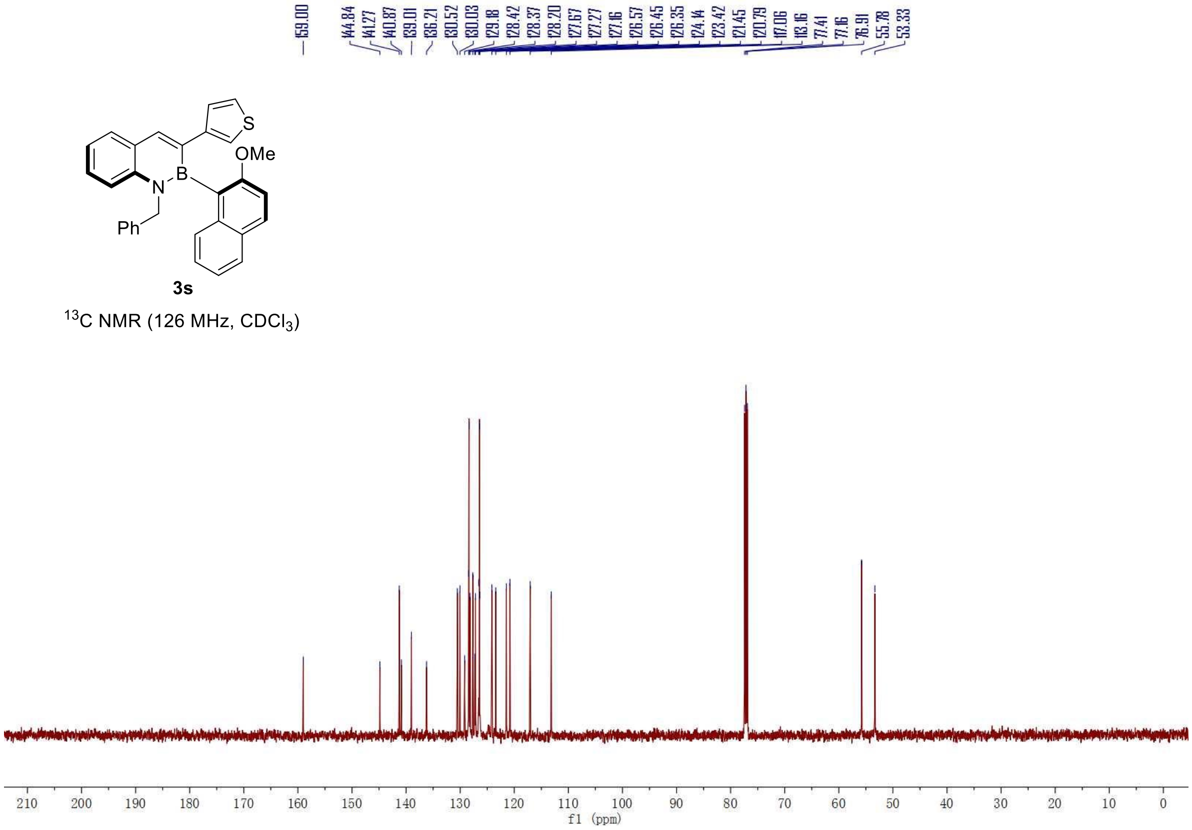

Supplementary Figure 153. ^{13}C NMR spectrum of 3s

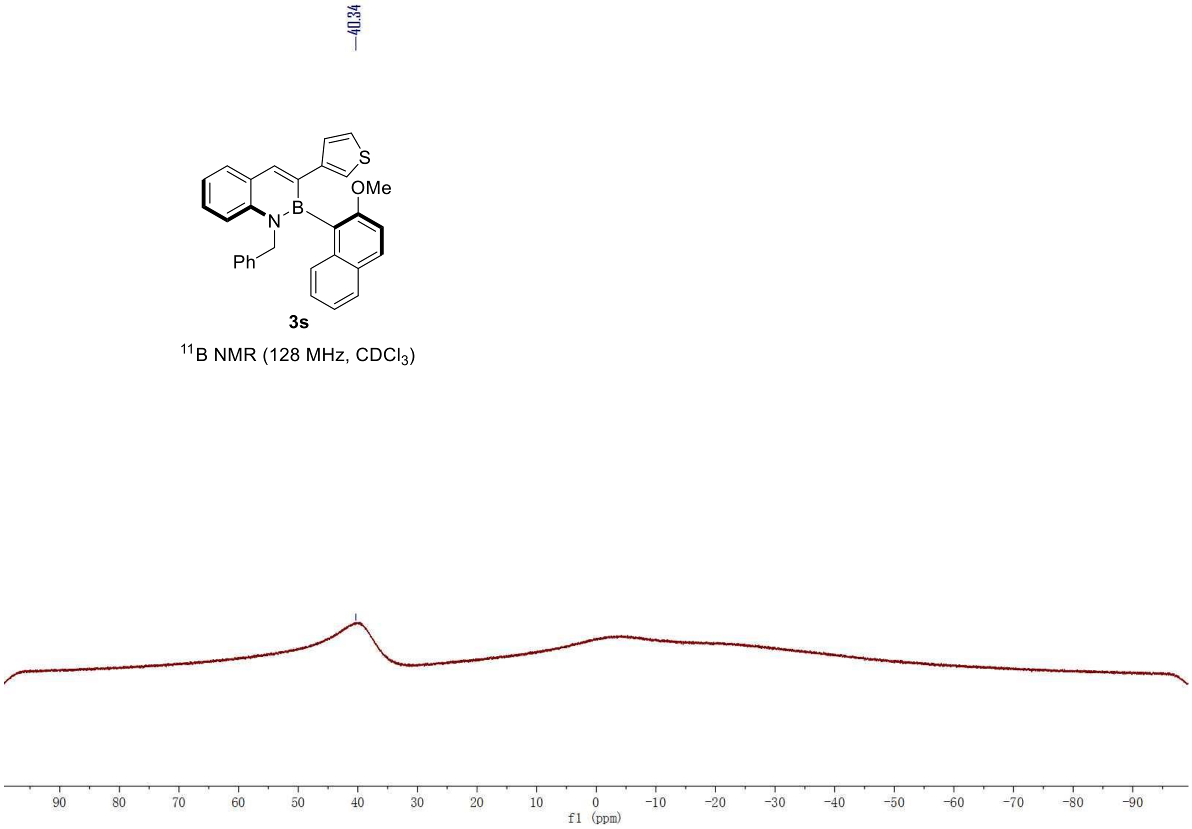

Supplementary Figure 154. ^{11}B NMR spectrum of 3s

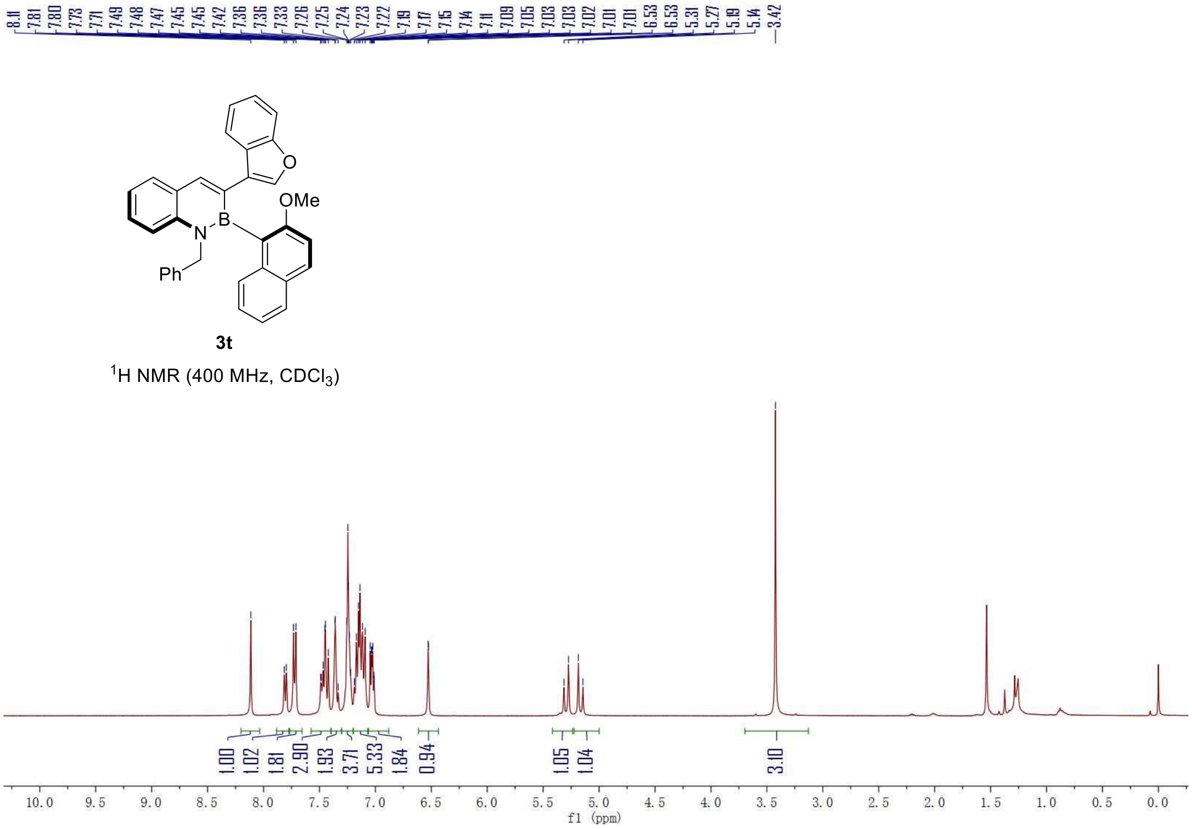

Supplementary Figure 155. ^{1}H NMR spectrum of 3t

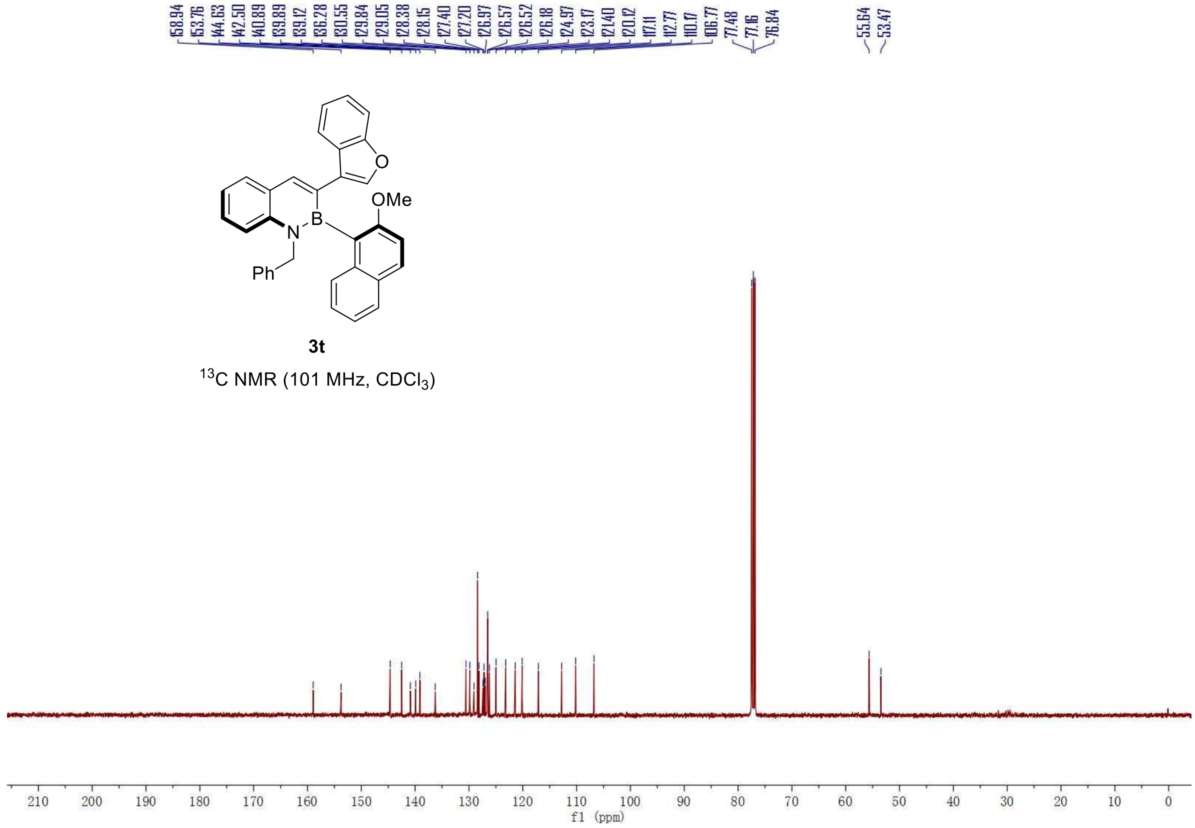

Supplementary Figure 156. ^{13}C NMR spectrum of 3t

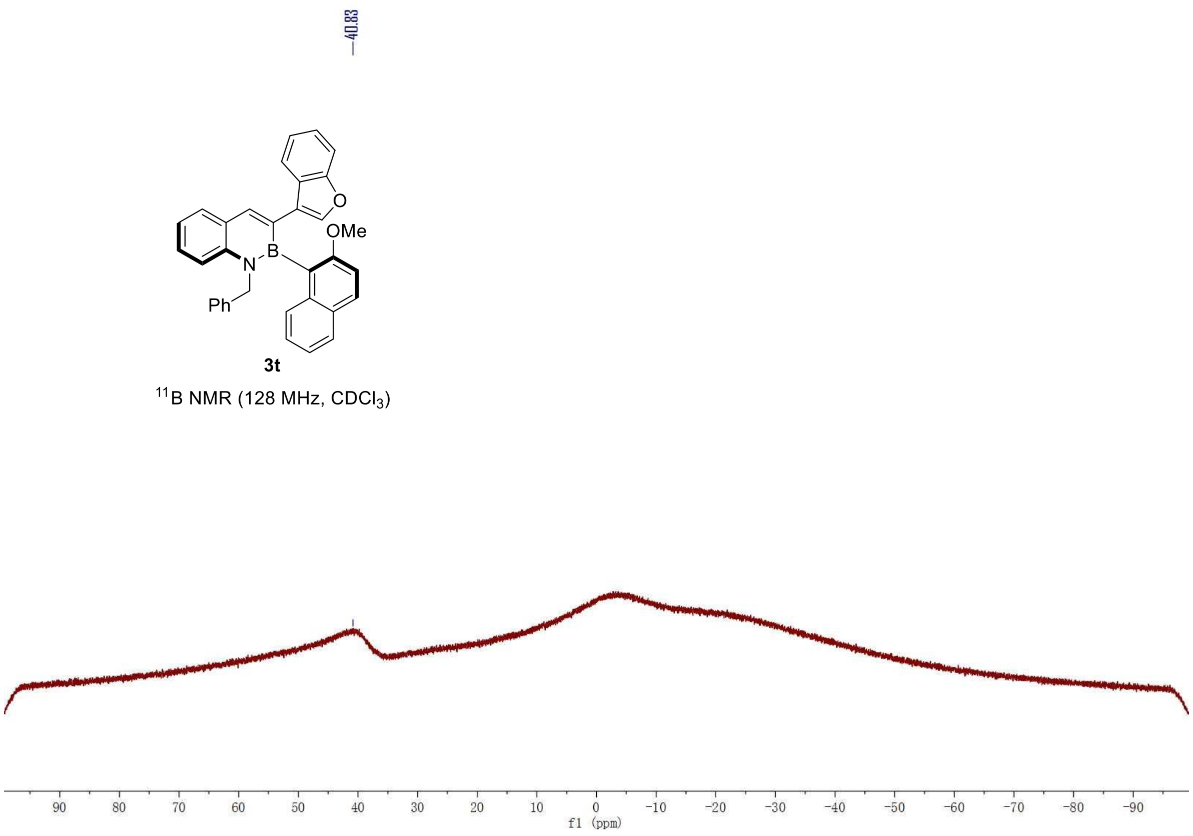

Supplementary Figure 157. ^{11}B NMR spectrum of 3t

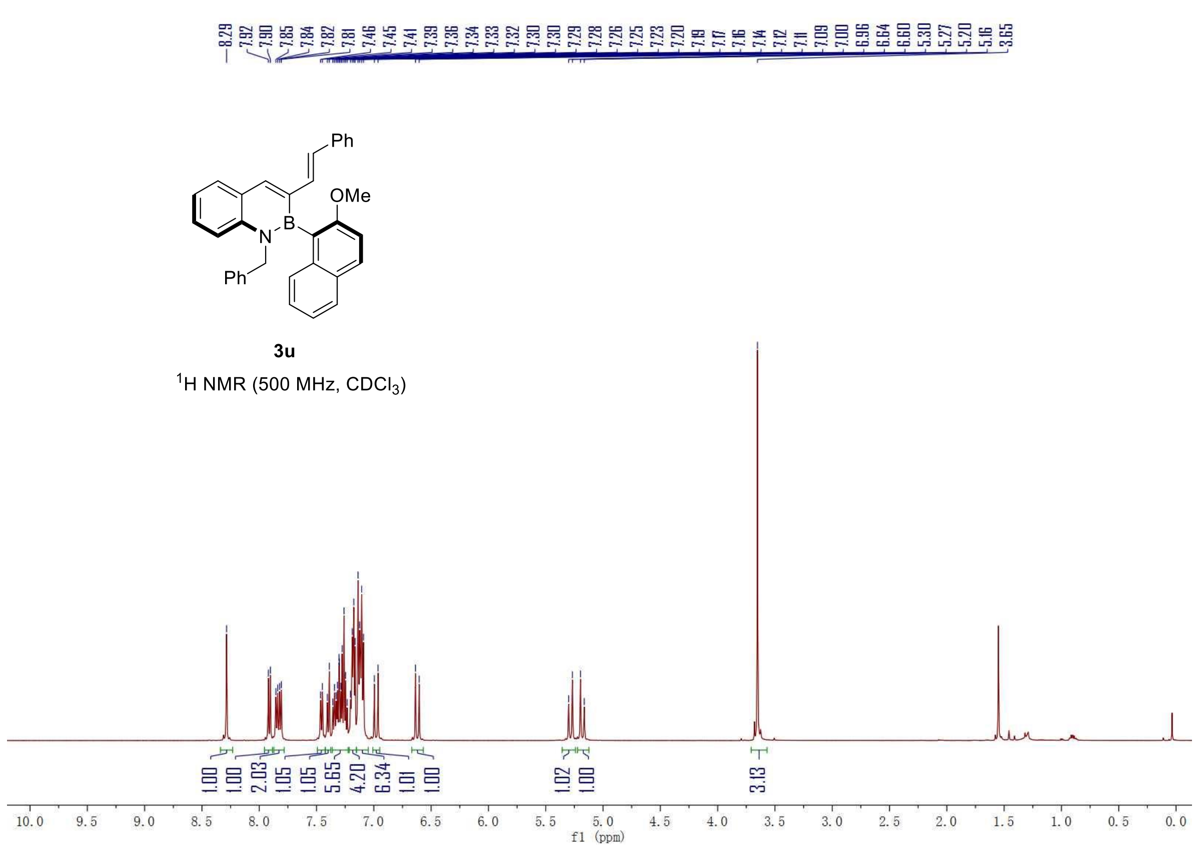

Supplementary Figure 158. ^{1}H NMR spectrum of 3u

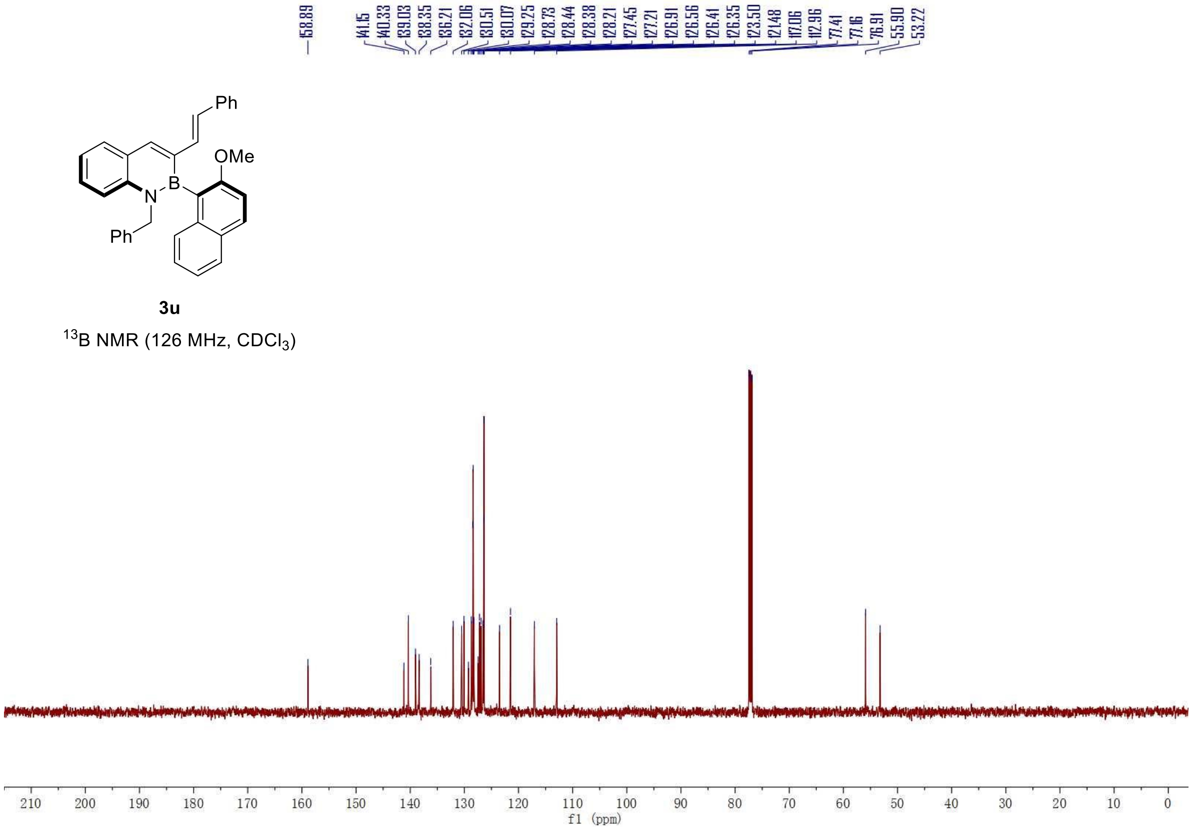

Supplementary Figure 159. ^{13}C NMR spectrum of 3u

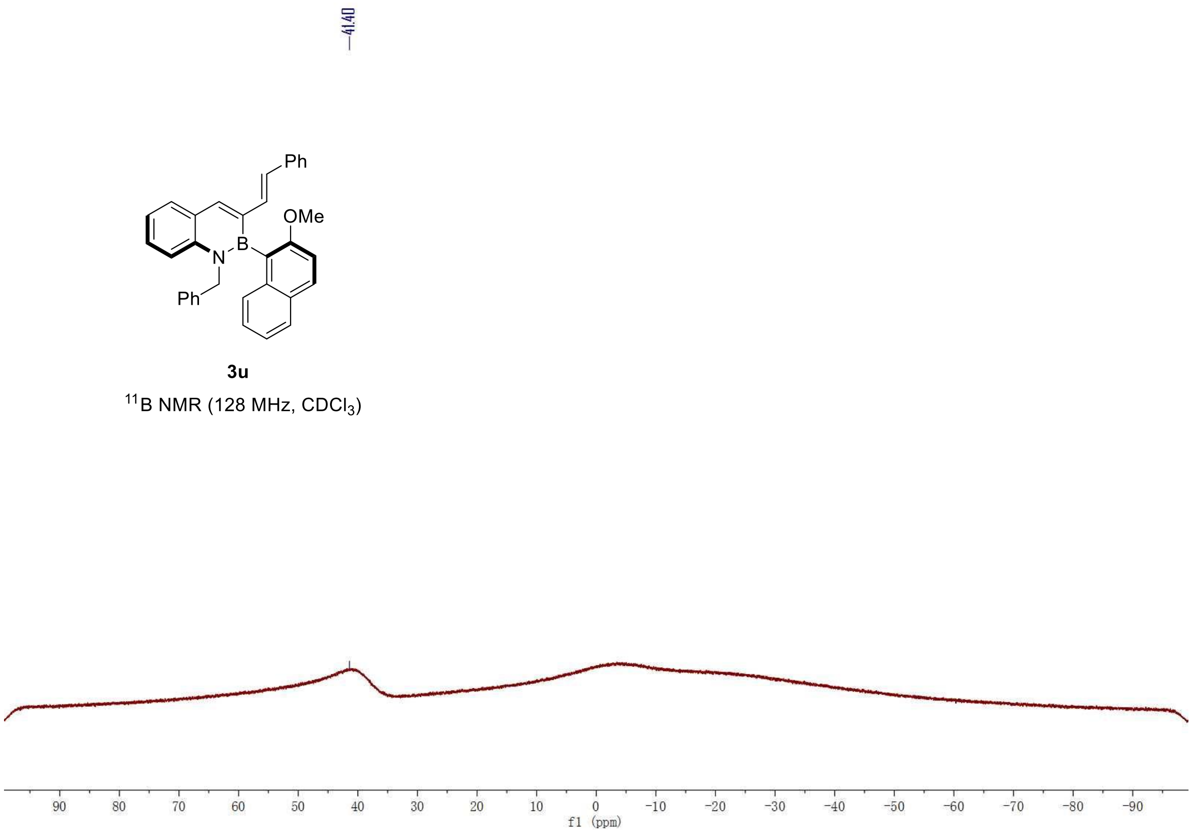

Supplementary Figure 160. ^{11}B NMR spectrum of 3u

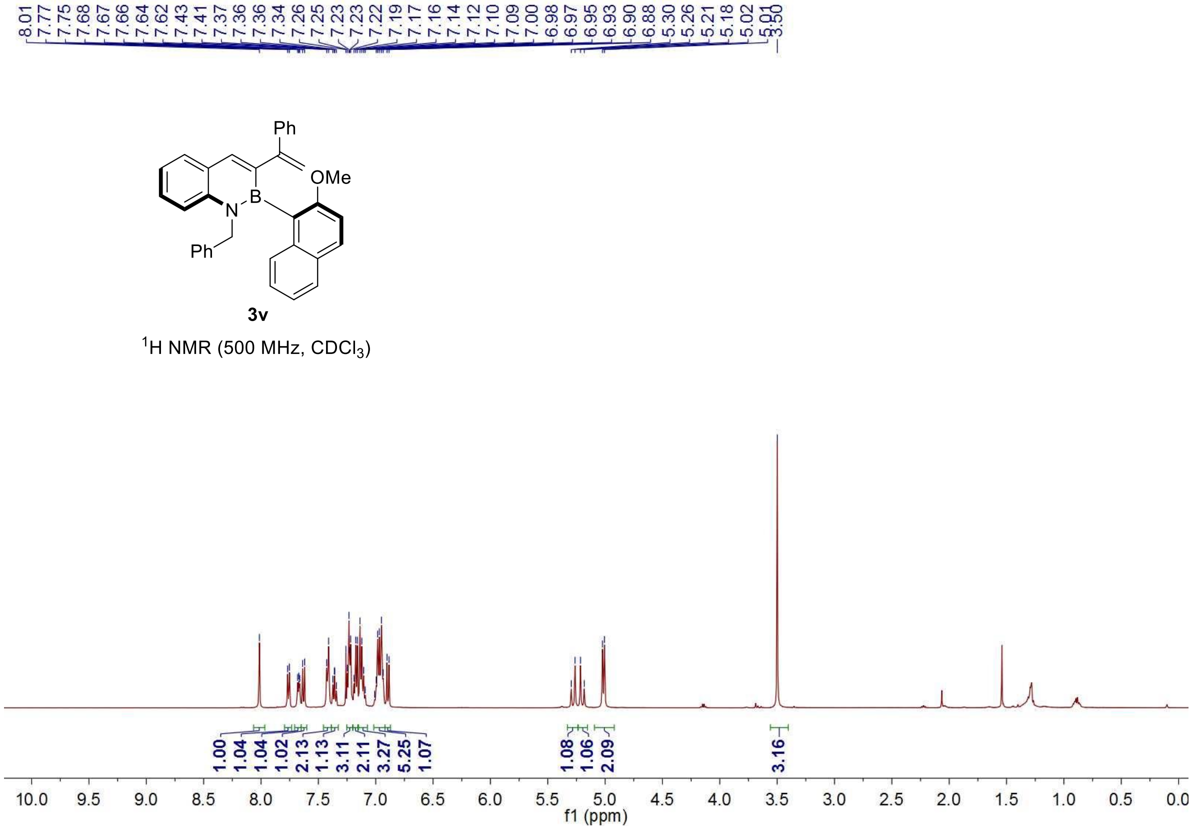

Supplementary Figure 161. ^{1}H NMR spectrum of 3v

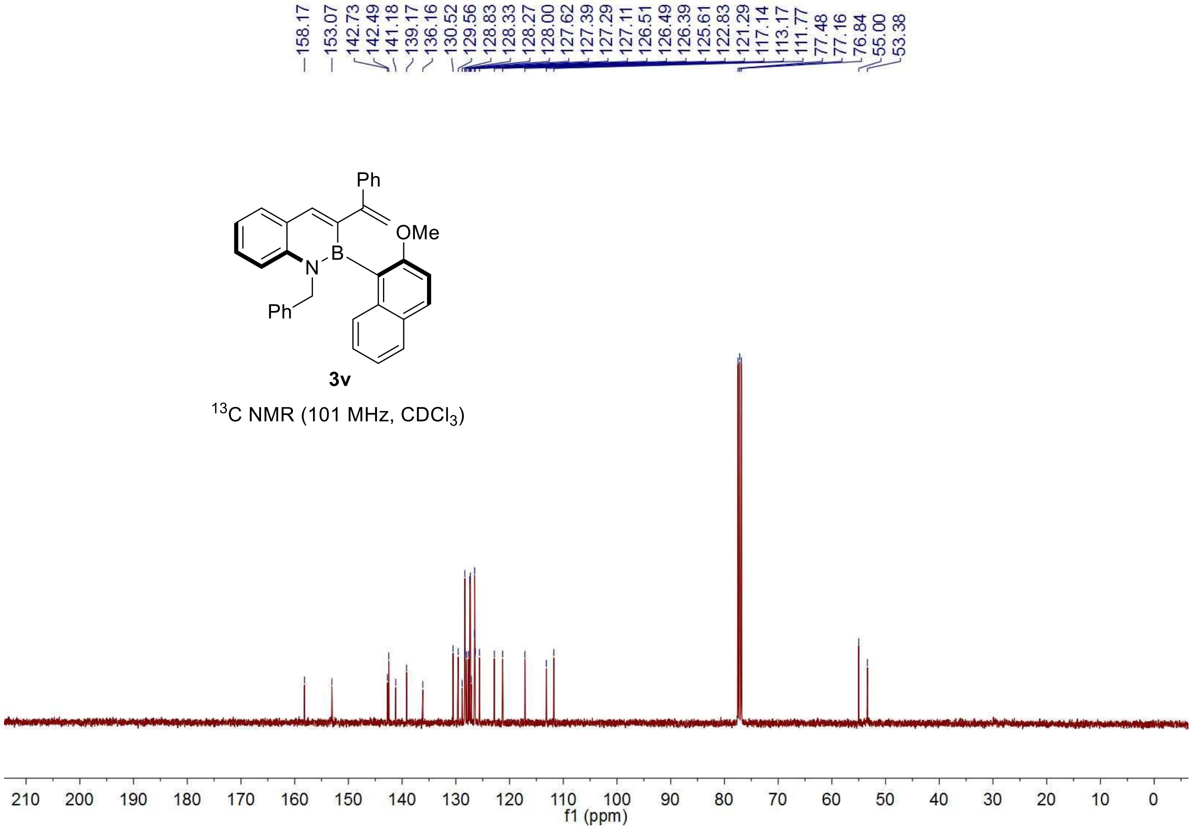

Supplementary Figure 162. ^{13}C NMR spectrum of 3v

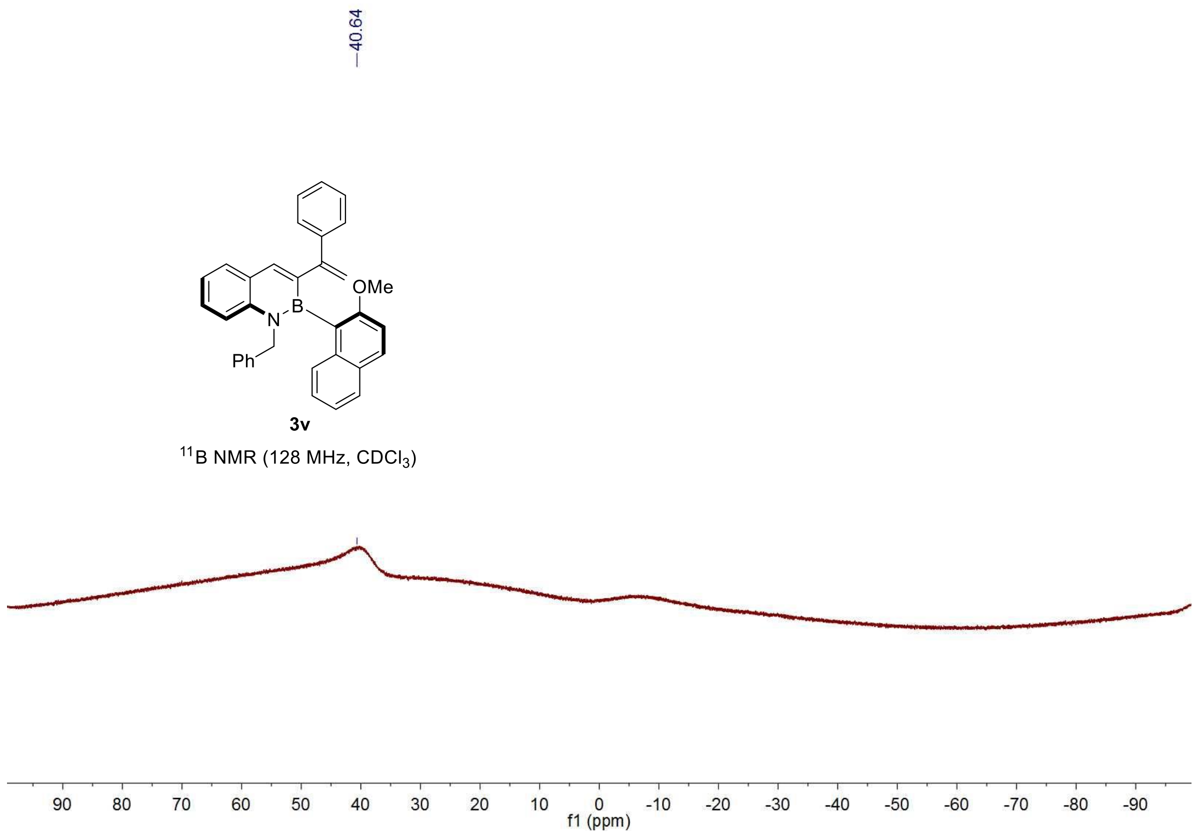

Supplementary Figure 163. ^{11}B NMR spectrum of 3v

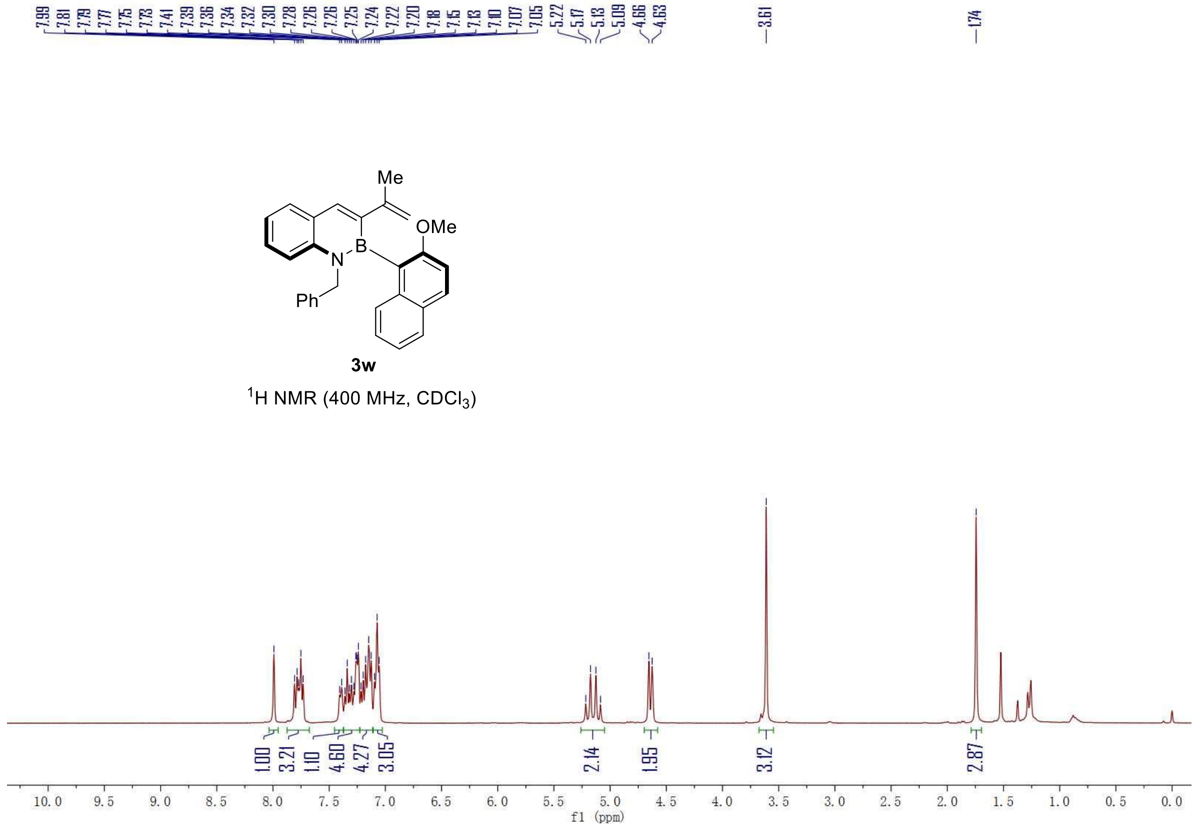

Supplementary Figure 164. ^{1}H NMR spectrum of 3w

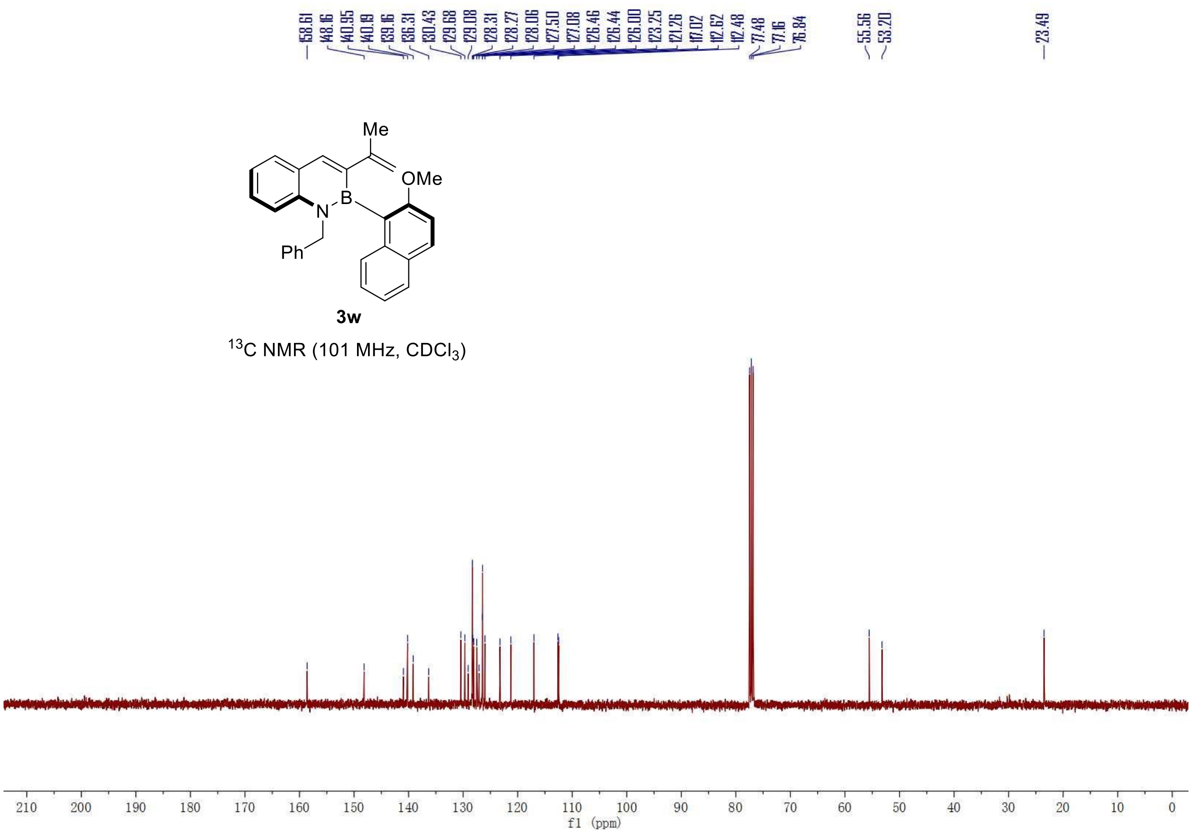

Supplementary Figure 165. ^{13}C NMR spectrum of 3w

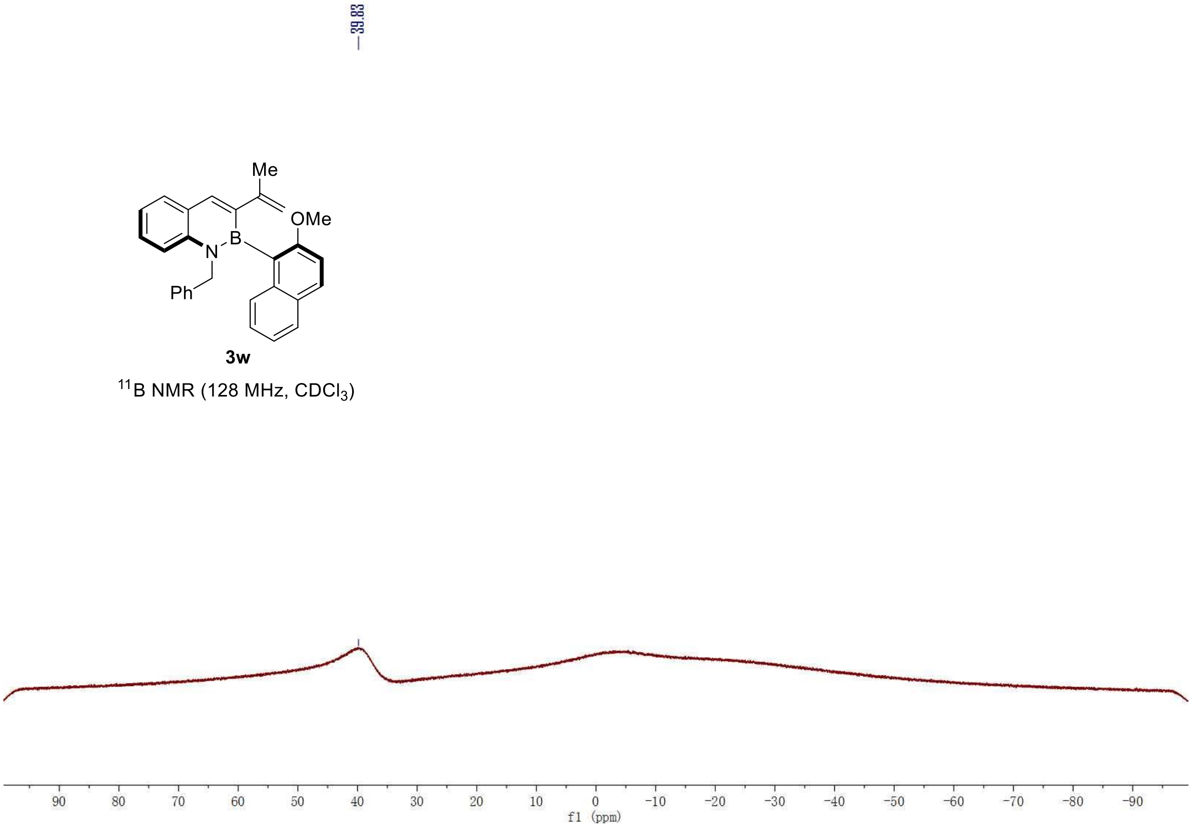

Supplementary Figure 166. ^{11}B NMR spectrum of 3w

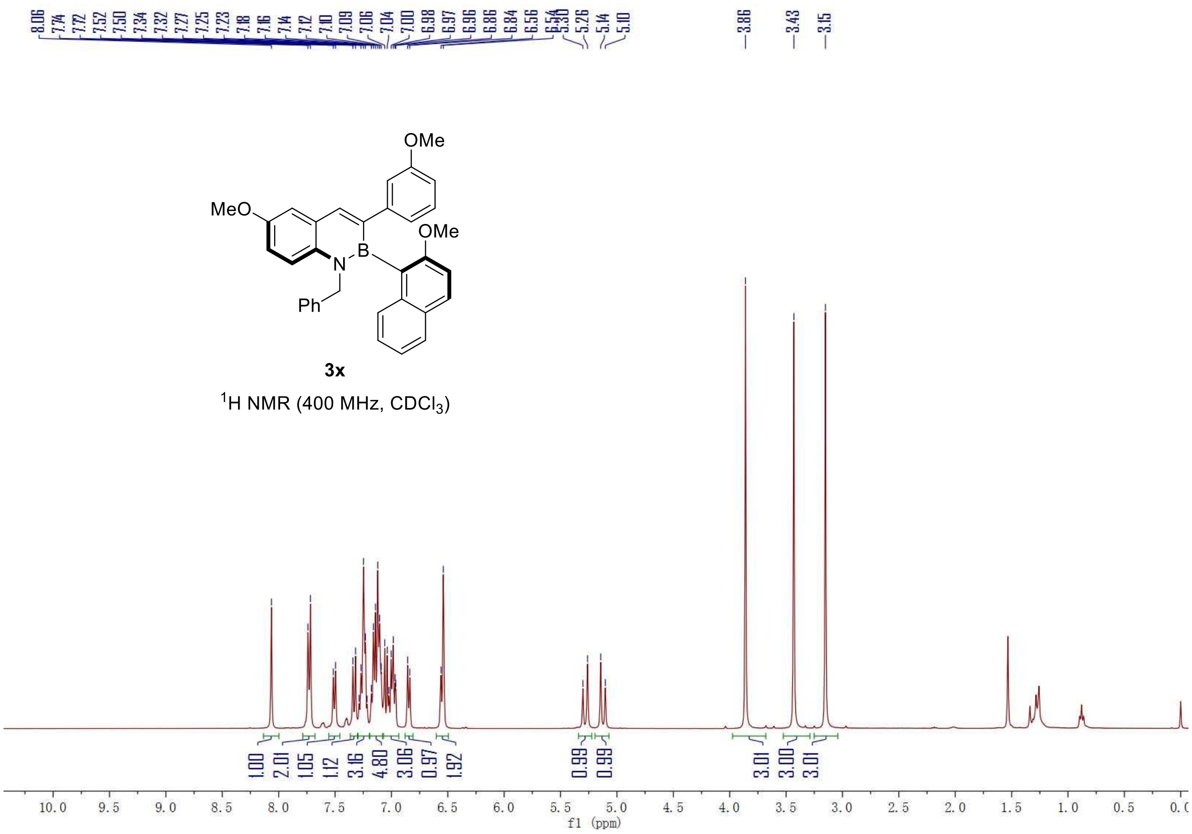

Supplementary Figure 167. ^{1}H NMR spectrum of 3x

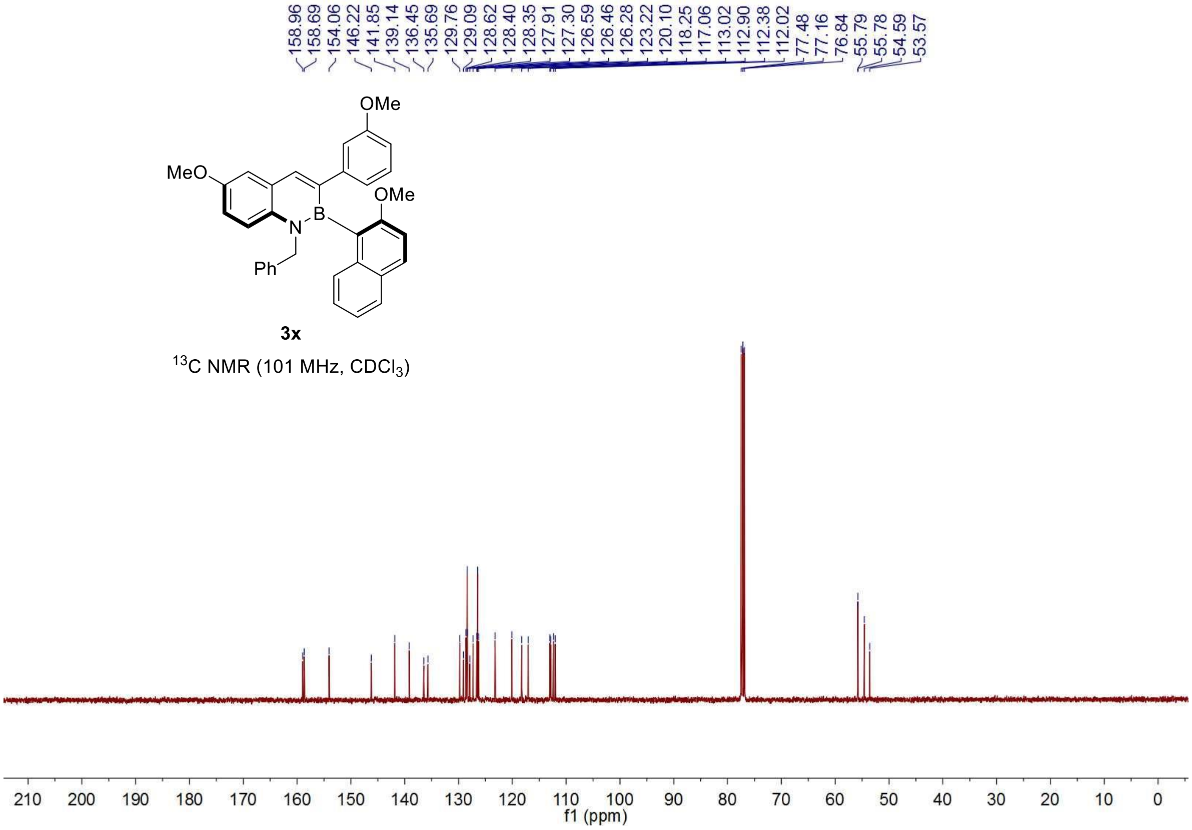

Supplementary Figure 168. ^{13}C NMR spectrum of 3x

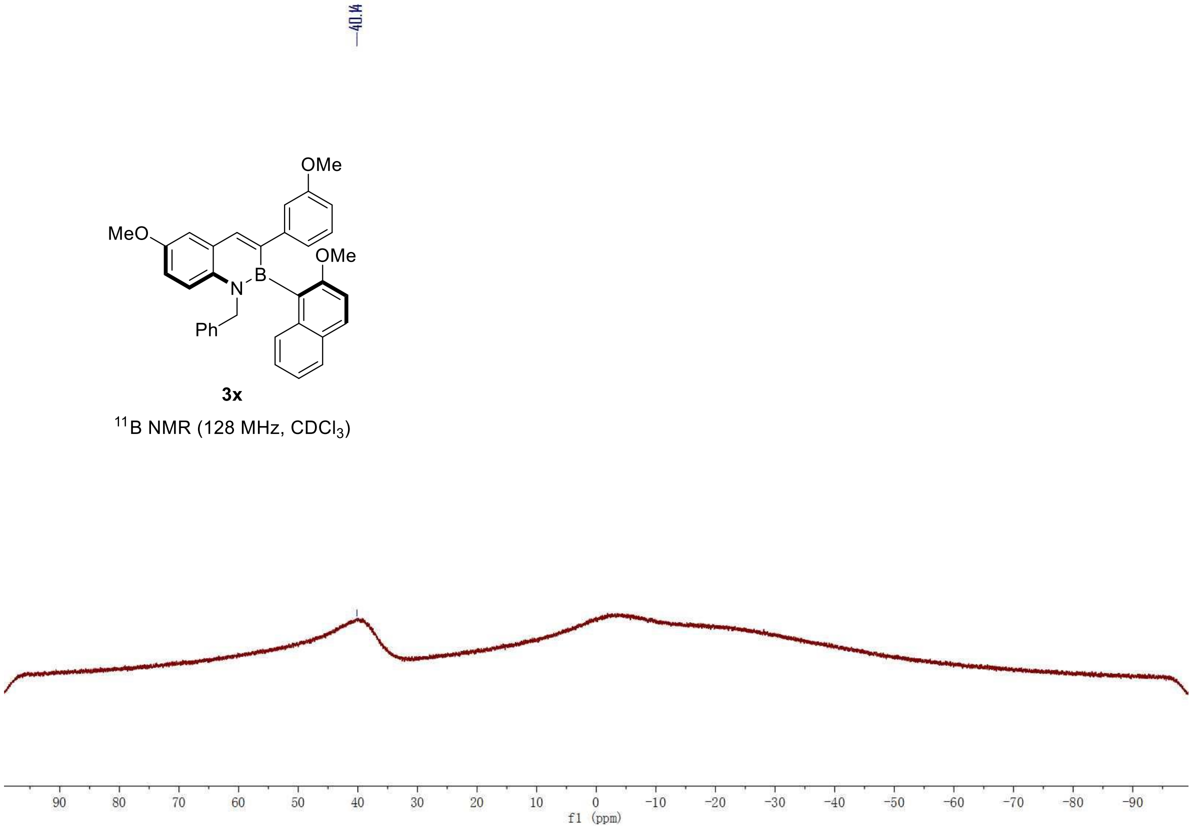

Supplementary Figure 169. ^{11}B NMR spectrum of 3x

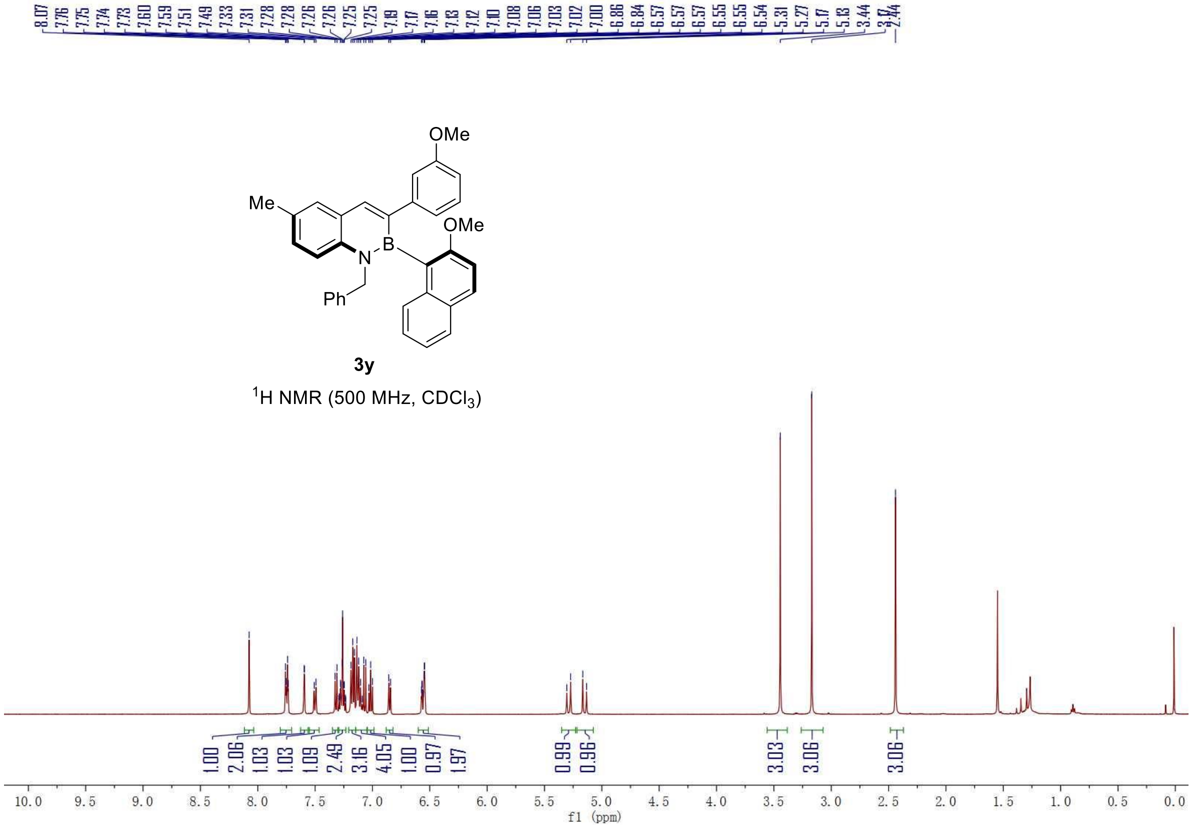

Supplementary Figure 170. ^{1}H NMR spectrum of 3y

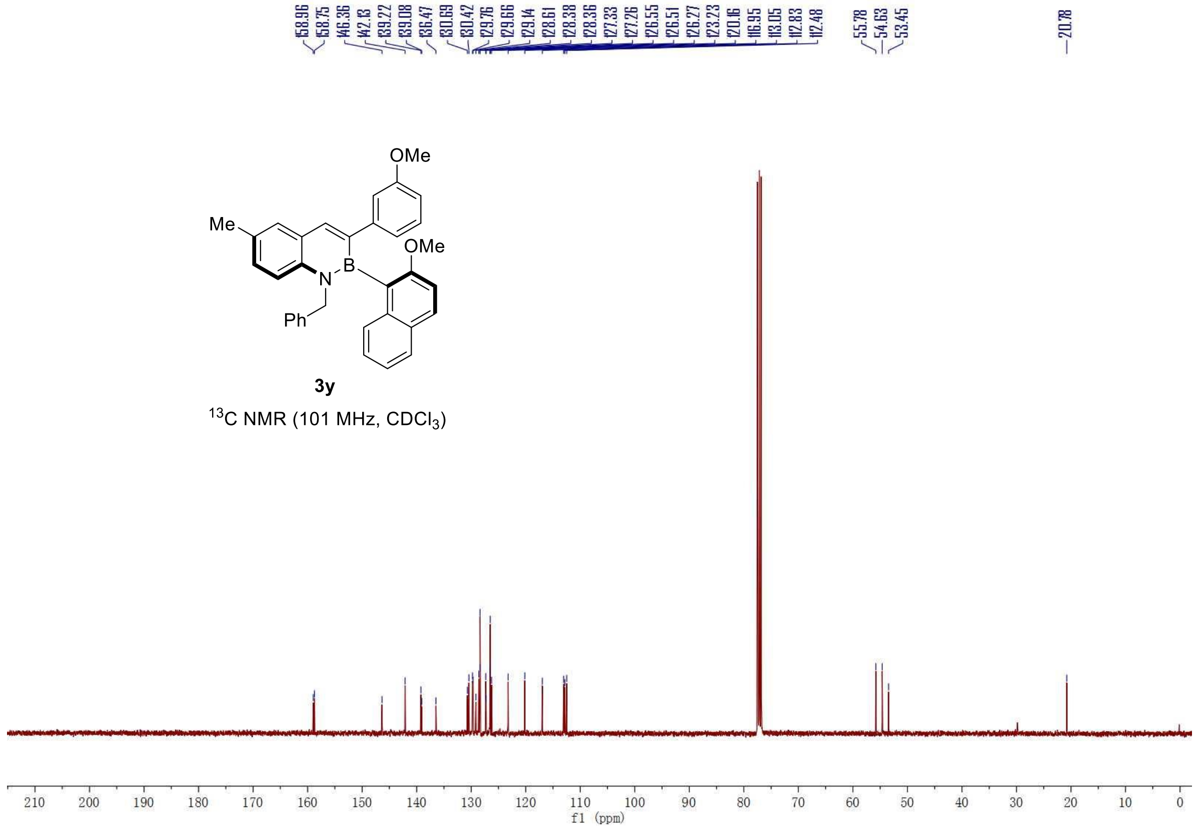

Supplementary Figure 171. ^{13}C NMR spectrum of 3y

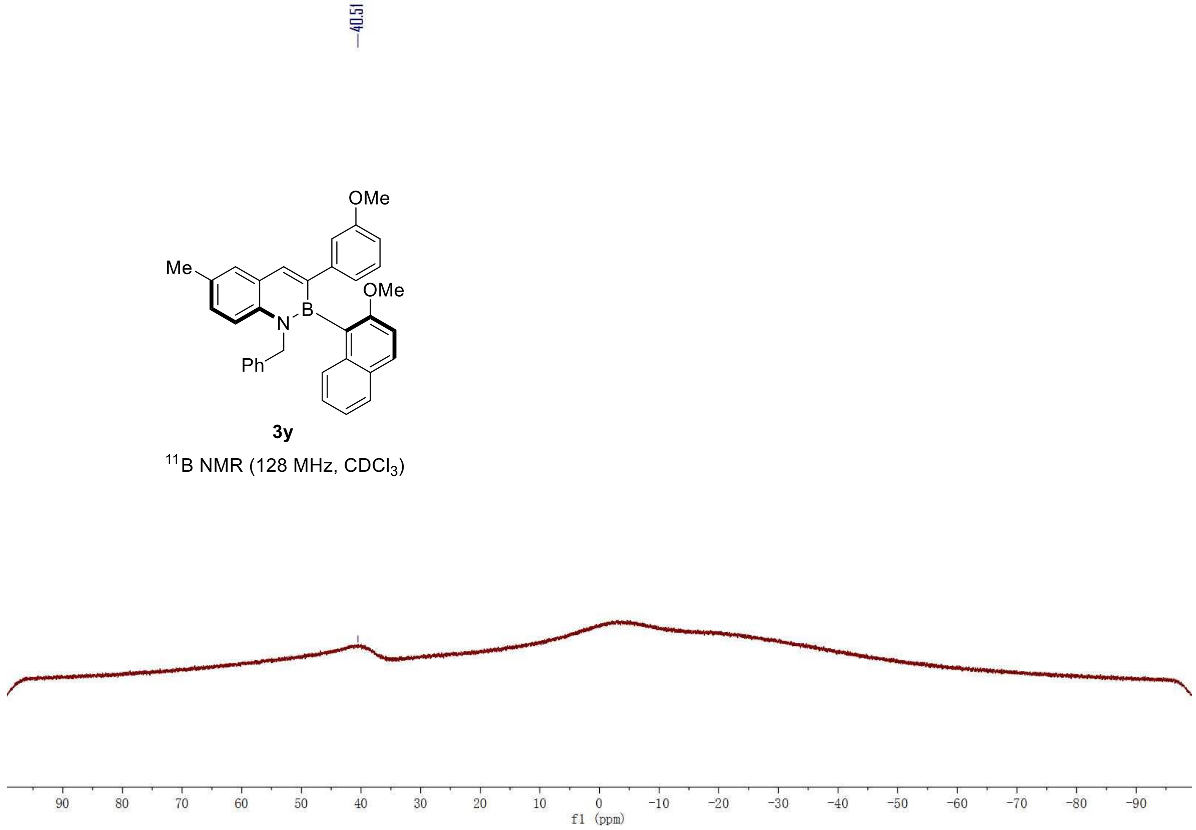

Supplementary Figure 172. ^{11}B NMR spectrum of 3y

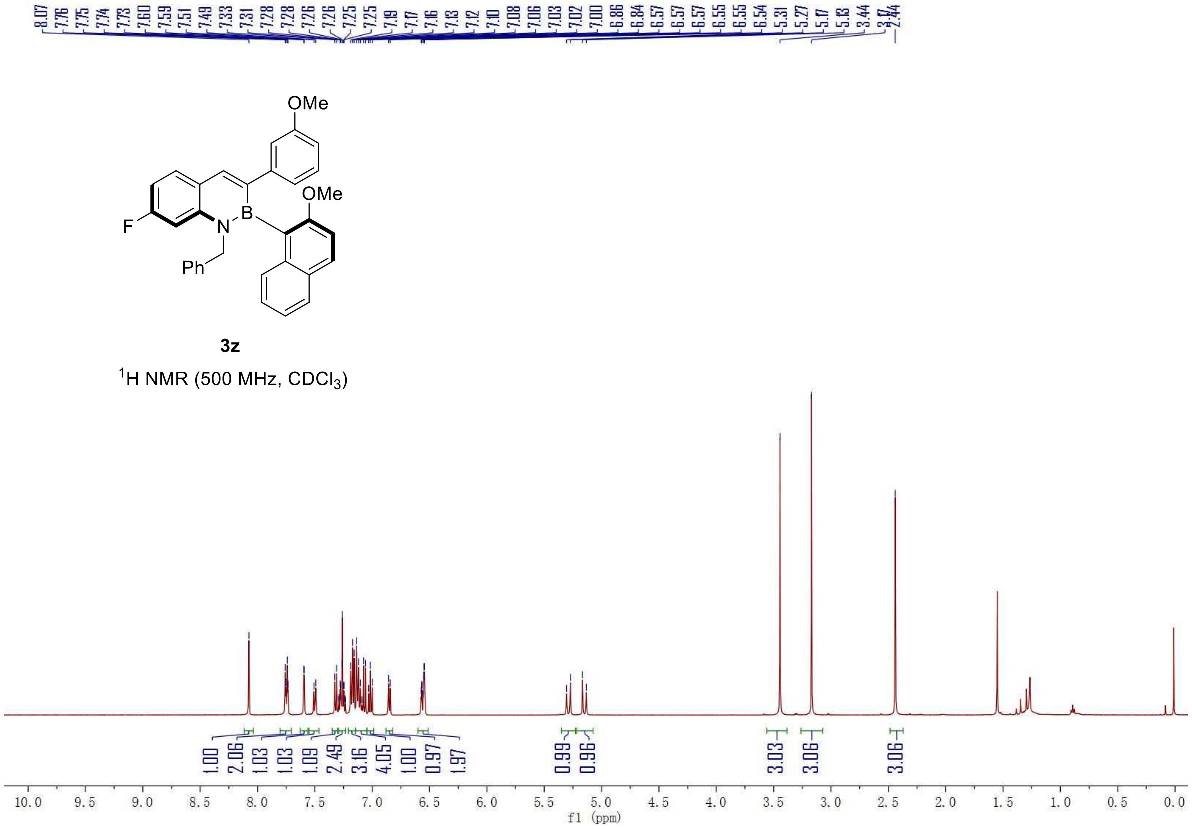

Supplementary Figure 173. ^{1}H NMR spectrum of 3z

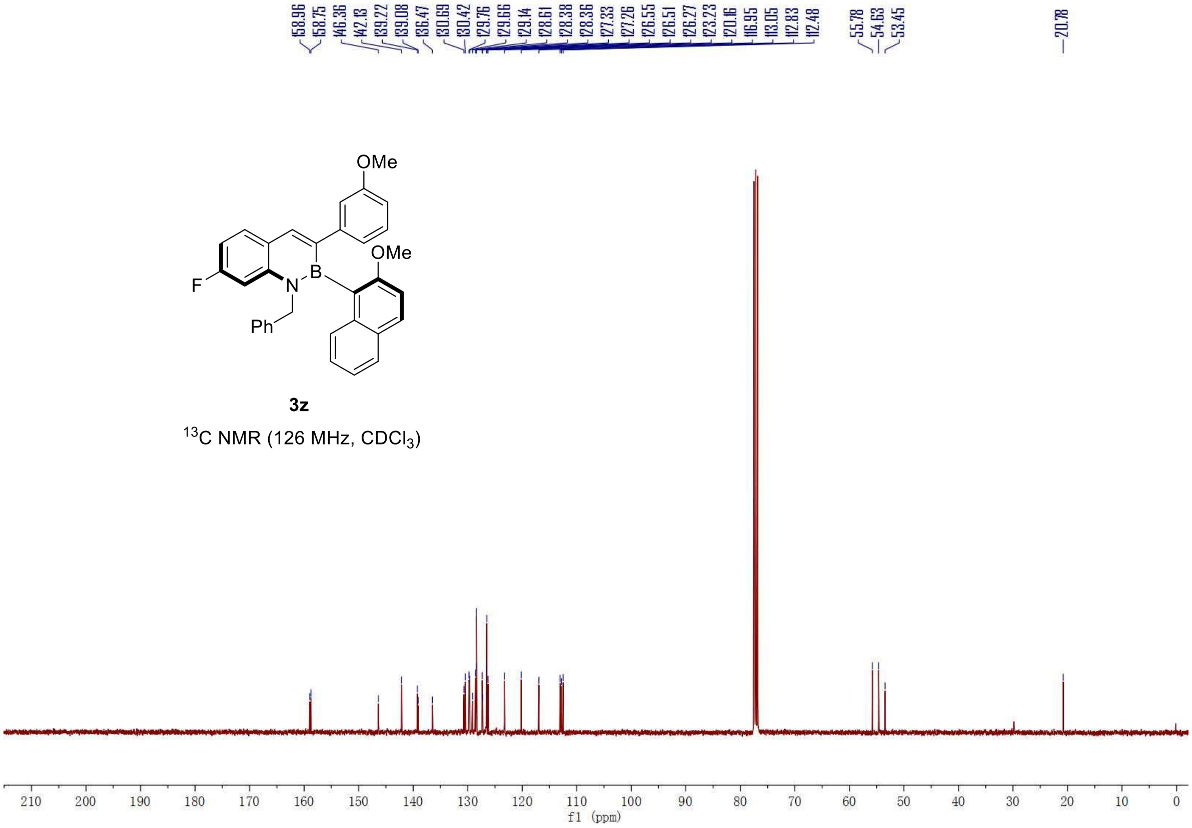

Supplementary Figure 174. ^{13}C NMR spectrum of 3z

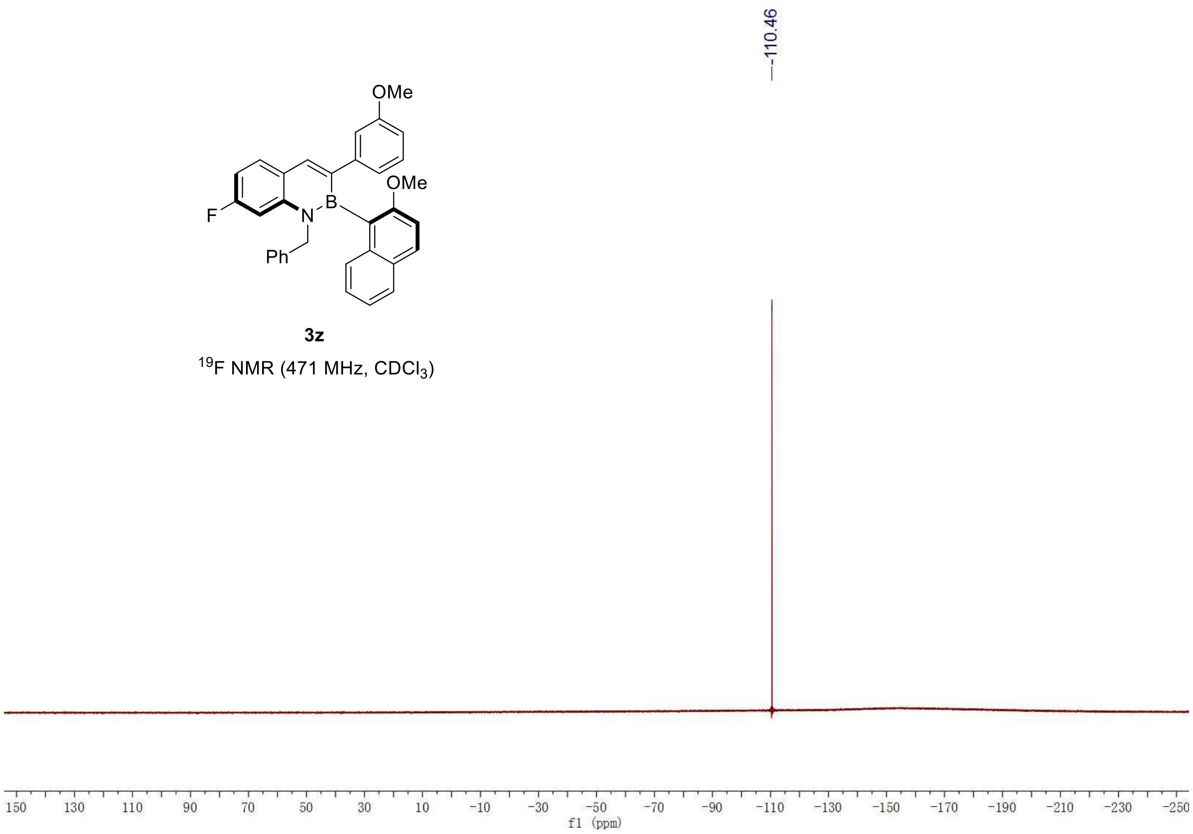

Supplementary Figure 175. ^{19}F NMR spectrum of 3z

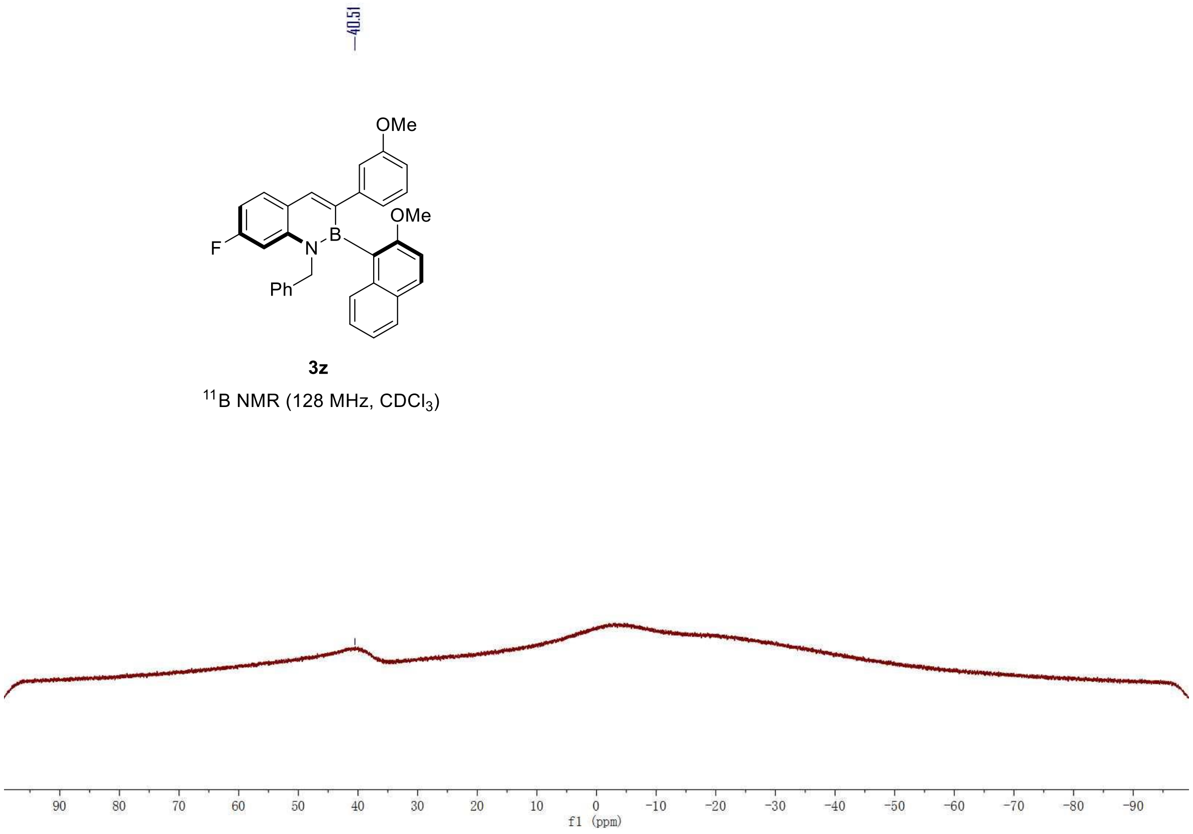

Supplementary Figure 176. ^{11}B NMR spectrum of 3z

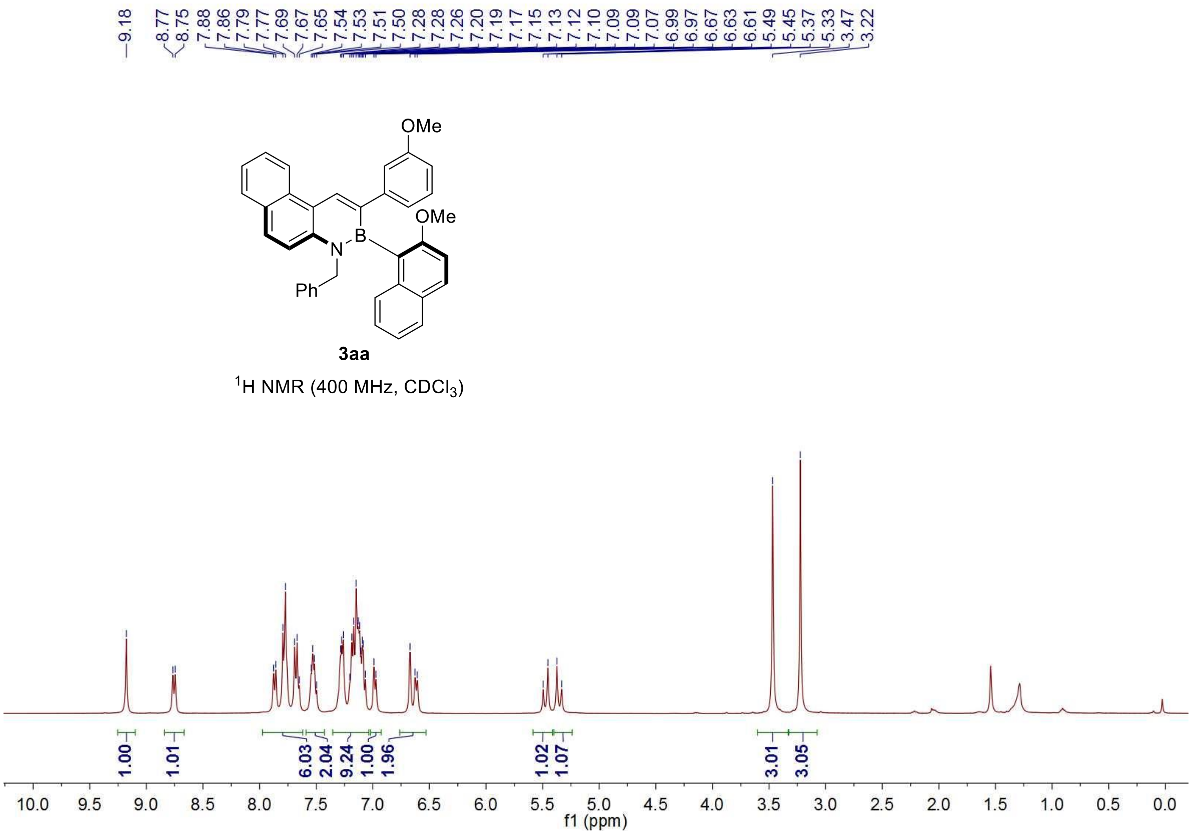

Supplementary Figure 177. ^{1}H NMR spectrum of 3aa

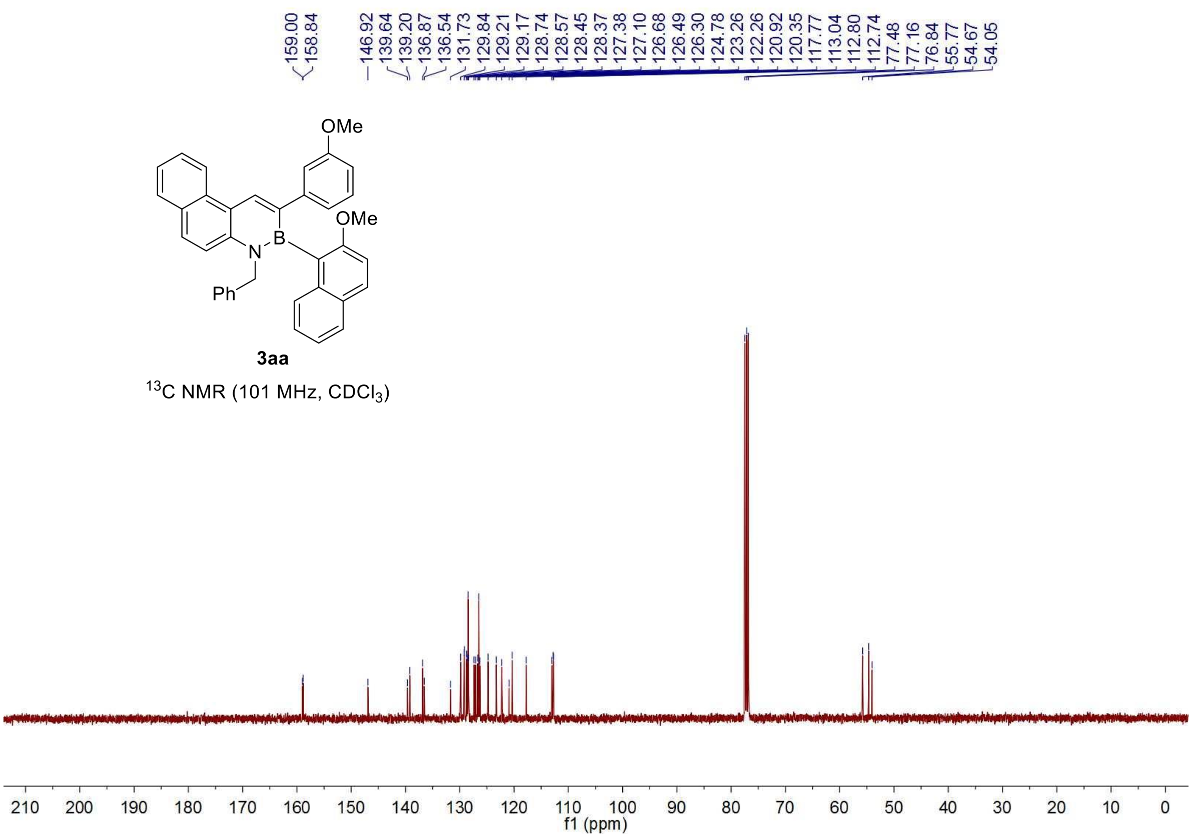

Supplementary Figure 178. ^{13}C NMR spectrum of 3aa

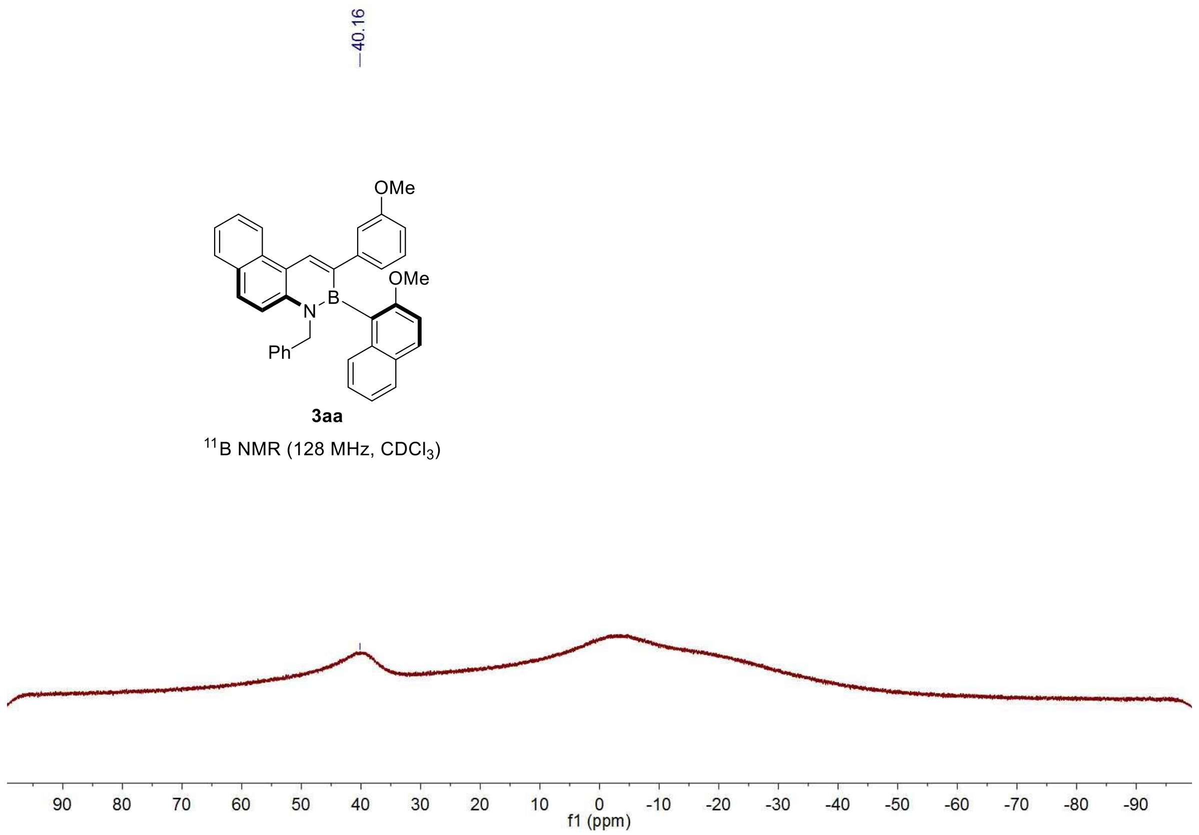

Supplementary Figure 179. ^{11}B NMR spectrum of 3aa

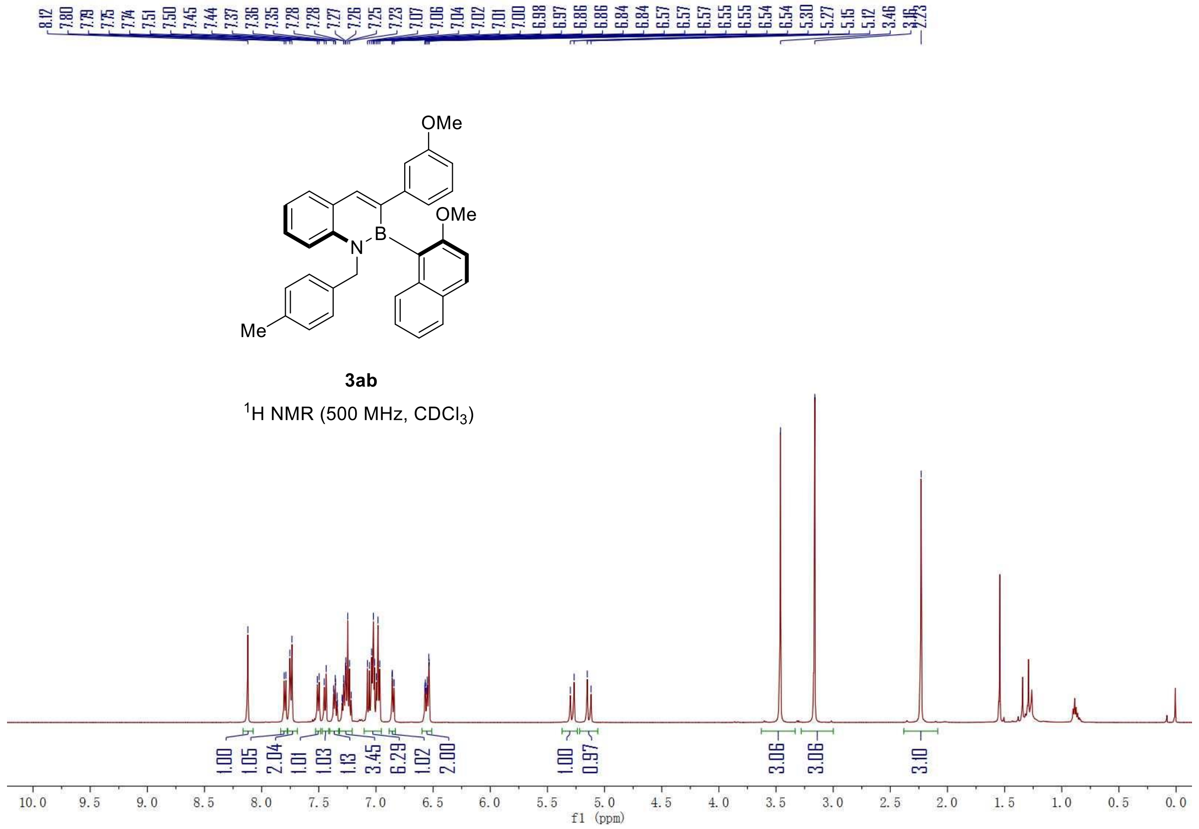

Supplementary Figure 180. ^{1}H NMR spectrum of 3ab

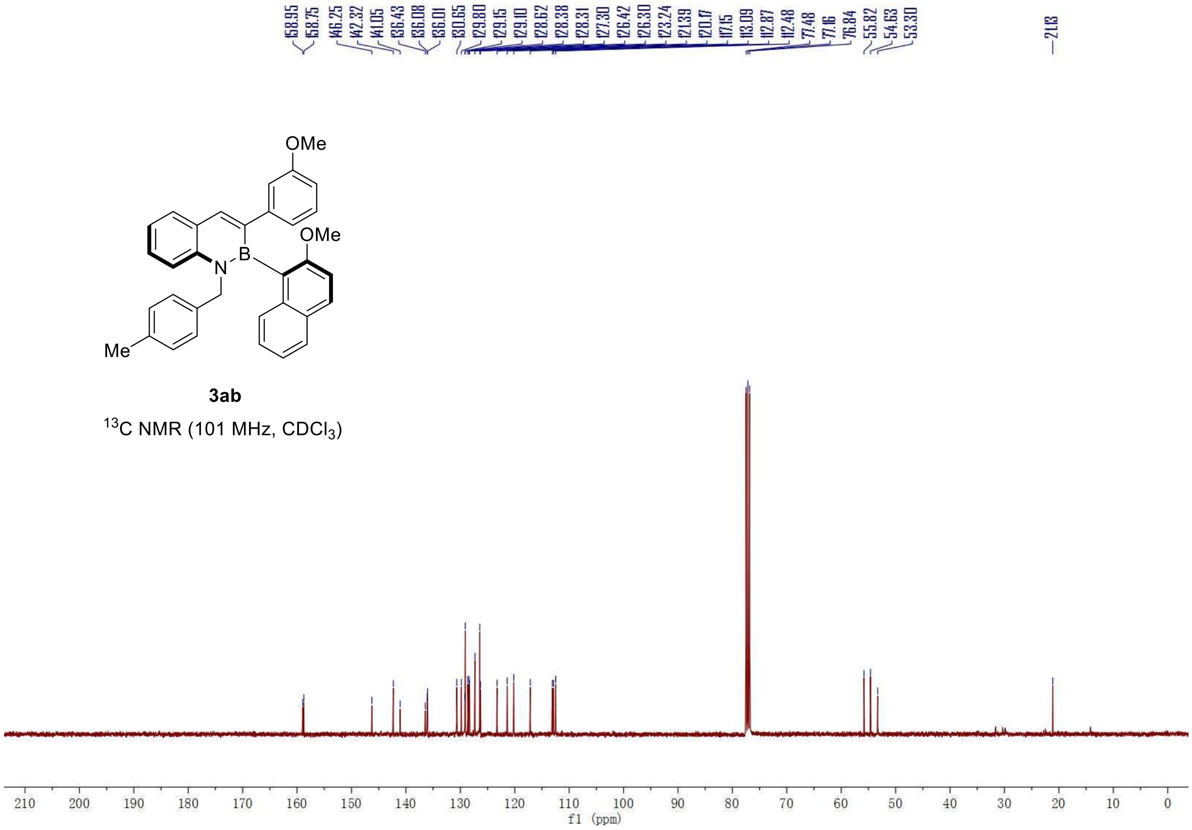

Supplementary Figure 181. ^{13}C NMR spectrum of 3ab

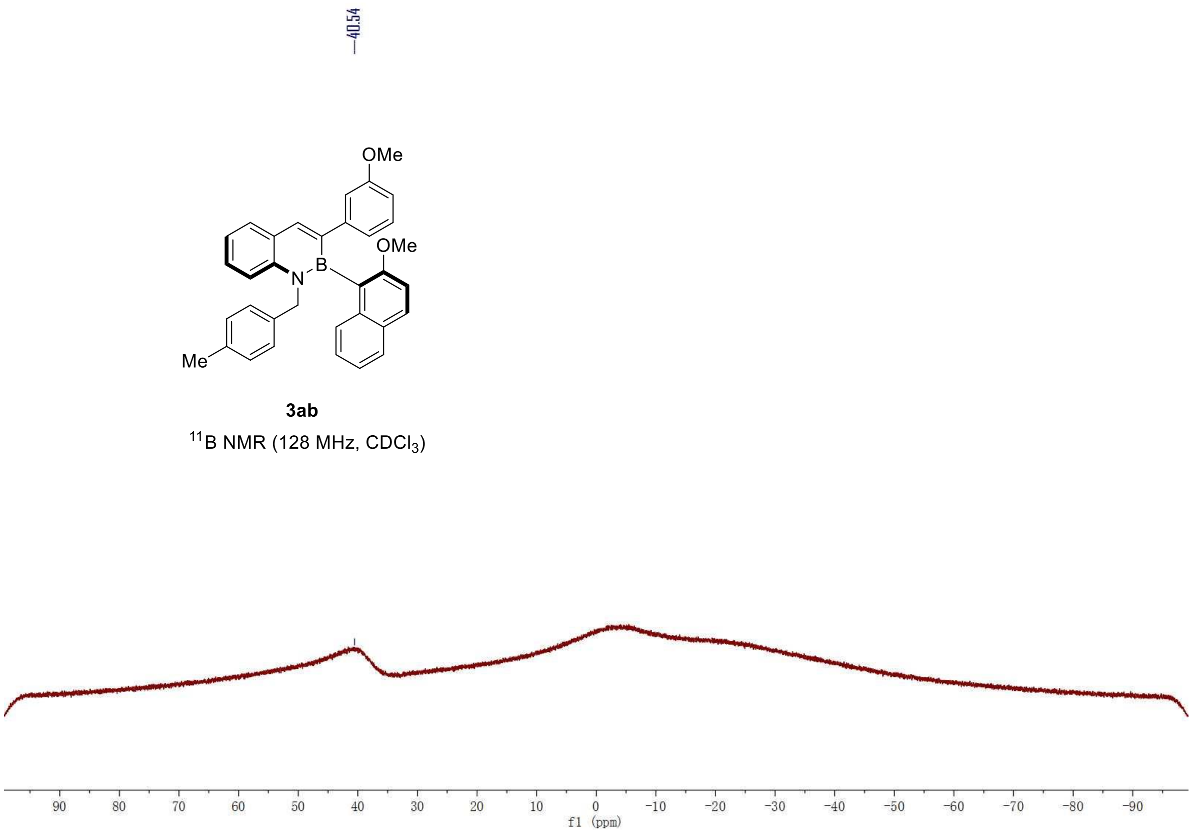

Supplementary Figure 182. ^{11}B NMR spectrum of 3ab

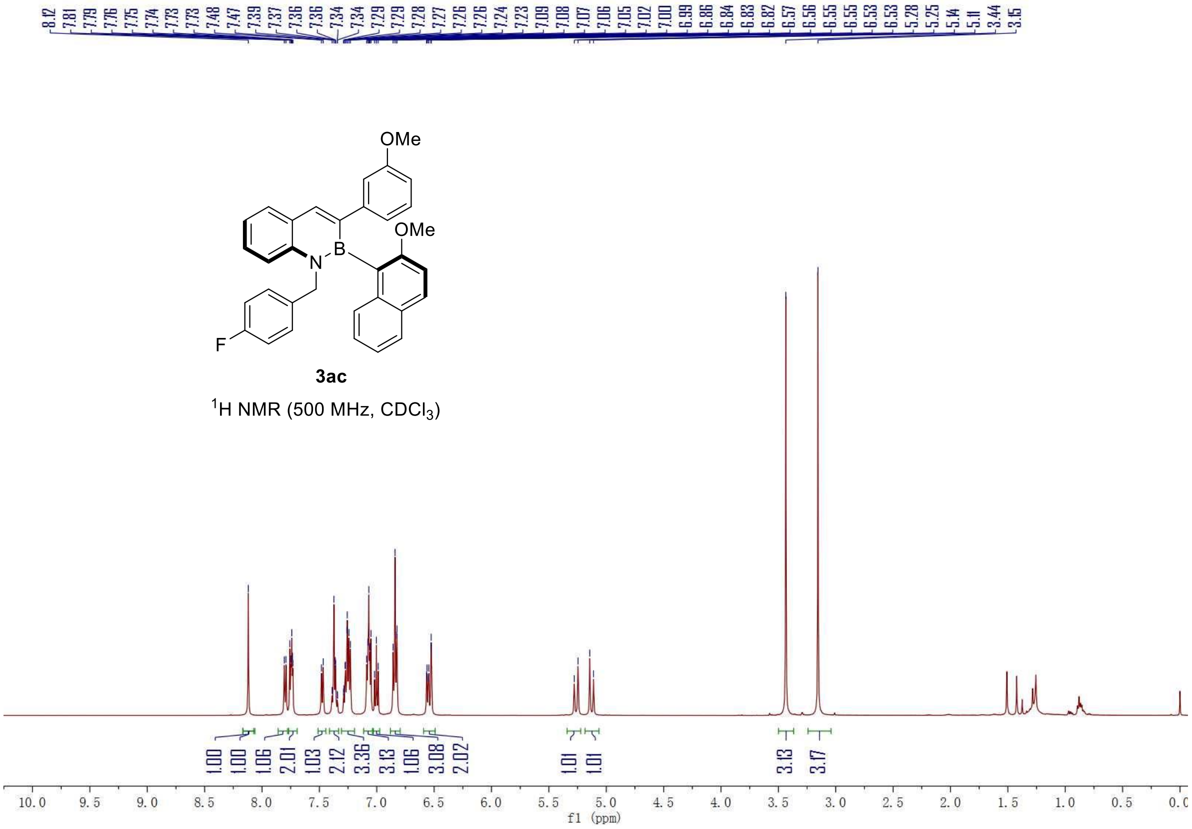

Supplementary Figure 183. ^{1}H NMR spectrum of 3ac

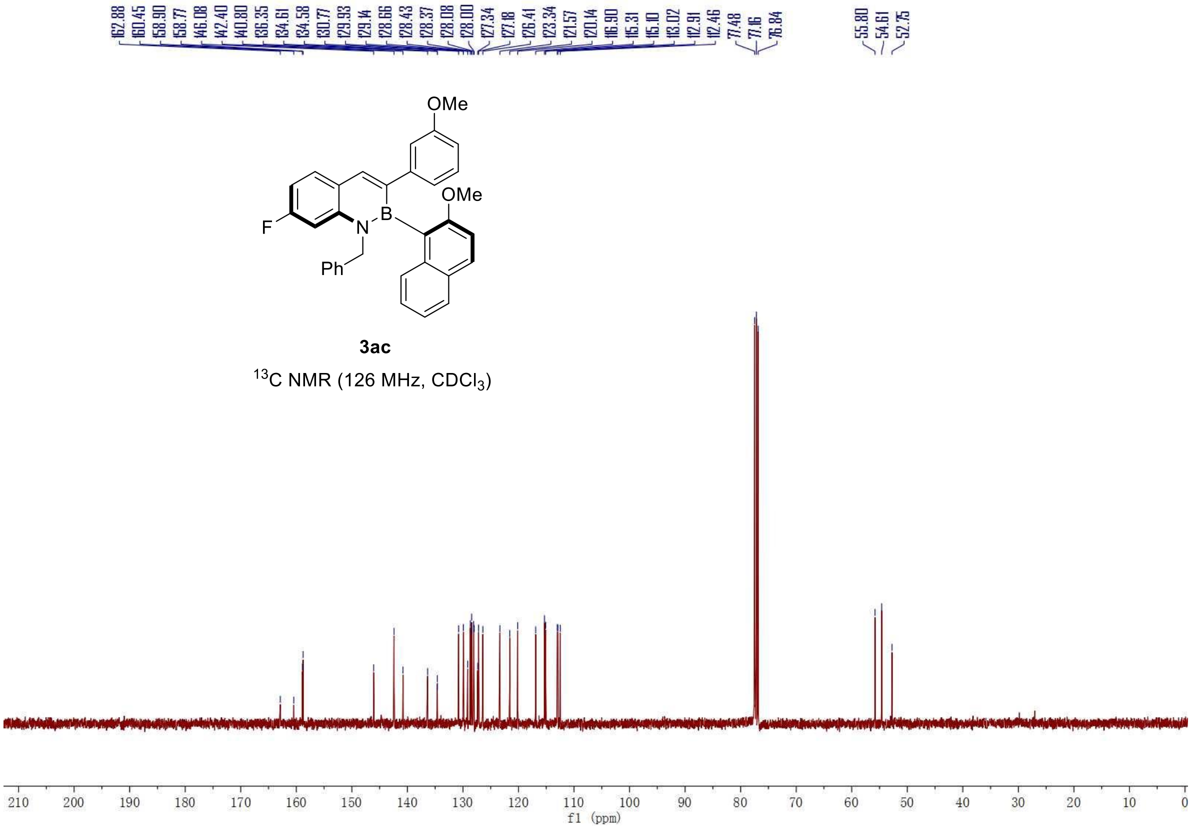

Supplementary Figure 184. ^{13}C NMR spectrum of 3ac

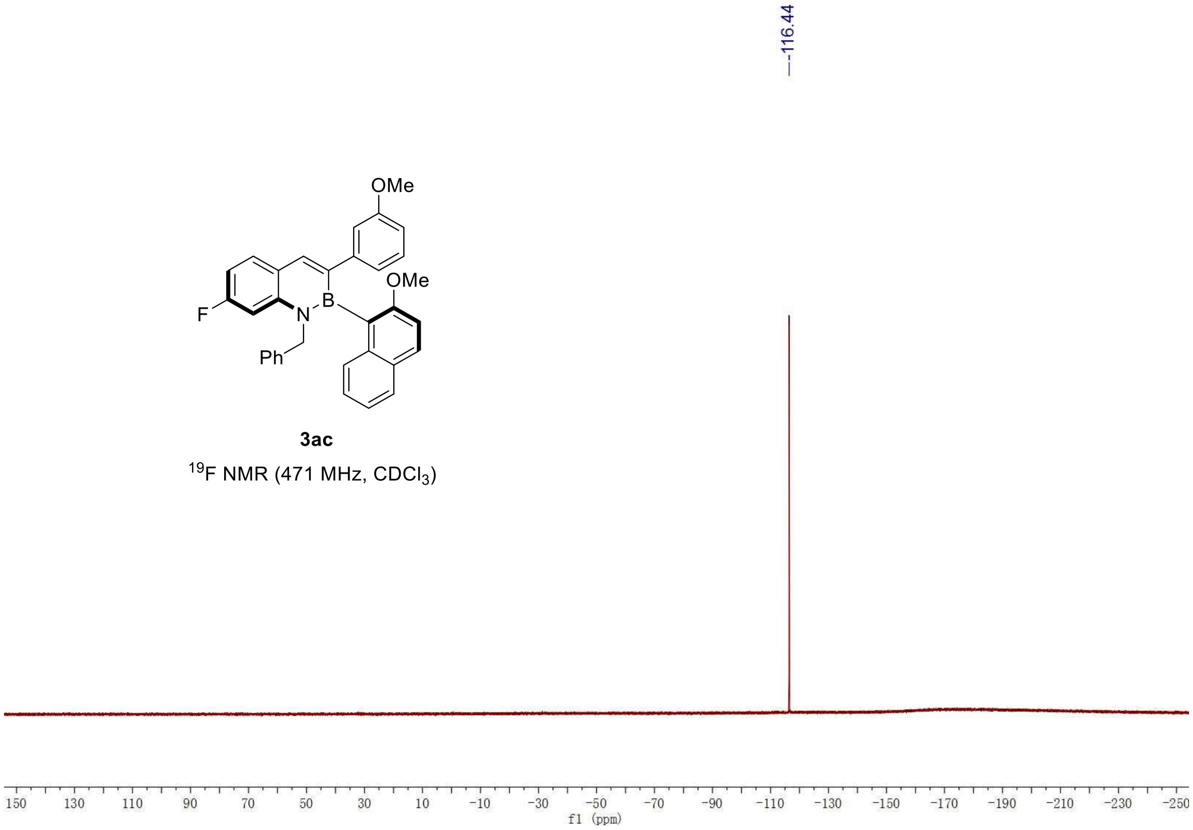

Supplementary Figure 185. ^{19}F NMR spectrum of 3ac

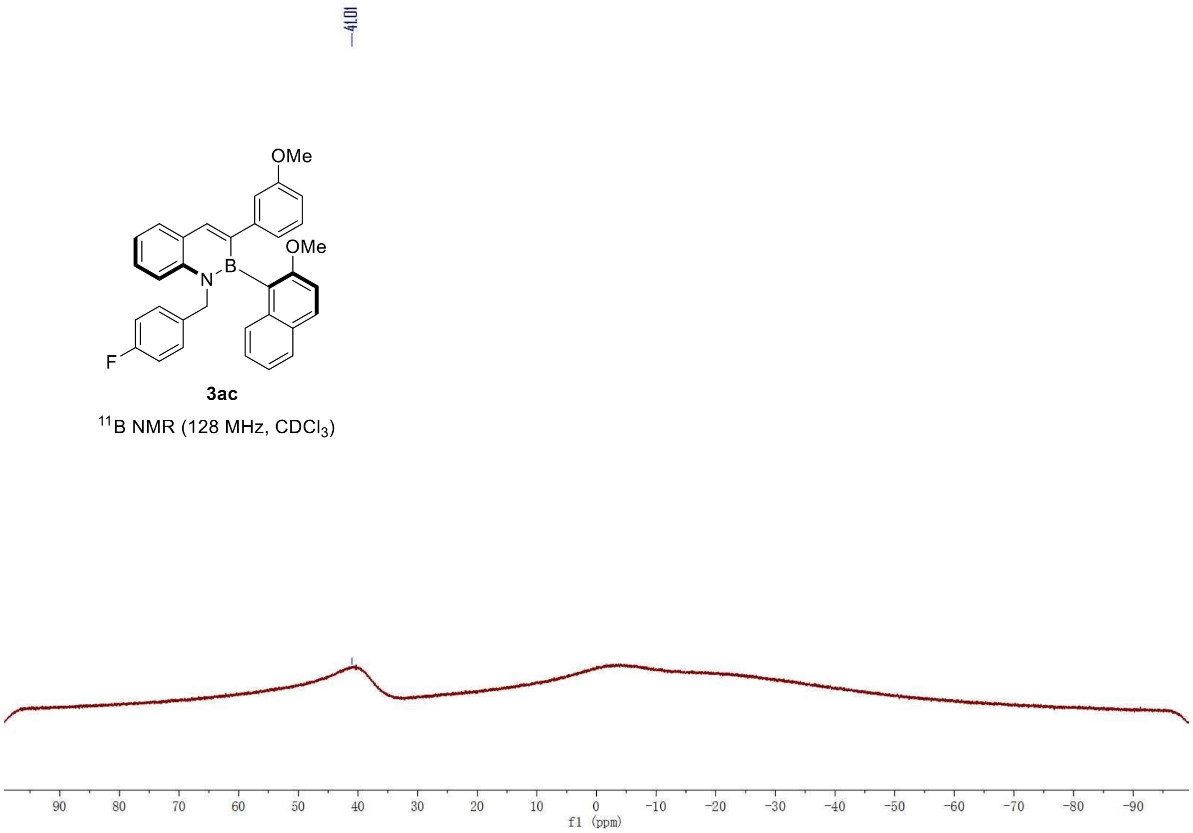

Supplementary Figure 186. ^{11}B NMR spectrum of 3ac

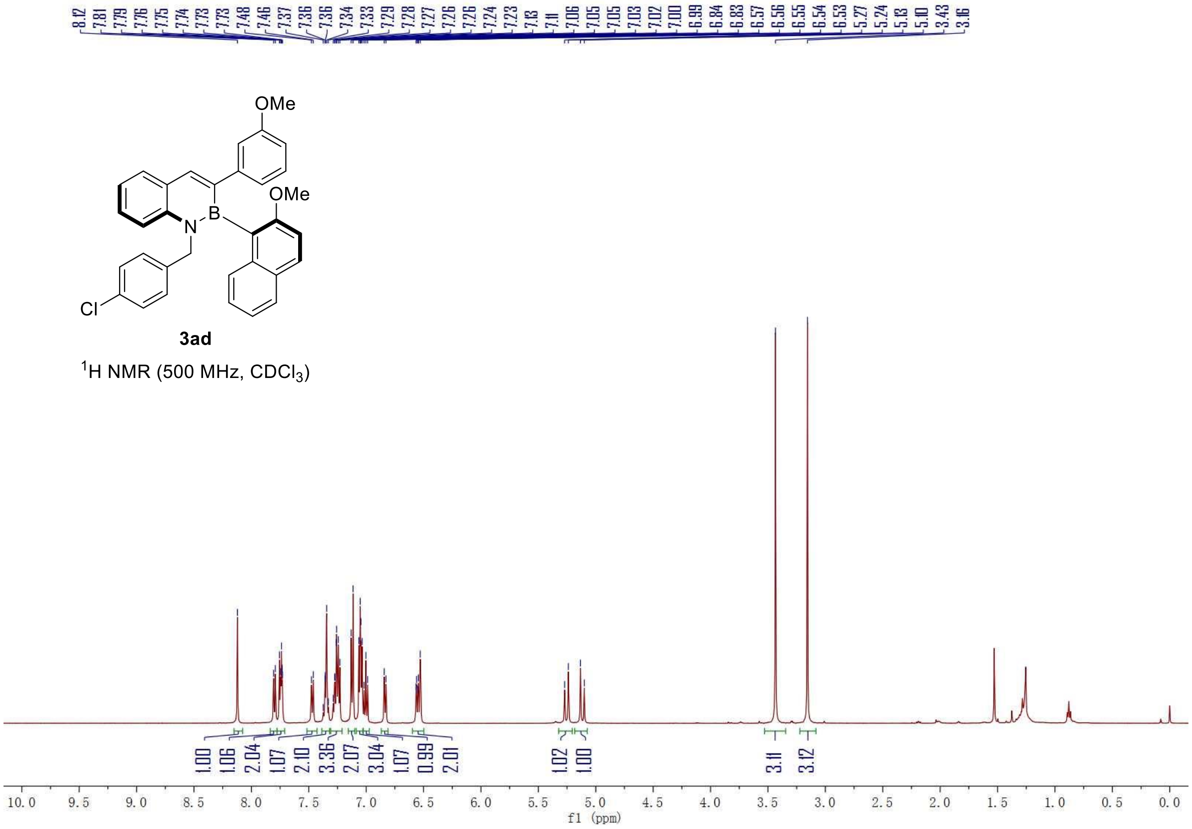

Supplementary Figure 187. ^{1}H NMR spectrum of 3ad

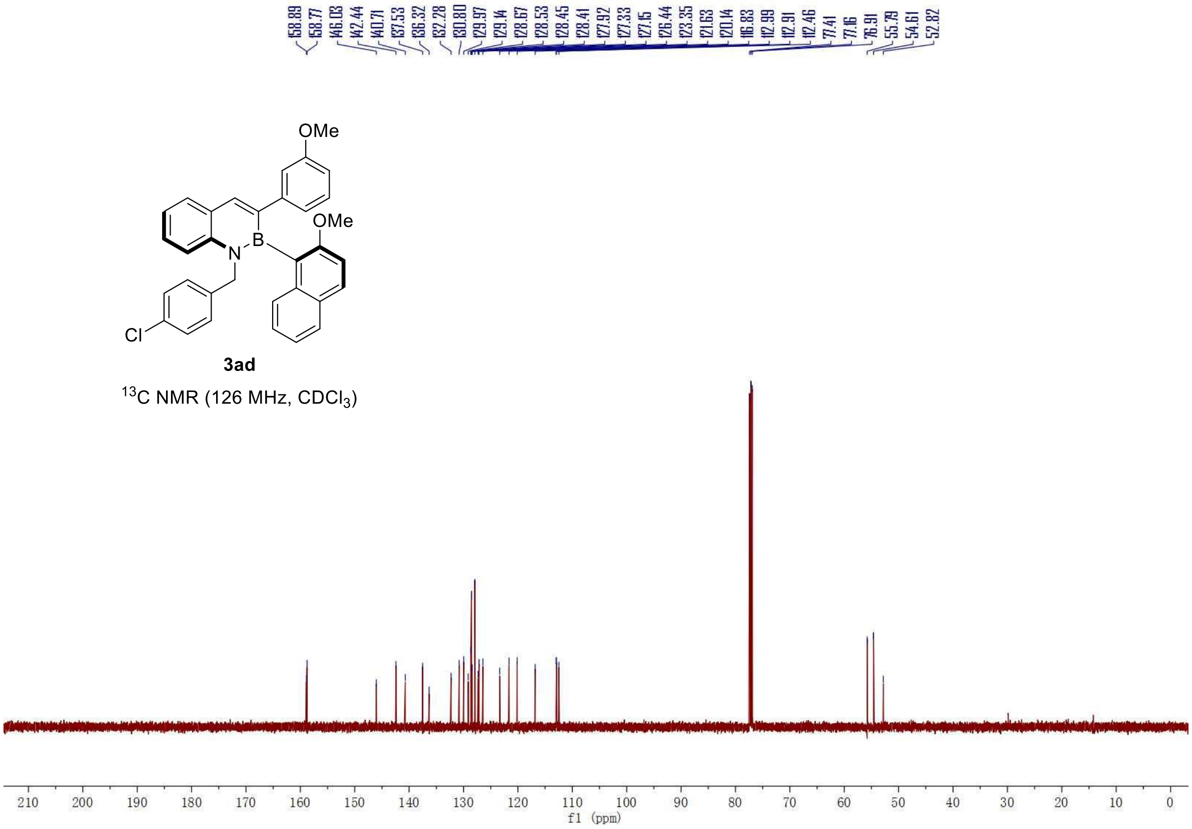

Supplementary Figure 188. ^{13}C NMR spectrum of 3ad

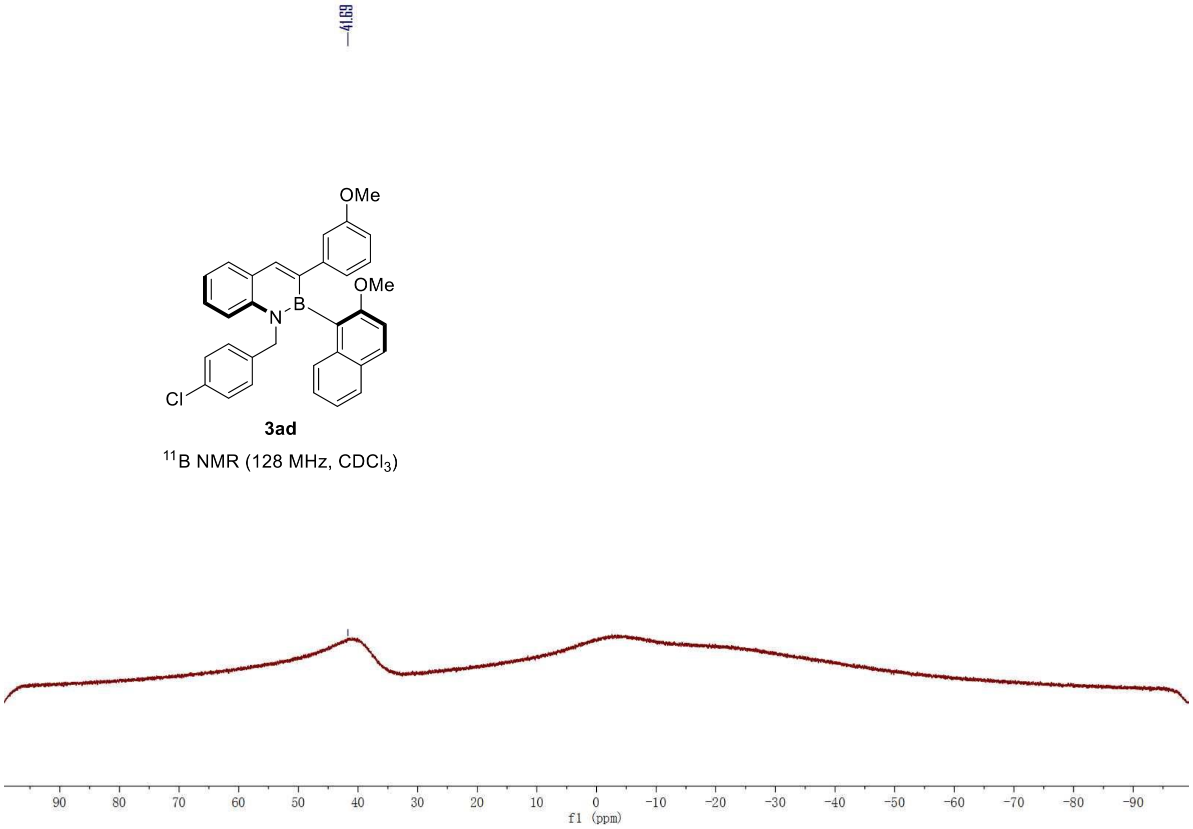

Supplementary Figure 189. ^{11}B NMR spectrum of 3ad

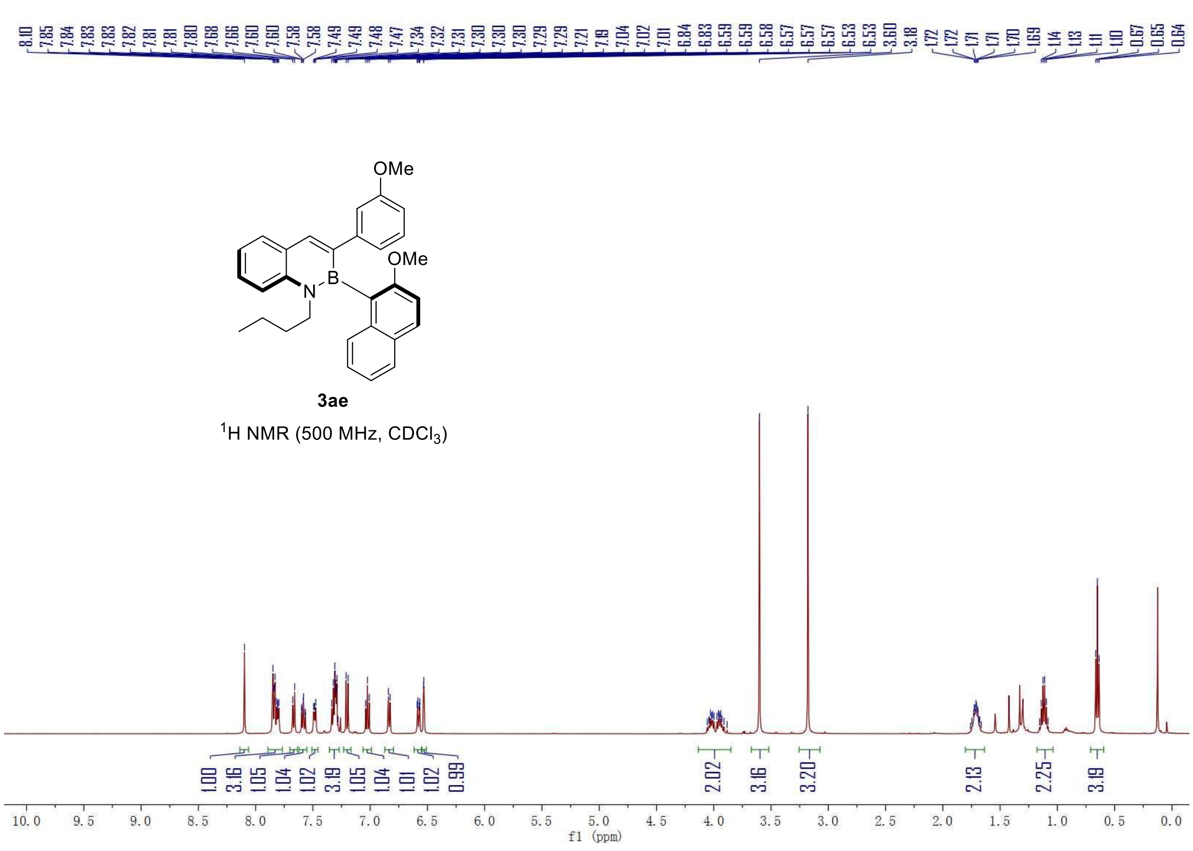

Supplementary Figure 190. ^{1}H NMR spectrum of 3ae

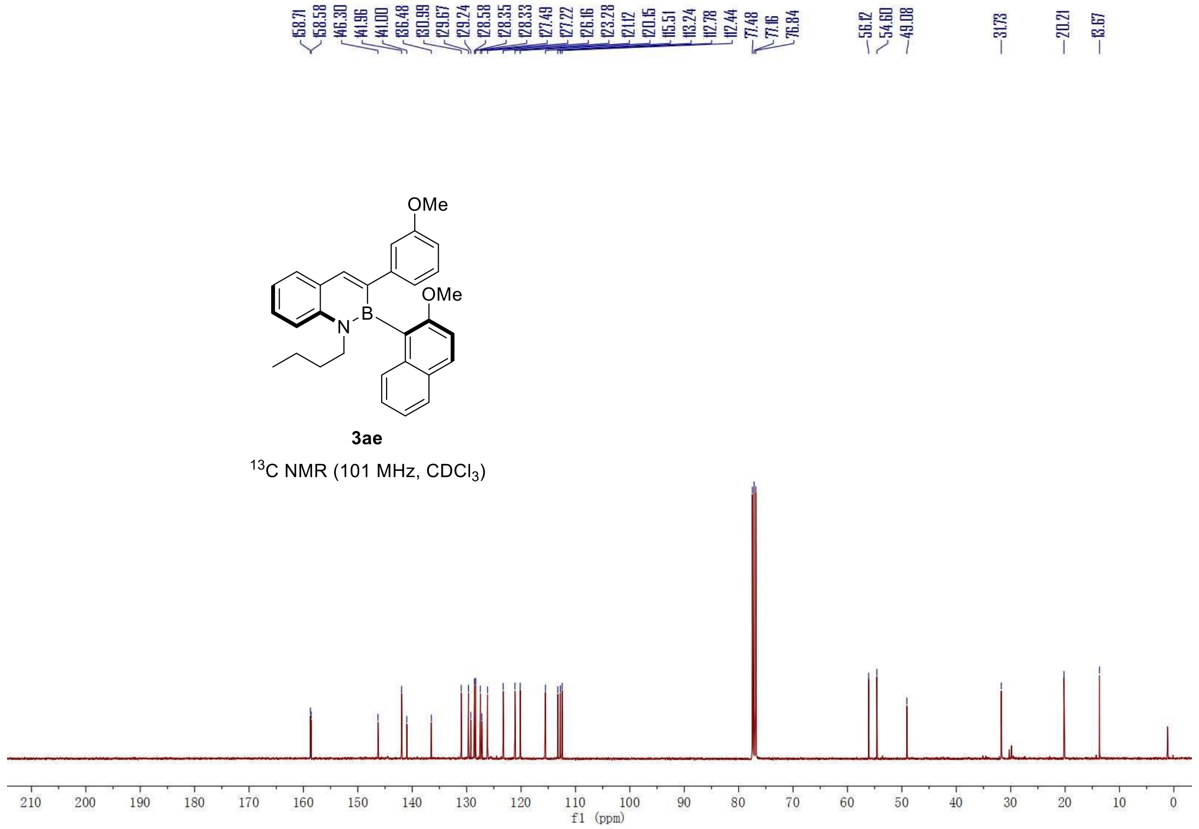

Supplementary Figure 191. ^{13}C NMR spectrum of 3ae

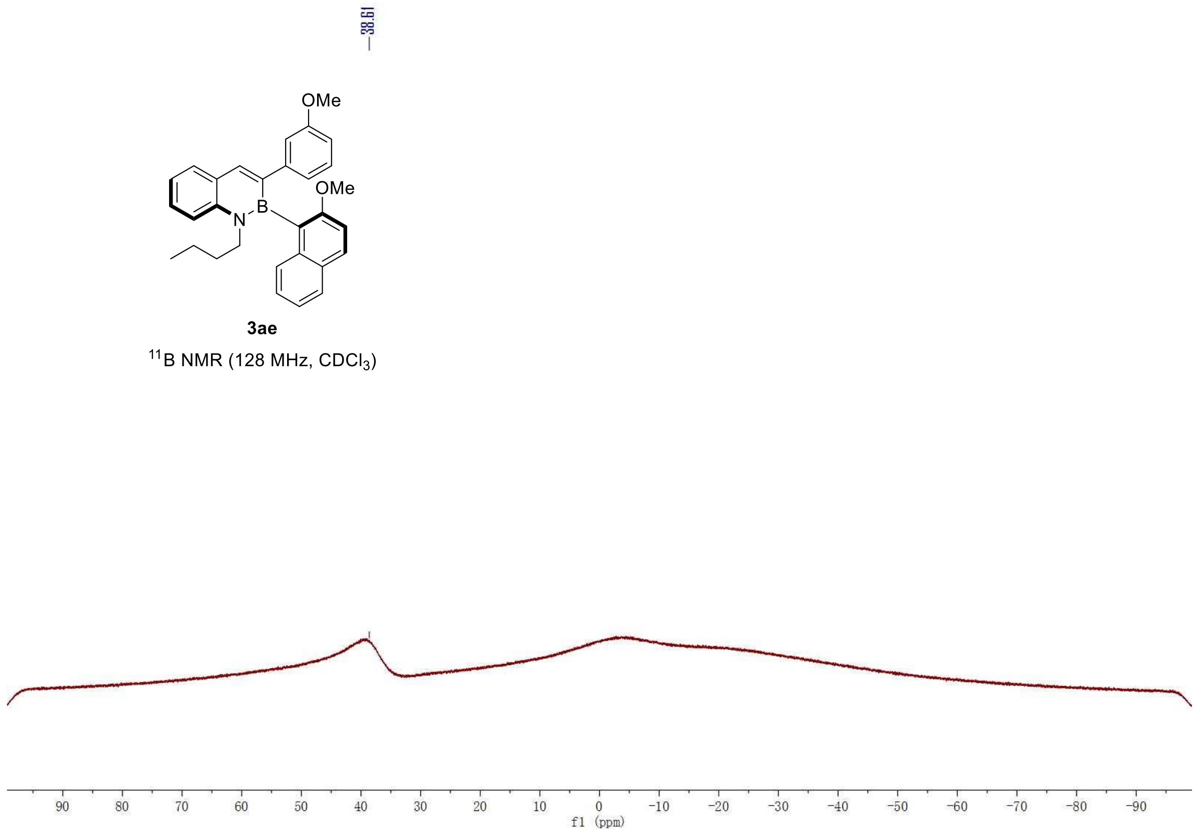

Supplementary Figure 192. ^{11}B NMR spectrum of 3ae

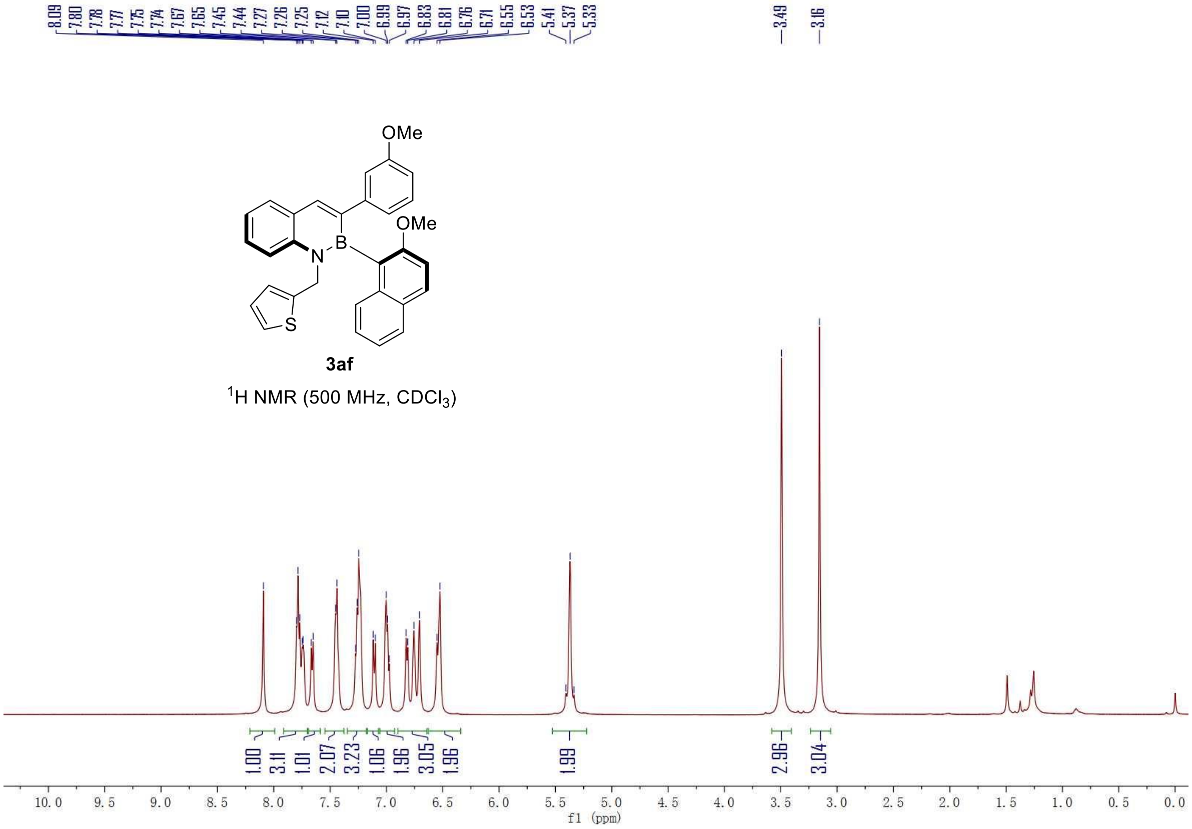

Supplementary Figure 193. ^{1}H NMR spectrum of 3af

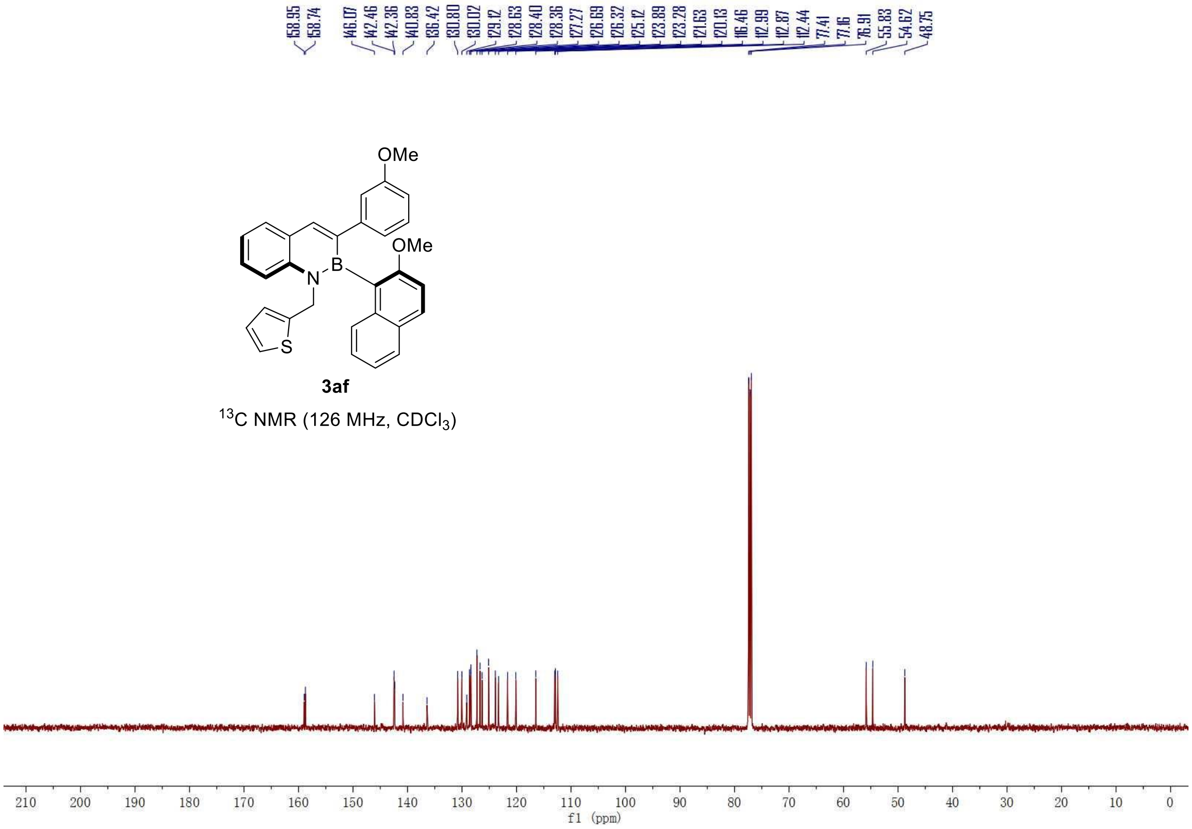

Supplementary Figure 194. ^{13}C NMR spectrum of 3af

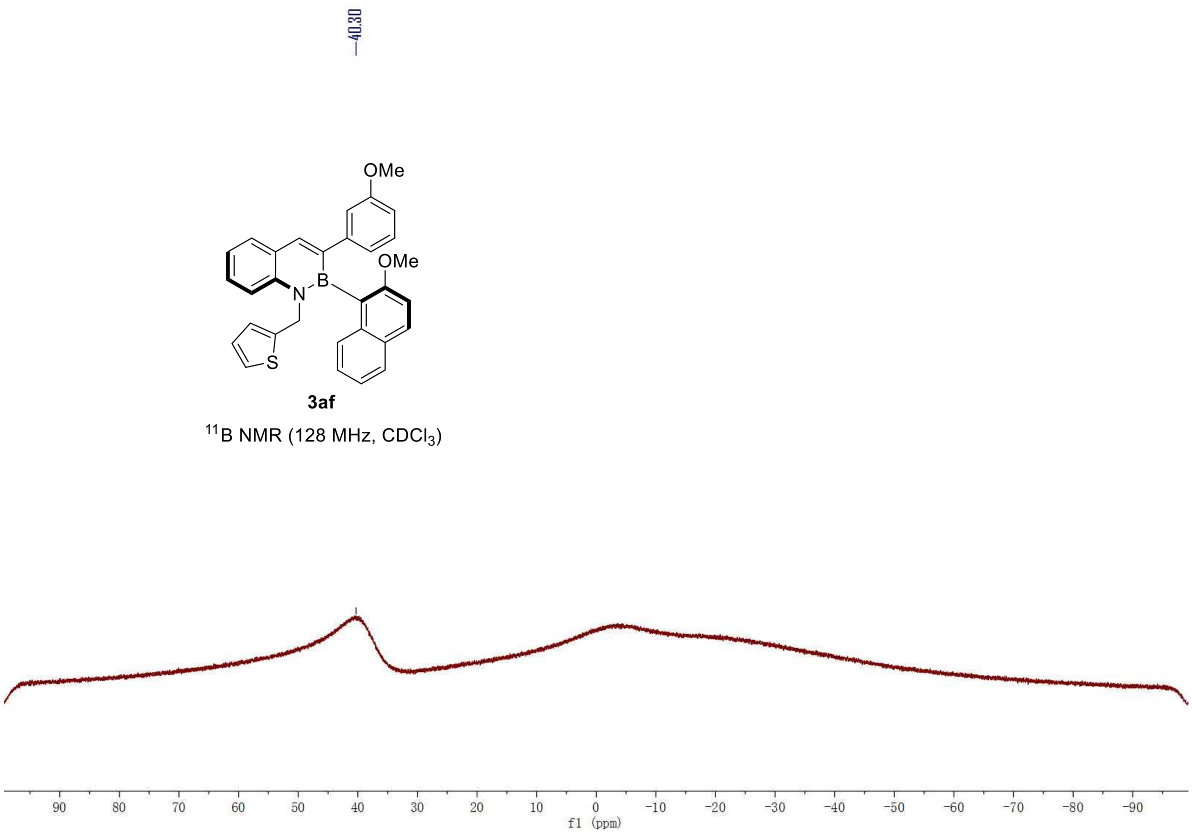

Supplementary Figure 195. ^{11}B NMR spectrum of 3af

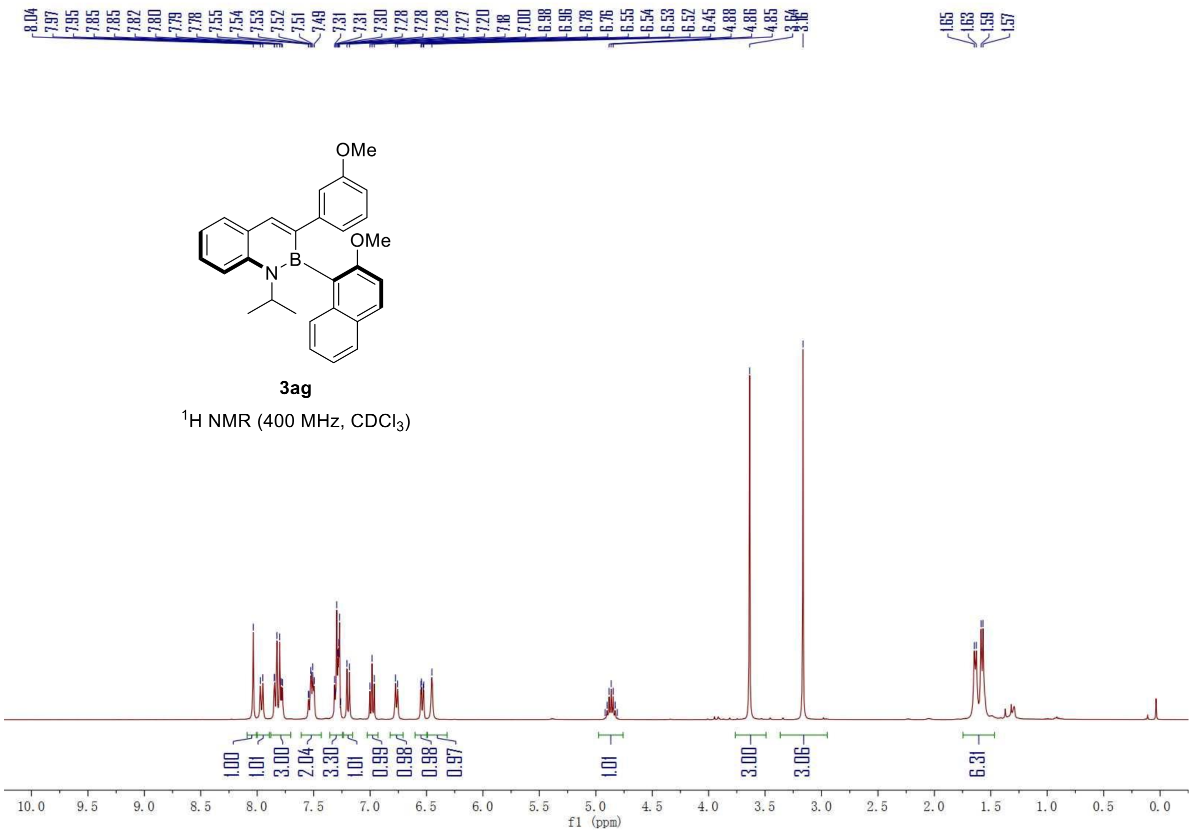

Supplementary Figure 196. ^{11}B NMR spectrum of 3ag

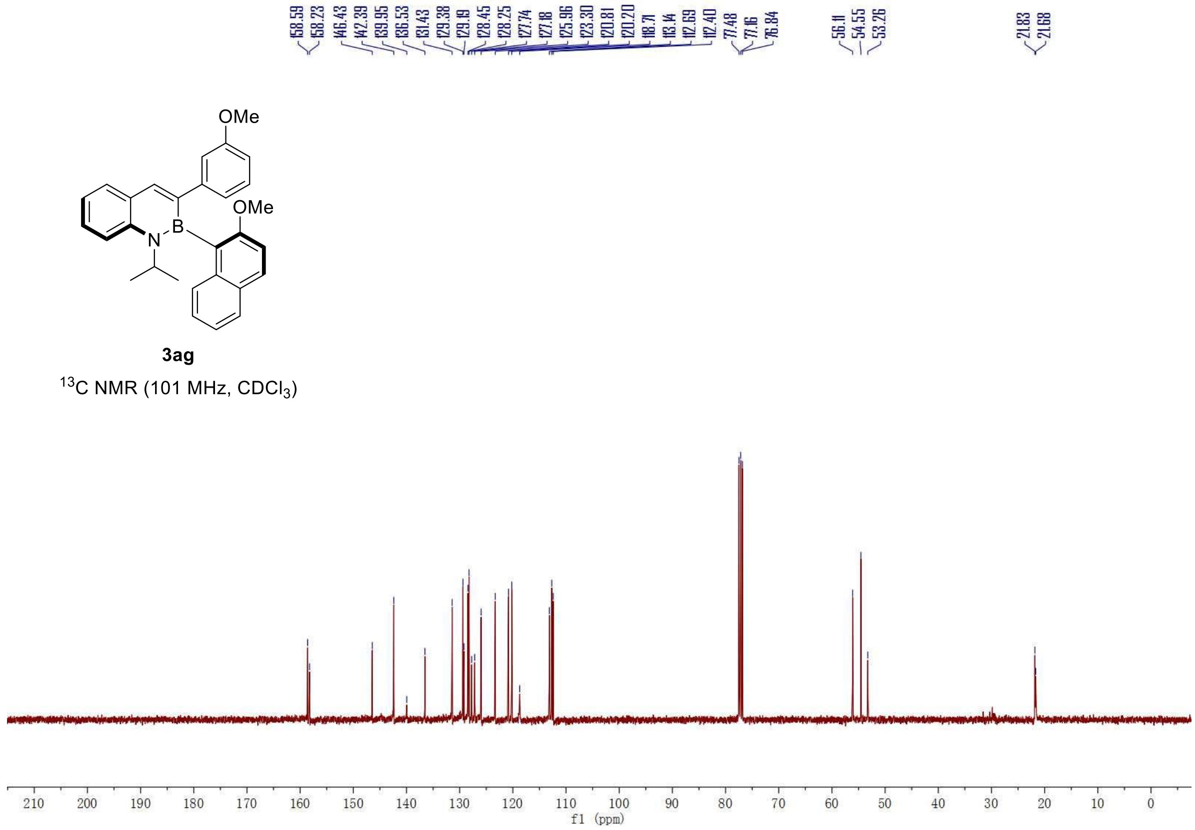

Supplementary Figure 197. ^{13}C NMR spectrum of 3ag

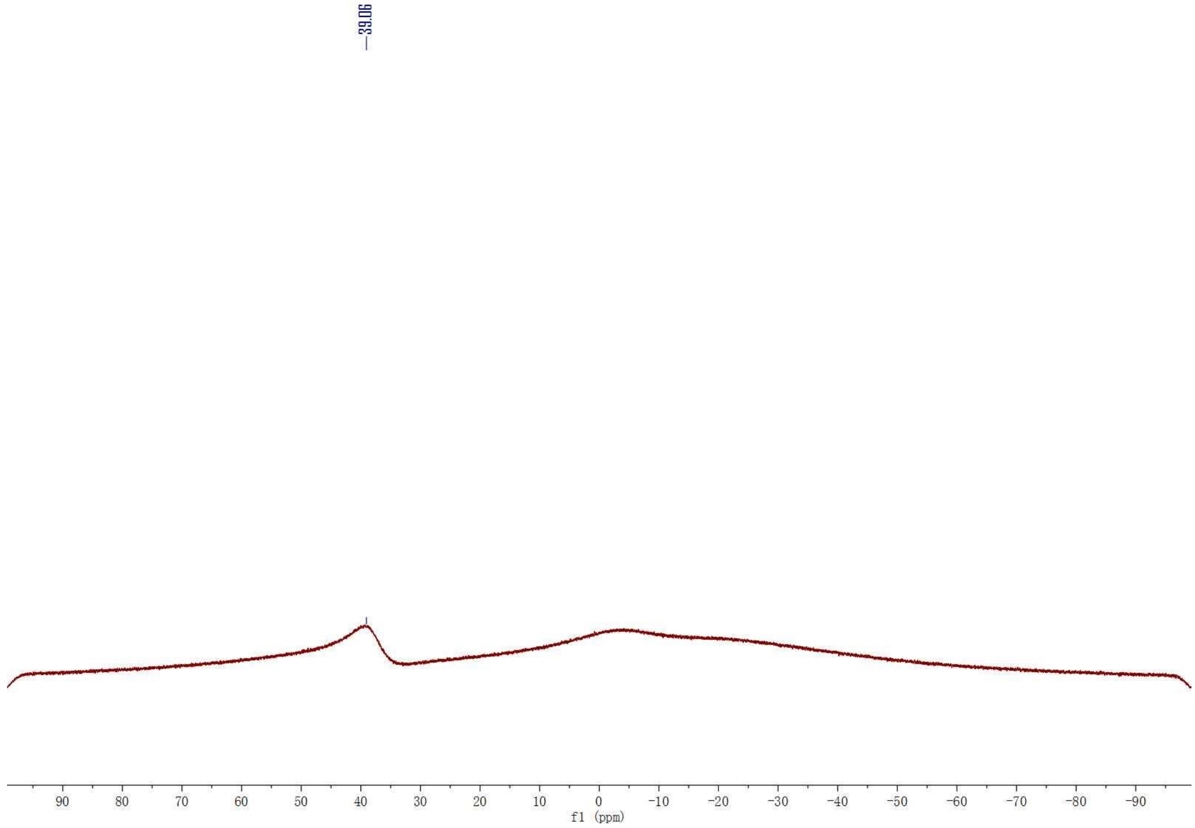

Supplementary Figure 198. ^{11}B NMR spectrum of 3ag

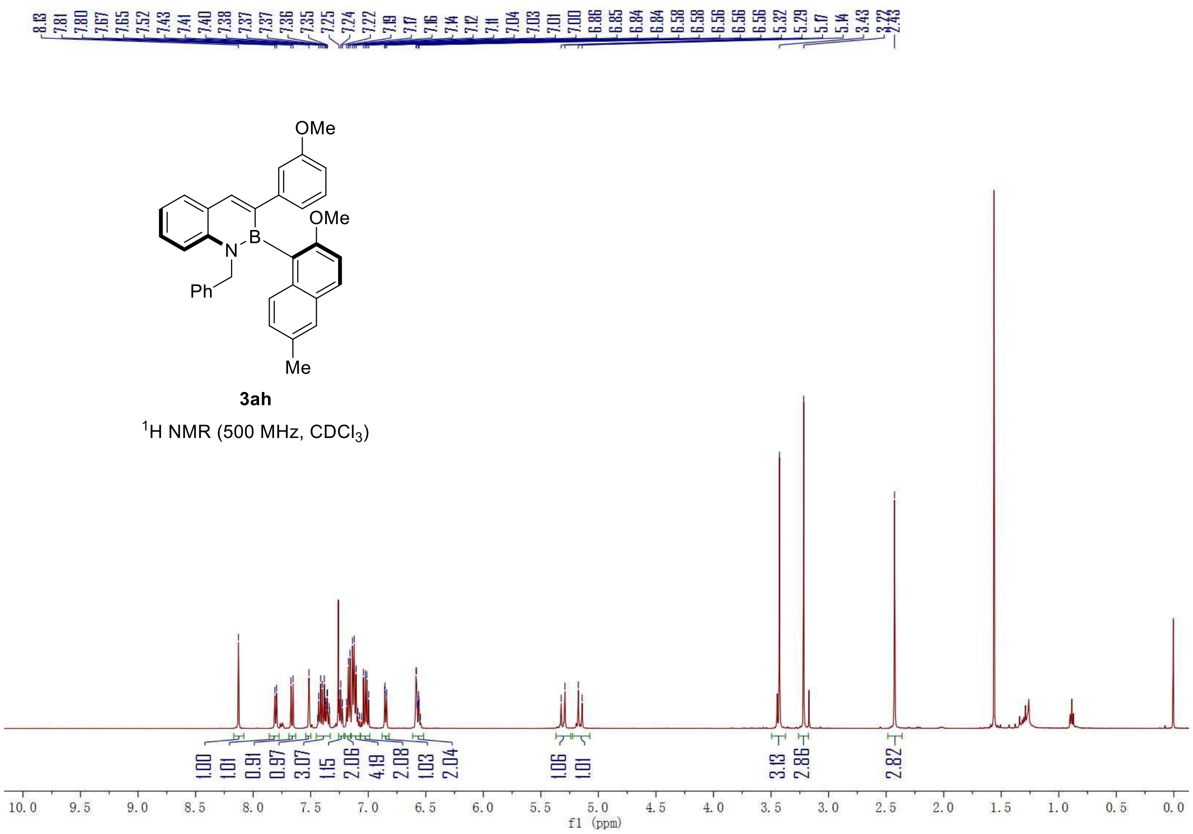

Supplementary Figure 199. ^{1}H NMR spectrum of 3ah

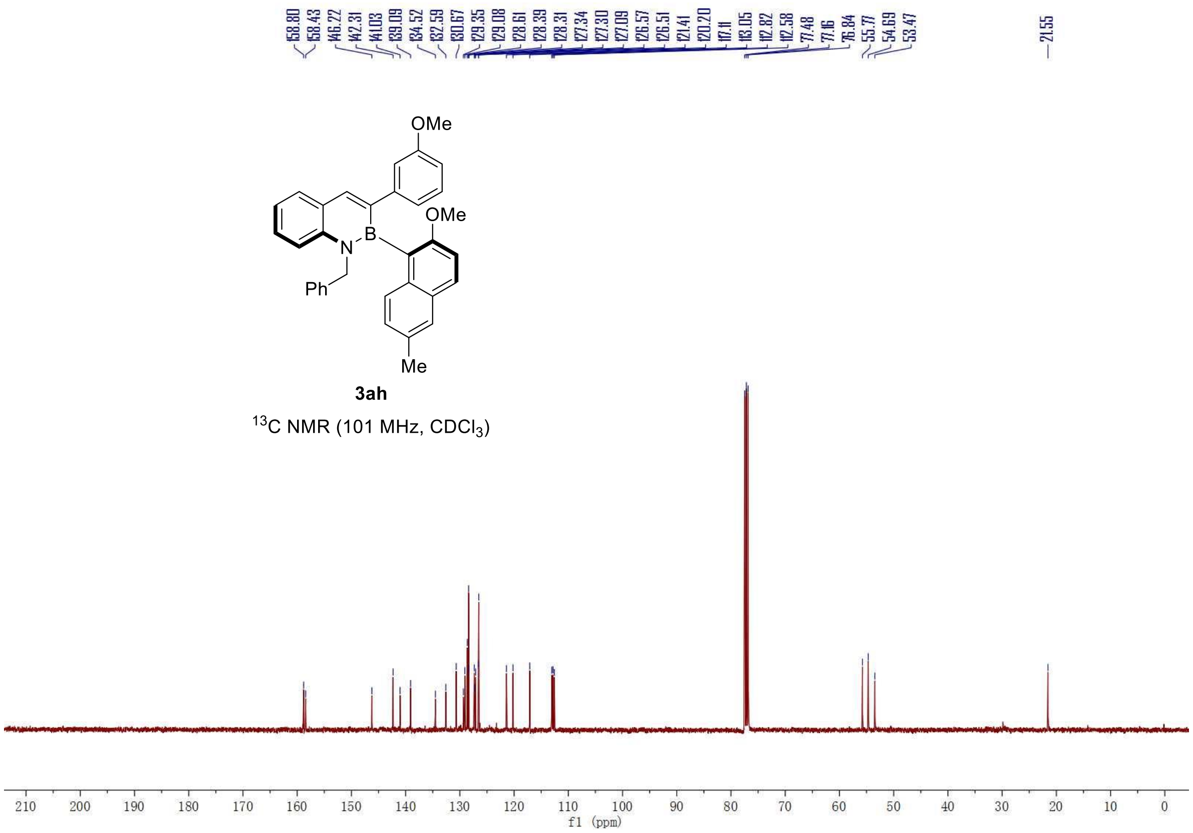

Supplementary Figure 200. ^{13}C NMR spectrum of 3ah

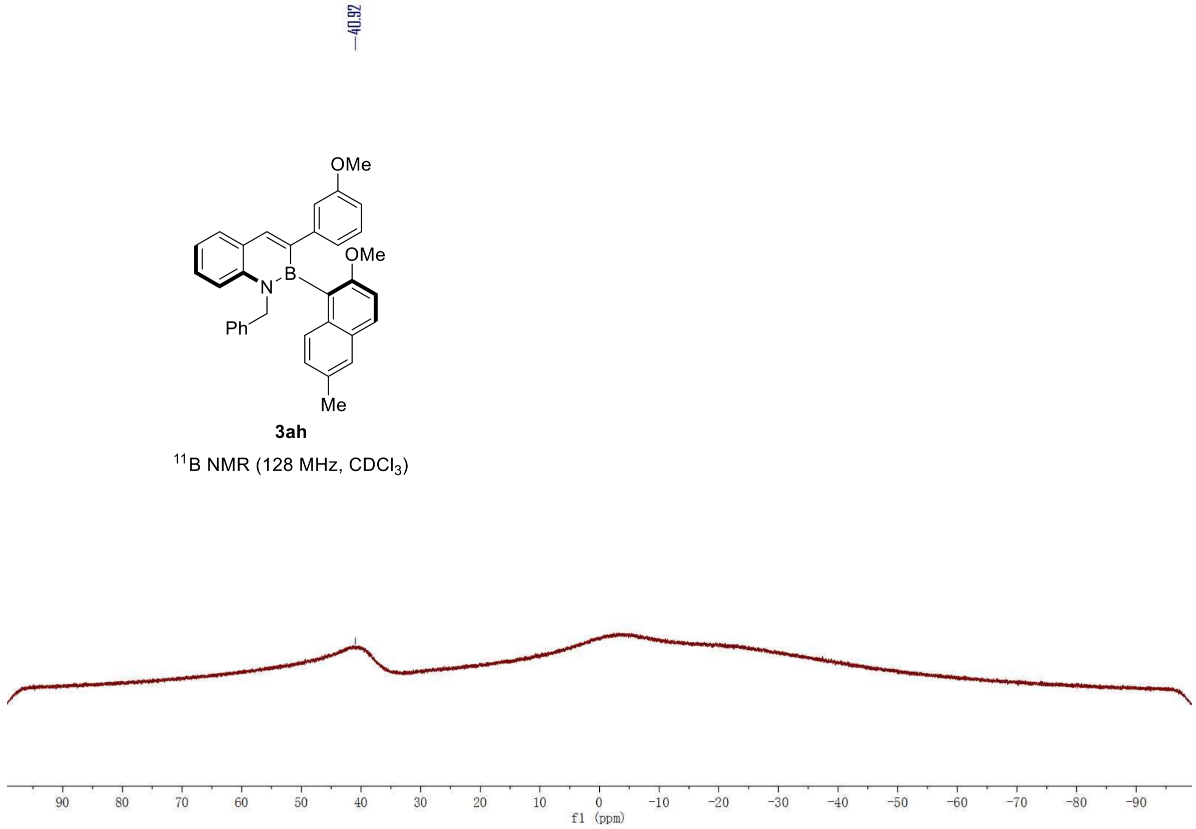

Supplementary Figure 201. ^{11}B NMR spectrum of 3ah

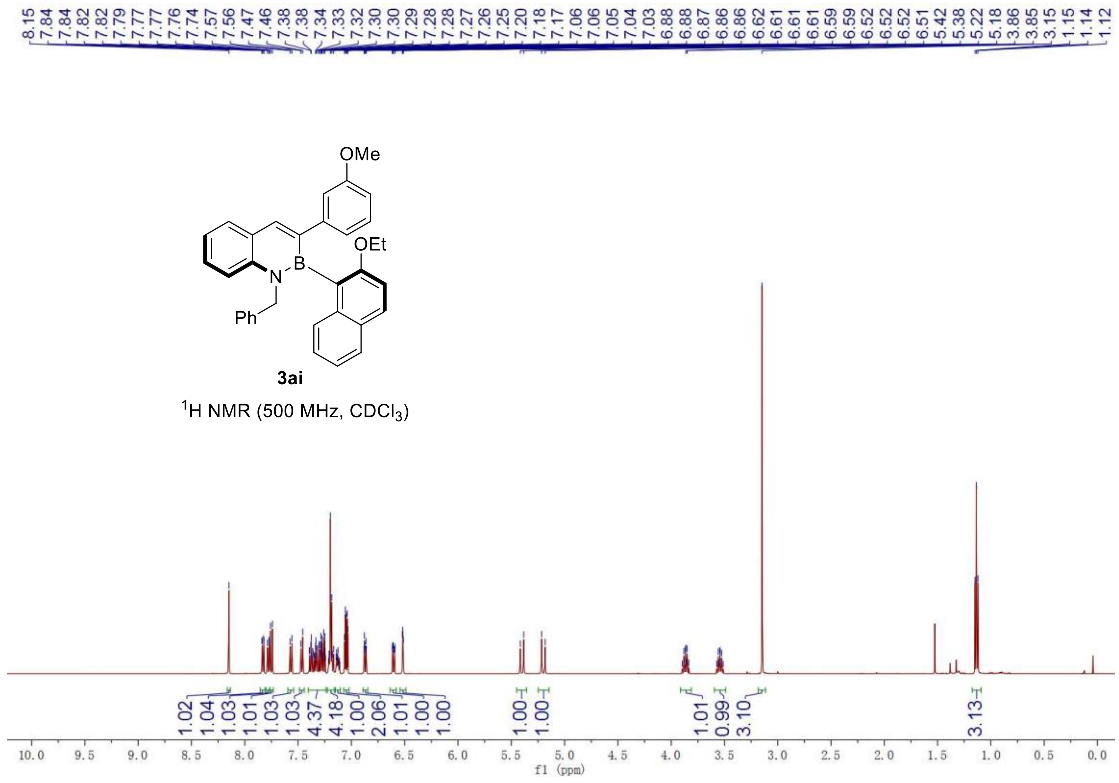

Supplementary Figure 202. ^{1}H NMR spectrum of 3ai

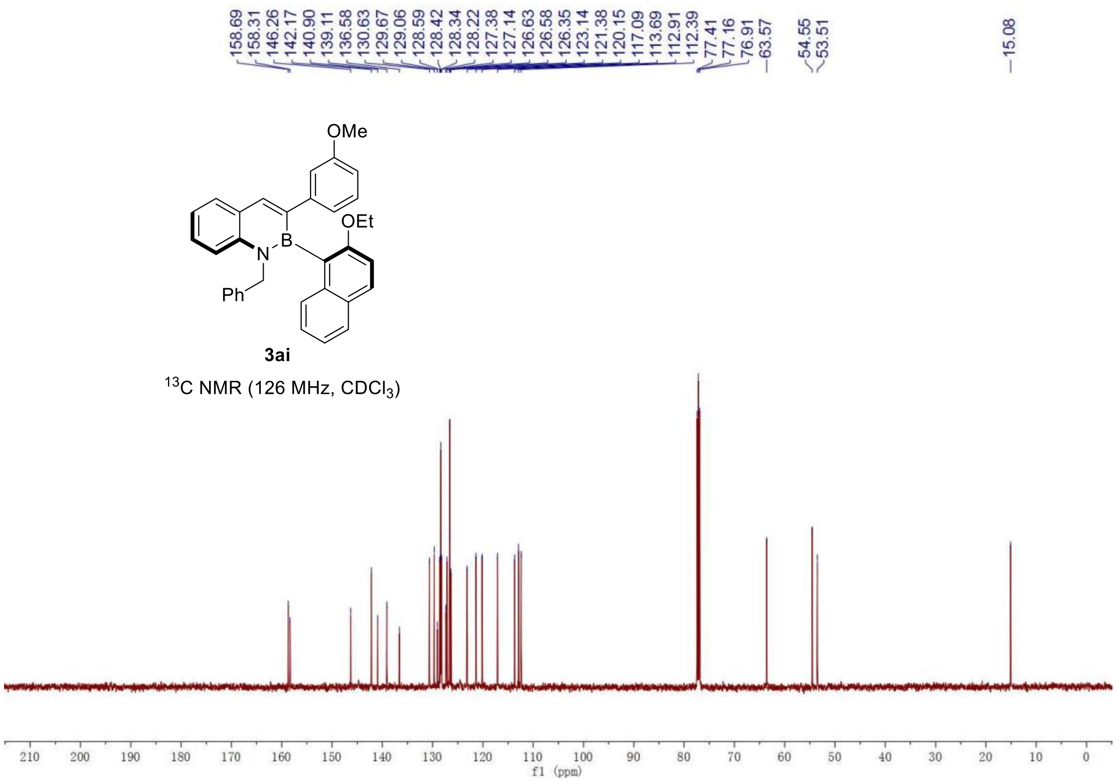

Supplementary Figure 203. ^{13}C NMR spectrum of 3ai

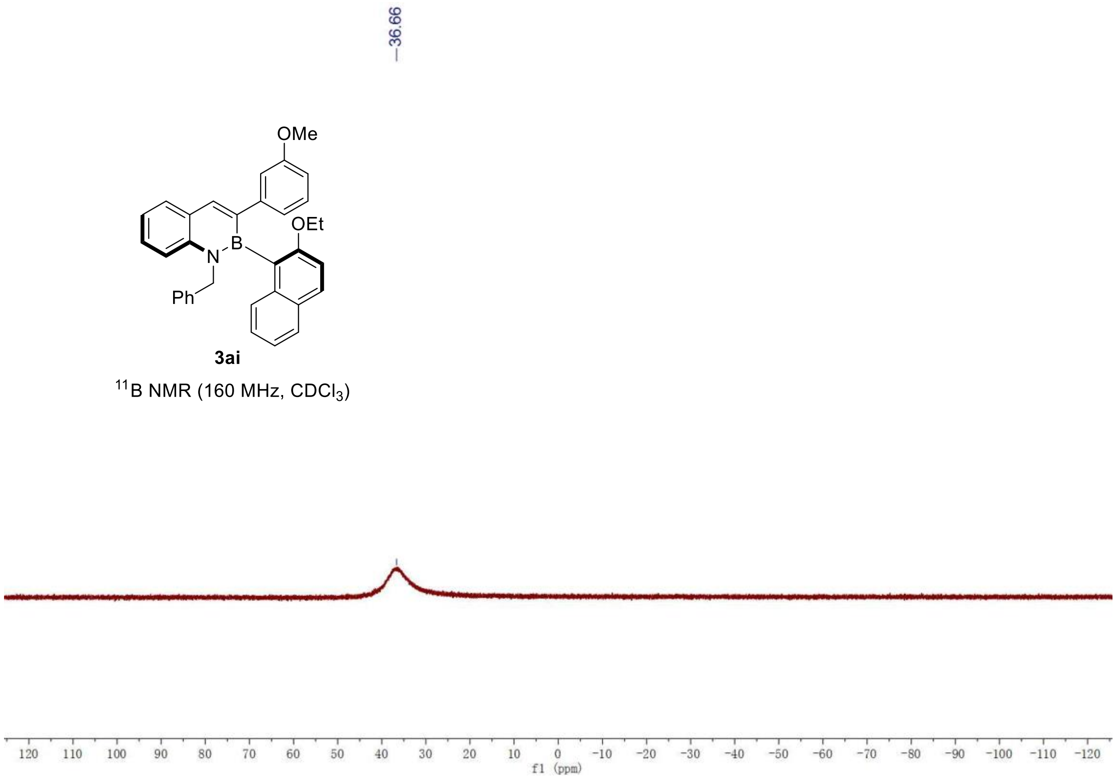

Supplementary Figure 204. ^{11}B NMR spectrum of 3ai

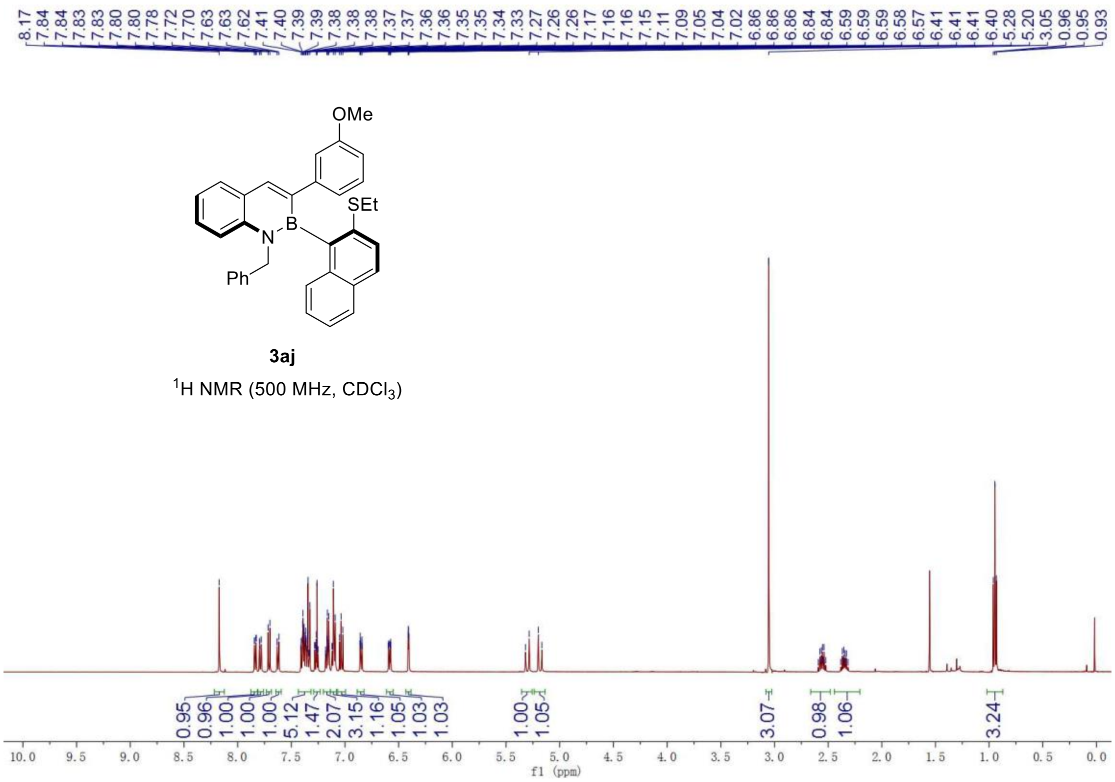

Supplementary Figure 205. ^{1}H NMR spectrum of 3aj

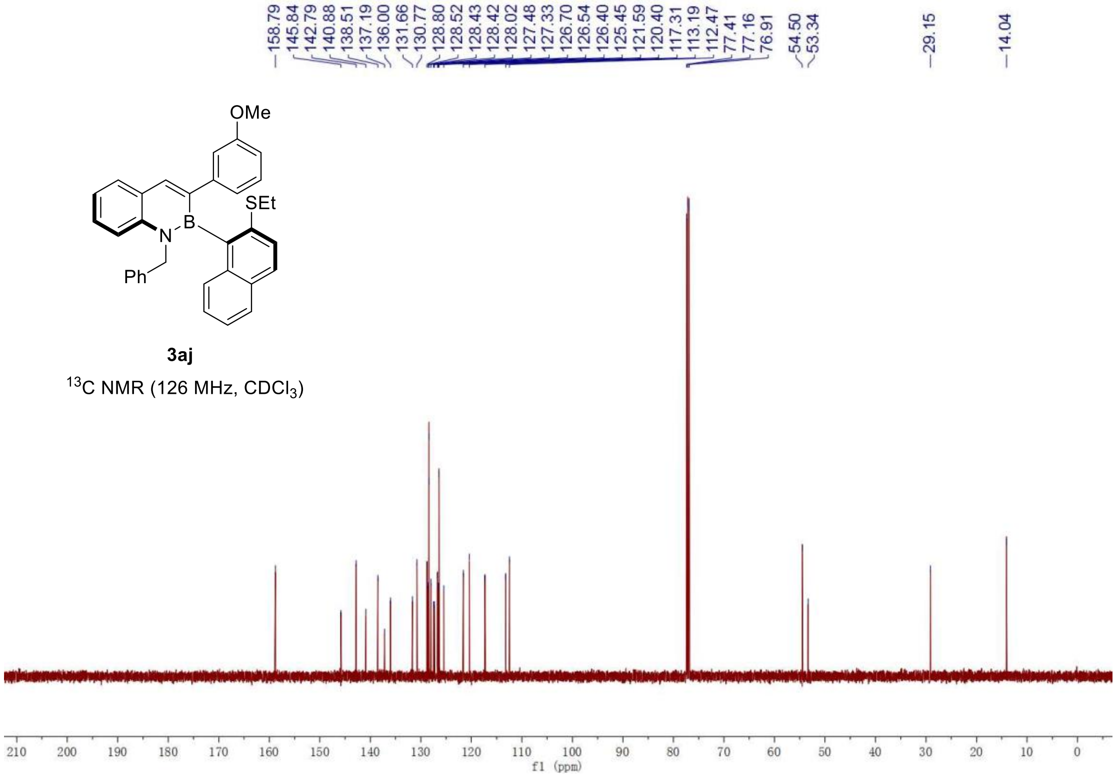

Supplementary Figure 206. ^{13}C NMR spectrum of 3aj

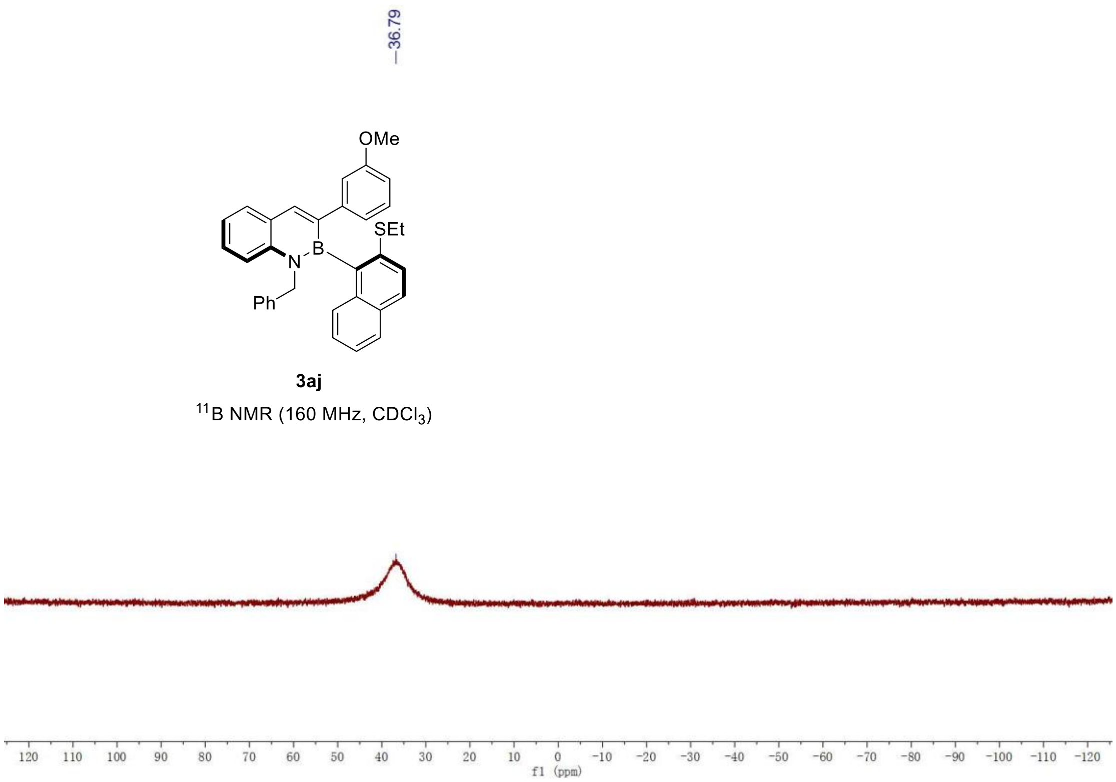

Supplementary Figure 207. ^{11}B NMR spectrum of 3aj

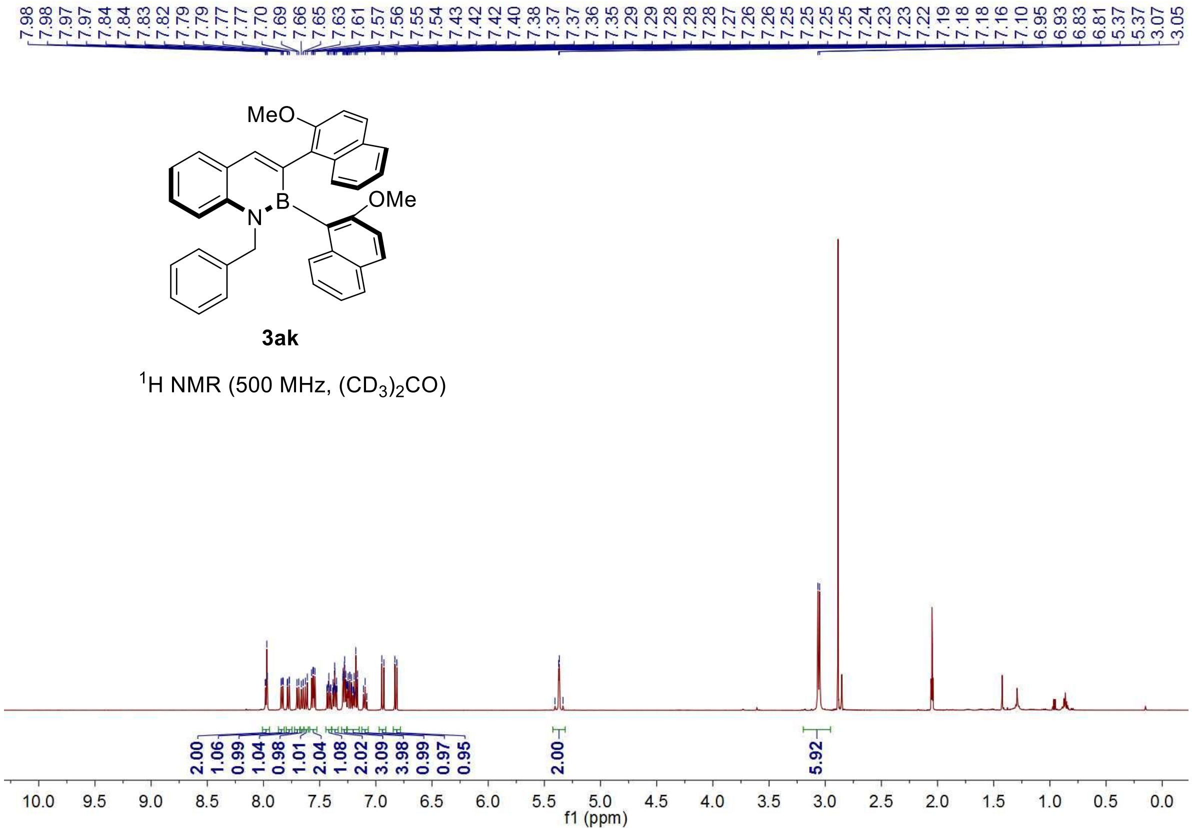

Supplementary Figure 208. ^{1}H NMR spectrum of 3ak

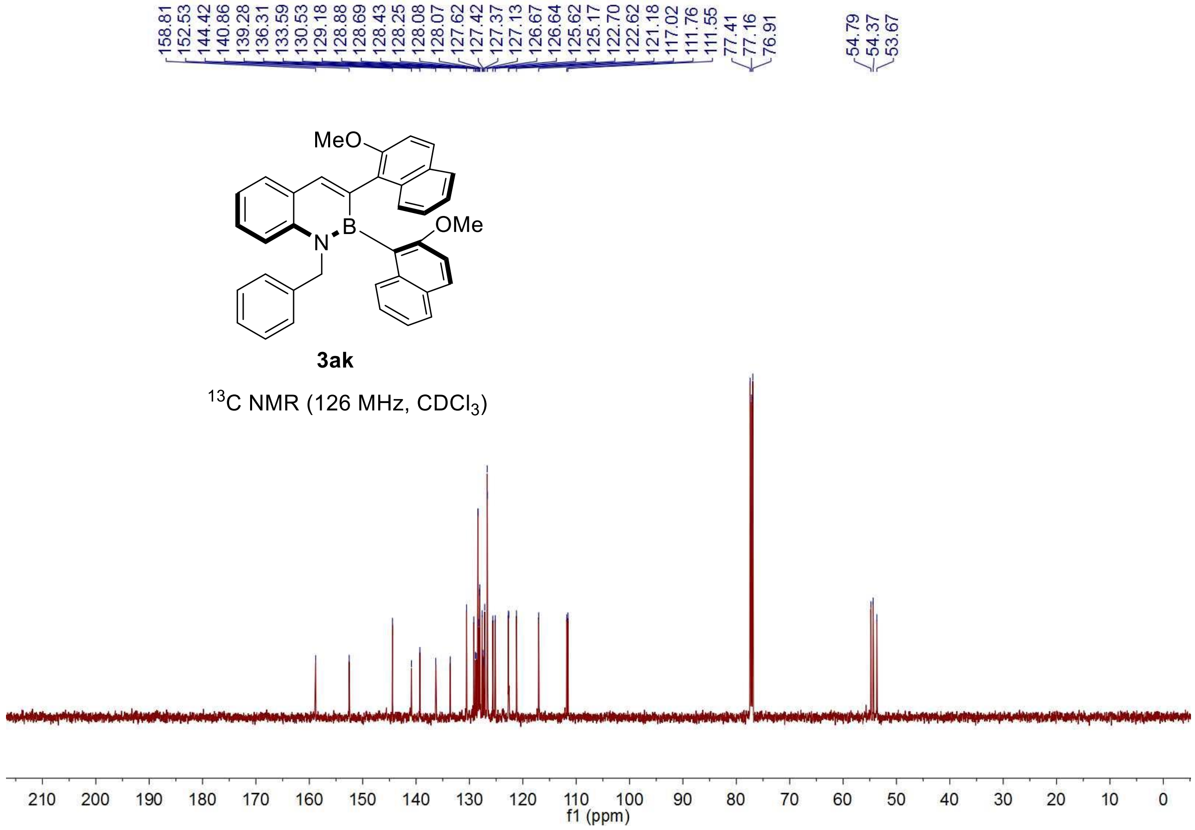

Supplementary Figure 209. ^{13}C NMR spectrum of 3ak

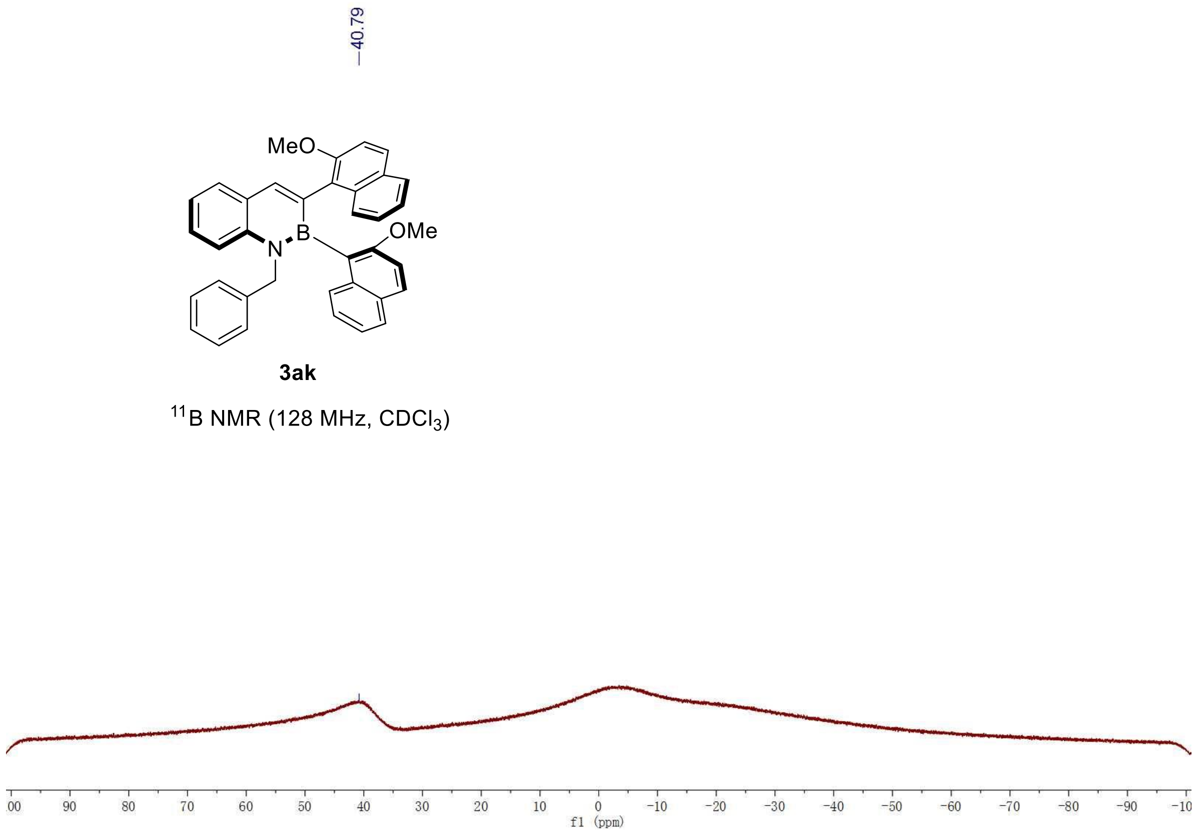

Supplementary Figure 210. ^{11}B NMR spectrum of 3ak

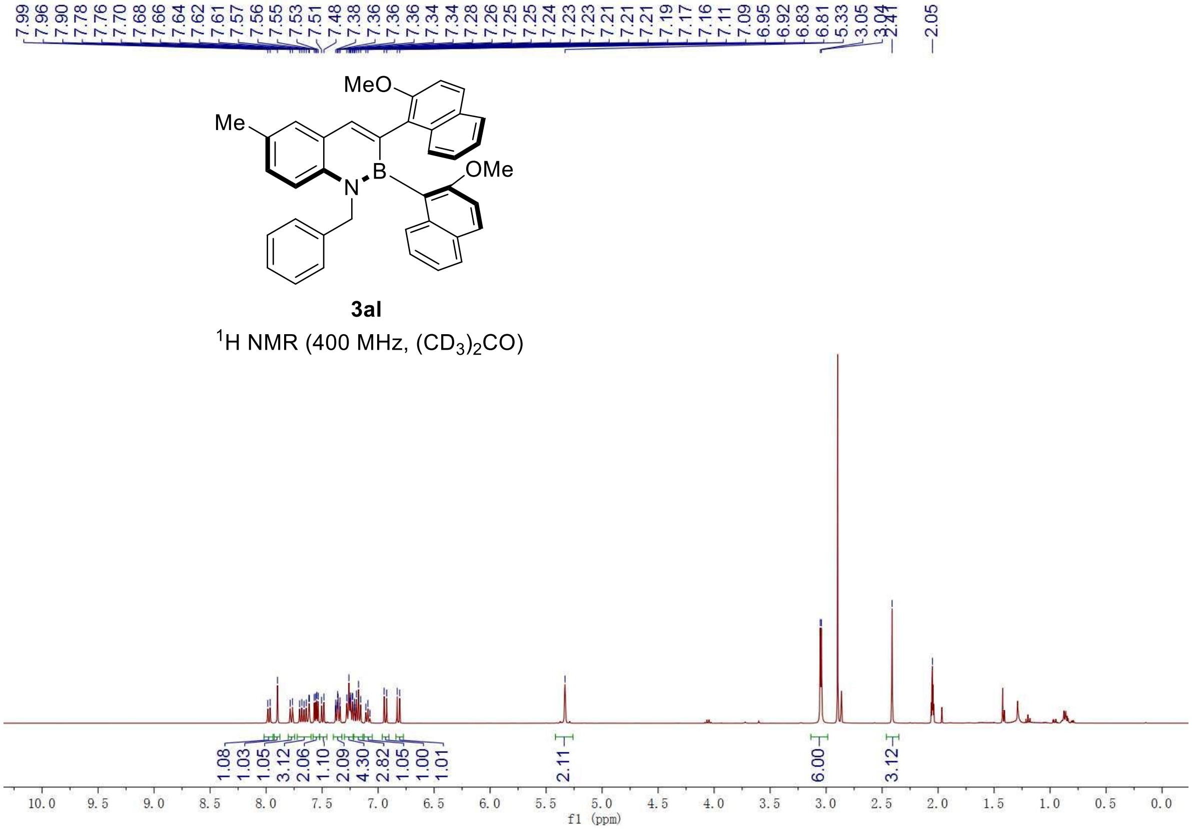

Supplementary Figure 211. ^{1}H NMR spectrum of 3al

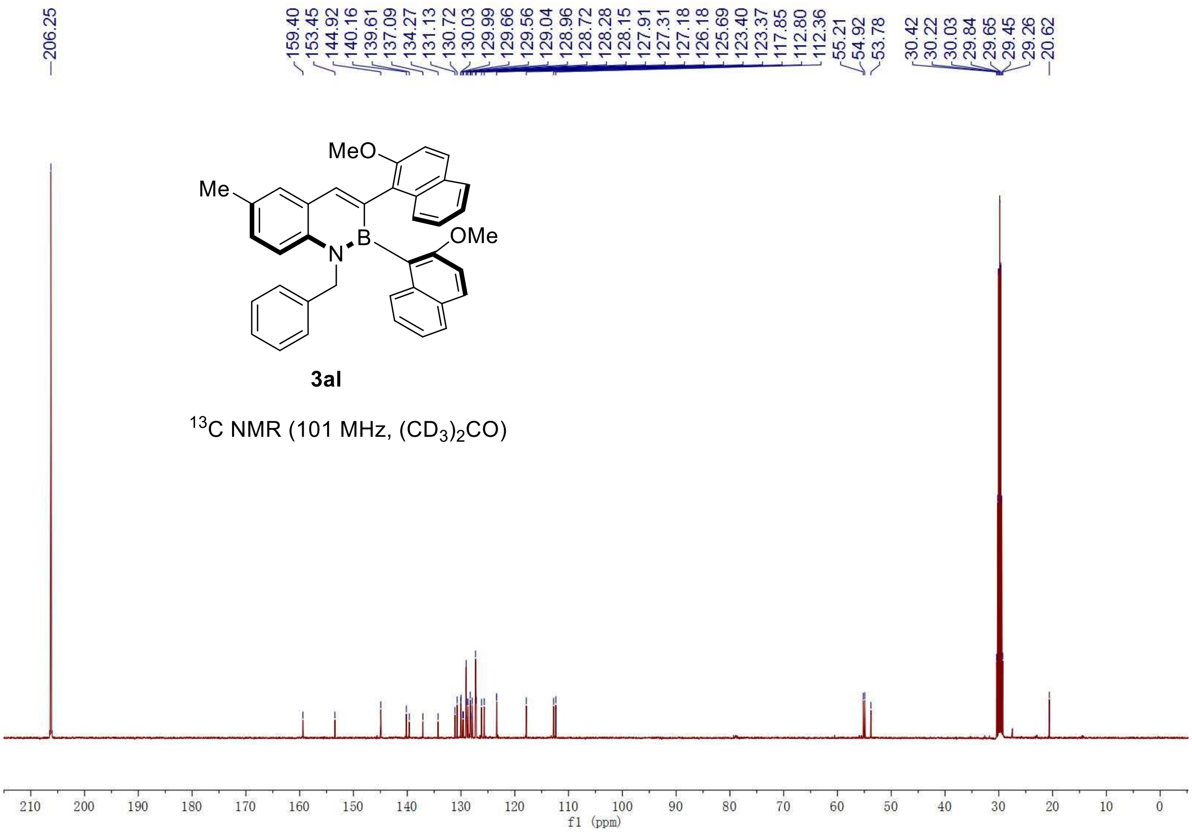

Supplementary Figure 212. ^{13}C NMR spectrum of 3al

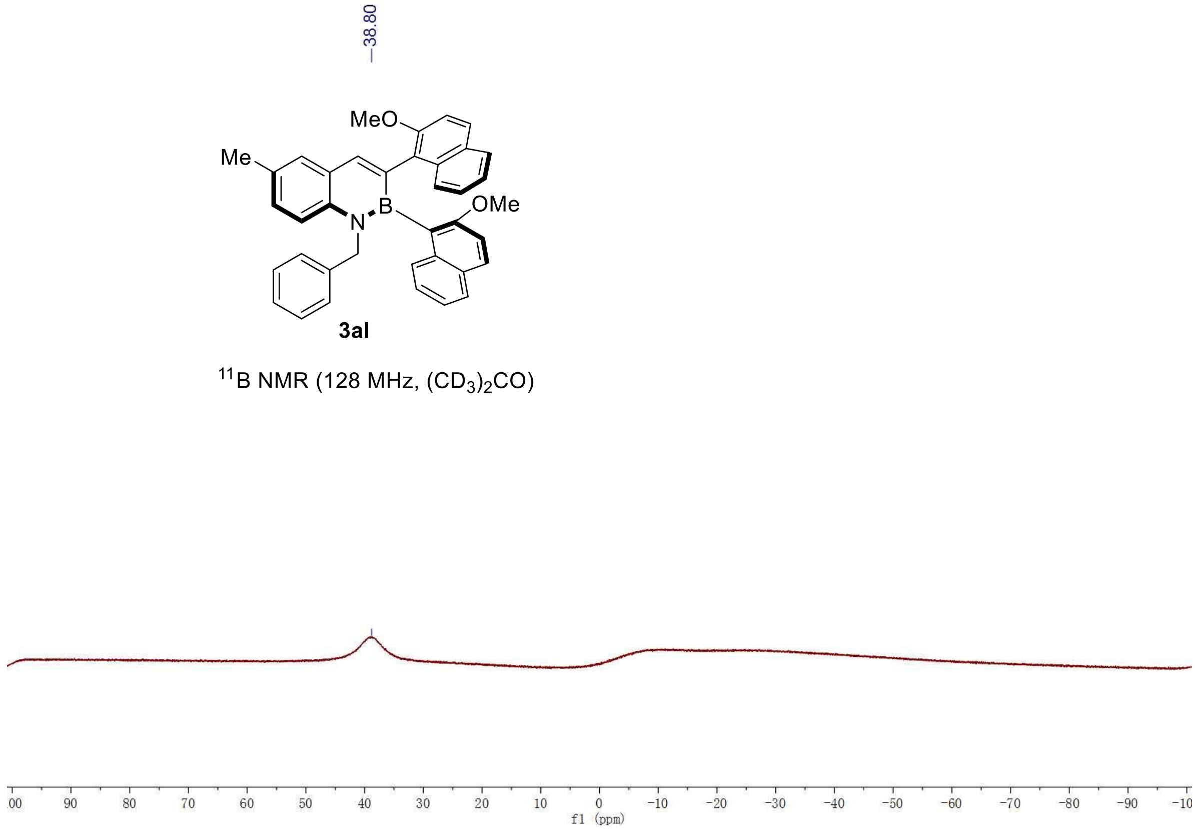

Supplementary Figure 213. ^{11}B NMR spectrum of 3al

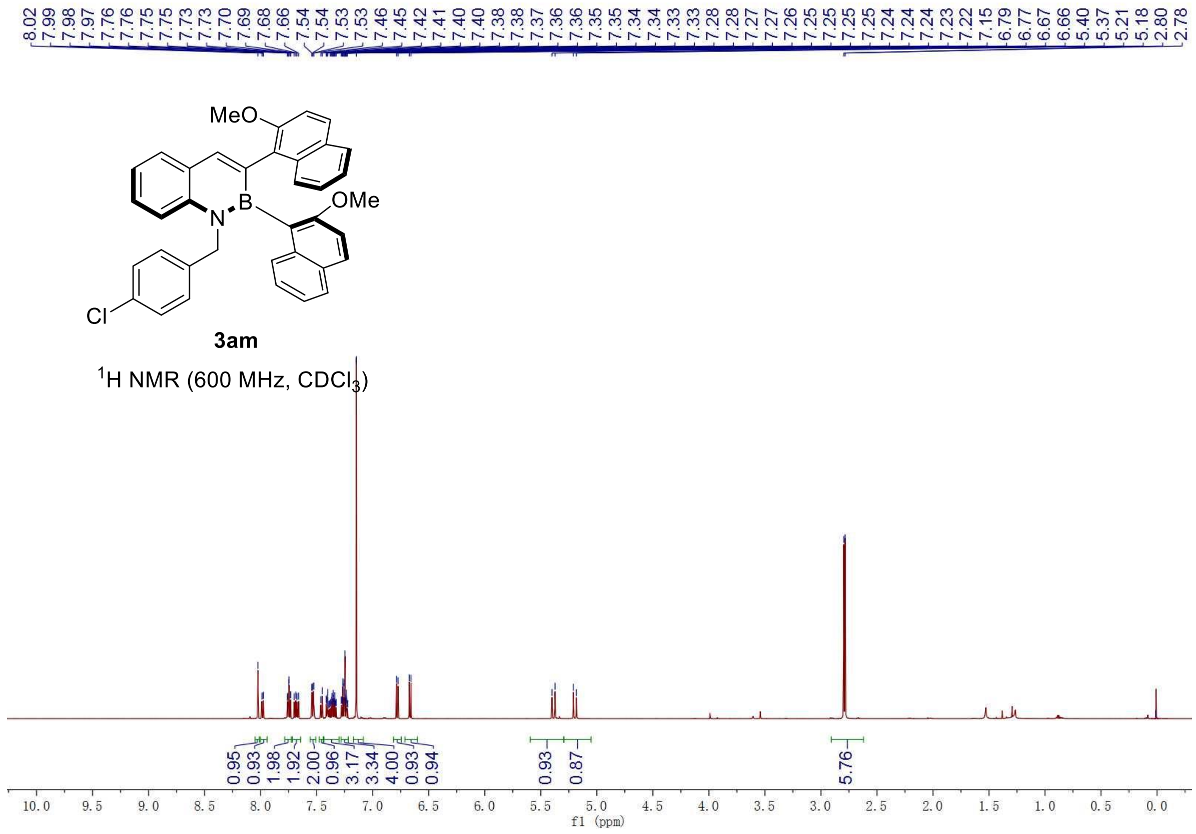

Supplementary Figure 214. ^{1}H NMR spectrum of 1a

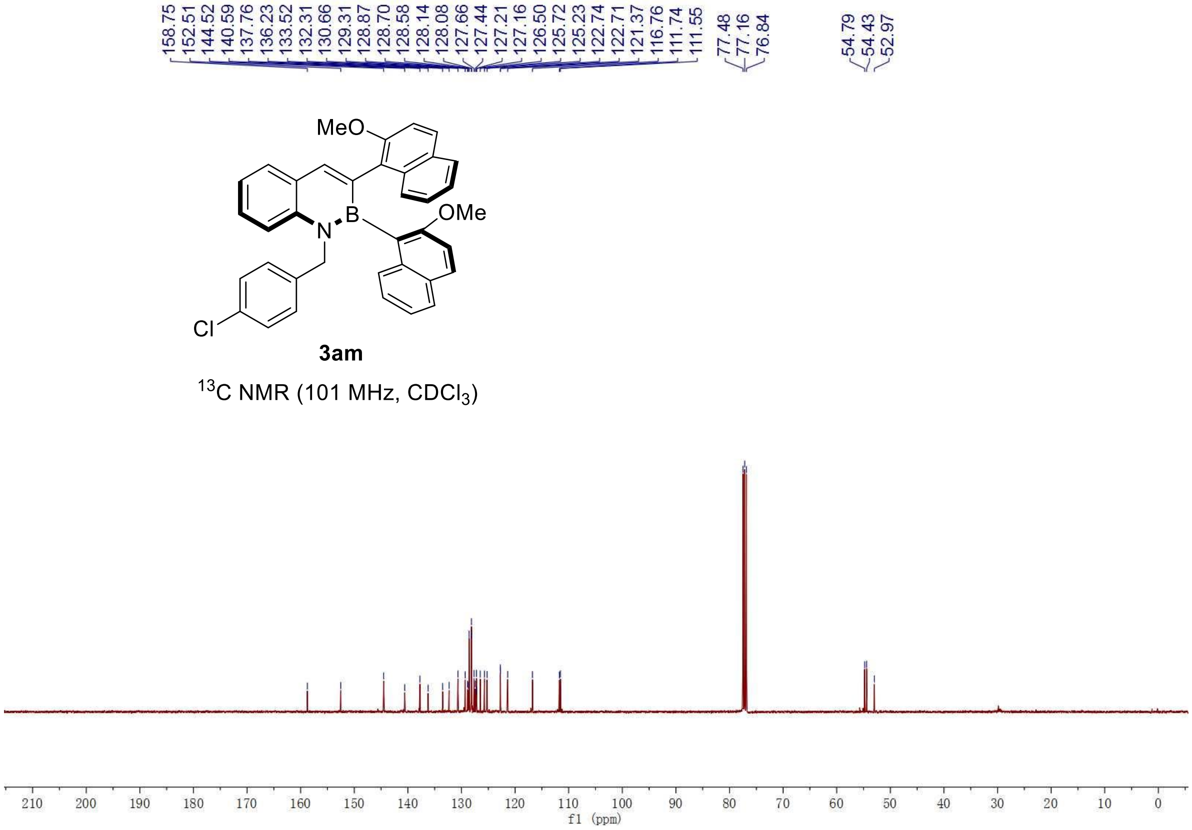

Supplementary Figure 215. ^{13}C NMR spectrum of 3am

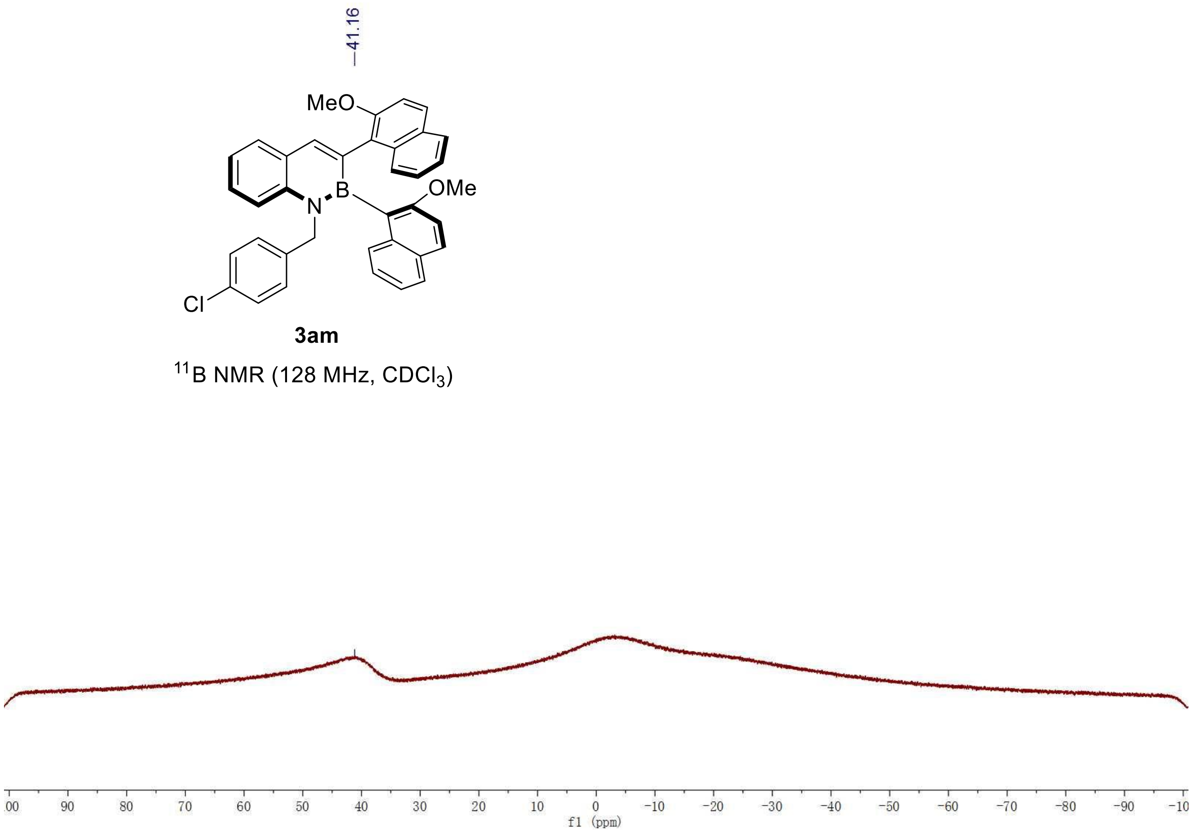

Supplementary Figure 216. ^{11}B NMR spectrum of 3am

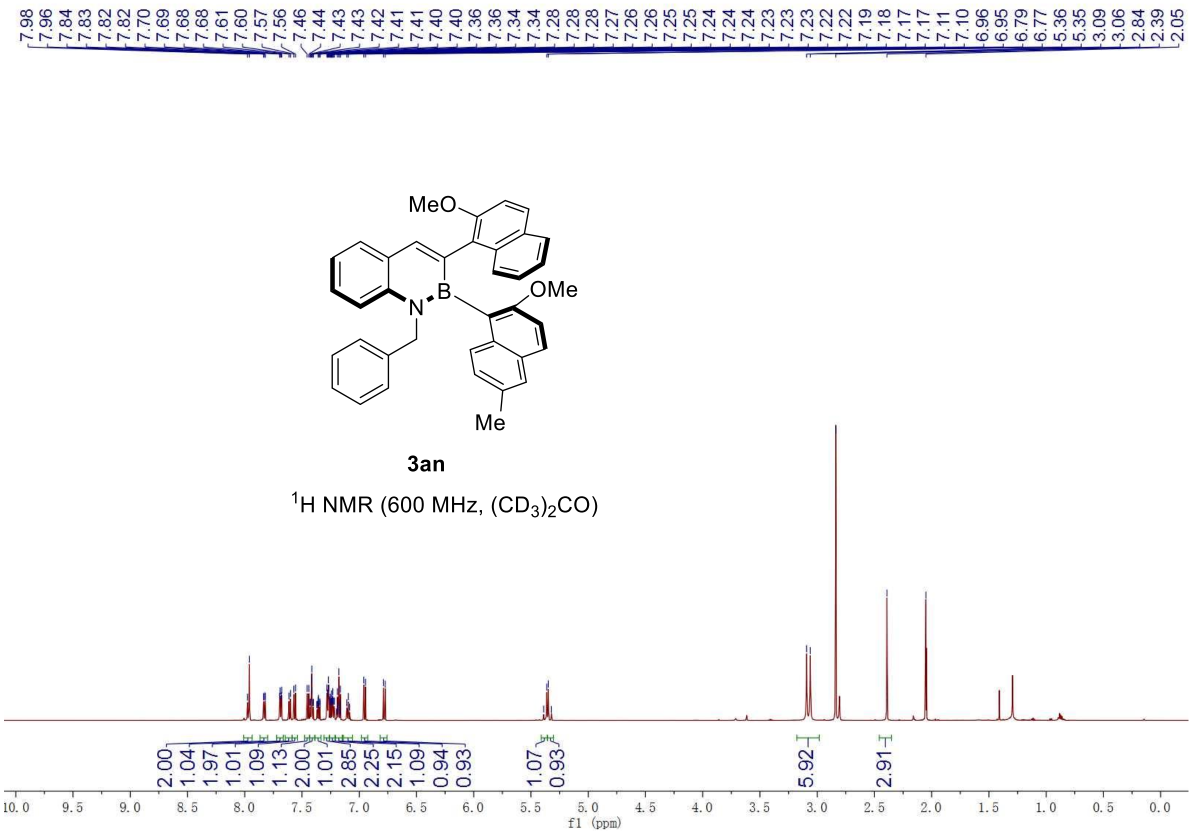

Supplementary Figure 217. **^{1}H NMR spectrum of 3an**

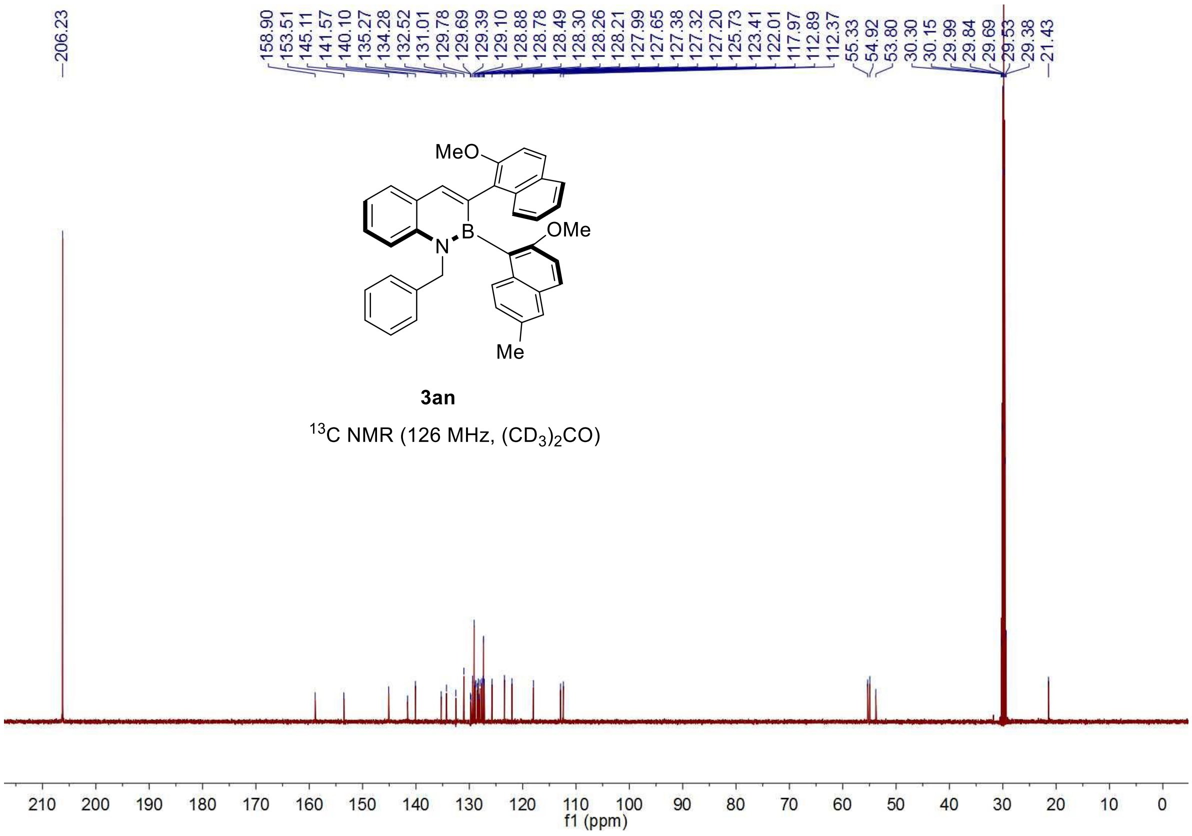

Supplementary Figure 218. **^{13}C NMR spectrum of 3an**

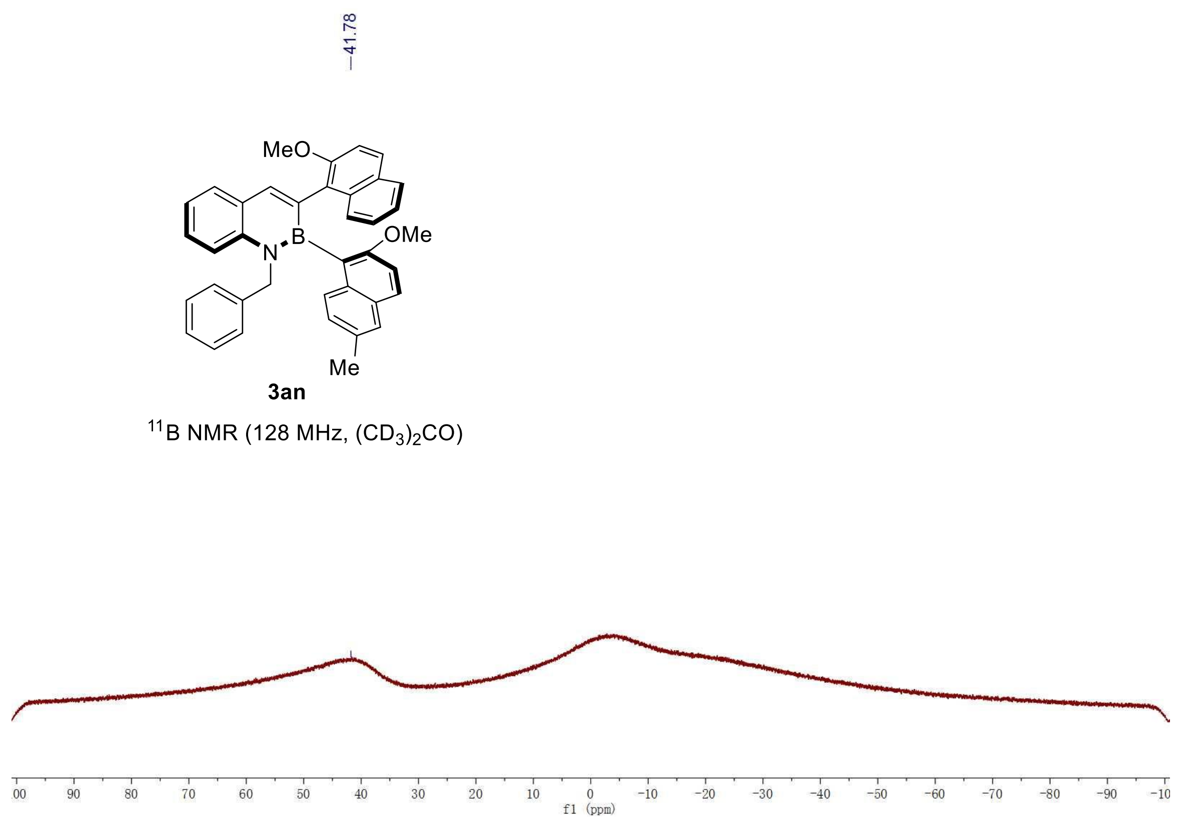

Supplementary Figure 219. ^{11}B NMR spectrum of 3an

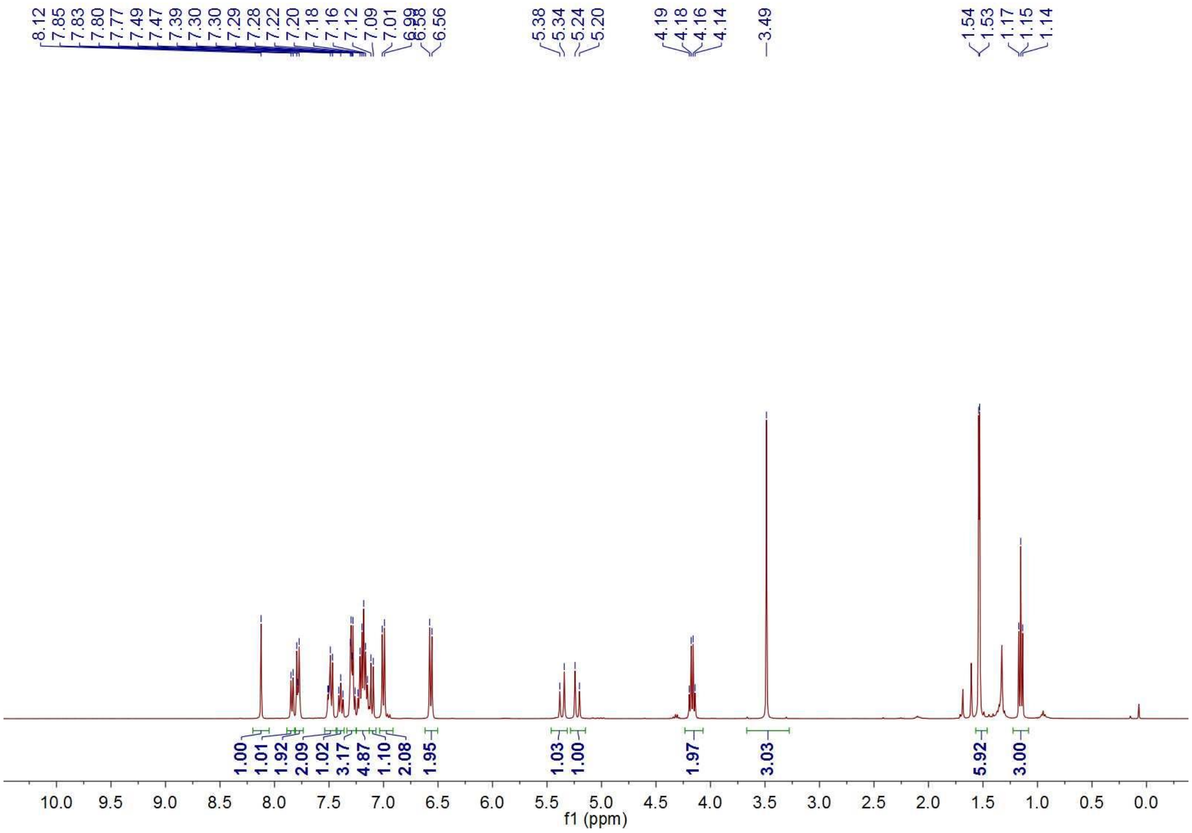

Supplementary Figure 220. ^{1}H NMR spectrum of 4a

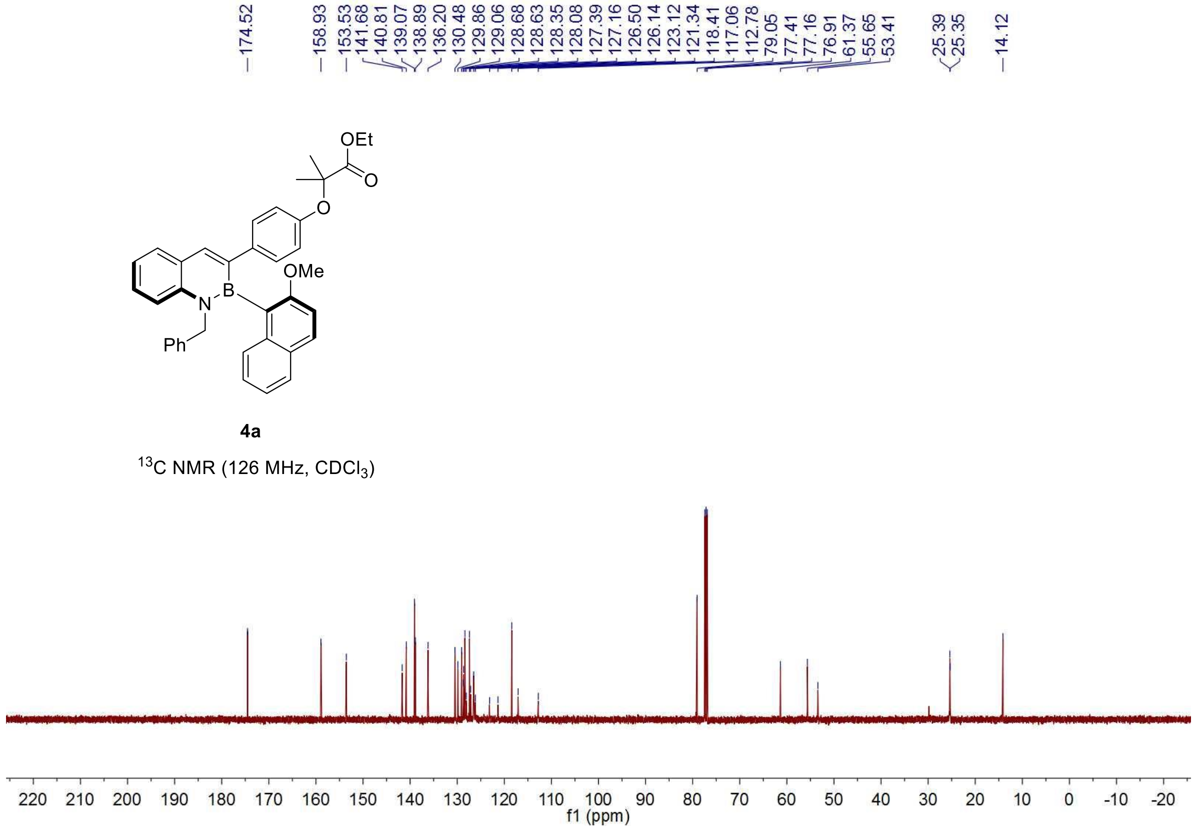

Supplementary Figure 221. ^{13}C NMR spectrum of 4a

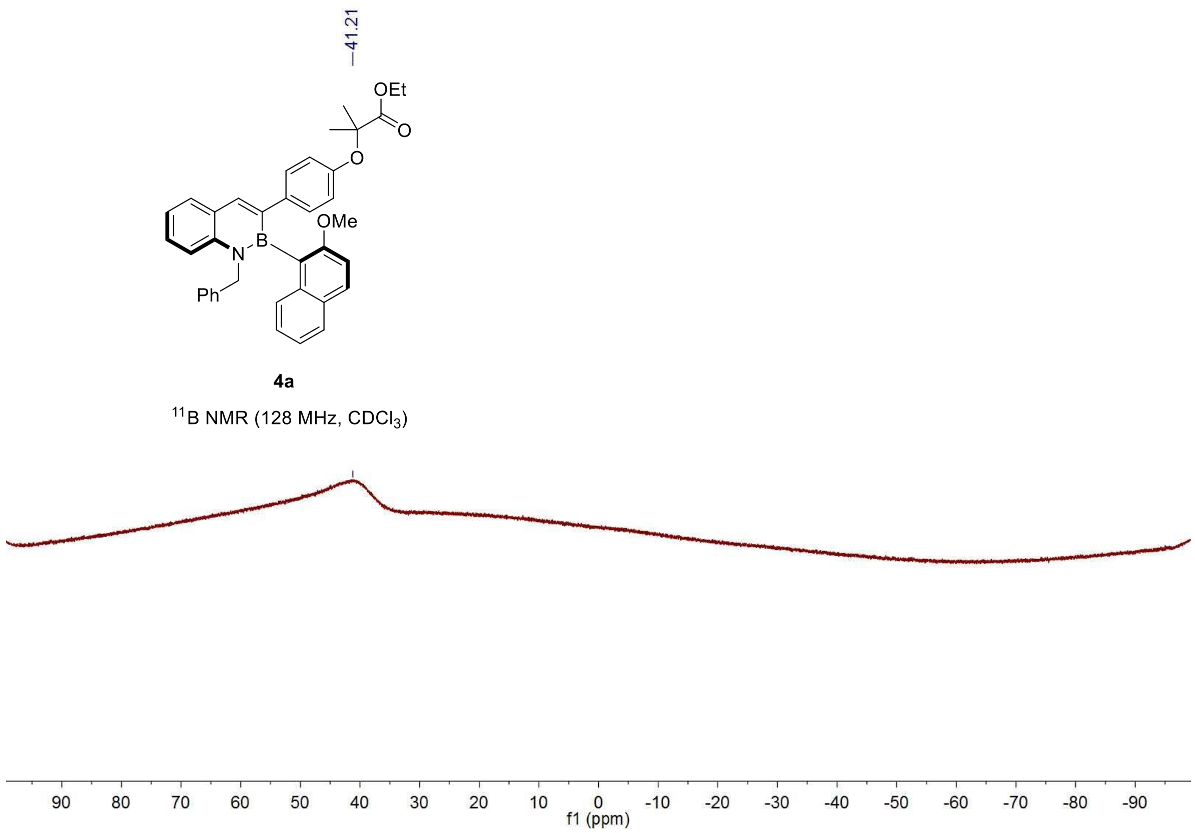

Supplementary Figure 222. ^{11}B NMR spectrum of 4a

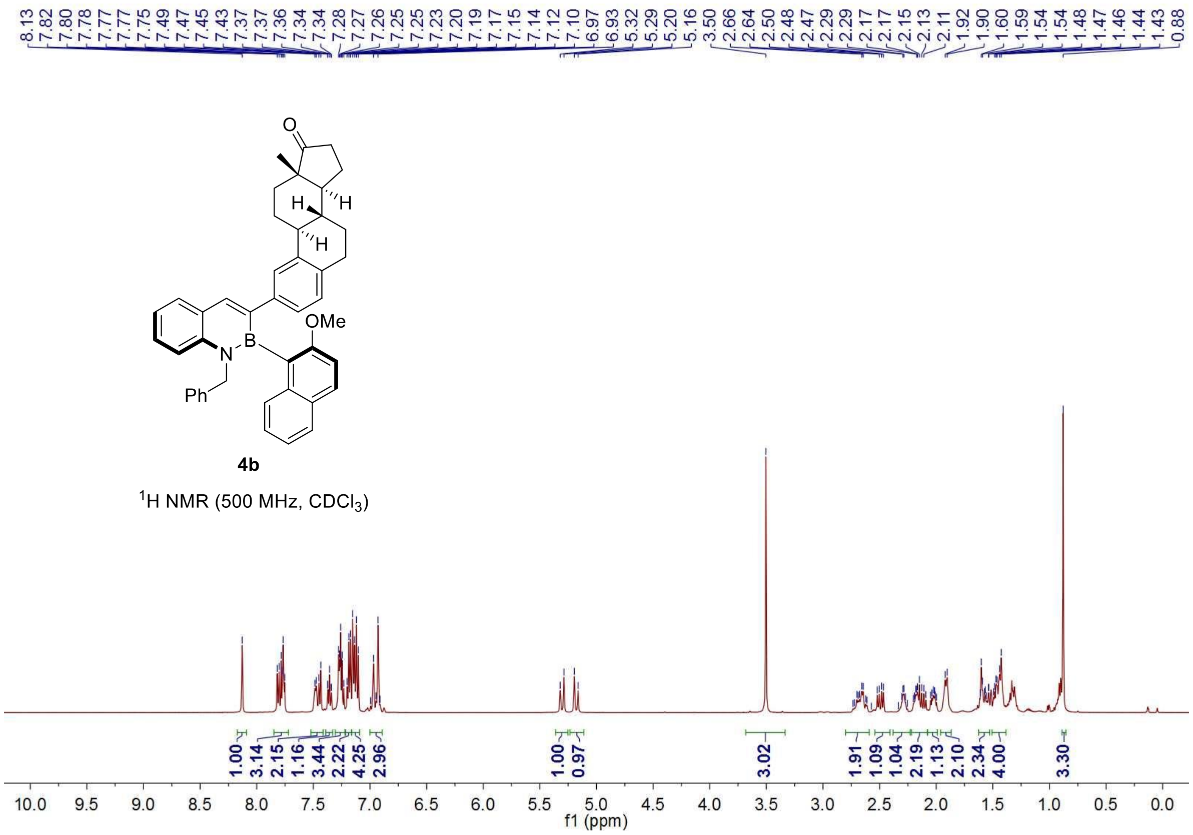

Supplementary Figure 223. ^{1}H NMR spectrum of 4b

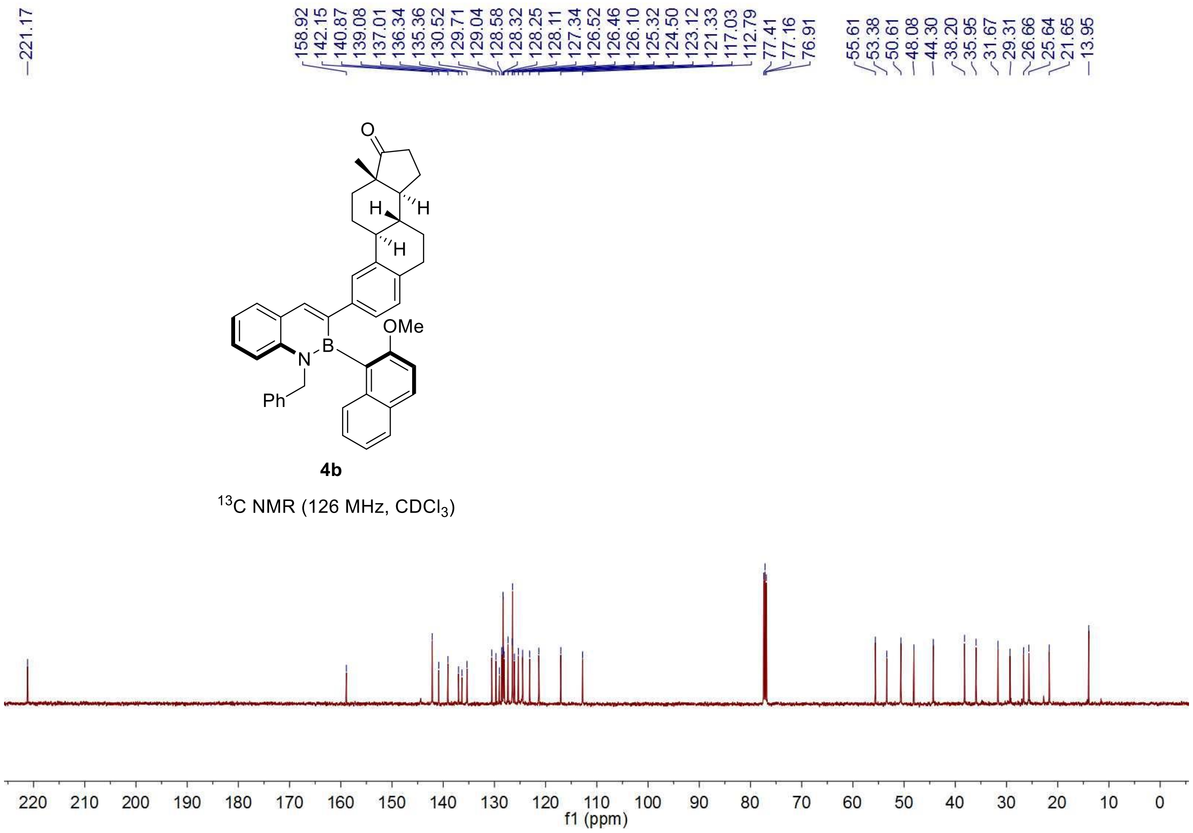

Supplementary Figure 224. ^{13}C NMR spectrum of 4b

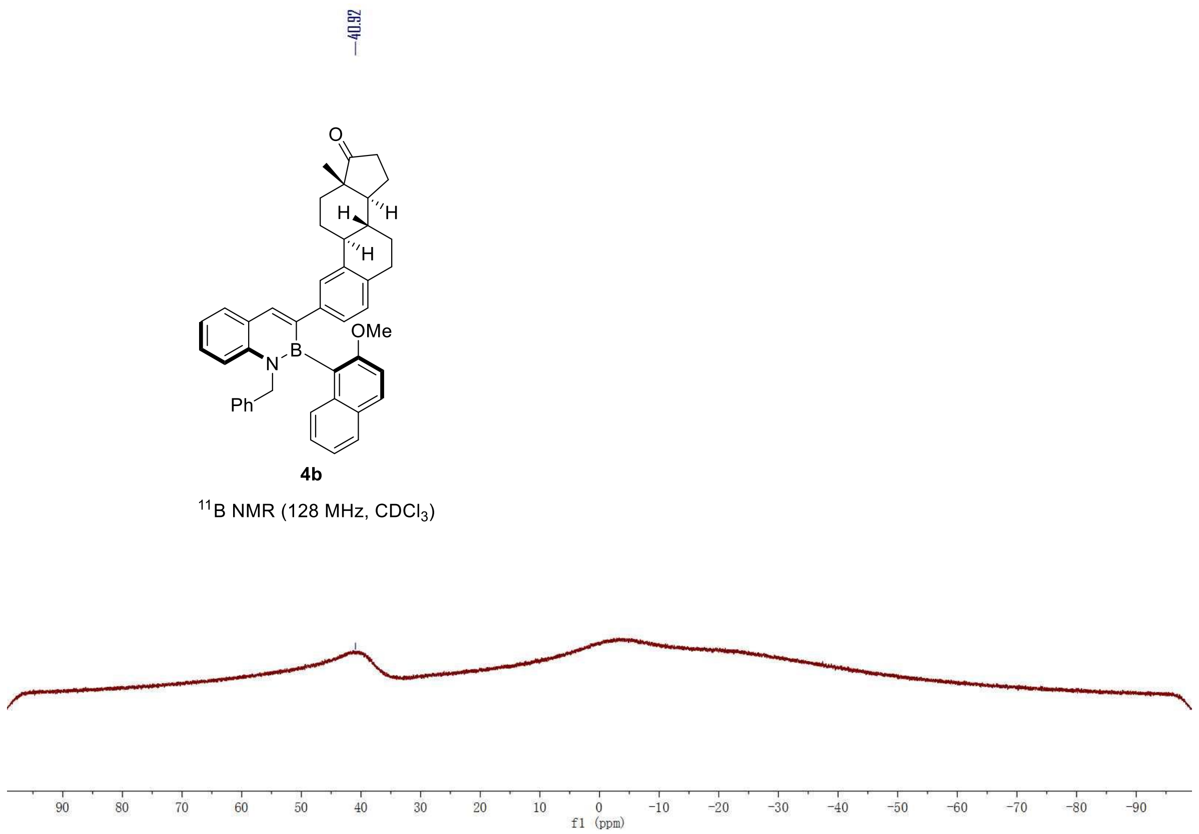

Supplementary Figure 225. ^{11}B NMR spectrum of 4b

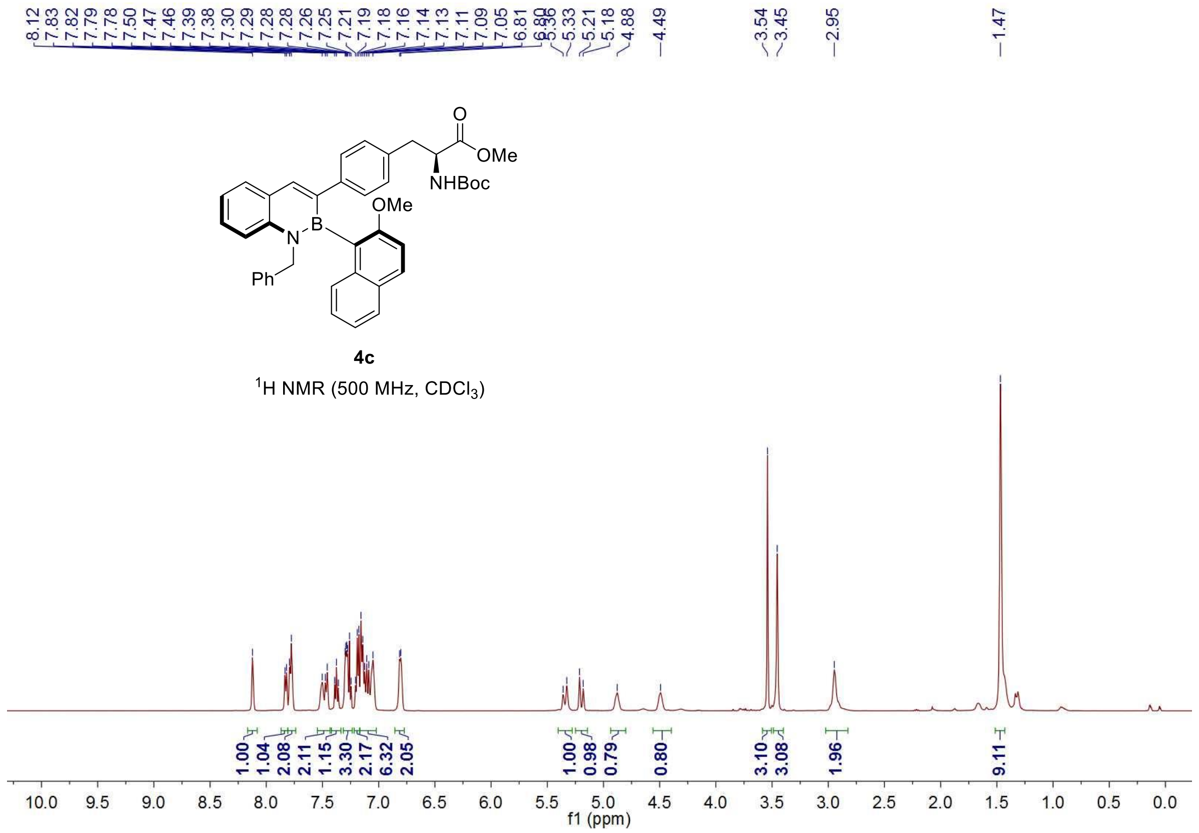

Supplementary Figure 226. ^{1}H NMR spectrum of 4c

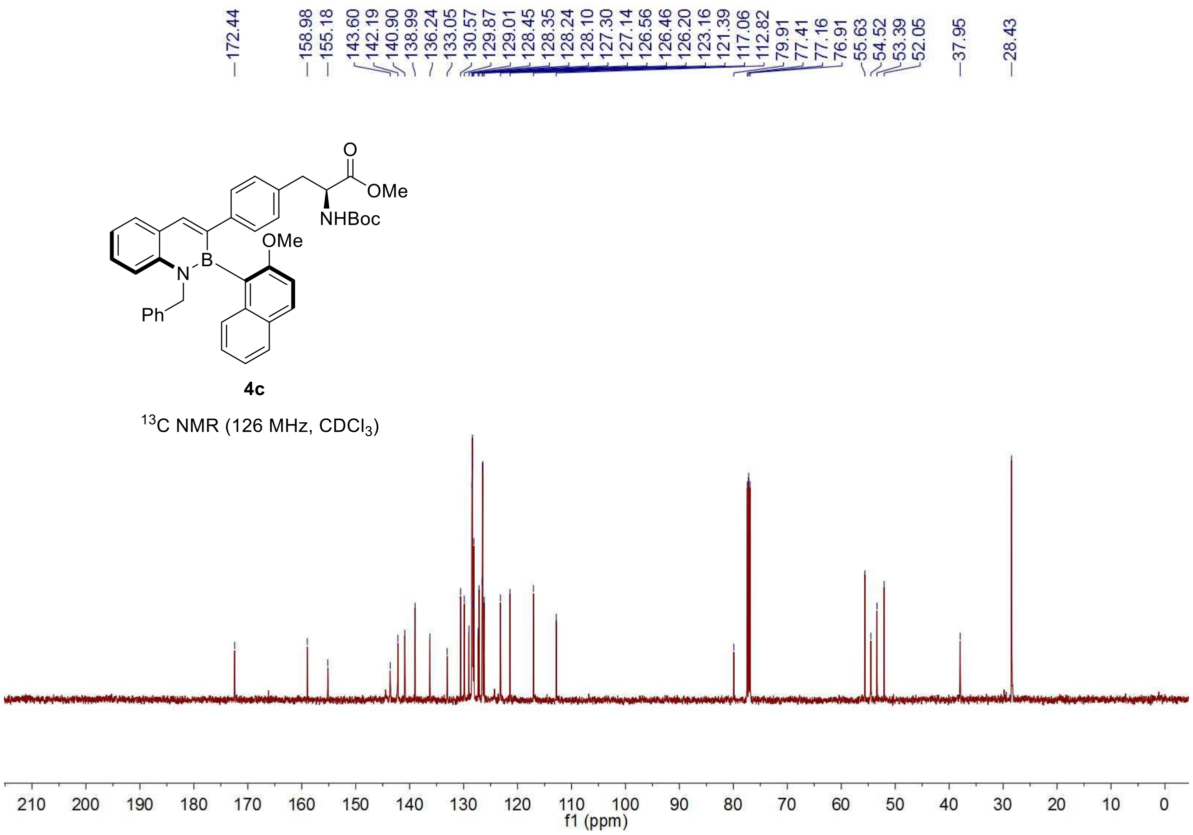

Supplementary Figure 227. ^{13}C NMR spectrum of 4c

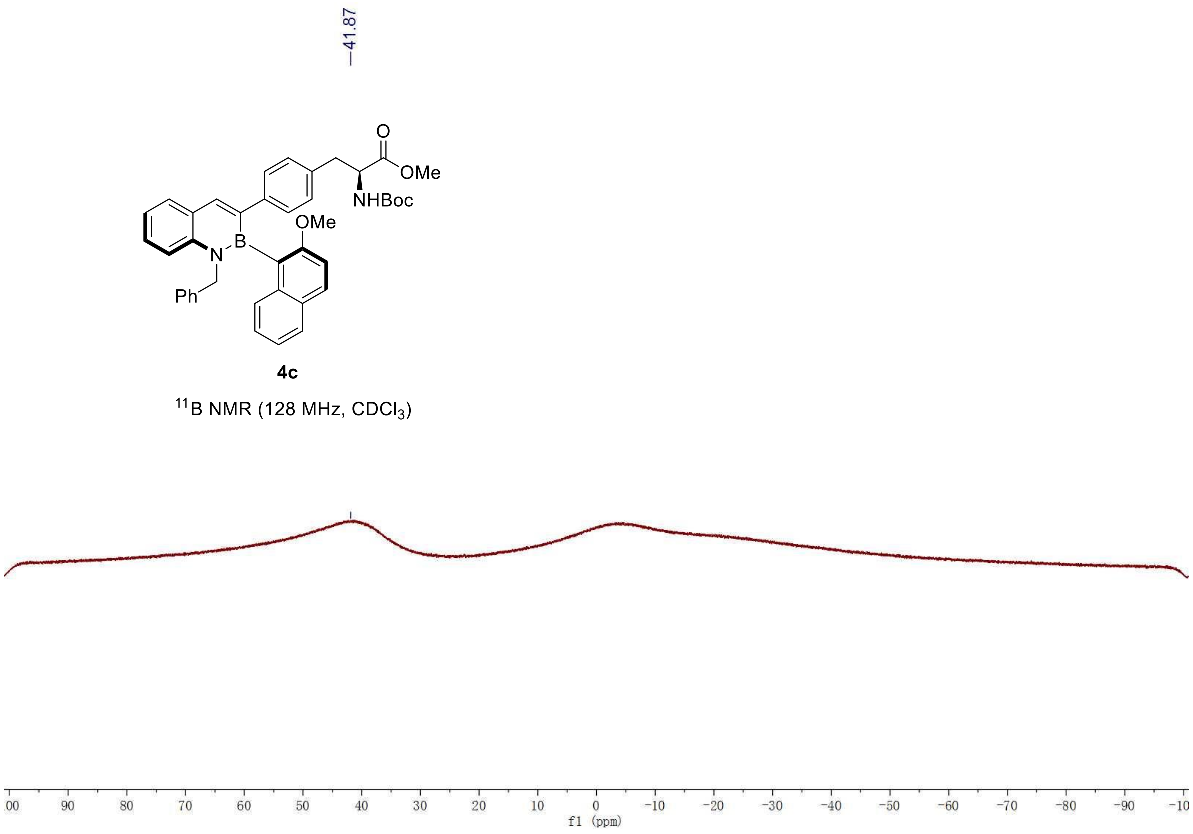

Supplementary Figure 228. ^{11}B NMR spectrum of 4c

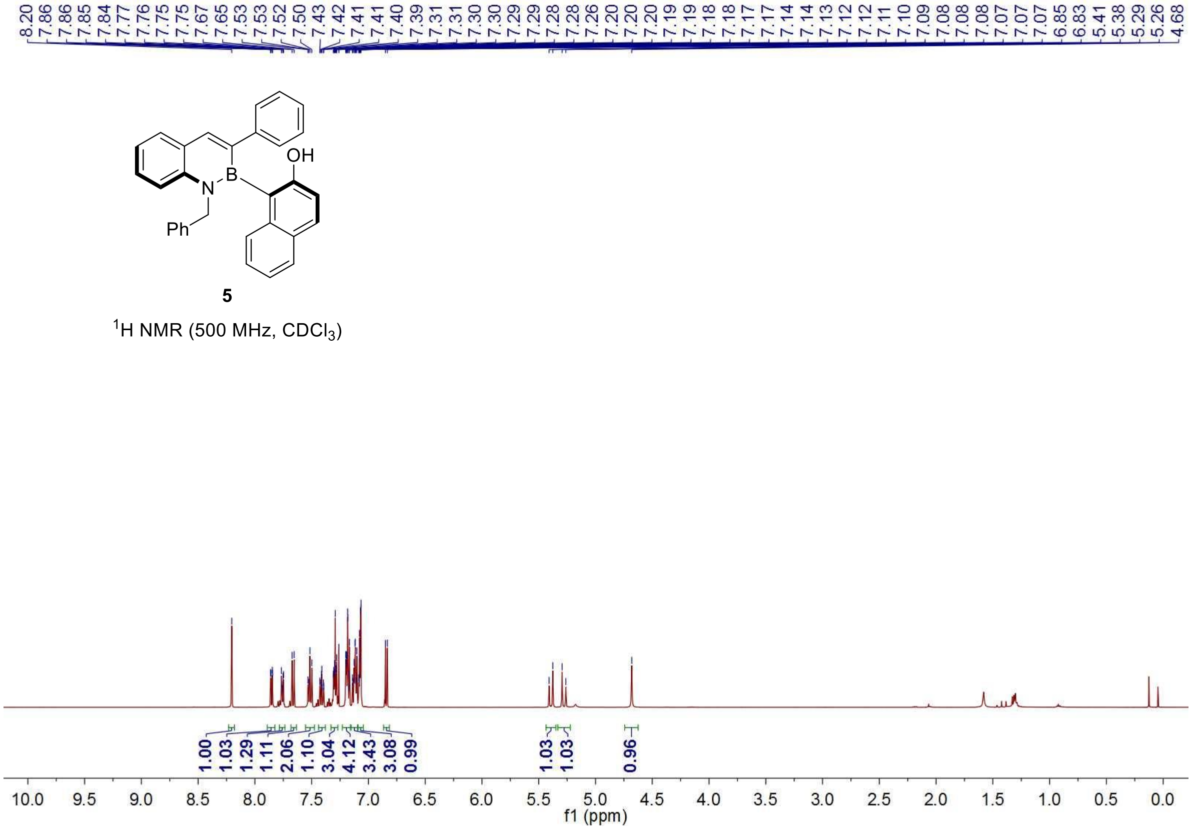

Supplementary Figure 229. ^{1}H NMR spectrum of 5

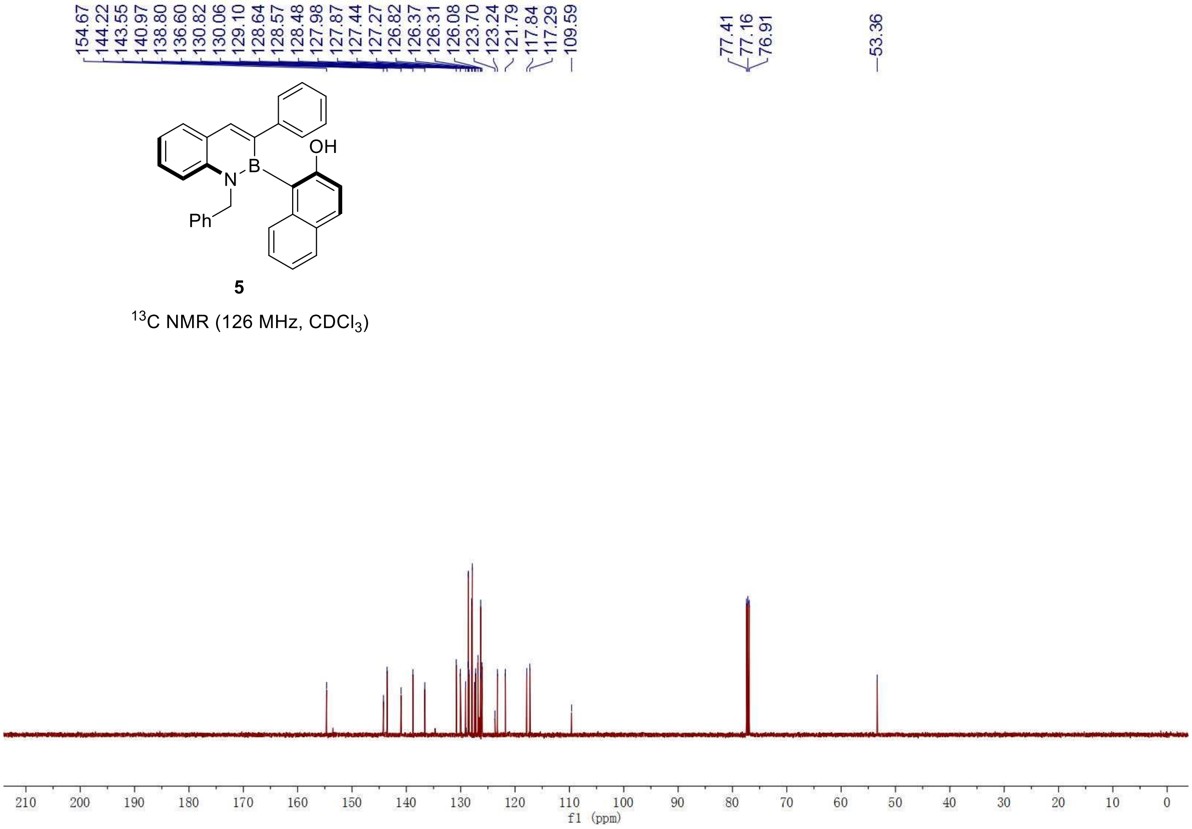

Supplementary Figure 230. ^{13}C NMR spectrum of 5

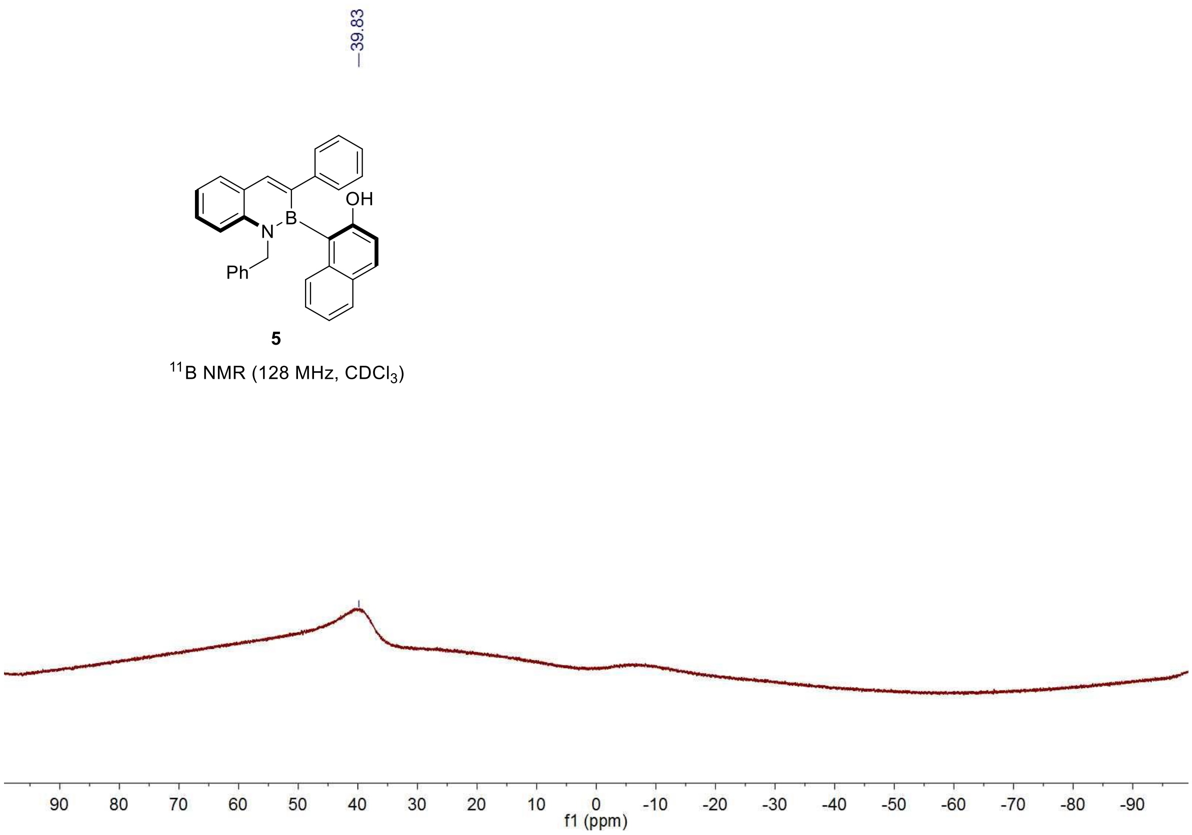

Supplementary Figure 231. ^{11}B NMR spectrum of 5

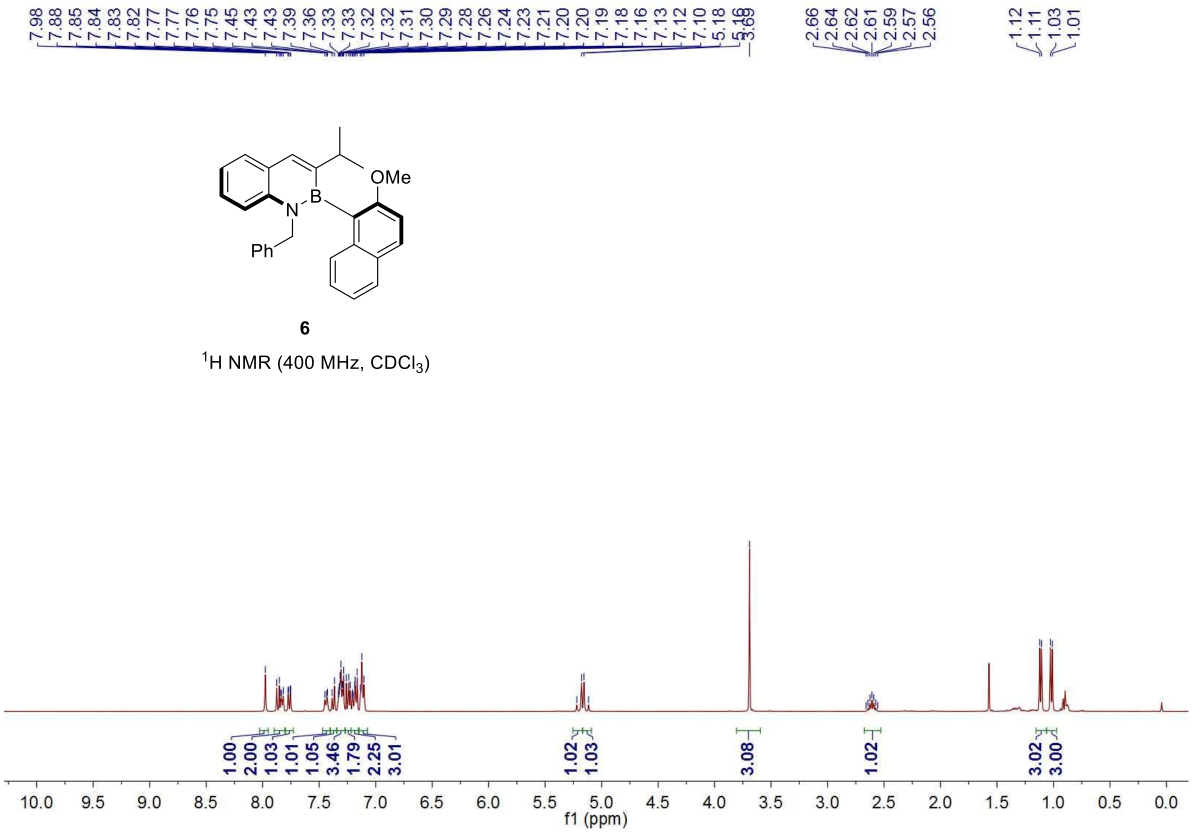

Supplementary Figure 232. ^{1}H NMR spectrum of 6

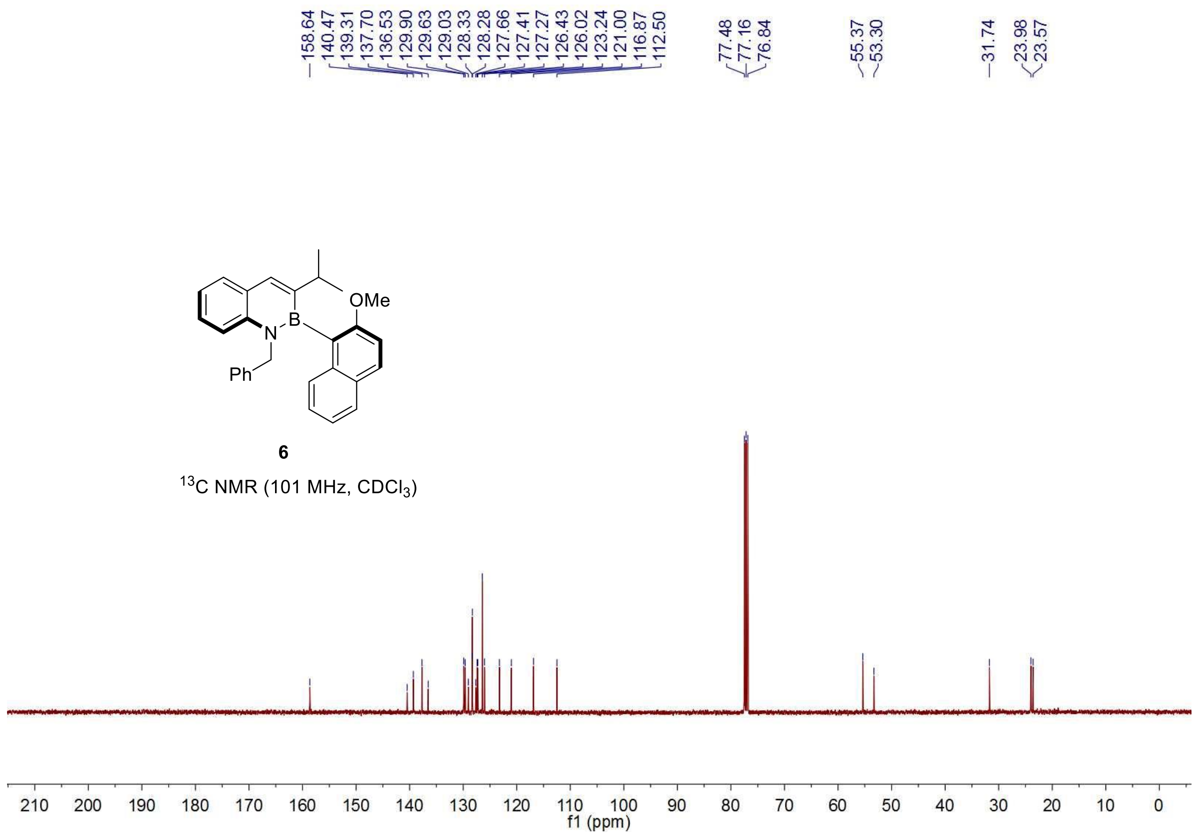

Supplementary Figure 233. ^{13}C NMR spectrum of 6

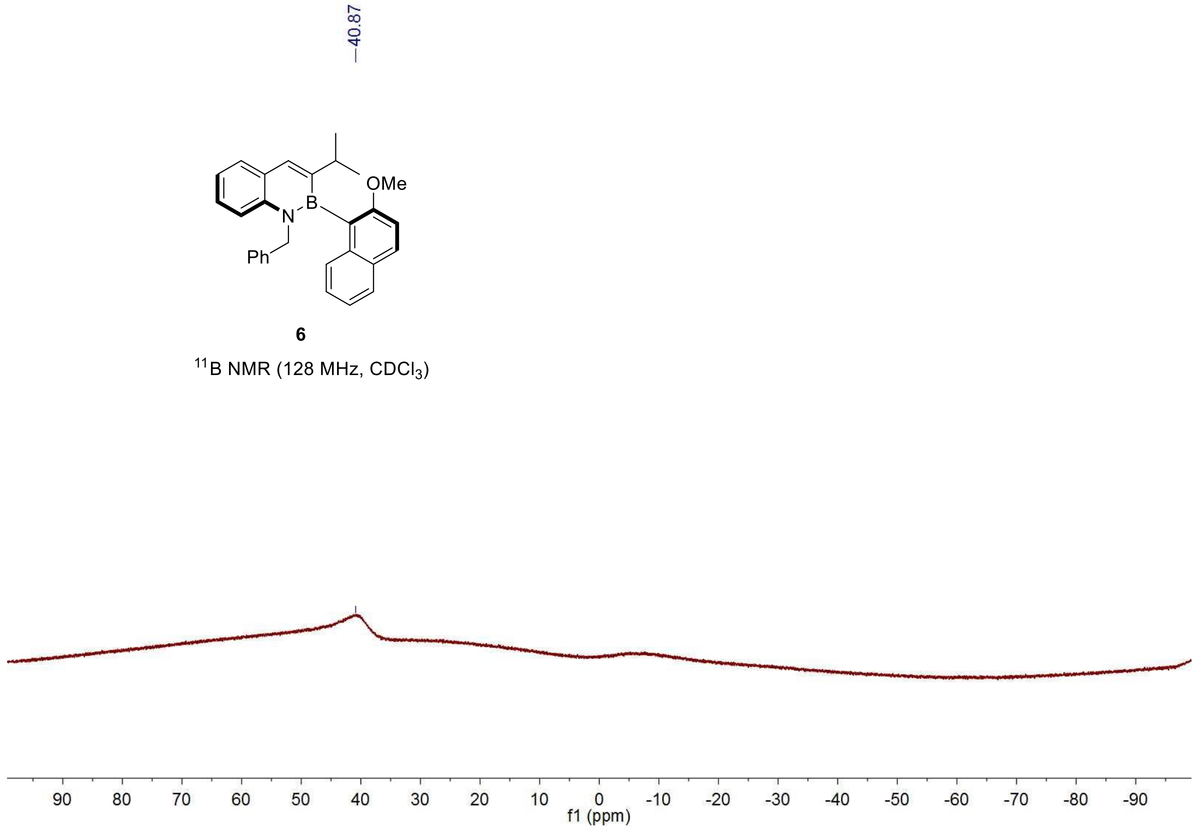

Supplementary Figure 234. ^{11}B NMR spectrum of 6

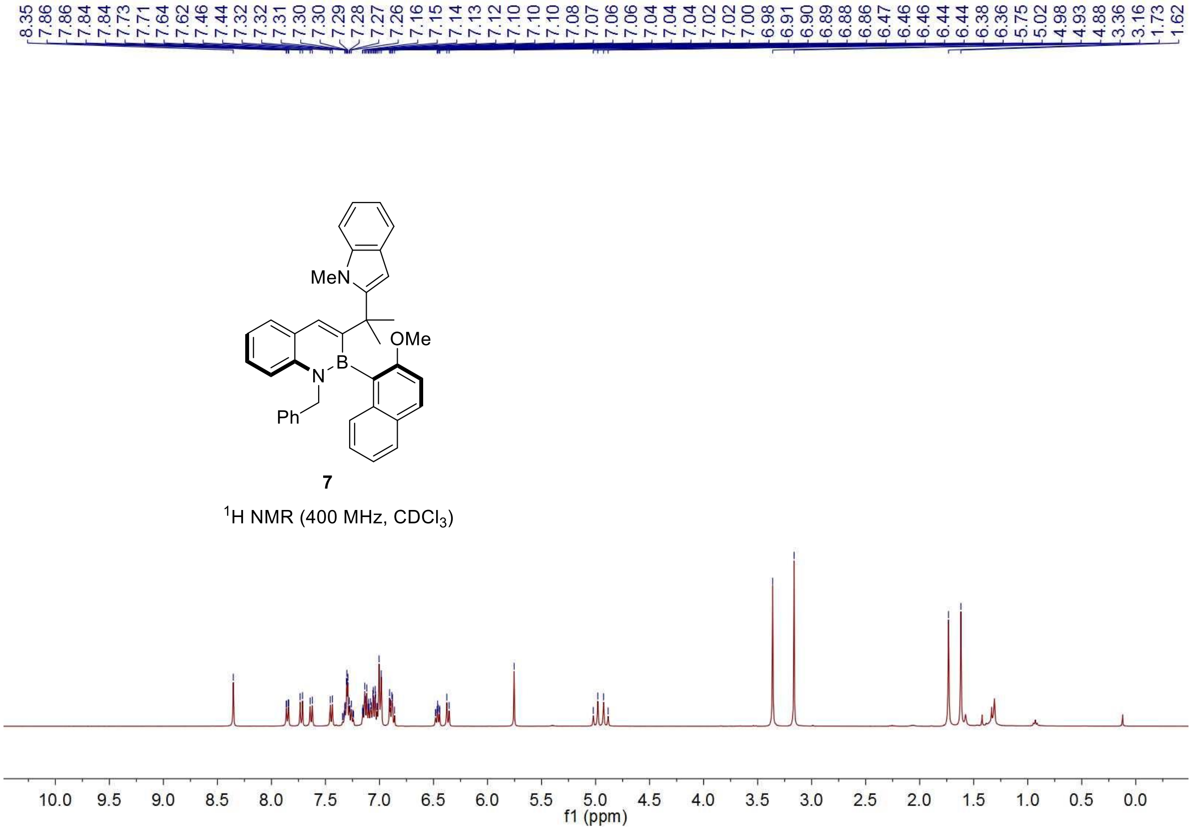

Supplementary Figure 235. ^{1}H NMR spectrum of 7

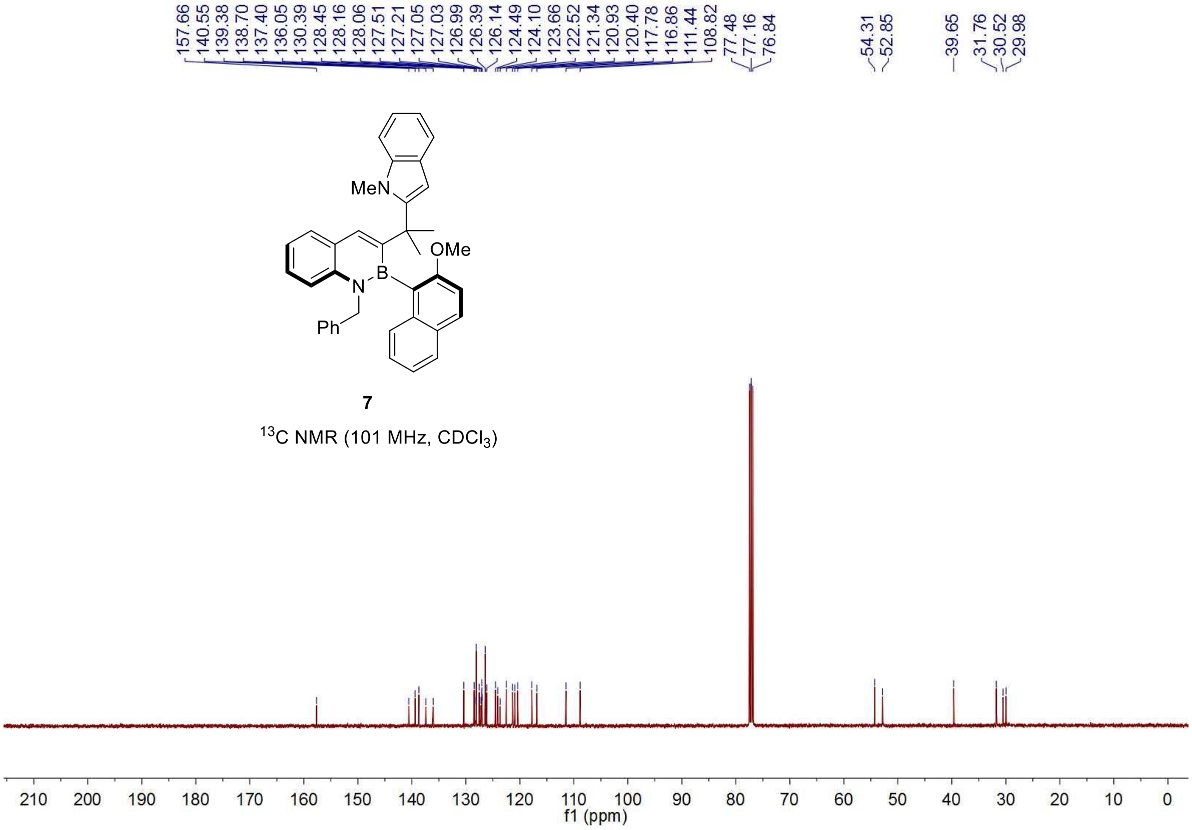

Supplementary Figure 236. ^{13}C NMR spectrum of 7

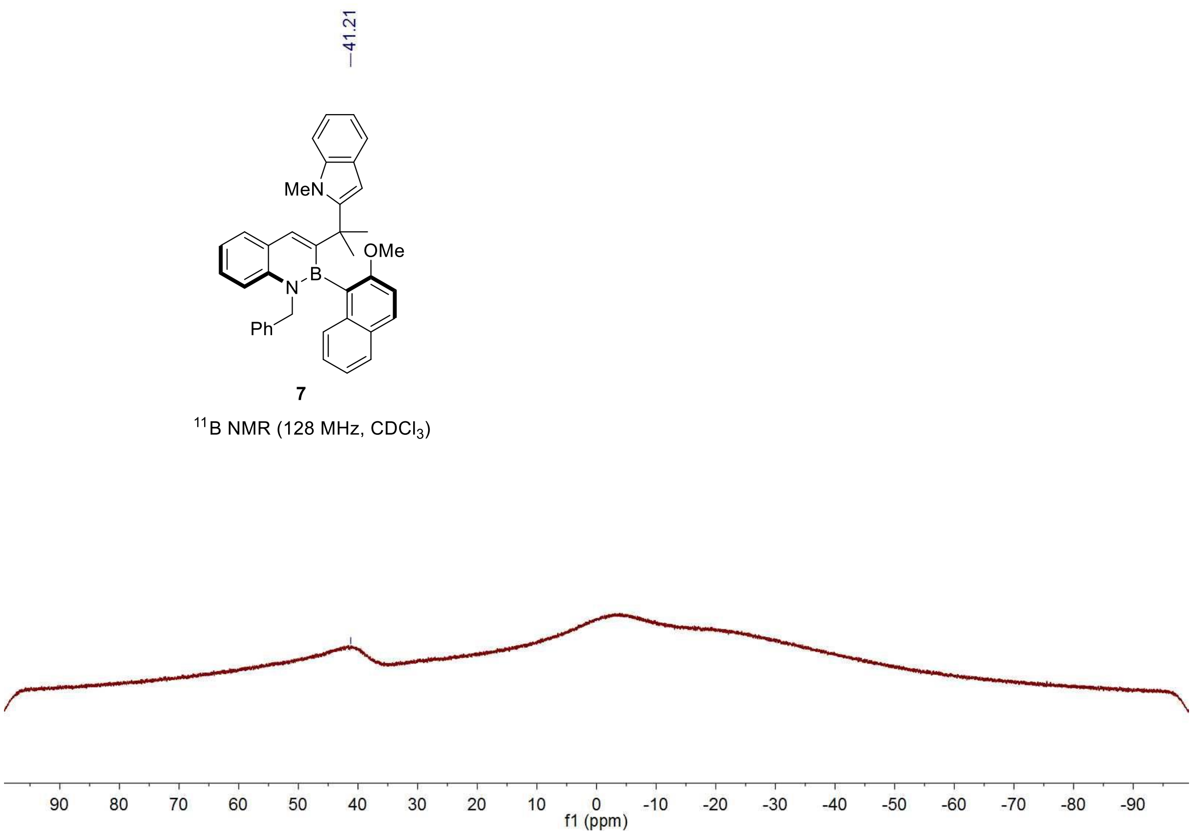

Supplementary Figure 237. ^{11}B NMR spectrum of 7

4 HPLC Spectra

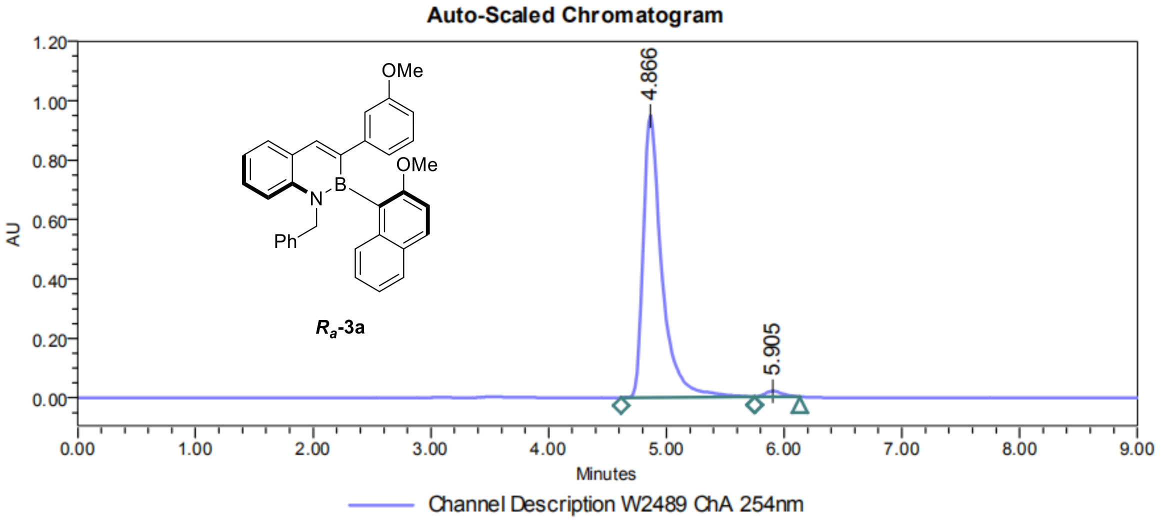

Peak Results

	Retention Time (min)	Int Type	Width (sec)	Area (µV*sec)	Height (µV)	% Area
1	4.866	VV	68.000	10517643	948505	98.11
2	5.905	Vb	23.000	202930	20094	1.89

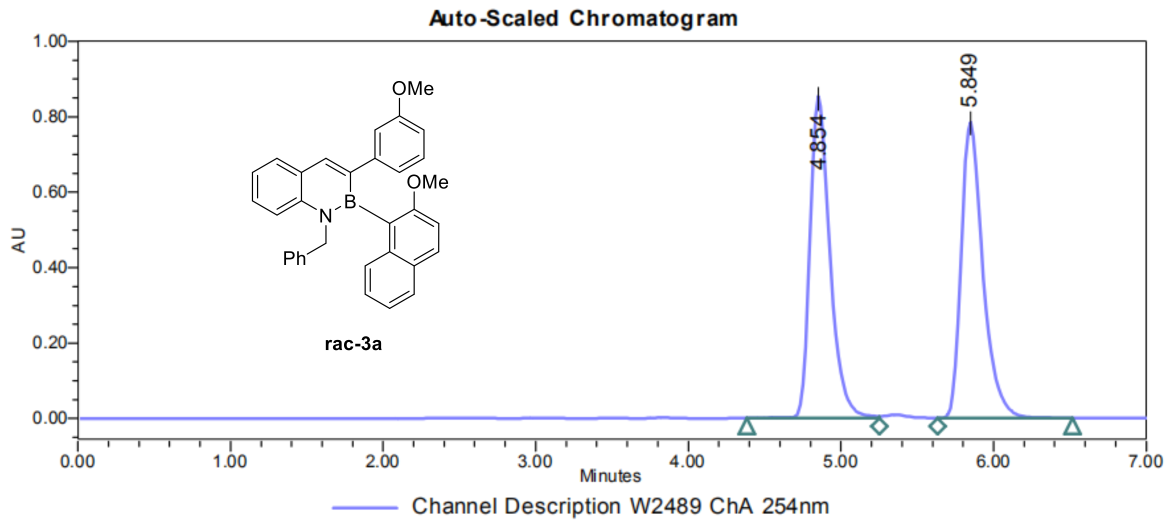

Peak Results

	Retention Time (min)	Int Type	Width (sec)	Area (µV*sec)	Height (µV)	% Area
1	4.854	BV	52.000	7782894	852712	50.17
2	5.849	VB	53.000	7728704	783343	49.83

Supplementary Figure 238. Chiral HPLC analysis of 3a

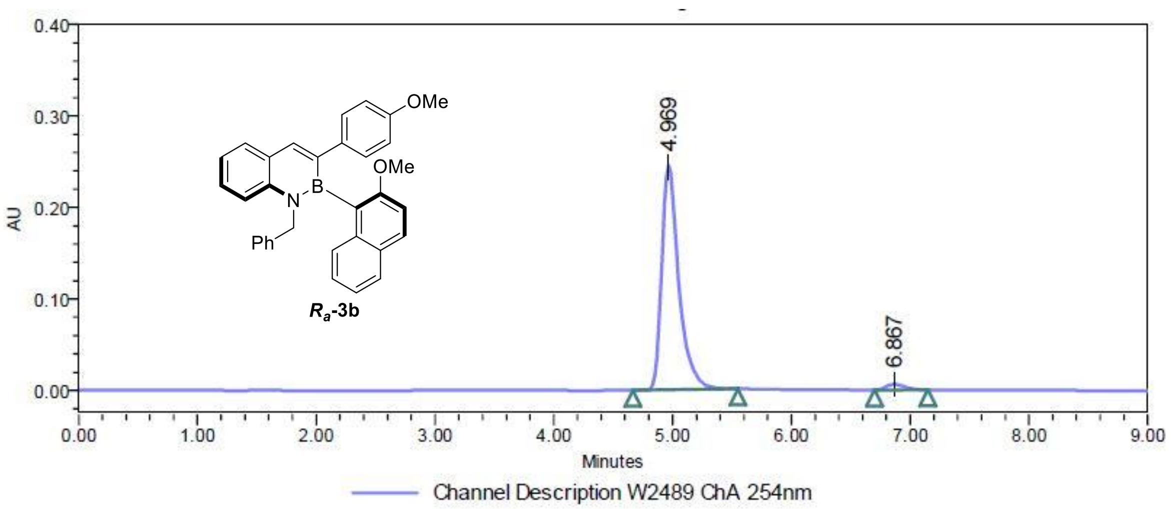

Peak Results

	Retention Time (min)	Int Type	Width (sec)	Area (µV*sec)	Height (µV)	% Area
1	4.969	BB	53.000	2445413	244739	97.22
2	6.867	bb	27.000	69896	6483	2.78

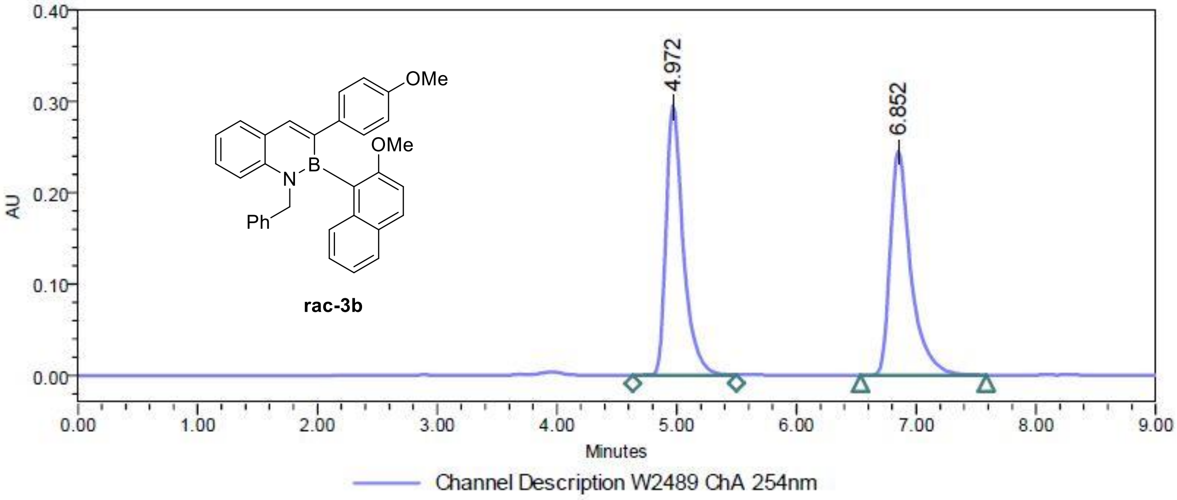

Peak Results

	Retention Time (min)	Int Type	Width (sec)	Area (µV*sec)	Height (µV)	% Area
1	4.972	VV	52.000	2946703	295328	50.05
2	6.852	BB	63.000	2941370	245320	49.95

Supplementary Figure 239. Chiral HPLC analysis of 3b

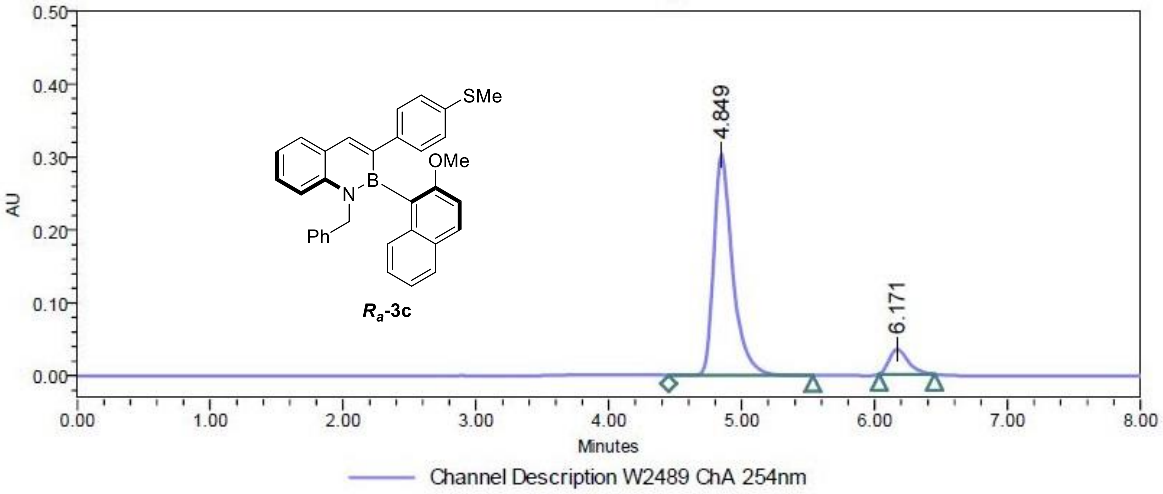

***R*$_a$-3c**

Peak Results

	Retention Time (min)	Int Type	Width (sec)	Area (µV*sec)	Height (µV)	% Area
1	4.849	Vb	65.000	3017956	302885	89.76
2	6.171	bb	25.000	344447	34460	10.24

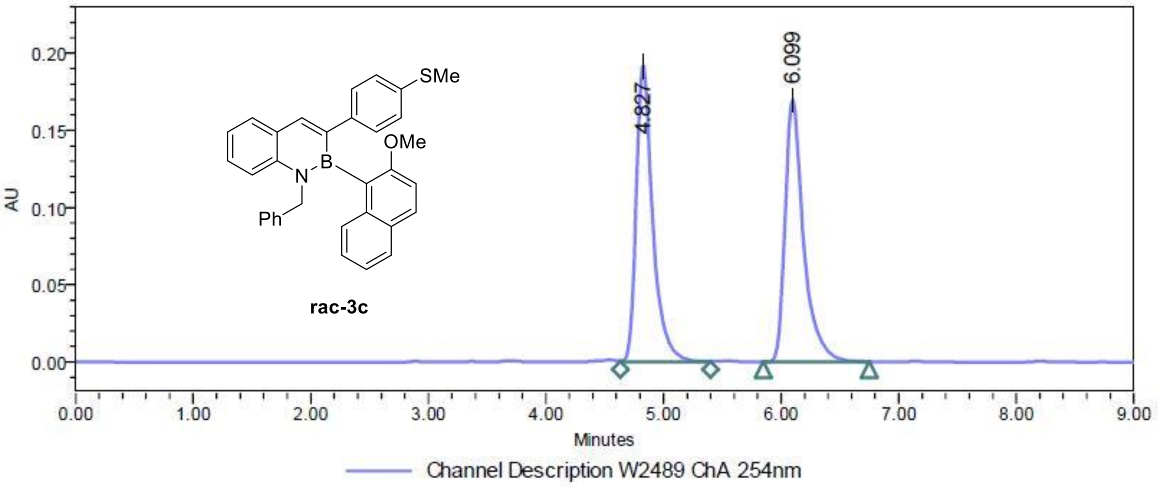

rac-3c

Peak Results

	Retention Time (min)	Int Type	Width (sec)	Area (µV*sec)	Height (µV)	% Area
1	4.827	VV	46.000	1882237	192285	50.42
2	6.099	BB	54.000	1850600	169670	49.58

Supplementary Figure 240. Chiral HPLC analysis of 3c

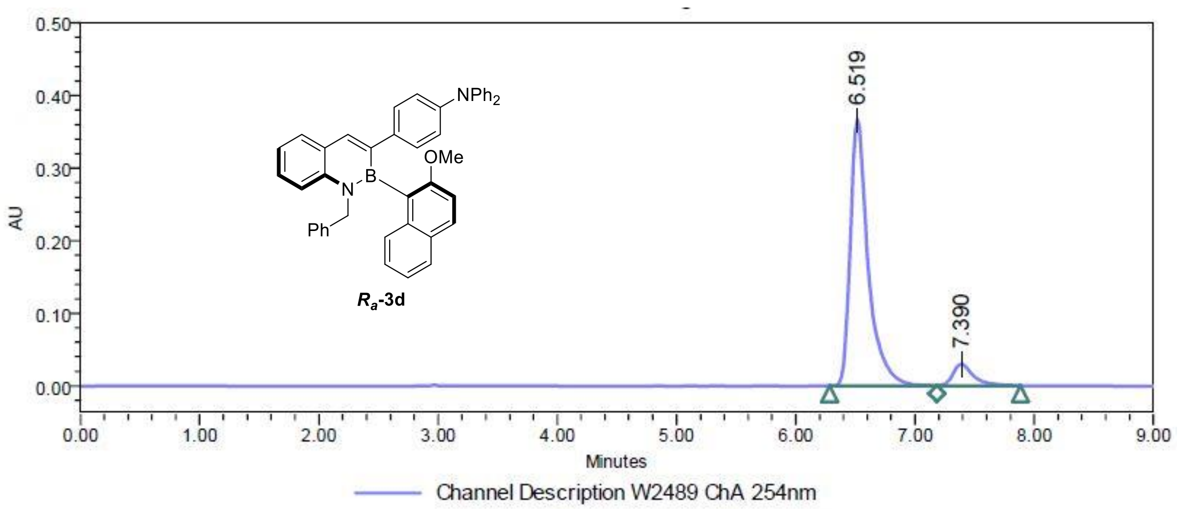

Peak Results

	Retention Time (min)	Int Type	Width (sec)	Area (µV*sec)	Height (µV)	% Area
1	6.519	BV	54.000	3824187	366351	91.45
2	7.390	Vb	42.000	357509	30197	8.55

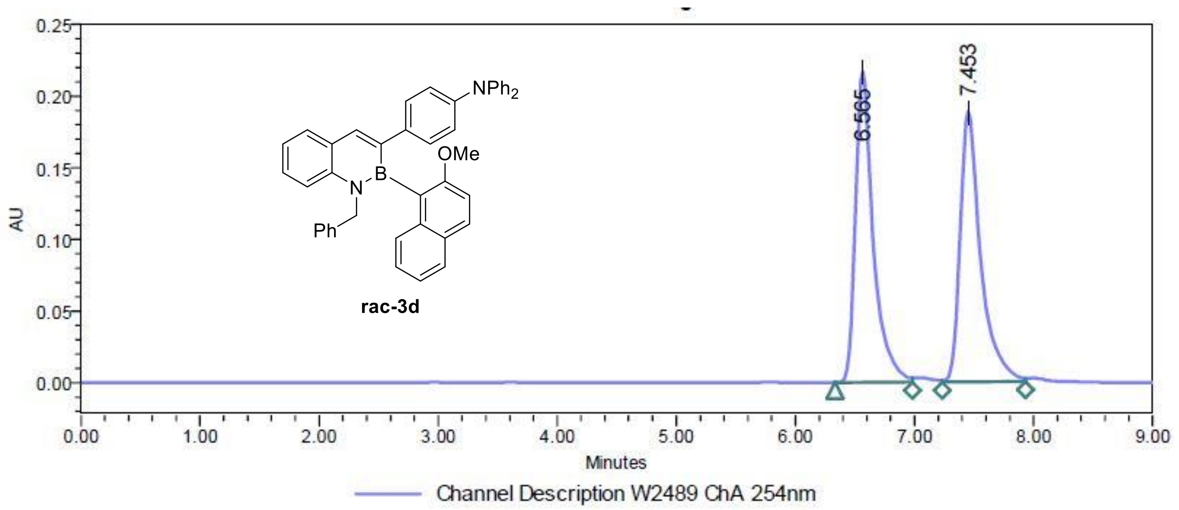

Peak Results

	Retention Time (min)	Int Type	Width (sec)	Area (µV*sec)	Height (µV)	% Area
1	6.565	BV	39.000	2261396	216403	49.95
2	7.453	VV	42.000	2266204	188605	50.05

Supplementary Figure 241. Chiral HPLC analysis of 3d

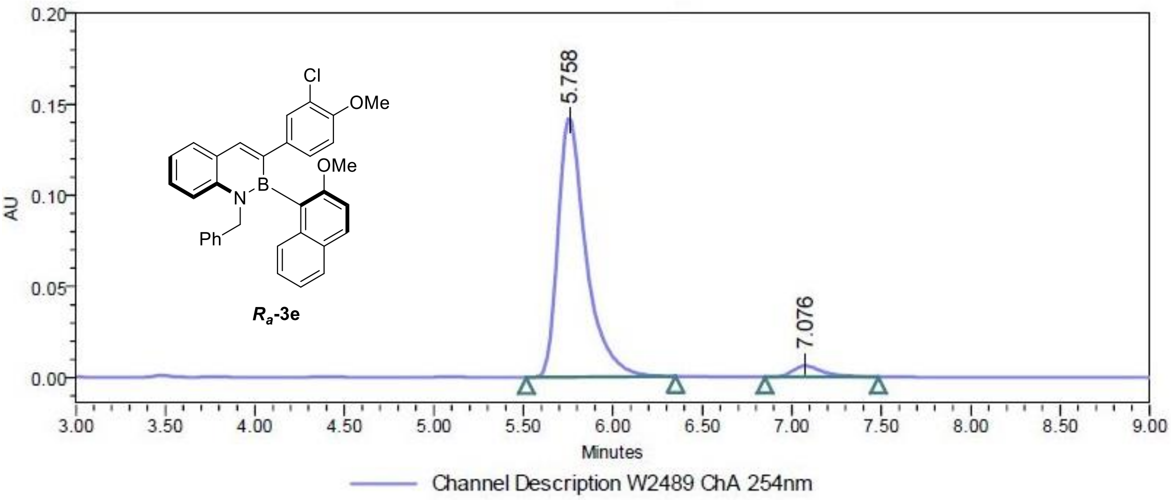

Peak Results

	Retention Time (min)	Int Type	Width (sec)	Area (µV*sec)	Height (µV)	% Area
1	5.758	BB	50.000	1543439	142443	95.47
2	7.076	BB	38.000	73177	6146	4.53

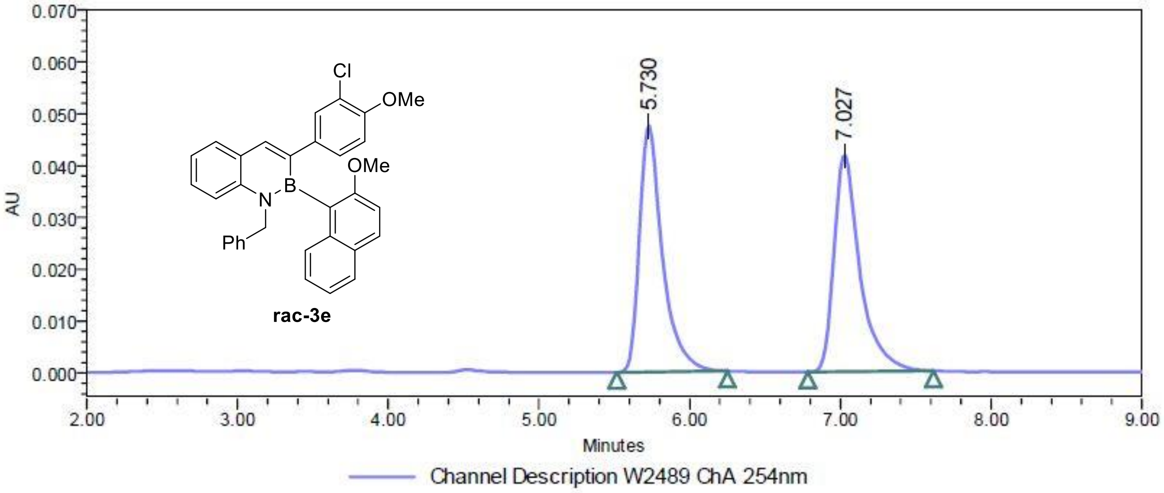

Peak Results

	Retention Time (min)	Int Type	Width (sec)	Area (µV*sec)	Height (µV)	% Area
1	5.730	BB	44.000	504148	47463	50.17
2	7.027	BB	50.000	500744	41742	49.83

Supplementary Figure 242. Chiral HPLC analysis of 3e

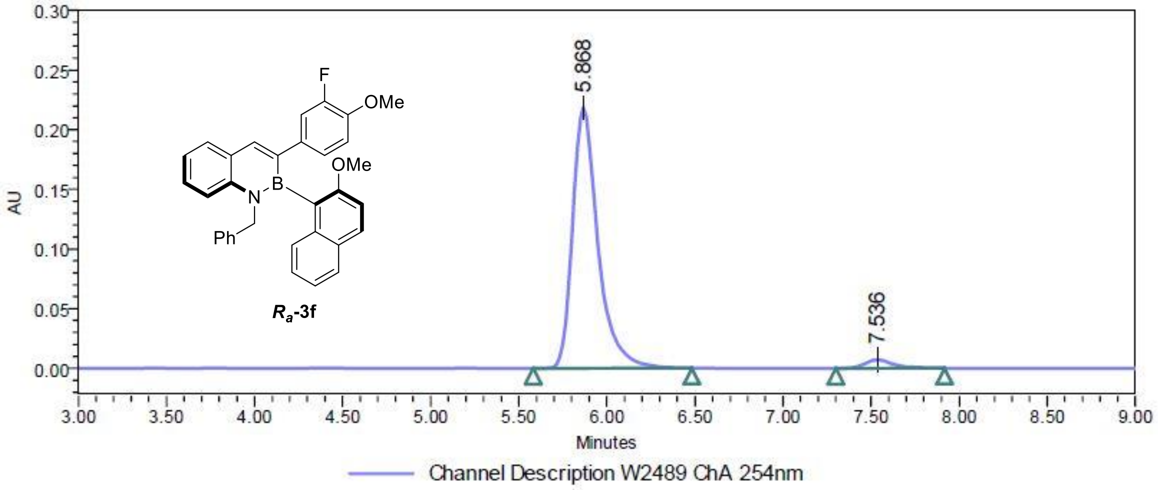

Peak Results

	Retention Time (min)	Int Type	Width (sec)	Area (μV*sec)	Height (μV)	% Area
1	5.868	BB	54.000	2180808	218134	96.51
2	7.536	BB	37.000	78924	7002	3.49

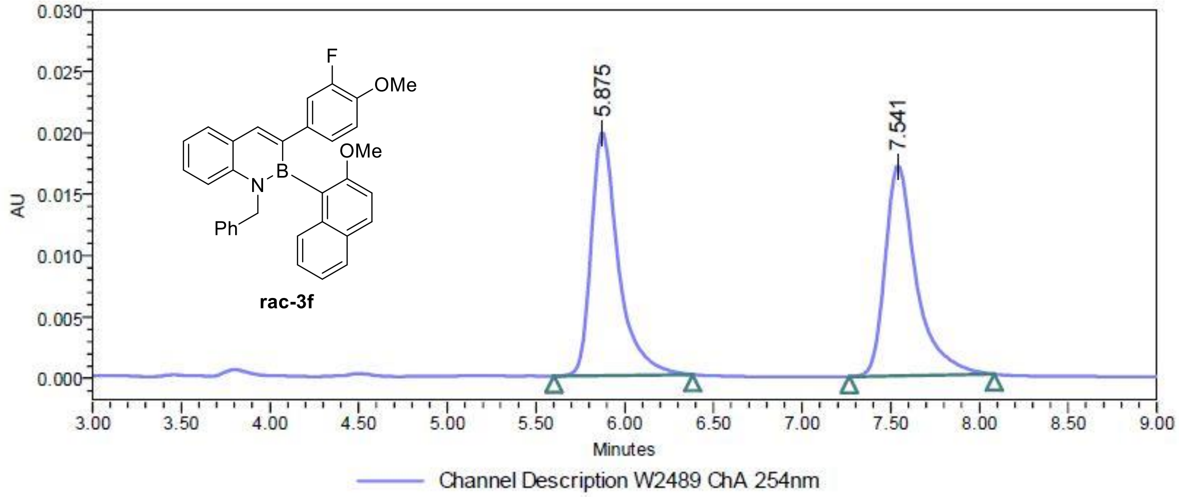

Peak Results

	Retention Time (min)	Int Type	Width (sec)	Area (μV*sec)	Height (μV)	% Area
1	5.875	BB	47.000	210799	19895	50.20
2	7.541	BB	49.000	209157	17147	49.80

Supplementary Figure 243. Chiral HPLC analysis of 3f

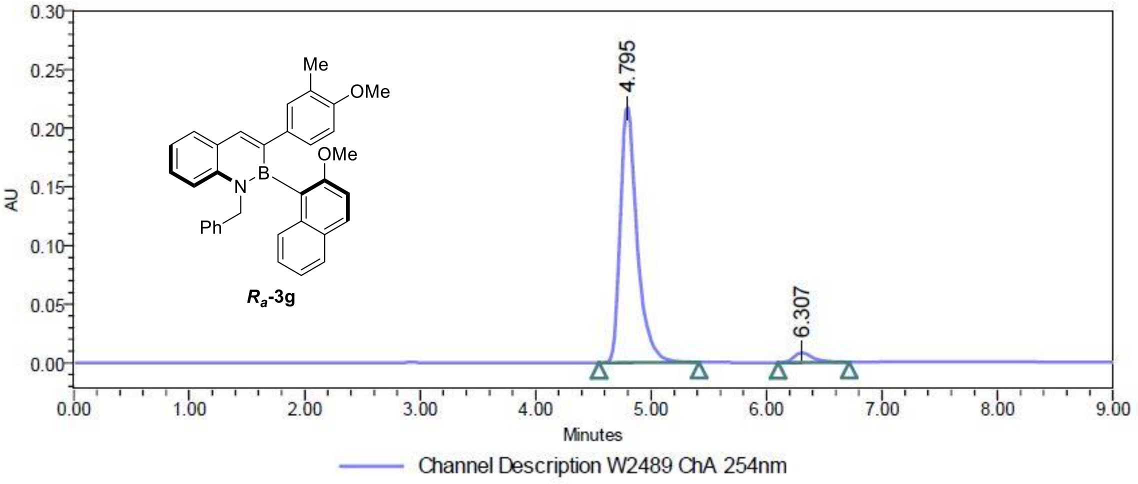

Peak Results

	Retention Time (min)	Int Type	Width (sec)	Area (μV*sec)	Height (μV)	% Area
1	4.795	BB	52.000	2203217	217784	95.96
2	6.307	BB	37.000	92732	8234	4.04

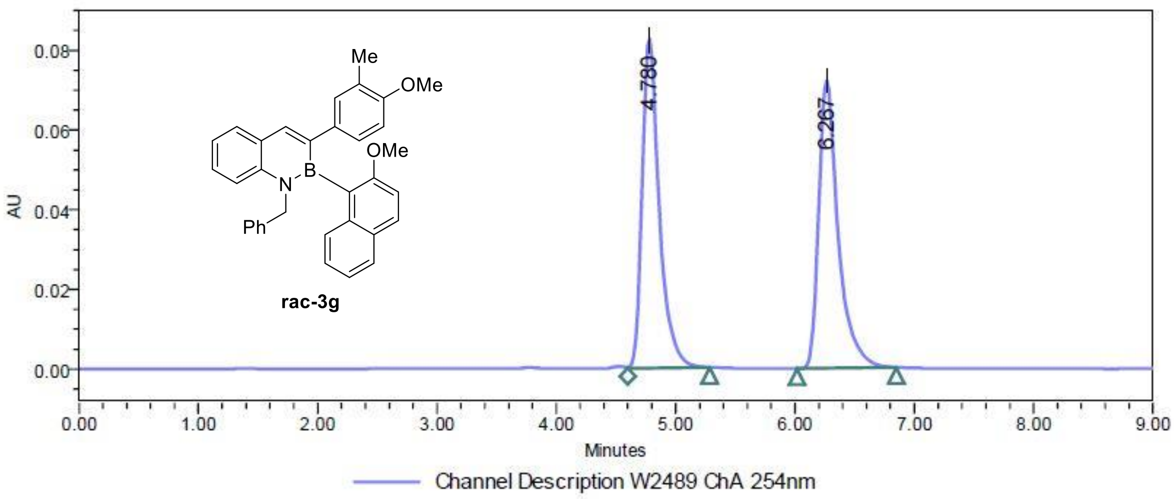

Peak Results

	Retention Time (min)	Int Type	Width (sec)	Area (μV*sec)	Height (μV)	% Area
1	4.780	VB	41.000	821663	82480	50.08
2	6.267	BB	50.000	818889	72113	49.92

Supplementary Figure 244. Chiral HPLC analysis of 3g

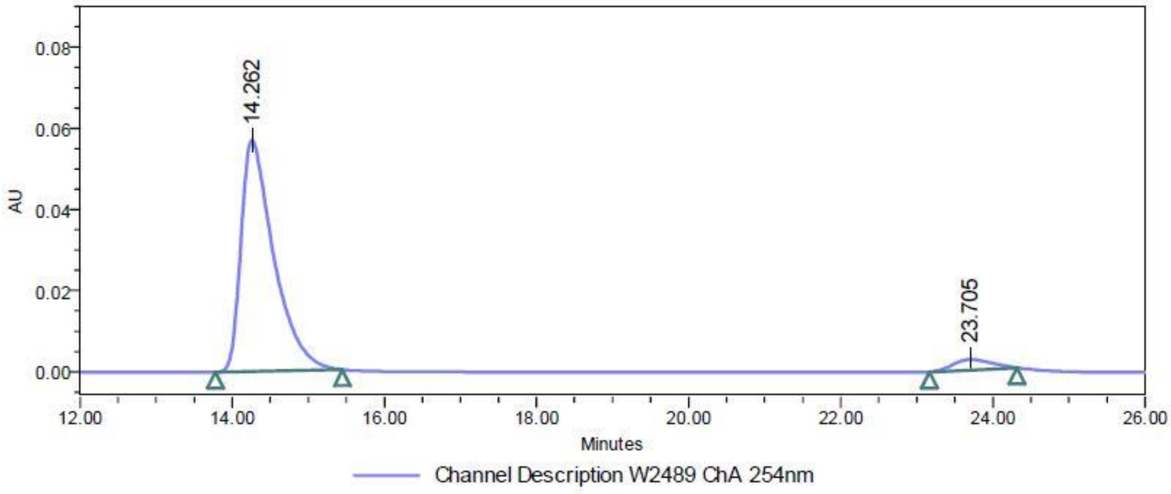

Peak Results

	Retention Time (min)	Int Type	Width (sec)	Area (µV*sec)	Height (µV)	% Area
1	14.262	BB	100.000	1703242	57011	95.00
2	23.705	BB	69.000	89677	2579	5.00

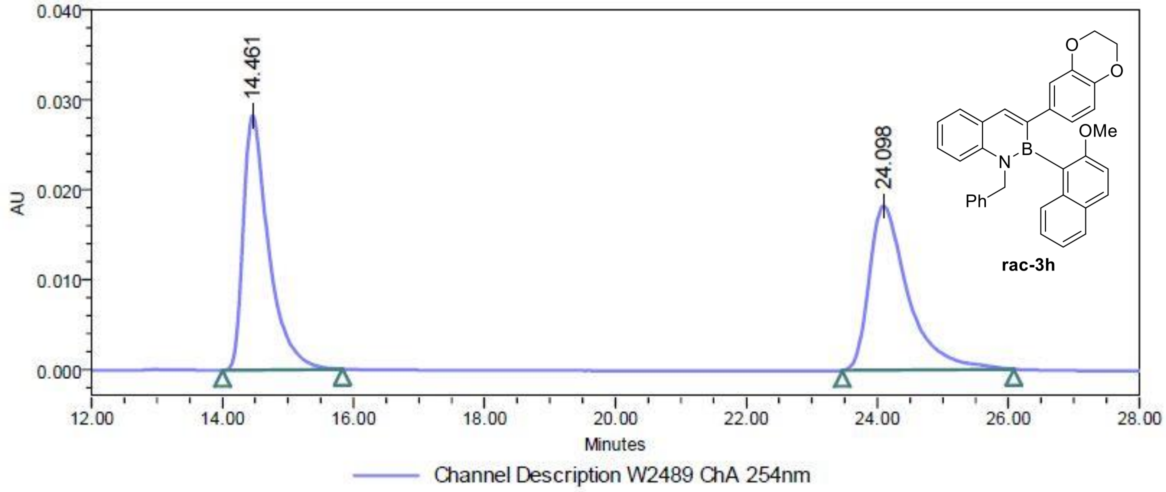

Peak Results

	Retention Time (min)	Int Type	Width (sec)	Area (µV*sec)	Height (µV)	% Area
1	14.461	Bb	110.000	762219	28315	50.17
2	24.098	Bb	157.000	757059	18257	49.83

Supplementary Figure 245. Chiral HPLC analysis of 3h

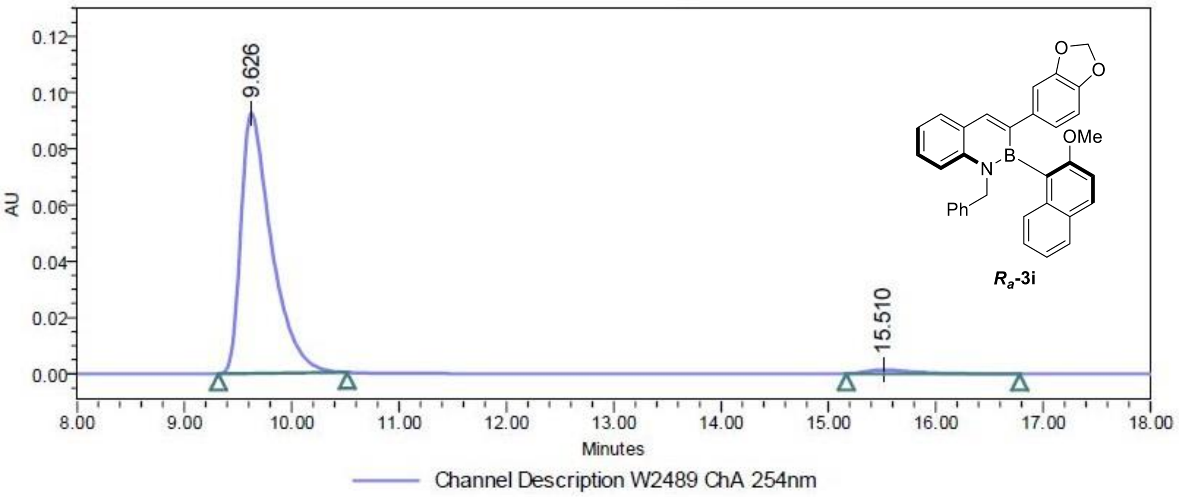

Peak Results

	Retention Time (min)	Int Type	Width (sec)	Area (µV*sec)	Height (µV)	% Area
1	9.626	BB	72.000	1830636	92740	97.78
2	15.510	BB	97.000	41471	1374	2.22

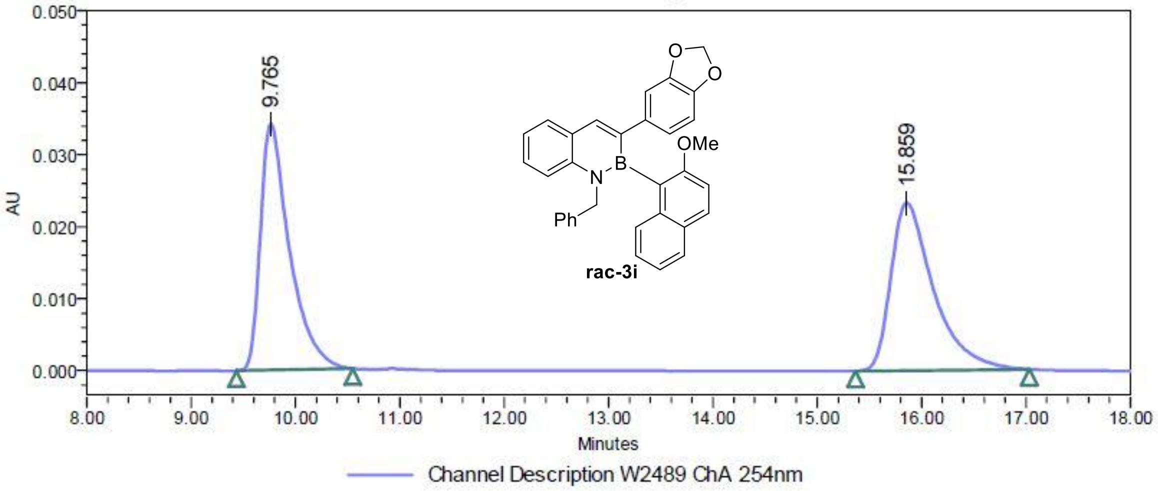

Peak Results

	Retention Time (min)	Int Type	Width (sec)	Area (µV*sec)	Height (µV)	% Area
1	9.765	BB	67.000	664162	34229	50.03
2	15.859	Bb	100.000	663265	23312	49.97

Supplementary Figure 246. Chiral HPLC analysis of 3i

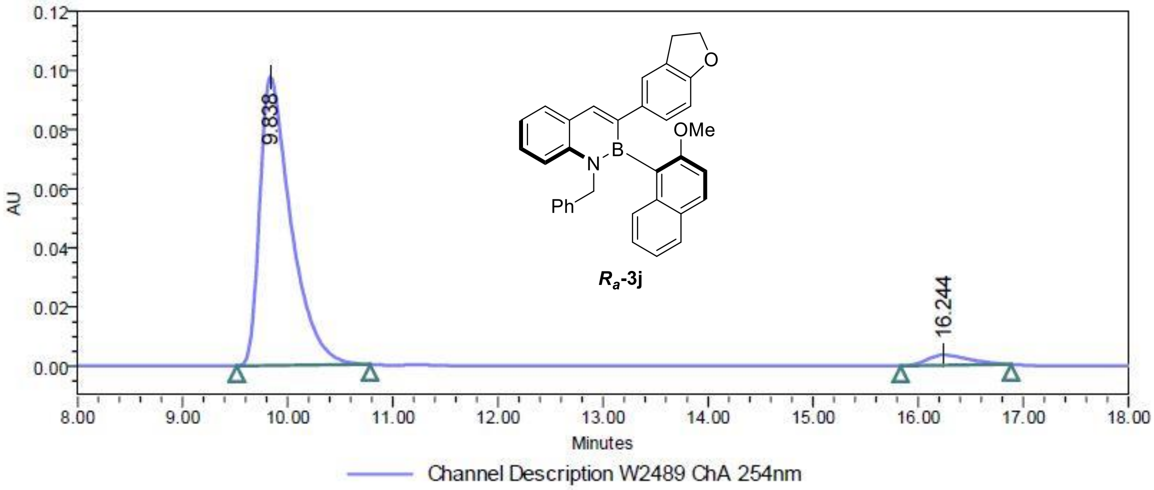

Peak Results

	Retention Time (min)	Int Type	Width (sec)	Area (µV*sec)	Height (µV)	% Area
1	9.838	BB	76.000	2011886	97736	95.30
2	16.244	BB	63.000	99171	3516	4.70

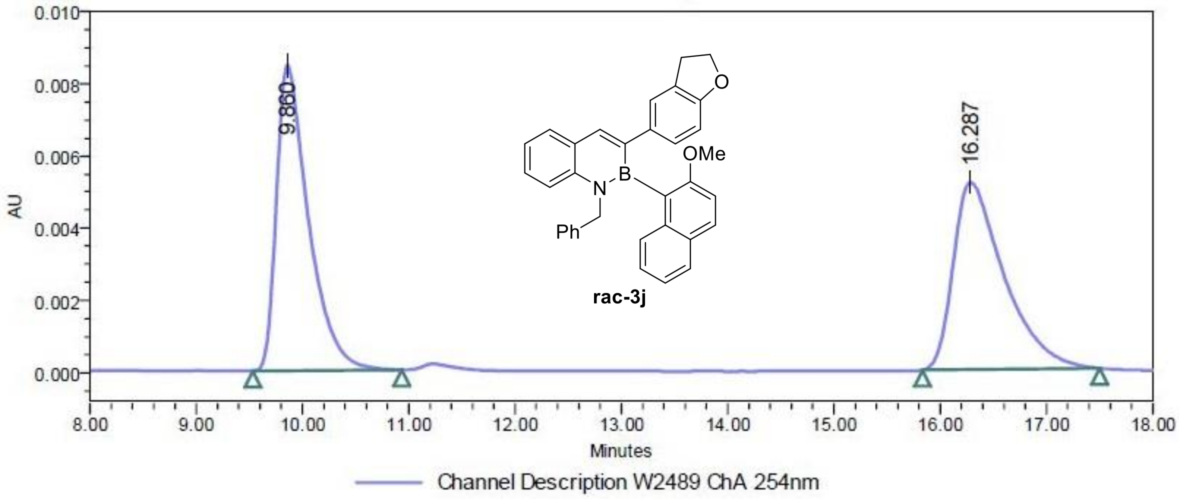

Peak Results

	Retention Time (min)	Int Type	Width (sec)	Area (µV*sec)	Height (µV)	% Area
1	9.860	Bb	84.000	176658	8479	50.66
2	16.287	Bb	100.000	172083	5207	49.34

Supplementary Figure 247. Chiral HPLC analysis of 3j

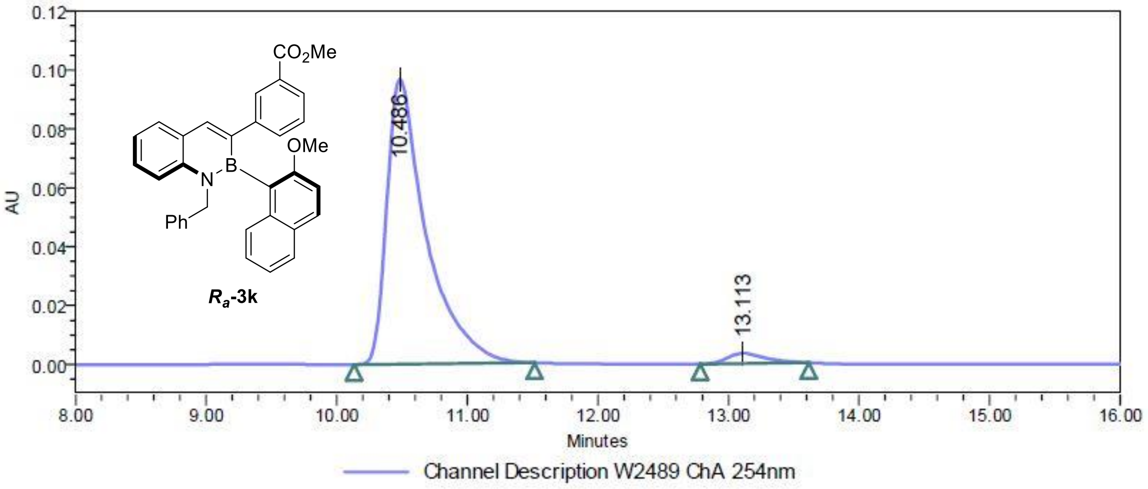

Peak Results

	Retention Time (min)	Int Type	Width (sec)	Area (µV*sec)	Height (µV)	% Area
1	10.486	BB	83.000	2024681	96888	96.62
2	13.113	BB	50.000	70894	3454	3.38

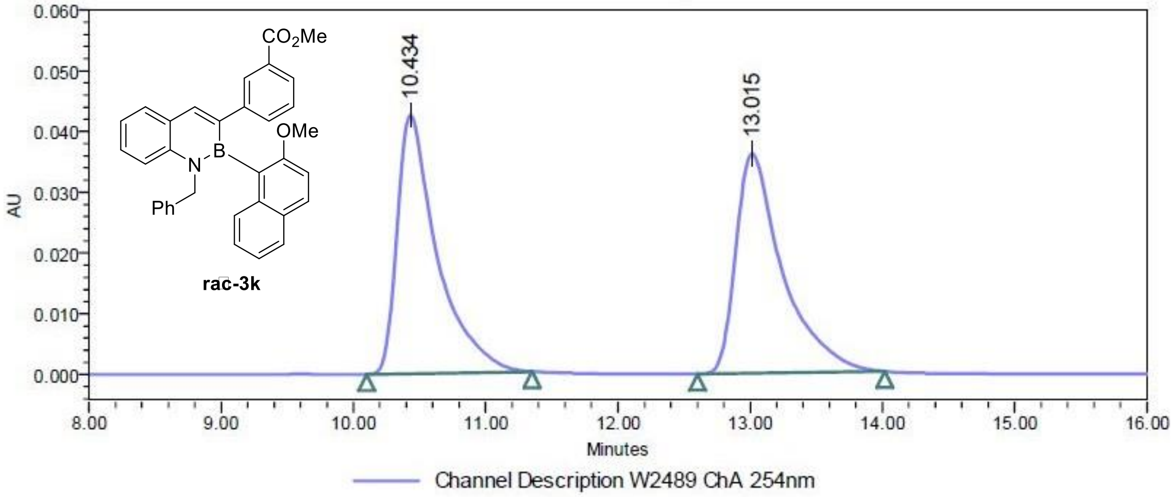

Peak Results

	Retention Time (min)	Int Type	Width (sec)	Area (µV*sec)	Height (µV)	% Area
1	10.434	BB	75.000	893115	42629	50.11
2	13.015	BB	85.000	889342	36207	49.89

Supplementary Figure 248. Chiral HPLC analysis of 3k

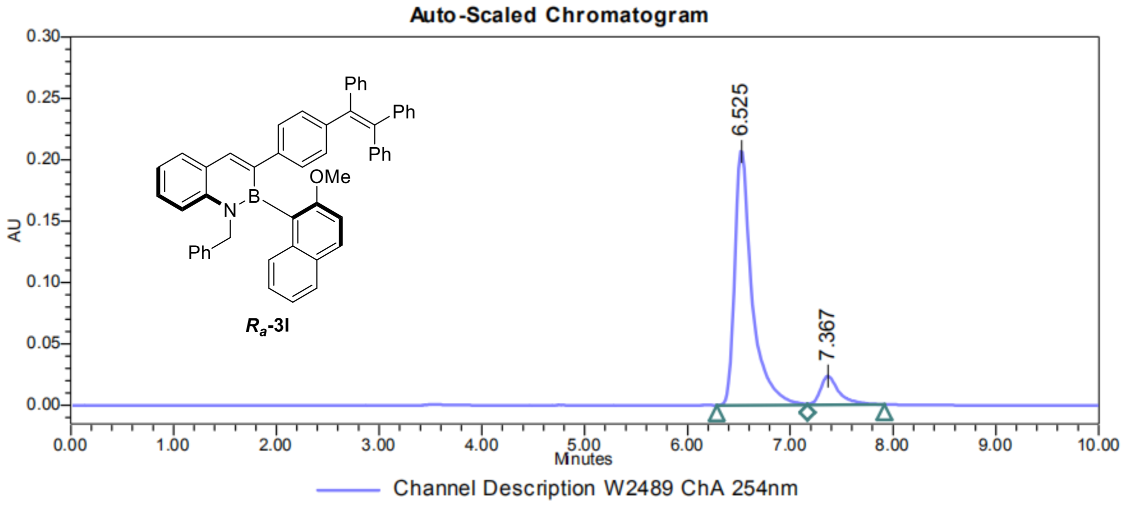

Peak Results

	Retention Time (min)	Int Type	Width (sec)	Area (µV*sec)	Height (µV)	% Area
1	6.525	BV	53.000	2428255	208066	89.31
2	7.367	VB	45.000	290510	23292	10.69

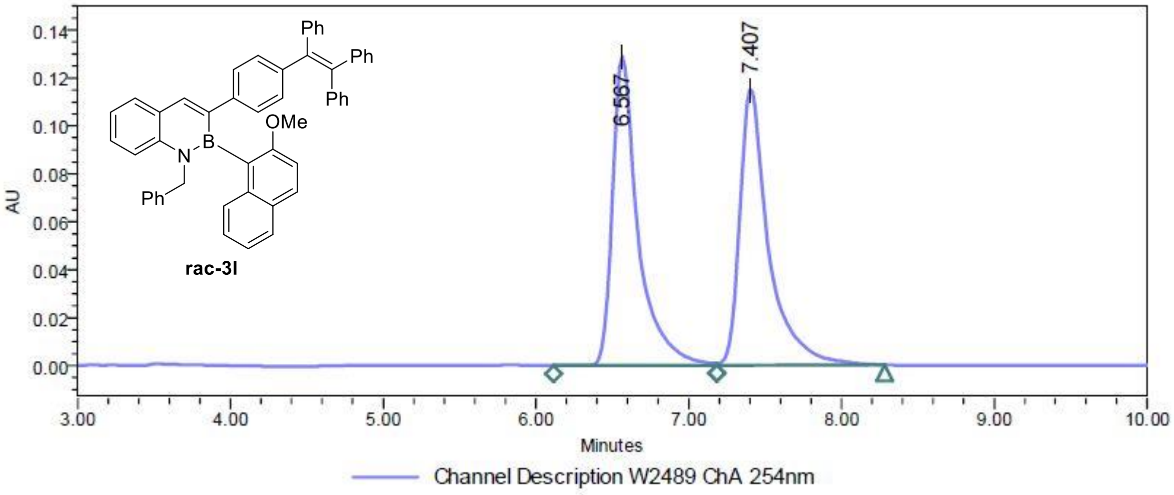

Peak Results

	Retention Time (min)	Int Type	Width (sec)	Area (µV*sec)	Height (µV)	% Area
1	6.567	VV	64.000	1500463	128618	49.87
2	7.407	VB	66.000	1508104	115370	50.13

Supplementary Figure 249. Chiral HPLC analysis of 3l

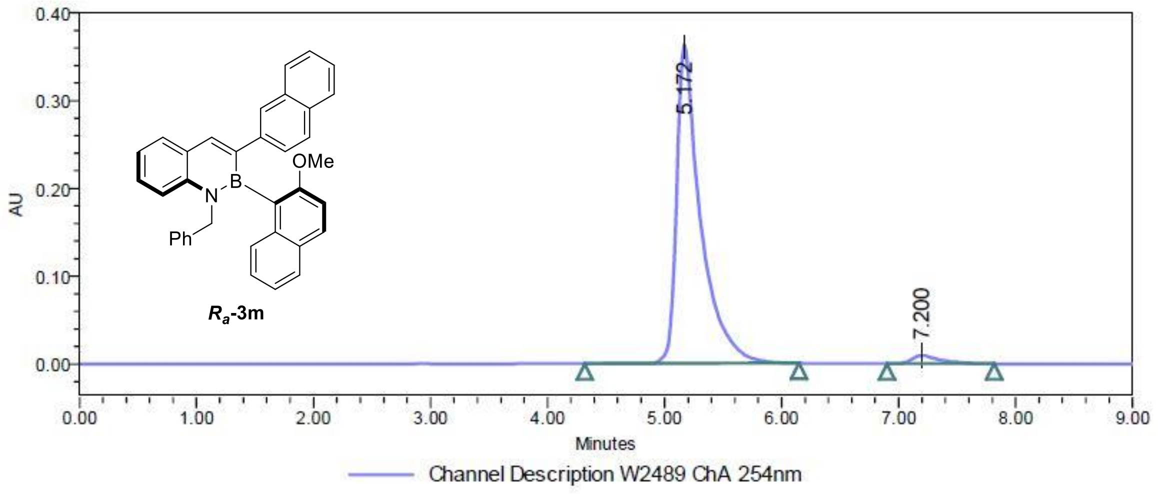

Peak Results

	Retention Time (min)	Int Type	Width (sec)	Area (µV*sec)	Height (µV)	% Area
1	5.172	BB	110.000	5143665	363350	97.02
2	7.200	BB	55.000	157900	9555	2.98

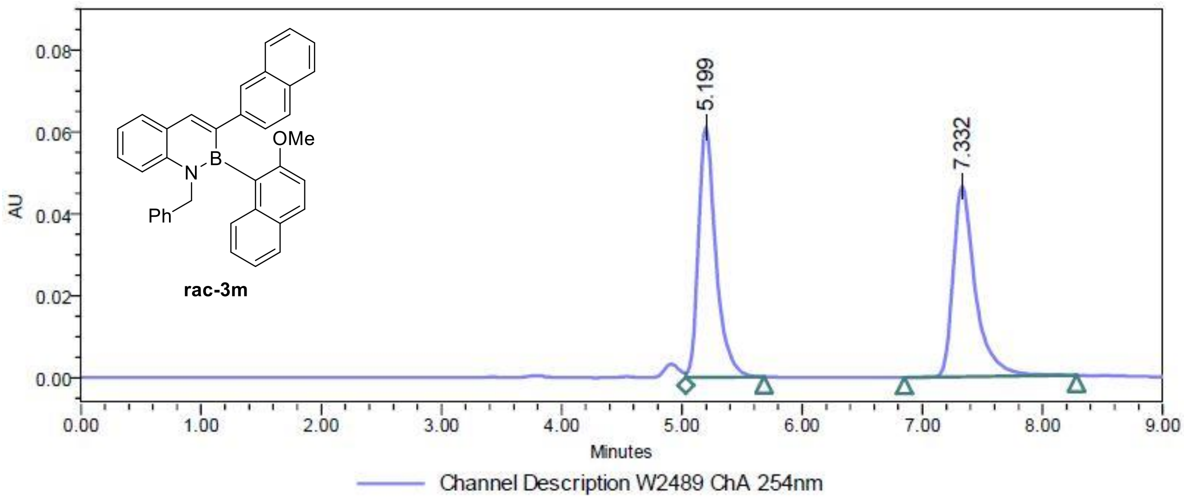

Peak Results

	Retention Time (min)	Int Type	Width (sec)	Area (µV*sec)	Height (µV)	% Area
1	5.199	VB	39.000	612886	60989	50.61
2	7.332	bb	86.000	598108	46526	49.39

Supplementary Figure 250. Chiral HPLC analysis of 3m

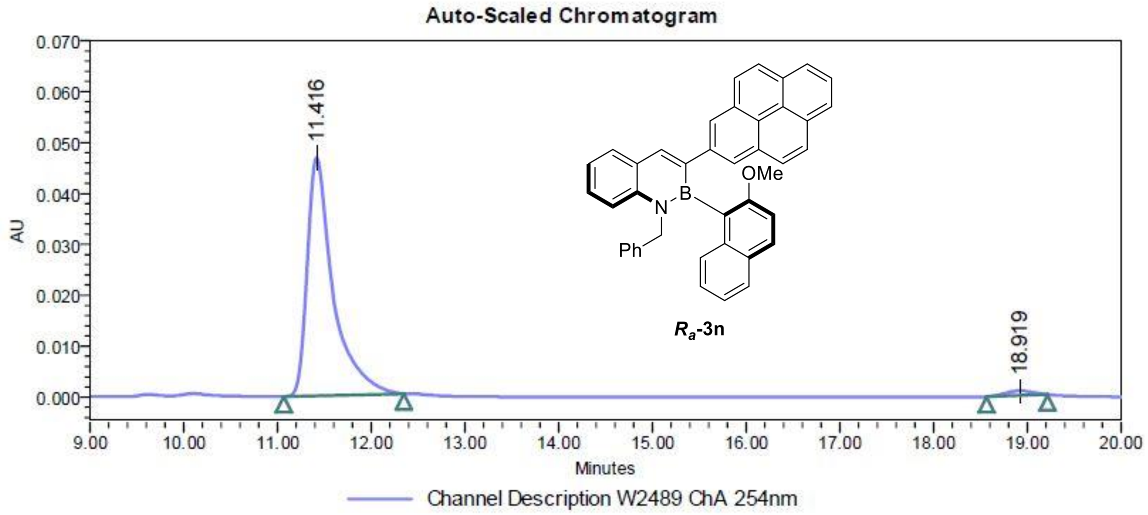

Peak Results

	Retention Time (min)	Int Type	Width (sec)	Area (µV*sec)	Height (µV)	% Area
1	11.416	BB	77.000	901410	46770	97.90
2	18.919	BB	39.000	19306	943	2.10

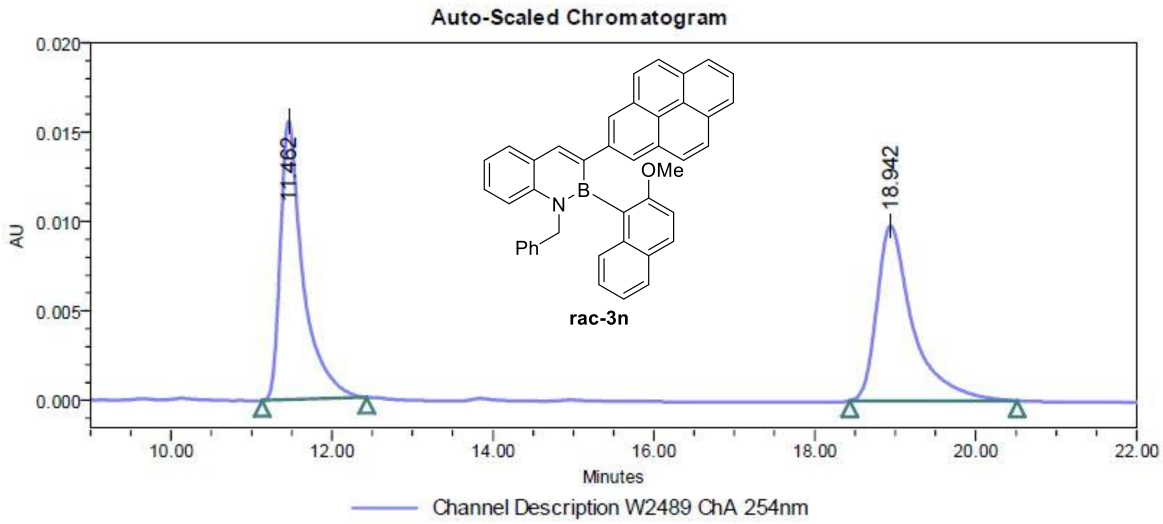

Peak Results

	Retention Time (min)	Int Type	Width (sec)	Area (µV*sec)	Height (µV)	% Area
1	11.462	Bb	78.000	304942	15609	50.03
2	18.942	Bb	125.000	304537	9796	49.97

Supplementary Figure 251. Chiral HPLC analysis of 3n

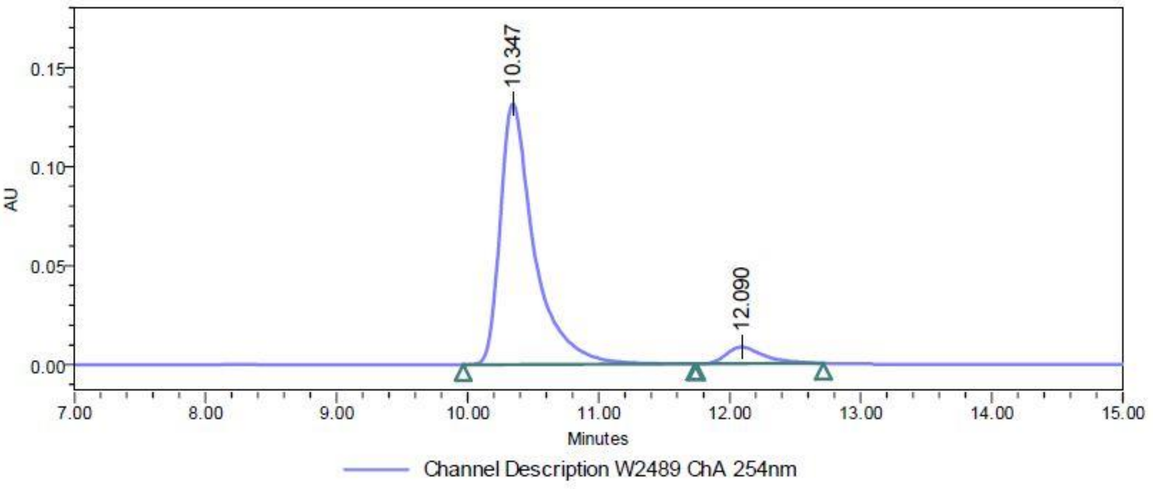

Peak Results

	Retention Time (min)	Int Type	Width (sec)	Area (µV*sec)	Height (µV)	% Area
1	10.347	BB	106.000	2368663	131580	93.29
2	12.090	BB	58.000	170453	8491	6.71

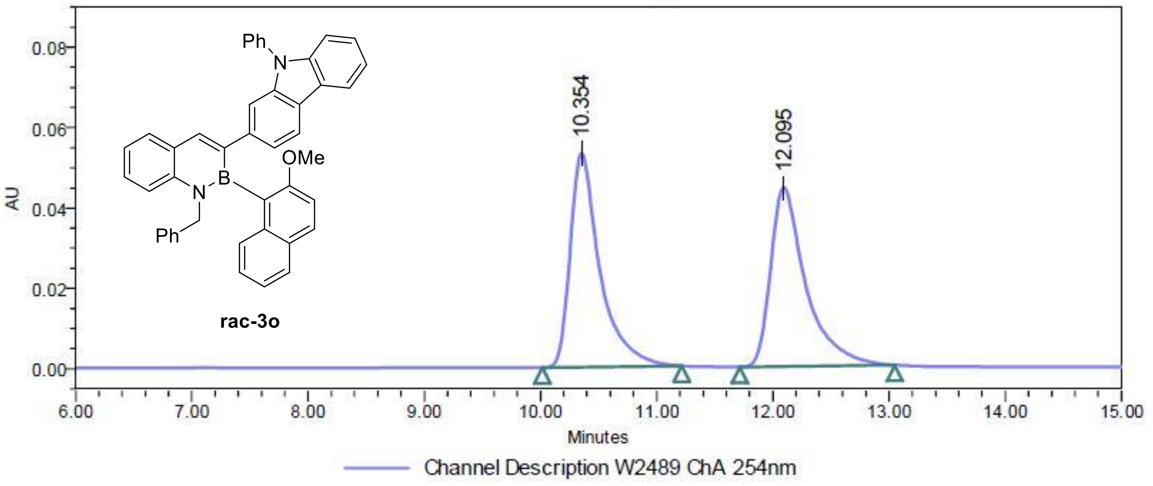

Peak Results

	Retention Time (min)	Int Type	Width (sec)	Area (µV*sec)	Height (µV)	% Area
1	10.354	BB	72.000	947663	53315	50.26
2	12.095	BB	80.000	937914	44500	49.74

Supplementary Figure 252. Chiral HPLC analysis of 3o

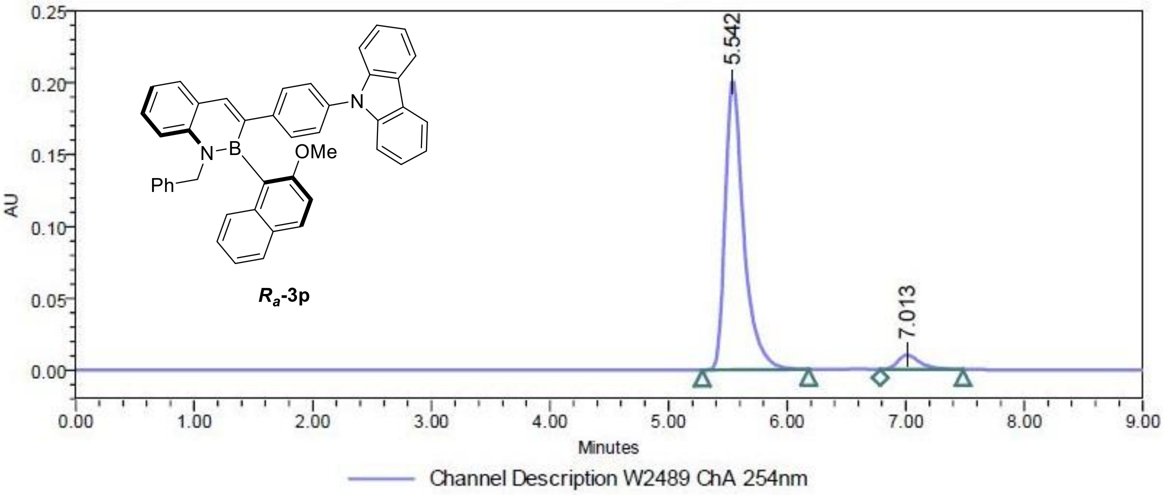

Peak Results

	Retention Time (min)	Int Type	Width (sec)	Area (μV*sec)	Height (μV)	% Area
1	5.542	BB	54.000	2189545	202002	94.14
2	7.013	VB	42.000	136235	10166	5.86

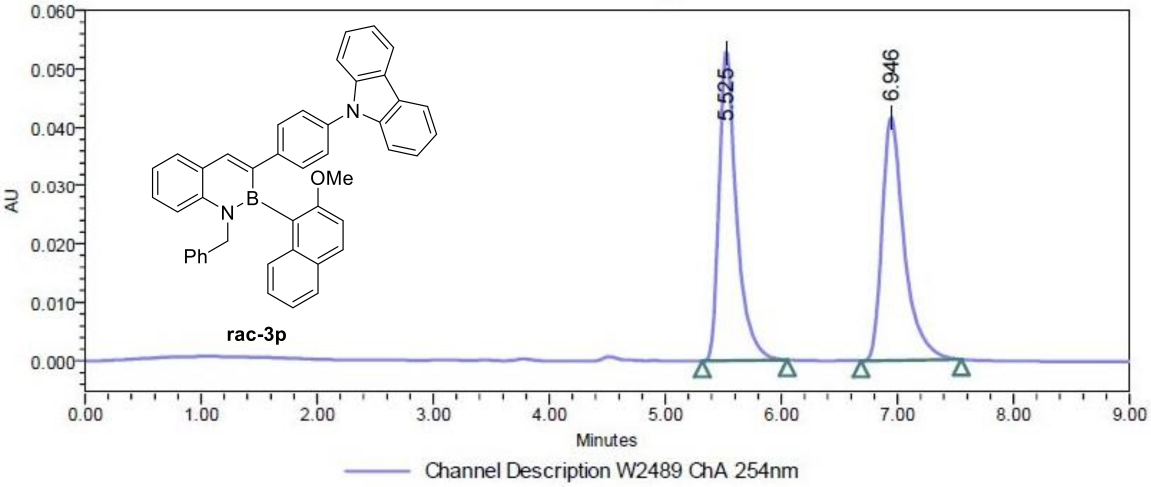

Peak Results

	Retention Time (min)	Int Type	Width (sec)	Area (μV*sec)	Height (μV)	% Area
1	5.525	BB	44.000	557672	53145	50.23
2	6.946	BB	52.000	552455	41721	49.77

Supplementary Figure 253. Chiral HPLC analysis of 3p

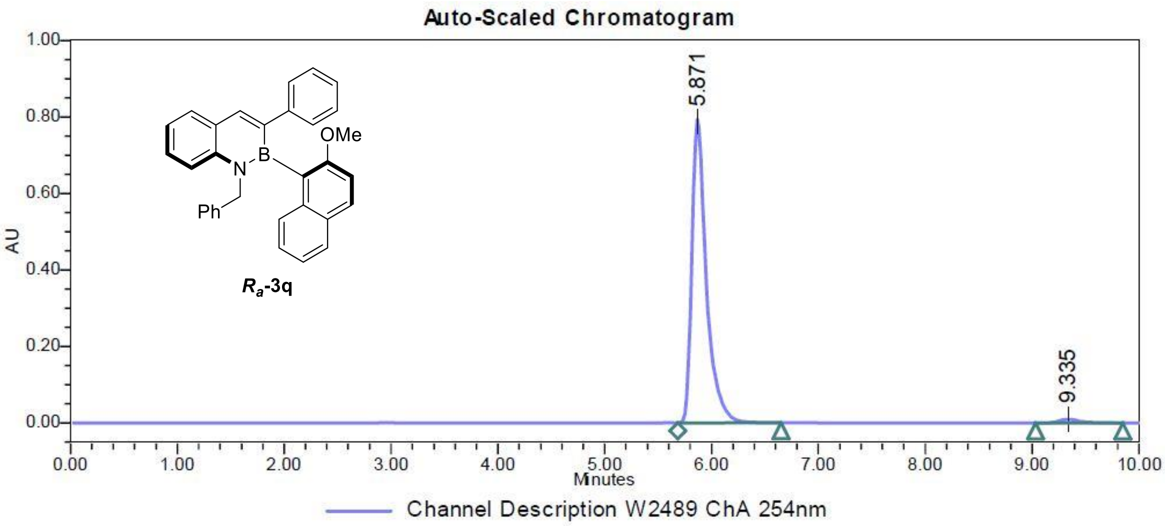

Peak Results

	Retention Time (min)	Int Type	Width (sec)	Area (µV*sec)	Height (µV)	% Area
1	5.871	VB	58.000	7462897	793706	98.30
2	9.335	BB	49.000	129061	9372	1.70

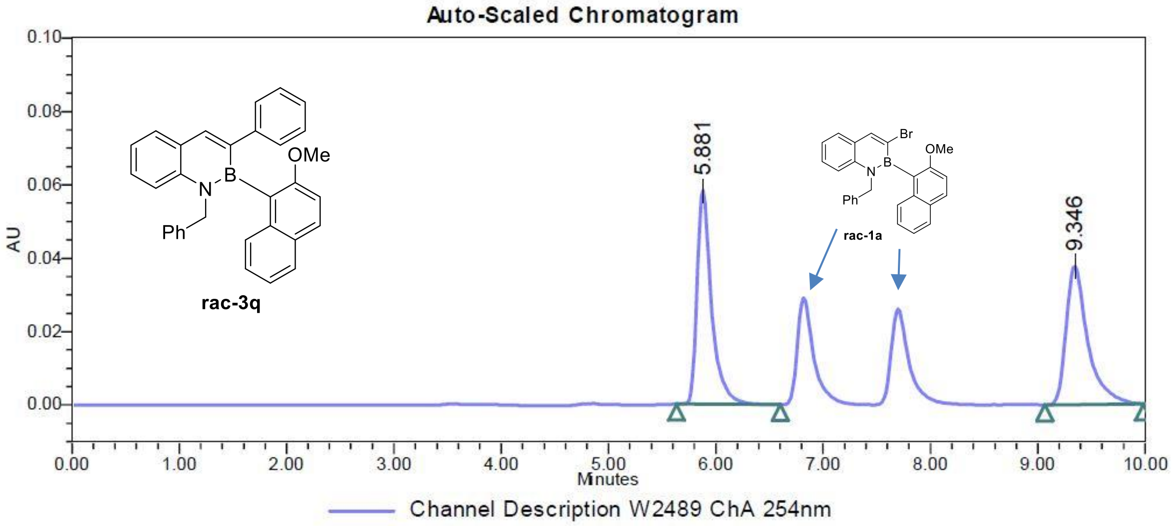

Peak Results

	Retention Time (min)	Int Type	Width (sec)	Area (µV*sec)	Height (µV)	% Area
1	5.881	BB	58.000	546197	58157	50.74
2	9.346	BB	55.000	530168	37725	49.26

Supplementary Figure 254. Chiral HPLC analysis of 3q

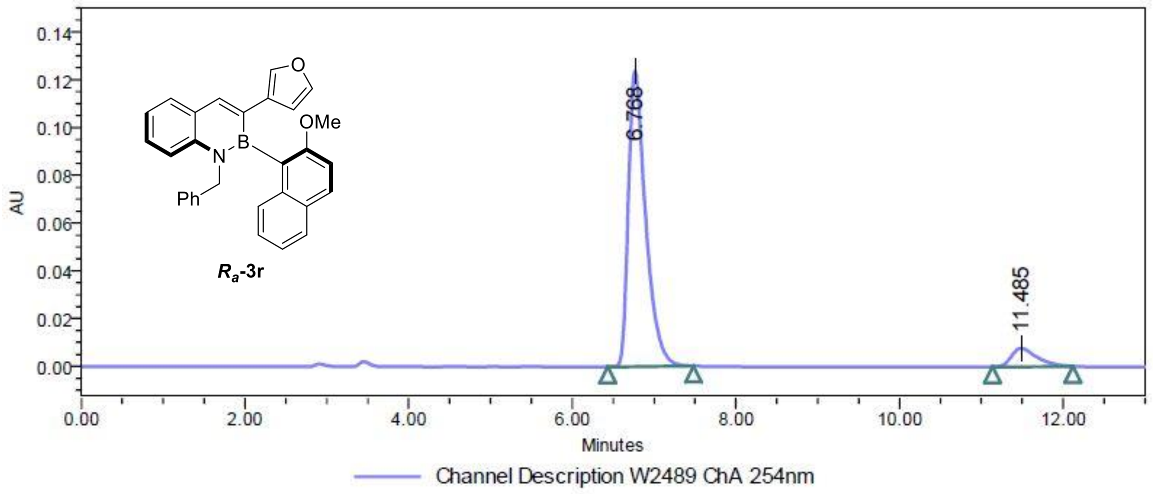

Peak Results

	Retention Time (min)	Int Type	Width (sec)	Area (µV*sec)	Height (µV)	% Area
1	6.768	BB	63.000	1821290	123841	91.61
2	11.485	BB	59.000	166886	7701	8.39

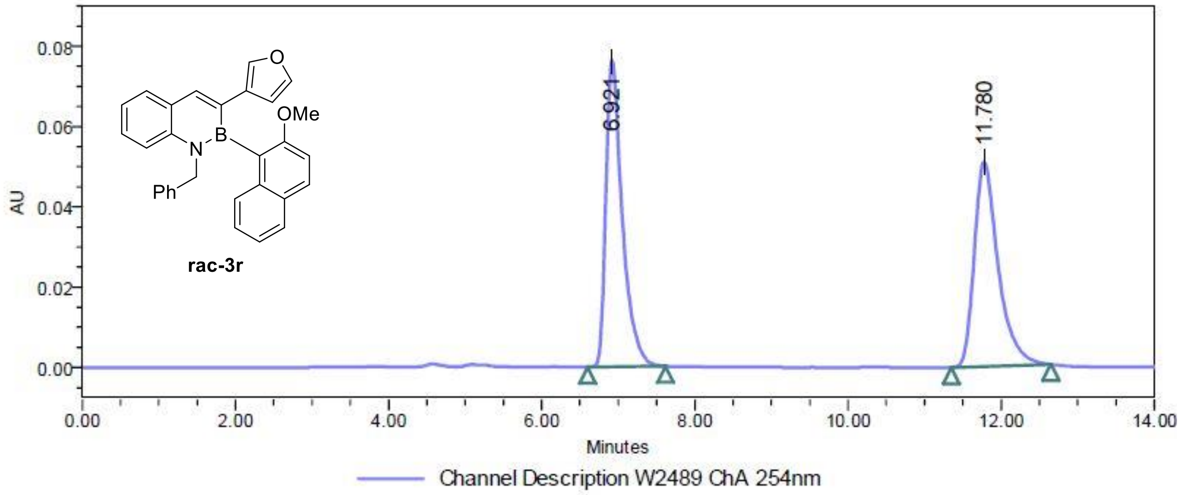

Peak Results

	Retention Time (min)	Int Type	Width (sec)	Area (µV*sec)	Height (µV)	% Area
1	6.921	BB	61.000	1136822	76420	50.64
2	11.780	BB	78.000	1108242	50936	49.36

Supplementary Figure 255. Chiral HPLC analysis of 3r

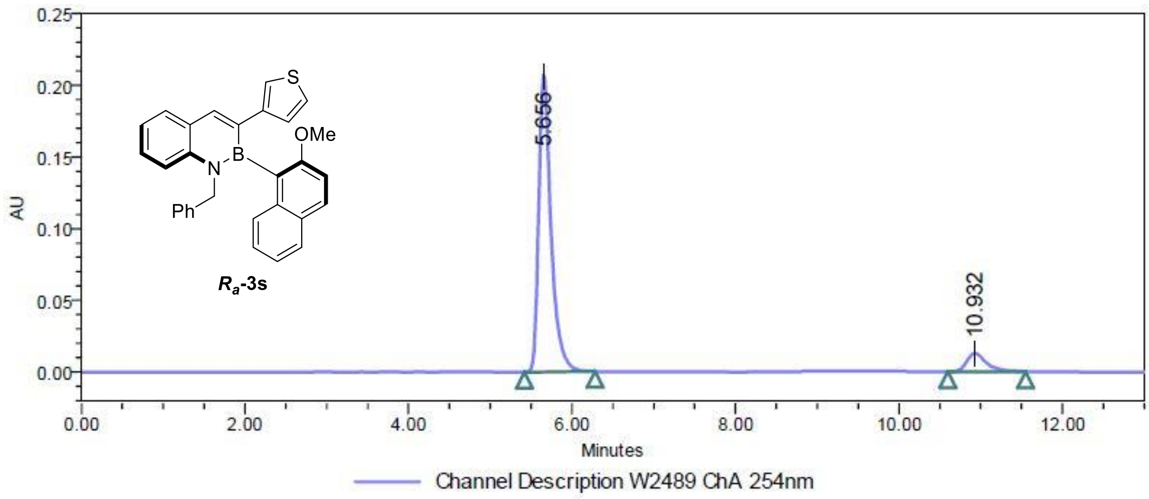

Peak Results

	Retention Time (min)	Int Type	Width (sec)	Area (µV*sec)	Height (µV)	% Area
1	5.656	BB	52.000	2162369	207541	90.84
2	10.932	BB	57.000	218164	12856	9.16

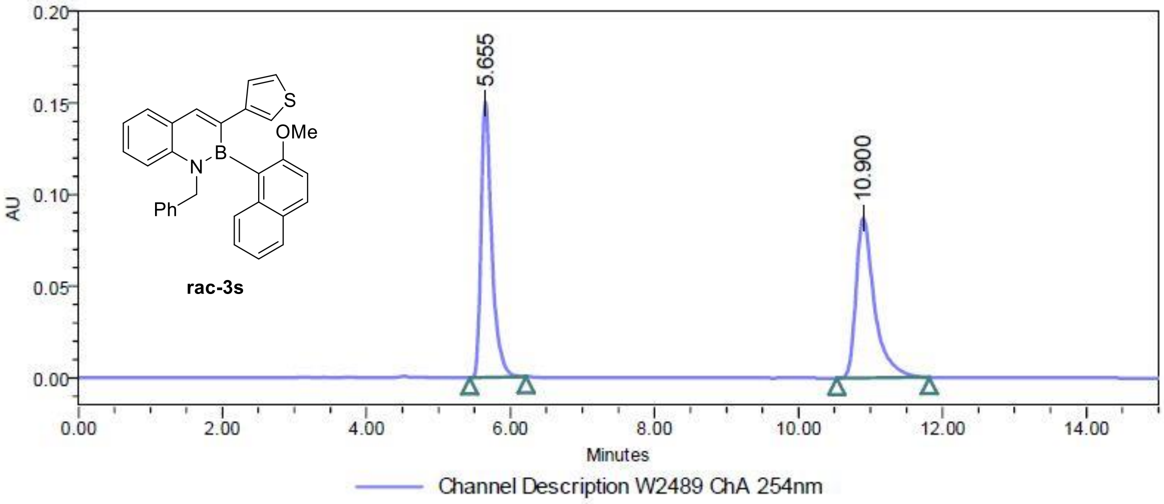

Peak Results

	Retention Time (min)	Int Type	Width (sec)	Area (µV*sec)	Height (µV)	% Area
1	5.655	BB	47.000	1555895	150692	49.96
2	10.900	BB	77.000	1558183	87238	50.04

Supplementary Figure 256. Chiral HPLC analysis of 3s

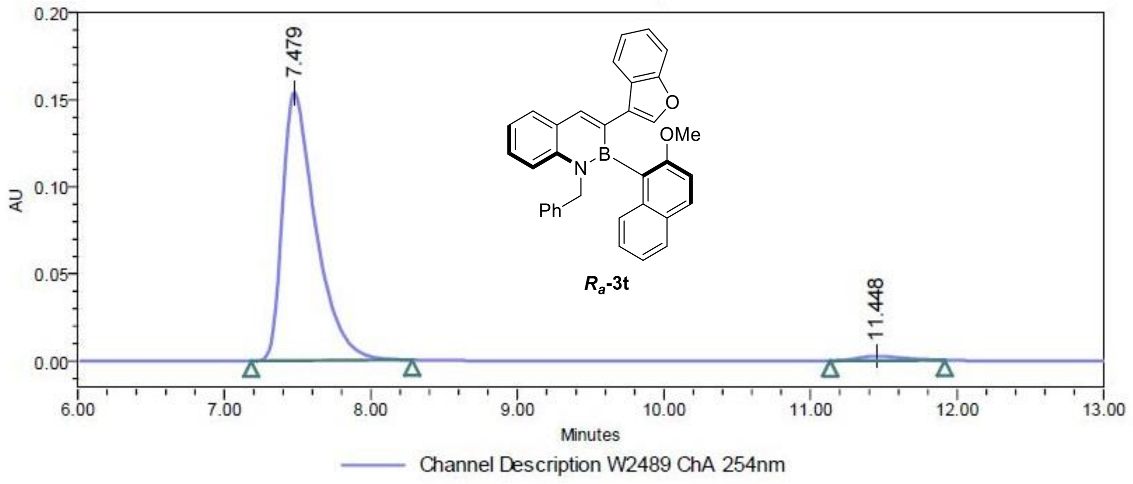

Peak Results

	Retention Time (min)	Int Type	Width (sec)	Area (µV*sec)	Height (µV)	% Area
1	7.479	BB	66.000	2424401	153890	97.87
2	11.448	BB	47.000	52753	2580	2.13

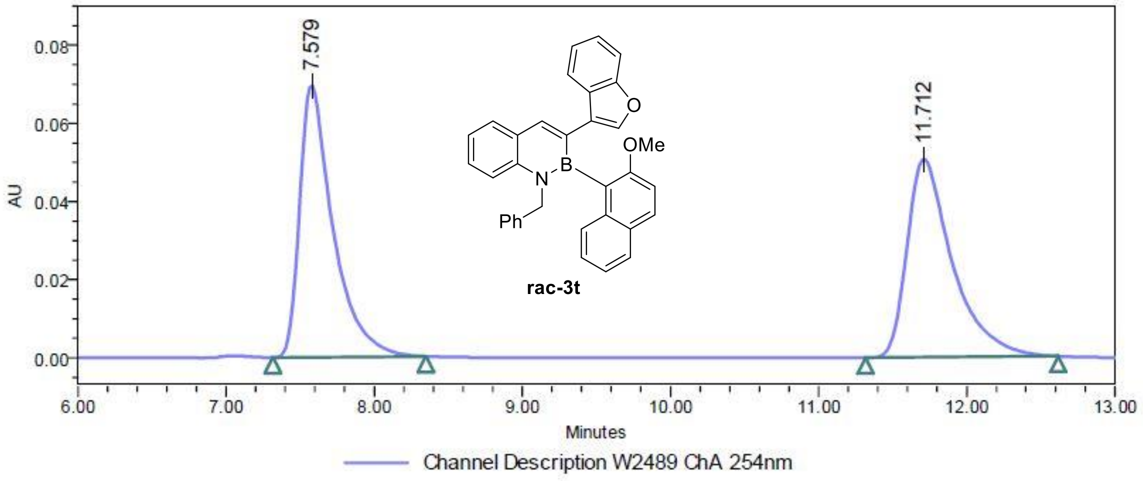

Peak Results

	Retention Time (min)	Int Type	Width (sec)	Area (µV*sec)	Height (µV)	% Area
1	7.579	BB	62.000	1087504	69528	50.16
2	11.712	BB	78.000	1080507	50716	49.84

Supplementary Figure 257. Chiral HPLC analysis of 3t

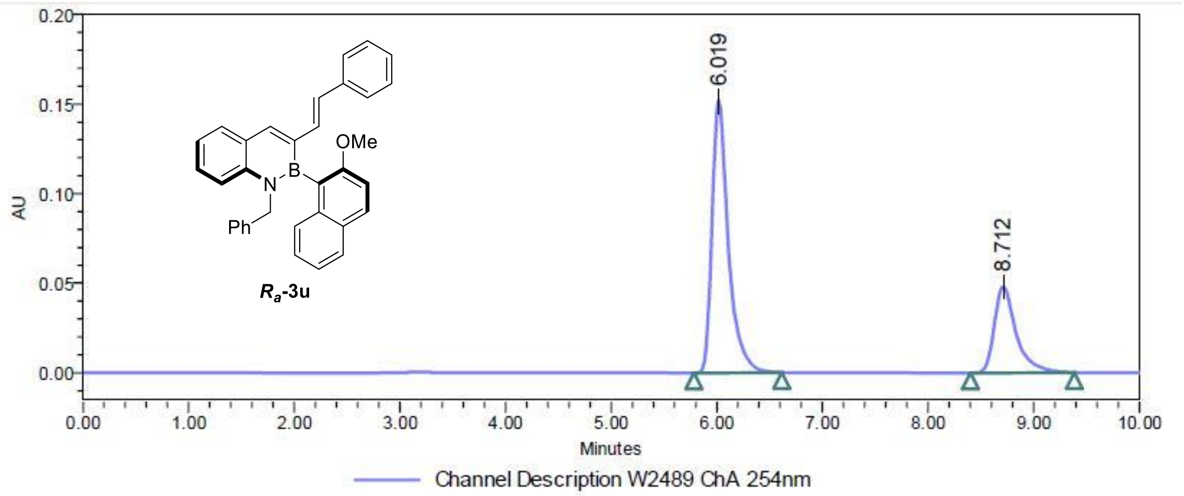

Peak Results

	Retention Time (min)	Int Type	Width (sec)	Area (µV*sec)	Height (µV)	% Area
1	6.019	BB	50.000	1629792	152013	70.23
2	8.712	BB	59.000	690928	48098	29.77

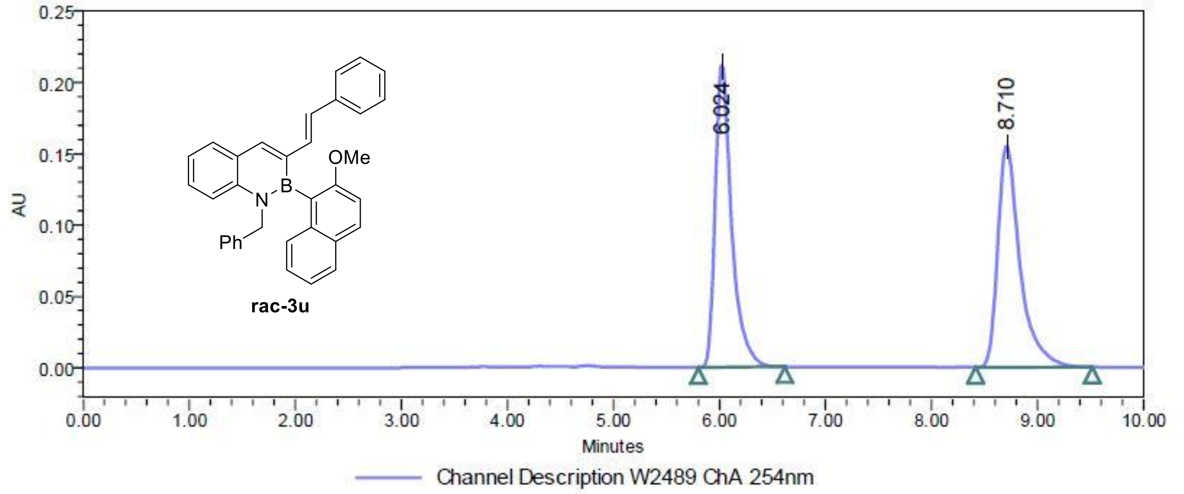

Peak Results

	Retention Time (min)	Int Type	Width (sec)	Area (µV*sec)	Height (µV)	% Area
1	6.024	BB	49.000	2278252	212551	50.13
2	8.710	BB	66.000	2266665	155127	49.87

Supplementary Figure 258. Chiral HPLC analysis of 3u

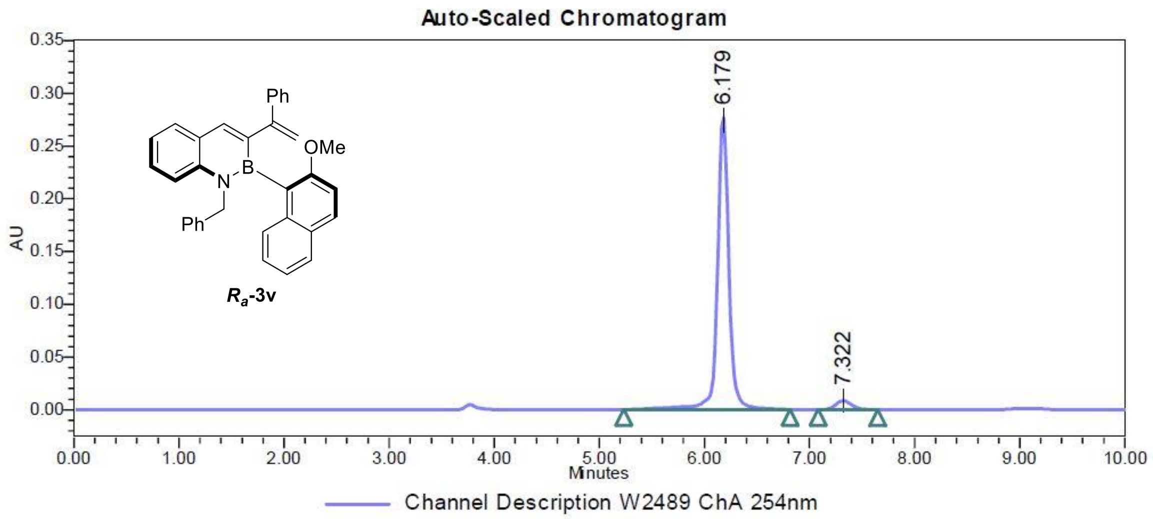

Peak Results

	Retention Time (min)	Int Type	Width (sec)	Area (µV*sec)	Height (µV)	% Area
1	6.179	BB	95.000	2098019	276174	95.87
2	7.322	BB	34.000	90327	8569	4.13

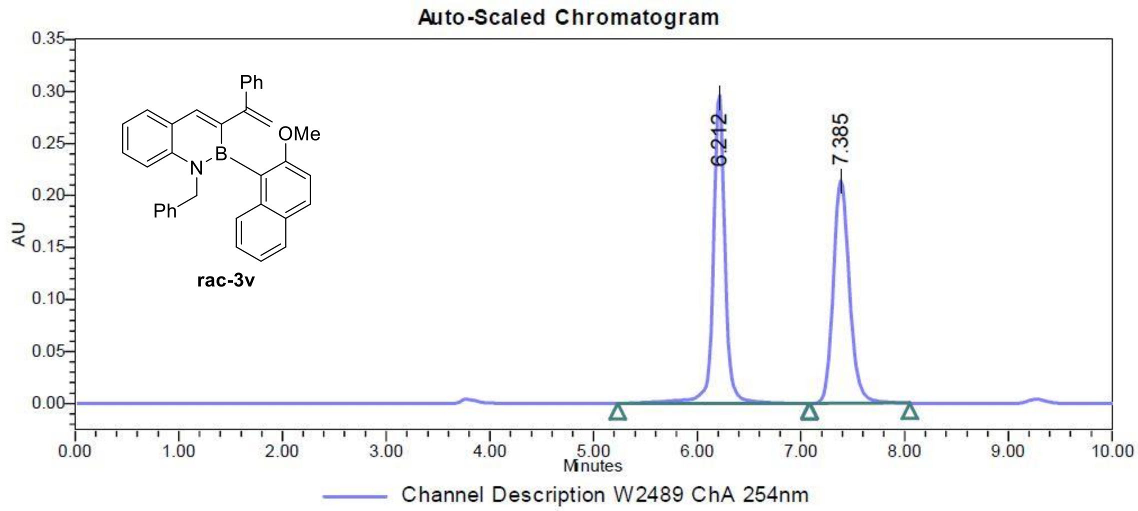

Peak Results

	Retention Time (min)	Int Type	Width (sec)	Area (µV*sec)	Height (µV)	% Area
1	6.212	BB	111.000	2333375	296039	49.99
2	7.385	BB	58.000	2334673	213980	50.01

Supplementary Figure 259. Chiral HPLC analysis of 3v

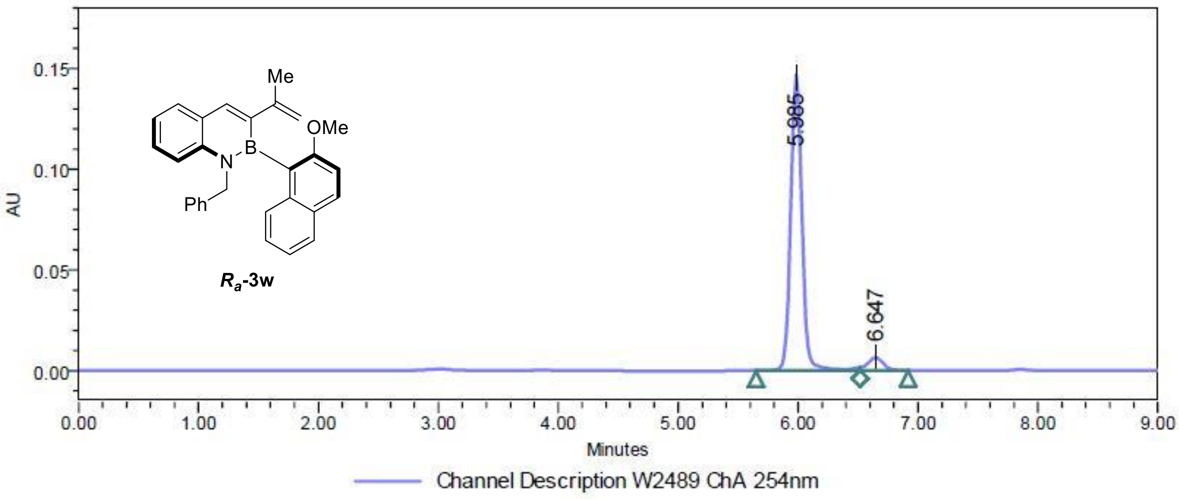

Peak Results

	Retention Time (min)	Int Type	Width (sec)	Area (µV*sec)	Height (µV)	% Area
1	5.985	bv	52.000	953407	146058	94.22
2	6.647	vB	24.000	58527	6412	5.78

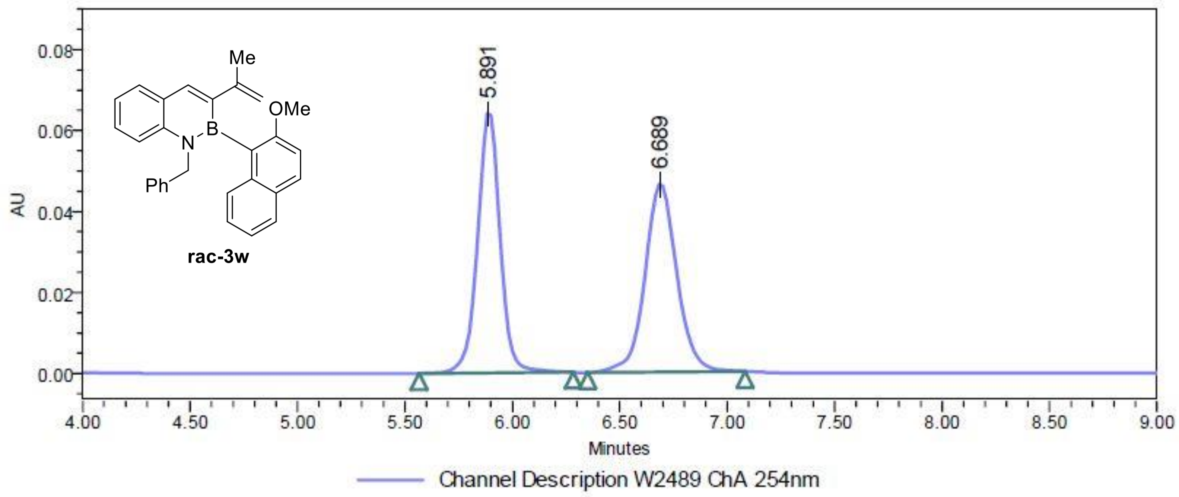

Peak Results

	Retention Time (min)	Int Type	Width (sec)	Area (µV*sec)	Height (µV)	% Area
1	5.891	BB	43.000	469799	64469	49.48
2	6.689	BB	44.000	479757	46465	50.52

Supplementary Figure 260. Chiral HPLC analysis of 3w

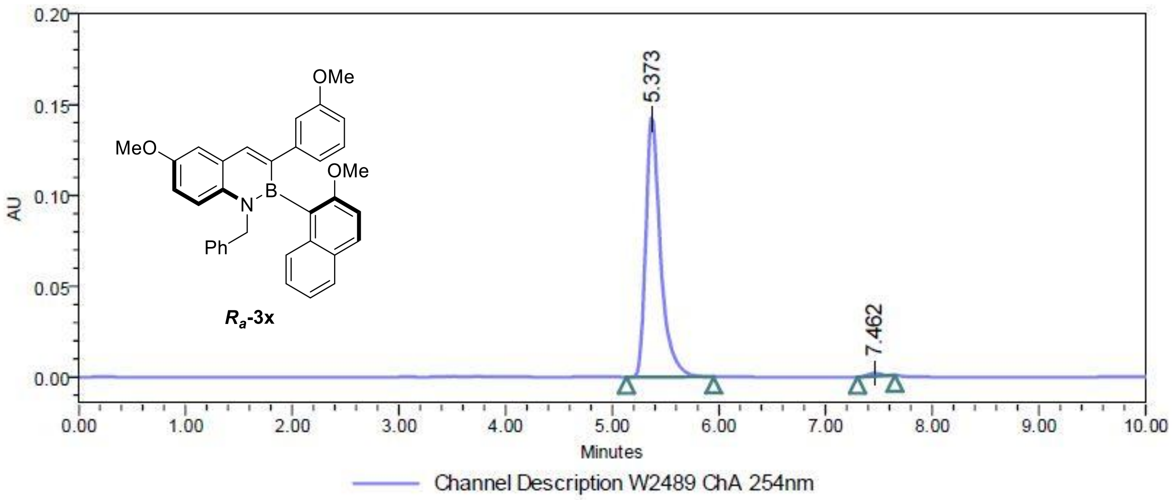

Peak Results

	Retention Time (min)	Int Type	Width (sec)	Area (µV*sec)	Height (µV)	% Area
1	5.373	BB	49.000	1383622	143155	98.89
2	7.462	BB	21.000	15569	1788	1.11

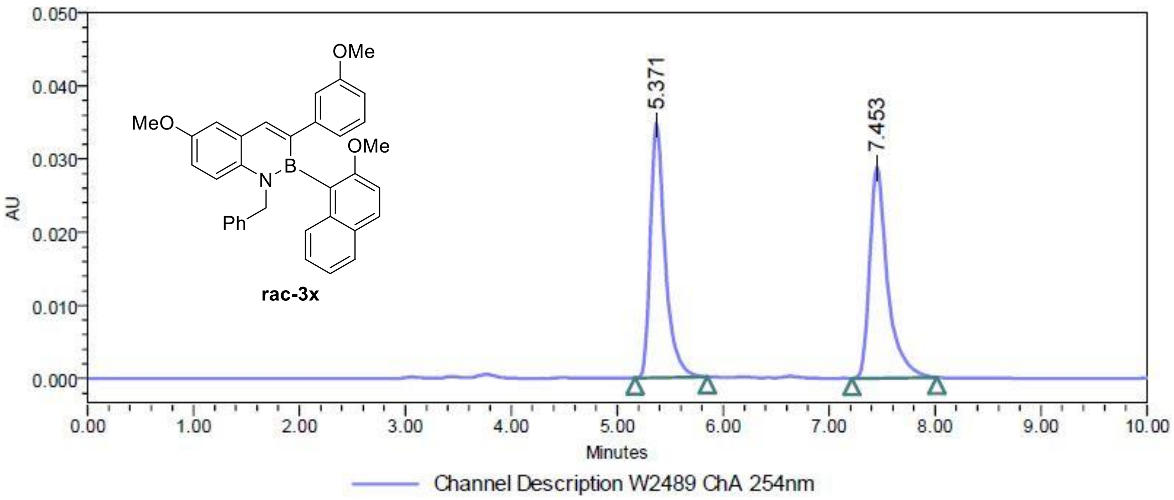

Peak Results

	Retention Time (min)	Int Type	Width (sec)	Area (µV*sec)	Height (µV)	% Area
1	5.371	BB	41.000	335771	34752	49.84
2	7.453	BB	48.000	337943	28811	50.16

Supplementary Figure 261. Chiral HPLC analysis of 3x

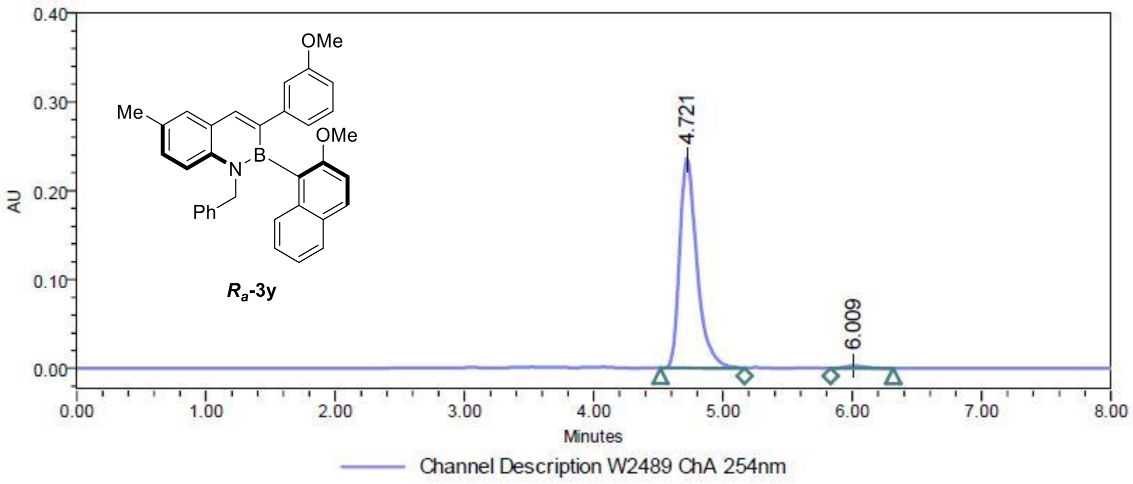

Peak Results

	Retention Time (min)	Int Type	Width (sec)	Area (µV*sec)	Height (µV)	% Area
1	4.721	BV	39.000	2201379	236365	98.71
2	6.009	VB	29.000	28772	2946	1.29

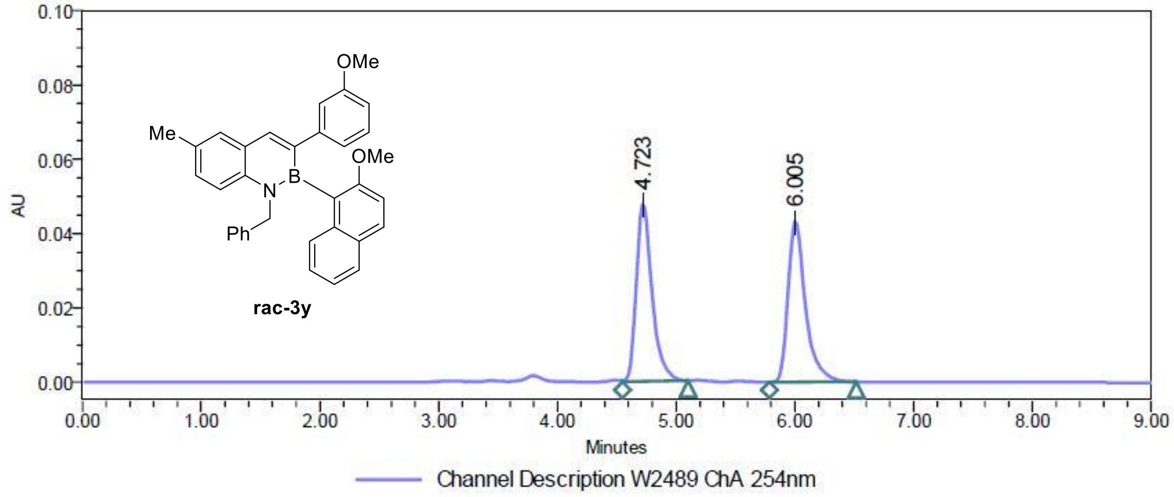

Peak Results

	Retention Time (min)	Int Type	Width (sec)	Area (µV*sec)	Height (µV)	% Area
1	4.723	VB	33.000	438372	47825	49.89
2	6.005	VB	44.000	440316	43286	50.11

Supplementary Figure 262. Chiral HPLC analysis of 3y

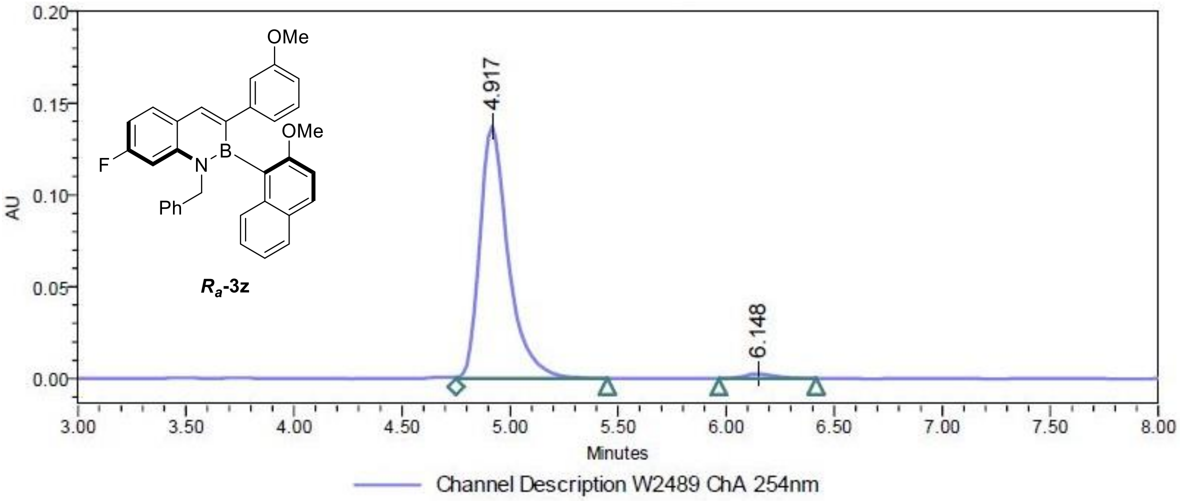

Peak Results

	Retention Time (min)	Int Type	Width (sec)	Area (µV*sec)	Height (µV)	% Area
1	4.917	VB	42.000	1258644	137152	98.10
2	6.148	BB	27.000	24424	2555	1.90

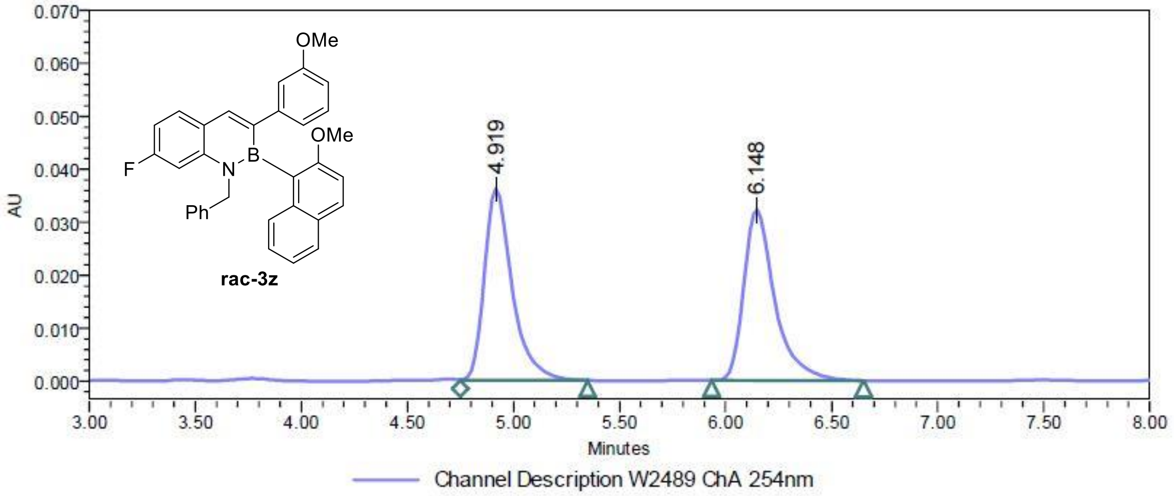

Peak Results

	Retention Time (min)	Int Type	Width (sec)	Area (µV*sec)	Height (µV)	% Area
1	4.919	VB	36.000	330478	36152	50.07
2	6.148	BB	43.000	329548	32207	49.93

Supplementary Figure 263. Chiral HPLC analysis of 3z

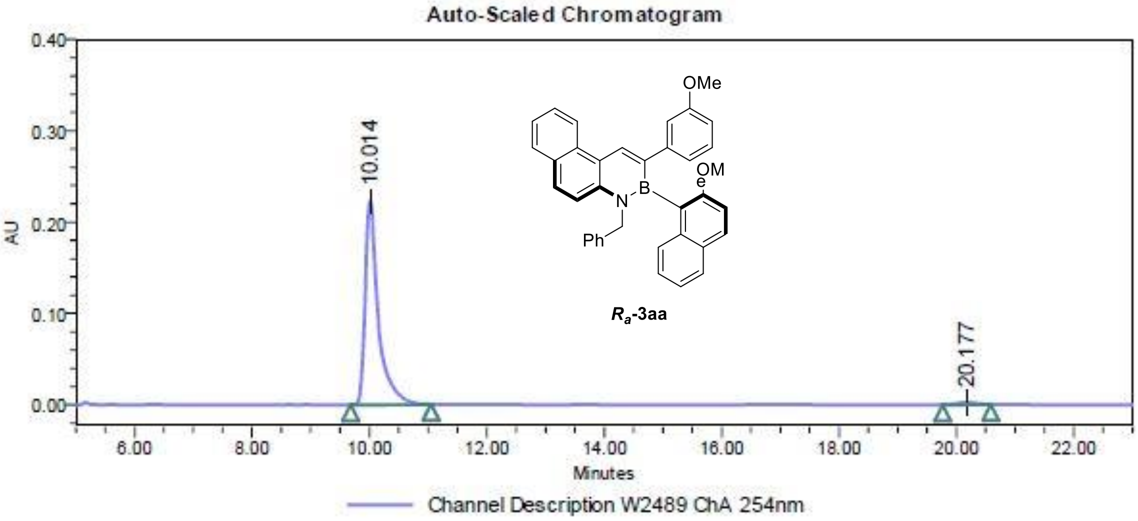

Peak Results

	Retention Time (min)	Int Type	Width (sec)	Area (µV*sec)	Height (µV)	% Area
1	10.014	BB	82.000	3469143	223397	98.53
2	20.177	BB	49.000	51855	2358	1.47

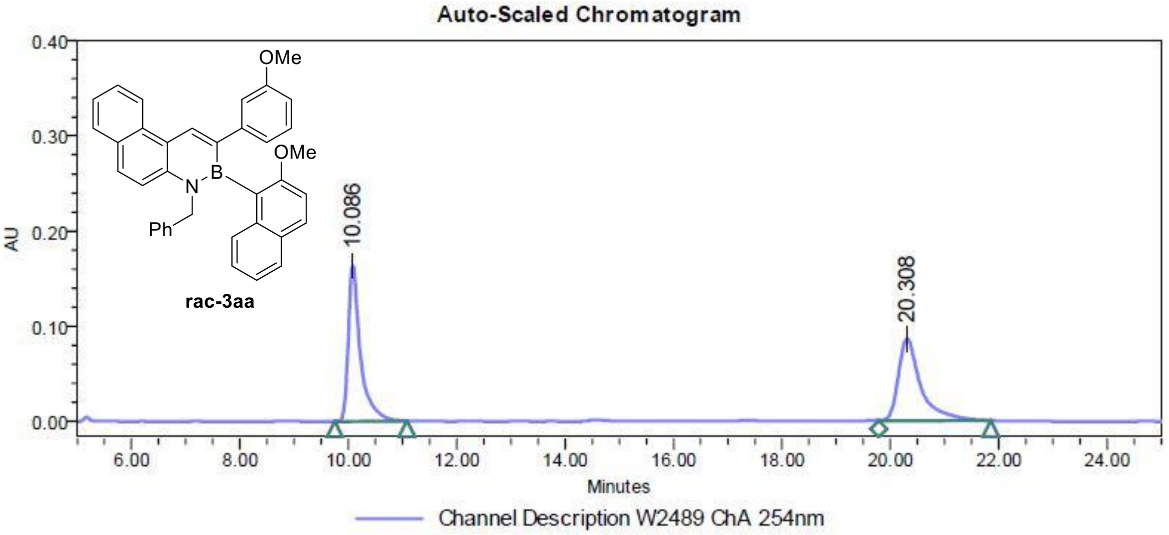

Peak Results

	Retention Time (min)	Int Type	Width (sec)	Area (µV*sec)	Height (µV)	% Area
1	10.086	BB	80.000	2568594	163628	50.63
2	20.308	VB	124.000	2504357	86291	49.37

Supplementary Figure 264. Chiral HPLC analysis of 3aa

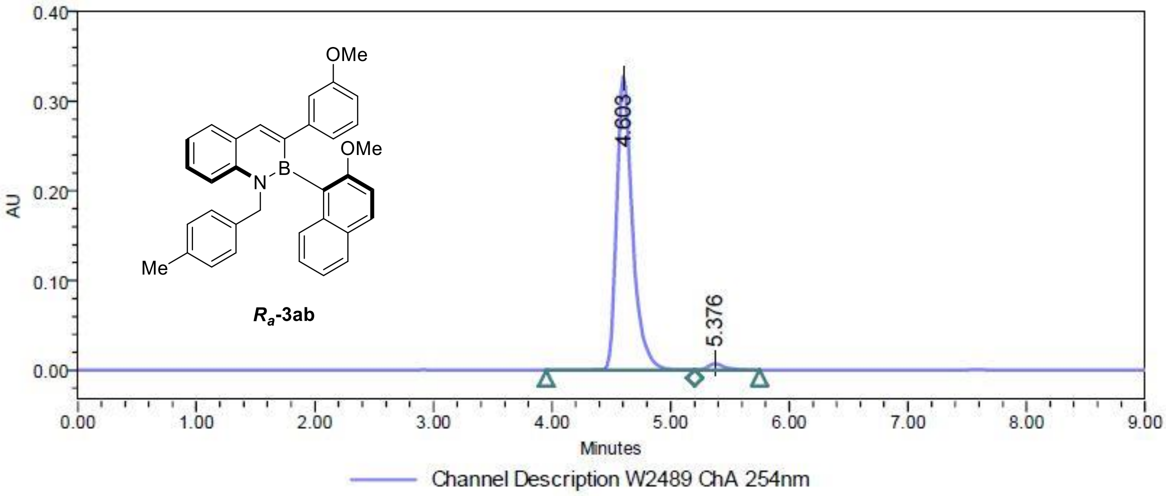

Peak Results

	Retention Time (min)	Int Type	Width (sec)	Area (µV*sec)	Height (µV)	% Area
1	4.603	BV	75.000	3032657	327012	97.74
2	5.376	VB	33.000	70103	7135	2.26

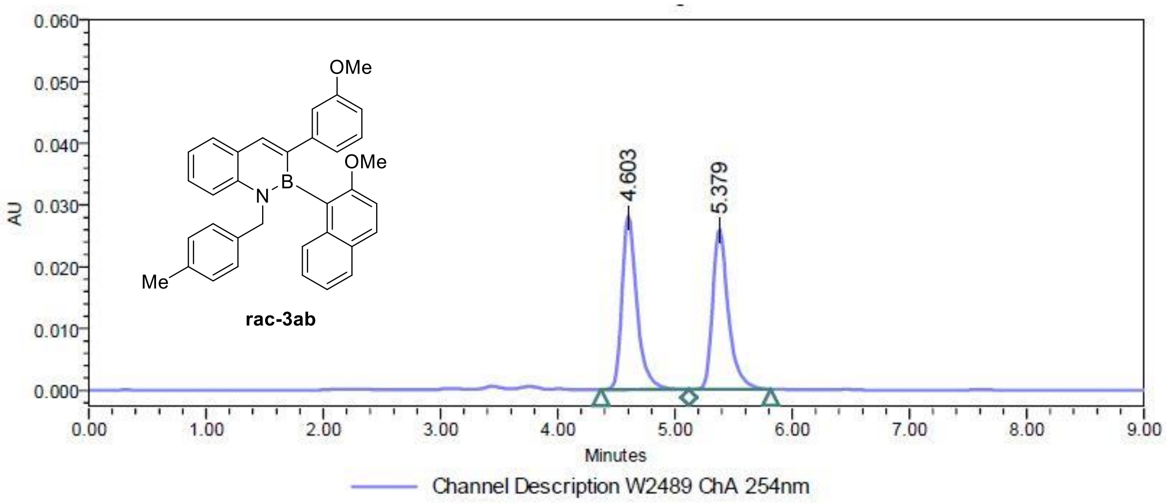

Peak Results

	Retention Time (min)	Int Type	Width (sec)	Area (µV*sec)	Height (µV)	% Area
1	4.603	BV	45.000	249983	28008	50.78
2	5.379	VB	42.000	242346	25915	49.22

Supplementary Figure 265. Chiral HPLC analysis of 3ab

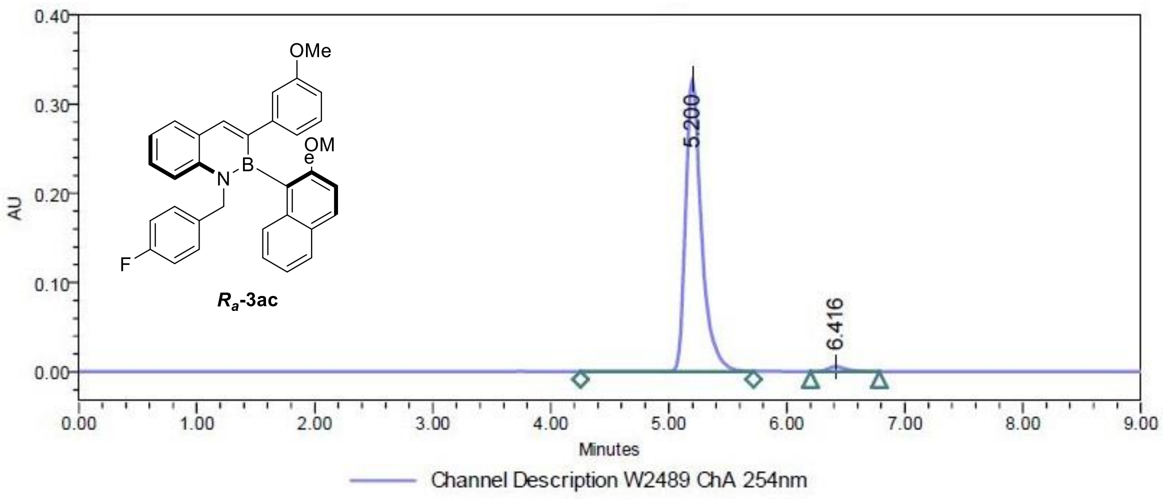

Peak Results

	Retention Time (min)	Int Type	Width (sec)	Area (µV*sec)	Height (µV)	% Area
1	5.200	VV	88.000	3063730	326924	98.12
2	6.416	BB	35.000	58766	5706	1.88

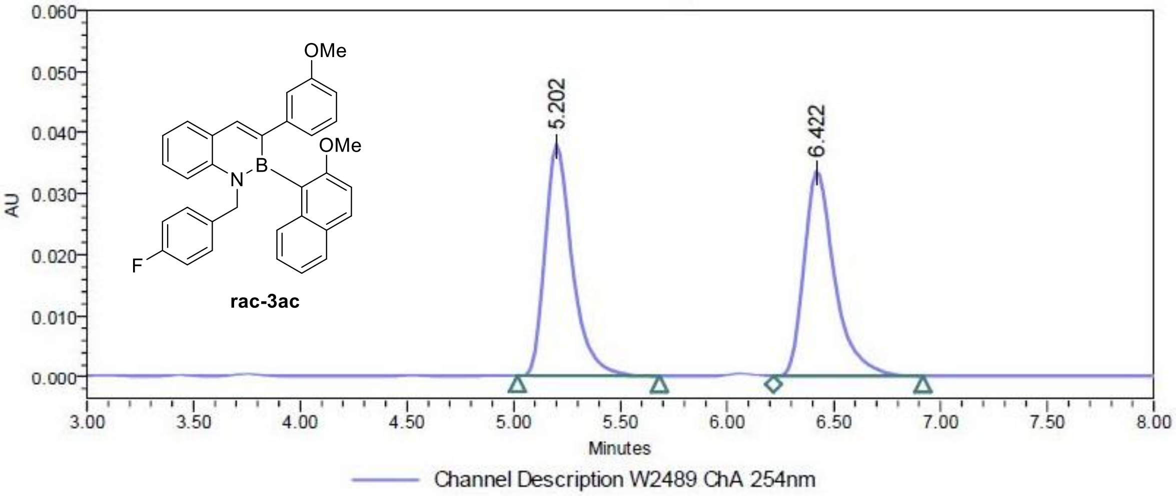

Peak Results

	Retention Time (min)	Int Type	Width (sec)	Area (µV*sec)	Height (µV)	% Area
1	5.202	BB	40.000	339515	37725	49.92
2	6.422	VB	42.000	340638	33562	50.08

Supplementary Figure 266. Chiral HPLC analysis of 3ac

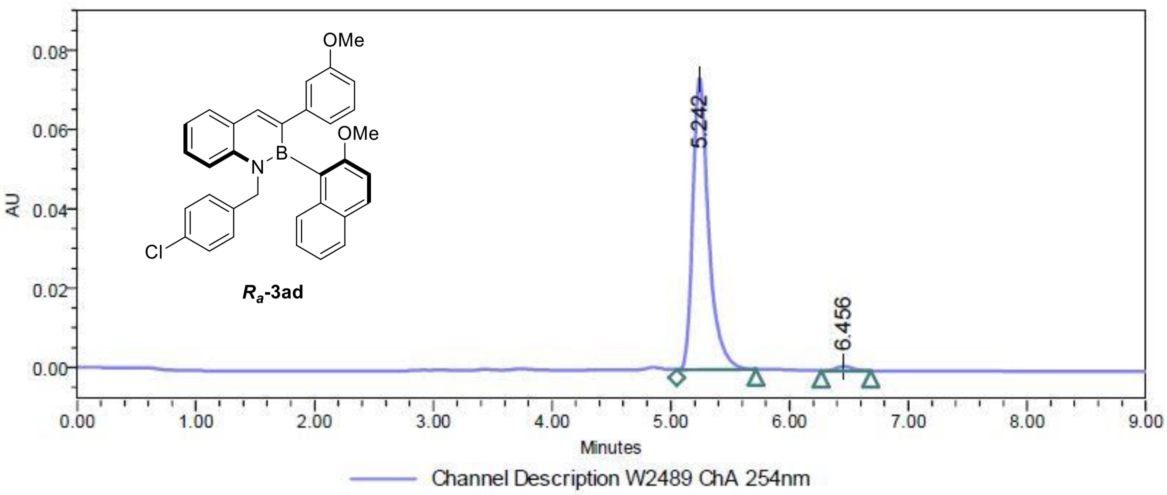

***R*$_a$-3ad**

Peak Results

	Retention Time (min)	Int Type	Width (sec)	Area (μV*sec)	Height (μV)	% Area
1	5.242	VB	40.000	683080	73884	98.53
2	6.456	BB	25.000	10188	1095	1.47

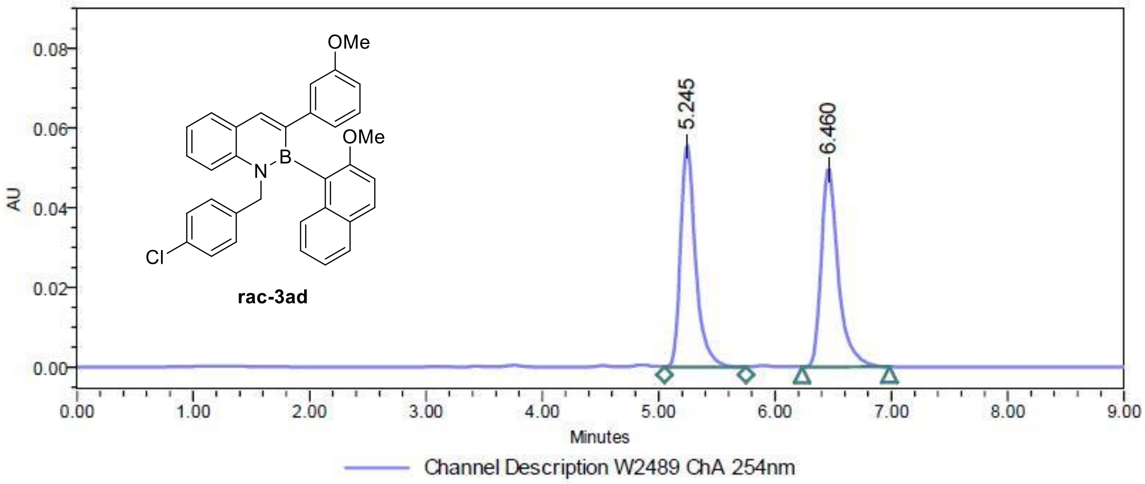

rac-3ad

Peak Results

	Retention Time (min)	Int Type	Width (sec)	Area (μV*sec)	Height (μV)	% Area
1	5.245	VV	42.000	502099	55639	49.90
2	6.460	BB	45.000	504079	49675	50.10

Supplementary Figure 267. Chiral HPLC analysis of 3ad

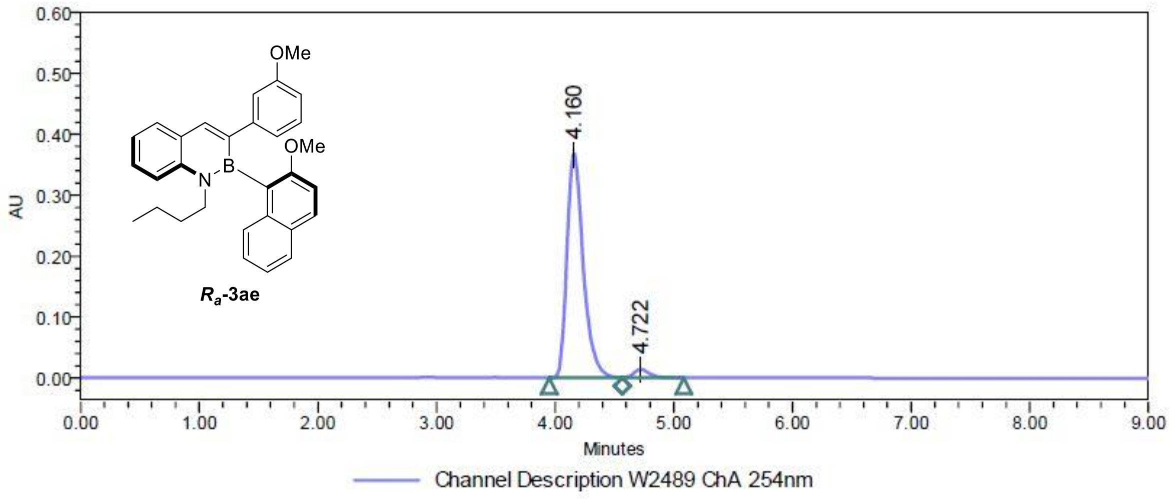

Peak Results

	Retention Time (min)	Int Type	Width (sec)	Area (μV*sec)	Height (μV)	% Area
1	4.160	BV	37.000	3499067	368527	96.33
2	4.722	VB	31.000	133405	14110	3.67

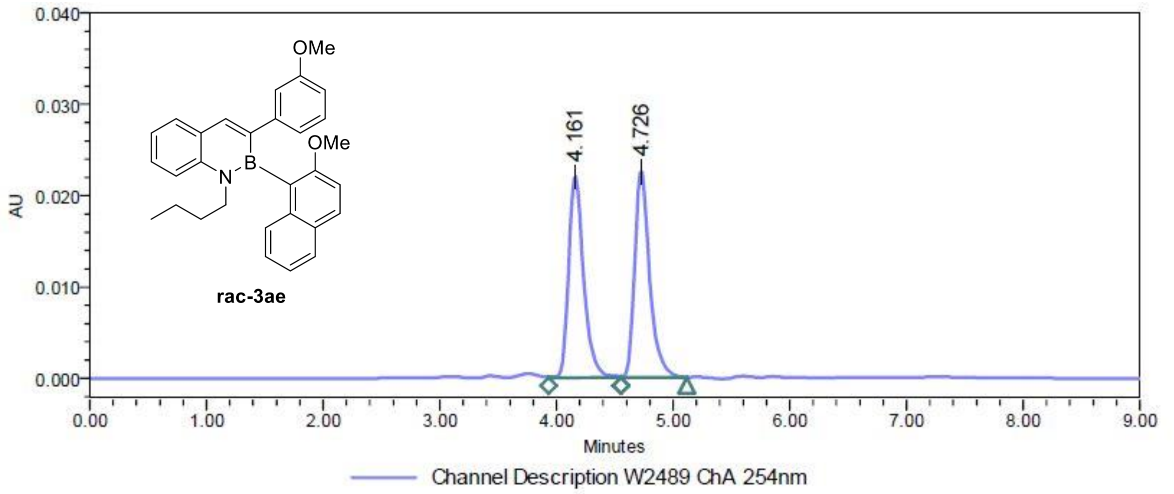

Peak Results

	Retention Time (min)	Int Type	Width (sec)	Area (μV*sec)	Height (μV)	% Area
1	4.161	VV	37.000	198066	22069	49.60
2	4.726	VB	34.000	201252	22595	50.40

Supplementary Figure 268. Chiral HPLC analysis of 3ae

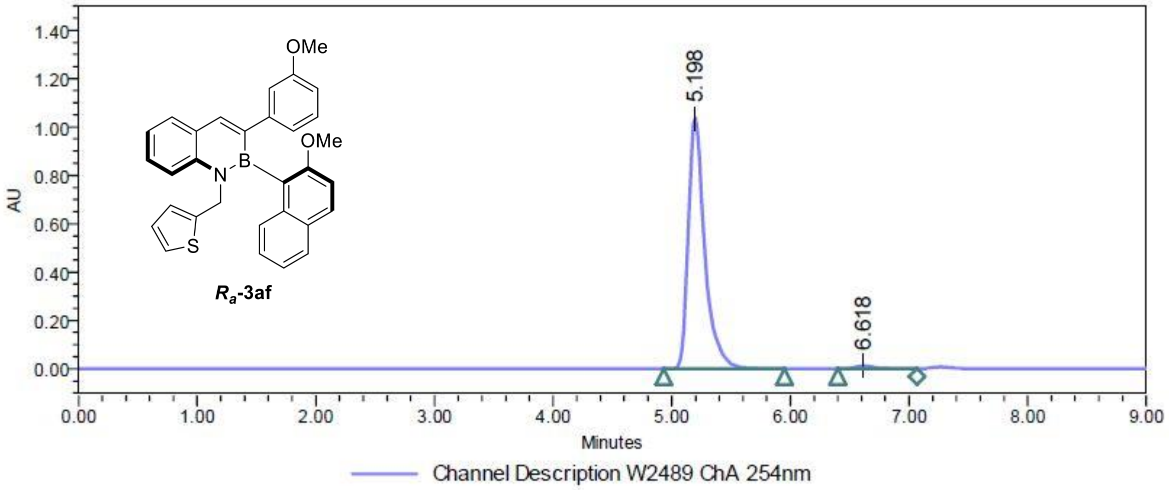

Peak Results

	Retention Time (min)	Int Type	Width (sec)	Area (µV*sec)	Height (µV)	% Area
1	5.198	BB	61.000	9957639	1034630	98.69
2	6.618	BV	40.000	132350	12512	1.31

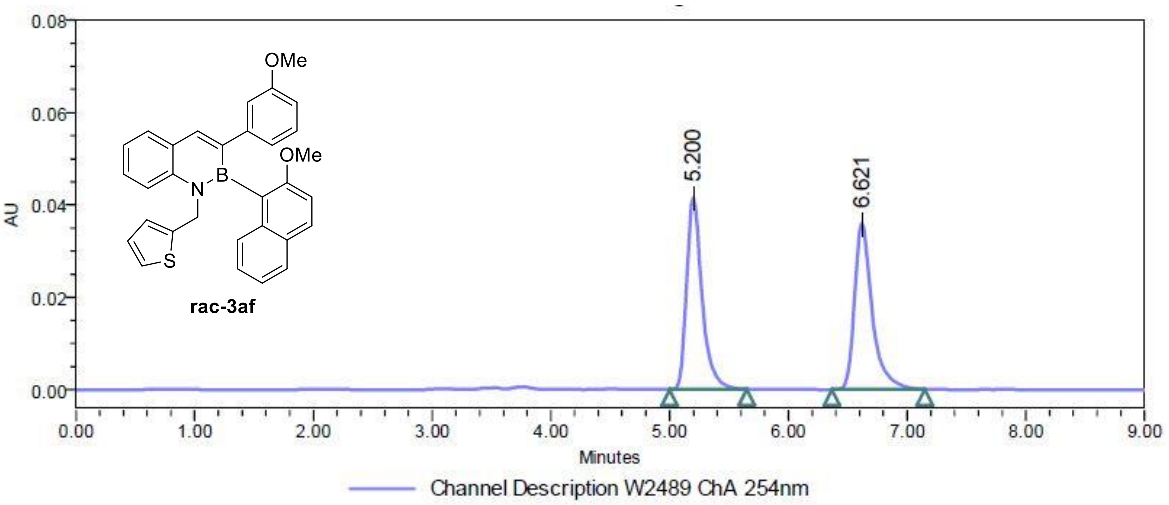

Peak Results

	Retention Time (min)	Int Type	Width (sec)	Area (µV*sec)	Height (µV)	% Area
1	5.200	BB	39.000	373295	41318	49.97
2	6.621	BB	47.000	373734	35984	50.03

Supplementary Figure 269. Chiral HPLC analysis of 3af

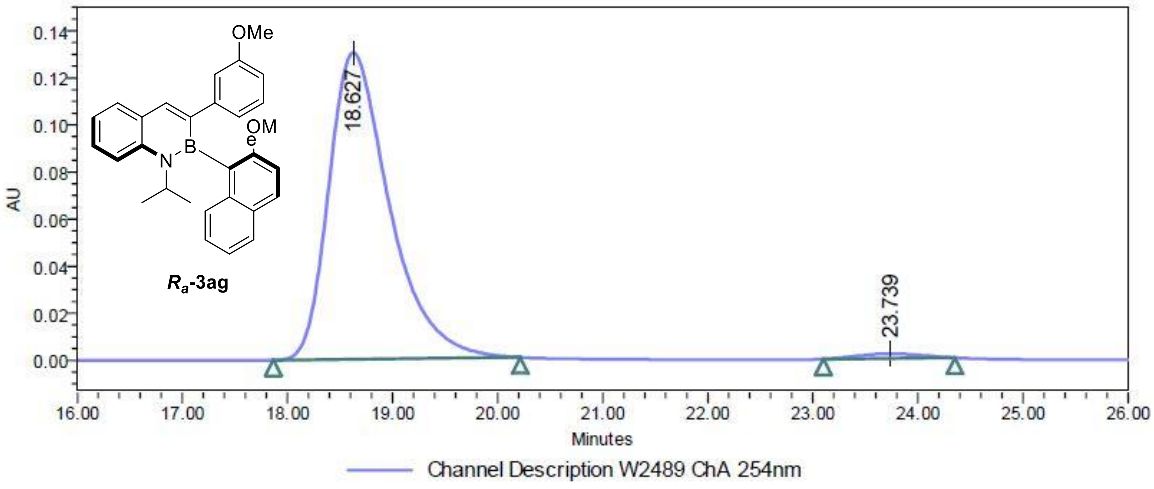

Peak Results

	Retention Time (min)	Int Type	Width (sec)	Area (µV*sec)	Height (µV)	% Area
1	18.627	BB	141.000	5236630	130246	98.46
2	23.739	BB	75.000	82077	1949	1.54

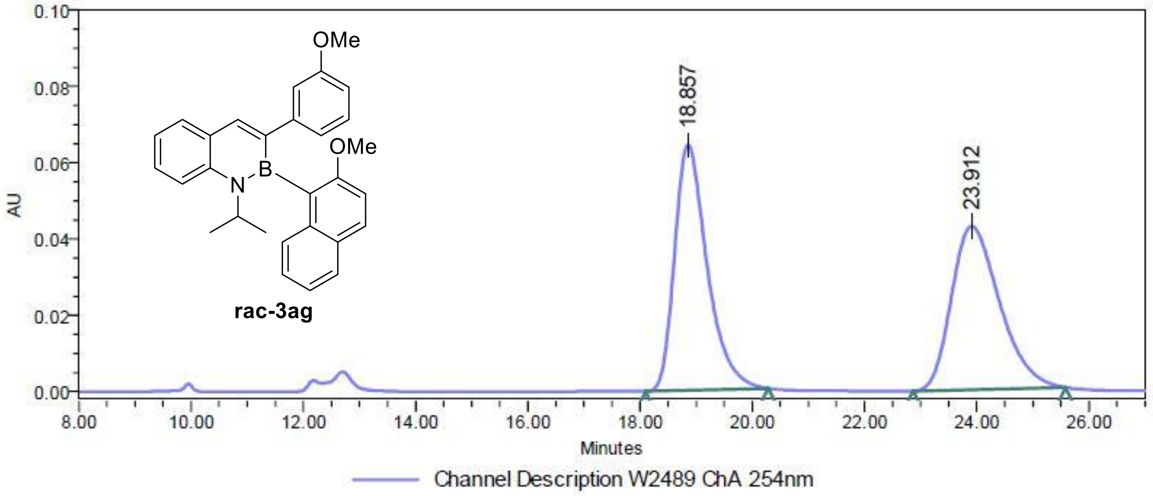

Peak Results

	Retention Time (min)	Int Type	Width (sec)	Area (µV*sec)	Height (µV)	% Area
1	18.857	BB	131.000	2594105	64320	50.36
2	23.912	BB	163.000	2556610	42870	49.64

Supplementary Figure 270. Chiral HPLC analysis of 3ag

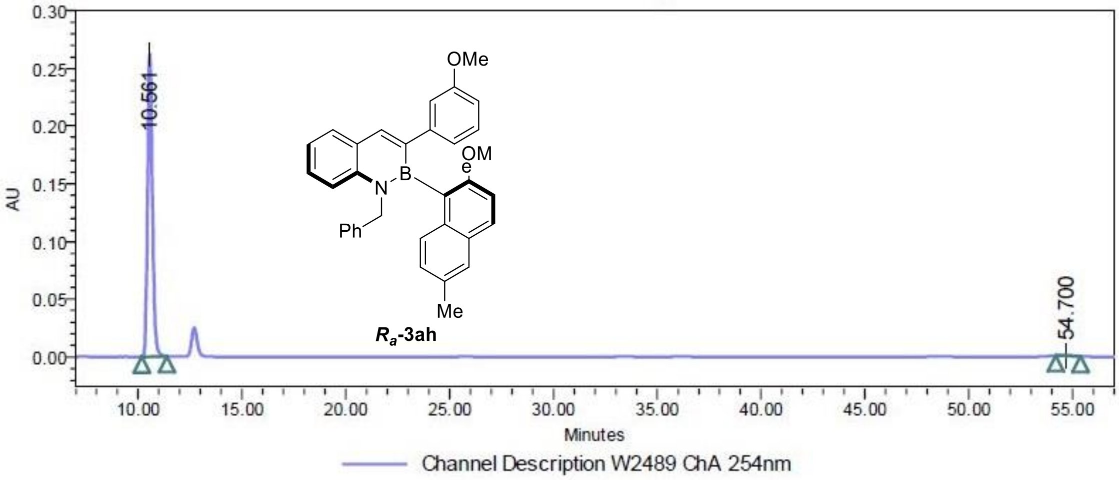

Peak Results

	Retention Time (min)	Int Type	Width (sec)	Area (µV*sec)	Height (µV)	% Area
1	10.561	bb	73.000	4289091	262270	99.62
2	54.700	bb	71.000	16353	374	0.38

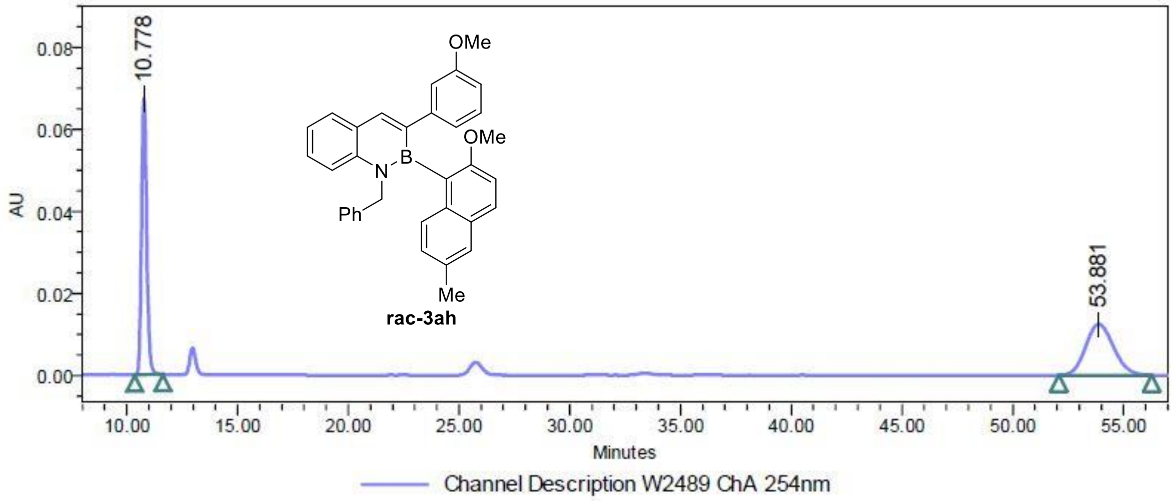

Peak Results

	Retention Time (min)	Int Type	Width (sec)	Area (µV*sec)	Height (µV)	% Area
1	10.778	Bb	76.000	1097923	67478	50.09
2	53.881	bb	251.000	1093870	12419	49.91

Supplementary Figure 271. Chiral HPLC analysis of 3ah

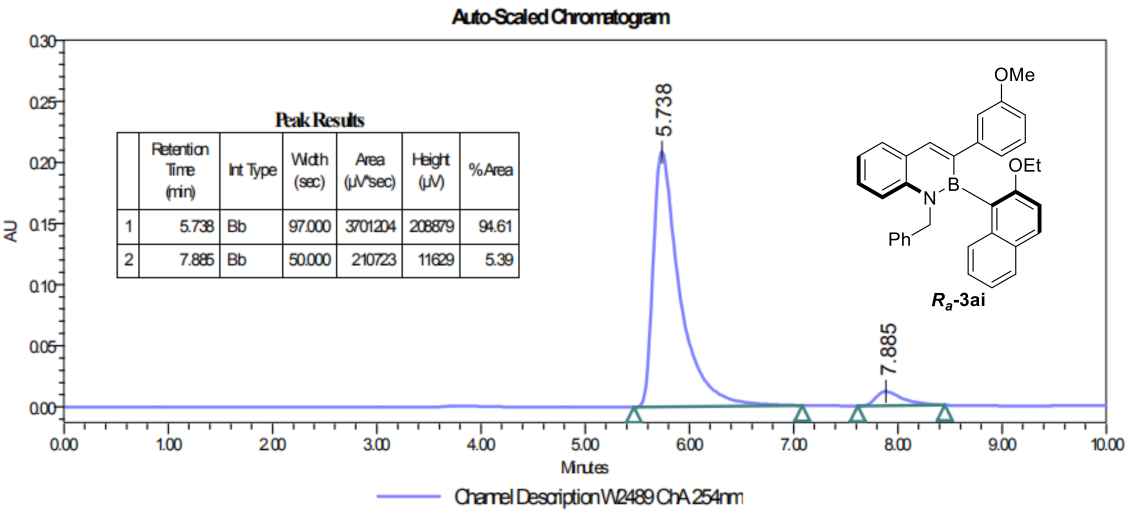

Peak Results

	Retention Time (min)	Int Type	Width (sec)	Area (µV*sec)	Height (µV)	% Area
1	5.738	Bb	97.000	3701204	208879	94.61
2	7.885	Bb	50.000	210723	11629	5.39

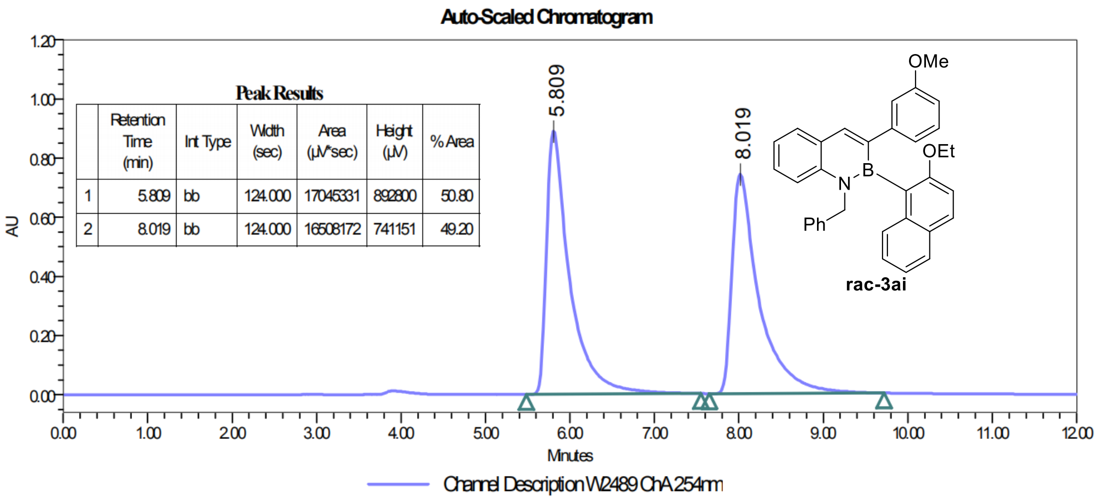

Peak Results

	Retention Time (min)	Int Type	Width (sec)	Area (µV*sec)	Height (µV)	% Area
1	5.809	bb	124.000	17045331	892800	50.80
2	8.019	bb	124.000	16508172	741151	49.20

Supplementary Figure 272. Chiral HPLC analysis of 3ai

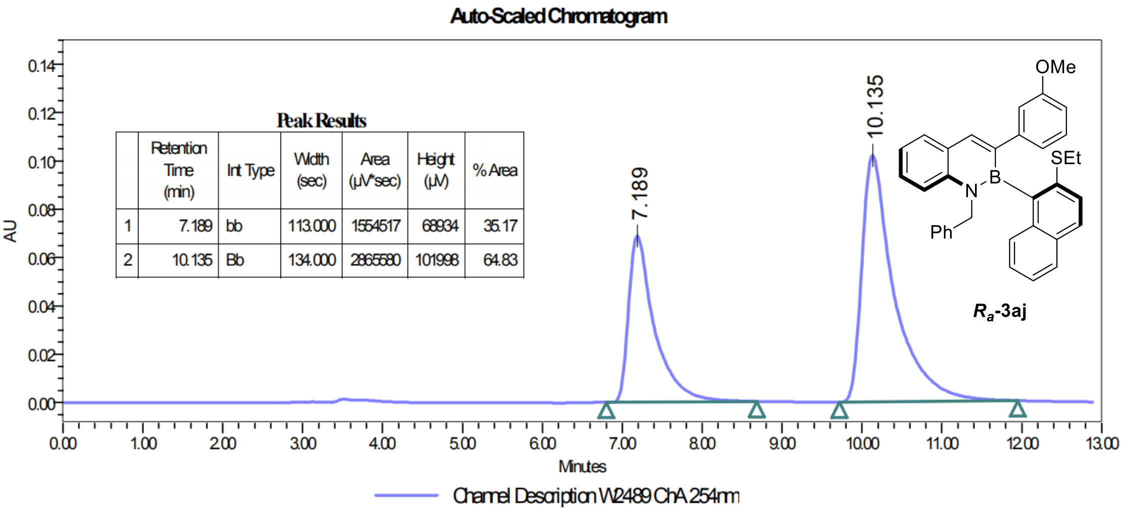

Peak Results

	Retention Time (min)	Int Type	Width (sec)	Area (µV*sec)	Height (µV)	% Area
1	7.189	bb	113.000	1554517	68934	35.17
2	10.135	Bb	134.000	2865580	101998	64.83

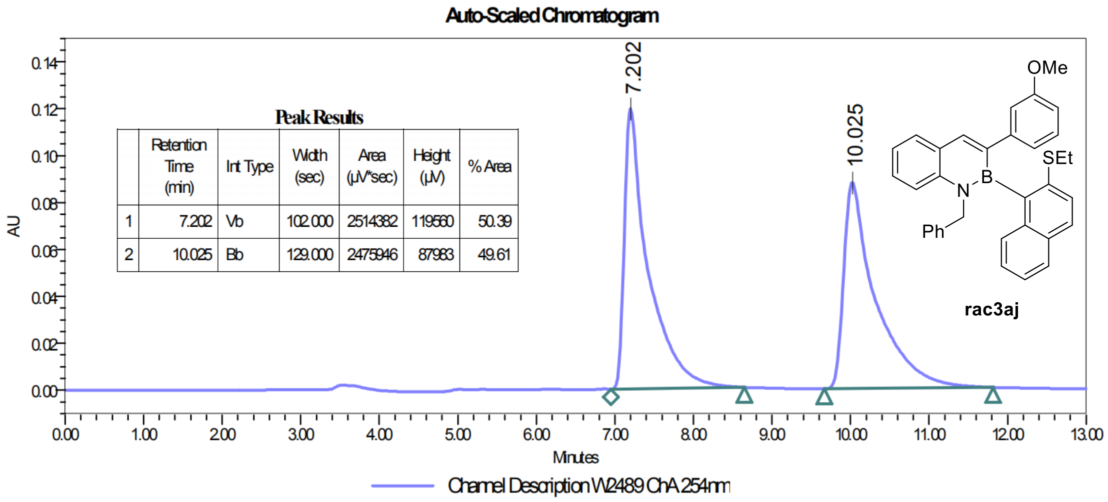

Peak Results

	Retention Time (min)	Int Type	Width (sec)	Area (µV*sec)	Height (µV)	% Area
1	7.202	Vb	102.000	2514382	119560	50.39
2	10.025	Bb	129.000	2475946	87983	49.61

Supplementary Figure 273. Chiral HPLC analysis of 3aj

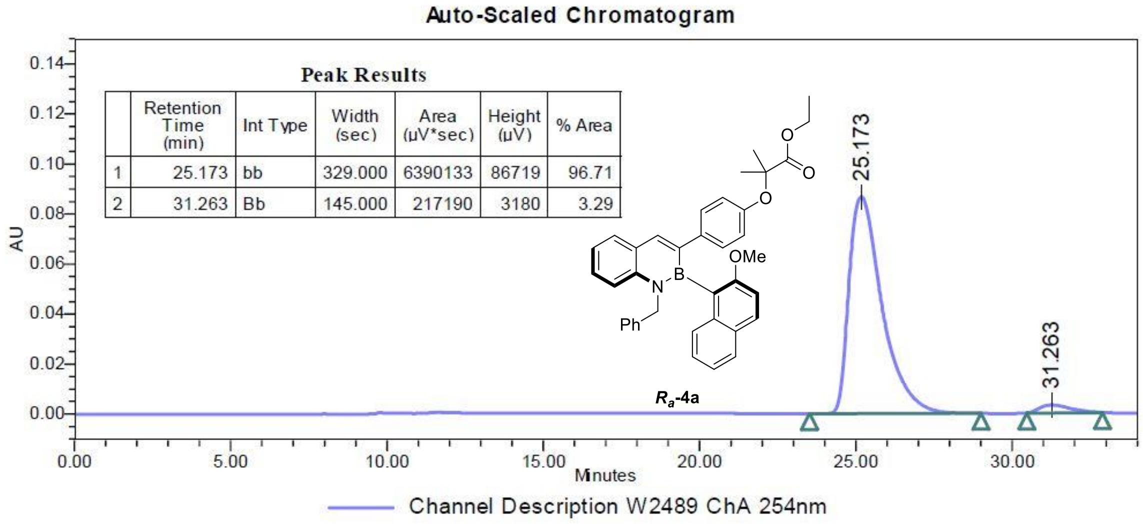

Peak Results

	Retention Time (min)	Int Type	Width (sec)	Area (μV*sec)	Height (μV)	% Area
1	25.173	bb	329.000	6390133	86719	96.71
2	31.263	Bb	145.000	217190	3180	3.29

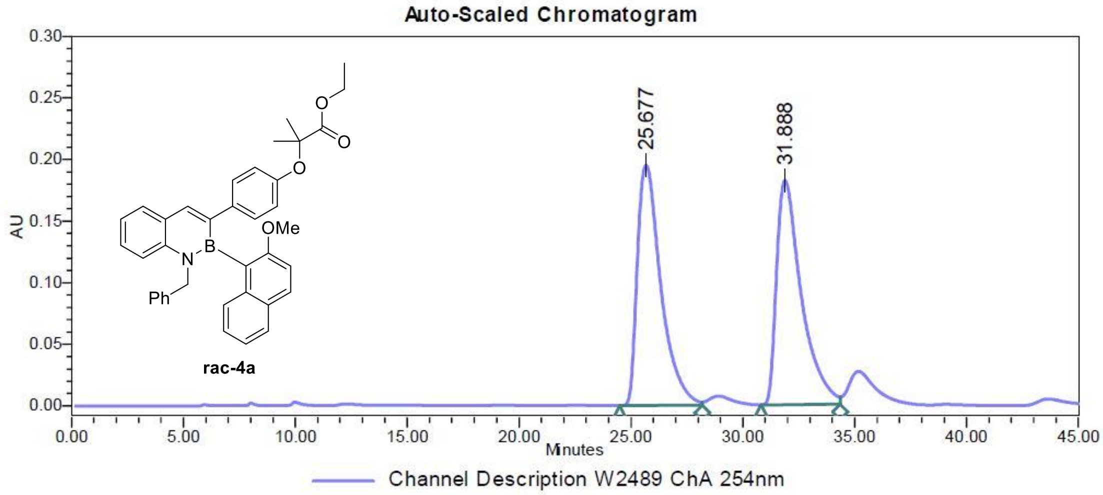

Peak Results

	Retention Time (min)	Int Type	Width (sec)	Area (μV*sec)	Height (μV)	% Area
1	25.677	BV	221.000	14341763	195038	50.43
2	31.888	BV	212.000	14098849	182133	49.57

Supplementary Figure 274. Chiral HPLC analysis of 4a

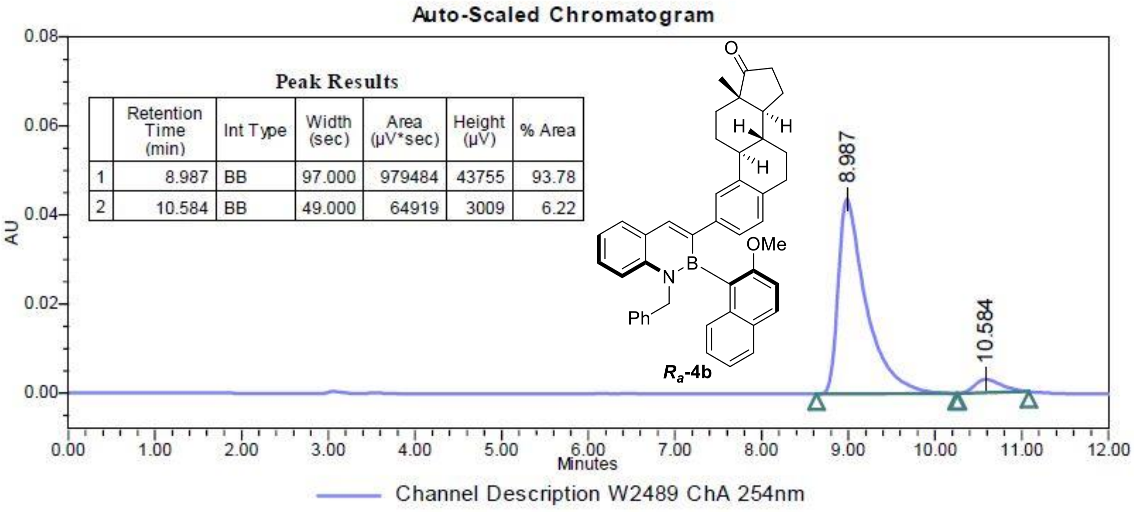

Peak Results

	Retention Time (min)	Int Type	Width (sec)	Area (µV*sec)	Height (µV)	% Area
1	8.987	BB	97.000	979484	43755	93.78
2	10.584	BB	49.000	64919	3009	6.22

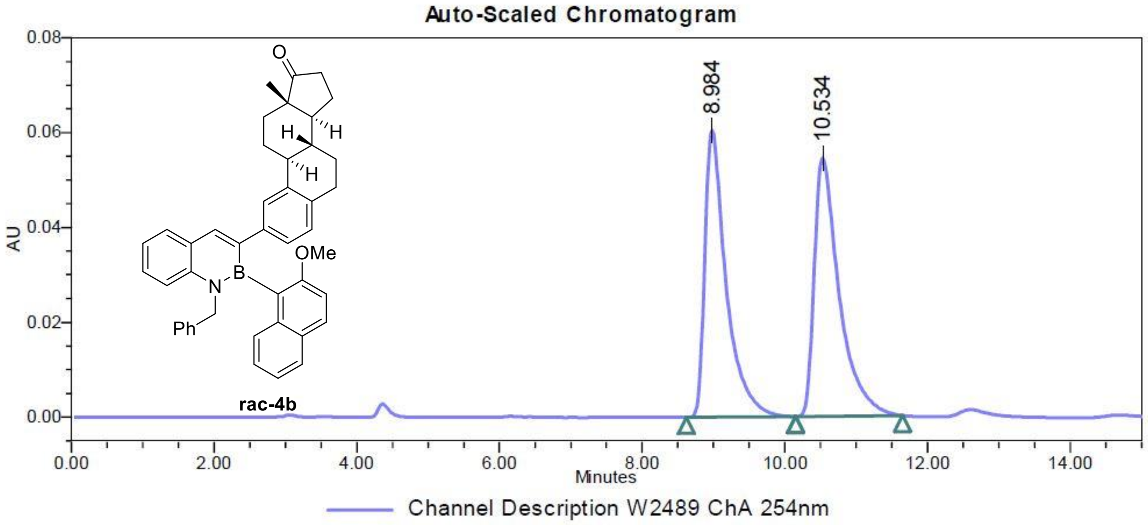

Peak Results

	Retention Time (min)	Int Type	Width (sec)	Area (µV*sec)	Height (µV)	% Area
1	8.984	BB	92.000	1258696	60486	48.81
2	10.534	BB	90.000	1320029	54429	51.19

Supplementary Figure 275. Chiral HPLC analysis of 4b

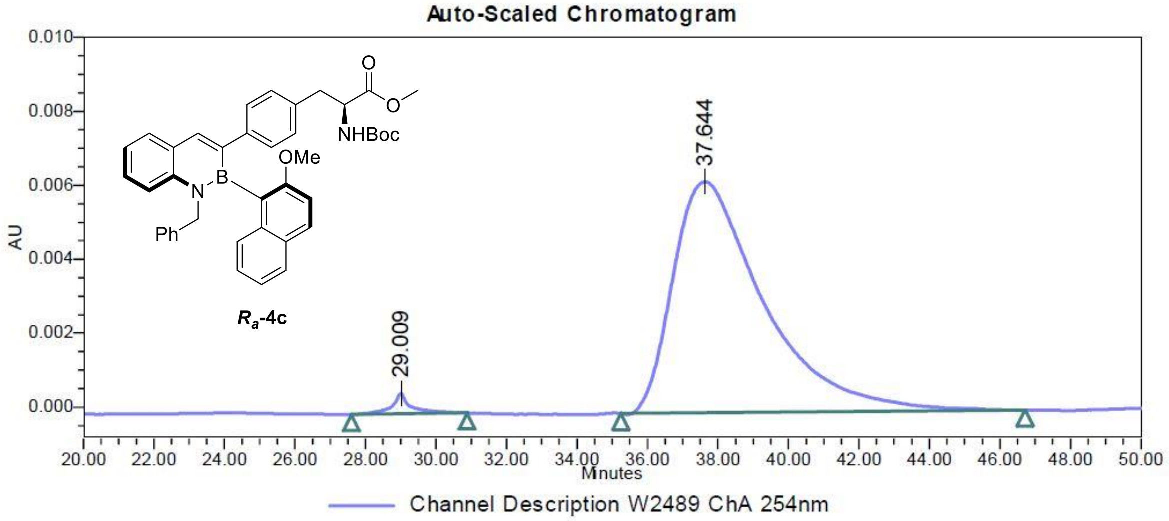

Peak Results

	Retention Time (min)	Int Type	Width (sec)	Area (µV*sec)	Height (µV)	% Area
1	29.009	bb	197.000	21439	555	1.88
2	37.644	bb	688.000	1120942	6243	98.12

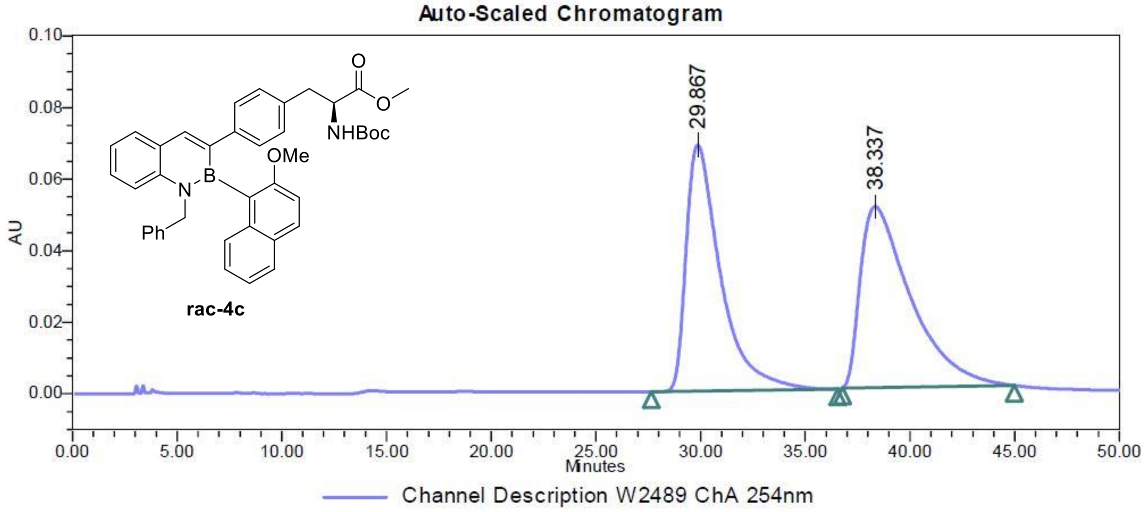

Peak Results

	Retention Time (min)	Int Type	Width (sec)	Area (µV*sec)	Height (µV)	% Area
1	29.867	bb	534.000	7863224	68804	48.08
2	38.337	bb	492.000	8491392	50598	51.92

Supplementary Figure 276. Chiral HPLC analysis of 4c

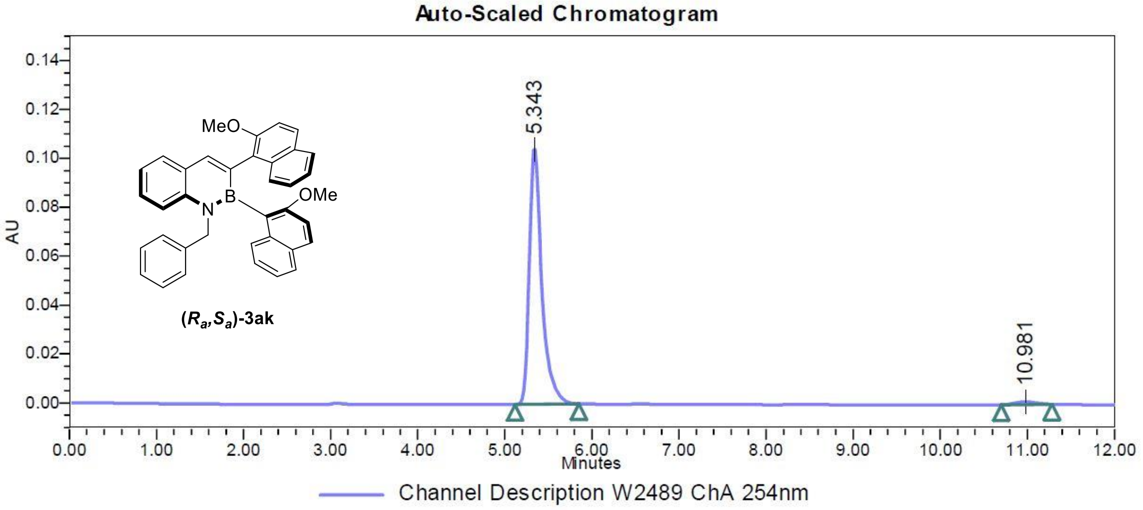

Peak Results

	Retention Time (min)	Int Type	Width (sec)	Area (µV*sec)	Height (µV)	% Area
1	5.343	BB	44.000	1045051	104695	98.30
2	10.981	BB	35.000	18105	1116	1.70

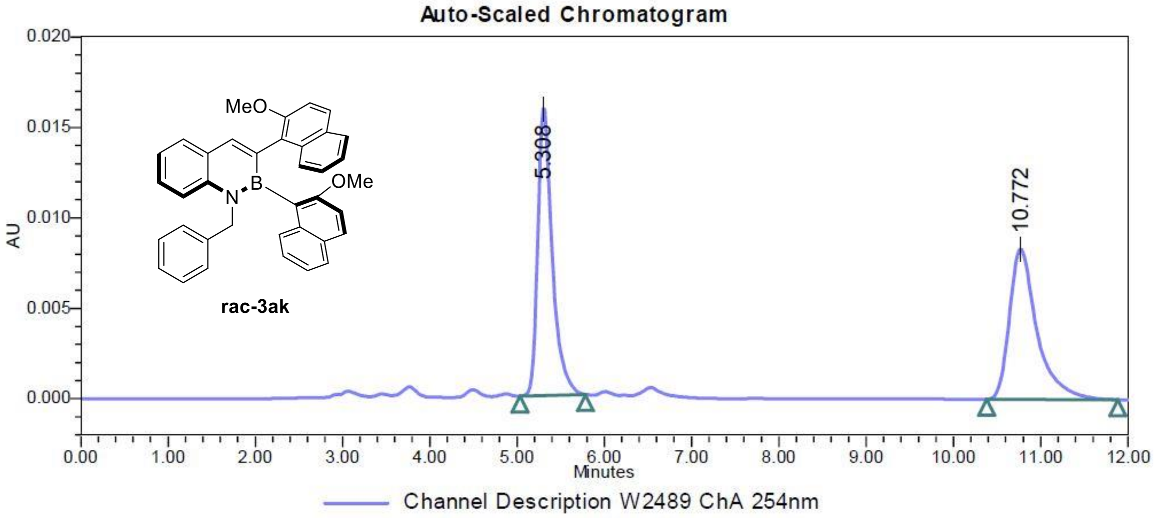

Peak Results

	Retention Time (min)	Int Type	Width (sec)	Area (µV*sec)	Height (µV)	% Area
1	5.308	BB	45.000	183936	15955	50.13
2	10.772	Bb	90.000	182947	8319	49.87

Supplementary Figure 277. Chiral HPLC analysis of 3ak

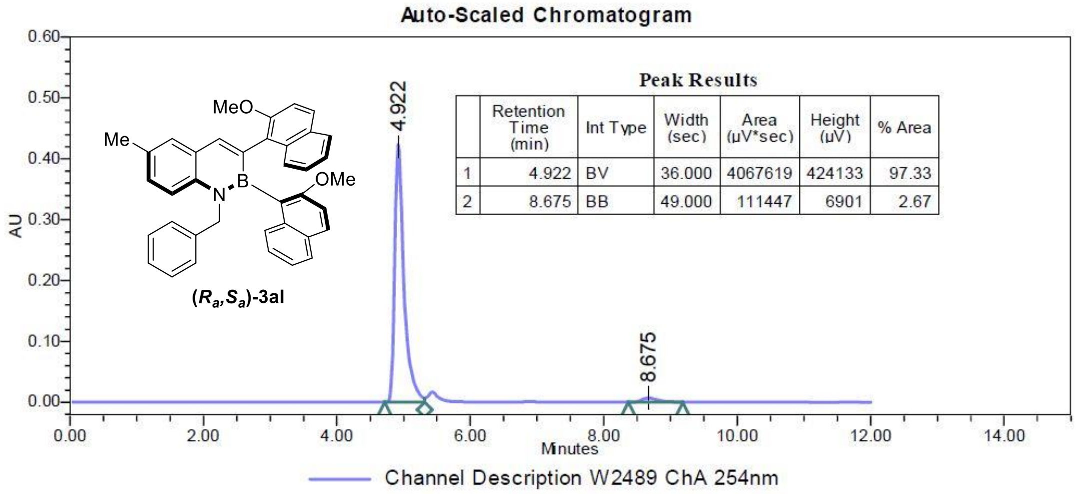

Peak Results

	Retention Time (min)	Int Type	Width (sec)	Area (µV*sec)	Height (µV)	% Area
1	4.922	BV	36.000	4067619	424133	97.33
2	8.675	BB	49.000	111447	6901	2.67

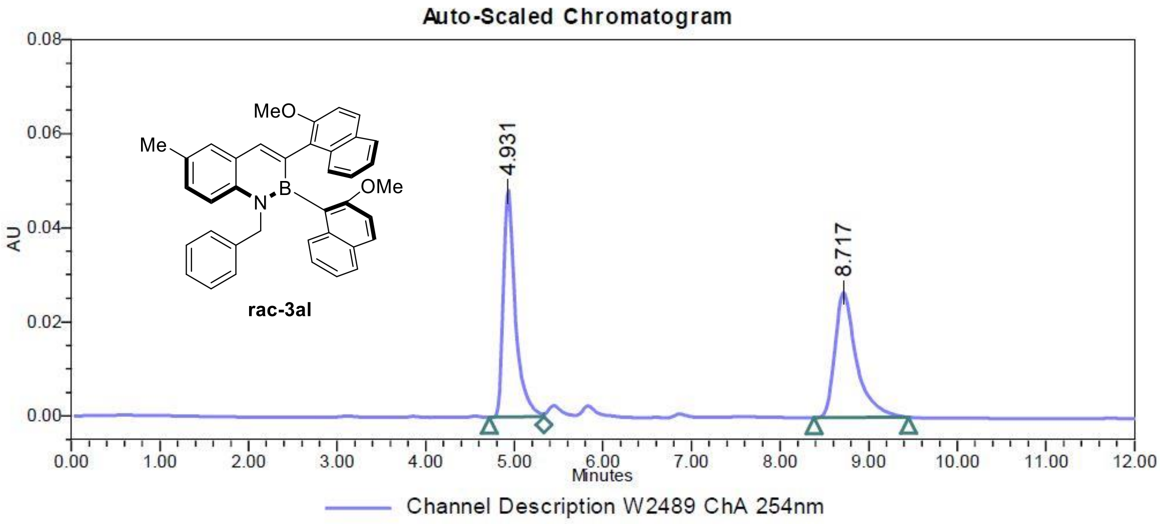

Peak Results

	Retention Time (min)	Int Type	Width (sec)	Area (µV*sec)	Height (µV)	% Area
1	4.931	BV	37.000	455613	48041	50.29
2	8.717	BB	64.000	450351	26552	49.71

Supplementary Figure 278. Chiral HPLC analysis of 3al

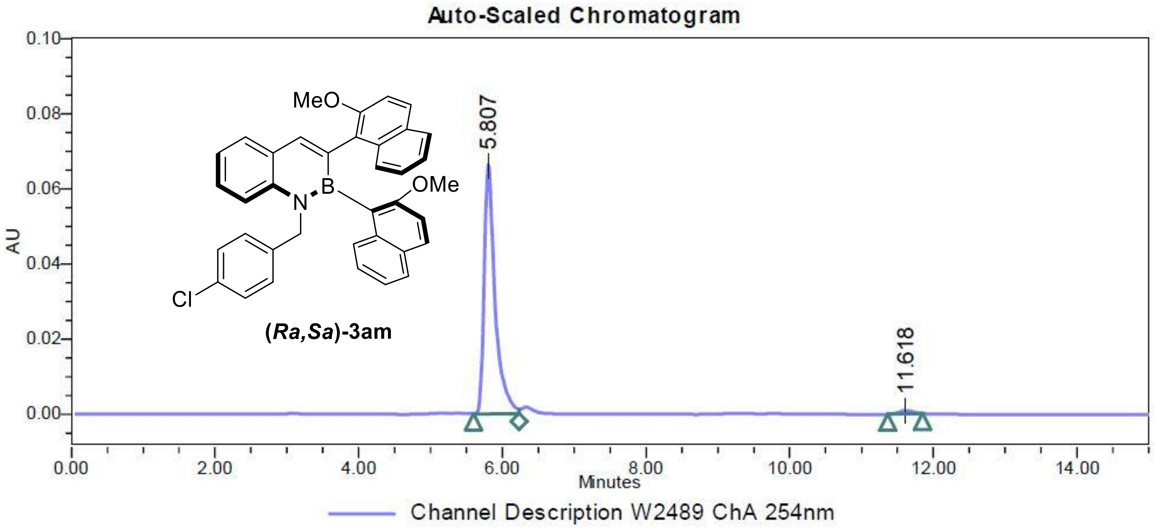

Peak Results

	Retention Time (min)	Int Type	Width (sec)	Area (µV*sec)	Height (µV)	% Area
1	5.807	BV	38.000	703849	66584	98.34
2	11.618	BB	29.000	11864	874	1.66

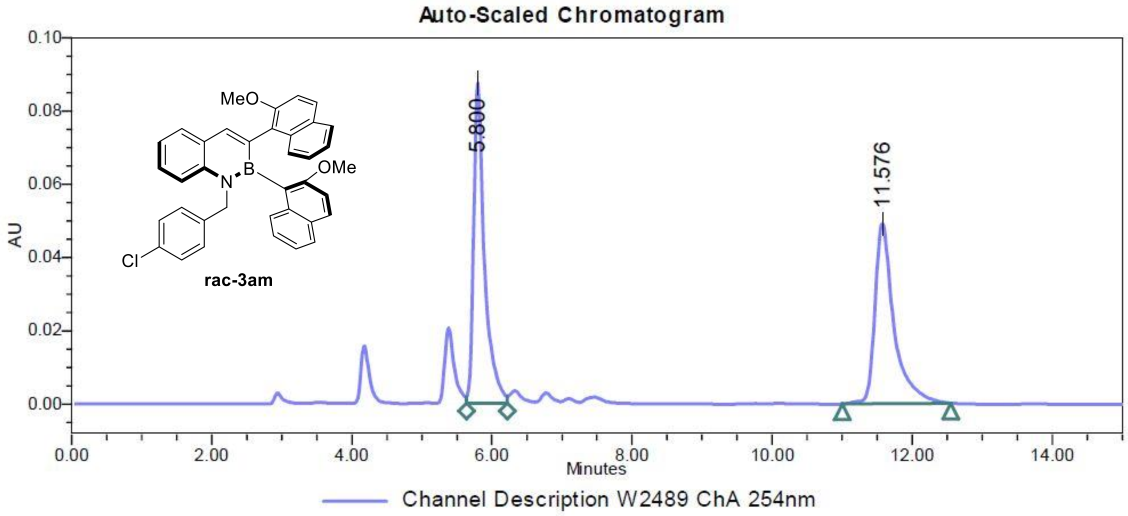

Peak Results

	Retention Time (min)	Int Type	Width (sec)	Area (µV*sec)	Height (µV)	% Area
1	5.800	VV	35.000	946557	87251	50.47
2	11.576	BB	93.000	928878	49159	49.53

Supplementary Figure 279. Chiral HPLC analysis of 3am

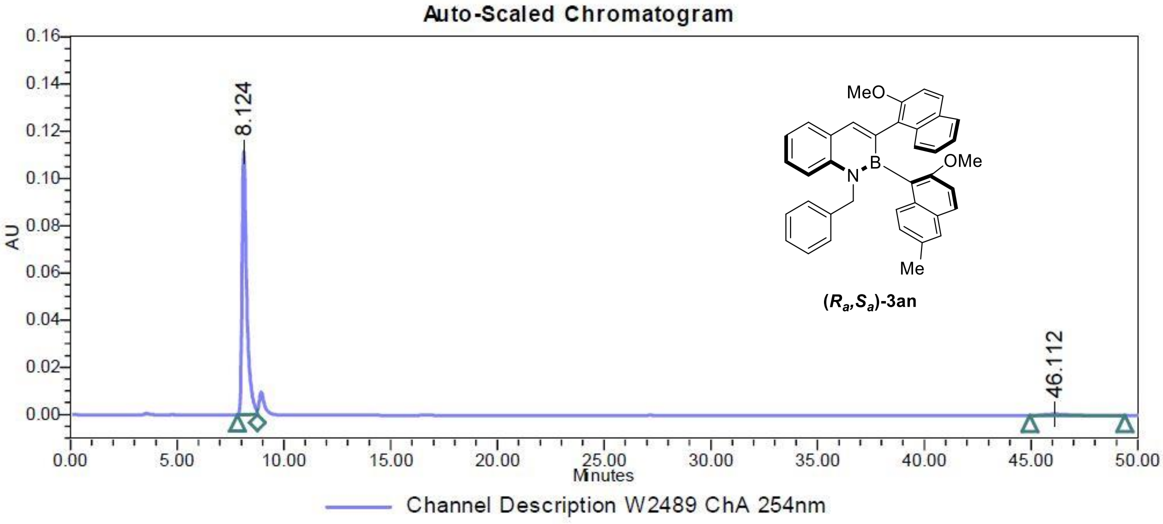

Peak Results

	Retention Time (min)	Int Type	Width (sec)	Area (μV*sec)	Height (μV)	% Area
1	8.124	BV	56.000	1740224	112072	97.34
2	46.112	bb	267.000	47518	602	2.66

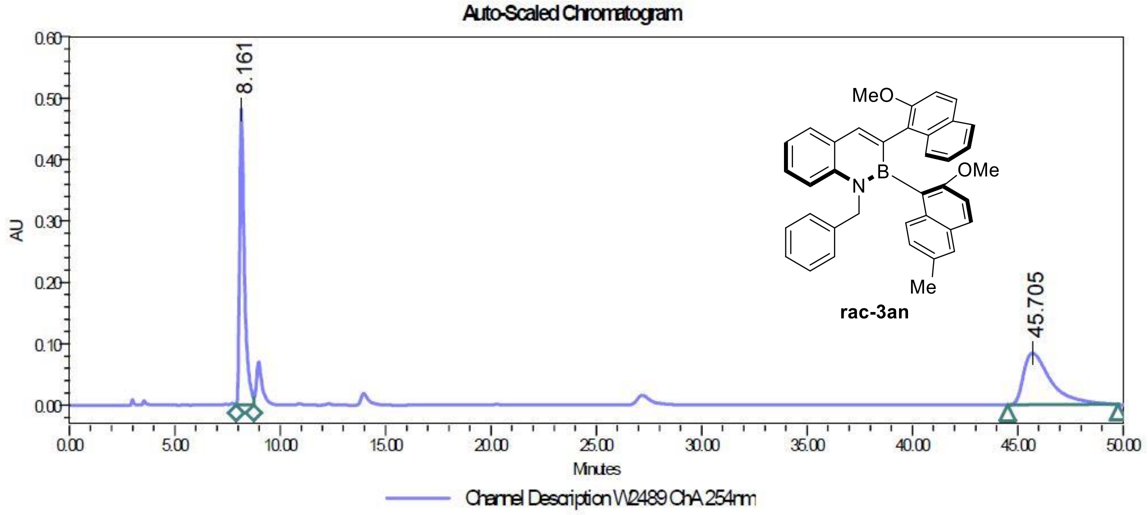

Peak Results

	Retention Time (min)	Int Type	Width (sec)	Area (μV*sec)	Height (μV)	% Area
1	8.161	VV	51.000	7684278	482578	50.06
2	45.705	Bb	314.000	7665392	84146	49.94

Supplementary Figure 280. Chiral HPLC analysis of 3an

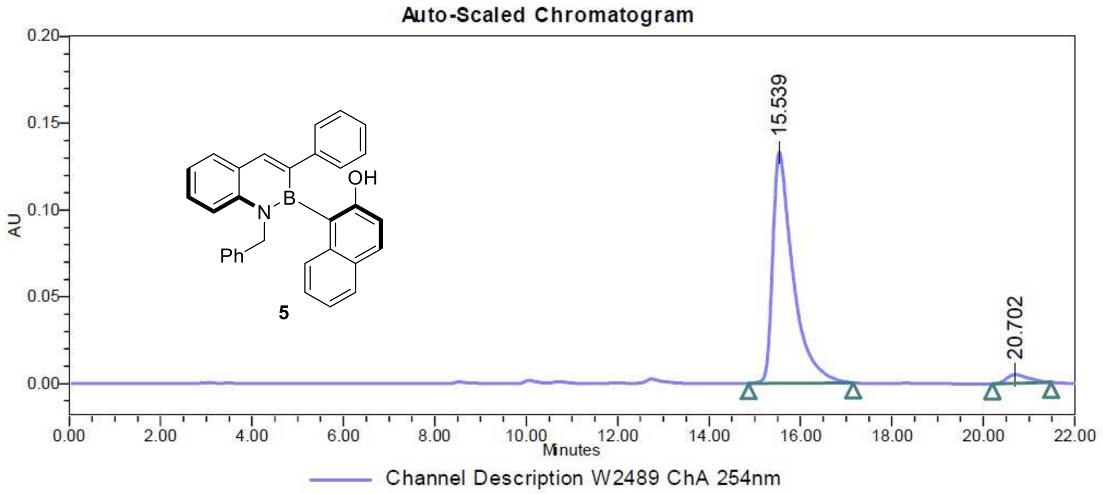

Peak Results

	Retention Time (min)	Int Type	Width (sec)	Area (µV*sec)	Height (µV)	% Area
1	15.539	BB	137.000	4100304	133339	96.06
2	20.702	BB	77.000	168033	4994	3.94

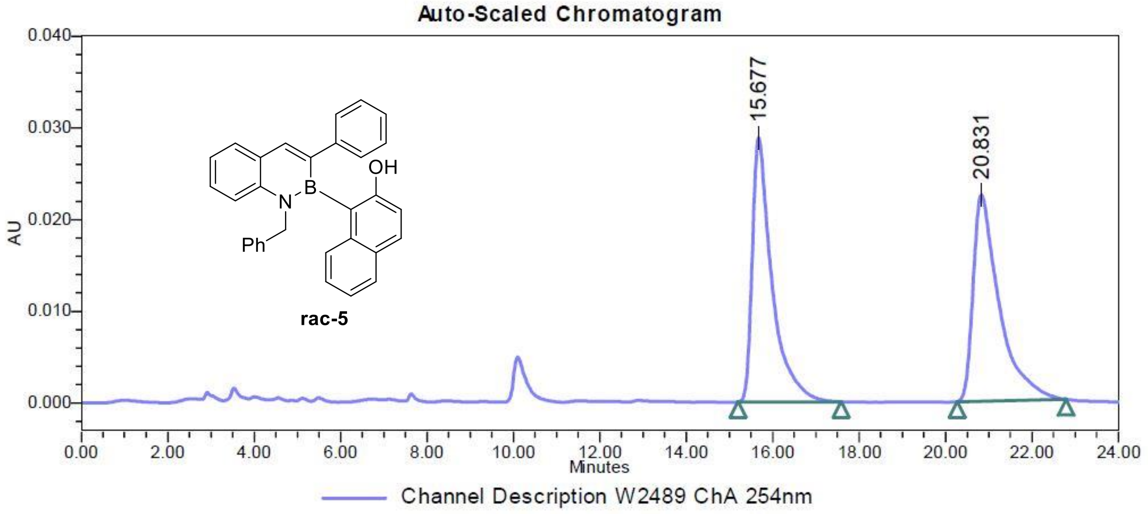

Peak Results

	Retention Time (min)	Int Type	Width (sec)	Area (µV*sec)	Height (µV)	% Area
1	15.677	Bb	143.000	912593	28839	49.04
2	20.831	Bb	151.000	948492	22522	50.96

Supplementary Figure 281. Chiral HPLC analysis of 5

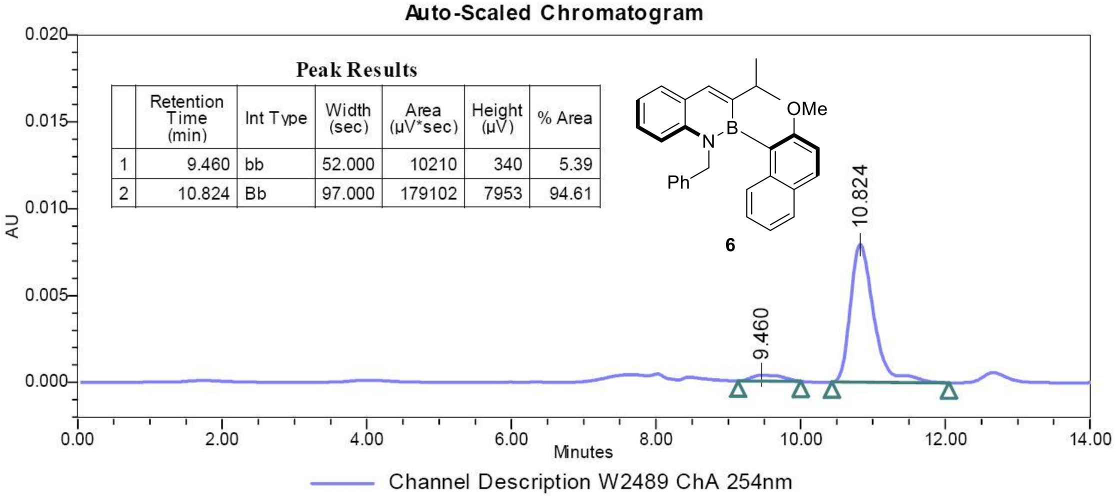

Peak Results

	Retention Time (min)	Int Type	Width (sec)	Area (µV*sec)	Height (µV)	% Area
1	9.460	bb	52.000	10210	340	5.39
2	10.824	Bb	97.000	179102	7953	94.61

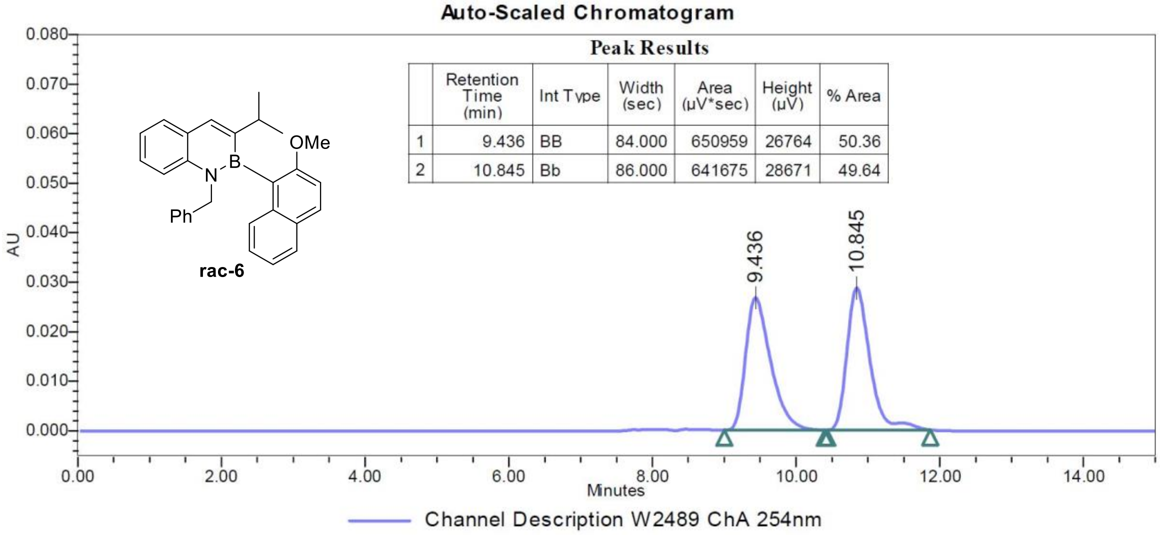

Peak Results

	Retention Time (min)	Int Type	Width (sec)	Area (µV*sec)	Height (µV)	% Area
1	9.436	BB	84.000	650959	26764	50.36
2	10.845	Bb	86.000	641675	28671	49.64

Supplementary Figure 282. Chiral HPLC analysis of 6

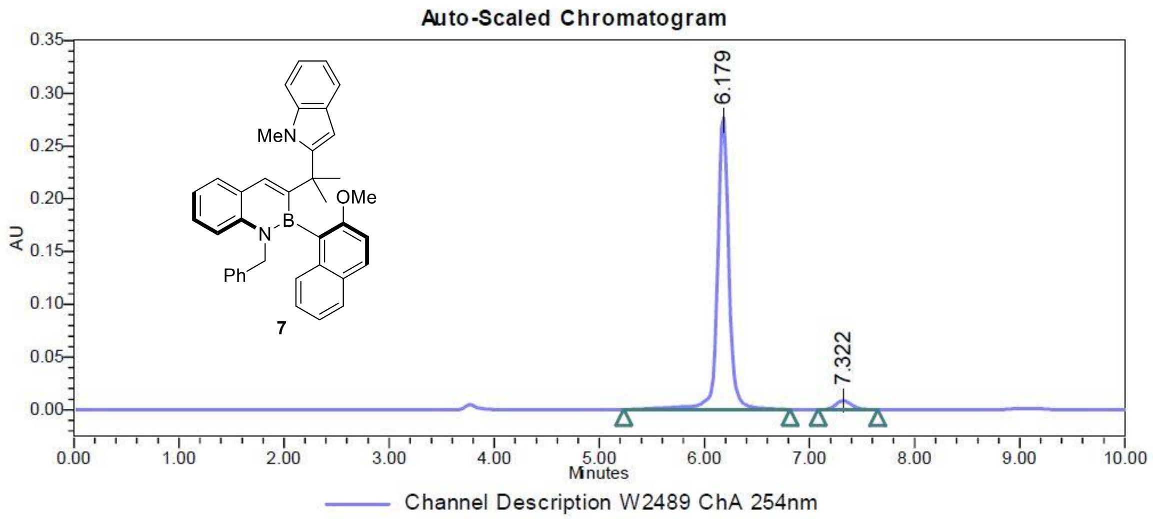

Peak Results

	Retention Time (min)	Int Type	Width (sec)	Area (µV*sec)	Height (µV)	% Area
1	6.179	BB	95.000	2098019	276174	95.87
2	7.322	BB	34.000	90327	8569	4.13

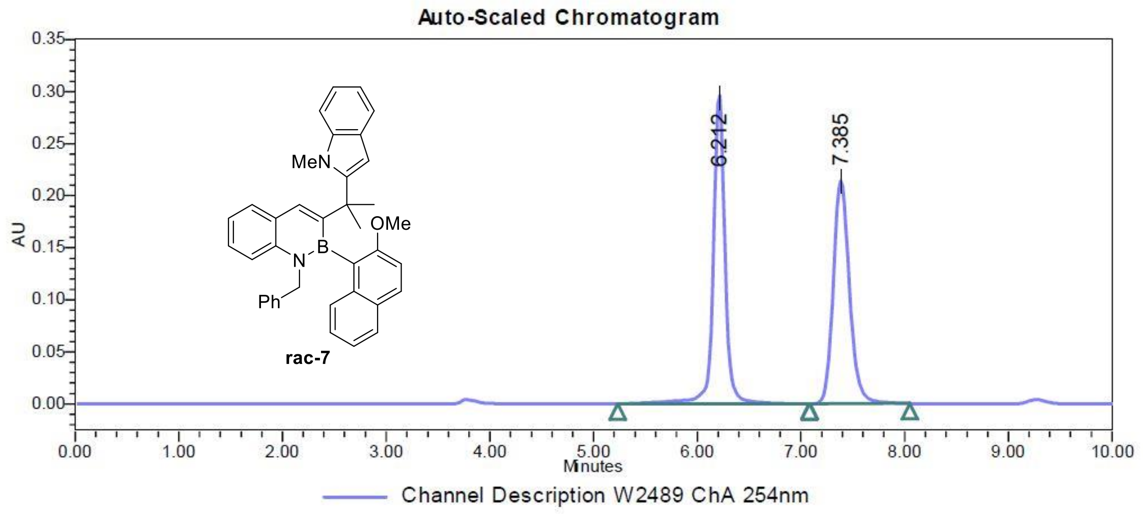

Peak Results

	Retention Time (min)	Int Type	Width (sec)	Area (µV*sec)	Height (µV)	% Area
1	6.212	BB	111.000	2333375	296039	49.99
2	7.385	BB	58.000	2334673	213980	50.01

Supplementary Figure 283. Chiral HPLC analysis of 7

5 Supplementary References

1. Frisch, M. J., Trucks, G. W., Schlegel, H. B., Scuseria, G. E., Robb, M. A., Cheeseman, J. R., Scalmani, G., Barone, V., Petersson, G. A., Nakatsuji, H., Li, X., Caricato, M., Marenich, A. V., Bloino, J., Janesko, B. G., Gomperts, R., Mennucci, B., Hratchian, H. P., Ortiz, J. V., Izmaylov, A. F., Sonnenberg, J. L., Williams, Ding, F., Lipparini, F., Egidi, F., Goings, J., Peng, B., Petrone, A., Henderson, T., Ranasinghe, D., Zakrzewski, V. G., Gao, J., Rega, N., Zheng, G., Liang, W., Hada, M., Ehara, M., Toyota, K., Fukuda, R., Hasegawa, J., Ishida, M., Nakajima, T., Honda, Y., Kitao, O., Nakai, H., Vreven, T.,Throssell, K., Montgomery Jr., J. A., Peralta, J. E., Ogliaro, F., Bearpark, M. J., Heyd, J. J., Brothers, E. N., Kudin, K. N., Staroverov, V. N., Keith, T. A., Kobayashi, R., Normand, J., Raghavachari, K., Rendell, A. P., Burant, J. C., Iyengar, S. S., Tomasi, J., Cossi, M., Millam, J. M., Klene, M., Adamo, C., Cammi, R., Ochterski, J. W., Martin, R. L., Morokuma, K., Farkas, O., Foresman, J. B., & Fox, D. J. *Gaussian 16 Rev. A.03*, Wallingford, CT, 2016.
2. Becke, A. D. Density-functional thermochemistry. III. The role of exact exchange. *J. Chem. Phys.* **98**, 5648-5652 (1993).
3. Grimme, S., Antony, J., Ehrlich, S. & Krieg, H. A consistent and accurate ab initio parametrization of density functional dispersion correction (DFT-D) for the 94 elements H-Pu. *J. Chem. Phys.***,** 132, 154104 (2010).
4. Barone, V. & Cossi, M. Quantum Calculation of Molecular Energies and Energy Gradients in Solution by a Conductor Solvent Model. *J. Phys. Chem. A***, 102**, 1995-2001 (1998).
5. Cossi, M., Rega, N., Scalmani, G. & Barone, V. Energies, structures, and electronic properties of molecules in solution with the C-PCM solvation model. *J. Comput. Chem.* **24**, 669-681 (2003).
6. Weigend, F. & Ahlrichs, R. Balanced basis sets of split valence, triple zeta valence and quadruple zeta valence quality for H to Rn: Design and assessment of accuracy. *Phys. Chem. Chem. Phys.***, 7**, 3297-3305 (2005).
7. Bannwarth, C., Ehlert, S. & Grimme, S. GFN2-xTB—An Accurate and Broadly Parametrized Self-Consistent Tight-Binding Quantum Chemical Method with Multipole Electrostatics and Density-Dependent Dispersion Contributions. *J. Chem. Theory Comput.* **15**, 1652-1671 (2019).
8. Zhao, Y. & Truhlar, D. G. The M06 suite of density functionals for main group thermochemistry, thermochemical kinetics, noncovalent interactions, excited states, and transition elements: two new functionals and systematic testing of four M06-class functionals and 12 other functionals. *Theor. Chem. Acc.* **120**, 215-241 (2008).
9. Fukui, K. The path of chemical reactions-the IRC approach. *Acc. Chem. Res.* **14**, 363-368 (1981).
10. Schäfer, A., Huber, C. & Ahlrichs, R. Fully optimized contracted Gaussian basis sets of triple zeta valence quality for atoms Li to Kr. **100**, 5829-5835 (1994).
11. Chai, J.-D. & Head-Gordon, M. Long-range corrected hybrid density functionals with damped atom–atom dispersion corrections. *Phys. Chem. Chem. Phys.* **10**, 6615-6620 (2008).
12. Marenich, A. V., Cramer, C. J. & Truhlar, D. G. Universal solvation model based on solute electron density and on a continuum model of the solvent defined by the bulk dielectric constant and atomic surface tensions. *J. Phys. Chem. B***, 113**, 6378-6396 (2009).
