# Supplementary material for: Construction of C-B axial chirality via dynamic kinetic asymmetric cross-coupling mediated by tetracoordinate boron
Source: Nat Commun. 2023 Jul 24;14:4438. doi: 10.1038/s41467-023-40164-6 (PMC10366327; doi:10.1038/s41467-023-40164-6)
Supplement: Supplementary file 3 — Description of Additional Supplementary Files [file 41467_2023_40164_MOESM3_ESM.pdf]

Description of Additional Supplementary Files

File Name: Supplementary Data 1

Description: Crystallographic data for compound **3a** (CCDC: 2245394).
